# Highly efficient multiplex human T cell engineering without double-strand breaks using Cas9 base editors

**DOI:** 10.1038/s41467-019-13007-6

**Published:** 2019-11-19

**Authors:** Beau R. Webber, Cara-lin Lonetree, Mitchell G. Kluesner, Matthew J. Johnson, Emily J. Pomeroy, Miechaleen D. Diers, Walker S. Lahr, Garrett M. Draper, Nicholas J. Slipek, Branden A. Smeester, Klaus N. Lovendahl, Amber N. McElroy, Wendy R. Gordon, Mark J. Osborn, Branden S. Moriarity

**Affiliations:** 1 Department of Pediatrics, University of Minnesota, Minneapolis, MN USA; 2 Masonic Cancer Center, University of Minnesota, Minneapolis, MN USA; 3 Center for Genome Engineering, University of Minnesota, Minneapolis, MN USA; 4 Stem Cell Institute, University of Minnesota, Minneapolis, MN USA; 5 Department of Biochemistry, Molecular Biology, and Biophysics, University of Minnesota, Minneapolis, MN USA

**Keywords:** Genetic engineering, Immunotherapy, CRISPR-Cas9 genome editing

## Abstract

The fusion of genome engineering and adoptive cellular therapy holds immense promise for the treatment of genetic disease and cancer. Multiplex genome engineering using targeted nucleases can be used to increase the efficacy and broaden the application of such therapies but carries safety risks associated with unintended genomic alterations and genotoxicity. Here, we apply base editor technology for multiplex gene modification in primary human T cells in support of an allogeneic CAR-T platform and demonstrate that base editor can mediate highly efficient multiplex gene disruption with minimal double-strand break induction. Importantly, multiplex base edited T cells exhibit improved expansion and lack double strand break-induced translocations observed in T cells edited with Cas9 nuclease. Our findings highlight base editor as a powerful platform for genetic modification of therapeutically relevant primary cell types.

## Abstract (web summary)

Multiplexed genome engineering with Cas9 can increase efficiency but also the risk of unintended alterations. Here the authors demonstrate the use of multiplexed base editors on primary T cells with reduced translocation frequency.

## Introduction

Chimeric antigen receptor engineered T cell (CAR-T) immunotherapy has shown efficacy against a subset of hematological malignancies^1,2^, yet its autologous nature and ineffectiveness against epithelial and solid cancers limit widespread application. To overcome these limitations, targeted nucleases have been used to disrupt checkpoint inhibitors and genes involved in alloreactivity^3–6^. However, the production of allogeneic, “off-the-shelf” T cells with enhanced function requires multiplex genome editing strategies that risk off-target (OT) effects, chromosomal rearrangements, and genotoxicity due to simultaneous double-strand break (DSB) induction at multiple loci^7–10^. Moreover, it has been well documented that DSBs are toxic lesions that can drive genetic instability^11,12^. Alternatively, CRISPR-Cas9 base editors afford programmable enzymatic nucleotide conversion at targeted loci without induction of DSBs^13,14^. We reasoned this technology could be used to knockout gene function in human T cells while minimizing safety concerns associated with current nuclease platforms. Through systematic reagent and dose optimization, we demonstrate highly efficient multiplex base editing and consequent protein knockout in primary human T cells at loci relevant to the generation of allogeneic CAR-T cells including the T-cell receptor ɑ constant (*TRAC*) locus, β-2 microglobulin (*B2M*), and programmed cell death 1 (*PDCD1*). Multiplex base edited T cells equipped with a CD19 CAR kill target cells more efficiently; and importantly, both DSB induction and translocation frequency are greatly reduced compared with cells engineered with Cas9 nuclease. Collectively, our results establish base editor as an efficient multiplex gene editing platform to enhance both the safety and efficacy of engineered T cell-based immunotherapies.

## Results

Base editing has been previously used to induce premature stop (pmSTOP) codons for gene knockout in mice and in mammalian cells^15–18^, and to induce exon skipping by disrupting splice acceptor (SA) sites^19^. However, we reasoned that splice-site disruption, including splice donor (SD) sites, could also be effective for gene knockout and may have several advantages over induction of pmSTOP codons (Supplementary Fig. 1). For instance, stop codon readthrough has been shown to occur at frequencies up to 31% in some genes, and can be promoted under conditions of cellular stress^20,21^. Splice-site editing mitigates this concern as it alters gene processing at the RNA level^22^, which is less likely to be bypassed at the translational level. In addition, current base editors do not produce strict C to T edits, with even the most recent base editors producing up to 25% nontarget editing (C to G/A)^23^. In the context of pmSTOP, nontarget edits preclude pmSTOP codon formation, thereby lowering the efficiency of protein knockout, and instead create potentially undesirable amino acid changes.

**Fig. 1:**
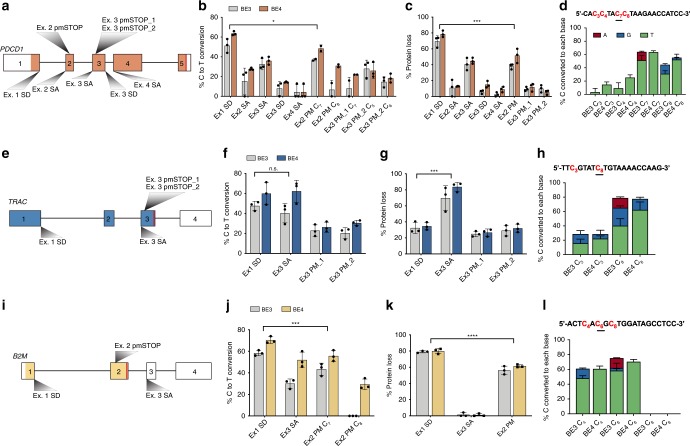
Assessment of guide RNA activity for gene disruption at *PDCD1*, *TRAC, and B2M*. **a** Diagram of *PDCD1* locus indicating the relative locations of each sgRNA. Colored portion of boxes represent protein-coding region, vertical red line indicates stop codon. **b** Quantification of C to T conversion of target base for each *PDCD1* sgRNA (*n* = 3 independent T-cell donors). **c** PDCD1 protein knockout frequency (*n* = 3 independent T-cell donors). **d** Quantification of C to T/A/G conversion at all Cs within the detected editing window (shown in red) of the *PDCD1* Ex1 SD sgRNA (n = 3 independent T-cell donors). Underlined C indicates target nucleotide critical for proper splicing. **e** Diagram of *TRAC* locus indicating the relative locations of each sgRNA. **f** Quantification of C to T conversion at target base for each *TRAC* sgRNA (*n* = 3 independent T-cell donors). **g** TRAC protein knockout frequency as determined by flow cytometry for CD3 loss (*n* = 3 independent T-cell donors). **h** Quantification of C to T/A/G conversion at all cytosines within the detected editing window (shown in red) of the *TRAC* Ex3 SA sgRNA (*n* = 3 independent T-cell donors). **i** Diagram of *B2M* locus indicating the relative locations of each sgRNA. **j** Quantification of C to T conversion of target base for each *B2M* sgRNA (*n* = 3 independent T-cell donors). **k** B2M protein knockout frequency (*n* = 3 independent T-cell donors). **l** Quantification of C to T/A/G conversion at all cytosines within the detected editing window (shown in red) of the *B2M* Ex1 SD sgRNA (data represented as mean ± SD, *n* = 3 independent biological T-cell donors). *P*-values calculated by the Student’s paired two-tailed *t* test between the highest-editing guide and the second highest-editing treatment (n.s. *P* > 0.05, *P ≤ 0.05, **P ≤ 0.01, ***P ≤ 0.001, ****P ≤ 0.0001)

To assess the performance of both pmSTOP introduction and splice-site disruption, we designed a panel of single guide RNAs (sgRNA) to convert amino acid codons to pmSTOPs or to disrupt SD and SA sequences within *PDCD1, TRAC*, and *B2M* (Fig. 1a, e, i; Supplemental Table 1). Individual sgRNAs were co-delivered as chemically modified RNA oligonucleotides^24^ with first-generation BE3^13^ or BE4^23^ mRNA to T cells by electroporation. Target C to T editing rates were assessed by Sanger sequencing and EditR, an analysis software developed by our group to expedite and economize analysis of base editing at the genetic level^25^ (baseeditr.com).

First, we targeted the checkpoint gene *PDCD1* (PD-1) by designing eight sgRNAs; three of which were predicted to introduce pmSTOP codons, two targeted disruption of SD sites (GT:**C**A), and three targeted disruption of SA sites (AG:T**C**) (Fig. 1a). We found that co-delivery of sgRNAs with BE3 or BE4 mRNA mediated measurable editing of target Cs at all target loci, with several candidate sgRNAs exhibiting significantly higher rates of editing than others (Fig. 1b, Supplementary Fig. 2). Specifically, we found that targeting the SD site of *PDCD1* exon 1 resulted in the highest rate of target C to T editing with both BE3 (51.3 ± 7.0%, M ± SD) and BE4 (63.7 ± 2.1%) mRNA (Fig. 1b). The next two most efficient sgRNAs targeted the exon 3 SA site (32.6 ± 5.5% for BE3; 36.0 ± 4.0% for BE4) and a candidate pmSTOP site in exon 2 (37.1 ± 1.2% for BE3; 48.5 ± 3.7% for BE4) (Fig. 1b). To determine whether genetic editing results in protein loss we assessed expression of PD-1 protein by flow cytometry. Concordant with our genetic analysis, targeting *PDCD1* exon 1 SD resulted in the highest rate of protein loss (69.5 ± 7.0% for BE3; 78.6 ± 4.1% for BE4), followed by exon 3 SA (40.6 ± 7.8% for BE3; 44.7 ± 3.8% for BE4), and exon 2 pmSTOP (37.9 ± 3.4% for BE3; 51.5 ± 9.0% for BE4) (Fig. 1c).

Informed by our *PDCD1* results, we designed a focused panel of sgRNAs targeting *TRAC* (Fig. 1e). Here we found that C to T conversion was highest at the exon 1 SD site (47.6 ± 4.6% for BE3; 60.0 ± 11.3% for BE4) and exon 3 SA site (40.3 ± 9.7% for BE3; 62.3 ± 11.0% for BE4), with BE4 exhibiting higher editing rates than BE3 at each target (Fig. 1f). Efficient editing was also observed at two pmSTOP candidate sites in exon 3, albeit at lower efficiencies than that of either splice-site disrupting sgRNA (Fig. 1f). Both the exon 1 SD and exon 3 SA sites were edited at similar frequencies, yet disruption of the exon 3 SA site resulted in the highest rate of TCR disruption as measured by loss of cell-surface CD3 expression (69.0 ± 15.3% for BE3; 83.7 ± 5.8% for BE4) (Fig. 1g).

We next targeted *B2M* using a similar strategy (Fig. 1i). BE4 mRNA delivered with an sgRNA targeting the exon 1 SD site showed the most efficient C to T conversion of the target base (58.3 ± 2.5% for BE3; 70.3 ± 3.2% for BE4) (Fig. 1j), resulting in efficient knockout of B2M protein (79.1 ± 1.3% for BE3; 80.0 ± 3.2% for BE4) (Fig. 1k). We also identified a candidate pmSTOP site in exon 2 that resulted in relatively efficient C to T editing (43.3 ± 5.7% for BE3; 55.7 ± 5.0% for BE4), and protein knockout (56.2 ± 5.1% for BE3; 61.5 ± 1.8% for BE4) (Fig. 1j, k). Notably, targeting the SA site of noncoding exon 3 produced efficient C to T editing but did not result in a detectable reduction in protein expression (Fig. 1j, k).

Nontarget editing (i.e. C to A or G) has been reported for BE3^13^ and is reduced with BE4, which contains a second uracil glycosylase inhibitor (UGI) fused in series at the C-terminus^23^. We evaluated nontarget editing rates for all Cs within the editing window (predominantly bases 4–8 of protospacer) of our most efficient sgRNAs with BE3 and BE4. As expected, BE4 showed reduced nontarget editing compared with BE3 at all loci (−14% ± 6.6%, *P* < 2.2e-16, Paired one-way *t*-test) (Fig. 1d, h, l; Supplementary Fig. 2). Despite having only nickase function, low-level indel formation has been observed with both BE3 and BE4^13,23^. Thus, we used next-generation sequencing (NGS) to measure indel frequency at all target sites after editing (Supplementary Fig. 3). Indels were detectable with both BE3 and BE4 at levels that varied based upon target site. Consistent with prior publications, BE4 exhibited an overall reduced indel frequency (−4.8 ± 6.1%, *P* < 4.6e-16, Paired one-way t-test) (Supplementary Fig. 3)^23^.

**Fig. 2:**
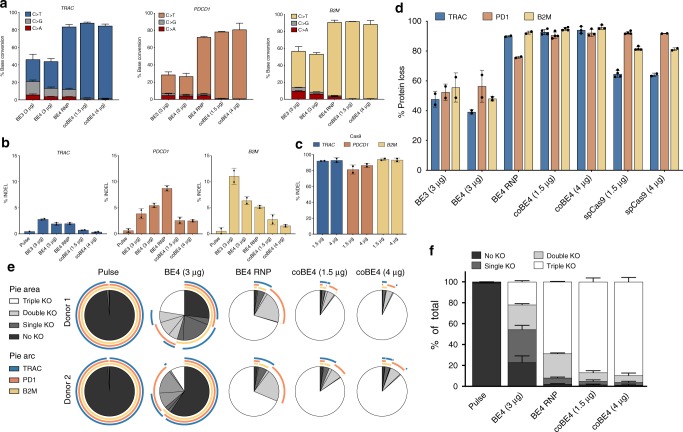
Optimization of multiplex editing using optimal sgRNAs (TRAC Ex3 SA, B2M Ex1 SD, and PD-1 Ex1 SD). **a** Conversion frequency of target cytosine to all other bases at *TRAC, PDCD1*, and *B2M* as analyzed by NGS following co-delivery of three target sgRNA with first-generation BE3 or BE4 mRNA; BE4 protein complexed with sgRNA (BE4 RNP); or codon optimized BE4 (coBE4) mRNA. **b** Indel frequency at *TRAC, PDCD1*, and *B2M* as analyzed by NGS following co-delivery of three target sgRNA with first-generation BE3 or BE4 mRNA; BE4 RNP; or coBE4 mRNA. **c** Indel frequency at *TRAC, PDCD1*, and *B2M* as analyzed by NGS following co-delivery of three target sgRNA and SpCas9 nuclease mRNA. **d** Frequency of TRAC, PD-1, and B2M protein loss measured by flow cytometry seven days post electroporation. **e** SPICE representation of multiplex flow cytometric analysis performed seven days post electroporation. **f** Quantification of fractions of WT, single, double, and triple gene KO. Data represented as mean ± SD, *n* = 2–4 independent biological T-cell donors

Toward our goal of validating a multiplex editing strategy that could be utilized to generate allogeneic, “off-the-shelf” T cells with enhanced function, we co-delivered our top sgRNA for each gene along with first-generation BE3 or BE4 mRNA. Surprisingly, knockout efficiency at each target was substantially reduced for both BE3 and BE4 when delivered in a multiplex setting (Supplementary Fig. 4). To determine if the reduced editing efficiency was due to low protein levels, we delivered equal doses of BE3, BE4, and nuclease active *Streptococcus pyogenes* Cas9 (SpCas9) mRNA to T cells and measured protein expression at 24 h after electroporation. Strikingly, while SpCas9 protein expression was readily detectable, BE3 and BE4 protein were undetectable (Supplementary Fig. 5). To address this issue, we first delivered BE3 and BE4 mRNA at a dose 2× higher (3 µg) than that used in our initial multiplex experiments (1.5 µg). This strategy improved editing efficiency at each locus, but the efficiencies were still lower than those observed in our single gene targeting experiments (Fig. 2a).

During the course of these experiments, independent reports emerged identifying problems related to the use of first-generation BE3 and BE4 expression vectors that severely reduce both transcriptional and translational efficiency in human cells^17,26^. To circumvent these issues, we delivered purified BE4 protein as a ribonucleoprotein (RNP) complex with our most effective sgRNA for each target. By optimizing our electroporation protocol for RNP delivery, we found that BE4 RNP mediated improved editing efficiency over a 2x dose of first-generation BE4 mRNA (Fig. 2a, *P* = 0.0052, paired one-way *t-*test). Next, we codon optimized the sequence of BE4 (coBE4) and observed increased protein expression when delivered as mRNA (Supplementary Fig. 5). When coBE4 mRNA was delivered at both our standard dose (1.5 µg) and a higher dose (4 µg) with all three of our optimal sgRNAs, we achieved substantially higher rates of multiplex target C to T editing at all three loci across multiple independent T-cell donors, exceeding 90% in some instances (Fig. 2a, *P* = 0.0018, paired one-way *t* test). Nontarget editing observed with first-generation BE3 and BE4 mRNA was reduced slightly when using BE4 RNP, and even further reduced with both doses of coBE4 mRNA (Fig. 2a). We next evaluated the rate of indel formation at each target site after multiplex base editing and, in accordance with previous studies, found lower rates of indel formation at each site with all forms of BE compared with SpCas9 nuclease (Fig. 2b, c). Both low and high doses of coBE4 mRNA exhibited the lowest overall frequency of indel formation at all sites examined (Fig. 2b).

Multiplex protein knockout was analyzed for each target gene by flow cytometry, and the frequency of protein loss correlated well with genetic editing frequencies (Fig. 2d, *r* = 0.90, *t* = 10.932, df = 28, *P* = 1.3e−11, paired two-way *t*-test). BE4 RNP demonstrated more efficient protein knockout than first-generation BE3 and BE4 mRNA, yet coBE4 mRNA was most efficient, exceeding 90% protein loss for each gene at both low and high mRNA doses. This rate is on par with, and even exceeds, protein KO by SpCas9 at some targets (Fig. 2d).

At the mRNA level, disruption of *PDCD1* and *B2M* exon 1 SD sites resulted in near complete loss of transcript, presumably via nonsense mediated decay caused by intron retention (Supplementary Fig. 7)^27–29^. Disruption of *TRAC* exon 3 SA did not reduce overall transcript levels, but instead caused robust skipping of exon 3 which encodes a portion of the connecting peptide as well as the transmembrane and cytoplasmic domains of TCRɑ and is essential for TCR assembly and function (Supplementary Fig. 7)^30^.

A key consideration of multiplex editing is the resultant proportion of cells carrying each potential combination of gene knockout. To better understand this phenomenon in our experiments, we evaluated protein expression of all target genes simultaneously by flow cytometry and used SPICE analysis to determine the proportion of individual cells having no knockout, single gene knockout, double gene knockout, or triple gene knockout; as well as the combination of proteins lost within each of these fractions (Fig. 2e). While first-generation BE4 mRNA generated an endpoint cell population with a diverse combination of knockout phenotypes, the frequency of triple knockout cells was low (21.9 ± 1.1%). The proportion of triple knockout cells was substantially higher using BE4 RNP (68.6 ± 0.37%), and even further increased with coBE4 mRNA at 1.5 µg (86.6 ± 3.75%) and 4 µg (89.57 ± 4.2%) (Fig. 2f).

OT DSB induction is an important challenge facing nuclease platforms^31^. To evaluate the specificity of our optimal sgRNAs for each target, we utilized computational prediction as well as unbiased GUIDE-seq (GS) to identify OT editing^31^. Optimal sgRNAs were delivered individually with SpCas9 nuclease or BE4 mRNA and editing at the top 10 predicted OT sites was evaluated by NGS (Supplemental Table 2). No editing was observed at any of the computationally predicted *B2M* or *TRAC* OT sites in either the SpCas9 nuclease or BE4 treatment conditions (Supplementary Fig. 8). At the predicted *PDCD1* OT sites we observed a single OT edit with an indel frequency of 13.1% using SpCas9 mRNA (Supplementary Fig. 8). Strikingly, C to T editing at this site was only 0.9% with BE4 mRNA, and indel formation was near the low detection limit of our assay (0.1%) (Supplementary Fig. 8). To identify potential OT sites in an unbiased fashion, we conducted GS analysis in both primary T cells and U2OS. We observed successful OnT oligo integration in both cell types and identified 21 candidate OT sites across our three optimal sgRNAs (Supplementary Data 9a–c**)**. Only 4/21 candidate sites aligned to >10 reads, and these were the only candidate sites shared between primary T cells and U2OS. The OT site with the highest number of GS reads, *PDCD1* GS_OT1 (1919 primary T cell; 811 U2OS), was the validated OT site (OT2) identified computationally (Supplementary Fig. 8). Editing at the remaining three GS OT sites was near or below the limit of detection by NGS (Supplementary Fig. 9d).

Recent reports on base editing in immortalized cell lines have established that cells enriched for plasmid-based overexpression of BE3 have transient, sgRNA-independent OT editing of RNA transcripts at conserved APOBEC1 motifs^32,33^. Such OT editing could have potentially deleterious impacts on cellular function in a therapeutic setting. To investigate OT RNA editing in multiplex edited T cells, we delivered coBE4 mRNA with and without our three optimal sgRNAs and measured RNA editing at 12, 24, and 48 h post electroporation using the program MultiEditR (https://moriaritylab.shinyapps.io/multieditr/)^34^. We used a rational approach that accounts for transcripts with OT RNA editing observed in the original reports^32^, as well as the expression of these transcripts in activated T cells (DICE, dice-database.org) in order to select five candidates with a high probability of RNA editing (Supplementary Fig. 10a, b)^35^. When analyzing the data across genes (Supplementary Fig. 10c) there was no significant effect of the treatment condition, T cell donor, or time point on the observed estimates of RNA editing (One-way repeated measures ANOVA, *P* > 0.10, *N* = 26). These data suggest that if OT RNA editing is present, it is at a level below the ~7% limit of detection of quantitative analysis of Sanger sequencing^25^.

**Fig. 3:**
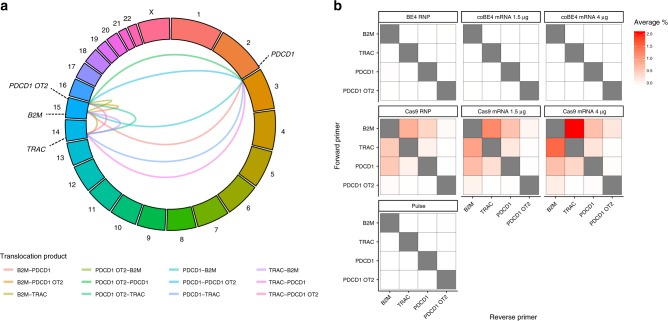
Translocation frequencies in multiplex edited T cells. **a** Circos plot of possible translocation outcomes resulting from double-strand break induction at *TRAC, B2M, PDCD1*, and *PDCD1* OT site. **b** Droplet-digital PCR quantification of translocation frequencies. All assays run in technical duplicate across *n* = 2 independent biological T-cell donors

Nuclease-mediated multiplex editing has also been reported to generate undesired translocations in human T cells^3^. As base editing substantially reduces the frequency of DSB formation, we reasoned that translocations should likewise be reduced using our base editing approach. To test our hypothesis, we used droplet-digital PCR (ddPCR) to quantify the frequency of 12 possible translocation outcomes predicted to occur between our three target loci and the single identified OT site (Fig. 3a). Following co-delivery of our three optimal sgRNAs with either SpCas9 nuclease RNP or mRNA, we were able to detect all 12 predicted translocation outcomes at varying frequencies using ddPCR, with a subset of the most frequent translocations confirmed by fluorescent in situ hybridization (FISH) (Fig. 3b, Supplementary Figs. 11 and 12**)**. In all cases, SpCas9 mRNA resulted in the highest rate of translocation, with translocations between *TRAC* and *B2M* being most frequent (2.04 ± 0.09%) (Fig. 3b). In stark contrast, translocation outcomes between our three target loci were virtually undetectable in cell populations receiving BE4 RNP or either dose of coBE4 mRNA with our optimal sgRNAs (Fig. 3b, Supplementary Fig. 11). In a single replicate from one donor, the *PDCD1*:*B2M* assay gave rise to two positive droplets with low-dose coBE4 mRNA (calculated frequency = 0.003 ± 0.006%). Because no positive droplets were detected with BE4 RNP or high-dose coBE4 mRNA, these may be artifactual. Translocations between our three target loci and the *PDCD1* OT site were also detected in SpCas9-treated T cells, albeit at a lower frequency, while BE4 treated samples showed no signals above the detection limit (0.01%) of our assay (Fig. 3b, Supplementary Fig. 12).

**Fig. 4:**
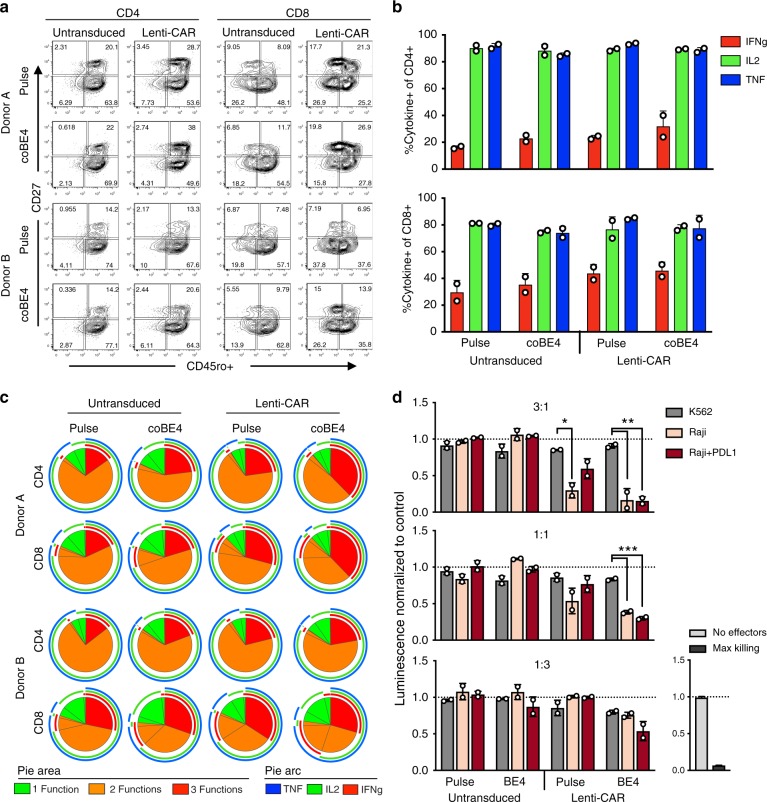
Function of multiplex edited T cells. **a** Expression of the memory marker CD27 and CD45ro following editing and expansion. Production of cytokines individually (**b**) and in combination (**c**) by CD4 and CD8 T cells following activation. **d** Ability of T cells to kill CD19^neg^ K562, CD19^pos^ Raji cells, or CD19^pos/^PD-L1^pos^ Raji cells as measured by luciferase luminescence assay following co-culture with T cells. Graph titles indicate E:T ratio. Data represented as mean ± SD, with assays run in triplicate in *n* = 2 independent biological T-cell donors. *P*-values calculated by the Student’s paired two-tailed *t* test (n.s. *P* > 0.05, *P ≤ 0.05, **P ≤ 0.01, ***P ≤ 0.001, ****P ≤ 0.0001)

We next sought to determine whether multiplex knockout T cells generated using our base editing strategy retain cytokine functionality and are capable of mediating target cell killing when equipped with a CAR. We performed phenotypic evaluation of both electroporation pulse control and coBE4 knockout T cells with and without a CD19-specific CAR by analyzing markers of differentiation^36^. Both untransduced and CAR-transduced T cells exhibited similar differentiation phenotypes, with the fractions of effector and memory populations similar between control and coBE4 knockout T cells (Fig. 4a). CAR transduction and cell expansion were also comparable between pulse and coBE4 mRNA groups (Supplementary Fig. 13). Following activation, a high frequency of both untransduced and CAR-transduced coBE4 knockout T cells exhibited robust production of cytokines IL-2, TNFα, and IFNγ (Fig. 4b). Cytokine polyfunctionality was similarly retained following the multiplex editing process (Fig. 4c). Collectively, these data demonstrate that multiplex coBE4 editing combined with CAR transduction did not negatively impact T-cell phenotype or function. Finally, to determine if coBE4 knockout T cells equipped with the CD19 CAR retained the ability to kill target cells, we conducted in vitro co-culture assays with nontarget CD19^neg^/PD-L1^neg^ K562; target CD19^pos^/PD-L1^neg^ Raji; and target CD19^pos^/PD-L1^pos^ Raji engineered to overexpress PD-L1, which would normally act to inhibit killing by T cells expressing cell-surface PD-1. Both control and coBE4 knockout T cells mediated specific killing of CD19^pos^ but not CD19^neg^ target cells (Fig. 4d). However, only coBE4 knockout T cells were able to achieve significant killing of CD19^pos^/PD-L1^pos^ target cells, with the efficiency of killing equivalent to that of CD19^pos^/PD-L1^neg^ target cells (Fig. 4d).

## Discussion

As we come to better understand the requirements for successful cell-based immunotherapy and gene therapy, and as enthusiasm grows for the production of universal, allogeneic cells, highly multiplexed gene editing will likely become commonplace. However, it has been well documented that DSBs are toxic lesions that can drive genomic instability and cell death^11,12^. This is a lesser concern when engineering cells for research but could lead to transformation or reduced function when gene editing cells for therapeutic use. Concerns surrounding DSBs are further heightened in the context of multiplex gene editing where multiple, simultaneous DSBs can compound toxicity. This is highlighted by the parabolic relationship between the number of discrete DSB sites and potential translocation outcomes, such that an editing strategy targeting ten loci could generate 90 potential translocations, not accounting for other potential genomic alterations such as inversions and large deletions. To overcome these issues, we have implemented base editors for multiplex T cell engineering and demonstrate that splice-site disruption through base editing offers an efficient, widely applicable, and safer approach compared with the use of DSB-inducing targeted nucleases.

Interestingly, we find both higher rates of nontarget editing and indel formation when using BE4 RNP compared with coBE4 expressed from transfected mRNA. This observation may be due to the extended BE4 residence time achieved when expressed from mRNA as opposed to direct BE4 protein delivery. As even free UGI has been shown to reduce both indel frequency and nontarget editing in the context of BE3^37^, the extended residence time achieved by mRNA delivery may allow BE4 UGI domains additional capacity to mitigate DSB formation and nontarget editing^37^.

In our current study we utilized lentiviral delivery of CD19-specific CAR, which is the current industry standard in CAR-T therapy. However, this approach has many drawbacks, including the risk of insertional mutagenesis, variable CAR expression, and gene silencing^38–40^. To overcome these issues, a number of groups have demonstrated high efficiency, site-specific integration using Cas9 nuclease along with rAAV-delivered DNA donor templates for homologous recombination (HR). This raises the possibility that BE4 could be deployed to safely and efficiently knockout multiple genes with simultaneous introduction of therapeutic transgenes in a site-specific fashion using rAAV and Cas9 orthologs, such as *Staphylococcus aureus* Cas9 (SaCas9) or *Francisella novella* Cas9 (FnCas9)^41,42^. The application of Cas9 orthologs would allow for simultaneous use of distinct sgRNAs specific to BE4 and Cas9 nuclease without concerns of cross-utilization. Alternatively, it has been demonstrated that DNA nicks can stimulate HR, albeit with lower efficiency, suggesting that BE4 could mediate gene knockout through deaminase activity, while simultaneously directing HR through its nickase function^43^.

One notable difference between the use of base editors and targeted nucleases is the number of potential outcomes from the editing event. Nuclease-mediated DSBs are repaired through the highly variable nonhomologous end joining pathway, resulting in a spectrum of indels; some of which will not introduce frame-shift mutations and will thus have unknown significance to gene expression and function. Alternatively, our base editing approach has a limited number of outcomes, all resulting in the loss of function of the native SD or acceptor, even when considering nontarget editing. Yet it is important to consider that disruption of the native splice site may not always result in a nonfunctional product. In the current study, disruption of SD sites resulted in NMD and loss of transcript; conversely, SA disruption caused skipping of the target exon but no reduction in transcript level. Thus, when targeting SA sites, the editing strategy should prioritize disruption of function-critical exons and account for alternative splicing events that could maintain the biological function of the target gene.

Translocation analysis using small ddPCR amplicons (>200 bp) spanning the sgRNA target site demonstrated that base editing with optimal reagents virtually eliminates detectable translocations, whereas Cas9 nuclease produces numerous translocations, some at frequencies exceeding ~2%. Notably, larger deletions were also identified at the site of translocation through Sanger sequencing of subcloned junction PCR amplicons (~500 bp) from SpCas9-treated cells (Supplementary Fig. 14). These data suggest the presence of more complex genomic rearrangements similar to those reported previously^9,10^ that are not detected by our current ddPCR assays. Considering the variability in efficiency of nucleic acid delivery between cells by electroporation, it is possible that cells receiving high levels of SpCas9/sgRNA may harbor translocations more frequently. Provocatively, and perhaps as a result of eliminating DSB and consequent genomic alterations, T cells exhibited significantly improved expansion following multiplex editing with coBE4 vs. SpCas9 nuclease (Supplementary Fig. 15). Further evaluation will be required to definitively identify the mechanism underlying this phenomenon.

Although we demonstrate that BE4 substantially reduces DSB induction and translocations compared with SpCas9 nuclease, the potential remains for undesirable events to occur. For instance, sgRNA-independent OT deamination of genomic cytosines was reported following BE3 overexpression in mouse zygotes^44^ and rice^45^. In separate studies, sgRNA-independent editing of RNA transcripts was observed in cultured human cells expressing high levels of editor protein, however the corresponding edits were not observed in genomic DNA in this setting^32,33,46^. Interestingly, OT RNA editing was not detected in cells expressing low levels of editor enzyme^32^. This latter observation may explain the absence of detectable OT RNA editing in our study, where BE4 protein was expressed transiently and at relatively low levels. Further studies investigating these potential events should be undertaken prior to clinical translation, though the issue could be largely mitigated by employing recently developed variants shown to have minimal OT RNA editing^33,46^. Despite these areas of uncertainty, the base editor platform represents an effective strategy for highly efficient multiplex engineering of therapeutic primary cells with an improved safety profile compared with current nuclease technologies.

## Methods

### Editing reagents and vector cloning

BE3 (https://www.addgene.org/73021) and BE4 (https://www.addgene.org/100802/) were cloned into pmRNA production vector and mRNA was produced commercially (Trilink Biotechnologies). coBE4 was derived by codon optimization of the BE4 open reading frame followed by gene synthesis (GenScript). The AA sequence of coBE4 is included in the Supplementary Information. coBE4 was subcloned into pmRNA production vector and mRNA was produced commercially by Trilink Biotechnologies. BE4 protein was produced commercially (Aldevron, Fargo, ND). DNA sequences for CD19 chimeric antigen receptor linked by a T2A to RQR8 were synthesized as gBlock Gene Fragments (IDT, Coralville, IA). Fragments were Gibson Assembled^47^ into pRRL (https://www.addgene.org/36247). Gibson reactions were transformed into DH10β *Escherichia coli* and plated on LB agar with ampicillin. Plasmid DNA was purified from colonies using the GeneJET Plasmid Miniprep Kit (ThermoFisher, Waltham, MA). Following confirmation by Sanger sequencing, clones were sent to the University of Minnesota Viral Vector & Cloning Core (VVCC) for production and titration of viral particles.

### Guide RNA design

Guide RNAs (sgRNAs) were designed using the base editing splice-site disruption sgRNA design program SpliceR (https://z.umn.edu/splicer) SpliceR is written in the R statistical programming language (v. 3.4.3). Briefly, SpliceR takes a target Ensembl transcript ID, a base editor PAM variant, and a species as an input. Using the exon and intron sequences from Ensembl, the program extracts the region surrounding every splice site based on a user-specified window. The pattern of N_20_-NGG is then matched to the antisense strand of the extracted sequence. Matched patterns are then scored based on the position of the target motif within the predicted editing window based on previous publications^13^. Subsequently, sgRNAs are scored based on their position within the transcript, where sgRNAs earlier in the transcript receive a higher score. pmSTOP-inducing gRNAs were designed using the Benchling base editing gRNA design tool (https://benchling.com/pub/liu-base-editor).

### CD3+ T-cell isolation

Peripheral blood mononuclear cells (PBMCs) were isolated from Trima Accel leukoreduction system (LRS) chambers using ammonium chloride-based red blood cell lysis. CD3+ T cells were isolated from the PBMC population by immunomagnetic negative selection using the EasySep Human T-cell Isolation Kit (STEMCELL Technologies, Cambridge, MA). T cells were frozen at 10–20 × 10^6^ cells per 1 mL of Cryostor CS10 (STEMCELL Technologies, Cambridge, MA) and thawed into culture as needed.

### T-cell culture

T cells were cultured in OpTmizer CTS T cell Expansion SFM containing 2.5% CTS Immune Cell SR (ThermoFisher, Waltham, MA), l-Glutamine, Penicillin/Streptomycin, N-Acetyl-l-cysteine (10 mM), IL-2 (300 IU/mL), IL-7 (5 ng/mL), and IL-15 (5 ng/mL) at 37 °C and 5% CO_2_. T cells were activated with Dynabeads Human T-Activator CD3/CD28 (ThermoFisher, Waltham, MA) at a 2:1 bead:cell ratio for 48–72 h prior to electroporation. Following electroporation, T cells were maintained at ~1 × 10^6^/mL in normal tissue culture flasks for experiments optimizing editing efficiency. For large-scale expansion studies and functional assays, edited T cells were expanded in gas-permeable rapid expansion (G-Rex) vessels (Wilson Wolf Manufacturing, New Brighton MN).

### T-cell electroporation

After 48 h, Dynabeads were magnetically removed and cells washed with PBS once prior to resuspension in appropriate electroporation buffer. For singleplex experiments, 3 × 10^5^ T cells were electroporated with 1 µg of chemically modified sgRNA (Synthego, Menlo Park, CA) and 1.5 µg SpCas9, BE3, or BE4 mRNA (TriLink Biotechnologies, San Diego, CA) in a 10 µL tip using the Neon Transfection System (ThermoFisher, Waltham, MA) under the following conditions: 1400 volts, pulse width of 10 ms, three pulses. The 4D-nucleofector (Lonza, Basel, Switzerland) and P3 kit was used for multiplex studies with 1 × 10^6^ T cells per 20 µL cuvette, 1.5–4 µg BE mRNA as indicated, and the Nucleofector program EO-115. RNP were generated by incubation of 10 µg SpCas9 protein (IDT, Coralville, IA), or 12 µg BE4 protein (Aldevron, Fargo, ND) with 3 µg of each chemically modified sgRNA (Synthego, Menlo Park, CA) for 15 min at room temperature, and electroporated using the Nucleofector program EH-115. T cells were allowed to recover in antibiotic-free medium at 37 °C, 5% CO_2_ for 20 min following gene transfer, and were then cultured in complete CTS OpTmizer T cell Expansion SFM as described above.

### Lentiviral transduction

T cells were transduced 24 h after transfection with pRRL-MND-CAR19-RQR8 lentiviral vector (UMN Viral Vector & Cloning Core) at an MOI of 20 by spinfection on Retronectin (Takara)-coated plates.

### Genomic DNA analysis

Genomic DNA was isolated from T cells 5 days post electroporation by spin column-based purification. Base editing efficiency was analyzed on the genomic level by PCR amplification of CRISPR-targeted loci (Supplemental Table 4), Sanger sequencing of the PCR amplicons, and subsequent analysis of the Sanger sequencing traces using the web app EditR (baseeditr.com)^25^. NGS was also performed on the same PCR amplicons.

### RNA isolation, cDNA sequencing, and quantitative RT-PCR

Total RNA was extracted from cells using the PureLink RNA kit (ThermoFisher, Waltham, MA). Five hundred nanograms of purified RNA was reverse transcribed into cDNA using the Transcriptor First Strand Synthesis kit (Roche, Basal, Switzerland). Quantitative RT-PCR was performed in triplicate using SYBR green mix (ThermoFisher, Waltham, MA) on an ABI 7500 machine (Applied Bio Systems, Foster City, CA). All measurements were calculated using the ΔΔCT method and expressed as fold change relative to respective control.

### Next generation sequencing & analysis

Primers with Nextera universal primer adapters (Illumina, San Diego, CA) were designed to amplify a 375–425 bp site surrounding the region of interest using Primer3Plus (Supplemental Table 2). Genomic DNA was PCR-amplified using AccuPrime Taq DNA Polymerase, high fidelity according to the manufacturer’s protocol (Invitrogen, Carlsbad, CA), using the cycle [94 °C—2:00]−30 × [94 °C—0:30, 55 °C—0:30, 68 °C—0:30]-[68 °C—5:00]-[4 °C—hold]. Amplicons were purified from 1% agarose gel using the QIAquick Gel Extraction Kit (Qiagen, Hilden, Germany). Samples were submitted to the University of Minnesota Genomics Center for subsequent amplification with indexing primers and sequencing on a MiSeq 2 × 300 bp run (Illumina, San Diego, CA). A minimum of 1000 aligned read-pairs were generated per OnT site, and 10,000 read-pairs for OT sites. Raw fastq files were analyzed against a reference sequence and sgRNA protospacer sequence using the CRISPR-Cas9 editing analysis pipeline CRISPR-DAV^48^. Output ‘sample_snp.xlsx’ and ‘sample_len.xlsx’ were compiled and analyzed using a custom R markdown script (R v3.4.2**)**. NGS reads are available on the NCBI Sequence Read Archive database with the accession code ‘PRJNA561429’. Scripts for analysis of raw sequencing reads are available on ‘GitHub [https://github.com/MitchellKluesner/CRISPR-DAV_analysis]’.

### GUIDE-seq

GUIDE-seq was performed as described previously for U2OS^31^, with modifications for application to primary T cells. Briefly, CD3+ T cells were stimulated using anti-CD3/CD28 Dynabeads in complete T-cell media for 36–48 h prior to electroporation. In a 100 μL tip, 3 × 10^6^ T cells were electroporated with 15 μg SpCas9 mRNA, 10 μg each sgRNA, and 8–16 pmol of GUIDE-seq dsODN using the Neon electroporation system with pulse conditions 1400 V, 10 ms, 3 pulses. OnT integration of dsDNA oligo was confirmed by PCR and TIDE^49^ analysis prior to downstream processing and analysis. To improve the efficiency of target recovery, shearing by sonication was replaced with an enzymatic shearing strategy using NEB’s Ultra II FS kit (NEB, Ipswich, MA). Data processing and analysis was carried out using the GUIDE-seq analysis pipeline (https://github.com/aryeelab/guideseq).

### RNA OT analysis

Candidate OT editing sites were selected using 293T RNAseq data that was a kind gift from Dr Hui Yang^32^ and the Database of Immune Cell eQTLs (DICE, dice-database.org). Percent of maximal expression for each Gene in CD4^+^–CD8^+^ T-cells was estimated as; Percent Maximal Expression = (Reads^i^_CD4+_ × Reads^i^_CD8+_)/max(Reads_CD4+_ × Reads_CD8+_). The top five genes that were both highly expressed and had previously observed editing in HEK 293T cells were selected for targeted analysis. At each indicated time point, cells were harvested and RNA was extracted using PureLink mRNA Mini Kit according to the manufacturer’s protocol (ThermoFisher, Waltham, MA). RNA was reverse transcribed to cDNA using the Transcriptor First Strand cDNA Synthesis Kit with both random hexamers and oligo dT primers (Roche, Basel, Switzerland). Amplicons specific to regions suspected of editing were amplified using the aforementioned PCR protocol with Accuprime Taq HiFi Polymerase (ThermoFisher, Waltham, MA) (Supplemental Table 5). PCR products were purified using the MinElute 96 UF PCR Purification Kit (Qiagen, Hilden, Germany) and sent for Sanger sequencing (Eurofins, Luxembourg). Percent editing was estimated using the program MultiEditR (https://moriaritylab.shinyapps.io/multieditr/)^34^.

### Flow cytometry

Prior to flow cytometry, singleplex PDCD1 disrupted T cells were re stimulated using CD3/CD28 Dynabeads for 48 h as described above. In multiplex experiments with TRAC knockout, T cells were activated with phorbol 12-myristate 13-acetate (PMA; 100 ng/mL; Sigma-Aldrich, St. Louis, MO) and ionomycin (250 ng/mL; MilliporeSigma, Burlington, MA) for 24 h. T cells treated with PMA/ionomycin were washed with PBS, resuspended in culture medium, and incubated for an additional 24 h prior to flow cytometry. A total of 5 × 10^5^ T cells were stained in a 100 µL volume with 1 µL fluorophore-conjugated anti-human CD3 (BD Biosciences #555335), B2M (BioLegend #316306, #316314), and CD279 (PD-1) (BioLegend #329920) antibodies. Anti-human CD34 monoclonal antibody (QBEnd10) (ThermoFisher #MA1-10205) was used to detect CD19-T2A-RQR8 CAR expression^50^ at 10 µL/5 × 10^5^ cells. Fixable Viability Dye eFluor780 or LIVE/DEAD Fixable Aqua Dead Cell Stain (ThermoFisher #65-0865-14 1:500 dilution, #L34966 1:40 dilution) were used to assess cell viability. T cells were acquired on LSR II or LSRFortessa flow cytometers using FACSDiva software, and data were analyzed using FlowJo v10 software. As stimulation does not uniformly upregulate PD-1 expression in all control T cells, PD-1^+^ cell frequencies were normalized. The ratio (*r*_PD1_) of PD-1^+^ cells to PD-1^-^ subpopulations in control samples was used to calculate the normalized values (*F*′_pos_ and *F*′_neg_) of PD-1^+^ and PD-1^−^ subpopulations from the nonnormalized values (*F*°_pos_ and *F*°_pos_) for all samples as follows:

$$F^\prime _{{\mathrm{pos}}} = F^\circ _{{\mathrm{pos}}} + F^\circ _{{\mathrm{pos}}}\left( {1 - r_{{\mathrm{PD1}}}} \right)$$

$$F^\prime _{{\mathrm{neg}}} = F^\circ _{{\mathrm{neg}}} - F^\circ_{{\mathrm{pos}}}\left( {1 - r_{{\mathrm{PD1}}}} \right)$$

### Cytokine profiling

A total of 2 × 10^5^ T cells were incubated for 12 h in 200 µL of OpTmizer CTS T cell Expansion SFM containing 2.5% CTS Immune Cell SR, l-glutamine, penicillin/streptomycin, N-acetyl-l-cysteine (10 mM) that contained monensin (0.7 μg/mL; BD Biosciences) and brefeldin A (10 μg/mL; Sigma-Aldrich) in the absence or presence of K562 cells or Raji cells, or Raji cells engineered to over-express PDL1. After washing, cells were surface stained in a 100 µL volume for CD4 (BD Horizon #564651, 5 µL), CD8 (BD Horizon #564912, 0.5 µL), CD27 (BioLegend #302830, 2 µL), and CD45ro (BioLegend #304236, 2 µL); eFluor780 amine reactive dye was used to exclude dead cells from the analysis. Following permeabilization (Cytofix/Cytoperm kit; BD Biosciences), cells were stained for CD3 (BD Horizon #564001, 2.5 µL), gamma interferon (IFN-γ) (eBioscience #48-7319-42, 3 µL), interleukin 2 (IL-2) (Invitrogen #25-7029-42, 2 µL), and tumor necrosis factor (TNF) (eBioscience #17-7349-82, 0.4 µL). Between 5 × 10^4^ and 1 × 10^5^ events were collected in each case. Electronic compensation was conducted with mAb capture beads (BD Biosciences) stained separately with the individual mAbs used in the test samples. Cells were analyzed using a modified Fortessa flow cytometer (BD Immunocytometry Systems). Data were analyzed using FlowJo version 9.9.3. Forward scatter area vs. forward scatter height was used to gate out cell aggregates and dead cells were removed from the analysis to reduce background staining. Background levels of staining and cytokine production were determined using unstimulated T cells.

### Translocation quantification by ddPCR

Translocation PCR assays were designed using PrimerQuest software (Integrated DNA Technologies, Coralville IA) using settings for 2 primers + probe qPCR (Supplemental Table 3). Each sample was run as a duplexed assay consisting of an internal reference primer + probe set (HEX) and an experimental primer + probe set (FAM). Primers and probes were ordered from IDT. Reactions were set up using the ddPCR Supermix for Probes (no dUTP) (Bio-Rad, Hercules, CA) with 200 ng of genomic DNA per assay according to manufacturer’s instructions. Droplets were generated and analyzed using QX200 Droplet-digital PCR system (Bio-Rad, Hercules, CA). Frequency was calculated as fractional abundance adjusted for two-copies of reference sequence per genome using the QuantaSoft ver 14.0 software (Bio-Rad, Hercules, CA).

### Cytogenetic analysis by FISH

*TRAC, B2M*, and *PDCD1* DNA probes were labeled by nick translation reaction (Nick Translation Kit—Abbott Molecular) using Green—500 dUTP (Enzo Life Science). Sizes of the nick translated fragments were checked by electrophoresis on a 1% TBE gel. The labeled DNA was precipitated in COT-1 DNA, salmon sperm DNA, sodium acetate and 95% ethanol, then dried and resuspended in 50% formamide hybridization buffer. The probe/hybridization buffer mix and slides were denatured, probe was applied to the slides, and slides were hybridized for 48 h at 37° in a humidified chamber. After hybridization, the FISH slides were washed in a 2× SSC solution at 72° for 15 s, and counterstained with DAPI. Fluorescent signals were visualized on an Olympus BX61 microscope workstation (Applied Spectral Imaging, Vista, CA) with DAPI and FITC filter sets. FISH images were captured using an interferometer-based CCD cooled camera (ASI) and FISHView ASI software.

### Cytotoxicity assay

Luciferase-expressing K562 (ATCC), Raji (ATCC), or Raji-PDL1 (generated from the Raji line) cells were seeded into a 96-well round-bottom plate (3 × 10^4^ cells/well). T cells were counted and added to the wells in triplicate at the indicated E:T ratios. Target cells without effectors served as a negative control (spontaneous cell death) and target cells incubated with 1% NP-40 served as positive control (maximum killing). Co-cultures were incubated at 37 °C for 48 h. After incubation, d-luciferin (potassium salt; Gold Biotechnology) was added to each well at a final concentration of 25 µg/mL and incubated 10 min before imaging. Luminescence was read in endpoint mode using BioTek Synergy microplate reader. Target cells with no effectors were set as 100% survival and killing in experimental samples was measured against this baseline.

### Immunoblotting assay

Proteins were isolated from 1 × 10^6^ cells in complete RIPA buffer with protease and phosphatase inhibitors (Sigma-Aldrich, COEDTAF-RO, P5726, and P0044). Total protein was quantified using the Pierce BCA Protein Assay Kit (ThermoFisher, Waltham, MA) according to the manufacturer’s protocol. 3 µg/µL of cell lysate was run and analyzed on the Wes platform after being denatured at 95 °C for 5 min according to the manufacturer’s protocol (ProteinSimple, San Jose, CA). Primary antibodies against SpCas9 (Cell Signaling #14697) and actin (Cell Signaling #8457) were used at 1:100 and 1:50 dilutions, respectively, in kit-supplied buffer and platform-optimized secondary antibodies were purchased from ProteinSimple.

### Data analysis and visualization

All statistical analyses were performed in R studio. The level of significance was set at *α* = 0.05. Data were subjected to analyses for the assumptions of normality and homeodascity prior to statistical testing. Student’s pairwise one-tailed or two-tailed t-tests were used as indicated in the text. Data were visualized using either Prism 8 (Graphpad), or R studio employing various tidyverse (https://www.tidyverse.org/) and Bioconductor (https://www.bioconductor.org/) packages.

### Reporting summary

Further information on research design is available in the Nature Research Reporting Summary linked to this article.

## Supplementary information

Supplementary Information

Dataset 1

Dataset 3

Dataset 2

Description of Additional Supplementary Files

Source Data

Reporting Summary

## Data Availability

Next-generation sequencing reads are available on the NCBI Sequence Read Archive database with the accession code PRJNA561429. Scripts for analysis of raw sequencing reads are available on GitHub. Source data are available from corresponding author upon request.
