# Author Correction: Highly efficient multiplex human T cell engineering without double-strand breaks using Cas9 base editors

**DOI:** 10.1038/s41467-019-13778-y

**Published:** 2019-12-06

**Authors:** Beau R. Webber, Cara-lin Lonetree, Mitchell G. Kluesner, Matthew J. Johnson, Emily J. Pomeroy, Miechaleen D. Diers, Walker S. Lahr, Garrett M. Draper, Nicholas J. Slipek, Branden A. Smeester, Klaus N. Lovendahl, Amber N. McElroy, Wendy R. Gordon, Mark J. Osborn, Branden S. Moriarity

**Affiliations:** 1 Department of Pediatrics, University of Minnesota, Minneapolis, MN USA; 2 Masonic Cancer Center, University of Minnesota, Minneapolis, MN USA; 3 Center for Genome Engineering, University of Minnesota, Minneapolis, MN USA; 4 Stem Cell Institute, University of Minnesota, Minneapolis, MN USA; 5 Department of Biochemistry, Molecular Biology, and Biophysics, University of Minnesota, Minneapolis, MN USA

**Keywords:** Genetic engineering, Immunotherapy, CRISPR-Cas9 genome editing

Correction to: *Nature Communications* 10.1038/s41467-019-13007-6, published online 19 November 2019

The original version of this Article contained an error in the spelling of the author Branden A. Smeester, which was incorrectly given as Branden S. Smeester. This has now been corrected in both the PDF and HTML versions of the Article.

