# Supplementary material for: Highly efficient multiplex human T cell engineering without double-strand breaks using Cas9 base editors
Source: Nat Commun. 2019 Nov 19;10:5222. doi: 10.1038/s41467-019-13007-6 (PMC6864045; doi:10.1038/s41467-019-13007-6)
Supplement: Supplementary file 1 — Supplementary Information [file 41467_2019_13007_MOESM1_ESM.pdf]

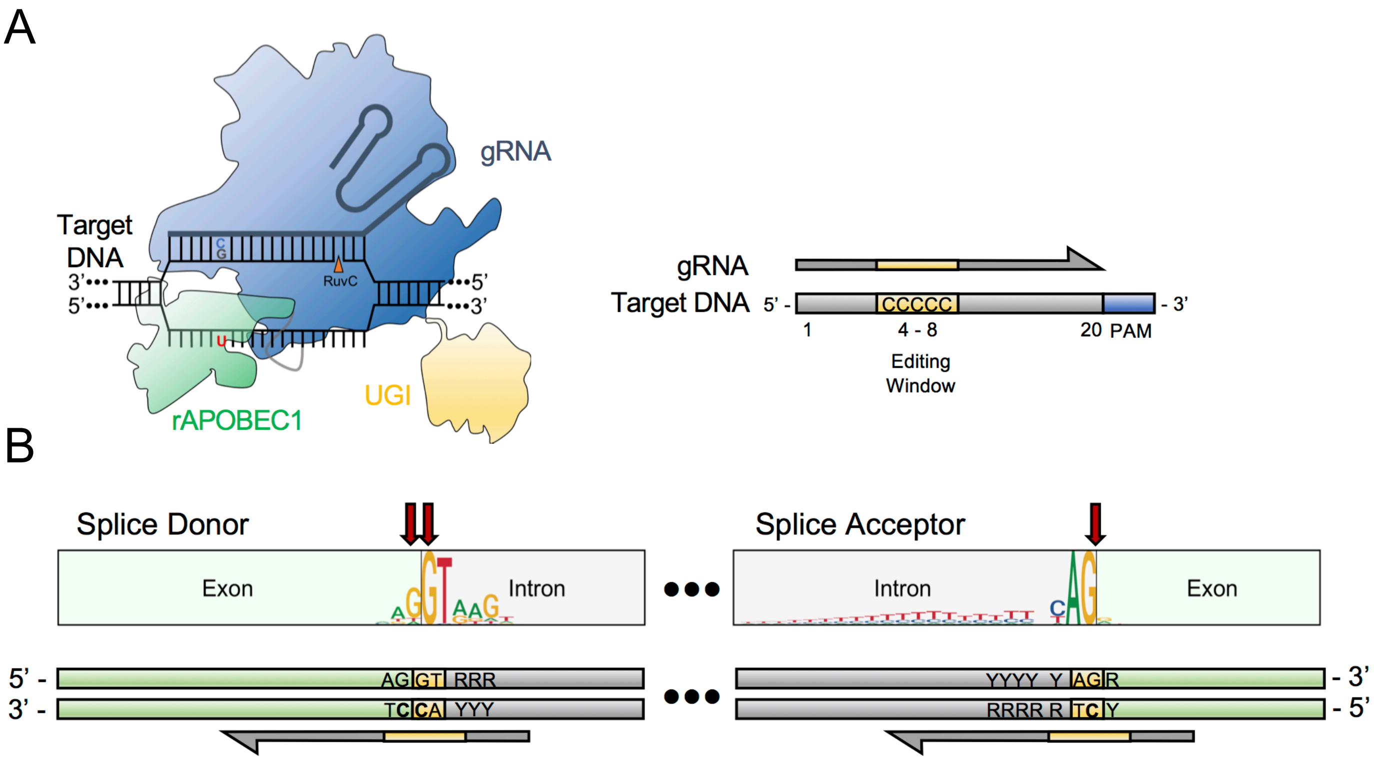


**Supplementary Figure 1**: **Targeting splice sites using base editor. A.** Diagram depicting Cas9 base editor (BE) bound to target DNA (left) and protospacer depicting the purported base editing window achieved with BE3 and BE4 (right). **B.** Logo diagrams depicting the consensus sequence of mammalian splice donor (SD) and splice acceptor (SA) elements and the related orientation of protospacers utilized for BE knockout via splice site disruption. Red arrows indicate target Cs (G antisense) predicted to disrupt splicing following conversion.

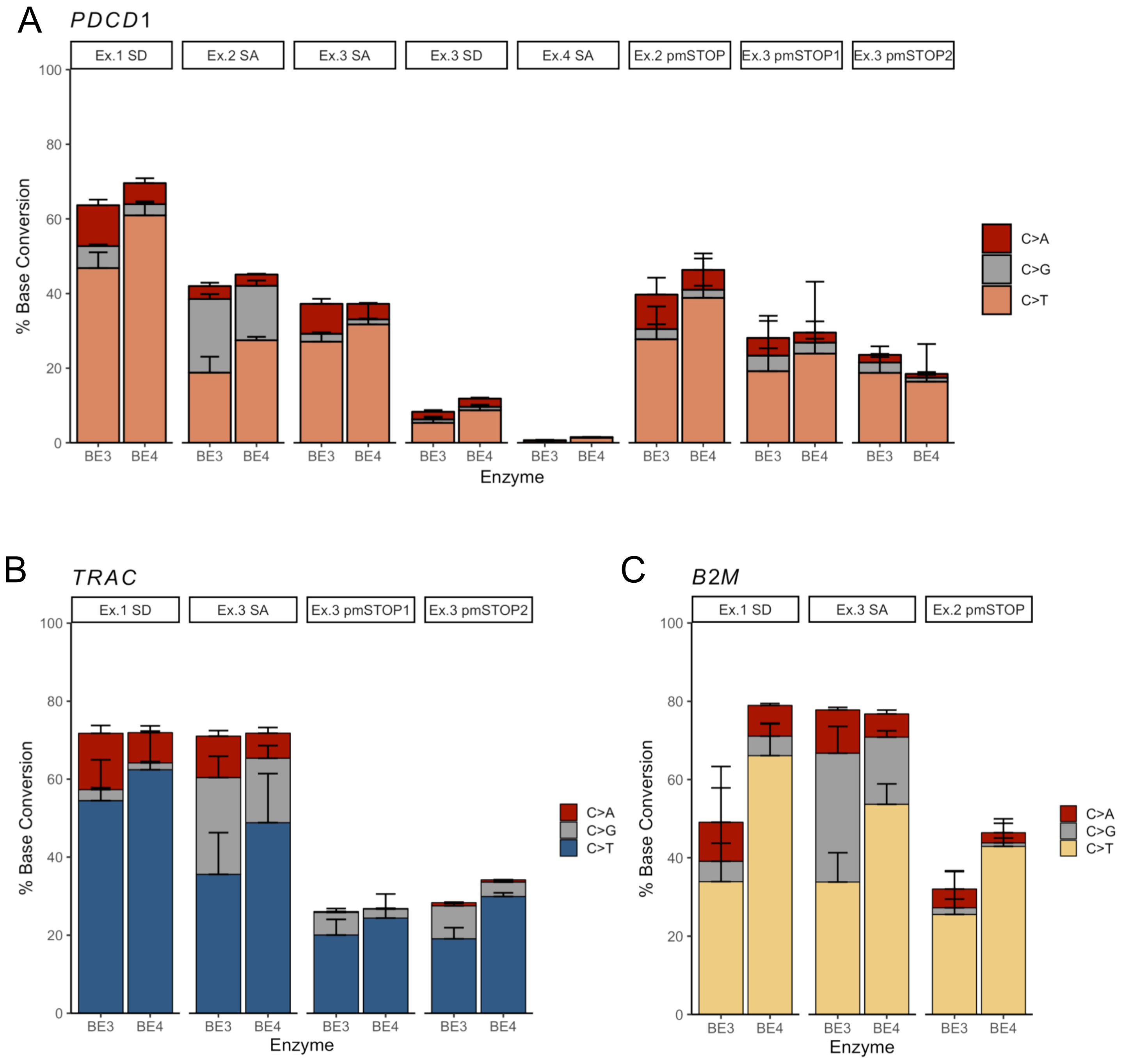


**Supplementary Figure 2. Non-target editing for each sgRNA in figure 1.** Data is analyzed from NGS. Height of stacked bars represents mean, with error bars ± 1 standard deviation. *n=3 independent donors.* Source data are provided as a Source Data file.

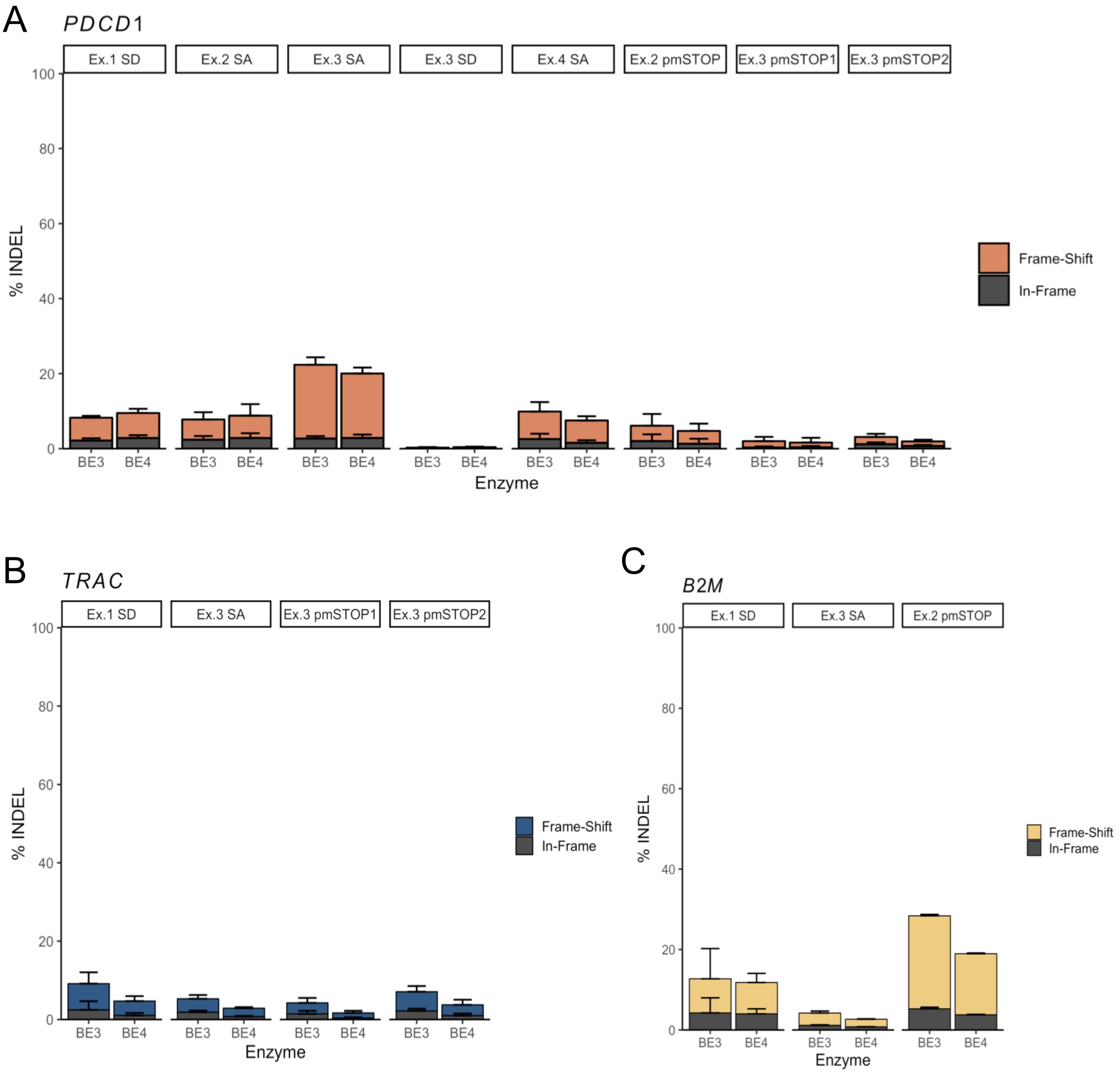


**Supplementary Figure 3. Indels for all samples in Figure 1.** Data is analyzed from NGS. Height of stacked bars represents mean, with error bars ± 1 standard deviation. *n=3 independent donors.* Source data are provided as a Source Data file.

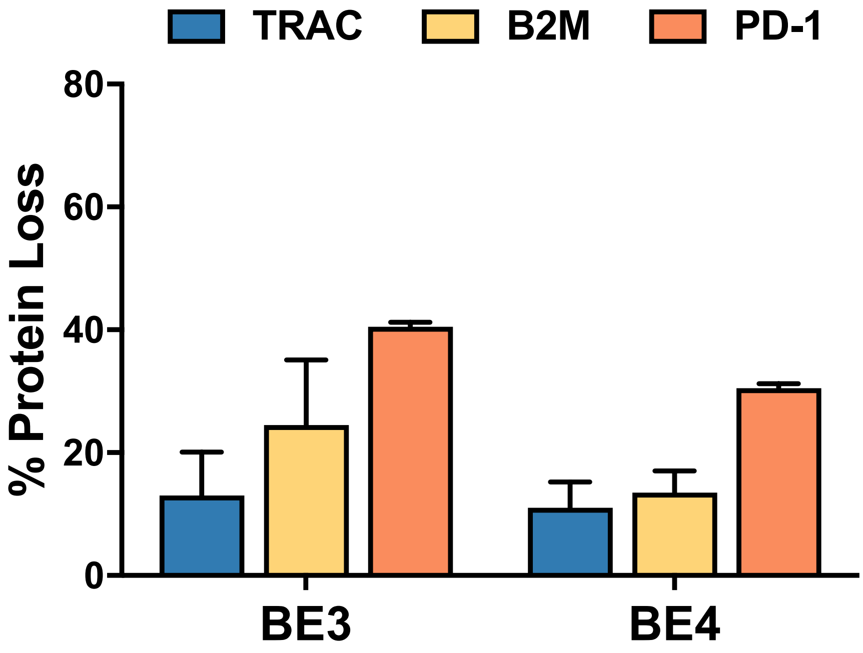


**Supplementary Figure 4. Multiplex base editing of T cells using first-generation, low-dose (1.5 µg) BE3 or BE4 mRNA.** Bar graph depicting base editor mediated knockout of TRAC, B2M and PD-1 at the protein level. Protein expression was assessed via flow cytometry as described in the methods section. *n = 2 independent donors.* Source data are provided as a Source Data file.

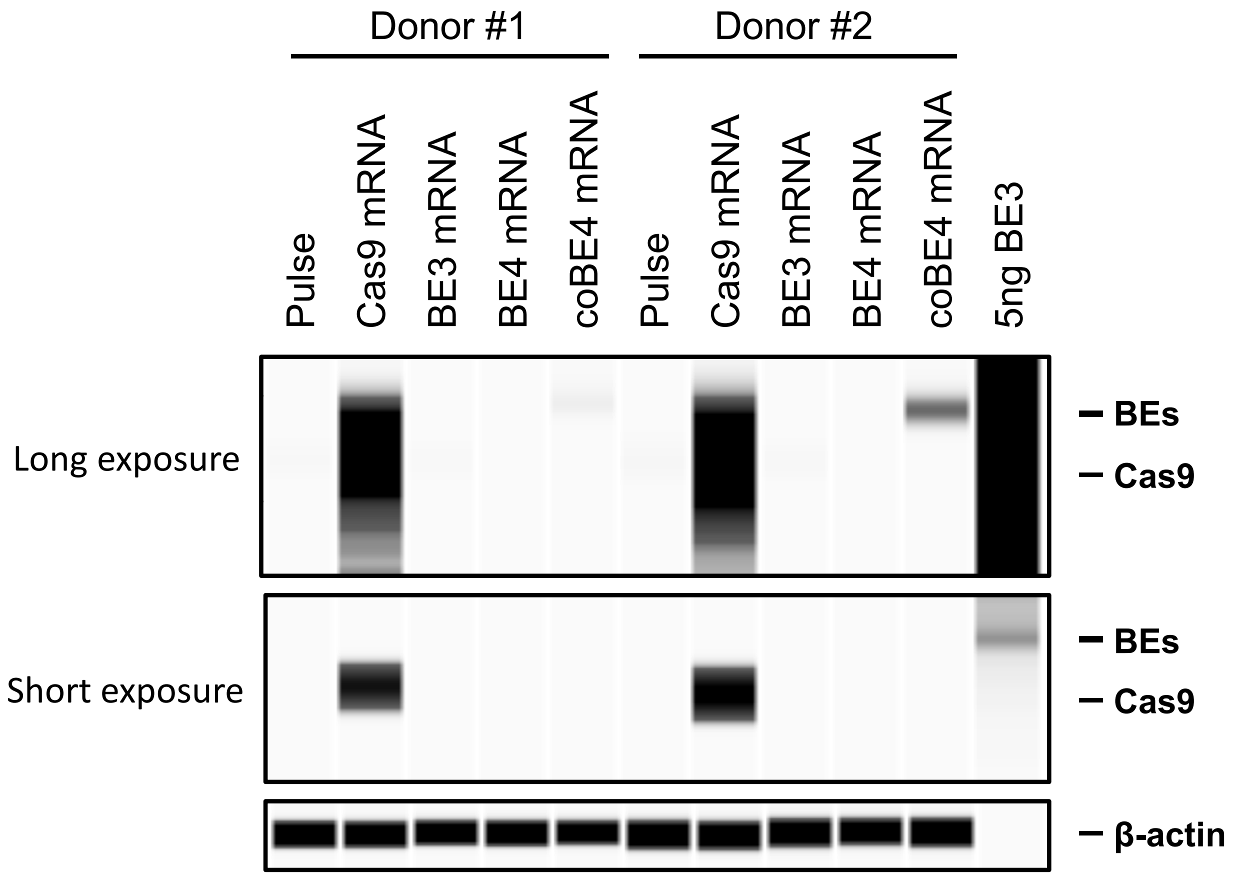


**Supplementary Figure 5. Base editor protein levels following electroporation of T cells.** Digital western blot results assessing the protein level achieved using mRNA encoding Cas9, BE3, BE4, and codon-optimized BE4 at 24 post electroporation of stimulated T cells in two independent donors. Purified BE3 protein was also used as a positive control for antibody detection of BE protein. Source data are provided as a Source Data file.

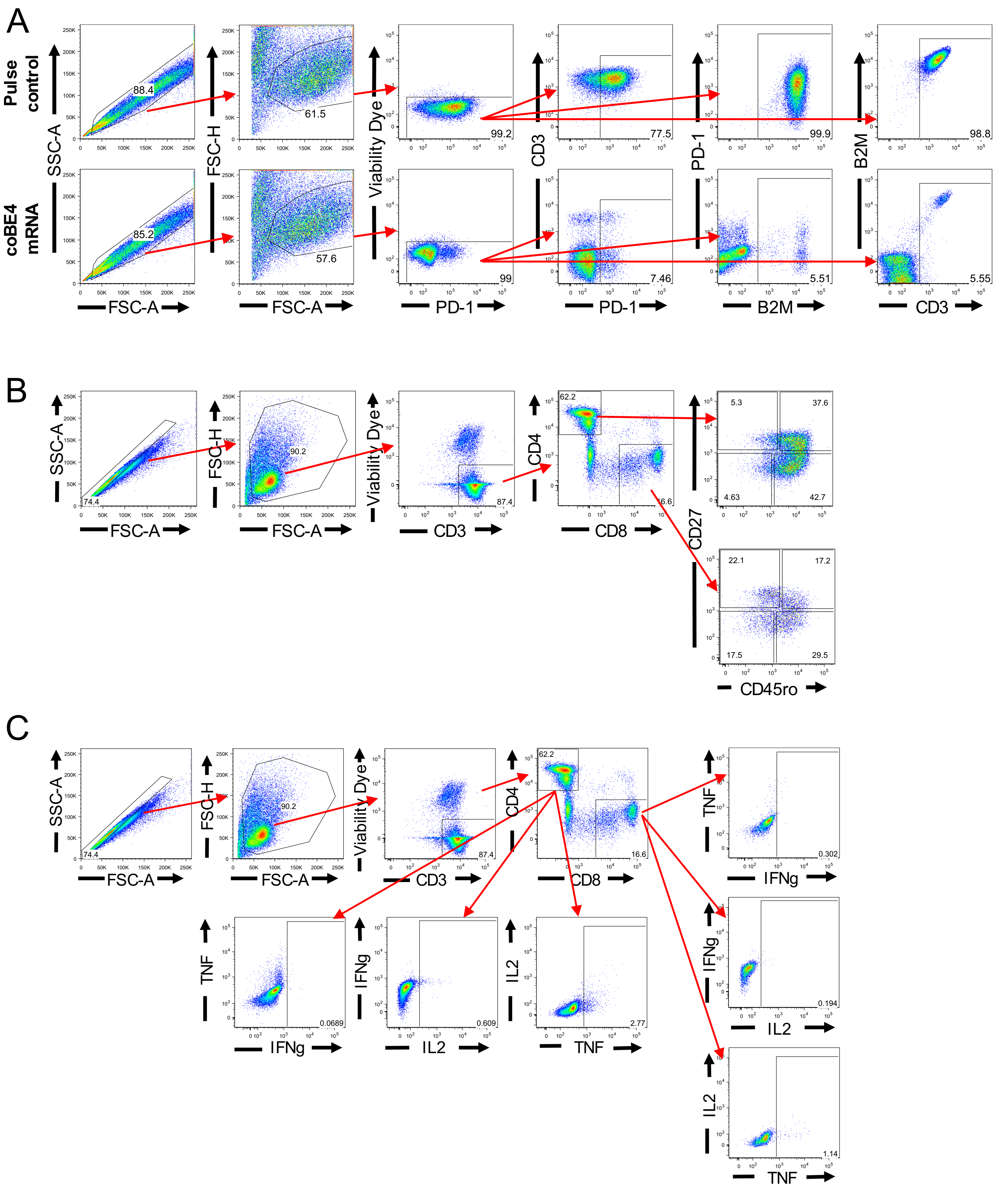


**Supplementary Figure 6. Gating tree and representative flow plots for Figures 1, 2, 4a, 4b. (A)** Gating tree for Figures 1 and 2. **(B)** Gating tree for Figure 4a. **(C)** Gating tree for Figure 4b.

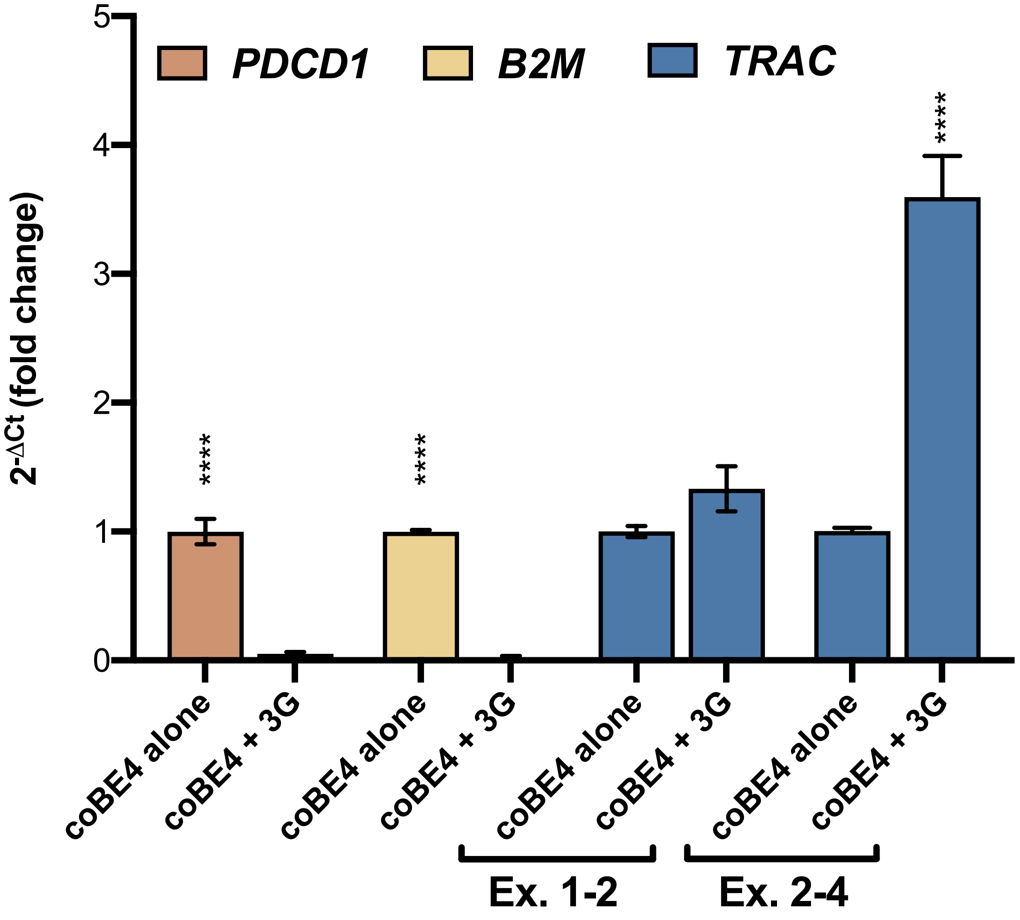


**Supplementary Figure 7. mRNA expression level of target genes in base edited T cells.** T cells were electroporated with coBE4 mRNA alone or with optimal sgRNAs targeting *PDCD1, B2M, and TRAC.* Five days post electroporation mRNA was collected and transcript levels were determined using RT-qPCR. Specific exon junction assayed is indicated for *TRAC.* Source data are provided as a Source Data file.

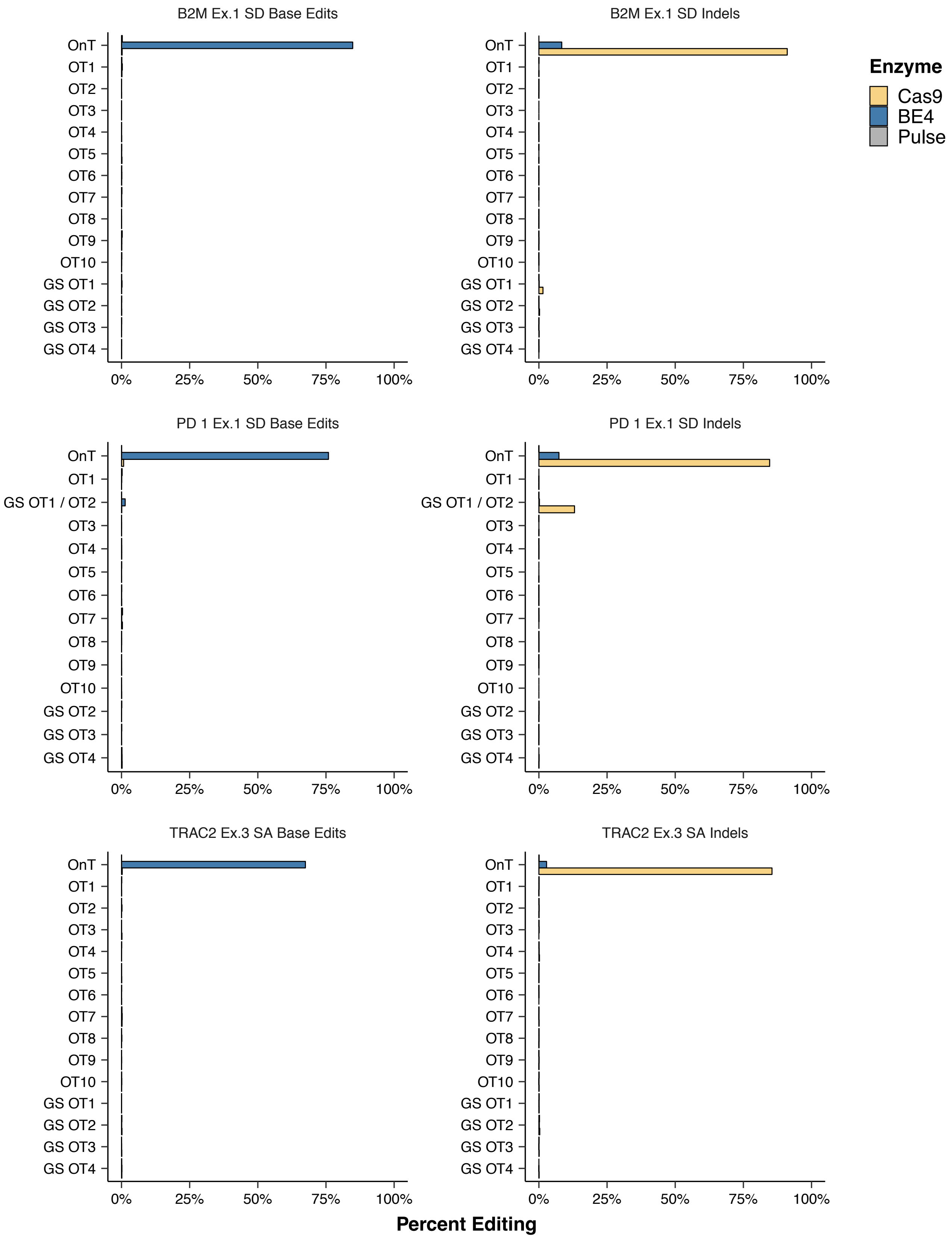


**Supplementary Figure 8: Assessment of computationally predicted off-target base editing and indel formation.** Base editing **(A)** and indel **(B)** frequency at on-target and top 10 computationally predicted off-target sgRNA binding sites, assessed using next generation sequencing, using optimal sgRNAs targeting *TRAC*, *B2M* or *PDCD1* combined with Cas9 or BE4 mRNA in T cells. Source data are provided as a Source Data file.

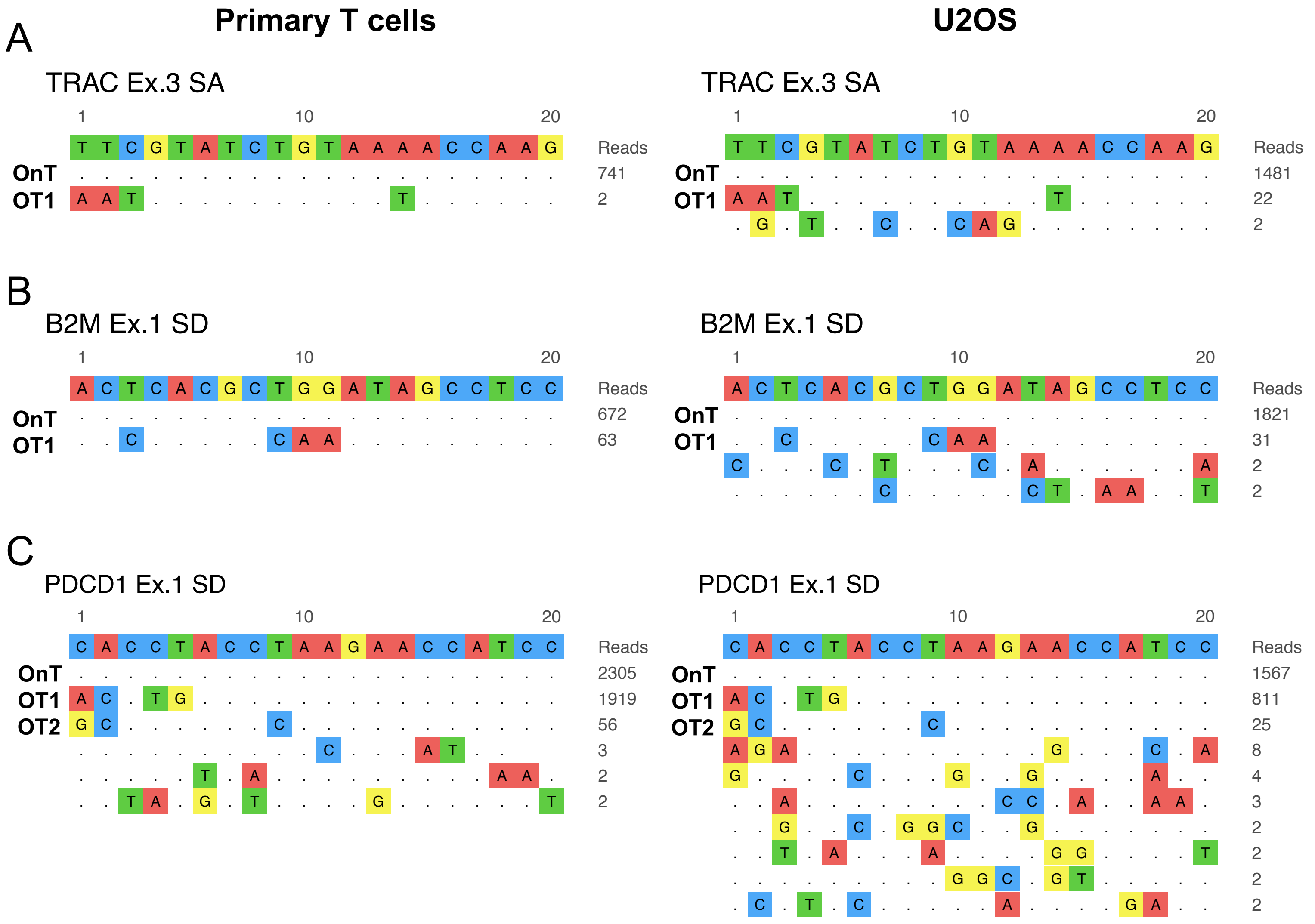


D

| OT | Cas9 Indels | BE4 Indels | BE4 %C to T |
|---|---|---|---|
| TRAC Ex.3 SA GS_OT1 | <0.1% | <0.1% | <0.1% |
| B2M Ex.1 SD GS_OT1 | 0.2% | <0.1% | <0.1% |
| PDCD1 Ex.1 SD GS_OT1* | 13.1% | <0.1% | 0.9% |
| PDCD1 Ex.1 SD GS_OT2 | <0.1% | <0.1% | <0.1% |

**Supplementary Figure 9: GUIDE-seq assessment of off-target editing.** GUIDE-seq (GS) analysis in primary human T cells and U2OS for **(A)** *TRAC* Ex.3 SA**, (B)** *B2M* Ex.1 SD, and **(C)** *PDCD1* Ex.1 SD. **(D)** Editing levels determined by NGS at OT sites identified in both primary T cells and U2OS**.** **PDCD1* GS-OT1 was also computationally identified as *PDCD1* OT2 in **Supplemental Data 8**.

A

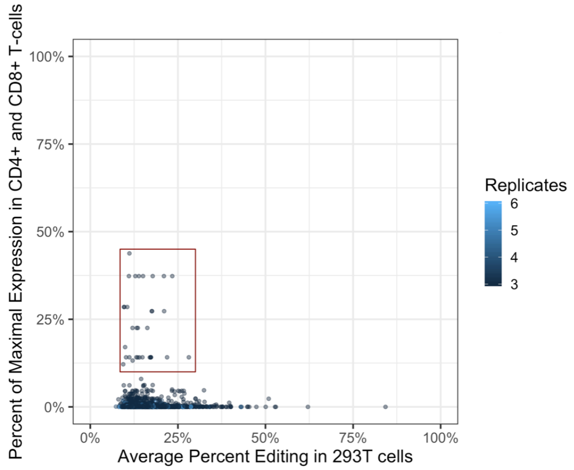


B

| Gene | Edits | Average % Edit | SD % Edit | Transcript Length (bp) | CD4+ Reads | CD8+ Reads |
|---|---|---|---|---|---|---|
| CDV3 | 9 | 16.8 | 5.56 | 1161 | 1981.57 | 1876.55 |
| RPS12 | 7 | 16.43 | 4.5 | 2749 | 2776.58 | 3531.95 |
| DDX21 | 4 | 13.79 | 1.78 | 876 | 2780.54 | 2128.8 |
| CYCS | 3 | 18.74 | 2.02 | 236 | 3221.52 | 2228.1 |
| RPL37 | 3 | 14.83 | 1.87 | 1043 | 1740.31 | 2131.52 |

C

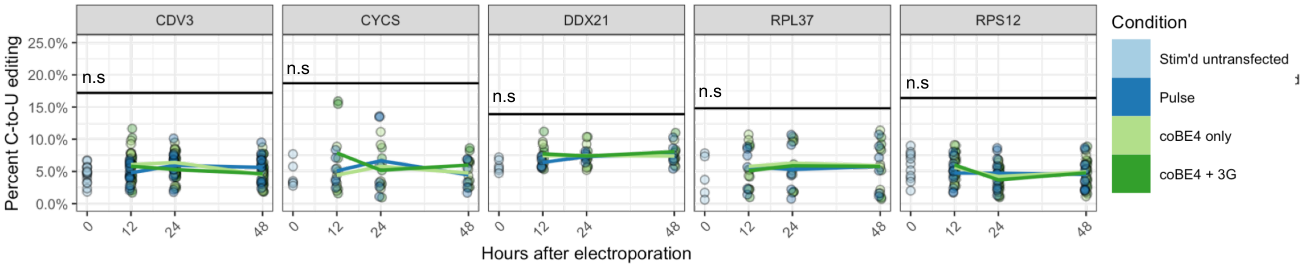


**Supplementary Figure 10: Off-target RNA editing analysis. (A)** Percent maximal gene expression in CD4⁺-CD8⁺ T cells plotted against multiplicate RNA editing observed in previous reports in HEK 293T cells. Red box indicates transcripts selected based on observed editing in HEK 293T and high expression in T cells. **(B)** Summary of gene transcripts analyzed for off-target RNA editing. **(C)** Line plots of the measured C-to-U editing across each treatment condition over indicated time points. Black horizontal line represents % editing previously observed in HEK 293T cells. Differences in measured % T fluorescence are not significant with respect to condition, time points, and donor across all genes tested (One way repeated measures ANOVA, *P* > 0.10, N = 26). Source data are provided as a Source Data file.

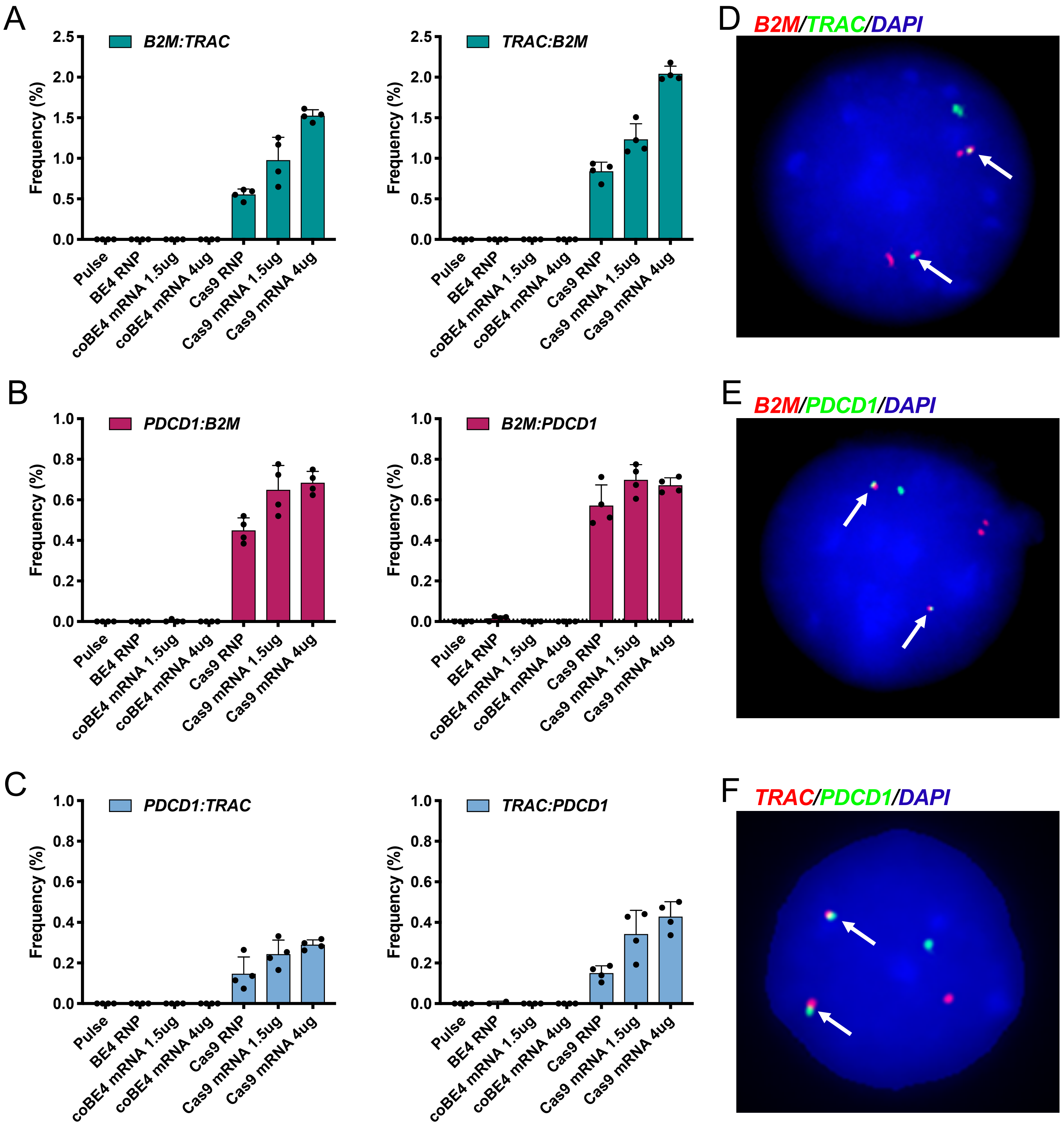


**Supplementary Figure 11: Translocation frequency between *TRAC, B2M, and PDCD1* target loci.** Droplet digital PCR (ddPCR) quantification of translocation frequency between *TRAC and B2M* **(A)**, *B2M and PDCD1* **(B)**, and *PDCD1 and TRAC* **(C)** following delivery of *TRAC, B2M,* and *PDCD1* sgRNA plus spCas9 protein, spCas9 mRNA, BE4 protein, or coBE4 mRNA at indicated doses. **(D, E, F)** Fluorescence *in situ* hybridization (FISH) verification of translocation event in multiplex Cas9 nuclease engineered T cells. *n=2 independent T cell donors assayed in duplicate.* Source data are provided as a Source Data file.

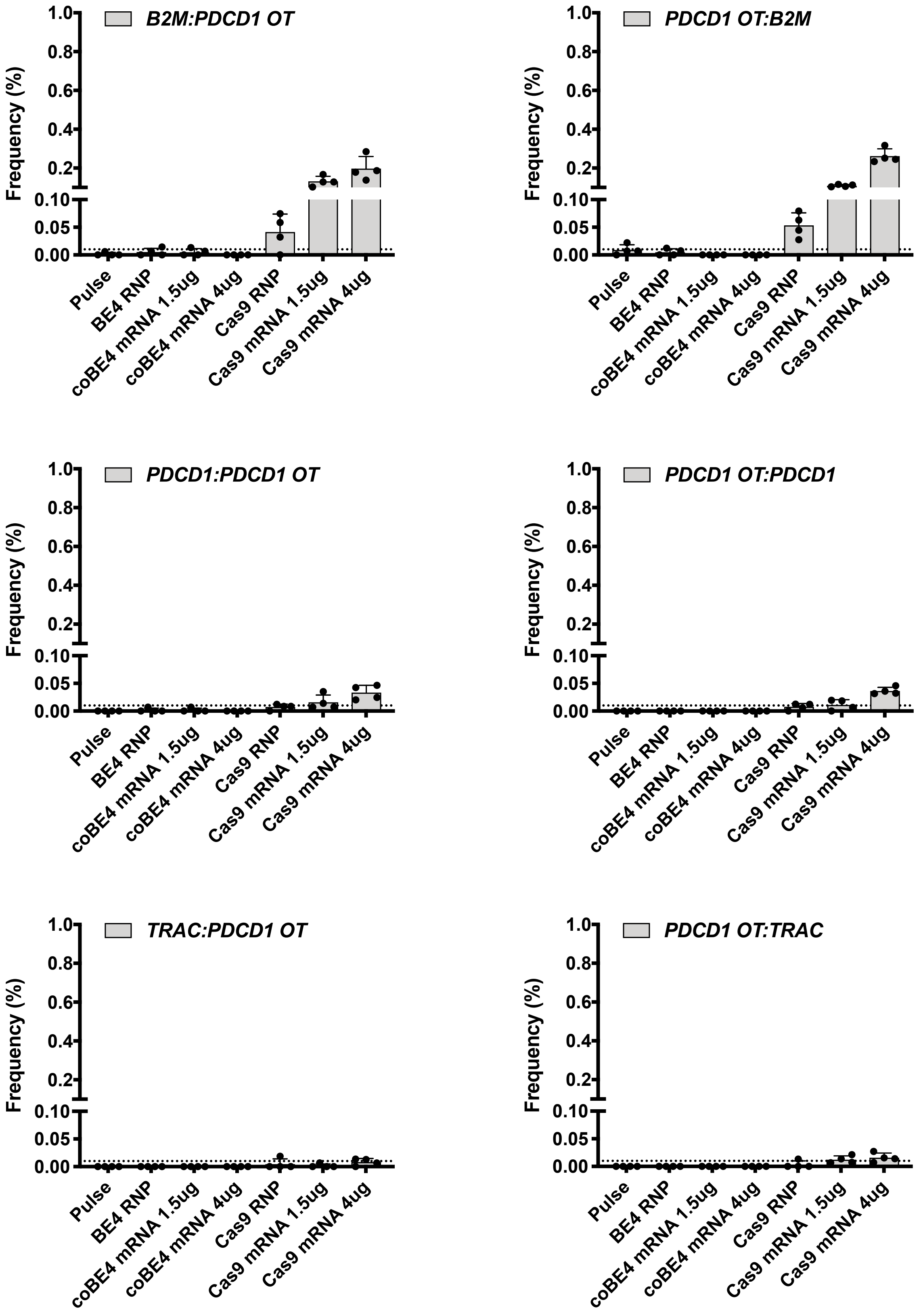


**Supplementary Figure 12: Translocation frequency between target loci and PDCD1 off-target site.** Droplet digital PCR quantification of translocation frequency between *PDCD1* OT site and *TRAC, B2M,* and *PDCD1* after delivery of three sgRNA and spCas9 protein, spCas9 mRNA, BE4 protein, or coBE4 mRNA. Dashed line indicates 0.01% limit of detection (LOD). *n=2 independent T cell donors assayed in duplicate.* Source data are provided as a Source Data file.

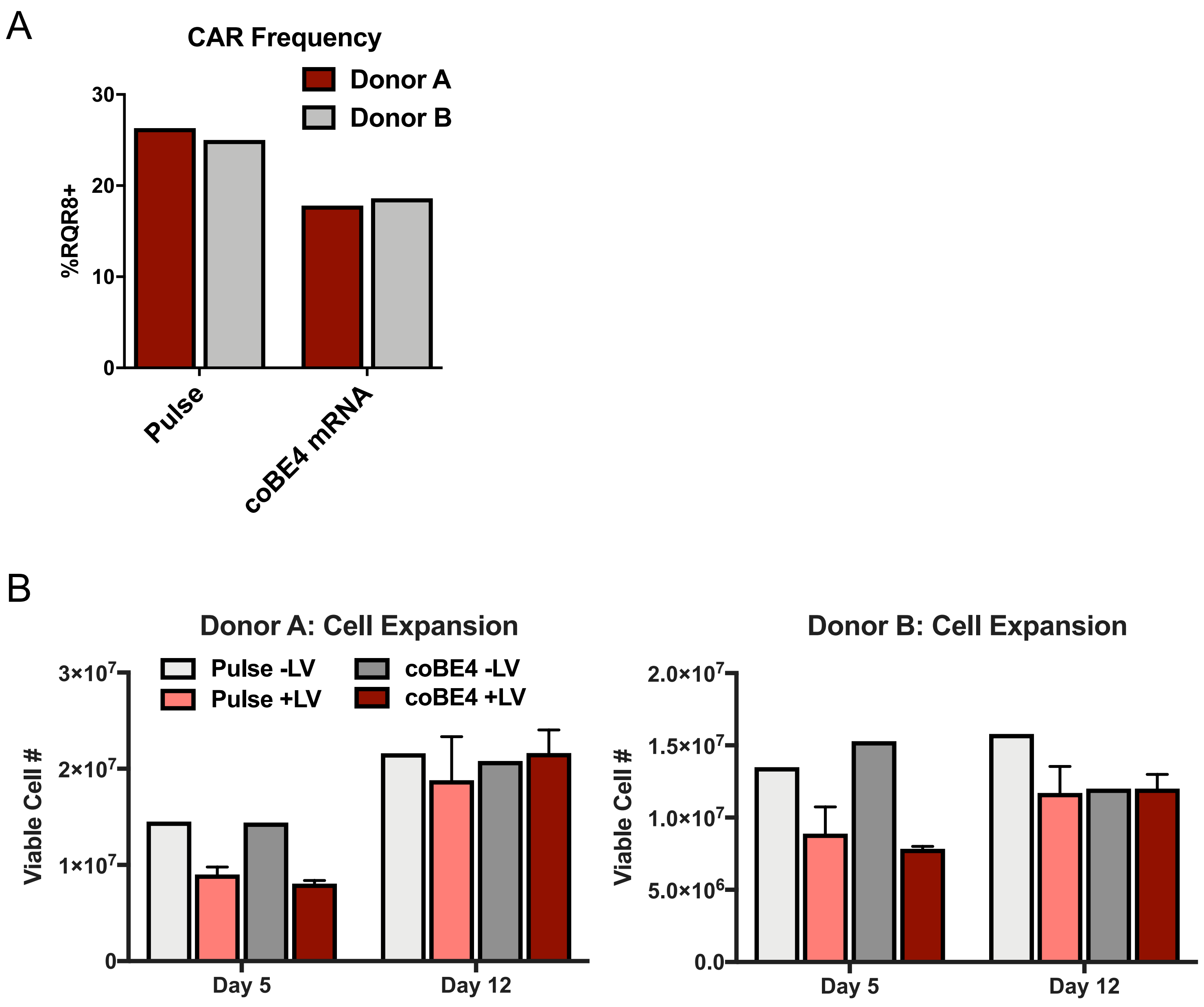


**Supplementary Figure 13: CAR transduction and T cell expansion efficiency. A.** Bar graph depicting the frequency of transduced T cells using the MND-CD19 CAR-RQR8 lentiviral vectors, MOI of 20, via staining for RQR8 in two independent donors. RQR8 is a hybrid molecule containing domains for staining with CD34 and CD20 specific antibody and serves as a surrogate for determining CAR positive T cell frequency. **B.** Bar graphs depicting the number of viable cells at day 5 and 12 post electroporation and transduction. *n=2 independent donors.* Source data are provided as a Source Data file.

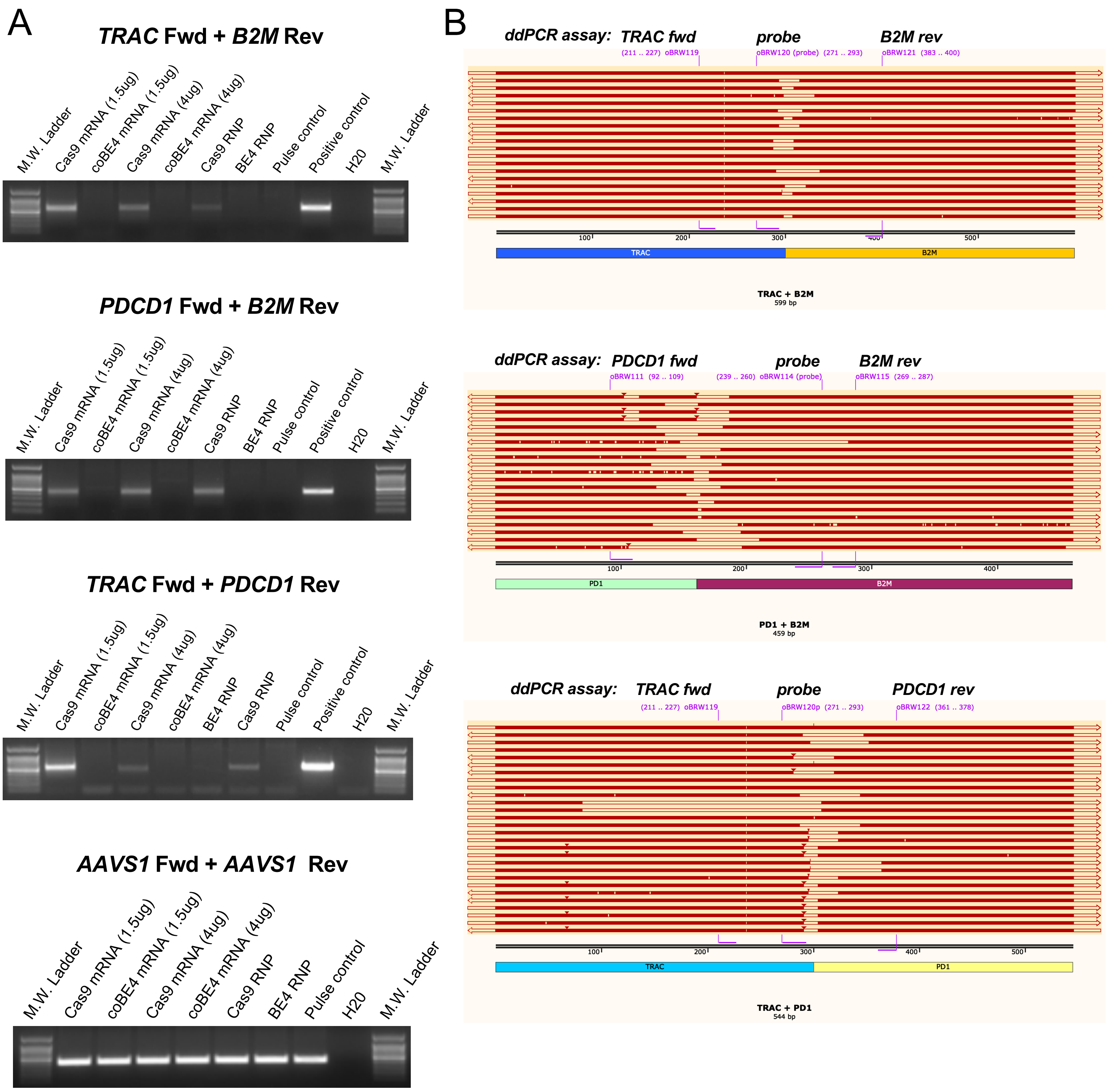


**Supplementary Figure 14: Sequencing of sub-cloned PCR products spanning translocation junctions. A.** Results of translocation PCR performed between the noted target genes using Cas9 or BE mRNA or protein, as noted. An AAVS1 control PCR was also performed to confirm gDNA quality and functionality for PCR. **B.** PCR products from (A) were TA cloned into TOPO plasmids and subsequently analyzed via Sanger sequencing. Resultant chromatograms were then aligned to a hypothetical 'perfect' junction sequence between the noted target gene gRNA cut sites and aligned. Also depicted are ddPCR probes used to generate the data presented in main Figure 3. Source data are provided as a Source Data file.

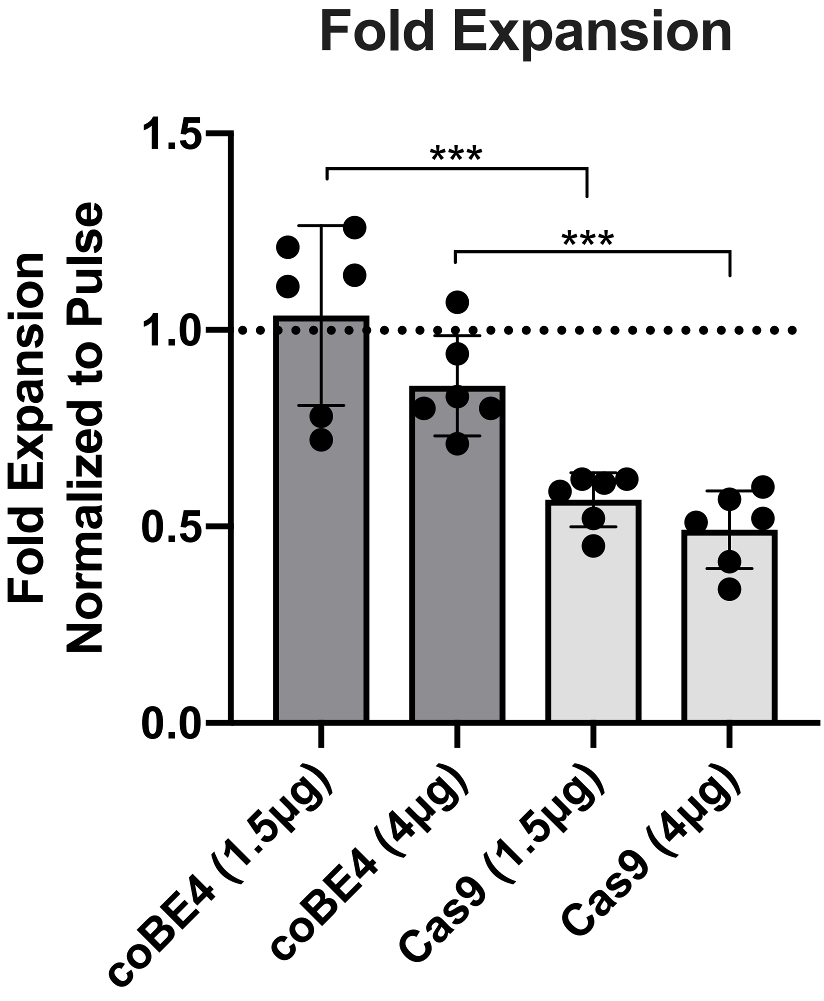


**Supplementary Figure 15: Expansion of multiplex edited T cells.** T cells were electroporated with nothing (pulse) or the indicated dose of coBE4 or spCas9 nuclease mRNA plus *PDCD1, B2M, and TRAC* sgRNAs. Electroporated T cells were immediately transferred to one well of a G-Rex 24-well culture vessel and allowed to expand for 12 days, at which point viable cells were quantified. Fold expansion was determined for each condition and normalized to the pulse control. *n=3 independent T cell donors assayed in duplicate.* Source data are provided as a Source Data file.

**Supplemental Table 1. Guide RNA sequences and associated information.**

| Gene | gRNA Name | 5' - gRNA Sequence - 3' | Orientation | Target base(s) | Predicted Outcome |
|---|---|---|---|---|---|
| *PDCD1* | Ex. 1 SD | CACCTACCTAAGAACCATCC | Antisense | C7 | Splice donor disruption: GT → AT |
| *PDCD1* | Ex. 2 SA | GGAGTCTGAGAGATGGAGAG | Antisense | C6 | Splice acceptor disruption: AG → AA |
| *PDCD1* | Ex. 3 SA | TTCTCTCTGGAAGGGCACAA | Antisense | C7 | Splice acceptor disruption: AG → AA |
| *PDCD1* | Ex. 3 SD | GACGTTACCTCGTGCGGCCC | Antisense | C8 | Splice donor disruption: GT → AT |
| *PDCD1* | Ex. 4 SA | CCTGCAGAGAAACACACTTG | Antisense | C2 | Splice acceptor disruption: AG → AA |
| *PDCD1* | Ex. 2 pmSTOP | GGGGTTCCAGGGCCTGTCTG | Antisense | C7, C8 | pmSTOP induction: TGG (Trp) → TAG, TGA, TAA |
| *PDCD1* | Ex. 3 pmSTOP_1 | CAGTTCCAAACCCTGGTGGT | Sense | C7 | pmSTOP induction: CAA (Gln) → TAA |
| *PDCD1* | Ex. 3 pmSTOP_2 | GGACCCAGACTAGCAGCACC | Antisense | C5, C6 | pmSTOP induction: TGG (Trp) → TAG, TGA, TAA |
| *TRAC* | Ex. 1 SD | CTTACCTGGGCTGGGGAAGA | Antisense | C5 | Splice donor disruption: GT → AT |
| *TRAC* | Ex. 3 SA | TTCGTATCTGTAAAACCAAG | Antisense | C8 | Splice acceptor disruption: AG → AA |
| *TRAC* | Ex. 3 pmSTOP_1 | TTTCAAAACCTGTCAGTGAT | Sense | C4 | pmSTOP induction: CAA (Gln) → TAA |
| *TRAC* | Ex. 3 pmSTOP_2 | TTCAAAACCTGTCAGTGATT | Sense | C3 | pmSTOP induction: CAA (Gln) → TAA |
| *B2M* | Ex. 1 SD | ACTCACGCTGGATAGCCTCC | Antisense | C6 | Splice donor disruption: GT → AT |
| *B2M* | Ex. 3 SA | TCGATCTATGAAAAAGACAG | Antisense | C6 | Splice acceptor disruption: AG → AA |
| *B2M* | Ex. 2 pmSTOP | CTTACCCCACTTAACTATCT | Antisense | C7, C8 | pmSTOP induction: TGG (Trp) → TAG, TGA, TAA |

**Supplemental Table 2. OT NGS primer sequences.**

| Site Name | Off-Target Sequence | Primer Name | Primer Sequence |
|---|---|---|---|
| B2M_Ex1_SD_OnT.1 | ACTCACGCTGGATAGCCTCC | B2M_Ex1_SD_OnT Fwd 1 | TCGTCGGCAGCGTCAGATGTGTATAAGAGACAGATCCAGCCCTGGACTAGC |
| B2M_Ex1_SD_OnT.1 | ACTCACGCTGGATAGCCTCC | B2M_Ex1_SD_OnT Rev 1 | GTCTCGTGGGCTCGGAGATGTGTATAAGAGACAGCTCTCTCTAACCTGGCACTG |
| B2M_Ex1_SD_OnT.1 | ACTCACGCTGGATAGCCTCC | B2M_Ex1_SD_OnT Fwd 2 | TCGTCGGCAGCGTCAGATGTGTATAAGAGACAGATCCAGCCCTGGACTAGC |
| B2M_Ex1_SD_OnT.1 | ACTCACGCTGGATAGCCTCC | B2M_Ex1_SD_OnT Rev 2 | GTCTCGTGGGCTCGGAGATGTGTATAAGAGACAGCCTCTCTCTAACCTGGCACT |
| B2M_Ex1_SD_OT1.1 | TCTGCCCCTGGATAGCCTCC | B2M_Ex1_SD_OT1 Fwd 1 | TCGTCGGCAGCGTCAGATGTGTATAAGAGACAGCCCAGACATGAGAAGGTTAT |
| B2M_Ex1_SD_OT1.1 | TCTGCCCCTGGATAGCCTCC | B2M_Ex1_SD_OT1 Rev 1 | GTCTCGTGGGCTCGGAGATGTGTATAAGAGACAGCTTTACAGGCTCCCCTTC |
| B2M_Ex1_SD_OT1.1 | TCTGCCCCTGGATAGCCTCC | B2M_Ex1_SD_OT1 Fwd 2 | TCGTCGGCAGCGTCAGATGTGTATAAGAGACAGCCCAGACATGAGAAGGTTAT |
| B2M_Ex1_SD_OT1.1 | TCTGCCCCTGGATAGCCTCC | B2M_Ex1_SD_OT1 Rev 2 | GTCTCGTGGGCTCGGAGATGTGTATAAGAGACAGGTCTAGGCGTCCTCATCAC |
| B2M_Ex1_SD_OT2.1 | ACTCACCTTCCATAGCCTCC | B2M_Ex1_SD_OT2 Fwd 1 | TCGTCGGCAGCGTCAGATGTGTATAAGAGACAGGGATGTTCTTTGGTGTTTGT |
| B2M_Ex1_SD_OT2.1 | ACTCACCTTCCATAGCCTCC | B2M_Ex1_SD_OT2 Rev 1 | GTCTCGTGGGCTCGGAGATGTGTATAAGAGACAGGACTCCGTCTCTGAACACTC |
| B2M_Ex1_SD_OT2.1 | ACTCACCTTCCATAGCCTCC | B2M_Ex1_SD_OT2 Fwd 2 | TCGTCGGCAGCGTCAGATGTGTATAAGAGACAGGAATGTTGGGATGTTCTTTG |
| B2M_Ex1_SD_OT2.1 | ACTCACCTTCCATAGCCTCC | B2M_Ex1_SD_OT2 Rev 2 | GTCTCGTGGGCTCGGAGATGTGTATAAGAGACAGGACTCCGTCTCTGAACACTC |
| B2M_Ex1_SD_OT3.1 | GCTCCTGCTGCATAGCCTCC | B2M_Ex1_SD_OT3 Fwd 1 | TCGTCGGCAGCGTCAGATGTGTATAAGAGACAGATAGTTGCCATTTCTGCTTG |
| B2M_Ex1_SD_OT3.1 | GCTCCTGCTGCATAGCCTCC | B2M_Ex1_SD_OT3 Rev 1 | GTCTCGTGGGCTCGGAGATGTGTATAAGAGACAGGAGGGTGAAAGACTGAAAAA |
| B2M_Ex1_SD_OT3.1 | GCTCCTGCTGCATAGCCTCC | B2M_Ex1_SD_OT3 Fwd 2 | TCGTCGGCAGCGTCAGATGTGTATAAGAGACAGAATAGTTGCCATTTCTGCTT |
| B2M_Ex1_SD_OT3.1 | GCTCCTGCTGCATAGCCTCC | B2M_Ex1_SD_OT3 Rev 2 | GTCTCGTGGGCTCGGAGATGTGTATAAGAGACAGGAGGGTGAAAGACTGAAAAA |
| B2M_Ex1_SD_OT4.1 | TCTCACTGTGGTTAGCCTCC | B2M_Ex1_SD_OT4 Fwd 1 | TCGTCGGCAGCGTCAGATGTGTATAAGAGACAGTCTTTTTGTGAAGGCTTTTC |
| B2M_Ex1_SD_OT4.1 | TCTCACTGTGGTTAGCCTCC | B2M_Ex1_SD_OT4 Rev 1 | GTCTCGTGGGCTCGGAGATGTGTATAAGAGACAGTTTGTCACCAGCTGATTGTA |
| B2M_Ex1_SD_OT4.1 | TCTCACTGTGGTTAGCCTCC | B2M_Ex1_SD_OT4 Fwd 2 | TCGTCGGCAGCGTCAGATGTGTATAAGAGACAGTCTTTTTGTGAAGGCTTTTC |
| B2M_Ex1_SD_OT4.1 | TCTCACTGTGGTTAGCCTCC | B2M_Ex1_SD_OT4 Rev 2 | GTCTCGTGGGCTCGGAGATGTGTATAAGAGACAGTGTCACCAGCTGATTGTAGA |
| B2M_Ex1_SD_OT5.1 | TCTCTCACTGGATAGCCTAC | B2M_Ex1_SD_OT5 Fwd 1 | TCGTCGGCAGCGTCAGATGTGTATAAGAGACAGCTCAAGAGCAAGAAACCAGT |
| B2M_Ex1_SD_OT5.1 | TCTCTCACTGGATAGCCTAC | B2M_Ex1_SD_OT5 Rev 1 | GTCTCGTGGGCTCGGAGATGTGTATAAGAGACAGAGGACTTTGAAATACCAGCA |

| | | | |
|---|---|---|---|
| B2M_Ex1_SD_OT5.1 | TCTCTCACTGGATAGCCTAC | B2M_Ex1_SD_OT5 Fwd 2 | TCGTCGGCAGCGTCAGATGTGTATAAGAGACAGGTGAGTCAGCAGCTCAAGA |
| B2M_Ex1_SD_OT5.1 | TCTCTCACTGGATAGCCTAC | B2M_Ex1_SD_OT5 Rev 2 | GTCTCGTGGGCTCGGAGATGTGTATAAGAGACAGAGGACTTTGAAATACCAGCA |
| B2M_Ex1_SD_OT6.1 | TCTCTCACTGGATAGCCTAC | B2M_Ex1_SD_OT6 Fwd 1 | TCGTCGGCAGCGTCAGATGTGTATAAGAGACAGCTCAAGAGCAAGAAACCAGT |
| B2M_Ex1_SD_OT6.1 | TCTCTCACTGGATAGCCTAC | B2M_Ex1_SD_OT6 Rev 1 | GTCTCGTGGGCTCGGAGATGTGTATAAGAGACAGAGGACTTTGAAATACCAGCA |
| B2M_Ex1_SD_OT6.1 | TCTCTCACTGGATAGCCTAC | B2M_Ex1_SD_OT6 Fwd 2 | TCGTCGGCAGCGTCAGATGTGTATAAGAGACAGGTGAGTCAGCAGCTCAAGA |
| B2M_Ex1_SD_OT6.1 | TCTCTCACTGGATAGCCTAC | B2M_Ex1_SD_OT6 Rev 2 | GTCTCGTGGGCTCGGAGATGTGTATAAGAGACAGAGGACTTTGAAATACCAGCA |
| B2M_Ex1_SD_OT7.1 | TCTCTCACTGGATAGCCTAC | B2M_Ex1_SD_OT7 Fwd 1 | TCGTCGGCAGCGTCAGATGTGTATAAGAGACAGCTCAAGAGCAAGAAACCAGT |
| B2M_Ex1_SD_OT7.1 | TCTCTCACTGGATAGCCTAC | B2M_Ex1_SD_OT7 Rev 1 | GTCTCGTGGGCTCGGAGATGTGTATAAGAGACAGAGGACTTTGAAATACCAGCA |
| B2M_Ex1_SD_OT7.1 | TCTCTCACTGGATAGCCTAC | B2M_Ex1_SD_OT7 Fwd 2 | TCGTCGGCAGCGTCAGATGTGTATAAGAGACAGGTGAGTCAGCAGCTCAAGA |
| B2M_Ex1_SD_OT7.1 | TCTCTCACTGGATAGCCTAC | B2M_Ex1_SD_OT7 Rev 2 | GTCTCGTGGGCTCGGAGATGTGTATAAGAGACAGAGGACTTTGAAATACCAGCA |
| B2M_Ex1_SD_OT8.1 | AGCCACCCTGGAGAGCCTCC | B2M_Ex1_SD_OT8 Fwd 1 | TCGTCGGCAGCGTCAGATGTGTATAAGAGACAGAGCTCAGCTTGCTCCACT |
| B2M_Ex1_SD_OT8.1 | AGCCACCCTGGAGAGCCTCC | B2M_Ex1_SD_OT8 Rev 1 | GTCTCGTGGGCTCGGAGATGTGTATAAGAGACAGGCTTCTTGAGGCTGTTCA |
| B2M_Ex1_SD_OT8.1 | AGCCACCCTGGAGAGCCTCC | B2M_Ex1_SD_OT8 Fwd 2 | TCGTCGGCAGCGTCAGATGTGTATAAGAGACAGAGCTCAGCTTGCTCCACT |
| B2M_Ex1_SD_OT8.1 | AGCCACCCTGGAGAGCCTCC | B2M_Ex1_SD_OT8 Rev 2 | GTCTCGTGGGCTCGGAGATGTGTATAAGAGACAGCTTCTTGAGGCTGTTCAGG |
| B2M_Ex1_SD_OT9.1 | ACTCCCGCTGGAAAGCCTGC | B2M_Ex1_SD_OT9 Fwd 1 | TCGTCGGCAGCGTCAGATGTGTATAAGAGACAGCTTTCGCCAGCTCAAAAA |
| B2M_Ex1_SD_OT9.1 | ACTCCCGCTGGAAAGCCTGC | B2M_Ex1_SD_OT9 Rev 1 | GTCTCGTGGGCTCGGAGATGTGTATAAGAGACAGCAGCGAGCTCTACTGGTG |
| B2M_Ex1_SD_OT9.1 | ACTCCCGCTGGAAAGCCTGC | B2M_Ex1_SD_OT9 Fwd 2 | TCGTCGGCAGCGTCAGATGTGTATAAGAGACAGCTTTCGCCAGCTCAAAAA |
| B2M_Ex1_SD_OT9.1 | ACTCCCGCTGGAAAGCCTGC | B2M_Ex1_SD_OT9 Rev 2 | GTCTCGTGGGCTCGGAGATGTGTATAAGAGACAGCGAGCTCTACTGGTGCTG |
| B2M_Ex1_SD_OT10.1 | CCTCCCGCTGTGTAGCCTCC | B2M_Ex1_SD_OT10 Fwd 1 | TCGTCGGCAGCGTCAGATGTGTATAAGAGACAGGGTGGATTACAATGGAAACA |
| B2M_Ex1_SD_OT10.1 | CCTCCCGCTGTGTAGCCTCC | B2M_Ex1_SD_OT10 Rev 1 | GTCTCGTGGGCTCGGAGATGTGTATAAGAGACAGCATGAAAGGGGGTTATACAT |
| B2M_Ex1_SD_OT10.1 | CCTCCCGCTGTGTAGCCTCC | B2M_Ex1_SD_OT10 Fwd 2 | TCGTCGGCAGCGTCAGATGTGTATAAGAGACAGGGATTACAATGGAAACATCAA |
| B2M_Ex1_SD_OT10.1 | CCTCCCGCTGTGTAGCCTCC | B2M_Ex1_SD_OT10 Rev 2 | GTCTCGTGGGCTCGGAGATGTGTATAAGAGACAGCATGAAAGGGGGTTATACAT |
| PD-1_Ex.1_SD_OnT.1 | CACCTACCTAAGAACCATCC | PD-1_Ex.1_SD_OnT Fwd 1 | TCGTCGGCAGCGTCAGATGTGTATAAGAGACAGCTGCCAGGGACTGAGAGT |
| PD-1_Ex.1_SD_OnT.1 | CACCTACCTAAGAACCATCC | PD-1_Ex.1_SD_OnT Rev 1 | GTCTCGTGGGCTCGGAGATGTGTATAAGAGACAGGTGGATGTGGAGGAAGAG |

| | | | |
|---|---|---|---|
| PD-1_Ex.1_SD_OnT.1 | CACCTACCTAAGAACCATCC | PD-1_Ex.1_SD_OnT Fwd 2 | TCGTCGGCAGCGTCAGATGTGTATAAGAGACAGCTGCCAGGGACTGAGAGT |
| PD-1_Ex.1_SD_OnT.1 | CACCTACCTAAGAACCATCC | PD-1_Ex.1_SD_OnT Rev 2 | GTCTCGTGGGCTCGGAGATGTGTATAAGAGACAGACGTGGATGTGGAGGAAG |
| PD-1_Ex.1_SD_OT1.1 | CGGCCACCTGAGAACCATCC | PD-1_Ex.1_SD_OT1 Fwd 1 | TCGTCGGCAGCGTCAGATGTGTATAAGAGACAGTTTCACTTCTATCCCACACC |
| PD-1_Ex.1_SD_OT1.1 | CGGCCACCTGAGAACCATCC | PD-1_Ex.1_SD_OT1 Rev 1 | GTCTCGTGGGCTCGGAGATGTGTATAAGAGACAGAAAGTTCTCTGGTTCCTGTG |
| PD-1_Ex.1_SD_OT1.1 | CGGCCACCTGAGAACCATCC | PD-1_Ex.1_SD_OT1 Fwd 2 | TCGTCGGCAGCGTCAGATGTGTATAAGAGACAGTTTCACTTCTATCCCACACC |
| PD-1_Ex.1_SD_OT1.1 | CGGCCACCTGAGAACCATCC | PD-1_Ex.1_SD_OT1 Rev 2 | GTCTCGTGGGCTCGGAGATGTGTATAAGAGACAGGAAAAGTTCTCTGGTTCCTG |
| PD-1_Ex.1_SD_OT2.1 | ACCTGACCTAAGAACCATCC | PD-1_Ex.1_SD_OT2 Fwd 1 | TCGTCGGCAGCGTCAGATGTGTATAAGAGACAGGGGCCACTTGTGCTAGAG |
| PD-1_Ex.1_SD_OT2.1 | ACCTGACCTAAGAACCATCC | PD-1_Ex.1_SD_OT2 Rev 1 | GTCTCGTGGGCTCGGAGATGTGTATAAGAGACAGACTAGTGCCCATGATAGCAG |
| PD-1_Ex.1_SD_OT2.1 | ACCTGACCTAAGAACCATCC | PD-1_Ex.1_SD_OT2 Fwd 2 | TCGTCGGCAGCGTCAGATGTGTATAAGAGACAGCATGTACACGTCTGACCACT |
| PD-1_Ex.1_SD_OT2.1 | ACCTGACCTAAGAACCATCC | PD-1_Ex.1_SD_OT2 Rev 2 | GTCTCGTGGGCTCGGAGATGTGTATAAGAGACAGGAATTTACTAGTGCCCATGAT |
| PD-1_Ex.1_SD_OT3.1 | CTTCTATCTCAGAACCATCC | PD-1_Ex.1_SD_OT3 Fwd 1 | TCGTCGGCAGCGTCAGATGTGTATAAGAGACAGACACTAACGATTGCTGATGA |
| PD-1_Ex.1_SD_OT3.1 | CTTCTATCTCAGAACCATCC | PD-1_Ex.1_SD_OT3 Rev 1 | GTCTCGTGGGCTCGGAGATGTGTATAAGAGACAGTTTCTCACTCGCTCTTTCTC |
| PD-1_Ex.1_SD_OT3.1 | CTTCTATCTCAGAACCATCC | PD-1_Ex.1_SD_OT3 Fwd 2 | TCGTCGGCAGCGTCAGATGTGTATAAGAGACAGCACTAACGATTGCTGATGAC |
| PD-1_Ex.1_SD_OT3.1 | CTTCTATCTCAGAACCATCC | PD-1_Ex.1_SD_OT3 Rev 2 | GTCTCGTGGGCTCGGAGATGTGTATAAGAGACAGTTTCTCACTCGCTCTTTCTC |
| PD-1_Ex.1_SD_OT4.1 | TACCCAGCTCAGAACCATCC | PD-1_Ex.1_SD_OT4 Fwd 1 | TCGTCGGCAGCGTCAGATGTGTATAAGAGACAGTTTCTAGCTTCTGCCTTCTC |
| PD-1_Ex.1_SD_OT4.1 | TACCCAGCTCAGAACCATCC | PD-1_Ex.1_SD_OT4 Rev 1 | GTCTCGTGGGCTCGGAGATGTGTATAAGAGACAGTGCTTTTCAGAATTGATGTG |
| PD-1_Ex.1_SD_OT4.1 | TACCCAGCTCAGAACCATCC | PD-1_Ex.1_SD_OT4 Fwd 2 | TCGTCGGCAGCGTCAGATGTGTATAAGAGACAGATTTCTAGCTTCTGCCTTCTC |
| PD-1_Ex.1_SD_OT4.1 | TACCCAGCTCAGAACCATCC | PD-1_Ex.1_SD_OT4 Rev 2 | GTCTCGTGGGCTCGGAGATGTGTATAAGAGACAGTGCTTTTCAGAATTGATGTG |
| PD-1_Ex.1_SD_OT5.1 | CACTCACTTAAGTACCATCC | PD-1_Ex.1_SD_OT5 Fwd 1 | TCGTCGGCAGCGTCAGATGTGTATAAGAGACAGTGTAGTTCAGGGCTGTTAGG |
| PD-1_Ex.1_SD_OT5.1 | CACTCACTTAAGTACCATCC | PD-1_Ex.1_SD_OT5 Rev 1 | GTCTCGTGGGCTCGGAGATGTGTATAAGAGACAGTTTCAAAACTAACCAATCTGC |
| PD-1_Ex.1_SD_OT5.1 | CACTCACTTAAGTACCATCC | PD-1_Ex.1_SD_OT5 Fwd 2 | TCGTCGGCAGCGTCAGATGTGTATAAGAGACAGGCTGTTAGGGAGACTGAGAA |
| PD-1_Ex.1_SD_OT5.1 | CACTCACTTAAGTACCATCC | PD-1_Ex.1_SD_OT5 Rev 2 | GTCTCGTGGGCTCGGAGATGTGTATAAGAGACAGTTTCAAAACTAACCAATCTGC |
| PD-1_Ex.1_SD_OT6.1 | CACAAACCTGAGAACCATCG | PD-1_Ex.1_SD_OT6 Fwd 1 | TCGTCGGCAGCGTCAGATGTGTATAAGAGACAGGTGAGGAGGCATCCGAGT |
| PD-1_Ex.1_SD_OT6.1 | CACAAACCTGAGAACCATCG | PD-1_Ex.1_SD_OT6 Rev 1 | GTCTCGTGGGCTCGGAGATGTGTATAAGAGACAGGCAAATTAAAGAATCTCTGAAAA |

| | | | |
|---|---|---|---|
| PD-1_Ex.1_SD_OT6.1 | CACAAACCTGAGAACCATCG | PD-1_Ex.1_SD_OT6 Fwd 2 | TCGTCGGCAGCGTCAGATGTGTATAAGAGACAGGAAGGAGGGGGTGAGGAG |
| PD-1_Ex.1_SD_OT6.1 | CACAAACCTGAGAACCATCG | PD-1_Ex.1_SD_OT6 Rev 2 | GTCTCGTGGGCTCGGAGATGTGTATAAGAGACAGAAAGTAAAGCATTTCTGAATCC |
| PD-1_Ex.1_SD_OT7.1 | CACCTCCATTTGAACCATCC | PD-1_Ex.1_SD_OT7 Fwd 1 | TCGTCGGCAGCGTCAGATGTGTATAAGAGACAGACCCTGCACAGAACCTATAA |
| PD-1_Ex.1_SD_OT7.1 | CACCTCCATTTGAACCATCC | PD-1_Ex.1_SD_OT7 Rev 1 | GTCTCGTGGGCTCGGAGATGTGTATAAGAGACAGAAGGACTTGGCTTGTCTTCT |
| PD-1_Ex.1_SD_OT7.1 | CACCTCCATTTGAACCATCC | PD-1_Ex.1_SD_OT7 Fwd 2 | TCGTCGGCAGCGTCAGATGTGTATAAGAGACAGACCCTGCACAGAACCTATAA |
| PD-1_Ex.1_SD_OT7.1 | CACCTCCATTTGAACCATCC | PD-1_Ex.1_SD_OT7 Rev 2 | GTCTCGTGGGCTCGGAGATGTGTATAAGAGACAGGACTTGGCTTGTCTTCTGAT |
| PD-1_Ex.1_SD_OT8.1 | CACCCACCTAAGCACCATCT | PD-1_Ex.1_SD_OT8 Fwd 1 | TCGTCGGCAGCGTCAGATGTGTATAAGAGACAGCCTATTTCATATTGGGTGGA |
| PD-1_Ex.1_SD_OT8.1 | CACCCACCTAAGCACCATCT | PD-1_Ex.1_SD_OT8 Rev 1 | GTCTCGTGGGCTCGGAGATGTGTATAAGAGACAGTTCAAAACACAGGGAAAACT |
| PD-1_Ex.1_SD_OT8.1 | CACCCACCTAAGCACCATCT | PD-1_Ex.1_SD_OT8 Fwd 2 | TCGTCGGCAGCGTCAGATGTGTATAAGAGACAGTTTCATATTGGGTGGATTGT |
| PD-1_Ex.1_SD_OT8.1 | CACCCACCTAAGCACCATCT | PD-1_Ex.1_SD_OT8 Rev 2 | GTCTCGTGGGCTCGGAGATGTGTATAAGAGACAGTTCAAAACACAGGGAAAACT |
| PD-1_Ex.1_SD_OT9.1 | CACCTTCATCAGAACCATCT | PD-1_Ex.1_SD_OT9 Fwd 1 | TCGTCGGCAGCGTCAGATGTGTATAAGAGACAGGCAGCATCACCTGTGTAAC |
| PD-1_Ex.1_SD_OT9.1 | CACCTTCATCAGAACCATCT | PD-1_Ex.1_SD_OT9 Rev 1 | GTCTCGTGGGCTCGGAGATGTGTATAAGAGACAGGAAGTAAAAAGCAGAGGAAGC |
| PD-1_Ex.1_SD_OT9.1 | CACCTTCATCAGAACCATCT | PD-1_Ex.1_SD_OT9 Fwd 2 | TCGTCGGCAGCGTCAGATGTGTATAAGAGACAGAGCAGCATCACCTGTGTAA |
| PD-1_Ex.1_SD_OT9.1 | CACCTTCATCAGAACCATCT | PD-1_Ex.1_SD_OT9 Rev 2 | GTCTCGTGGGCTCGGAGATGTGTATAAGAGACAGGAAGTAAAAAGCAGAGGAAGC |
| PD-1_Ex.1_SD_OT10.1 | CAGCTATCTCAGAACCTTCC | PD-1_Ex.1_SD_OT10 Fwd 1 | TCGTCGGCAGCGTCAGATGTGTATAAGAGACAGTACATGTTTTCTATATGAGGCATT |
| PD-1_Ex.1_SD_OT10.1 | CAGCTATCTCAGAACCTTCC | PD-1_Ex.1_SD_OT10 Rev 1 | GTCTCGTGGGCTCGGAGATGTGTATAAGAGACAGCTTCCTCTAAGTCTCAGCTCAT |
| PD-1_Ex.1_SD_OT10.1 | CAGCTATCTCAGAACCTTCC | PD-1_Ex.1_SD_OT10 Fwd 2 | TCGTCGGCAGCGTCAGATGTGTATAAGAGACAGTTTTCTATTTTTCCATACTTTTTAG |
| PD-1_Ex.1_SD_OT10.1 | CAGCTATCTCAGAACCTTCC | PD-1_Ex.1_SD_OT10 Rev 2 | GTCTCGTGGGCTCGGAGATGTGTATAAGAGACAGAGCCTTTCAGATTAGTCAGG |
| TRAC2_Ex.3_SA_OnT.1 | TTCGTATCTGTAAAACCAAG | TRAC2_Ex.3_SA_OnT Fwd 1 | TCGTCGGCAGCGTCAGATGTGTATAAGAGACAGTCTCAGAGCTTAGGATGCAC |
| TRAC2_Ex.3_SA_OnT.1 | TTCGTATCTGTAAAACCAAG | TRAC2_Ex.3_SA_OnT Rev 1 | GTCTCGTGGGCTCGGAGATGTGTATAAGAGACAGTCTTGAAACACAATACTGTTGG |
| TRAC2_Ex.3_SA_OnT.1 | TTCGTATCTGTAAAACCAAG | TRAC2_Ex.3_SA_OnT Fwd 2 | TCGTCGGCAGCGTCAGATGTGTATAAGAGACAGTCTCAGAGCTTAGGATGCAC |
| TRAC2_Ex.3_SA_OnT.1 | TTCGTATCTGTAAAACCAAG | TRAC2_Ex.3_SA_OnT Rev 2 | GTCTCGTGGGCTCGGAGATGTGTATAAGAGACAGCTTGAAACACAATACTGTTGG |
| TRAC2_Ex.3_SA_OT1.1 | TTGGGATCTTTAAAACCAAG | TRAC2_Ex.3_SA_OT1 Fwd 1 | TCGTCGGCAGCGTCAGATGTGTATAAGAGACAGACATACATTGCCTTACTTTGC |
| TRAC2_Ex.3_SA_OT1.1 | TTGGGATCTTTAAAACCAAG | TRAC2_Ex.3_SA_OT1 Rev 1 | GTCTCGTGGGCTCGGAGATGTGTATAAGAGACAGTTTTTGACTGCCAGAAGGT |

| | | | |
|---|---|---|---|
| TRAC2_Ex.3_SA_OT1.1 | TTGGGATCTTTAAAACCAAG | TRAC2_Ex.3_SA_OT1 Fwd 2 | TCGTCGGCAGCGTCAGATGTGTATAAGAGACAGACATACATTGCCTTACTTTGC |
| TRAC2_Ex.3_SA_OT1.1 | TTGGGATCTTTAAAACCAAG | TRAC2_Ex.3_SA_OT1 Rev 2 | GTCTCGTGGGCTCGGAGATGTGTATAAGAGACAGGGAAGCCAAAAGTTATACATGA |
| TRAC2_Ex.3_SA_OT2.1 | TGAGCATCTGTAAAACCAAG | TRAC2_Ex.3_SA_OT2 Fwd 1 | TCGTCGGCAGCGTCAGATGTGTATAAGAGACAGAGTTTGGCATCTTCTTTACCT |
| TRAC2_Ex.3_SA_OT2.1 | TGAGCATCTGTAAAACCAAG | TRAC2_Ex.3_SA_OT2 Rev 1 | GTCTCGTGGGCTCGGAGATGTGTATAAGAGACAGAGTGGGGCTTCTCATCAC |
| TRAC2_Ex.3_SA_OT2.1 | TGAGCATCTGTAAAACCAAG | TRAC2_Ex.3_SA_OT2 Fwd 2 | TCGTCGGCAGCGTCAGATGTGTATAAGAGACAGAAGTTTGGCATCTTCTTTACC |
| TRAC2_Ex.3_SA_OT2.1 | TGAGCATCTGTAAAACCAAG | TRAC2_Ex.3_SA_OT2 Rev 2 | GTCTCGTGGGCTCGGAGATGTGTATAAGAGACAGAGTGGGGCTTCTCATCAC |
| TRAC2_Ex.3_SA_OT3.1 | TTCTAATCTCTAAAACCAAG | TRAC2_Ex.3_SA_OT3 Fwd 1 | TCGTCGGCAGCGTCAGATGTGTATAAGAGACAGAATGATAGATCCCAGCTGAA |
| TRAC2_Ex.3_SA_OT3.1 | TTCTAATCTCTAAAACCAAG | TRAC2_Ex.3_SA_OT3 Rev 1 | GTCTCGTGGGCTCGGAGATGTGTATAAGAGACAGCTTTCCTCCTTGCATGTATT |
| TRAC2_Ex.3_SA_OT3.1 | TTCTAATCTCTAAAACCAAG | TRAC2_Ex.3_SA_OT3 Fwd 2 | TCGTCGGCAGCGTCAGATGTGTATAAGAGACAGGGAGCCACAGATTAAATGAT |
| TRAC2_Ex.3_SA_OT3.1 | TTCTAATCTCTAAAACCAAG | TRAC2_Ex.3_SA_OT3 Rev 2 | GTCTCGTGGGCTCGGAGATGTGTATAAGAGACAGTCTTTCCTCCTTGCATGTAT |
| TRAC2_Ex.3_SA_OT4.1 | GTGGTATCTGCAAAACCAAG | TRAC2_Ex.3_SA_OT4 Fwd 1 | TCGTCGGCAGCGTCAGATGTGTATAAGAGACAGTCATGAATGGTGACTCAGAA |
| TRAC2_Ex.3_SA_OT4.1 | GTGGTATCTGCAAAACCAAG | TRAC2_Ex.3_SA_OT4 Rev 1 | GTCTCGTGGGCTCGGAGATGTGTATAAGAGACAGAAATGCCAGCCACTTTTT |
| TRAC2_Ex.3_SA_OT4.1 | GTGGTATCTGCAAAACCAAG | TRAC2_Ex.3_SA_OT4 Fwd 2 | TCGTCGGCAGCGTCAGATGTGTATAAGAGACAGAAATCATGAATGGTGACTCAG |
| TRAC2_Ex.3_SA_OT4.1 | GTGGTATCTGCAAAACCAAG | TRAC2_Ex.3_SA_OT4 Rev 2 | GTCTCGTGGGCTCGGAGATGTGTATAAGAGACAGAAATGCCAGCCACTTTTT |
| TRAC2_Ex.3_SA_OT5.1 | TCCTCATGTGTAAAACCAAG | TRAC2_Ex.3_SA_OT5 Fwd 1 | TCGTCGGCAGCGTCAGATGTGTATAAGAGACAGGCCAAACCATATTAGCAAAC |
| TRAC2_Ex.3_SA_OT5.1 | TCCTCATGTGTAAAACCAAG | TRAC2_Ex.3_SA_OT5 Rev 1 | GTCTCGTGGGCTCGGAGATGTGTATAAGAGACAGTTGAGTTCATGAGAATCGTG |
| TRAC2_Ex.3_SA_OT5.1 | TCCTCATGTGTAAAACCAAG | TRAC2_Ex.3_SA_OT5 Fwd 2 | TCGTCGGCAGCGTCAGATGTGTATAAGAGACAGGCCAAACCATATTAGCAAAC |
| TRAC2_Ex.3_SA_OT5.1 | TCCTCATGTGTAAAACCAAG | TRAC2_Ex.3_SA_OT5 Rev 2 | GTCTCGTGGGCTCGGAGATGTGTATAAGAGACAGATTGAGTTCATGAGAATCGTG |
| TRAC2_Ex.3_SA_OT6.1 | TCTGCATCTTTAAAACCAAG | TRAC2_Ex.3_SA_OT6 Fwd 1 | TCGTCGGCAGCGTCAGATGTGTATAAGAGACAGGCAAACGTACACTGTAATGC |
| TRAC2_Ex.3_SA_OT6.1 | TCTGCATCTTTAAAACCAAG | TRAC2_Ex.3_SA_OT6 Rev 1 | GTCTCGTGGGCTCGGAGATGTGTATAAGAGACAGGCTTTGCTGAGACCATAGAT |
| TRAC2_Ex.3_SA_OT6.1 | TCTGCATCTTTAAAACCAAG | TRAC2_Ex.3_SA_OT6 Fwd 2 | TCGTCGGCAGCGTCAGATGTGTATAAGAGACAGGCAAACGTACACTGTAATGC |
| TRAC2_Ex.3_SA_OT6.1 | TCTGCATCTTTAAAACCAAG | TRAC2_Ex.3_SA_OT6 Rev 2 | GTCTCGTGGGCTCGGAGATGTGTATAAGAGACAGATGCTTTGCTGAGACCATAG |
| TRAC2_Ex.3_SA_OT7.1 | TTTGTATCTTTAAAACCATG | TRAC2_Ex.3_SA_OT7 Fwd 1 | TCGTCGGCAGCGTCAGATGTGTATAAGAGACAGCAAAGTGCTGGGATTACAGA |
| TRAC2_Ex.3_SA_OT7.1 | TTTGTATCTTTAAAACCATG | TRAC2_Ex.3_SA_OT7 Rev 1 | GTCTCGTGGGCTCGGAGATGTGTATAAGAGACAGTGCAAAGTTTTATGTAGTTTAAGTG |

| | | | |
|---|---|---|---|
| TRAC2_Ex.3_SA_OT7.1 | TTTGTATCTTTAAAACCATG | TRAC2_Ex.3_SA_OT7 Fwd 2 | TCGTCGGCAGCGTCAGATGTGTATAAGAGACAGCAAAGTGCTGGGATTACAGA |
| TRAC2_Ex.3_SA_OT7.1 | TTTGTATCTTTAAAACCATG | TRAC2_Ex.3_SA_OT7 Rev 2 | GTCTCGTGGGCTCGGAGATGTGTATAAGAGACAGAATTTGCAAAGTTTTATGTAGTTT |
| TRAC2_Ex.3_SA_OT8.1 | TTCTTATGTGTGAAACCAAG | TRAC2_Ex.3_SA_OT8 Fwd 1 | TCGTCGGCAGCGTCAGATGTGTATAAGAGACAGTGGGACTCTTGGTTCTGTAT |
| TRAC2_Ex.3_SA_OT8.1 | TTCTTATGTGTGAAACCAAG | TRAC2_Ex.3_SA_OT8 Rev 1 | GTCTCGTGGGCTCGGAGATGTGTATAAGAGACAGTTTTTGTTGTTGTTTTACTTGAA |
| TRAC2_Ex.3_SA_OT8.1 | TTCTTATGTGTGAAACCAAG | TRAC2_Ex.3_SA_OT8 Fwd 2 | TCGTCGGCAGCGTCAGATGTGTATAAGAGACAGTTGGGACTCTTGGTTCTGTA |
| TRAC2_Ex.3_SA_OT8.1 | TTCTTATGTGTGAAACCAAG | TRAC2_Ex.3_SA_OT8 Rev 2 | GTCTCGTGGGCTCGGAGATGTGTATAAGAGACAGTTTTTGTTGTTGTTTTACTTGAA |
| TRAC2_Ex.3_SA_OT9.1 | CTCTCTTCTGTAAAACCAAG | TRAC2_Ex.3_SA_OT9 Fwd 1 | TCGTCGGCAGCGTCAGATGTGTATAAGAGACAGAAATTCTTCTTGGGCTCAG |
| TRAC2_Ex.3_SA_OT9.1 | CTCTCTTCTGTAAAACCAAG | TRAC2_Ex.3_SA_OT9 Rev 1 | GTCTCGTGGGCTCGGAGATGTGTATAAGAGACAGGCTCATTACTGGGTTAAGCA |
| TRAC2_Ex.3_SA_OT9.1 | CTCTCTTCTGTAAAACCAAG | TRAC2_Ex.3_SA_OT9 Fwd 2 | TCGTCGGCAGCGTCAGATGTGTATAAGAGACAGGAAATTCTTCTTGGGCTCAG |
| TRAC2_Ex.3_SA_OT9.1 | CTCTCTTCTGTAAAACCAAG | TRAC2_Ex.3_SA_OT9 Rev 2 | GTCTCGTGGGCTCGGAGATGTGTATAAGAGACAGGCTCATTACTGGGTTAAGCA |
| TRAC2_Ex.3_SA_OT10.1 | TGGGTTTCTTTAAAACCAAG | TRAC2_Ex.3_SA_OT10 Fwd 1 | TCGTCGGCAGCGTCAGATGTGTATAAGAGACAGTCTAGGCTCTTGACACCATC |
| TRAC2_Ex.3_SA_OT10.1 | TGGGTTTCTTTAAAACCAAG | TRAC2_Ex.3_SA_OT10 Rev 1 | GTCTCGTGGGCTCGGAGATGTGTATAAGAGACAGGAACAACTAGACCCAATGTGA |
| TRAC2_Ex.3_SA_OT10.1 | TGGGTTTCTTTAAAACCAAG | TRAC2_Ex.3_SA_OT10 Fwd 2 | TCGTCGGCAGCGTCAGATGTGTATAAGAGACAGGTCTAGGCTCTTGACACCAT |
| TRAC2_Ex.3_SA_OT10.1 | TGGGTTTCTTTAAAACCAAG | TRAC2_Ex.3_SA_OT10 Rev 2 | GTCTCGTGGGCTCGGAGATGTGTATAAGAGACAGGAACAACTAGACCCAATGTGA |

**Supplemental Table 3. Primer and probe sequences used for ddPCR analysis.**

| Translocation | Forward Primer | Probe | Reverse Primer |
|---|---|---|---|
| *B2M* Exon 3 Reference | GGTTTCATCCATCCGACATTGAAGTTGAC | GACCAGTCCTTGCTGAAAGACAAGTCTG | GGGTGAATTCAGTGTAGTACAAGAGATAG |
| *PDCD1:B2M* | GGCATGCAGATCCCACAG | AAGTCACGGAGCGAGAGAGCAC | GGCCACCAAGGAGAACTTG |
| *PDCD1:TRAC* | GGCATGCAGATCCCACAG | CCTGTCAGTGATTGGGTTCCGAATCCTCCTCC | CATGAGCAGATTAAACCCGGCCAC |
| *B2M:TRAC* | ATGTCTCGCTCCGTGGCCTTAG | CCTGTCAGTGATTGGGTTCCGAATCCTCCTCC | CATGAGCAGATTAAACCCGGCCAC |
| *B2M:PDCD1* | GGGCATTCCTGAAGCTGAC | CCTTAGCTGTGCTCGCGCTACT | AGGGACTGAGGGTGGAAG |
| *TRAC:B2M* | CAGCCTGCTCTGCCTTG | CATGCAAGCCCATAACCGCTGTG | AAGTCACGGAGCGAGAGA |
| *TRAC:PDCD1* | CAGCCTGCTCTGCCTTG | CATGCAAGCCCATAACCGCTGTG | AGGGACTGAGGGTGGAAG |
| *TRAC:PD1 OT2.1* | CAGCCTGCTCTGCCTTG | CATGCAAGCCCATAACCGCTGTG | GTTGGCTAAGAATCTGAGAAGGG |
| *B2M:PD1 OT2.1* | GGGCATTCCTGAAGCTGAC | CCTTAGCTGTGCTCGCGCTACT | GTTGGCTAAGAATCTGAGAAGGG |
| *PD1:PD1 OT2.1* | GGCATGCAGATCCCACAG | TCTGGGCGGTGCTACAACTGG | TCTGGGCGGTGCTACAACTGG |
| *PD1 OT2.1:TRAC* | AGAGAGAGAGACGCATGGTCAACC | CCTGTCAGTGATTGGGTTCCGAATCCTCCTCC | CATGAGCAGATTAAACCCGGCCAC |
| *PD1 OT2.1:B2M* | CCACTGTTTTACTTCTAGCCAGTC | AAGTCACGGAGCGAGAGAGCAC | GGCCACCAAGGAGAACTTG |
| *PD1 OT2.1:PD1* | CCACTGTTTTACTTCTAGCCAGTC | CAGGGACTGAGGGTGGAAGGTC | CAGGGACTGAGAGTGAAAGGTC |

**Supplemental Table 4. Primers used for Sanger sequencing and EditR analysis.**

| Target Site | Primer Name | Primer Sequence |
|---|---|---|
| **PDCD1 Ex1 SD** | PD1 Ex1 SD Fwd | ACCCTCCCTTCAACCTGAC |
| | PD1 Ex1 SD Rev | AAGCCACACAGCTCAGGGTA |
| **PDCD1 Ex2** | PD1 Ex2 Fwd | GCCTCACGTAGAAGGAAGAGG |
| | PD1 Ex2 Rev | CAGTTGTGTGACACGGAAGC |
| **PDCD1 Ex3** | PD1 Ex3 Fwd | ACCGGCATCTCTGTCCTCTA |
| | PD1 Ex3 Rev | CTTAGTCCAGGGGCCTTCAT |
| **PDCD1 Ex4** | PD1 Ex4 Fwd | AAACCCTGGTGGTTGGTGTCG |
| | PD1 Ex4 Rev | GGTCCTGGCTATAATAGAATGTGAGTCC |
| **TRAC Ex1** | TRAC Ex1 Fwd | TCACGAGCAGCTGGTTTCTA |
| | TRAC Ex1 Rev | CCATTCCTGAAGCAAGGAAA |
| **TRAC Ex3** | TRAC Ex3 Fwd | GGACCCGAGGTATTGTGATG |
| | TRAC Ex3 Rev | TTAGGATGCACCCAGAGACC |
| **B2M Ex1** | B2M Ex1 Fwd | TTGGAGACAGGTGACGGTCC |
| | B2M Ex1 Rev | TTATCGACGCCCTAAACTTTGTCC |
| **B2M Ex2** | B2M Ex2 Fwd | GGTGCCTGATATAGCTTGACACC |
| | B2M Ex2 Rev | GACTCATTCAGGGTAGTATGGCC |
| **B2M Ex3** | B2M Ex3 Fwd | GTTTGTAAGTCCTGCTGTCCTAGC |
| | B2M Ex3 Rev | CCTGTAGGATTCTTCTTTCCCTGC |

**Supplemental Table 5. Primer sequences used for OT RNA editing analysis.**

| Primer | Sequence | Product Size |
|---|---|---|
| CDV3 cDNA Ex.5 Fwd 1 | AGCCTTTTGGTGTGTCTTTA | 525 |
| CDV3 cDNA Ex.5 Rev 1 | GCTCAGTTACTCACCCAAAT | |
| CDV3 cDNA Ex.5 Fwd 2 | AGCGTTTTCAGATAGGAGTTT | 522 |
| CDV3 cDNA Ex.5 Rev 2 | GTACAATGGACAGCCTCATC | |
| CYCS cDNA Ex.3 Fwd | GGTATCACTGTTTACCCCTTT | 510 |
| CYCS cDNA Ex.3 Rev | TGGCTGTTTAAAGACAATCC | |
| DDX21 cDNA Ex.15 Fwd | CTCAAAAGTGTCCCTTAATAGTG | 553 |
| DDX21 cDNA Ex.15 Rev | TCTTGAGCAATGCTAATCTG | |
| RPL37 cDNA Ex.4 Fwd 1 | CTTGACCTGACCCATGTATT | 420 |
| RPL37 cDNA Ex.4 Rev 1 | CAAGGATAACTACCCAGCAG | |
| RPL37 cDNA Ex.4 Fwd 2 | ACCCCTTCTGTATCCTTGAT | 384 |
| RPL37 cDNA Ex.4 Rev 2 | GCCTTTCTCCTGTACTGTTTT | |
| RPS12 cDNA Fwd | TGCGTTCAAGATTCAACTTC | 458 |
| RPS12 cDNA Rev | TGTGAGCCAAAGATTTATTTC | |

**Supplemental Note:** Amino Acid Sequence of coBE4.

MSSETGPVAVDPTLRRRIEPHEFEVFFDPRELRKETCLLYEINWGGRHSIWRHTSQNT
NKHVEVNFIEKFTTERYFCPNTRCSITWFLSWSPCGECSRAITEFLSRYPHVTLFIYIAR
LYHHADPRNRQGLRDLISSGVTIQIMTEQESGYCWRNFVNYSPSNEAHWPRYPHLWV
RLYVLELYCIILGLPPCLNILRRKQPQLTFFTIALQSCHYQRLPPHILWATGLKSGGSSG
GSSGSETPGTSESATPESSGGSSGGSDKKYSIGLAIGTNSVGWAVITDEYKVPSKKFK
VLGNTDRHSIKKNLIGALLFDSGETAEATRLKRTARRRYTRRKNRICYLQEIFSNEMAKV
DDSFFHRLEESFLVEEDKKHERHPIFGNIVDEVAYHEKYPTIYHLRKKLVDSTDKADLRL
IYLALAHMIKFRGHFLIEGDLNPDNSDVDKLFIQLVQTYNQLFEENPINASGVDAKAILSA
RLSKSRRLENLIAQLPGEKKNGLFGNLIALSLGLTPNFKSNFDLAEDAKLQLSKDTYDD
DLDNLLAQIGDQYADLFLAAKNLSDAILLSDILRVNTEITKAPLSASMIKRYDEHHQDLTL
LKALVRQQLPEKYKEIFFDQSKNGYAGYIDGGASQEEFYKFIKPILEKMDGTEELLVKLN
REDLLRKQRTFDNGSIPHQIHLGELHAILRRQEDFYPFLKDNREKIEKILTFRIPYYVGPL
ARGNSRFAWMTRKSEETITPWNFEEVVDKGASAQSFIERMTNFDKNLPNEKVLPKHS
LLYEYFTVYNELTKVKYVTEGMRKPAFLSGEQKKAIVDLLFKTNRKVTVKQLKEDYFKKI
ECFDSVEISGVEDRFNASLGTYHDLLKIIKDKDFLDNEENEDILEDIVLTLTLFEDREMIE
ERLKTYAHLFDDKVMKQLKRRRYTGWGRLSRKLINGIRDKQSGKTILDFLKSDGFANR
NFMQLIHDDSLTFKEDIQKAQVSGQGDSLHEHIANLAGSPAIKKGILQTVKVVDELVKV
MGRHKPENIVIEMARENQTTQKGQKNSRERMKRIEEGIKELGSQILKEHPVENTQLQN
EKLYLYYLQNGRDMYVDQELDINRLSDYDVDHIVPQSFLKDDSIDNKVLTRSDKNRGK
SDNVPSEEVVKKMKNYWRQLLNAKLITQRKFDNLTKAERGGLSELDKAGFIKRQLVET
RQITKHVAQILDSRMNTKYDENDKLIREVKVITLKSKLVSDFRKDFQFYKVREINNYHHA
HDAYLNAVVGTALIKKYPKLESEFVYGDYKVYDVRKMIAKSEQEIGKATAKYFFYSNIM
NFFKTEITLANGEIRKRPLIETNGETGEIVWDKGRDFATVRKVLSMPQVNIVKKTEVQTG
GFSKESILPKRNSDKLIARKKDWDPKKYGGFDSPTVAYSVLVVAKVEKGKSKKLKSVK
ELLGITIMERSSFEKNPIDFLEAKGYKEVKKDLIIKLPKYSLFELENGRKRMLASAGELQK
GNELALPSKYVNFLYLASHYEKLKGSPEDNEQKQLFVEQHKHYLDEIIEQISEFSKRVIL
ADANLDKVLSAYNKHRDKPIREQAENIIHLFTLTNLGAPAAFKYFDTTIDRKRYTSTKEV
LDATLIHQSITGLYETRIDLSQLGGDSGGSGGSGGSTNLSDIIEKETGKQLVIQESILMLP
EEVEEVIGNKPESDILVHTAYDESTDENVMLLTSDAPEYKPWALVIQDSNGENKIKMLS
GGSGGSGGSTNLSDIIEKETGKQLVIQESILMLPEEVEEVIGNKPESDILVHTAYDESTD
ENVMLLTSDAPEYKPWALVIQDSNGENKIKMLSGGSKRTADGSEFESPKKKRKVE*
