# Supplementary material for: Highly efficient multiplex human T cell engineering without double-strand breaks using Cas9 base editors
Source: Nat Commun. 2019 Nov 19;10:5222. doi: 10.1038/s41467-019-13007-6 (PMC6864045; doi:10.1038/s41467-019-13007-6)
Supplement: Supplementary file 2 — Dataset 1 [file 41467_2019_13007_MOESM2_ESM.pdf]

Highly efficient multiplex human T cell engineering without double-strand breaks using Cas9 base editors

Appendix I - Tile Plots of Next Generation Sequencing

1. A PDCD1 Ex.1 SD + BE3 unconc. mRNA 1.5 µg

	C_{-10}	C_{-9}	G_{-8}	C_{-7}	C_{-6}	G_{-5}	A_{-4}	C_{-3}	C_{-2}	C_{-1}	C_{1}	A_{2}	C_{3}	C_{4}	T_{5}	A_{6}	C_{7}	C_{8}	T_{9}	A_{10}	A_{11}	G_{12}	A_{13}	A_{14}	C_{15}	C_{16}	A_{17}	T_{18}	C_{19}	C_{20}	T_{21}	G_{22}	G_{23}	Indel
A	0	0	0.1	0	0	0.4	99	0.1	0.1	0.1	0.1	96.5	0.4	1.8	0.5	98.5	9.3	6.8	0.1	98.3	96.2	0.1	98.4	98.7	0.1	0.1	98.8	0.1	0	0	0	0	0	8.1
C	99.7	99.9	0	99.9	99.8	0.2	0.6	99.9	99.7	99.8	99.6	3.3	96.5	91.4	0.6	1.4	42.3	57.6	0.9	1.7	3.6	0.5	1.4	1.2	99.8	99.8	1	1	99.7	99.9	0.3	0.2	1	
G	0.2	0	99.9	0	0.1	99.4	0.3	0	0.1	0.1	0.1	0.2	0	0.4	0.4	0	6.4	12.2	0.4	0	0.2	99.3	0.2	0.1	0	0	0.2	0.7	0.1	0	0.2	99.5	98.8	
T	0.1	0	0	0	0.1	0	0	0	0.1	0.1	0.2	0.1	3.1	6.3	98.5	0	42.1	23.4	98.6	0	0.1	0	0	0	0.1	0.1	0	98.3	0.2	0.1	99.5	0.2	0.2	

2. B PDCD1 Ex.1 SD + BE3 unconc. mRNA 1.5 µg

	C_{-10}	C_{-9}	G_{-8}	C_{-7}	C_{-6}	G_{-5}	A_{-4}	C_{-3}	C_{-2}	C_{-1}	C_{1}	A_{2}	C_{3}	C_{4}	T_{5}	A_{6}	C_{7}	C_{8}	T_{9}	A_{10}	A_{11}	G_{12}	A_{13}	A_{14}	C_{15}	C_{16}	A_{17}	T_{18}	C_{19}	C_{20}	T_{21}	G_{22}	G_{23}	Indel
A	0	0	0.1	0	0	0.4	99.7	0	0	0	0.1	98.9	0.3	1.9	0.3	99	11.6	6.8	0.1	99.6	97.6	0.1	98.5	99.3	0.1	0	99.4	0	0.1	0	0	0	0	8.8
C	99.8	99.9	0	99.9	99.8	0.1	0.2	99.9	99.8	99.8	99.8	1.1	95.9	90.9	0.5	0.9	34.5	51.6	0.6	0.3	2.3	0.1	1.4	0.6	99.8	99.9	0.6	0.5	99.7	99.9	0.1	0.1	0.4	
G	0.1	0	99.9	0	0	99.4	0	0	0	0	0	0.1	0.1	0.8	0.1	0	5.7	14.6	0.2	0	0	99.8	0.1	0.1	0	0	0	0.2	0	0	0.1	99.8	99.5	
T	0.1	0.1	0	0	0.1	0.1	0	0	0.1	0.1	0.1	0	3.7	6.4	99.1	0.1	48.2	27	99.1	0	0	0	0	0	0.1	0.1	0	99.2	0.2	0.1	99.8	0.1	0.1	

3. C PDCD1 Ex.1 SD + BE3 unconc. mRNA 1.5 µg

	C_{-10}	C_{-9}	G_{-8}	C_{-7}	C_{-6}	G_{-5}	A_{-4}	C_{-3}	C_{-2}	C_{-1}	C_{1}	A_{2}	C_{3}	C_{4}	T_{5}	A_{6}	C_{7}	C_{8}	T_{9}	A_{10}	A_{11}	G_{12}	A_{13}	A_{14}	C_{15}	C_{16}	A_{17}	T_{18}	C_{19}	C_{20}	T_{21}	G_{22}	G_{23}	Indel
A	0	0	0.1	0	0	0.3	99.7	0	0	0	0	99.1	0.4	2.2	0.5	99.3	12.1	7.3	0.1	99.6	97.7	0	98.9	99.5	0.1	0	99.7	0	0.2	0	0	0	0	8.9
C	99.9	99.9	0	99.9	99.9	0.1	0.2	99.8	99.8	99.9	99.9	0.8	95.6	89.4	0.4	0.6	32.2	49.5	0.8	0.4	2.2	0.1	1	0.4	99.7	99.9	0.3	0.3	99.5	99.9	0.1	0.1	0.3	
G	0	0	99.9	0	0	99.5	0.1	0	0	0	0	0.1	0.1	0.5	0	0	5.6	14	0.2	0	0.1	99.8	0.1	0.1	0	0	0	0.2	0	0	0.1	99.8	99.6	
T	0	0.1	0	0	0.1	0.1	0	0.2	0.1	0	0.1	0	3.8	7.9	99	0.1	50.2	29.2	98.9	0	0	0	0	0	0.2	0	0	99.5	0.3	0	99.8	0.1	0	

4. A PDCD1 Ex.1 SD + BE4 unconc. mRNA 1.5 µg

	C_{-10}	C_{-9}	G_{-8}	C_{-7}	C_{-6}	G_{-5}	A_{-4}	C_{-3}	C_{-2}	C_{-1}	C_{1}	A_{2}	C_{3}	C_{4}	T_{5}	A_{6}	C_{7}	C_{8}	T_{9}	A_{10}	A_{11}	G_{12}	A_{13}	A_{14}	C_{15}	C_{16}	A_{17}	T_{18}	C_{19}	C_{20}	T_{21}	G_{22}	G_{23}	Indel
A	0	0	0	0.1	0	0.2	99.1	0.2	0.1	0.1	0.1	95.6	0.3	1.8	0.4	98.2	4.9	5.2	0	98.2	95.2	0.1	98.2	98.4	0.1	0.1	98.6	0.1	0.1	0	0	0	0	11.6
C	99.8	99.8	0	99.8	99.9	0.2	0.6	99.7	99.7	99.7	99.7	4.2	90.3	78.4	0.7	1.7	31.4	36.1	0.7	1.7	4.7	0.4	1.6	1.3	99.8	99.9	1.2	0.8	99.4	99.8	0.2	0.1	0.9	
G	0	0	99.9	0	0	99.5	0.2	0	0.1	0	0	0.1	0.1	0.5	0.4	0	2.9	10.2	0.4	0.1	0.1	99.5	0.2	0.2	0	0	0.2	0.7	0	0	0.2	99.6	98.9	
T	0.1	0.2	0	0	0.1	0.1	0	0.1	0.1	0.2	0.2	0	9.2	19.3	98.6	0.1	60.9	48.5	98.8	0	0	0.1	0.1	0	0.1	0.1	0	98.5	0.5	0.1	99.6	0.2	0.1	

5. B PDCD1 Ex.1 SD + BE4 unconc. mRNA 1.5 µg

	C_{-10}	C_{-9}	G_{-8}	C_{-7}	C_{-6}	G_{-5}	A_{-4}	C_{-3}	C_{-2}	C_{-1}	C_{1}	A_{2}	C_{3}	C_{4}	T_{5}	A_{6}	C_{7}	C_{8}	T_{9}	A_{10}	A_{11}	G_{12}	A_{13}	A_{14}	C_{15}	C_{16}	A_{17}	T_{18}	C_{19}	C_{20}	T_{21}	G_{22}	G_{23}	Indel
A	0	0	0.1	0	0	0.6	99.5	0	0	0	0	98.8	0.2	1.3	0.2	99	4.9	4.7	0	99.6	97.9	0.1	98.5	99.2	0	0	99.5	0	0	0	0	0	0	8.2
C	99.7	99.9	0	99.9	99.9	0.1	0.3	99.8	99.8	99.9	99.8	1.1	89.7	77.5	0.5	0.8	27.7	32.3	0.6	0.4	2.1	0.1	1.3	0.6	99.9	99.9	0.4	0.5	99.6	99.9	0.1	0.1	0.3	
G	0.1	0	99.9	0	0	99.3	0.1	0	0.1	0	0	0.1	0.1	0.4	0.1	0.1	2.7	8.3	0.2	0	0.1	99.8	0.1	0.1	0	0	0	0.1	0	0	0.1	99.8	99.6	
T	0.2	0.1	0	0.1	0.1	0	0	0.1	0.1	0.1	0.1	0	10	20.9	99.2	0.1	64.7	54.8	99.2	0.1	0	0	0	0	0.1	0.1	0	99.4	0.4	0	99.8	0.1	0.1	

6. C PDCD1 Ex.1 SD + BE4 unconc. mRNA 1.5 µg

	C_{-10}	C_{-9}	G_{-8}	C_{-7}	C_{-6}	G_{-5}	A_{-4}	C_{-3}	C_{-2}	C_{-1}	C_{1}	A_{2}	C_{3}	C_{4}	T_{5}	A_{6}	C_{7}	C_{8}	T_{9}	A_{10}	A_{11}	G_{12}	A_{13}	A_{14}	C_{15}	C_{16}	A_{17}	T_{18}	C_{19}	C_{20}	T_{21}	G_{22}	G_{23}	Indel
A	0	0.1	0.1	0	0	0.4	99.7	0.1	0	0	0	99.1	0.3	1.5	0.3	99.3	7.1	5.9	0	99.7	98.6	0	99	99.5	0	0	99.7	0	0	0	0	0	0.1	8.7
C	99.8	99.9	0	99.9	99.9	0	0.2	99.6	99.8	99.9	99.9	0.8	92.9	83.2	0.5	0.6	32.2	39.3	0.6	0.2	1.3	0.1	0.9	0.4	99.8	99.9	0.3	0.3	99.7	99.9	0.1	0	0.2	
G	0	0	99.9	0	0	99.5	0.1	0	0	0	0	0.1	0.1	0.3	0.1	0	3.4	9.7	0.2	0	0.1	99.9	0	0	0	0	0	0.1	0	0	0.1	99.8	99.7	
T	0.1	0.1	0	0.1	0.1	0.1	0	0.3	0.1	0	0.1	0	6.7	15	99.2	0.1	57.2	45.1	99.2	0	0	0	0	0	0.1	0	0	99.6	0.3	0	99.8	0.1	0.1	

7. D PDCD1 Ex.1 SD + Pulse

	C_{-10}	C_{-9}	G_{-8}	C_{-7}	C_{-6}	G_{-5}	A_{-4}	C_{-3}	C_{-2}	C_{-1}	C_{1}	A_{2}	C_{3}	C_{4}	T_{5}	A_{6}	C_{7}	C_{8}	T_{9}	A_{10}	A_{11}	G_{12}	A_{13}	A_{14}	C_{15}	C_{16}	A_{17}	T_{18}	C_{19}	C_{20}	T_{21}	G_{22}	G_{23}	Indel
A	0	0	0	0	0	0	99.6	0	0	0	0	99.2	0	0	0	99.8	0	0	0	99.6	97.7	0	98.8	99.5	0	0	99.6	0	0	0	0	0	0	0.1
C	100	99.9	0	100	99.9	0	0.3	100	99.9	100	100	0.7	100	100	0.3	0.2	99.9	99.9	0.3	0.3	2.2	0.1	1.2	0.4	100	99.9	0.4	0.4	100	100	0.1	0.1	0.4	
G	0	0	99.9	0	0	99.9	0.1	0	0	0	0	0.1	0	0	0	0	0	0.1	0.1	0	0.1	99.8	0.1	0	0	0	0	0.1	0	0	0.1	99.8	99.5	
T	0	0	0	0	0	0	0	0	0	0	0	0	0	0	99.7	0	0.1	0	99.6	0	0	0	0	0	0	0	0	99.5	0	0	99.8	0.1	0.1	

8. A PDCD1 Ex.1 SD + Pulse

	C_{-10}	C_{-9}	G_{-8}	C_{-7}	C_{-6}	G_{-5}	A_{-4}	C_{-3}	C_{-2}	C_{-1}	C_{1}	A_{2}	C_{3}	C_{4}	T_{5}	A_{6}	C_{7}	C_{8}	T_{9}	A_{10}	A_{11}	G_{12}	A_{13}	A_{14}	C_{15}	C_{16}	A_{17}	T_{18}	C_{19}	C_{20}	T_{21}	G_{22}	G_{23}	Indel
A	0	0	0.1	0	0	0	99.8	0	0	0	0	99.2	0	0	0	99.7	0	0	0	99.7	98.1	0	98.8	99.4	0	0	99.7	0	0	0	0	0	0	0
C	100	99.9	0	100	99.9	0	0.2	99.9	99.9	99.9	99.9	0.7	100	100	0.2	0.2	100	100	0.4	0.3	1.8	0.1	1.1	0.6	100	99.9	0.3	0.3	100	100	0.1	0.1	0.3	
G	0	0	99.9	0	0	99.9	0	0	0	0	0	0.1	0	0	0	0	0	0	0.1	0	0.1	99.8	0	0	0	0	0	0.1	0	0	0.1	99.8	99.6	
T	0	0	0	0	0	0	0	0	0	0	0	0	0	0	99.7	0	0	0	99.4	0	0	0	0	0	0	0	0	99.5	0	0	99.8	0.1	0.1	

9. A PDCD1 Ex.2 SA + BE3 unconc. mRNA 1.5 µg

	G_{-10}	C_{-9}	C_{-8}	T_{-7}	G_{-6}	T_{-5}	C_{-4}	T_{-3}	G_{-2}	G_{-1}	G_1	G_2	A_3	G_4	T_5	C_6	T_7	G_8	A_9	G_{10}	A_{11}	G_{12}	A_{13}	T_{14}	G_{15}	G_{16}	A_{17}	G_{18}	A_{19}	G_{20}	A_{21}	G_{22}	G_{23}	Indel
A	0.1	0	0	0.1	0.1	0.1	0.2	0.2	0	0	0	0.1	95.7	0	0.1	2.5	0	0	94.2	0	93.5	0	96	0.1	0	0.1	95	0	85.7	0	94.4	0	0	11.1
C	0	99.9	99.9	0.4	0	0.8	96.7	0.6	0	0.1	0.1	0.1	2.9	0	0.5	64.9	0.4	0.2	3.4	0.1	1.8	0	1.3	0.2	0	0.1	1.6	0.1	2.2	0	1.4	0	0	
G	99.8	0	0	1	99.8	3	0.5	1.8	99.8	99.7	99.7	99.8	1.3	99.9	5.7	18.6	1.8	99.5	2.3	99.8	4.6	99.9	2.4	1.5	99.9	99.7	3.2	99.7	11.9	99.9	4.1	99.8	99.7	
T	0.1	0	0	98.6	0.1	96.2	2.5	97.4	0.2	0.2	0.2	0.1	0.1	0	93.8	14	97.8	0.3	0.1	0.1	0.1	0	0.2	98.2	0.1	0.1	0.2	0.2	0.2	0	0.2	0.2	0.2	

10. B PDCD1 Ex.2 SA + BE3 unconc. mRNA 1.5 µg

	G_{-10}	C_{-9}	C_{-8}	T_{-7}	G_{-6}	T_{-5}	C_{-4}	T_{-3}	G_{-2}	G_{-1}	G_1	G_2	A_3	G_4	T_5	C_6	T_7	G_8	A_9	G_{10}	A_{11}	G_{12}	A_{13}	T_{14}	G_{15}	G_{16}	A_{17}	G_{18}	A_{19}	G_{20}	A_{21}	G_{22}	G_{23}	Indel
A	0	0	0	0	0.1	0.1	0.7	0	0	0.1	0.1	0.2	97.7	0.1	0.2	4.2	0	0.1	98.5	0	96.8	0	98.5	0	0	0.1	98.2	0.1	93.7	0	96.8	0	0	6.1
C	0	99.9	99.9	0.2	0	0.6	94.2	0.3	0	0	0	0	1.2	0	0.3	52.5	0.3	0.1	0.6	0	0.9	0	0.6	0.1	0	0	0.5	0	0.9	0	0.6	0	0	
G	99.9	0.1	0	0.4	99.9	1.1	0.9	0.6	99.8	99.8	99.8	99.7	1	99.8	2.2	21.2	0.9	99.8	0.9	99.9	2.2	99.9	0.8	0.2	99.9	99.9	1.3	99.9	5.2	99.9	2.5	99.9	100	
T	0	0	0.1	99.4	0	98.3	4.1	99.1	0.1	0.1	0	0	0.1	0.1	97.3	22.2	98.8	0.1	0.1	0	0.1	0	0.1	99.7	0	0	0	0	0.2	0	0.1	0	0	

11. C PDCD1 Ex.2 SA + BE3 unconc. mRNA 1.5 µg

	G_{-10}	C_{-9}	C_{-8}	T_{-7}	G_{-6}	T_{-5}	C_{-4}	T_{-3}	G_{-2}	G_{-1}	G_1	G_2	A_3	G_4	T_5	C_6	T_7	G_8	A_9	G_{10}	A_{11}	G_{12}	A_{13}	T_{14}	G_{15}	G_{16}	A_{17}	G_{18}	A_{19}	G_{20}	A_{21}	G_{22}	G_{23}	Indel
A	0.1	0.1	0.1	0	0.1	0	0.6	0	0.1	0.1	0.2	0.2	97.5	0	0.2	3.8	0	0	98.3	0.1	96.7	0	98.5	0.1	0	0	98.1	0	93.6	0	96.6	0	0	6.1
C	0.1	99.8	99.9	0.3	0	0.6	94.8	0.3	0	0	0	0.1	1.3	0	0.4	56.5	0.3	0	0.6	0	0.9	0.1	0.6	0.1	0	0	0.4	0	0.9	0	0.7	0	0	
G	99.9	0.1	0	0.5	99.8	1.4	1	0.7	99.8	99.8	99.7	99.7	1.1	99.9	2.5	19.5	1	99.8	1	99.9	2.3	99.9	0.8	0.4	99.9	99.9	1.4	99.9	5.3	99.9	2.6	100	100	
T	0	0	0	99.2	0.1	98	3.6	98.9	0.1	0.1	0.1	0	0	0	96.9	20.2	98.7	0.1	0.1	0	0.1	0	0.1	99.3	0	0	0.1	0	0.1	0.1	0.1	0	0	

12. A PDCD1 Ex.2 SA + BE4 unconc. mRNA 1.5 µg

	G_{-10}	C_{-9}	C_{-8}	T_{-7}	G_{-6}	T_{-5}	C_{-4}	T_{-3}	G_{-2}	G_{-1}	G_1	G_2	A_3	G_4	T_5	C_6	T_7	G_8	A_9	G_{10}	A_{11}	G_{12}	A_{13}	T_{14}	G_{15}	G_{16}	A_{17}	G_{18}	A_{19}	G_{20}	A_{21}	G_{22}	G_{23}	Indel
A	0.1	0.1	0	0	0	0.2	1	0	0.1	0.1	0.1	0.5	93.7	0	0.1	2.9	0	0.1	93.9	0.2	92.1	0.1	95.7	0.2	0	0.1	94.8	0.1	85.3	0	94.2	0	0	13.8
C	0.1	99.7	99.9	0.4	0	0.8	88.9	0.5	0	0.1	0.1	0.1	4.1	0	0.5	54	0.4	0	3.4	0.1	2.1	0	1.4	0.2	0	0.1	1.7	0	2	0	1.3	0	0	
G	99.7	0.1	0	1.2	99.8	3.2	1.2	1.6	99.8	99.6	99.6	99.3	2.2	99.9	6.2	15.7	1.7	99.6	2.6	99.7	5.6	99.9	2.6	1.8	99.9	99.8	3.4	99.8	12.5	99.9	4.4	99.8	99.8	
T	0.1	0	0	98.4	0.1	95.8	9	97.8	0.1	0.2	0.2	0.1	0.1	0	93.2	27.4	97.9	0.2	0.1	0.1	0.2	0	0.2	97.9	0	0.1	0.1	0.1	0.2	0	0.1	0.1	0.2	

13. B PDCD1 Ex.2 SA + BE4 unconc. mRNA 1.5 µg

	G_{-10}	C_{-9}	C_{-8}	T_{-7}	G_{-6}	T_{-5}	C_{-4}	T_{-3}	G_{-2}	G_{-1}	G_1	G_2	A_3	G_4	T_5	C_6	T_7	G_8	A_9	G_{10}	A_{11}	G_{12}	A_{13}	T_{14}	G_{15}	G_{16}	A_{17}	G_{18}	A_{19}	G_{20}	A_{21}	G_{22}	G_{23}	Indel
A	0	0	0	0	0.1	0.1	1	0	0.1	0.2	0.2	0.5	98.2	0.1	0.1	3.2	0	0.2	98.6	0.1	97.4	0	98.7	0	0	0.1	98.3	0.1	94.1	0	96.9	0	0	6.4
C	0	99.9	99.9	0.3	0	0.6	88.3	0.4	0	0	0	0.1	0.9	0	0.4	53.3	0.2	0	0.6	0	0.8	0	0.5	0.1	0	0	0.5	0	1	0	0.7	0	0	
G	99.9	0	0	0.4	99.8	1.1	1.3	0.7	99.9	99.7	99.6	99.3	0.8	99.9	2	15.1	0.7	99.8	0.7	99.8	1.8	100	0.7	0.2	99.9	99.9	1.2	99.9	4.7	99.9	2.3	99.9	99.9	
T	0	0	0.1	99.3	0	98.2	9.4	98.8	0.1	0.1	0.1	0.1	0	0	97.6	28.4	99	0	0	0	0.1	0	0.1	99.7	0	0	0	0	0.2	0	0.1	0	0	

14. C PDCD1 Ex.2 SA + BE4 unconc. mRNA 1.5 µg

	G_{-10}	C_{-9}	C_{-8}	T_{-7}	G_{-6}	T_{-5}	C_{-4}	T_{-3}	G_{-2}	G_{-1}	G_1	G_2	A_3	G_4	T_5	C_6	T_7	G_8	A_9	G_{10}	A_{11}	G_{12}	A_{13}	T_{14}	G_{15}	G_{16}	A_{17}	G_{18}	A_{19}	G_{20}	A_{21}	G_{22}	G_{23}	Indel
A	0	0.1	0.1	0	0.1	0	1	0.1	0.1	0.2	0.2	0.4	97.4	0.1	0.2	2.9	0	0	98.3	0	96.2	0	98.4	0.1	0	0.1	98.2	0	93.2	0	96.4	0	0	6.2
C	0	99.5	99.8	0.3	0	0.5	88.5	0.3	0	0	0	0.1	1.4	0	0.3	57.4	0.2	0.1	0.7	0	1	0	0.6	0.1	0	0	0.4	0.1	0.8	0	0.7	0	0	
G	99.9	0.3	0	0.5	99.8	1.9	1.3	1.1	99.9	99.8	99.6	99.4	1.1	99.8	2.6	13.1	1	99.8	1	99.9	2.7	100	1	0.4	99.9	99.9	1.4	99.9	5.9	99.9	2.8	99.9	99.9	
T	0	0	0	99.2	0.1	97.5	9.2	98.5	0	0	0.2	0.1	0.1	0	96.8	26.6	98.8	0	0	0	0.1	0	0.1	99.4	0	0	0	0	0.1	0	0.1	0.1	0.1	

15. D PDCD1 Ex.2 SA + Pulse

	G_{-10}	C_{-9}	C_{-8}	T_{-7}	G_{-6}	T_{-5}	C_{-4}	T_{-3}	G_{-2}	G_{-1}	G_1	G_2	A_3	G_4	T_5	C_6	T_7	G_8	A_9	G_{10}	A_{11}	G_{12}	A_{13}	T_{14}	G_{15}	G_{16}	A_{17}	G_{18}	A_{19}	G_{20}	A_{21}	G_{22}	G_{23}	Indel
A	0.1	0	0	0	0.1	0	0	0	0	0.1	0	0.1	98.6	0	0	0	0	0	98.7	0	97.1	0	98.5	0	0	0	98.2	0	94	0	96.7	0	0	0.1
C	0	99.8	99.9	0.3	0	0.5	100	0.3	0	0	0	0	0.7	0	0.5	99.9	0.4	0	0.6	0	0.9	0	0.6	0.1	0	0	0.5	0	0.9	0	0.7	0	0	
G	99.9	0.1	0	0.3	99.9	1	0	0.6	99.9	99.9	100	99.9	0.7	100	1.8	0.1	0.7	100	0.7	100	2	100	0.8	0.3	99.9	100	1.3	99.9	5	99.9	2.5	100	100	
T	0	0.1	0	99.4	0.1	98.5	0	99.1	0	0	0	0	0	0	97.7	0	98.9	0	0	0	0	0	0.1	99.6	0	0	0	0	0.1	0	0.1	0	0	

16. A PDCD1 Ex.2 SA + Pulse

	G_{-10}	C_{-9}	C_{-8}	T_{-7}	G_{-6}	T_{-5}	C_{-4}	T_{-3}	G_{-2}	G_{-1}	G_1	G_2	A_3	G_4	T_5	C_6	T_7	G_8	A_9	G_{10}	A_{11}	G_{12}	A_{13}	T_{14}	G_{15}	G_{16}	A_{17}	G_{18}	A_{19}	G_{20}	A_{21}	G_{22}	G_{23}	Indel
A	1.3	0	0	0	0	0	0	0	0.1	0	0	0	98.6	0	0	0	0	0	98.4	0	97	0	98.2	0	0	0	98.1	0	93.2	0	96.5	0	0	0.1
C	0	99.9	100	0.3	0	0.6	100	0.4	0	0	0	0	0.7	0	0.4	99.9	0.3	0	0.8	0	0.9	0	0.7	0.1	0	0	0.6	0	1.2	0	0.8	0	0	
G	98.7	0	0	0.4	99.9	1.1	0	0.9	99.9	99.9	99.9	99.9	0.6	99.9	2.1	0.1	1	100	0.8	99.9	2	100	1	0.3	99.9	100	1.3	99.9	5.4	100	2.6	99.9	99.9	
T	0	0	0	99.3	0	98.3	0	98.8	0.1	0	0	0	0	0	97.4	0	98.7	0	0.1	0	0.1	0	0.1	99.6	0	0	0	0	0.2	0	0.1	0.1	0	

17. A PDCD1 Ex.3 SA + BE3 unconc. mRNA 1.5 µg

	A_{-10}	C_{-9}	T_{-8}	T_{-7}	C_{-6}	T_{-5}	G_{-4}	C_{-3}	C_{-2}	C_{-1}	T_{1}	T_{2}	C_{3}	T_{4}	C_{5}	T_{6}	C_{7}	T_{8}	G_{9}	G_{10}	A_{11}	A_{12}	G_{13}	G_{14}	G_{15}	C_{16}	A_{17}	C_{18}	A_{19}	A_{20}	A_{21}	G_{22}	G_{23}	Indel
A	88.9	0.7	0.1	0.3	1	0	0	0.5	0.2	0	0	0	0	0.1	3.8	0.1	7.5	0	0	0.1	99.9	100	0	0	0.1	0	99.9	0.3	100	99.5	99.9	0.3	0	23.3
C	5.1	97.7	2	1.7	93	1.2	0.1	98.7	99.6	99.7	0.3	0.1	83.4	0.1	58.9	0.3	63.2	0.3	0	0	0	0	0	0	0	100	0	99.7	0	0	0.1	0	0	
G	5.8	1.1	6	3.4	2.2	2.4	99.7	0.6	0.2	0	0	0	0	0	0.1	0	2.4	0	100	99.9	0.1	0	100	100	99.9	0	0.1	0	0	0.5	0	99.7	100	
T	0.2	0.4	91.9	94.6	3.8	96.4	0.2	0.2	0	0.3	99.7	99.9	16.6	99.7	37.1	99.6	26.9	99.7	0	0	0	0	0	0	0	0	0	0	0	0	0	0	0	

18. B PDCD1 Ex.3 SA + BE3 unconc. mRNA 1.5 µg

	A_{-10}	C_{-9}	T_{-8}	T_{-7}	C_{-6}	T_{-5}	G_{-4}	C_{-3}	C_{-2}	C_{-1}	T_{1}	T_{2}	C_{3}	T_{4}	C_{5}	T_{6}	C_{7}	T_{8}	G_{9}	G_{10}	A_{11}	A_{12}	G_{13}	G_{14}	G_{15}	C_{16}	A_{17}	C_{18}	A_{19}	A_{20}	A_{21}	G_{22}	G_{23}	Indel
A	99.8	0.2	0	0.1	0.9	0	0	0	0	0	0.1	0	0.3	0	2.7	0.2	7.1	0.2	0	0.1	99.9	99.8	0	0	0	0.5	99.8	0.5	99.9	99.9	99.8	0	0	20.1
C	0.1	99.5	0.2	0	94.9	0.2	0	99.9	99.9	99.9	0.3	0.3	83.5	0.4	58.5	0.2	66	0.1	0	0	0	0	0	0	0	99.4	0	99.5	0	0	0	0	0	
G	0	0	0	0	0.1	0	99.9	0	0	0	0	0	0.1	0	0.8	0	2.3	0.1	99.9	99.9	0.1	0.2	100	100	100	0	0.2	0	0	0.1	0.2	100	100	
T	0	0.3	99.8	99.8	4.1	99.8	0	0	0.1	0.1	99.6	99.7	16.2	99.5	37.9	99.6	24.7	99.7	0	0	0	0	0	0	0	0.1	0	0	0	0	0	0	0	

19. C PDCD1 Ex.3 SA + BE3 unconc. mRNA 1.5 µg

	A_{-10}	C_{-9}	T_{-8}	T_{-7}	C_{-6}	T_{-5}	G_{-4}	C_{-3}	C_{-2}	C_{-1}	T_{1}	T_{2}	C_{3}	T_{4}	C_{5}	T_{6}	C_{7}	T_{8}	G_{9}	G_{10}	A_{11}	A_{12}	G_{13}	G_{14}	G_{15}	C_{16}	A_{17}	C_{18}	A_{19}	A_{20}	A_{21}	G_{22}	G_{23}	Indel
A	99.8	0.2	0	0.1	1	0	0.1	0.1	0.1	0	0.1	0	0.4	0	3	0.2	9.6	0.1	0	0	99.8	99.7	0	0	0.1	0.7	99.8	0.4	99.9	99.8	99.8	0	0	23.7
C	0.1	99.4	0.2	0.1	91.9	0.2	0.1	99.8	99.9	99.8	0.3	0.1	78.9	0.4	51.4	0.3	59.1	0.1	0	0	0	0	0	0	0	99.3	0	99.5	0	0	0	0	0	
G	0	0	0.1	0.1	0.1	0.1	99.5	0	0	0	0	0	0.1	0	1.1	0	1.7	0	99.9	100	0.2	0.3	100	100	99.9	0	0.2	0	0.1	0.2	0.2	100	100	
T	0	0.4	99.7	99.7	7	99.7	0.2	0	0	0.1	99.6	99.8	20.6	99.5	44.5	99.5	29.7	99.8	0	0	0	0	0	0	0	0	0	0.1	0	0	0	0	0	

20. A PDCD1 Ex.3 SA + BE4 unconc. mRNA 1.5 µg

	A_{-10}	C_{-9}	T_{-8}	T_{-7}	C_{-6}	T_{-5}	G_{-4}	C_{-3}	C_{-2}	C_{-1}	T_{1}	T_{2}	C_{3}	T_{4}	C_{5}	T_{6}	C_{7}	T_{8}	G_{9}	G_{10}	A_{11}	A_{12}	G_{13}	G_{14}	G_{15}	C_{16}	A_{17}	C_{18}	A_{19}	A_{20}	A_{21}	G_{22}	G_{23}	Indel
A	89	0.6	0.1	0.2	1.4	0.1	0.1	0.3	0.1	0	0.3	0	0.5	0.1	1	0	4.2	0	0.1	0.2	99.9	99.7	0.1	0	0.2	0	99.8	0.1	100	100	100	0	0	22.5
C	5.8	96.9	2.7	1.8	87.9	1.4	0.1	98.8	99.8	99.9	0.2	0.2	71	0.1	52.9	0.2	57.8	0	0	0	0	0	0	0	0	100	0	99.9	0	0	0	0	0	
G	5	1.4	6.7	4.5	1.2	2.2	99.4	0.6	0.1	0	0	0	0	0	0.5	0.1	1.1	0	99.9	99.8	0.1	0.3	99.9	100	99.8	0	0.2	0	0	0	0	100	100	
T	0.2	1.2	90.5	93.5	9.4	96.4	0.5	0.3	0	0.1	99.5	99.8	28.5	99.8	45.6	99.7	36.9	100	0	0	0	0	0	0	0	0	0	0	0	0	0	0	0	

21. B PDCD1 Ex.3 SA + BE4 unconc. mRNA 1.5 µg

	A_{-10}	C_{-9}	T_{-8}	T_{-7}	C_{-6}	T_{-5}	G_{-4}	C_{-3}	C_{-2}	C_{-1}	T_{1}	T_{2}	C_{3}	T_{4}	C_{5}	T_{6}	C_{7}	T_{8}	G_{9}	G_{10}	A_{11}	A_{12}	G_{13}	G_{14}	G_{15}	C_{16}	A_{17}	C_{18}	A_{19}	A_{20}	A_{21}	G_{22}	G_{23}	Indel
A	99.9	0.2	0	0	0.9	0.1	0	0	0	0	0	0	0.2	0	1.7	0.1	4.2	0	0.1	0.1	99.9	99.7	0	0	0.1	0.4	99.8	0.5	100	99.9	99.8	0	0	17.7
C	0	99.1	0.2	0	91.2	0.1	0	100	99.9	99.9	0.3	0.2	78	0.3	63.2	0.2	68.8	0.1	0	0	0	0	0	0	0	99.5	0	99.4	0	0	0	0	0	
G	0.1	0	0	0	0.1	0	100	0	0	0	0	0	0.1	0.1	0.5	0	1.4	0.1	99.9	99.8	0.1	0.2	100	100	99.9	0	0.2	0	0	0.1	0.2	100	100	
T	0	0.7	99.8	99.9	7.9	99.8	0	0	0.1	0.1	99.7	99.8	21.8	99.6	34.6	99.6	25.6	99.8	0	0	0	0	0	0	0	0	0	0	0	0	0	0	0	

22. C PDCD1 Ex.3 SA + BE4 unconc. mRNA 1.5 µg

	A_{-10}	C_{-9}	T_{-8}	T_{-7}	C_{-6}	T_{-5}	G_{-4}	C_{-3}	C_{-2}	C_{-1}	T_{1}	T_{2}	C_{3}	T_{4}	C_{5}	T_{6}	C_{7}	T_{8}	G_{9}	G_{10}	A_{11}	A_{12}	G_{13}	G_{14}	G_{15}	C_{16}	A_{17}	C_{18}	A_{19}	A_{20}	A_{21}	G_{22}	G_{23}	Indel
A	99.8	0.2	0	0.1	2	0.1	0	0	0	0	0	0	0.2	0.1	1.8	0.1	4.2	0	0	0.1	99.9	99.8	0	0.1	0.1	0.7	99.8	0.5	99.9	99.9	99.8	0	0	19.9
C	0.1	98.8	0.2	0.1	89.5	0.2	0	99.9	99.9	99.9	0.2	0.1	73.7	0.2	55.4	0.2	61.6	0.1	0	0	0	0	0	0	0	99.3	0	99.3	0	0	0	0	0	
G	0	0	0	0	0.1	0	99.9	0	0	0	0	0	0.2	0.1	1	0.1	1.5	0.1	100	99.9	0.1	0.2	99.9	99.9	99.9	0	0.2	0	0.1	0.1	0.2	100	100	
T	0	1	99.7	99.8	8.3	99.7	0	0.1	0.1	0.1	99.8	99.8	25.9	99.6	41.9	99.7	32.7	99.8	0	0	0	0	0	0	0	0	0	0.1	0	0	0	0	0	

23. D PDCD1 Ex.3 SA + Pulse

	A_{-10}	C_{-9}	T_{-8}	T_{-7}	C_{-6}	T_{-5}	G_{-4}	C_{-3}	C_{-2}	C_{-1}	T_{1}	T_{2}	C_{3}	T_{4}	C_{5}	T_{6}	C_{7}	T_{8}	G_{9}	G_{10}	A_{11}	A_{12}	G_{13}	G_{14}	G_{15}	C_{16}	A_{17}	C_{18}	A_{19}	A_{20}	A_{21}	G_{22}	G_{23}	Indel
A	99.9	0	0	0	0	0	0	0	0	0	0	0	0	0	0	0	0	0	0	0	99.9	99.8	0	0	0.1	0.5	99.8	0.5	100	99.9	99.8	0	0	0
C	0	100	0.2	0.1	99.9	0.2	0	100	100	100	0.2	0.2	99.9	0.3	100	0.2	99.9	0.2	0	0	0	0	0	0	0	99.4	0	99.5	0	0	0	0	0	
G	0	0	0	0	0	0	100	0	0	0	0	0	0	0.1	0	0	0	0	100	100	0.1	0.2	100	100	99.9	0	0.2	0	0	0.1	0.2	100	100	
T	0	0	99.8	99.9	0.1	99.8	0	0	0	0	99.7	99.8	0	99.7	0	99.7	0	99.8	0	0	0	0	0	0	0	0	0	0	0	0	0	0	0	

24. A PDCD1 Ex.3 SA + Pulse

	A_{-10}	C_{-9}	T_{-8}	T_{-7}	C_{-6}	T_{-5}	G_{-4}	C_{-3}	C_{-2}	C_{-1}	T_{1}	T_{2}	C_{3}	T_{4}	C_{5}	T_{6}	C_{7}	T_{8}	G_{9}	G_{10}	A_{11}	A_{12}	G_{13}	G_{14}	G_{15}	C_{16}	A_{17}	C_{18}	A_{19}	A_{20}	A_{21}	G_{22}	G_{23}	Indel
A	99.9	0	0	0	0	0	0	0	0	0	0	0	0.1	0	0	0	0	0	0.1	0	99.9	99.8	0	0	0.1	0.6	99.8	0.6	100	99.9	99.8	0	0	0
C	0	99.9	0.2	0.1	99.9	0.2	0	100	100	99.9	0.3	0.3	99.8	0.2	99.9	0.3	99.9	0.1	0	0	0	0	0	0	0	99.4	0	99.4	0	0	0	0	0	
G	0.1	0	0	0	0	0.1	100	0	0	0	0	0	0	0.2	0	0.1	0	0	99.9	100	0.1	0.1	100	100	99.9	0	0.1	0	0	0	0.2	100	100	
T	0	0	99.8	99.9	0.1	99.7	0	0	0	0	99.7	99.7	0	99.6	0	99.7	0	99.8	0	0	0	0	0	0	0	0	0	0	0	0	0	0	0	

25. A PDCD1 Ex.3 SD + BE3 unconc. mRNA 1.5 µg

	G_{-10}	G_{-9}	G_{-8}	C_{-7}	T_{-6}	G_{-5}	G_{-4}	G_{-3}	A_{-2}	T_{-1}	G_1	A_2	C_3	G_4	T_5	T_6	A_7	C_8	C_9	T_{10}	C_{11}	G_{12}	T_{13}	G_{14}	C_{15}	G_{16}	G_{17}	C_{18}	C_{19}	C_{20}	G_{21}	G_{22}	G_{23}	Indel
A	0	0	0.1	0	0	0.1	0	0.3	99.5	0	0	97.5	0	0	0	0.1	97.9	1.5	0	0	0	0.1	0	0.1	0	0.1	0.1	0	0	0	0.1	0	0.2	0.1
C	0	0	0.1	99.7	0.3	0	0	0	0.1	0	0	1.9	99.3	0	0	0	2	94.6	99.1	0.7	97.5	0	0.1	0.3	99.5	0	0	100	100	99.7	0	0	0	
G	100	100	99.8	0.2	2.6	99.9	100	99.7	0.3	1.7	100	0.5	0.4	100	0.3	0.1	0.1	0.5	0.2	0.8	2.2	99.9	4.3	99.1	0.4	99.9	99.9	0	0	0.3	99.9	100	99.8	
T	0	0	0	0	97.1	0	0	0	0.1	98.3	0	0	0.3	0	99.7	99.7	0	3.4	0.7	98.5	0.2	0	95.6	0.5	0	0	0	0	0	0	0	0	0	

26. B PDCD1 Ex.3 SD + BE3 unconc. mRNA 1.5 µg

	G_{-10}	G_{-9}	G_{-8}	C_{-7}	T_{-6}	G_{-5}	G_{-4}	G_{-3}	A_{-2}	T_{-1}	G_1	A_2	C_3	G_4	T_5	T_6	A_7	C_8	C_9	T_{10}	C_{11}	G_{12}	T_{13}	G_{14}	C_{15}	G_{16}	G_{17}	C_{18}	C_{19}	C_{20}	G_{21}	G_{22}	G_{23}	Indel
A	0	0	0	0.2	0	0	0	0.1	99.4	0	0	95.6	0.1	0	0	0.1	96.6	2.4	0.2	0	0.2	0	0	0	0.1	0	0	0.1	0	0.2	0.1	0	0.1	0.4
C	0	0	0	98.6	0.1	0	0	0	0.2	0	0	3.3	98.7	0	0.1	0.1	3.1	90	97.6	1.1	96.1	0	0.1	0.1	98.8	0	0	99.9	99.9	98.7	0	0	0	
G	100	100	99.9	1	6.3	100	100	99.8	0.3	2.3	99.9	1	0.7	99.9	0.2	0.4	0.3	1.4	0.4	2.2	3	99.9	5.9	99.5	1.1	99.9	100	0	0	1.1	99.9	100	99.9	
T	0	0	0	0.1	93.5	0	0	0	0.1	97.6	0	0	0.5	0.1	99.7	99.5	0	6.2	1.7	96.7	0.8	0	94.1	0.4	0.1	0	0	0	0	0	0	0	0	

27. C PDCD1 Ex.3 SD + BE3 unconc. mRNA 1.5 µg

	G_{-10}	G_{-9}	G_{-8}	C_{-7}	T_{-6}	G_{-5}	G_{-4}	G_{-3}	A_{-2}	T_{-1}	G_1	A_2	C_3	G_4	T_5	T_6	A_7	C_8	C_9	T_{10}	C_{11}	G_{12}	T_{13}	G_{14}	C_{15}	G_{16}	G_{17}	C_{18}	C_{19}	C_{20}	G_{21}	G_{22}	G_{23}	Indel
A	0	0	0	0.1	0	0	0	0.1	99.4	0	0	95.8	0	0	0	0	96.7	2.2	0.4	0	0.2	0	0	0	0.1	0	0	0	0	0.3	0.1	0	0.1	0.4
C	0	0	0	98.9	0.1	0	0	0	0.2	0.2	0	3.1	98.7	0	0.2	0.1	3.1	90.4	97.9	1.1	96.2	0	0	0.1	98.8	0	0	99.8	100	98.6	0	0	0	
G	100	100	99.9	0.8	6.3	100	99.9	99.9	0.4	2.2	99.9	1	0.7	99.9	0.3	0.2	0.2	0.9	0.4	2.3	3	100	5.6	99.5	1.1	100	100	0.1	0	1.1	99.9	100	99.9	
T	0	0	0	0.1	93.5	0	0	0	0.1	97.6	0	0	0.6	0.1	99.5	99.6	0	6.4	1.4	96.7	0.6	0	94.3	0.4	0	0	0	0.1	0	0	0	0	0	

28. A PDCD1 Ex.3 SD + BE4 unconc. mRNA 1.5 µg

	G_{-10}	G_{-9}	G_{-8}	C_{-7}	T_{-6}	G_{-5}	G_{-4}	G_{-3}	A_{-2}	T_{-1}	G_1	A_2	C_3	G_4	T_5	T_6	A_7	C_8	C_9	T_{10}	C_{11}	G_{12}	T_{13}	G_{14}	C_{15}	G_{16}	G_{17}	C_{18}	C_{19}	C_{20}	G_{21}	G_{22}	G_{23}	Indel
A	0	0	0.2	0.1	0	0.1	0.1	0.1	99.6	0	0	97.8	0	0	0	0	98.3	2.4	0.8	0	0.1	0	0	0.1	0	0	0	0	0	0.1	0.1	0.1	0	0.3
C	0	0	0	99.4	0	0	0	0	0.2	0	0.1	1.8	98.8	0	0.3	0.1	1.6	90	93.1	1	96.1	0	0.1	0	99.5	0	0	99.9	99.8	99.2	0	0	0	
G	100	100	99.8	0.3	3.3	99.9	99.9	99.9	0.1	1.8	99.8	0.4	0.5	99.9	0.2	0.1	0	0.6	1.8	1	2.5	100	4.3	99.5	0.5	100	100	0	0	0.6	99.9	99.9	100	
T	0	0	0	0.1	96.7	0	0	0	0	98.2	0.1	0	0.6	0.1	99.5	99.7	0	7.1	4.3	97.9	1.3	0	95.6	0.4	0	0	0	0	0.2	0.2	0	0	0	

29. B PDCD1 Ex.3 SD + BE4 unconc. mRNA 1.5 µg

	G_{-10}	G_{-9}	G_{-8}	C_{-7}	T_{-6}	G_{-5}	G_{-4}	G_{-3}	A_{-2}	T_{-1}	G_1	A_2	C_3	G_4	T_5	T_6	A_7	C_8	C_9	T_{10}	C_{11}	G_{12}	T_{13}	G_{14}	C_{15}	G_{16}	G_{17}	C_{18}	C_{19}	C_{20}	G_{21}	G_{22}	G_{23}	Indel
A	0	0	0.1	0.2	0	0	0.1	0.2	99.5	0	0.1	95.6	0.1	0	0	0.1	96.7	1.9	0.4	0	0.4	0	0	0	0	0	0	0	0	0.2	0.1	0	0	0.6
C	0	0	0	98.7	0.2	0	0	0	0.2	0.1	0	3.4	98.7	0	0.1	0.1	3	87.3	92.2	1.2	94.7	0	0.1	0.1	99	0	0	99.9	99.9	98.6	0	0	0	
G	100	100	99.9	0.9	6	99.9	99.9	99.8	0.3	2.5	99.8	1	0.6	99.8	0.2	0.3	0.2	1	1.4	2.5	2.9	99.9	6.2	99.5	1	100	100	0.1	0	1.1	99.9	100	99.9	
T	0	0	0	0.1	93.8	0	0	0	0	97.4	0.1	0	0.6	0.2	99.7	99.5	0	9.8	6	96.3	2	0	93.7	0.4	0	0	0	0	0	0	0	0	0	

30. C PDCD1 Ex.3 SD + BE4 unconc. mRNA 1.5 µg

	G_{-10}	G_{-9}	G_{-8}	C_{-7}	T_{-6}	G_{-5}	G_{-4}	G_{-3}	A_{-2}	T_{-1}	G_1	A_2	C_3	G_4	T_5	T_6	A_7	C_8	C_9	T_{10}	C_{11}	G_{12}	T_{13}	G_{14}	C_{15}	G_{16}	G_{17}	C_{18}	C_{19}	C_{20}	G_{21}	G_{22}	G_{23}	Indel
A	0	0	0	0.2	0	0	0	0.2	99.5	0	0	96.1	0.1	0.1	0	0.1	96.6	2.4	0.6	0	0.4	0.1	0	0	0.1	0	0	0.1	0	0.2	0	0	0.1	0.3
C	0	0	0	98.7	0.1	0	0	0	0.2	0.1	0	3	98.6	0	0.1	0.1	3.1	87.2	92.8	1	94	0	0.1	0.1	98.8	0	0	99.8	99.9	98.7	0	0	0	
G	100	100	99.9	1	6.1	100	99.9	99.8	0.2	2.5	99.9	0.9	0.7	99.8	0.2	0.3	0.3	1.2	1.1	2.3	2.9	99.9	6.2	99.6	1	100	100	0.1	0	1.1	99.9	100	99.9	
T	0	0	0	0.1	93.7	0	0	0	0	97.4	0	0	0.6	0.1	99.7	99.4	0	9.3	5.5	96.7	2.7	0	93.7	0.3	0.1	0	0	0	0	0	0	0	0	

31. A PDCD1 Ex.4 SA + BE3 unconc. mRNA 1.5 µg

	T_{-10}	C_{-9}	C_{-8}	T_{-7}	A_{-6}	T_{-5}	T_{-4}	G_{-3}	T_{-2}	C_{-1}	C_1	C_2	T_3	G_4	C_5	A_6	G_7	A_8	G_9	A_{10}	A_{11}	A_{12}	C_{13}	A_{14}	C_{15}	A_{16}	C_{17}	T_{18}	T_{19}	G_{20}	G_{21}	G_{22}	G_{23}	Indel
A	0	0	0	0.1	100	0	0	0.1	0.1	0.6	0.2	0	0.1	0.1	1.6	99.4	0	97.2	0.1	99.7	99.3	98.4	0	99.4	0	99.1	0	0	0	0.1	0	0.1	0	14.3
C	0.2	99.8	100	0.3	0	0	0	0	0.1	95.9	99.5	99.6	0.2	0	83	0.5	0.1	1.7	0.1	0.2	0.6	1.3	99.7	0.5	99.7	0.5	99.2	0.1	0.1	0.1	0	0	0	
G	0.1	0	0	0.1	0	0	0	99.8	0	0.1	0	0	0.1	99.9	1.7	0	99.9	1.1	99.8	0.1	0.1	0.3	0.1	0.1	0.1	0.4	0.5	2	2.5	99.7	100	99.9	99.8	
T	99.7	0.1	0	99.6	0	100	100	0.2	99.8	3.4	0.3	0.4	99.7	0	13.7	0	0	0	0	0	0	0	0.2	0	0.2	0	0.2	97.9	97.3	0.1	0	0	0.2	

32. B PDCD1 Ex.4 SA + BE3 unconc. mRNA 1.5 µg

	T_{-10}	C_{-9}	C_{-8}	T_{-7}	A_{-6}	T_{-5}	T_{-4}	G_{-3}	T_{-2}	C_{-1}	C_1	C_2	T_3	G_4	C_5	A_6	G_7	A_8	G_9	A_{10}	A_{11}	A_{12}	C_{13}	A_{14}	C_{15}	A_{16}	C_{17}	T_{18}	T_{19}	G_{20}	G_{21}	G_{22}	G_{23}	Indel
A	0	0.1	0	0.1	100	0	0	0	0	1	0.3	0.2	0	0.1	2.1	99.7	0	99	0	99.7	99.4	97.9	0	99.5	0	99.7	0	0	0	0.2	0	0	0	8.3
C	0.4	99.7	100	0.1	0	0.1	0.1	0	0.1	95.3	99.3	98.8	0.1	0.2	80.2	0.1	0	0.8	0	0.2	0.4	1.9	99.8	0.3	99.8	0.1	99.8	0.2	0.2	0	0	0	0	
G	0.2	0	0	0	0	0	0	99.9	0	0.3	0	0.2	0	99.7	1.5	0.2	99.9	0.1	100	0.1	0.1	0.2	0	0.2	0	0.2	0.1	0.6	0.1	99.8	99.8	99.9	99.9	
T	99.4	0.2	0	99.8	0	99.9	99.9	0.1	99.8	3.4	0.3	0.8	99.8	0.1	16.2	0	0	0	0	0	0	0	0.2	0	0.2	0	0.1	99.2	99.7	0	0.1	0.1	0	

33. C PDCD1 Ex.4 SA + BE3 unconc. mRNA 1.5 µg

	T_{-10}	C_{-9}	C_{-8}	T_{-7}	A_{-6}	T_{-5}	T_{-4}	G_{-3}	T_{-2}	C_{-1}	C_{1}	C_{2}	T_{3}	G_{4}	C_{5}	A_{6}	G_{7}	A_{8}	G_{9}	A_{10}	A_{11}	A_{12}	C_{13}	A_{14}	C_{15}	A_{16}	C_{17}	T_{18}	T_{19}	G_{20}	G_{21}	G_{22}	G_{23}	Indel
A	0	0.1	0	0	100	0	0	0.1	0	0.8	0.1	0.1	0	0.1	2	99.7	0.1	98.9	0	99.7	99.4	97.5	0.1	99.5	0	99.5	0.1	0	0	0	0	0.1	0	7.1
C	0.5	99.7	100	0.1	0	0.1	0	0.1	0.2	95.8	99.4	99.4	0.1	0	82.1	0.2	0	1	0	0.2	0.5	2.2	99.6	0.3	99.5	0.1	99.7	0.4	0.2	0	0	0	0	
G	0.1	0	0	0.1	0	0	0.1	99.7	0	0.5	0	0	0	99.9	1.7	0.1	99.9	0.1	99.9	0.1	0.1	0.3	0	0.2	0.1	0.2	0.2	0.8	0.1	99.9	99.9	99.9	99.9	
T	99.4	0.1	0	99.8	0	99.9	99.8	0.1	99.8	2.9	0.5	0.5	99.8	0	14.2	0	0	0	0	0	0	0	0.4	0	0.4	0.2	0	98.8	99.7	0.1	0.1	0	0	

34. A PDCD1 Ex.4 SA + BE4 unconc. mRNA 1.5 µg

	T_{-10}	C_{-9}	C_{-8}	T_{-7}	A_{-6}	T_{-5}	T_{-4}	G_{-3}	T_{-2}	C_{-1}	C_{1}	C_{2}	T_{3}	G_{4}	C_{5}	A_{6}	G_{7}	A_{8}	G_{9}	A_{10}	A_{11}	A_{12}	C_{13}	A_{14}	C_{15}	A_{16}	C_{17}	T_{18}	T_{19}	G_{20}	G_{21}	G_{22}	G_{23}	Indel
A	0	0	0	0	100	0	0	0	0	7.1	0	0	0	0	0	100	0	98.7	0	100	98.7	98.7	0	100	0	97.4	0	0	0	0	0	0	0	2.8
C	0	100	100	0	0	0	0	0	0	92.9	100	100	0	0	88.4	0	0	0	0	0	0	1.4	100	0	100	1.3	97.4	0	0	0	0	0	0	
G	0	0	0	0	0	0	0	100	0	0	0	0	0	100	0	0	100	1.4	100	0	1.3	0	0	0	0	1.3	2.6	4	1.3	100	100	100	100	
T	100	0	0	100	0	100	100	0	100	0	0	0	100	0	11.6	0	0	0	0	0	0	0	0	0	0	0	0	96	98.7	0	0	0	0	

35. B PDCD1 Ex.4 SA + BE4 unconc. mRNA 1.5 µg

	T_{-10}	C_{-9}	C_{-8}	T_{-7}	A_{-6}	T_{-5}	T_{-4}	G_{-3}	T_{-2}	C_{-1}	C_{1}	C_{2}	T_{3}	G_{4}	C_{5}	A_{6}	G_{7}	A_{8}	G_{9}	A_{10}	A_{11}	A_{12}	C_{13}	A_{14}	C_{15}	A_{16}	C_{17}	T_{18}	T_{19}	G_{20}	G_{21}	G_{22}	G_{23}	Indel
A	0	0.2	0	0	100	0	0	0.1	0.1	0.9	0.2	0.2	0	0.1	1.3	99.8	0	99.2	0	99.8	99.5	97.8	0	99.6	0	99.8	0.1	0	0.1	0	0	0.1	0	6.2
C	0.4	99.2	99.9	0.1	0	0	0	0	0.1	93.2	98.9	98.3	0.1	0	80.5	0.1	0	0.6	0	0.1	0.4	2	99.8	0.3	99.7	0.1	99.8	0.3	0.2	0	0	0	0	
G	0.1	0.1	0	0	0	0	0	99.9	0	0.4	0	0.1	0	99.8	1.3	0.1	99.9	0.1	100	0	0.1	0.2	0	0.1	0	0.1	0	0.6	0.1	99.9	99.9	99.9	99.9	
T	99.5	0.6	0.1	99.8	0	99.9	99.9	0.1	99.8	5.5	0.9	1.4	99.8	0	16.9	0	0	0	0	0	0	0	0.1	0	0.3	0	0.1	99.1	99.6	0.1	0	0	0	

36. C PDCD1 Ex.4 SA + BE4 unconc. mRNA 1.5 µg

	T_{-10}	C_{-9}	C_{-8}	T_{-7}	A_{-6}	T_{-5}	T_{-4}	G_{-3}	T_{-2}	C_{-1}	C_{1}	C_{2}	T_{3}	G_{4}	C_{5}	A_{6}	G_{7}	A_{8}	G_{9}	A_{10}	A_{11}	A_{12}	C_{13}	A_{14}	C_{15}	A_{16}	C_{17}	T_{18}	T_{19}	G_{20}	G_{21}	G_{22}	G_{23}	Indel
A	0	0	0	0.1	100	0	0	0	0.1	1.1	0.3	0.1	0	0.1	1.6	99.8	0.2	99	0.1	99.6	99.5	97.9	0	99.5	0	99.8	0.1	0	0	0	0	0	0	8.8
C	0.4	99.3	99.9	0.2	0	0.1	0	0	0.1	92.3	99	98.6	0.1	0	75.4	0.2	0.1	0.7	0	0.2	0.4	1.8	99.4	0.3	99.8	0.1	99.7	0.3	0.2	0	0	0	0	
G	0.2	0	0	0.1	0	0.1	0	99.8	0	0.1	0	0	0	99.9	1.3	0.1	99.7	0.1	99.8	0.1	0.1	0.2	0.2	0.2	0	0.2	0.1	0.6	0.1	99.9	99.9	99.9	100	
T	99.4	0.7	0.1	99.7	0	99.8	99.9	0.1	99.8	6.4	0.7	1.3	99.8	0	21.7	0	0	0.1	0.1	0.1	0	0.1	0.4	0	0.2	0	0.2	99.1	99.7	0.1	0	0	0	

37. D PDCD1 Ex.4 SA + Pulse

	T_{-10}	C_{-9}	C_{-8}	T_{-7}	A_{-6}	T_{-5}	T_{-4}	G_{-3}	T_{-2}	C_{-1}	C_{1}	C_{2}	T_{3}	G_{4}	C_{5}	A_{6}	G_{7}	A_{8}	G_{9}	A_{10}	A_{11}	A_{12}	C_{13}	A_{14}	C_{15}	A_{16}	C_{17}	T_{18}	T_{19}	G_{20}	G_{21}	G_{22}	G_{23}	Indel
A	0	0	0	0	100	0	0	0.1	0	0	0	0	0	0	0	99.8	0	99.1	0	99.8	99.4	97.5	0	99.4	0	99.8	0	0	0	0.1	0	0	0	0
C	0.4	99.9	100	0.1	0	0.2	0.1	0	0.1	100	99.9	100	0.1	0	99.9	0.1	0	0.7	0	0.2	0.4	2.2	100	0.4	100	0.1	100	0.3	0.2	0	0	0	0	
G	0.1	0	0	0	0	0	0	99.8	0	0	0	0	0	100	0	0.1	100	0.1	99.9	0.1	0.1	0.3	0	0.2	0	0.1	0	0.7	0.1	99.8	99.9	100	99.9	
T	99.5	0.1	0	99.8	0	99.8	99.9	0.1	99.9	0	0	0	99.9	0	0.1	0	0	0	0	0	0	0	0	0	0	0	0	99	99.6	0.1	0.1	0	0	

38. A PDCD1 Ex.4 SA + Pulse

	T_{-10}	C_{-9}	C_{-8}	T_{-7}	A_{-6}	T_{-5}	T_{-4}	G_{-3}	T_{-2}	C_{-1}	C_{1}	C_{2}	T_{3}	G_{4}	C_{5}	A_{6}	G_{7}	A_{8}	G_{9}	A_{10}	A_{11}	A_{12}	C_{13}	A_{14}	C_{15}	A_{16}	C_{17}	T_{18}	T_{19}	G_{20}	G_{21}	G_{22}	G_{23}	Indel
A	0	0	0	0	100	0	0	0	0	0	0	0	0.1	0.1	0	99.8	0	99.2	0	99.9	99.6	97.6	0	99.7	0	99.8	0.1	0	0	0	0	0	0	0
C	0.4	100	100	0.1	0	0.1	0.1	0	0.2	100	99.9	100	0.1	0	100	0.1	0	0.6	0	0.1	0.3	2.2	100	0.2	100	0.1	99.9	0.2	0.4	0	0	0	0	
G	0.2	0	0	0.1	0	0	0	99.9	0	0	0	0	0	99.9	0	0.1	100	0.1	100	0.1	0.1	0.2	0	0.1	0	0.1	0	0.6	0.2	99.9	99.9	99.9	100	
T	99.5	0	0	99.8	0	99.9	99.9	0	99.8	0	0	0	99.8	0	0	0	0	0	0	0	0	0	0	0	0	0	0	99.1	99.4	0	0	0	0	

39. A PDCD1 Ex.2 pmSTOP + BE3 unconc. mRNA 1.5 µg

	A_{-10}	G_{-9}	A_{-8}	A_{-7}	G_{-6}	G_{-5}	T_{-4}	G_{-3}	G_{-2}	G_{-1}	G_{1}	G_{2}	G_{3}	G_{4}	T_{5}	T_{6}	C_{7}	C_{8}	A_{9}	G_{10}	G_{11}	G_{12}	C_{13}	C_{14}	T_{15}	G_{16}	T_{17}	C_{18}	T_{19}	G_{20}	G_{21}	G_{22}	G_{23}	Indel
A	99.3	0.4	99.3	99.4	0	0.1	0	0.1	0.1	0.1	0.1	0.1	0.1	0.1	0.1	0.1	11.4	1.9	99.3	0	0	0	0	0	0	0.1	0	0.3	0	0	0	0	0	11.3
C	0.4	0	0.3	0.2	0	0	0.2	0	0	0	0	0	0	0	0.4	0.2	54.8	81.2	0.5	0	0	0	99.9	99.9	0.3	0	0.6	98.2	0.5	0	0.1	0.1	0	
G	0.2	99.5	0.4	0.3	99.9	99.7	2.2	99.6	99.9	99.9	99.9	99.8	99.9	99.2	1.2	0.7	3.2	0.4	0.2	99.9	99.9	99.9	0	0	0.6	99.8	2.3	0.3	1.3	99.8	99.7	99.7	99.8	
T	0	0	0	0.1	0	0.1	97.5	0.3	0.1	0.1	0.1	0.1	0	0.7	98.3	99	30.5	16.4	0	0.1	0.1	0	0	0	99.1	0.1	97.1	1.2	98.2	0.1	0.2	0.2	0.1	

40. B PDCD1 Ex.2 pmSTOP + BE3 unconc. mRNA 1.5 µg

	A_{-10}	G_{-9}	A_{-8}	A_{-7}	G_{-6}	G_{-5}	T_{-4}	G_{-3}	G_{-2}	G_{-1}	G_{1}	G_{2}	G_{3}	G_{4}	T_{5}	T_{6}	C_{7}	C_{8}	A_{9}	G_{10}	G_{11}	G_{12}	C_{13}	C_{14}	T_{15}	G_{16}	T_{17}	C_{18}	T_{19}	G_{20}	G_{21}	G_{22}	G_{23}	Indel
A	98.6	0.2	98.6	99.5	0	0.1	0.1	0	0.1	0.1	0	0.1	0	0.4	0	0	12.2	2.5	99.7	0	0	0	0.1	0	0	0	0	0.2	0	0	0	0	0.1	5.4
C	0.2	0	0.3	0.1	0	0	0.2	0	0	0	0	0	0	0	0.3	0.2	49.2	77.1	0.2	0	0	0	99.6	99.8	0.2	0	0.4	97.7	0.2	0	0	0	0	
G	1.2	99.8	1.1	0.4	99.8	99.8	2	99.8	99.8	99.8	100	99.9	100	99	0.6	0.7	3.7	0.5	0.2	99.9	100	99.9	0.2	0.1	0.5	99.9	1.4	0.5	1	99.9	99.9	99.9	99.9	
T	0	0	0	0	0.2	0.1	97.8	0.1	0.1	0	0	0	0	0.6	99.1	99.1	34.8	19.9	0	0	0	0	0	0.1	99.4	0.1	98.2	1.6	98.8	0	0	0	0	

41. C PDCD1 Ex.2 pmSTOP + BE3 unconc. mRNA 1.5 µg

	A_{-10}	G_{-9}	A_{-8}	A_{-7}	G_{-6}	G_{-5}	T_{-4}	G_{-3}	G_{-2}	G_{-1}	G_1	G_2	G_3	G_4	T_5	T_6	C_7	C_8	A_9	G_{10}	G_{11}	G_{12}	C_{13}	C_{14}	T_{15}	G_{16}	T_{17}	C_{18}	T_{19}	G_{20}	G_{21}	G_{22}	G_{23}	Indel
A	99.5	0.1	99.4	99.8	0	0	0	0	0.1	0	0	0	0	0	0	0.2	4.1	0.6	99.7	0	0	0	0	0	0	0	0	0.1	0	0	0.1	0	0	1.7
C	0.1	0	0.2	0.1	0	0	0.1	0	0	0	0	0	0	0	0.3	0.1	76.7	88.7	0.2	0	0	0.1	99.8	99.9	0.2	0	0.4	99.5	0.3	0	0	0	0	
G	0.3	99.8	0.4	0.2	99.9	99.9	1	99.9	99.9	100	99.9	99.9	100	99.8	0.2	0.2	1.2	0.1	0.1	100	99.9	54.7	0	0	0.3	99.9	0.6	0.1	0.4	99.9	99.9	99.9	99.9	
T	0	0	0	0	0	0.1	98.8	0.1	0	0	0.1	0	0	0.2	99.5	99.5	17.9	10.7	0	0	0	45.2	0.1	0	99.5	0	98.9	0.3	99.3	0	0	0	0	

42. A PDCD1 Ex.2 pmSTOP + BE4 unconc. mRNA 1.5 µg

	A_{-10}	G_{-9}	A_{-8}	A_{-7}	G_{-6}	G_{-5}	T_{-4}	G_{-3}	G_{-2}	G_{-1}	G_1	G_2	G_3	G_4	T_5	T_6	C_7	C_8	A_9	G_{10}	G_{11}	G_{12}	C_{13}	C_{14}	T_{15}	G_{16}	T_{17}	C_{18}	T_{19}	G_{20}	G_{21}	G_{22}	G_{23}	Indel
A	99.3	1.7	99.2	99.5	0.1	0.2	0	0	0.1	0	0	0	0	0.1	0	0	5.4	1.9	99.3	0	0	0.1	0	0	0	0	0	0.2	0	0	0	0	0.1	8.3
C	0.4	0	0.3	0.3	0	0	0.2	0	0	0	0	0	0	0	0.5	0.2	41.7	57.8	0.4	0	0	0	99.9	99.9	0.2	0	0.6	98.4	0.5	0	0	0.1	0	
G	0.3	98.3	0.4	0.2	99.8	99.6	2.4	99.7	99.9	99.9	99.9	99.9	99.9	99.2	1.9	1.1	2.7	0.4	0.2	99.9	99.9	99.8	0	0	0.8	99.9	2.6	0.1	1.5	99.8	99.8	99.7	99.8	
T	0	0	0	0	0	0.2	97.4	0.2	0	0	0	0.1	0	0.7	97.6	98.6	50.2	40	0	0.1	0.1	0.1	0	0	98.9	0.1	96.7	1.3	98	0.1	0.2	0.2	0.1	

43. B PDCD1 Ex.2 pmSTOP + BE4 unconc. mRNA 1.5 µg

	A_{-10}	G_{-9}	A_{-8}	A_{-7}	G_{-6}	G_{-5}	T_{-4}	G_{-3}	G_{-2}	G_{-1}	G_1	G_2	G_3	G_4	T_5	T_6	C_7	C_8	A_9	G_{10}	G_{11}	G_{12}	C_{13}	C_{14}	T_{15}	G_{16}	T_{17}	C_{18}	T_{19}	G_{20}	G_{21}	G_{22}	G_{23}	Indel
A	99.1	1	99.2	99.7	0.1	0.2	0	0	0	0	0	0	0	0.1	0	0.1	8.3	2.5	99.7	0	0	0.1	0.1	0	0	0.2	0	0.2	0	0	0	0.1	0.1	4
C	0.1	0.1	0.2	0	0	0	0.2	0	0	0	0	0	0	0	0.2	0.1	48.9	68.9	0.1	0	0	0	99.8	100	0.2	0	0.4	97.8	0.3	0	0	0	0	
G	0.8	98.9	0.6	0.2	99.9	99.7	1.6	99.9	99.9	99.9	100	99.9	99.9	99.2	0.4	0.4	2.9	0.4	0.1	100	99.9	99.9	0.2	0	0.2	99.8	1	0.2	0.6	100	99.9	99.8	99.8	
T	0	0	0	0	0	0.1	98.3	0.1	0.1	0	0	0	0	0.7	99.3	99.4	39.8	28.2	0	0	0	0	0	0	99.6	0	98.6	1.8	99.2	0	0.1	0	0	

44. C PDCD1 Ex.2 pmSTOP + BE4 unconc. mRNA 1.5 µg

	A_{-10}	G_{-9}	A_{-8}	A_{-7}	G_{-6}	G_{-5}	T_{-4}	G_{-3}	G_{-2}	G_{-1}	G_1	G_2	G_3	G_4	T_5	T_6	C_7	C_8	A_9	G_{10}	G_{11}	G_{12}	C_{13}	C_{14}	T_{15}	G_{16}	T_{17}	C_{18}	T_{19}	G_{20}	G_{21}	G_{22}	G_{23}	Indel
A	99.6	0.2	99.4	99.8	0	0	0	0	0	0.1	0	0	0	0	0	0	2.3	0.7	99.8	0	0	0	0.1	0	0	0	0	0	0	0	0	0	0	1.9
C	0.1	0	0.2	0.1	0	0	0.1	0	0	0	0	0	0	0	0.2	0.1	70.3	78.6	0.1	0	0	0.1	99.7	99.9	0.2	0	0.4	99.5	0.2	0	0	0	0	
G	0.3	99.7	0.4	0.2	100	99.9	1.1	99.8	100	99.9	100	100	99.9	99.7	0.3	0.3	0.9	0.2	0.1	100	99.9	54.1	0	0	0.2	99.9	0.6	0.1	0.5	99.9	100	100	100	
T	0	0	0	0	0	0.1	98.7	0.2	0	0	0	0	0	0.3	99.5	99.5	26.4	20.5	0	0	0	45.8	0.1	0.1	99.6	0	98.9	0.4	99.3	0	0	0	0	

45. A PDCD1 Ex.2 pmSTOP + Cas9 mRNA 1.5 µg

	A_{-10}	G_{-9}	A_{-8}	A_{-7}	G_{-6}	G_{-5}	T_{-4}	G_{-3}	G_{-2}	G_{-1}	G_1	G_2	G_3	G_4	T_5	T_6	C_7	C_8	A_9	G_{10}	G_{11}	G_{12}	C_{13}	C_{14}	T_{15}	G_{16}	T_{17}	C_{18}	T_{19}	G_{20}	G_{21}	G_{22}	G_{23}	Indel
A	99	0	99	99.6	0.3	0.1	0.2	0	0.1	0.1	0	0.2	0	0.2	0	0	0.1	0.2	99.4	0	1.2	0.1	0.2	0.1	0.2	0.1	0.3	0.2	0.3	0.2	0.2	0.5	0.1	62.5
C	0.1	0	0.2	0.1	0	0	0.1	0	0.2	0	0	0	0	0	0.3	0.1	95.6	95.3	0.2	0	0	0.5	97.7	99.4	0.5	2.7	0.9	98.5	0.3	0.2	0.1	0.1	0.2	
G	0.9	99.9	0.8	0.4	99.7	99.8	1.6	99.8	99.8	99.9	99.9	99.8	99.9	99.7	0.3	0.3	0.2	0.1	0.3	99.8	98.6	99.3	0.3	0.3	0.6	96.3	1.3	0.5	0.7	99.4	99.2	99.3	99.6	
T	0	0	0	0	0	0	98.1	0.1	0	0	0	0	0.1	0.1	99.4	99.5	4.2	4.4	0.1	0.1	0.2	0.2	1.8	0.2	98.7	0.9	97.6	0.9	98.7	0.2	0.4	0.1	0.1	

46. B PDCD1 Ex.2 pmSTOP + Cas9 mRNA 1.5 µg

	A_{-10}	G_{-9}	A_{-8}	A_{-7}	G_{-6}	G_{-5}	T_{-4}	G_{-3}	G_{-2}	G_{-1}	G_1	G_2	G_3	G_4	T_5	T_6	C_7	C_8	A_9	G_{10}	G_{11}	G_{12}	C_{13}	C_{14}	T_{15}	G_{16}	T_{17}	C_{18}	T_{19}	G_{20}	G_{21}	G_{22}	G_{23}	Indel
A	99.5	0	99.4	99.7	0	0	0.1	0.1	0	0	0.1	0.2	0.1	0.2	0	0	0.4	0.1	99.2	0.4	0.2	0.2	0.3	0.1	0.4	0.1	0.4	0.1	0.1	0.2	0.2	0.2	0.2	47
C	0.1	0	0.2	0.1	0	0	0.1	0	0	0	0	0	0	0.2	0.4	0.2	93.6	93.9	0.2	0	0.1	0.3	98.8	99.6	0.2	0.4	0.4	99.4	0.4	0.3	0.2	0.2	0.1	
G	0.4	100	0.3	0.2	100	99.9	1	99.9	99.9	99.9	99.9	99.7	99.8	99.4	0.3	0.3	0.2	0.4	0.5	99.5	99.7	44.4	0.2	0	0.3	99.4	1.5	0.4	0.5	99.4	99	99.6	99.7	
T	0	0	0	0	0	0	98.8	0	0	0.1	0	0	0.1	0.2	99.2	99.4	5.7	5.6	0.1	0.1	0	55.1	0.7	0.2	99	0.1	97.7	0.1	99	0.1	0.5	0.1	0	

47. D PDCD1 Ex.2 pmSTOP + Pulse

	A_{-10}	G_{-9}	A_{-8}	A_{-7}	G_{-6}	G_{-5}	T_{-4}	G_{-3}	G_{-2}	G_{-1}	G_1	G_2	G_3	G_4	T_5	T_6	C_7	C_8	A_9	G_{10}	G_{11}	G_{12}	C_{13}	C_{14}	T_{15}	G_{16}	T_{17}	C_{18}	T_{19}	G_{20}	G_{21}	G_{22}	G_{23}	Indel
A	99.2	0	99.3	99.6	0	0	0	0	0	0	0	0	0	0	0	0	0	0.1	99.4	0.1	0.1	0.1	0	0	0	0.1	0	0	0	0	0.1	0	0.1	1.1
C	0.1	0	0.1	0	0	0	0.2	0	0	0	0	0	0	0	0.3	0.1	99.9	99.9	0.2	0	0	0	99.8	99.9	0.3	0	0.5	100	0.3	0	0	0	0	
G	0.7	100	0.6	0.3	100	100	1.1	100	99.9	100	100	99.9	99.9	99.9	0.2	0.2	0.1	0	0.3	99.9	99.9	99.9	0.1	0	0.3	99.9	1	0	0.6	99.9	99.9	100	99.9	
T	0	0	0	0	0	0	98.7	0	0	0	0	0	0	0.1	99.5	99.7	0	0	0.1	0	0	0	0.1	0	99.4	0.1	98.5	0	99.1	0	0	0	0	

48. D PDCD1 Ex.2 pmSTOP + Pulse

	A_{-10}	G_{-9}	A_{-8}	A_{-7}	G_{-6}	G_{-5}	T_{-4}	G_{-3}	G_{-2}	G_{-1}	G_1	G_2	G_3	G_4	T_5	T_6	C_7	C_8	A_9	G_{10}	G_{11}	G_{12}	C_{13}	C_{14}	T_{15}	G_{16}	T_{17}	C_{18}	T_{19}	G_{20}	G_{21}	G_{22}	G_{23}	Indel
A	99.2	0	99.3	99.7	0	0	0	0	0	0	0	0	0	0	0	0	0	0.1	99.7	0	0	0	0	0	0	0	0	0	0	0	0	0	0	0.8
C	0.1	0	0.2	0.1	0	0	0.1	0	0	0	0	0	0	0	0.3	0.1	99.9	99.9	0.1	0	0	0	99.8	99.9	0.2	0	0.5	99.9	0.3	0	0	0	0	
G	0.6	100	0.5	0.3	100	100	1.1	100	100	99.9	100	99.9	100	99.9	0.2	0.2	0	0	0.2	100	99.9	99.9	0.1	0	0.3	99.9	1	0	0.6	99.9	99.9	99.9	99.9	
T	0	0	0	0	0	0	98.7	0	0	0	0	0	0	0.1	99.6	99.7	0	0	0	0	0	0	0	0	99.5	0	98.5	0	99.1	0	0	0	0	

49. A PDCD1 Ex.2 pmSTOP + Pulse

	A_{-10}	G_{-9}	A_{-8}	A_{-7}	G_{-6}	G_{-5}	T_{-4}	G_{-3}	G_{-2}	G_{-1}	G_1	G_2	G_3	G_4	T_5	T_6	C_7	C_8	A_9	G_{10}	G_{11}	G_{12}	C_{13}	C_{14}	T_{15}	G_{16}	T_{17}	C_{18}	T_{19}	G_{20}	G_{21}	G_{22}	G_{23}	Indel
A	99.5	0	99.5	99.7	0	0	0	0	0	0.1	0	0	0	0	0	0	0	0	99.7	0	0	1.3	0	0	0	0	0	0	0	0.1	0	0	0	0.9
C	0.1	0	0.2	0.1	0	0	0.1	0	0	0	0	0	0	0	0.2	0.1	93.1	93.1	0.2	0	0	0	99.9	100	0.3	0	0.6	100	0.4	0	0	0	0	
G	0.4	100	0.2	0.2	100	100	1.2	100	100	99.9	100	99.9	100	99.8	0.3	0.2	0.1	0	0.1	99.9	99.9	98.7	0	0	0.4	99.9	1.1	0	0.9	99.9	99.9	99.9	99.9	
T	0	0	0	0	0	0	98.6	0	0	0	0	0	0	0.1	99.4	99.7	6.8	6.8	0	0	0	0	0	0	99.3	0	98.3	0	98.8	0.1	0	0	0	

50. A PDCD1 Ex.2 pmSTOP + Pulse

	A_{-10}	G_{-9}	A_{-8}	A_{-7}	G_{-6}	G_{-5}	T_{-4}	G_{-3}	G_{-2}	G_{-1}	G_1	G_2	G_3	G_4	T_5	T_6	C_7	C_8	A_9	G_{10}	G_{11}	G_{12}	C_{13}	C_{14}	T_{15}	G_{16}	T_{17}	C_{18}	T_{19}	G_{20}	G_{21}	G_{22}	G_{23}	Indel
A	99.3	0	99.5	99.7	0	0	0	0	0	0	0	0	0	0	0	0	0.3	0	99.7	0	0	0	0	0	0	0	0	0	0	0	0	0	0	0.9
C	0.2	0	0.2	0.1	0	0	0.2	0	0	0	0	0	0	0	0.2	0.1	97.4	97.8	0.2	0	0	0	99.9	100	0.2	0	0.6	100	0.4	0	0	0	0	
G	0.5	100	0.2	0.2	100	100	1.2	100	100	100	100	100	100	99.8	0.2	0.2	0.1	0	0.1	100	99.9	100	0	0	0.4	100	1	0	0.8	99.9	99.9	99.9	99.9	
T	0	0	0	0	0	0	98.7	0	0	0	0	0	0	0.2	99.5	99.7	2.2	2.1	0	0	0	0	0	0	99.4	0	98.4	0	98.8	0.1	0	0	0	

51. A PDCD1 Ex.3 pmSTOP_1 + BE3 unconc. mRNA 1.5 µg

	G_{-10}	C_{-9}	C_{-8}	A_{-7}	G_{-6}	C_{-5}	C_{-4}	G_{-3}	G_{-2}	C_{-1}	C_1	A_2	G_3	T_4	T_5	C_6	C_7	A_8	A_9	A_{10}	C_{11}	C_{12}	C_{13}	T_{14}	G_{15}	G_{16}	T_{17}	G_{18}	G_{19}	T_{20}	T_{21}	G_{22}	G_{23}	Indel
A	0	0.1	0.4	91.1	0	0.1	0.3	0.1	0	0.1	0.2	96.1	0	0	0	3.9	1.6	95.8	93.8	88.2	0.2	0.1	0.1	0	0	0	0	0	0.1	0	0	0	0	1.4
C	0.6	99.6	99.5	5	0.7	99.8	99.2	0.2	0.1	99.8	99.6	2.1	0.1	0.3	0.7	54.9	77.2	2.7	3.5	7.3	99.3	99.5	99.1	0.7	0	0	0.2	0	0	0.2	0.2	0	0	
G	99.3	0.2	0.1	3.8	99.1	0.1	0.4	99.5	99.7	0.1	0.1	1.7	99.8	1.7	2.7	6	6.3	1.4	2.4	3.9	0.3	0.3	0.5	7	99.8	99.5	1.4	99.9	99.9	0.6	2.1	99.8	99.9	
T	0.1	0	0	0.1	0.2	0	0.1	0.2	0.1	0	0	0	0.2	98	96.6	35.2	14.8	0	0.3	0.6	0.2	0	0.3	92.4	0.1	0.4	98.4	0.1	0	99.2	97.7	0.2	0.1	

52. B PDCD1 Ex.3 pmSTOP_1 + BE3 unconc. mRNA 1.5 µg

	G_{-10}	C_{-9}	C_{-8}	A_{-7}	G_{-6}	C_{-5}	C_{-4}	G_{-3}	G_{-2}	C_{-1}	C_1	A_2	G_3	T_4	T_5	C_6	C_7	A_8	A_9	A_{10}	C_{11}	C_{12}	C_{13}	T_{14}	G_{15}	G_{16}	T_{17}	G_{18}	G_{19}	T_{20}	T_{21}	G_{22}	G_{23}	Indel
A	0	0.1	0.5	91.8	0.1	0.1	0.3	0	0	0.1	0.2	96.1	0	0.1	0.1	2.3	1.1	96.8	94.8	89.3	0.2	0.2	0.1	0	0	0	0	0	0	0	0	0	0	1
C	0.5	99.6	99.2	4.5	0.6	99.7	99.1	0.2	0	99.9	99.6	2.4	0.1	0.3	0.7	74.5	88	1.9	2.9	6.4	99.3	99.5	99.2	0.6	0	0	0.1	0	0	0.1	0.1	0	0	
G	99.4	0.2	0.2	3.5	99.2	0.1	0.4	99.7	99.8	0	0.1	1.5	99.7	1.5	2.5	3.3	2.5	1.2	2.2	3.7	0.3	0.2	0.5	7.2	99.8	99.6	1.7	99.9	99.9	0.6	2.1	99.8	99.9	
T	0.1	0.1	0.1	0.1	0.2	0	0.1	0.2	0.1	0	0	0	0.2	98.2	96.7	19.9	8.4	0.1	0.2	0.6	0.2	0.1	0.2	92.2	0.2	0.3	98.2	0.1	0	99.3	97.7	0.2	0.1	

54. D PDCD1 Ex.3 pmSTOP_1 + BE3 unconc. mRNA 1.5 µg

	G_{-10}	C_{-9}	C_{-8}	A_{-7}	G_{-6}	C_{-5}	C_{-4}	G_{-3}	G_{-2}	C_{-1}	C_1	A_2	G_3	T_4	T_5	C_6	C_7	A_8	A_9	A_{10}	C_{11}	C_{12}	C_{13}	T_{14}	G_{15}	G_{16}	T_{17}	G_{18}	G_{19}	T_{20}	T_{21}	G_{22}	G_{23}	Indel
A	0.1	0.1	0.1	92.3	0.6	0.1	0.1	0.1	0.1	0.1	0.1	91.5	0.1	0.2	0	3.6	1.4	96.7	99.5	99.3	1	0.1	0	0	0.1	0.1	0.1	0	0	0	0	0.1	0	1.6
C	0.2	99.8	99.8	6.6	0.8	99.8	99.8	0.6	0.2	99.7	99.8	8	0.1	0.6	0.1	61.2	80.1	3.1	0.4	0.4	98.8	99.8	99.9	0.8	0	0	0.2	0	0	0.1	0.1	0.2	0.1	
G	99.6	0	0	0.4	98.2	0	0	99	99.6	0.1	0	0.4	99.3	2.5	0.4	3.9	4.3	0.1	0.1	0.2	0.1	0	0	0.9	98.9	99.2	1	99.3	99.5	0.7	0.2	98.4	99.3	
T	0.2	0	0.1	0.7	0.4	0.1	0.1	0.4	0.2	0.1	0	0.2	0.6	96.6	99.4	31.4	14.2	0.1	0	0.1	0.1	0.1	0.1	98.3	1	0.7	98.6	0.7	0.4	99.1	99.7	1.4	0.5	

55. A PDCD1 Ex.3 pmSTOP_1 + BE4 unconc. mRNA 1.5 µg

	G_{-10}	C_{-9}	C_{-8}	A_{-7}	G_{-6}	C_{-5}	C_{-4}	G_{-3}	G_{-2}	C_{-1}	C_1	A_2	G_3	T_4	T_5	C_6	C_7	A_8	A_9	A_{10}	C_{11}	C_{12}	C_{13}	T_{14}	G_{15}	G_{16}	T_{17}	G_{18}	G_{19}	T_{20}	T_{21}	G_{22}	G_{23}	Indel
A	0	0.1	0.5	90.5	0	0.1	0.3	0.1	0	0.1	0.2	95.9	0	0	0	2.5	1.1	95.7	93	87.2	0.1	0.2	0.1	0	0	0.1	0	0	0	0	0	0.1	0	1.1
C	0.6	99.6	99.4	5.5	0.6	99.7	99.2	0.2	0.2	99.8	99.6	2.2	0.1	0.4	1.1	64.5	80.4	2.6	3.6	7.3	99.2	99.3	99.1	0.7	0	0.1	0.1	0	0	0.2	0.4	0	0	
G	99.2	0.2	0.2	3.9	99.2	0.2	0.4	99.5	99.8	0.1	0.1	1.9	99.7	1.6	2.8	4.3	4.1	1.5	3.2	4.5	0.3	0.3	0.6	6.8	99.8	99.4	1.5	99.9	100	0.6	2.1	99.8	99.9	
T	0.2	0	0	0.1	0.1	0	0.1	0.2	0	0.1	0	0.1	0.2	98	96.1	28.7	14.4	0.1	0.3	1.1	0.3	0.1	0.2	92.5	0.2	0.4	98.4	0.1	0	99.2	97.5	0.1	0.1	

56. B PDCD1 Ex.3 pmSTOP_1 + BE4 unconc. mRNA 1.5 µg

	G_{-10}	C_{-9}	C_{-8}	A_{-7}	G_{-6}	C_{-5}	C_{-4}	G_{-3}	G_{-2}	C_{-1}	C_1	A_2	G_3	T_4	T_5	C_6	C_7	A_8	A_9	A_{10}	C_{11}	C_{12}	C_{13}	T_{14}	G_{15}	G_{16}	T_{17}	G_{18}	G_{19}	T_{20}	T_{21}	G_{22}	G_{23}	Indel
A	0	0.1	0.4	91.8	0	0.1	0.3	0.1	0.1	0.1	0.3	95.8	0	0	0	1.6	0.9	97	94.4	89.8	0.1	0.3	0.1	0	0	0	0	0	0	0	0	0	0	0.4
C	0.5	99.6	99.4	4.7	0.6	99.8	99.3	0.2	0	99.8	99.6	2.6	0.1	0.3	0.8	74.2	85.7	1.7	3.1	6	99.5	99.4	99.1	0.6	0	0.1	0.2	0	0	0.2	0.2	0	0	
G	99.4	0.3	0.2	3.5	99.2	0.1	0.3	99.6	99.8	0.1	0.1	1.5	99.7	1.7	2.5	3.5	2.2	1.2	2.3	3.6	0.2	0.2	0.6	7.1	99.7	99.6	1.6	99.9	99.9	0.5	2.3	99.8	99.9	
T	0.1	0	0	0.1	0.1	0	0	0.2	0.1	0	0	0.1	0.2	98	96.7	20.7	11.2	0.1	0.2	0.6	0.1	0.1	0.2	92.2	0.2	0.3	98.2	0.1	0	99.3	97.5	0.2	0.1	

58. D PDCD1 Ex.3 pmSTOP_1 + BE4 unconc. mRNA 1.5 µg

	G_{-10}	C_{-9}	C_{-8}	A_{-7}	G_{-6}	C_{-5}	C_{-4}	G_{-3}	G_{-2}	C_{-1}	C_1	A_2	G_3	T_4	T_5	C_6	C_7	A_8	A_9	A_{10}	C_{11}	C_{12}	C_{13}	T_{14}	G_{15}	G_{16}	T_{17}	G_{18}	G_{19}	T_{20}	T_{21}	G_{22}	G_{23}	Indel
A	0	0.1	0	92.8	0.4	0.1	0	0.1	0.1	0.1	0	91.6	0.1	0.1	0.1	2.3	0.9	97	99.7	99.5	0.8	0.1	0	0	0.1	0	0	0	0	0	0	0.1	0	1.3
C	0.1	99.9	99.9	6	0.7	99.8	99.9	0.4	0.1	99.8	99.9	7.8	0	0.4	0.1	61.2	76.1	2.8	0.2	0.3	98.8	99.8	99.9	0.6	0.1	0	0.2	0	0	0.1	0.1	0.1	0.1	
G	99.8	0	0	0.4	98.6	0	0	99.2	99.6	0	0	0.4	99.5	2.4	0.4	2.8	3.3	0.1	0.1	0.2	0.1	0	0	0.7	98.9	99.4	0.8	99.3	99.6	0.6	0.2	98.4	99.5	
T	0.1	0	0	0.7	0.3	0.1	0.1	0.3	0.2	0.1	0	0.2	0.4	97	99.5	33.7	19.8	0.1	0	0	0.4	0.1	0.1	98.7	1	0.5	99	0.6	0.3	99.3	99.8	1.4	0.4	

59. D PDCD1 Ex.3 pmSTOP_1 + Pulse

	G_{-10}	C_{-9}	C_{-8}	A_{-7}	G_{-6}	C_{-5}	C_{-4}	G_{-3}	G_{-2}	C_{-1}	C_1	A_2	G_3	T_4	T_5	C_6	C_7	A_8	A_9	A_{10}	C_{11}	C_{12}	C_{13}	T_{14}	G_{15}	G_{16}	T_{17}	G_{18}	G_{19}	T_{20}	T_{21}	G_{22}	G_{23}	Indel
A	0	0.2	0.4	90.9	0	0.1	0.3	0	0	0.1	0.2	95.7	0	0	0	0.1	0.2	96.5	94.5	88.5	0.1	0.1	0.1	0	0	0	0	0	0	0	0	0	0	0
C	0.5	99.6	99.4	5.4	0.7	99.7	99.3	0.2	0.1	99.8	99.7	2.5	0	0.3	1.2	99.8	99.7	2.1	2.9	6.9	99.7	99.6	99.2	0.7	0	0	0.1	0	0	0.1	0.2	0	0	
G	99.4	0.2	0.2	3.6	99.1	0.1	0.3	99.6	99.8	0.1	0.1	1.8	99.8	1.5	2.6	0	0.1	1.3	2.4	4	0.2	0.2	0.6	7.3	99.8	99.6	1.6	99.9	99.9	0.5	2.1	99.8	99.9	
T	0.1	0.1	0	0.1	0.1	0.1	0.1	0.2	0.1	0	0	0	0.2	98.1	96.2	0.1	0	0.1	0.2	0.7	0	0	0.2	92	0.2	0.4	98.4	0.1	0	99.4	97.7	0.2	0.1	

60. A PDCD1 Ex.3 pmSTOP_1 + Pulse

	G_{-10}	C_{-9}	C_{-8}	A_{-7}	G_{-6}	C_{-5}	C_{-4}	G_{-3}	G_{-2}	C_{-1}	C_1	A_2	G_3	T_4	T_5	C_6	C_7	A_8	A_9	A_{10}	C_{11}	C_{12}	C_{13}	T_{14}	G_{15}	G_{16}	T_{17}	G_{18}	G_{19}	T_{20}	T_{21}	G_{22}	G_{23}	Indel
A	0	0.2	0.5	91.1	0.1	0.1	0.5	0.1	0	0.1	0.4	95.8	0.1	0	0.1	0.2	0.3	96.1	93.4	88.2	0.1	0.2	0.2	0	0	0	0	0	0	0	0	0	0	0
C	0.5	99.5	99.2	5.2	0.8	99.7	99	0.2	0.1	99.8	99.4	2.4	0.1	0.3	1	99.8	99.6	2.1	3.1	7.1	99.6	99.4	99	0.8	0.1	0	0.1	0	0	0.1	0.2	0	0	
G	99.3	0.3	0.3	3.6	99.1	0.2	0.4	99.5	99.8	0.1	0.2	1.8	99.7	1.7	3.3	0	0.1	1.8	3.3	4	0.3	0.4	0.6	7.6	99.7	99.5	1.6	99.9	100	0.6	2.7	99.8	99.9	
T	0.2	0	0	0.1	0.1	0	0	0.2	0.1	0	0	0	0.2	98	95.6	0	0	0.1	0.2	0.7	0.1	0	0.2	91.6	0.2	0.4	98.2	0.1	0	99.2	97.1	0.2	0.1	

61. A PDCD1 Ex.3 pmSTOP_2 + BE3 unconc. mRNA 1.5 µg

	A_{-10}	T_{-9}	G_{-8}	A_{-7}	C_{-6}	G_{-5}	G_{-4}	C_{-3}	C_{-2}	A_{-1}	G_1	G_2	A_3	C_4	C_5	C_6	A_7	G_8	A_9	C_{10}	T_{11}	A_{12}	G_{13}	C_{14}	A_{15}	G_{16}	C_{17}	A_{18}	C_{19}	C_{20}	A_{21}	G_{22}	G_{23}	Indel
A	99	0	0	97	0.1	0.1	0.1	0.1	0.9	99.2	0	0.1	98.3	10.4	4	2	99.3	0	98.2	0.2	0	99.5	0.1	0.1	99	0	0.6	98.9	0	0.1	98.2	0	0	3.2
C	0.5	0.1	0	2.2	99.7	0	0	99.7	99	0.4	0	0	1.1	80.3	59.3	73.2	0.6	0	1.5	99.4	0	0.3	0	99.8	0.8	0	99.3	0.9	99.9	99.9	1.2	0.2	0.2	
G	0.6	2.1	100	0.7	0.1	99.9	99.9	0.2	0.1	0.4	100	99.9	0.6	0.4	3.8	3.9	0.2	99.9	0.2	0.2	0.6	0.2	99.9	0	0.1	99.9	0	0.2	0	0	0.6	99.7	99.8	
T	0	97.8	0	0	0.1	0	0	0	0	0	0	0	0	9	32.9	20.9	0	0	0	0.2	99.4	0	0	0	0	0.1	0	0	0	0	0	0.2	0	

62. B PDCD1 Ex.3 pmSTOP_2 + BE3 unconc. mRNA 1.5 µg

	A_{-10}	T_{-9}	G_{-8}	A_{-7}	C_{-6}	G_{-5}	G_{-4}	C_{-3}	C_{-2}	A_{-1}	G_1	G_2	A_3	C_4	C_5	C_6	A_7	G_8	A_9	C_{10}	T_{11}	A_{12}	G_{13}	C_{14}	A_{15}	G_{16}	C_{17}	A_{18}	C_{19}	C_{20}	A_{21}	G_{22}	G_{23}	Indel
A	98.6	0.1	0	95.6	0.2	0.1	0.2	0.1	1.5	99.2	0	0	97.8	3.9	2.4	1.9	98.9	0.1	97.8	0.4	0.1	98.9	0	0.3	98.4	0.1	0.8	98.8	0.1	0.1	96.8	0	0	1.8
C	0.6	0.2	0	3	99.7	0	0	99.8	98.4	0.6	0	0	1.6	91.1	79.6	86.2	0.8	0	1.9	99.3	0.1	0.6	0	99.7	1.1	0	99	1.1	99.8	99.8	1.5	0.1	0.2	
G	0.8	3.4	100	1.4	0.1	99.9	99.7	0.1	0	0.2	100	100	0.6	0.2	1.2	1.1	0.3	99.9	0.3	0.3	1.4	0.5	99.9	0	0.4	99.9	0.1	0.1	0	0.1	1.8	99.6	99.7	
T	0	96.3	0	0	0	0	0	0	0	0	0	0	0	4.8	16.8	10.8	0.1	0	0	0	98.4	0	0	0	0	0	0	0	0.1	0	0	0.2	0	

63. C PDCD1 Ex.3 pmSTOP_2 + BE3 unconc. mRNA 1.5 µg

	A_{-10}	T_{-9}	G_{-8}	A_{-7}	C_{-6}	G_{-5}	G_{-4}	C_{-3}	C_{-2}	A_{-1}	G_1	G_2	A_3	C_4	C_5	C_6	A_7	G_8	A_9	C_{10}	T_{11}	A_{12}	G_{13}	C_{14}	A_{15}	G_{16}	C_{17}	A_{18}	C_{19}	C_{20}	A_{21}	G_{22}	G_{23}	Indel
A	97.4	0.3	0.1	97.6	0.6	0	0.1	0.1	1.1	99.1	0.1	0.1	98.3	10.6	5.2	2.3	98.5	0.3	98.8	0.8	0.6	98.2	0.1	1.6	98.8	0.1	2.7	98.7	0.4	0.7	97	0.1	0.1	4.4
C	1.8	0.3	0.1	1.8	99	0	0	99.7	98.6	0.7	0	0.1	0.9	78.6	55.5	69.8	1	0.1	0.8	98.4	0.2	1.2	0.1	97.8	1	0.1	97	1.1	99.5	99.2	2.5	0	0.2	
G	0.7	2.9	99.6	0.6	0.3	99.8	99.8	0	0.2	0.2	99.8	99.6	0.7	0.5	3.3	3.3	0.4	99.5	0.3	0.2	2.2	0.5	99.6	0.4	0.2	99.7	0.3	0.1	0.1	0.1	0.4	99.8	99.6	
T	0.1	96.5	0.2	0	0.1	0.1	0.1	0.1	0.1	0	0.1	0.1	0.1	10.4	36	24.6	0.1	0.2	0.1	0.6	97	0.1	0.1	0.1	0	0.1	0.1	0	0	0	0	0.1	0.1	

64. A PDCD1 Ex.3 pmSTOP_2 + BE4 unconc. mRNA 1.5 µg

	A_{-10}	T_{-9}	G_{-8}	A_{-7}	C_{-6}	G_{-5}	G_{-4}	C_{-3}	C_{-2}	A_{-1}	G_1	G_2	A_3	C_4	C_5	C_6	A_7	G_8	A_9	C_{10}	T_{11}	A_{12}	G_{13}	C_{14}	A_{15}	G_{16}	C_{17}	A_{18}	C_{19}	C_{20}	A_{21}	G_{22}	G_{23}	Indel
A	98.6	0	0.5	95.7	0	0	0.2	0.2	0.9	99.3	0	0	97.5	1.8	1.4	0.5	99.1	0	98.4	0	0	98.8	0	0	99.5	0	0.9	99.8	0	0	97.9	0	0.2	1.4
C	0.9	0	0	3.7	100	0	0	99.5	99.1	0	0	0	1.6	89	86.4	92.2	0.9	0	1.6	99.1	0	0.9	0	100	0.5	0	99.1	0.2	99.8	100	1.8	0.2	0	
G	0.5	3.4	99.5	0.7	0	100	99.8	0.2	0	0.7	100	100	0.9	0	1.8	0.2	0	100	0	0	0.7	0.2	100	0	0	100	0	0	0.2	0	0.2	99.8	99.8	
T	0	96.6	0	0	0	0	0	0	0	0	0	0	0	9.2	10.3	7.1	0	0	0	0.9	99.3	0	0	0	0	0	0	0	0	0	0	0	0	

65. B PDCD1 Ex.3 pmSTOP_2 + BE4 unconc. mRNA 1.5 µg

	A_{-10}	T_{-9}	G_{-8}	A_{-7}	C_{-6}	G_{-5}	G_{-4}	C_{-3}	C_{-2}	A_{-1}	G_1	G_2	A_3	C_4	C_5	C_6	A_7	G_8	A_9	C_{10}	T_{11}	A_{12}	G_{13}	C_{14}	A_{15}	G_{16}	C_{17}	A_{18}	C_{19}	C_{20}	A_{21}	G_{22}	G_{23}	Indel
A	98.8	0.1	0	95.8	0.1	0.1	0	0.1	1.1	99.1	0.1	0.2	98	5.8	2.4	1.2	99.1	0	97.7	0.3	0	99.1	0.1	0.2	98.7	0	0.8	98.5	0.1	0	97.1	0	0	1.7
C	0.7	0.2	0	3.1	99.5	0.2	0	99.9	98.8	0.6	0	0.1	1.4	86.7	75	82.7	0.8	0	1.9	99.3	0.2	0.4	0	99.8	1.1	0	99.1	1.2	99.8	99.9	1.6	0.3	0.3	
G	0.5	3.1	100	1	0	99.8	99.9	0	0.1	0.3	99.9	99.7	0.5	0.2	1.8	1.3	0.1	99.9	0.3	0.2	1.1	0.4	99.8	0	0.2	99.9	0.1	0.2	0	0	1.3	99.4	99.6	
T	0	96.6	0	0	0.4	0	0	0	0	0	0	0	0	7.4	20.8	14.9	0	0	0	0.3	98.7	0	0.1	0	0	0	0	0	0.1	0	0	0.2	0.1	

66. C PDCD1 Ex.3 pmSTOP_2 + BE4 unconc. mRNA 1.5 µg

	A_{-10}	T_{-9}	G_{-8}	A_{-7}	C_{-6}	G_{-5}	G_{-4}	C_{-3}	C_{-2}	A_{-1}	G_1	G_2	A_3	C_4	C_5	C_6	A_7	G_8	A_9	C_{10}	T_{11}	A_{12}	G_{13}	C_{14}	A_{15}	G_{16}	C_{17}	A_{18}	C_{19}	C_{20}	A_{21}	G_{22}	G_{23}	Indel
A	97.5	0.2	0.2	97.7	0.6	0	0	0.1	1.1	99.3	0	0.2	98.2	8.4	3.2	1.4	98.7	0.2	98.8	0.8	0.5	98.6	0.1	1.6	98.8	0.2	2.6	98.8	0.4	0.7	97.2	0	0.1	2.8
C	1.7	0.3	0.1	1.8	99	0	0	99.8	98.7	0.5	0	0	0.9	77.2	60.1	69.8	0.9	0.1	0.8	98	0.2	1	0.1	97.9	0.9	0.1	97	1.1	99.5	99.2	2.4	0	0.2	
G	0.6	2.9	99.6	0.5	0.3	99.9	99.9	0	0.2	0.2	99.9	99.7	0.7	0.4	2	1.7	0.4	99.5	0.3	0.3	1.9	0.4	99.7	0.4	0.2	99.7	0.3	0.1	0.1	0.1	0.4	99.9	99.6	
T	0.1	96.6	0.2	0	0.1	0	0.1	0.1	0.1	0	0	0.1	0.2	14	34.8	27.1	0	0.2	0.1	0.9	97.5	0.1	0.1	0.1	0	0.1	0.1	0	0	0	0	0.1	0.1	

67. D PDCD1 Ex.3 pmSTOP_2 + Pulse

	A_{-10}	T_{-9}	G_{-8}	A_{-7}	C_{-6}	G_{-5}	G_{-4}	C_{-3}	C_{-2}	A_{-1}	G_{1}	G_{2}	A_{3}	C_{4}	C_{5}	C_{6}	A_{7}	G_{8}	A_{9}	C_{10}	T_{11}	A_{12}	G_{13}	C_{14}	A_{15}	G_{16}	C_{17}	A_{18}	C_{19}	C_{20}	A_{21}	G_{22}	G_{23}	Indel
A	99	0	0	96.8	0.1	0.1	0	0	1	99.3	0	0	98	0.1	0	0.5	99.3	0	97.9	0.2	0	99.2	0	0.2	98.8	0.1	0.6	99	0.1	0.1	97.8	0	0	0
C	0.4	0.1	0	2.4	99.8	0	0	99.9	99	0.4	0	0	1.4	99.9	100	99.5	0.6	0	1.8	99.8	0.1	0.5	0	99.8	1	0	99.3	0.8	99.9	99.9	1.4	0.1	0.2	
G	0.6	2.6	100	0.8	0	99.9	99.9	0	0	0.3	100	100	0.5	0	0	0	0.1	99.9	0.3	0	0.6	0.3	99.9	0	0.3	99.9	0	0.2	0	0	0.8	99.7	99.8	
T	0	97.3	0	0	0.1	0	0	0	0	0	0	0	0	0	0	0	0	0	0	0	99.3	0	0.1	0	0	0	0	0	0	0	0	0.1	0	

68. A PDCD1 Ex.3 pmSTOP_2 + Pulse

	A_{-10}	T_{-9}	G_{-8}	A_{-7}	C_{-6}	G_{-5}	G_{-4}	C_{-3}	C_{-2}	A_{-1}	G_{1}	G_{2}	A_{3}	C_{4}	C_{5}	C_{6}	A_{7}	G_{8}	A_{9}	C_{10}	T_{11}	A_{12}	G_{13}	C_{14}	A_{15}	G_{16}	C_{17}	A_{18}	C_{19}	C_{20}	A_{21}	G_{22}	G_{23}	Indel
A	98.9	0	0	93.2	0	0	0	0	1.1	100	0	0	95.5	0	0	3.4	97.7	0	98.9	0	0	98.9	0	1.1	100	0	1.1	97.7	1.1	0	94.3	0	0	0
C	1.1	0	0	6.8	98.9	0	0	100	98.9	0	0	0	3.4	100	98.9	95.5	2.3	0	1.1	100	0	0	0	98.9	0	0	98.9	2.3	98.9	98.9	1.1	0	0	
G	0	6.8	100	0	1.1	100	100	0	0	0	100	100	1.1	0	1.1	1.1	0	100	0	0	2.3	1.1	100	0	0	100	0	0	0	1.1	4.5	100	100	
T	0	93.2	0	0	0	0	0	0	0	0	0	0	0	0	0	0	0	0	0	0	97.7	0	0	0	0	0	0	0	0	0	0	0	0	

69. A TRAC Ex.1 SD + BE3 unconc. mRNA 1.5 µg

	C_{-10}	A_{-9}	A_{-8}	A_{-7}	G_{-6}	C_{-5}	T_{-4}	G_{-3}	C_{-2}	C_{-1}	C_{1}	T_{2}	T_{3}	A_{4}	C_{5}	C_{6}	T_{7}	G_{8}	G_{9}	G_{10}	C_{11}	T_{12}	G_{13}	G_{14}	G_{15}	G_{16}	A_{17}	A_{18}	G_{19}	A_{20}	A_{21}	G_{22}	G_{23}	Indel
A	0	98.7	99.9	99.7	0	0	0	0	0.1	0	0	0.1	1.2	99.7	15.5	13	0.1	0.1	0.1	0	0	0	0	0.1	0.1	0.1	99.7	99.7	0	99.3	99.6	0	0	14.8
C	100	1.2	0	0	0	99.9	0.1	0	99.7	99.9	99.6	0.2	0.1	0.1	29	31.9	0.1	0.1	0	0	99.8	0.1	0	0	0	0	0.2	0.1	0	0.5	0.2	0	0	
G	0	0.1	0	0.3	100	0	0	99.7	0.1	0	0	0	0	0	2.8	9.7	0	99.8	99.8	99.9	0	0	99.9	99.9	99.9	99.8	0	0.2	99.8	0.1	0.2	99.9	100	
T	0	0	0	0	0	0	99.9	0.2	0.2	0.1	0.4	99.7	98.7	0.2	52.7	45.5	99.7	0	0.1	0	0.1	99.8	0	0	0	0	0	0	0.1	0	0	0	0	

70. B TRAC Ex.1 SD + BE3 unconc. mRNA 1.5 µg

	C_{-10}	A_{-9}	A_{-8}	A_{-7}	G_{-6}	C_{-5}	T_{-4}	G_{-3}	C_{-2}	C_{-1}	C_{1}	T_{2}	T_{3}	A_{4}	C_{5}	C_{6}	T_{7}	G_{8}	G_{9}	G_{10}	C_{11}	T_{12}	G_{13}	G_{14}	G_{15}	G_{16}	A_{17}	A_{18}	G_{19}	A_{20}	A_{21}	G_{22}	G_{23}	Indel
A	0	99.9	99.9	99.8	0	0	0.1	0.1	0	0	0	0.1	1.4	99.9	15.8	13.1	0	0	0.1	0.1	0	0	0	0	0	0.2	99.9	99.8	0.1	99.6	99.8	0	0	7.4
C	99.9	0	0	0	0	99.9	0.1	0	99.9	99.9	99.9	0.2	0.1	0.1	15.2	18.5	0.1	0	0	0	99.9	0.1	0	0	0	0	0	0	0	0.2	0	0	0	
G	0	0.1	0	0.2	99.9	0	0	99.9	0	0	0	0	0	0	3.3	12	0	99.9	99.9	99.9	0	0	99.9	100	100	99.8	0	0.2	99.9	0.2	0.2	99.9	100	
T	0.1	0	0	0	0.1	0.1	99.9	0	0.1	0.1	0.1	99.7	98.5	0.1	65.7	56.4	99.9	0	0	0	0.1	99.9	0	0	0	0	0	0	0	0	0	0	0	

71. C TRAC Ex.1 SD + BE3 unconc. mRNA 1.5 µg

	C_{-10}	A_{-9}	A_{-8}	A_{-7}	G_{-6}	C_{-5}	T_{-4}	G_{-3}	C_{-2}	C_{-1}	C_{1}	T_{2}	T_{3}	A_{4}	C_{5}	C_{6}	T_{7}	G_{8}	G_{9}	G_{10}	C_{11}	T_{12}	G_{13}	G_{14}	G_{15}	G_{16}	A_{17}	A_{18}	G_{19}	A_{20}	A_{21}	G_{22}	G_{23}	Indel
A	0	99.9	100	99.8	0	0	0	0	0	0	0	0	0.8	99.8	12.1	10.7	0	0.1	0	0	0	0	0	0	0	0.1	99.9	99.7	0	99.6	99.7	0	0	5.3
C	99.9	0	0	0	0	99.9	0.1	0	99.9	99.9	99.9	0.2	0.1	0.1	40.5	44.1	0.2	0	0	0	99.9	0.1	0	0	0	0	0	0	0	0.2	0	0	0	
G	0	0.1	0	0.2	100	0	0	99.9	0	0	0	0	0	0	2.4	6.8	0	99.9	100	100	0	0	100	100	100	99.9	0.1	0.2	100	0.2	0.2	100	100	
T	0	0	0	0	0	0	99.9	0	0.1	0	0.1	99.8	99.2	0.1	45	38.5	99.8	0	0	0	0.1	99.8	0	0	0	0	0	0	0	0	0	0	0	

72. D TRAC Ex.1 SD + BE3 unconc. mRNA 1.5 µg

	C_{-10}	A_{-9}	A_{-8}	A_{-7}	G_{-6}	C_{-5}	T_{-4}	G_{-3}	C_{-2}	C_{-1}	C_{1}	T_{2}	T_{3}	A_{4}	C_{5}	C_{6}	T_{7}	G_{8}	G_{9}	G_{10}	C_{11}	T_{12}	G_{13}	G_{14}	G_{15}	G_{16}	A_{17}	A_{18}	G_{19}	A_{20}	A_{21}	G_{22}	G_{23}	Indel
A	0	99.9	100	99.7	0	0	0	0.1	0	0	0	0.1	0.8	99.8	15.6	13.5	0.1	0	0	0.1	0	0	0	0.1	0	0.1	99.8	99.7	0.1	99.7	99.7	0	0	6.9
C	99.9	0	0	0	0	99.9	0.1	0	99.9	99.9	99.8	0.2	0.1	0.1	26.7	30.6	0.1	0	0	0	99.9	0.1	0	0	0	0	0	0	0	0.2	0	0	0	
G	0	0.1	0	0.2	100	0	0	99.9	0	0	0	0	0	0	3.3	9.4	0	99.9	99.9	99.9	0	0	99.9	99.9	99.9	99.9	0.1	0.3	99.9	0.2	0.3	100	100	
T	0	0	0	0	0	0	99.9	0	0	0.1	0.2	99.8	99.1	0.1	54.4	46.5	99.8	0	0	0	0.1	99.8	0	0	0	0	0	0	0	0	0	0	0	

73. A TRAC Ex.1 SD + BE4 unconc. mRNA 1.5 µg

	C_{-10}	A_{-9}	A_{-8}	A_{-7}	G_{-6}	C_{-5}	T_{-4}	G_{-3}	C_{-2}	C_{-1}	C_{1}	T_{2}	T_{3}	A_{4}	C_{5}	C_{6}	T_{7}	G_{8}	G_{9}	G_{10}	C_{11}	T_{12}	G_{13}	G_{14}	G_{15}	G_{16}	A_{17}	A_{18}	G_{19}	A_{20}	A_{21}	G_{22}	G_{23}	Indel
A	0	98.6	100	99.7	0	0	0	0	0	0	0	0.1	0.5	99.8	5.9	5.8	0	0	0	0	0	0	0.1	0	0	0.1	99.8	99.8	0.1	99.3	99.5	0	0	6.6
C	99.9	1.3	0	0	0	99.9	0.1	0	99.8	99.8	99.9	0.1	0	0.1	20.5	21	0.1	0	0	0	99.8	0.1	0	0	0	0	0.1	0.1	0	0.6	0.2	0	0	
G	0	0	0	0.2	100	0	0	99.8	0.1	0	0	0	0	0	1.5	5.3	0	100	100	99.9	0	0	99.9	100	100	99.9	0	0.2	99.9	0.1	0.2	99.9	99.9	
T	0.1	0	0	0	0	0.1	99.9	0.1	0.1	0.1	0.1	99.7	99.4	0	72.1	67.8	99.9	0	0	0	0.2	99.8	0	0	0	0	0.1	0	0	0	0	0	0	

74. B TRAC Ex.1 SD + BE4 unconc. mRNA 1.5 µg

	C_{-10}	A_{-9}	A_{-8}	A_{-7}	G_{-6}	C_{-5}	T_{-4}	G_{-3}	C_{-2}	C_{-1}	C_{1}	T_{2}	T_{3}	A_{4}	C_{5}	C_{6}	T_{7}	G_{8}	G_{9}	G_{10}	C_{11}	T_{12}	G_{13}	G_{14}	G_{15}	G_{16}	A_{17}	A_{18}	G_{19}	A_{20}	A_{21}	G_{22}	G_{23}	Indel
A	0	99.9	100	99.8	0	0	0	0.1	0	0	0	0	0.9	99.8	9.4	7.6	0.1	0	0	0	0	0	0	0	0	0.1	99.9	99.8	0	99.7	99.7	0	0	4.7
C	99.9	0	0	0	0	99.9	0.1	0	99.9	99.8	99.9	0.1	0	0.1	25.5	27.1	0.1	0	0	0	99.9	0.1	0	0	0	0	0	0	0	0.1	0.1	0	0	
G	0	0.1	0	0.2	100	0	0	99.9	0	0	0	0	0	0	2.1	7	0	99.9	99.9	99.9	0	0	100	100	99.9	99.9	0.1	0.2	99.9	0.1	0.2	100	100	
T	0	0	0	0	0	0	99.9	0	0.1	0.1	0.1	99.8	99.1	0.1	63	58.2	99.8	0	0	0	0.1	99.9	0	0	0	0	0	0	0	0	0	0	0	

75. C TRAC Ex.1 SD + BE4 unconc. mRNA 1.5 µg

	C_{-10}	A_{-9}	A_{-8}	A_{-7}	G_{-6}	C_{-5}	T_{-4}	G_{-3}	C_{-2}	C_{-1}	C_1	T_2	T_3	A_4	C_5	C_6	T_7	G_8	G_9	G_{10}	C_{11}	T_{12}	G_{13}	G_{14}	G_{15}	G_{16}	A_{17}	A_{18}	G_{19}	A_{20}	A_{21}	G_{22}	G_{23}	Indel
A	0	99.9	100	99.7	0	0	0	0.1	0	0	0	0	0.3	99.9	8	6.1	0	0	0	0	0	0	0	0	0	0.1	99.8	99.7	0	99.6	99.7	0	0	2.8
C	100	0	0	0	0	99.9	0.1	0	100	99.9	99.9	0.2	0	0	38.1	39.2	0.1	0	0	0	99.9	0.1	0	0	0	0	0	0	0	0.2	0	0	0	
G	0	0.1	0	0.2	100	0	0	99.8	0	0	0	0	0	0	1.6	5.6	0	99.9	99.9	99.9	0	0	100	100	100	99.9	0.1	0.3	100	0.2	0.2	100	100	
T	0	0	0	0	0	0	99.9	0	0	0	0.1	99.8	99.6	0	52.2	49.1	99.9	0	0	0	0.1	99.9	0	0	0	0	0	0	0	0	0	0	0	

76. D TRAC Ex.1 SD + BE4 unconc. mRNA 1.5 µg

	C_{-10}	A_{-9}	A_{-8}	A_{-7}	G_{-6}	C_{-5}	T_{-4}	G_{-3}	C_{-2}	C_{-1}	C_1	T_2	T_3	A_4	C_5	C_6	T_7	G_8	G_9	G_{10}	C_{11}	T_{12}	G_{13}	G_{14}	G_{15}	G_{16}	A_{17}	A_{18}	G_{19}	A_{20}	A_{21}	G_{22}	G_{23}	Indel
A	0	99.9	100	99.7	0	0	0	0.1	0	0	0	0	0.6	99.8	9.6	7.8	0.1	0	0	0	0	0	0	0	0	0.1	99.9	99.8	0	99.7	99.8	0	0	4.2
C	99.9	0	0	0	0	99.9	0.1	0	99.9	99.8	99.8	0.2	0.1	0	34.8	36	0.1	0	0	0	99.9	0.1	0	0	0	0	0	0	0	0.2	0	0	0	
G	0	0.1	0	0.2	100	0	0	99.8	0	0	0	0	0	0	1.9	6	0	100	99.9	99.9	0	0	100	100	99.9	99.9	0.1	0.2	99.9	0.2	0.2	100	100	
T	0.1	0	0	0	0	0.1	99.9	0	0.1	0.1	0.1	99.8	99.3	0	53.8	50.3	99.8	0	0	0	0.1	99.9	0	0	0	0	0	0	0	0	0	0	0	

77. D TRAC Ex.1 SD + Cas9 mRNA 1.5 µg

	C_{-10}	A_{-9}	A_{-8}	A_{-7}	G_{-6}	C_{-5}	T_{-4}	G_{-3}	C_{-2}	C_{-1}	C_1	T_2	T_3	A_4	C_5	C_6	T_7	G_8	G_9	G_{10}	C_{11}	T_{12}	G_{13}	G_{14}	G_{15}	G_{16}	A_{17}	A_{18}	G_{19}	A_{20}	A_{21}	G_{22}	G_{23}	Indel
A	0	99.9	99.9	99.7	0	0	0.1	0.1	0	0.1	0	0.2	0	99.7	0.2	0.1	0.4	0	0.2	0.1	0.2	0.5	0.2	0.2	0.3	0.3	97.8	99.3	0.1	99.5	99.5	0.1	0	82.7
C	100	0	0	0	0	99.8	0.2	0.1	99.8	99.8	99.8	0.2	0.1	0.1	99.8	99.8	0.3	0.1	0.3	0.5	99.3	0.4	0.2	0.2	0.2	0.4	0.5	0.1	0.2	0.2	0.1	0.1	0.1	
G	0	0.1	0.1	0.3	99.9	0.1	0	99.8	0.1	0	0.1	0	0	0.1	0	0	0	99.7	99.5	98.8	0.1	0	99.3	99.4	99.2	98.6	0.3	0.3	99.3	0.2	0.2	99.5	99.7	
T	0	0	0	0	0	0	99.7	0.1	0.1	0.1	0.1	99.6	99.9	0.1	0	0	99.3	0.1	0	0.7	0.4	99.1	0.2	0.3	0.4	0.7	1.4	0.3	0.4	0	0.1	0.3	0.1	

78. D TRAC Ex.1 SD + Pulse

	C_{-10}	A_{-9}	A_{-8}	A_{-7}	G_{-6}	C_{-5}	T_{-4}	G_{-3}	C_{-2}	C_{-1}	C_1	T_2	T_3	A_4	C_5	C_6	T_7	G_8	G_9	G_{10}	C_{11}	T_{12}	G_{13}	G_{14}	G_{15}	G_{16}	A_{17}	A_{18}	G_{19}	A_{20}	A_{21}	G_{22}	G_{23}	Indel
A	0	99.9	100	99.7	0	0	0	0	0	0	0	0	0	99.8	0.2	0.1	0	0	0	0	0	0	0	0	0.1	0	99.9	99.7	0	99.7	99.7	0	0	0.1
C	100	0	0	0	0	100	0.2	0	100	99.9	100	0.1	0	0.1	99.6	99.7	0.1	0	0	0	100	0.1	0	0	0	0	0	0	0	0.1	0	0	0	
G	0	0.1	0	0.2	100	0	0	100	0	0	0	0	0	0.1	0	0	0	99.9	100	100	0	0	99.9	99.9	99.9	100	0.1	0.3	100	0.2	0.3	100	100	
T	0	0	0	0	0	0	99.8	0	0	0.1	0	99.8	100	0	0.2	0.2	99.9	0	0	0	0	99.9	0	0	0	0	0	0	0	0	0	0	0	

79. A TRAC Ex.1 SD + Pulse

	C_{-10}	A_{-9}	A_{-8}	A_{-7}	G_{-6}	C_{-5}	T_{-4}	G_{-3}	C_{-2}	C_{-1}	C_1	T_2	T_3	A_4	C_5	C_6	T_7	G_8	G_9	G_{10}	C_{11}	T_{12}	G_{13}	G_{14}	G_{15}	G_{16}	A_{17}	A_{18}	G_{19}	A_{20}	A_{21}	G_{22}	G_{23}	Indel
A	0	99.9	100	99.7	0	0	0	0	0	0	0	0	0	99.9	0.2	0	0	0	0	0	0	0	0	0	0	0	99.8	99.7	0	99.6	99.7	0	0	0.1
C	100	0	0	0	0	100	0.1	0	100	100	100	0.1	0	0	99.8	99.9	0.2	0	0	0	100	0.1	0	0	0	0	0	0	0	0.2	0	0	0	
G	0	0.1	0	0.3	100	0	0	100	0	0	0	0	0	0.1	0	0	0	100	99.9	100	0	0	100	100	99.9	100	0.1	0.2	100	0.2	0.2	100	100	
T	0	0	0	0	0	0	99.9	0	0	0	0	99.8	100	0	0	0	99.8	0	0	0	0	99.9	0	0	0	0	0	0	0	0	0	0	0	

80. A TRAC Ex.3 SA + BE3 unconc. mRNA 1.5 µg

	A_{-10}	A_{-9}	A_{-8}	G_{-7}	T_{-6}	T_{-5}	T_{-4}	A_{-3}	G_{-2}	G_{-1}	T_1	T_2	C_3	G_4	T_5	A_6	T_7	C_8	T_9	G_{10}	T_{11}	A_{12}	A_{13}	A_{14}	A_{15}	C_{16}	C_{17}	A_{18}	A_{19}	G_{20}	A_{21}	G_{22}	G_{23}	Indel
A	99.8	99.8	99.8	0	0	0	0	99.8	0	0	0	0	1.4	0.1	0	99.9	0.2	10.9	0	0	0	100	99.8	99.8	99.8	0.1	0.2	99.8	99.7	0	99.4	0	0	6.7
C	0.1	0.1	0	0	0.1	0.1	0	0	0	0	0.1	0.1	89.1	0	0.1	0	0.1	21.6	0.1	0	0	0	0.1	0.1	0	99.7	99.7	0.1	0.1	0	0.3	0	0	
G	0.1	0.1	0.2	100	0	0	0	0.1	100	100	0.1	0	0.2	99.8	0	0	0	24.5	0	99.9	0	0	0.1	0.1	0.2	0	0.1	0.1	0.2	99.8	0.3	99.9	99.9	
T	0	0	0	0	99.9	99.9	100	0	0	0	99.8	99.9	9.3	0.1	99.9	0.1	99.7	43	99.9	0.1	99.9	0	0	0	0	0.1	0	0	0	0.1	0	0	0.1	

81. B TRAC Ex.3 SA + BE3 unconc. mRNA 1.5 µg

	A_{-10}	A_{-9}	A_{-8}	G_{-7}	T_{-6}	T_{-5}	T_{-4}	A_{-3}	G_{-2}	G_{-1}	T_1	T_2	C_3	G_4	T_5	A_6	T_7	C_8	T_9	G_{10}	T_{11}	A_{12}	A_{13}	A_{14}	A_{15}	C_{16}	C_{17}	A_{18}	A_{19}	G_{20}	A_{21}	G_{22}	G_{23}	Indel
A	99.8	99.9	99.8	0	0	0	0	99.9	0	0	0	0	1.4	0	0.1	99.9	0.3	11.9	0	0	0	100	99.8	99.8	99.8	0.1	0	99.9	99.8	0	99.8	0.1	0.1	5.2
C	0.1	0.1	0	0	0.1	0.1	0	0	0	0	0.2	0.1	89.8	0	0.1	0	0.1	17.2	0	0	0.1	0	0.1	0	0	99.9	99.9	0	0	0	0	0	0	
G	0	0	0.2	100	0.1	0	0	0.1	99.9	100	0.1	0	0.3	100	0	0	0	30.4	0	99.9	0	0	0.1	0.1	0.1	0	0	0	0.2	99.9	0.2	99.9	99.9	
T	0	0	0	0	99.8	99.8	99.9	0	0	0	99.7	99.8	8.6	0	99.8	0.1	99.6	40.4	99.9	0	99.9	0	0	0	0	0.1	0.1	0	0	0.1	0	0	0	

82. C TRAC Ex.3 SA + BE3 unconc. mRNA 1.5 µg

	A_{-10}	A_{-9}	A_{-8}	G_{-7}	T_{-6}	T_{-5}	T_{-4}	A_{-3}	G_{-2}	G_{-1}	T_1	T_2	C_3	G_4	T_5	A_6	T_7	C_8	T_9	G_{10}	T_{11}	A_{12}	A_{13}	A_{14}	A_{15}	C_{16}	C_{17}	A_{18}	A_{19}	G_{20}	A_{21}	G_{22}	G_{23}	Indel
A	99.8	99.9	99.8	0	0	0	0	99.8	0	0	0	0	1	0	0	99.9	0.2	9.1	0	0	0	100	99.8	99.8	99.8	0	0	99.9	99.8	0	99.8	0	0	4
C	0.1	0.1	0	0	0	0.1	0	0.1	0	0	0.2	0.1	94.4	0	0.1	0	0.1	48.1	0.1	0	0	0	0	0	0	99.8	99.9	0	0	0	0	0	0	
G	0	0	0.2	99.9	0	0	0	0.1	99.9	99.9	0.1	0	0.1	99.9	0	0	0	19.5	0	100	0	0	0.1	0.1	0.2	0	0	0.1	0.1	99.9	0.2	100	99.9	
T	0	0	0	0	99.9	99.9	100	0	0	0	99.7	99.8	4.5	0	99.9	0	99.8	23.3	99.9	0	99.9	0	0	0	0	0.1	0	0	0	0	0	0	0	

83. D TRAC Ex.3 SA + BE3 unconc. mRNA 1.5 µg

	A_{-10}	A_{-9}	A_{-8}	G_{-7}	T_{-6}	T_{-5}	T_{-4}	A_{-3}	G_{-2}	G_{-1}	T_1	T_2	C_3	G_4	T_5	A_6	T_7	C_8	T_9	G_{10}	T_{11}	A_{12}	A_{13}	A_{14}	A_{15}	C_{16}	C_{17}	A_{18}	A_{19}	G_{20}	A_{21}	G_{22}	G_{23}	Indel
A	99.8	99.8	99.7	0	0	0	0	99.9	0	0.1	0	0	1.4	0.1	0	99.9	0.2	10.7	0	0	0	99.9	99.8	99.9	99.8	0	0	99.9	99.8	0	99.8	0	0	4.5
C	0.2	0.1	0	0	0.1	0.1	0	0	0	0	0.2	0.1	91.2	0	0.1	0	0.1	32.6	0.1	0	0	0	0	0	0	99.8	99.9	0	0	0	0	0	0	
G	0	0	0.2	100	0	0	0	0	99.9	99.9	0.1	0	0.2	99.9	0	0	0	24.1	0	100	0	0	0.1	0.1	0.2	0	0	0	0.1	99.8	0.2	99.9	99.9	
T	0	0	0	0	99.9	99.9	99.9	0	0.1	0	99.7	99.8	7.1	0	99.9	0.1	99.7	32.6	99.8	0	99.9	0	0	0	0	0.1	0	0	0	0.1	0	0	0	

84. A TRAC Ex.3 SA + BE4 unconc. mRNA 1.5 µg

	A_{-10}	A_{-9}	A_{-8}	G_{-7}	T_{-6}	T_{-5}	T_{-4}	A_{-3}	G_{-2}	G_{-1}	T_1	T_2	C_3	G_4	T_5	A_6	T_7	C_8	T_9	G_{10}	T_{11}	A_{12}	A_{13}	A_{14}	A_{15}	C_{16}	C_{17}	A_{18}	A_{19}	G_{20}	A_{21}	G_{22}	G_{23}	Indel
A	99.8	99.8	99.7	0	0	0	0	99.9	0	0	0	0	0.6	0.1	0	100	0.1	5.5	0	0	0	100	99.8	99.8	99.8	0	0.1	99.6	99.5	0	98.7	0.1	0.1	3
C	0.1	0.2	0.1	0	0.1	0.1	0	0	0	0	0.1	0.1	83.1	0	0.1	0	0.1	18.4	0.1	0	0	0	0	0.1	0.1	99.8	99.8	0.2	0.3	0	0.8	0.1	0	
G	0	0	0.2	99.9	0	0	0	0.1	99.9	99.9	0.1	0	0.1	99.9	0	0	0	13.2	0	99.9	0	0	0.1	0.1	0.2	0	0	0.1	0.2	99.8	0.4	99.8	99.9	
T	0	0	0	0	99.9	99.9	100	0	0	0	99.8	99.8	16.2	0	99.9	0	99.8	62.8	99.9	0	99.9	0	0	0	0	0.1	0.1	0	0	0.1	0	0.1	0.1	

85. B TRAC Ex.3 SA + BE4 unconc. mRNA 1.5 µg

	A_{-10}	A_{-9}	A_{-8}	G_{-7}	T_{-6}	T_{-5}	T_{-4}	A_{-3}	G_{-2}	G_{-1}	T_1	T_2	C_3	G_4	T_5	A_6	T_7	C_8	T_9	G_{10}	T_{11}	A_{12}	A_{13}	A_{14}	A_{15}	C_{16}	C_{17}	A_{18}	A_{19}	G_{20}	A_{21}	G_{22}	G_{23}	Indel
A	99.6	99.7	99.7	0	0	0	0	99.9	0	0	0	0	0.5	0.1	0	99.9	0.1	8.2	0	0	0	99.9	99.6	99.7	99.7	0.1	0	99.9	99.7	0	99.5	0	0	3.1
C	0.3	0.2	0.1	0	0.2	0.1	0	0	0	0	0.3	0.1	90.1	0	0.1	0	0.2	33.6	0.2	0	0.1	0	0.1	0	0	99.8	99.9	0	0	0	0.1	0	0	
G	0.1	0.1	0.2	100	0	0	0	0	100	100	0.1	0	0.1	99.9	0	0	0.1	19.8	0	99.9	0	0.1	0.3	0.2	0.3	0	0	0.1	0.3	99.8	0.4	100	99.9	
T	0	0	0	0	99.8	99.8	100	0	0	0	99.6	99.9	9.4	0	99.9	0	99.7	38.5	99.8	0	99.9	0	0	0	0	0.1	0.1	0	0	0.1	0	0	0	

86. C TRAC Ex.3 SA + BE4 unconc. mRNA 1.5 µg

	A_{-10}	A_{-9}	A_{-8}	G_{-7}	T_{-6}	T_{-5}	T_{-4}	A_{-3}	G_{-2}	G_{-1}	T_1	T_2	C_3	G_4	T_5	A_6	T_7	C_8	T_9	G_{10}	T_{11}	A_{12}	A_{13}	A_{14}	A_{15}	C_{16}	C_{17}	A_{18}	A_{19}	G_{20}	A_{21}	G_{22}	G_{23}	Indel
A	99.8	99.9	99.8	0	0	0	0	99.9	0	0	0	0	0.7	0	0	100	0.1	5.6	0	0	0	100	99.8	99.9	99.8	0	0	100	99.8	0	99.8	0	0	2.5
C	0.1	0.1	0	0	0	0.1	0	0	0	0	0.2	0.1	89	0	0.1	0	0.1	32.7	0.1	0	0	0	0.1	0	0	99.9	99.9	0	0	0	0	0	0	
G	0	0	0.2	100	0	0	0	0	99.9	100	0.1	0	0.1	100	0	0	0	16.5	0	100	0	0	0.1	0.1	0.2	0	0	0	0.1	99.9	0.2	100	99.9	
T	0	0	0	0	99.9	99.9	100	0	0	0	99.8	99.9	10.3	0	99.9	0	99.8	45.2	99.9	0	99.9	0	0	0	0	0.1	0.1	0	0	0	0	0	0	

87. D TRAC Ex.3 SA + BE4 unconc. mRNA 1.5 µg

	A_{-10}	A_{-9}	A_{-8}	G_{-7}	T_{-6}	T_{-5}	T_{-4}	A_{-3}	G_{-2}	G_{-1}	T_1	T_2	C_3	G_4	T_5	A_6	T_7	C_8	T_9	G_{10}	T_{11}	A_{12}	A_{13}	A_{14}	A_{15}	C_{16}	C_{17}	A_{18}	A_{19}	G_{20}	A_{21}	G_{22}	G_{23}	Indel
A	99.8	99.8	99.8	0	0	0	0	99.9	0.1	0	0	0	0.8	0	0	99.9	0.1	7.8	0	0	0	99.9	99.8	99.9	99.8	0	0	99.9	99.8	0	99.8	0	0	3.1
C	0.1	0.1	0	0	0.1	0.1	0	0	0	0	0.2	0.1	90.3	0	0	0	0.1	34.8	0.1	0	0	0	0	0	0	99.9	99.9	0	0	0	0	0	0	
G	0	0	0.2	100	0	0	0	0	99.9	99.9	0.1	0	0.1	99.9	0	0	0	18.3	0	99.9	0	0	0.1	0.1	0.2	0	0	0	0.1	99.9	0.2	100	99.9	
T	0	0	0	0	99.9	99.9	99.9	0	0	0	99.8	99.9	8.9	0	99.9	0	99.8	39.1	99.9	0	99.9	0	0	0	0	0.1	0	0	0	0	0	0	0	

88. A TRAC Ex.3 SA + Cas9 mRNA 1.5 µg

	A_{-10}	A_{-9}	A_{-8}	G_{-7}	T_{-6}	T_{-5}	T_{-4}	A_{-3}	G_{-2}	G_{-1}	T_1	T_2	C_3	G_4	T_5	A_6	T_7	C_8	T_9	G_{10}	T_{11}	A_{12}	A_{13}	A_{14}	A_{15}	C_{16}	C_{17}	A_{18}	A_{19}	G_{20}	A_{21}	G_{22}	G_{23}	Indel
A	99.8	99.4	99.6	0	0	0	0	99.9	0	0	0	0.1	0	0.1	0	99.9	0.1	0.2	0	0.1	0.1	99.6	99.3	99.4	99.1	0.3	0.4	99.4	98.9	0.1	99.4	0	0.1	94.9
C	0.1	0.5	0.1	0	0.1	0.2	0	0.1	0	0	0.3	0.1	99.9	0.1	0	0	0.3	99.7	0.2	0	0.5	0	0.3	0.1	0.6	99.6	99.2	0.1	0.1	0.2	0.1	0	0.4	
G	0	0	0.3	100	0	0	0	0	99.9	100	0.1	0	0	99.6	0.1	0.1	0	0	0	99.8	0.2	0.3	0.2	0.4	0.2	0	0.2	0.2	0.8	99.7	0.3	99.8	99.5	
T	0	0	0	0	99.9	99.8	100	0	0.1	0	99.6	99.8	0.1	0.1	99.9	0	99.6	0.1	99.8	0.1	99.2	0.1	0.2	0.1	0.1	0	0.2	0.3	0.1	0.1	0.2	0.1	0	

89. A TRAC Ex.3 SA + Pulse

	A_{-10}	A_{-9}	A_{-8}	G_{-7}	T_{-6}	T_{-5}	T_{-4}	A_{-3}	G_{-2}	G_{-1}	T_1	T_2	C_3	G_4	T_5	A_6	T_7	C_8	T_9	G_{10}	T_{11}	A_{12}	A_{13}	A_{14}	A_{15}	C_{16}	C_{17}	A_{18}	A_{19}	G_{20}	A_{21}	G_{22}	G_{23}	Indel
A	99.8	99.8	99.7	0	0	0	0	99.9	0	0	0	0	0	0	0	100	0	0	0	0	0	100	99.8	99.8	99.8	0	0	99.9	99.8	0	99.7	0	0	0
C	0.2	0.1	0.1	0	0.1	0.1	0	0	0	0	0.2	0.1	99.9	0	0	0	0.1	99.9	0.1	0	0	0	0.1	0	0	100	100	0	0	0	0	0	0	
G	0	0	0.3	100	0	0	0	0.1	100	100	0	0	0	99.9	0	0	0	0.1	0	100	0	0	0.2	0.1	0.2	0	0	0	0.1	99.9	0.3	100	99.9	
T	0	0	0	0	99.9	99.9	100	0	0	0	99.8	99.9	0	0	100	0	99.9	0	99.8	0	99.9	0	0	0	0	0	0	0	0	0	0	0	0	

90. D TRAC Ex.3 SA + Pulse

	A_{-10}	A_{-9}	A_{-8}	G_{-7}	T_{-6}	T_{-5}	T_{-4}	A_{-3}	G_{-2}	G_{-1}	T_1	T_2	C_3	G_4	T_5	A_6	T_7	C_8	T_9	G_{10}	T_{11}	A_{12}	A_{13}	A_{14}	A_{15}	C_{16}	C_{17}	A_{18}	A_{19}	G_{20}	A_{21}	G_{22}	G_{23}	Indel
A	99.8	99.8	99.7	0	0	0	0	99.9	0	0	0	0	0	0	0	99.9	0	0	0	0	0	100	99.8	99.8	99.8	0	0	99.9	99.8	0	99.7	0	0.1	0
C	0.1	0.1	0	0	0	0.1	0	0	0	0	0.2	0.1	99.9	0	0.1	0	0.1	100	0.1	0	0	0	0	0	0	100	99.9	0	0	0	0	0	0	
G	0	0	0.2	100	0	0	0	0.1	100	100	0	0	0	100	0	0	0	0	0	100	0	0	0.1	0.1	0.2	0	0	0	0.1	99.9	0.2	100	99.9	
T	0	0	0	0	99.9	99.9	100	0	0	0	99.7	99.9	0	0	99.9	0	99.8	0	99.9	0	99.9	0	0	0	0	0	0	0	0	0	0	0	0	

91. A TRAC Ex.3 pmSTOP_1 + BE3 unconc. mRNA 1.5 µg

	G_{-10}	A_{-9}	A_{-8}	C_{-7}	C_{-6}	T_{-5}	A_{-4}	A_{-3}	A_{-2}	C_{-1}	T_1	T_2	T_3	C_4	A_5	A_6	A_7	A_8	C_9	C_{10}	T_{11}	G_{12}	T_{13}	C_{14}	A_{15}	G_{16}	T_{17}	G_{18}	A_{19}	T_{20}	T_{21}	G_{22}	G_{23}	Indel
A	0.4	99.9	99.7	0	0	0	99.9	99.9	99.9	0	0	0	0	0.3	99.9	99.9	99.8	99.6	0	0	0	0	0.1	0.2	99.8	0.1	0	0	99.8	0	0	0.1	0	5.8
C	0	0	0.1	99.9	99.9	0.1	0	0	0.1	99.8	0.2	0.1	0	76	0	0	0	0.2	99.8	99.8	0.1	0.1	0.2	98.7	0	0	0.1	0	0	0.1	0	0	0	
G	99.5	0.1	0.2	0	0	0	0	0.1	0.1	0	0.1	0.2	0.1	4.7	0.1	0.1	0.1	0.2	0	0	0	99.9	0	0.1	0.1	99.9	0.2	99.9	0.2	0	0	99.9	99.9	
T	0	0	0	0.1	0	99.9	0	0	0	0.1	99.7	99.8	99.9	19.1	0	0	0	0.1	0.2	0.2	99.8	0	99.7	1.1	0	0	99.7	0	0	99.9	99.9	0	0	

92. B TRAC Ex.3 pmSTOP_1 + BE3 unconc. mRNA 1.5 µg

	G_{-10}	A_{-9}	A_{-8}	C_{-7}	C_{-6}	T_{-5}	A_{-4}	A_{-3}	A_{-2}	C_{-1}	T_1	T_2	T_3	C_4	A_5	A_6	A_7	A_8	C_9	C_{10}	T_{11}	G_{12}	T_{13}	C_{14}	A_{15}	G_{16}	T_{17}	G_{18}	A_{19}	T_{20}	T_{21}	G_{22}	G_{23}	Indel
A	0.2	99.9	99.6	0	0	0	99.9	100	99.8	0	0	0	0	0.2	100	99.8	99.9	99.5	0	0	0	0	0.1	0.1	99.9	0	0	0	100	0	0	0	0	4.8
C	0	0	0	99.9	99.9	0.1	0	0	0	99.8	0.4	0	0	76.4	0	0	0	0.2	99.8	99.9	0.1	0	0.2	99.1	0	0	0	0	0	0.1	0	0	0	
G	99.8	0.1	0.3	0	0	0	0.1	0	0.1	0	0	0.3	0.2	6.7	0	0.2	0.1	0.3	0.1	0	0	100	0	0.2	0	100	0	99.9	0	0	0	100	99.9	
T	0	0	0	0.1	0	99.9	0	0	0	0.1	99.5	99.7	99.8	16.6	0	0	0	0	0.1	0.1	99.8	0	99.6	0.6	0	0	100	0	0	99.9	100	0	0	

93. C TRAC Ex.3 pmSTOP_1 + BE3 unconc. mRNA 1.5 µg

	G_{-10}	A_{-9}	A_{-8}	C_{-7}	C_{-6}	T_{-5}	A_{-4}	A_{-3}	A_{-2}	C_{-1}	T_1	T_2	T_3	C_4	A_5	A_6	A_7	A_8	C_9	C_{10}	T_{11}	G_{12}	T_{13}	C_{14}	A_{15}	G_{16}	T_{17}	G_{18}	A_{19}	T_{20}	T_{21}	G_{22}	G_{23}	Indel
A	0.4	99.9	99.8	0	0	0	100	99.9	99.9	0	0	0	0	0.3	100	99.9	99.9	99.6	0	0	0	0	0	0.1	99.9	0	0	0	99.8	0	0	0	0	2.2
C	0	0	0	99.8	99.9	0	0	0	0	99.8	0.2	0	0	69.4	0	0	0	0.2	99.7	99.8	0.1	0	0.2	98.4	0	0	0.1	0	0	0.1	0	0	0	
G	99.5	0.1	0.2	0	0	0	0	0.1	0.1	0	0.1	0.1	0.2	5.9	0	0	0.1	0.2	0	0	0	99.9	0	0.1	0.1	100	0.1	100	0.1	0	0	99.9	100	
T	0	0	0	0.2	0.1	99.9	0	0	0	0.1	99.7	99.8	99.8	24.4	0	0	0	0	0.2	0.2	99.8	0	99.8	1.4	0	0	99.9	0	0	99.9	99.9	0	0	

94. A TRAC Ex.3 pmSTOP_1 + BE4 unconc. mRNA 1.5 µg

	G_{-10}	A_{-9}	A_{-8}	C_{-7}	C_{-6}	T_{-5}	A_{-4}	A_{-3}	A_{-2}	C_{-1}	T_1	T_2	T_3	C_4	A_5	A_6	A_7	A_8	C_9	C_{10}	T_{11}	G_{12}	T_{13}	C_{14}	A_{15}	G_{16}	T_{17}	G_{18}	A_{19}	T_{20}	T_{21}	G_{22}	G_{23}	Indel
A	0.5	99.9	99.6	0	0	0	99.9	99.9	99.9	0	0	0	0	0.2	99.9	99.9	99.8	99.4	0	0	0	0	0	0.1	99.8	0	0	0	99.8	0	0	0.1	0	2.1
C	0	0	0.2	99.8	99.9	0.1	0	0	0	99.8	0.2	0	0	80.2	0	0	0.2	0.4	99.6	99.8	0.1	0	0.2	99.2	0	0	0.1	0	0.1	0.1	0.1	0	0	
G	99.4	0.1	0.2	0	0	0	0	0.1	0.1	0	0.1	0.2	0.2	2.1	0	0	0.1	0.2	0	0	0.1	99.9	0	0.1	0.1	99.9	0.1	99.9	0.1	0	0	99.9	99.9	
T	0	0	0	0.1	0	99.9	0	0	0	0.2	99.8	99.7	99.8	17.6	0	0	0	0	0.3	0.2	99.8	0	99.8	0.6	0	0	99.8	0	0	99.9	99.9	0	0	

95. B TRAC Ex.3 pmSTOP_1 + BE4 unconc. mRNA 1.5 µg

	G_{-10}	A_{-9}	A_{-8}	C_{-7}	C_{-6}	T_{-5}	A_{-4}	A_{-3}	A_{-2}	C_{-1}	T_1	T_2	T_3	C_4	A_5	A_6	A_7	A_8	C_9	C_{10}	T_{11}	G_{12}	T_{13}	C_{14}	A_{15}	G_{16}	T_{17}	G_{18}	A_{19}	T_{20}	T_{21}	G_{22}	G_{23}	Indel
A	0.4	100	99.8	0	0	0	100	99.9	99.9	0	0	0	0	0.2	99.9	99.9	99.9	99.2	0	0	0	0.1	0	0.1	99.8	0	0.1	0.1	99.9	0	0	0.1	0	2
C	0	0	0.1	99.9	99.9	0.1	0	0.1	0	99.6	0.2	0	0.1	67.6	0	0	0.1	0.4	99.4	99.7	0.1	0	0.3	98.9	0	0	0.1	0	0	0.1	0.1	0	0	
G	99.5	0	0.2	0	0	0	0	0.1	0.1	0	0.1	0.1	0	2.5	0.1	0.1	0.1	0.4	0	0	0	99.9	0	0	0.2	99.9	0	99.9	0	0	0	99.9	100	
T	0.1	0	0	0.1	0.1	99.9	0	0	0	0.4	99.7	99.9	99.9	29.6	0	0	0	0	0.6	0.3	99.9	0.1	99.7	1	0	0.1	99.8	0	0.1	99.9	99.9	0	0	

96. C TRAC Ex.3 pmSTOP_1 + BE4 unconc. mRNA 1.5 µg

	G_{-10}	A_{-9}	A_{-8}	C_{-7}	C_{-6}	T_{-5}	A_{-4}	A_{-3}	A_{-2}	C_{-1}	T_1	T_2	T_3	C_4	A_5	A_6	A_7	A_8	C_9	C_{10}	T_{11}	G_{12}	T_{13}	C_{14}	A_{15}	G_{16}	T_{17}	G_{18}	A_{19}	T_{20}	T_{21}	G_{22}	G_{23}	Indel
A	0.9	99.9	99.8	0	0	0	100	99.9	99.9	0	0	0	0	0.2	100	99.9	99.9	99.6	0	0	0	0	0	0	99.9	0	0	0	99.8	0	0	0	0	1
C	0	0	0.1	99.8	99.9	0	0	0	0	99.8	0.2	0	0	71.6	0	0	0	0.2	99.5	99.7	0.2	0	0.2	99.1	0	0	0	0	0	0.1	0.1	0	0	
G	99	0.1	0.2	0	0	0	0	0.1	0.1	0	0.1	0.1	0.1	2.3	0	0	0.1	0.2	0	0	0	100	0	0	0	100	0	100	0.1	0	0	100	100	
T	0	0	0	0.2	0.1	99.9	0	0	0	0.2	99.7	99.8	99.9	25.9	0	0	0	0	0.4	0.3	99.8	0	99.8	0.9	0	0	99.9	0	0	99.9	99.9	0	0	

97. D TRAC Ex.3 pmSTOP_1 + Pulse

	G_{-10}	A_{-9}	A_{-8}	C_{-7}	C_{-6}	T_{-5}	A_{-4}	A_{-3}	A_{-2}	C_{-1}	T_1	T_2	T_3	C_4	A_5	A_6	A_7	A_8	C_9	C_{10}	T_{11}	G_{12}	T_{13}	C_{14}	A_{15}	G_{16}	T_{17}	G_{18}	A_{19}	T_{20}	T_{21}	G_{22}	G_{23}	Indel
A	0	99.9	99.7	0	0	0	100	99.9	99.9	0	0	0	0	0	100	99.9	99.9	99.6	0	0	0	0	0	0	99.9	0	0	0	99.8	0	0	0	0	0
C	0	0	0	100	100	0.1	0	0	0	100	0.2	0	0	100	0	0	0	0.1	100	100	0.1	0	0.2	100	0	0	0.1	0	0	0.1	0.1	0	0	
G	99.9	0.1	0.2	0	0	0	0	0.1	0	0	0	0.1	0.1	0	0	0	0.1	0.2	0	0	0	100	0	0	0.1	100	0	100	0.1	0	0	100	100	
T	0	0	0	0	0	99.9	0	0	0	0	99.7	99.8	99.8	0	0	0	0	0	0	0	99.8	0	99.8	0	0	0	99.9	0	0	99.9	99.9	0	0	

98. A TRAC Ex.3 pmSTOP_1 + Pulse

	G_{-10}	A_{-9}	A_{-8}	C_{-7}	C_{-6}	T_{-5}	A_{-4}	A_{-3}	A_{-2}	C_{-1}	T_1	T_2	T_3	C_4	A_5	A_6	A_7	A_8	C_9	C_{10}	T_{11}	G_{12}	T_{13}	C_{14}	A_{15}	G_{16}	T_{17}	G_{18}	A_{19}	T_{20}	T_{21}	G_{22}	G_{23}	Indel
A	0	99.9	99.8	0	0	0	100	99.9	99.9	0	0	0	0	0	100	100	99.9	99.6	0	0	0	0	0	0	99.8	0	0	0	99.8	0	0	0	0	0
C	0	0	0	100	100	0.1	0	0	0	100	0.3	0	0	100	0	0	0	0.2	100	100	0.2	0	0.2	99.9	0	0	0.1	0	0	0.1	0.1	0	0	
G	99.9	0.1	0.2	0	0	0	0	0.1	0.1	0	0.1	0.1	0.2	0	0	0	0	0.2	0	0	0	100	0	0	0.1	100	0	100	0.1	0	0	100	100	
T	0	0	0	0	0	99.9	0	0	0	0	99.7	99.8	99.8	0	0	0	0	0	0	0	99.8	0	99.8	0.1	0	0	99.9	0	0	99.9	99.9	0	0	

99. A TRAC Ex.3 pmSTOP_2 + BE3 unconc. mRNA 1.5 µg

	A_{-10}	A_{-9}	C_{-8}	C_{-7}	T_{-6}	A_{-5}	A_{-4}	A_{-3}	C_{-2}	T_{-1}	T_{1}	T_{2}	C_{3}	A_{4}	A_{5}	A_{6}	A_{7}	C_{8}	C_{9}	T_{10}	G_{11}	T_{12}	C_{13}	A_{14}	G_{15}	T_{16}	G_{17}	A_{18}	T_{19}	T_{20}	G_{21}	G_{22}	G_{23}	Indel
A	99.9	99.7	0	0	0	100	99.8	99.9	0.1	0	0	0.1	0.8	99.8	99.8	99.8	99.6	0.5	0.1	0	0	0.1	0.4	99.8	0.1	0	0	99.7	0.1	0	0.1	0	0.1	9
C	0	0.1	99.7	99.9	0.1	0	0.1	0.1	99.7	0.2	0.1	0.1	70.4	0	0	0.1	0.2	95.8	99.1	0.1	0	0.2	97.3	0.1	0.1	0.1	0	0.1	0.1	0.1	0	0	0	
G	0.1	0.2	0.1	0	0	0	0.1	0.1	0	0.1	0.2	0.1	7.8	0.1	0.1	0.1	0.2	0.7	0.1	0.1	99.9	0	0.1	0.1	99.8	0.2	99.9	0.2	0	0	99.9	100	99.9	
T	0	0	0.2	0	99.8	0	0	0	0.2	99.7	99.7	99.8	21	0	0	0	0	3	0.7	99.8	0	99.8	2.2	0.1	0	99.7	0	0.1	99.8	99.8	0	0	0	

100. B TRAC Ex.3 pmSTOP_2 + BE3 unconc. mRNA 1.5 µg

	A_{-10}	A_{-9}	C_{-8}	C_{-7}	T_{-6}	A_{-5}	A_{-4}	A_{-3}	C_{-2}	T_{-1}	T_{1}	T_{2}	C_{3}	A_{4}	A_{5}	A_{6}	A_{7}	C_{8}	C_{9}	T_{10}	G_{11}	T_{12}	C_{13}	A_{14}	G_{15}	T_{16}	G_{17}	A_{18}	T_{19}	T_{20}	G_{21}	G_{22}	G_{23}	Indel
A	99.9	99.7	0	0	0	99.9	99.9	99.9	0	0	0	0	1	99.9	99.9	99.8	99.5	0.6	0.1	0	0	0.1	0.2	99.9	0	0	0	99.8	0	0	0	0.1	0	7.3
C	0	0	99.8	99.9	0.1	0	0	0	99.8	0.2	0.1	0.1	74.9	0	0	0.1	0.2	95.7	99.4	0.2	0	0.2	97.7	0	0	0.1	0	0	0.1	0.1	0	0	0	
G	0.1	0.2	0	0	0	0	0.1	0	0	0.1	0.1	0.1	8.4	0	0.1	0.1	0.3	1	0.1	0	99.9	0	0.2	0	99.9	0.1	100	0.1	0	0	99.9	99.9	99.9	
T	0	0	0.1	0	99.9	0	0	0	0.1	99.7	99.8	99.8	15.8	0	0	0	0	2.7	0.4	99.8	0.1	99.7	1.9	0	0	99.8	0	0	99.9	99.9	0	0	0	

101. C TRAC Ex.3 pmSTOP_2 + BE3 unconc. mRNA 1.5 µg

	A_{-10}	A_{-9}	C_{-8}	C_{-7}	T_{-6}	A_{-5}	A_{-4}	A_{-3}	C_{-2}	T_{-1}	T_{1}	T_{2}	C_{3}	A_{4}	A_{5}	A_{6}	A_{7}	C_{8}	C_{9}	T_{10}	G_{11}	T_{12}	C_{13}	A_{14}	G_{15}	T_{16}	G_{17}	A_{18}	T_{19}	T_{20}	G_{21}	G_{22}	G_{23}	Indel
A	99.9	99.8	0	0	0	100	99.9	99.9	0.1	0	0	0	0.8	99.9	99.9	99.9	99.6	0.4	0.1	0	0	0	0.3	99.9	0	0	0	99.8	0	0	0.1	0	0.1	5
C	0	0	99.8	99.9	0	0	0	0	99.8	0.2	0	0.1	69.7	0	0	0	0.2	95.4	99.1	0.1	0	0.2	97.4	0	0	0.1	0	0	0.1	0	0	0	0	
G	0.1	0.2	0	0	0	0	0.1	0.1	0	0.1	0.1	0.1	9.1	0.1	0.1	0	0.2	0.8	0.1	0	99.9	0	0.2	0.1	100	0.1	100	0.1	0	0	99.9	100	99.9	
T	0	0	0.1	0	99.9	0	0	0	0.1	99.7	99.8	99.8	20.4	0	0	0	0	3.3	0.7	99.8	0.1	99.8	2.1	0	0	99.8	0	0.1	99.9	99.9	0	0	0	

102. A TRAC Ex.3 pmSTOP_2 + BE4 unconc. mRNA 1.5 µg

	A_{-10}	A_{-9}	C_{-8}	C_{-7}	T_{-6}	A_{-5}	A_{-4}	A_{-3}	C_{-2}	T_{-1}	T_{1}	T_{2}	C_{3}	A_{4}	A_{5}	A_{6}	A_{7}	C_{8}	C_{9}	T_{10}	G_{11}	T_{12}	C_{13}	A_{14}	G_{15}	T_{16}	G_{17}	A_{18}	T_{19}	T_{20}	G_{21}	G_{22}	G_{23}	Indel
A	99.9	99.8	0	0	0	100	99.9	99.9	0.1	0	0	0.1	0.6	99.9	99.9	99.9	99.7	0.6	0.1	0	0	0	0.2	99.8	0	0	0	99.8	0	0	0.1	0	0	5.7
C	0	0	99.7	99.9	0.1	0	0	0	99.6	0.2	0	0	65.2	0	0	0	0.1	91.7	96.5	0.2	0	0.2	96.7	0	0	0.1	0	0	0.1	0.1	0	0	0	
G	0.1	0.1	0	0	0	0	0.1	0.1	0	0.1	0.2	0.1	3.4	0.1	0.1	0.1	0.2	0.6	0.1	0	100	0	0.2	0.1	99.9	0.1	100	0.2	0	0	99.9	99.9	99.9	
T	0	0	0.3	0.1	99.8	0	0	0	0.3	99.7	99.8	99.8	30.8	0	0	0	0	7.2	3.3	99.8	0	99.8	2.9	0.1	0	99.8	0	0	99.9	99.9	0	0	0	

103. B TRAC Ex.3 pmSTOP_2 + BE4 unconc. mRNA 1.5 µg

	A_{-10}	A_{-9}	C_{-8}	C_{-7}	T_{-6}	A_{-5}	A_{-4}	A_{-3}	C_{-2}	T_{-1}	T_{1}	T_{2}	C_{3}	A_{4}	A_{5}	A_{6}	A_{7}	C_{8}	C_{9}	T_{10}	G_{11}	T_{12}	C_{13}	A_{14}	G_{15}	T_{16}	G_{17}	A_{18}	T_{19}	T_{20}	G_{21}	G_{22}	G_{23}	Indel
A	99.8	99.8	0	0	0	100	99.8	99.8	0	0	0	0	0.6	100	99.9	99.8	99.5	0.5	0.2	0	0	0	0.3	99.9	0	0	0	99.8	0	0	0	0	0	3.5
C	0	0.1	99.6	99.6	0	0	0	0.1	99.6	0.2	0	0	66.5	0	0	0	0.2	92.8	97.1	0.2	0	0.2	97.5	0	0	0.1	0	0	0.1	0.1	0	0	0	
G	0.1	0.2	0	0.1	0	0	0.2	0.1	0	0.1	0.1	0.1	4	0	0	0.1	0.3	0.9	0.1	0	99.9	0	0.1	0.1	99.9	0	99.9	0.2	0	0	100	100	99.9	
T	0	0	0.4	0.3	99.9	0	0	0	0.4	99.7	99.8	99.8	29	0	0	0	0.1	5.9	2.6	99.8	0	99.8	2.1	0	0	99.9	0	0	99.9	99.9	0	0	0	

104. C TRAC Ex.3 pmSTOP_2 + BE4 unconc. mRNA 1.5 µg

	A_{-10}	A_{-9}	C_{-8}	C_{-7}	T_{-6}	A_{-5}	A_{-4}	A_{-3}	C_{-2}	T_{-1}	T_{1}	T_{2}	C_{3}	A_{4}	A_{5}	A_{6}	A_{7}	C_{8}	C_{9}	T_{10}	G_{11}	T_{12}	C_{13}	A_{14}	G_{15}	T_{16}	G_{17}	A_{18}	T_{19}	T_{20}	G_{21}	G_{22}	G_{23}	Indel
A	99.9	99.7	0	0	0	100	99.9	99.9	0	0	0	0	0.5	99.9	99.9	99.9	99.5	0.4	0.2	0	0	0	0.2	99.9	0	0	0	99.8	0	0	0	0	0	2.1
C	0	0.1	99.6	99.9	0	0	0	0	99.6	0.2	0	0	65.8	0	0	0	0.2	91.6	96.5	0.1	0	0.2	97.1	0	0	0.1	0	0	0.1	0.1	0	0	0	
G	0.1	0.2	0	0	0	0	0.1	0.1	0	0.1	0.2	0.2	3.7	0	0	0.1	0.2	0.8	0.1	0	100	0	0.1	0	100	0	100	0.1	0	0	100	100	100	
T	0	0	0.3	0.1	99.9	0	0	0	0.4	99.7	99.8	99.8	29.9	0	0	0	0	7.2	3.2	99.8	0	99.7	2.6	0	0	99.9	0	0	99.9	99.9	0	0	0	

105. D TRAC Ex.3 pmSTOP_2 + Pulse

	A_{-10}	A_{-9}	C_{-8}	C_{-7}	T_{-6}	A_{-5}	A_{-4}	A_{-3}	C_{-2}	T_{-1}	T_{1}	T_{2}	C_{3}	A_{4}	A_{5}	A_{6}	A_{7}	C_{8}	C_{9}	T_{10}	G_{11}	T_{12}	C_{13}	A_{14}	G_{15}	T_{16}	G_{17}	A_{18}	T_{19}	T_{20}	G_{21}	G_{22}	G_{23}	Indel
A	99.9	99.7	0	0	0	100	99.9	99.9	0	0	0	0	0	100	99.9	99.9	99.6	0	0	0	0	0	0	99.9	0	0	0	99.8	0	0	0	0	0	0
C	0	0	100	100	0.1	0	0	0	100	0.2	0	0	100	0	0	0	0.1	100	100	0.1	0	0.2	100	0	0	0.1	0	0	0.1	0.1	0	0	0	
G	0.1	0.2	0	0	0	0	0.1	0	0	0	0.1	0.1	0	0	0	0.1	0.2	0	0	0	100	0	0	0.1	100	0	100	0.1	0	0	100	100	100	
T	0	0	0	0	99.9	0	0	0	0	99.7	99.8	99.8	0	0	0	0	0	0	0	99.8	0	99.8	0	0	0	99.9	0	0	99.9	99.9	0	0	0	

106. A TRAC Ex.3 pmSTOP_2 + Pulse

	A_{-10}	A_{-9}	C_{-8}	C_{-7}	T_{-6}	A_{-5}	A_{-4}	A_{-3}	C_{-2}	T_{-1}	T_{1}	T_{2}	C_{3}	A_{4}	A_{5}	A_{6}	A_{7}	C_{8}	C_{9}	T_{10}	G_{11}	T_{12}	C_{13}	A_{14}	G_{15}	T_{16}	G_{17}	A_{18}	T_{19}	T_{20}	G_{21}	G_{22}	G_{23}	Indel
A	99.9	99.8	0	0	0	100	99.9	99.9	0	0	0	0	0	100	100	99.9	99.6	0	0	0	0	0	0	99.8	0	0	0	99.8	0	0	0	0	0	0
C	0	0	100	100	0.1	0	0	0	100	0.3	0	0	100	0	0	0	0.2	100	100	0.2	0	0.2	99.9	0	0	0.1	0	0	0.1	0.1	0	0	0	
G	0.1	0.2	0	0	0	0	0.1	0.1	0	0.1	0.1	0.2	0	0	0	0	0.2	0	0	0	100	0	0	0.1	100	0	100	0.1	0	0	100	100	100	
T	0	0	0	0	99.9	0	0	0	0	99.7	99.8	99.8	0	0	0	0	0	0	0	99.8	0	99.8	0.1	0	0	99.9	0	0	99.9	99.9	0	0	0	

107. A B2M Ex.1 SD + BE3 unconc. mRNA 1.5 μg

	G_{-10}	G_{-9}	T_{-8}	A_{-7}	G_{-6}	G_{-5}	A_{-4}	G_{-3}	A_{-2}	G_{-1}	A_1	C_2	T_3	C_4	A_5	C_6	G_7	C_8	T_9	G_{10}	G_{11}	A_{12}	T_{13}	A_{14}	G_{15}	C_{16}	C_{17}	T_{18}	C_{19}	C_{20}	A_{21}	G_{22}	G_{23}	Indel
A	0.1	0.1	1.9	96.9	0.2	1	92.2	0.6	93.9	0.4	95.3	0.6	0.7	3	97.8	13.8	0.3	0.4	0.1	0.3	0.2	96.9	0.8	96.7	0.4	0.4	0.4	0.9	2.7	0.3	99.7	0.1	0.1	21.4
C	0	0	2.2	1	0.1	0.2	1.4	0.2	1.7	0.3	1.1	98.1	1.7	42.8	0.9	28.6	0.5	96.9	0.6	0.4	0.3	1.7	1	1.4	0.3	98.9	98.6	2.4	96.4	99.5	0.2	0	0	
G	99.9	99.8	12.7	2.1	99.6	98.7	6.3	99	4.4	99.1	3.5	1	4.4	10.2	1.1	9	99	1.8	3.4	99	99.3	1.3	4.3	1.7	98.8	0.6	0.9	3.5	0.4	0.1	0.1	99.9	99.9	
T	0	0.1	83.2	0	0.1	0.2	0.1	0.1	0.1	0.2	0.1	0.4	93.2	44	0.2	48.5	0.2	0.8	95.9	0.3	0.2	0.2	93.9	0.2	0.5	0.1	0.1	93.2	0.5	0.1	0	0	0	

108. B B2M Ex.1 SD + BE3 unconc. mRNA 1.5 μg

	G_{-10}	G_{-9}	T_{-8}	A_{-7}	G_{-6}	G_{-5}	A_{-4}	G_{-3}	A_{-2}	G_{-1}	A_1	C_2	T_3	C_4	A_5	C_6	G_7	C_8	T_9	G_{10}	G_{11}	A_{12}	T_{13}	A_{14}	G_{15}	C_{16}	C_{17}	T_{18}	C_{19}	C_{20}	A_{21}	G_{22}	G_{23}	Indel
A	0.1	0.1	0	99.8	0.3	1.1	99.8	0.6	99.8	0.3	99.7	0.3	0.1	3.1	99.3	16.2	0	0.2	0	0.2	0.1	99.7	0	99.9	0	0	0	0	0.1	0	99.8	0	0	16.8
C	0	0	0.1	0	0	0.2	0	0.2	0	0.2	0	99	0.2	39.5	0.2	24.1	0	98.5	0.1	0.1	0.1	0.2	0.1	0	0	99.9	99.9	0.1	99.1	99.9	0	0	0	
G	99.9	99.9	0	0.1	99.6	98.5	0.2	99.2	0.1	99.5	0.2	0.2	0	8.5	0.1	6.6	99.8	0.3	0.1	99.6	99.8	0.1	0.1	0.1	99.8	0	0	0.1	0.2	0	0.1	100	99.9	
T	0	0.1	99.8	0	0.1	0.1	0	0.1	0	0	0.1	0.5	99.7	48.9	0.4	53.1	0.1	1	99.8	0.1	0	0	99.7	0	0.1	0	0	99.8	0.6	0.1	0	0	0	

109. C B2M Ex.1 SD + BE3 unconc. mRNA 1.5 μg

	G_{-10}	G_{-9}	T_{-8}	A_{-7}	G_{-6}	G_{-5}	A_{-4}	G_{-3}	A_{-2}	G_{-1}	A_1	C_2	T_3	C_4	A_5	C_6	G_7	C_8	T_9	G_{10}	G_{11}	A_{12}	T_{13}	A_{14}	G_{15}	C_{16}	C_{17}	T_{18}	C_{19}	C_{20}	A_{21}	G_{22}	G_{23}	Indel
A	0	0	0	99.9	0	0	99.8	0	99.8	0	99.8	0	0	0	99.9	0	0	0	0	0	0.1	99.9	0	99.9	0	0	0	0	0	0	99.9	0	0	0
C	0	0	0.1	0	0	0	0	0	0	0	0	100	0	100	0	100	0	100	0.1	0	0	0	0	0	0	100	100	0.1	99.9	99.9	0	0	0	
G	100	99.9	0	0.1	100	100	0.2	100	0.1	100	0.1	0	0	0	0.1	0	100	0	0	100	99.9	0.1	0	0.1	100	0	0	0.1	0	0	0.1	100	100	
T	0	0	99.9	0	0	0	0	0	0	0	0	0	99.9	0	0	0	0	0	99.8	0	0	0	99.9	0	0	0	0	99.8	0	0	0	0	0	

110. A B2M Ex.1 SD + BE4 unconc. mRNA 1.5 μg

	G_{-10}	G_{-9}	T_{-8}	A_{-7}	G_{-6}	G_{-5}	A_{-4}	G_{-3}	A_{-2}	G_{-1}	A_1	C_2	T_3	C_4	A_5	C_6	G_7	C_8	T_9	G_{10}	G_{11}	A_{12}	T_{13}	A_{14}	G_{15}	C_{16}	C_{17}	T_{18}	C_{19}	C_{20}	A_{21}	G_{22}	G_{23}	Indel
A	0.1	0.1	1.8	97.1	0.8	3	92.3	2	94	1.2	95.5	0.4	0.6	1.9	98	8.2	0.1	0.3	0.1	0.1	0.8	97.5	0.6	97.5	0.2	0.3	0.3	0.5	2.6	0.1	99.8	0	0.1	14.3
C	0	0.1	2.2	0.8	0	0.3	1.4	0.4	1.6	0.4	1	97.8	1.5	33.2	0.8	24.3	0.4	96.5	0.3	0.2	0.2	1.2	0.7	1	0.2	99.2	98.9	1.9	96.2	99.7	0.1	0	0	
G	99.8	99.8	12.9	2	99	96.6	6.3	97.5	4.3	98.2	3.5	1.2	4	8.6	1.1	7.2	99.3	1.6	2.9	99.5	98.9	1.2	4.1	1.4	99.3	0.3	0.8	3.1	0.1	0	0.1	100	99.9	
T	0	0.1	83	0	0.1	0.1	0	0.1	0.1	0.2	0	0.6	93.9	56.3	0.2	60.2	0.2	1.6	96.7	0.2	0.1	0.1	94.7	0.2	0.3	0.1	0	94.5	1	0.1	0	0	0	

111. B B2M Ex.1 SD + BE4 unconc. mRNA 1.5 μg

	G_{-10}	G_{-9}	T_{-8}	A_{-7}	G_{-6}	G_{-5}	A_{-4}	G_{-3}	A_{-2}	G_{-1}	A_1	C_2	T_3	C_4	A_5	C_6	G_7	C_8	T_9	G_{10}	G_{11}	A_{12}	T_{13}	A_{14}	G_{15}	C_{16}	C_{17}	T_{18}	C_{19}	C_{20}	A_{21}	G_{22}	G_{23}	Indel
A	0.2	0.1	0	99.9	1.8	6.1	99.8	4	99.8	2.1	99.8	0.3	0	1.7	99.7	7.5	0	0.2	0	0.1	0.1	99.9	0	99.9	0	0	0	0	0.1	0	99.9	0	0	9.3
C	0	0	0.1	0	0	0.2	0	0.3	0	0.3	0	97.9	0.1	24.7	0	17.8	0	96.8	0.1	0	0	0.1	0.1	0	0	99.9	99.9	0.1	98.1	99.8	0	0	0	
G	99.8	99.8	0	0.1	98.1	93.6	0.2	95.7	0.1	97.5	0.1	0.4	0	4.6	0.1	2.8	99.9	0.3	0.1	99.8	99.8	0.1	0.1	0.1	99.9	0	0	0.1	0.1	0	0.1	100	100	
T	0	0	99.9	0	0.1	0.1	0	0.1	0	0.1	0	1.4	99.8	69	0.1	72	0.1	2.8	99.8	0	0	0	99.8	0	0	0.1	0.1	99.8	1.7	0.1	0	0	0	

112. C B2M Ex.1 SD + BE4 unconc. mRNA 1.5 μg

	G_{-10}	G_{-9}	T_{-8}	A_{-7}	G_{-6}	G_{-5}	A_{-4}	G_{-3}	A_{-2}	G_{-1}	A_1	C_2	T_3	C_4	A_5	C_6	G_7	C_8	T_9	G_{10}	G_{11}	A_{12}	T_{13}	A_{14}	G_{15}	C_{16}	C_{17}	T_{18}	C_{19}	C_{20}	A_{21}	G_{22}	G_{23}	Indel
A	0	0	0	99.9	0	0	99.7	0	99.8	0	99.8	0	0	0.1	99.9	0	0	0	0	0	0.1	99.9	0	99.9	0	0	0.1	0.1	0.1	0.1	99.9	0	0	0
C	0	0	0.1	0	0	0	0	0	0	0	0	100	0	99.9	0	99.9	0	100	0.1	0	0	0	0.1	0	0	99.9	99.9	0.1	99.9	99.9	0	0	0	
G	100	100	0.1	0.1	100	100	0.2	100	0.1	100	0.2	0	0.1	0	0.1	0	100	0	0.2	100	99.9	0.1	0.1	0.1	100	0	0	0.4	0	0	0.1	100	99.9	
T	0	0	99.8	0	0	0	0	0	0	0	0	0	99.8	0	0	0	0	0	99.8	0	0	0	99.8	0	0	0	0	99.4	0	0	0	0	0	

113. D B2M Ex.1 SD + BE4 unconc. mRNA 1.5 μg

	G_{-10}	G_{-9}	T_{-8}	A_{-7}	G_{-6}	G_{-5}	A_{-4}	G_{-3}	A_{-2}	G_{-1}	A_1	C_2	T_3	C_4	A_5	C_6	G_7	C_8	T_9	G_{10}	G_{11}	A_{12}	T_{13}	A_{14}	G_{15}	C_{16}	C_{17}	T_{18}	C_{19}	C_{20}	A_{21}	G_{22}	G_{23}	Indel
A	0.1	0.1	0	99.8	1.6	4.6	99.5	3	99.5	1.8	99.7	0.4	0.1	1.9	99.6	9.2	0	0.2	0	0.1	0.1	99.6	0.1	99.8	0	0	0.1	0.2	0.1	0	99.8	1	0	8.7
C	0	0	0.1	0	0	0.2	0.1	0.3	0.1	0.3	0.1	98.3	0.2	29.2	0.1	19.9	0	97.4	0.2	0	0.1	0.1	0.1	0	0	99.9	99.8	0.6	98.5	99.8	0	0	0	
G	99.8	99.8	0.4	0.1	98.3	95.1	0.4	96.6	0.3	97.8	0.2	0.2	0.3	5.5	0.1	3.7	99.9	0.3	0.3	99.8	99.8	0.2	0.3	0.1	99.9	0	0	0.4	0.1	0	0.2	98.9	99.9	
T	0.1	0.1	99.4	0	0.1	0.1	0	0.1	0	0.2	0	1	99.4	63.5	0.2	67.2	0.1	2.2	99.5	0.1	0.1	0.1	99.5	0	0.1	0	0.1	98.8	1.2	0.1	0	0.1	0	

114. A B2M Ex.1 SD + Pulse

	G_{-10}	G_{-9}	T_{-8}	A_{-7}	G_{-6}	G_{-5}	A_{-4}	G_{-3}	A_{-2}	G_{-1}	A_1	C_2	T_3	C_4	A_5	C_6	G_7	C_8	T_9	G_{10}	G_{11}	A_{12}	T_{13}	A_{14}	G_{15}	C_{16}	C_{17}	T_{18}	C_{19}	C_{20}	A_{21}	G_{22}	G_{23}	Indel
A	0.1	0	0.1	99.8	0	0	98.7	0.1	98.8	0.1	99.5	0.3	0.2	1.1	99.4	0.3	0.1	0.3	0.1	0.1	0.2	99.2	0.3	99.5	0.1	0.3	0.4	0.9	0.6	0.3	99.8	0.8	0.1	0
C	0	0	0.1	0.1	0	0	0.4	0	0.4	0.1	0.2	99.7	0.4	98.7	0.4	99.6	0	99.5	0.2	0	0	0.2	0.2	0.2	0	99.6	99.5	2.8	99.3	99.7	0.1	0	0	
G	99.8	99.9	1.6	0.1	99.9	99.8	0.9	99.7	0.7	99.4	0.2	0	1.8	0.1	0.1	0	99.7	0.2	1.6	99.7	99.5	0.2	1.3	0.3	99.8	0.1	0.1	2	0.1	0	0.1	98.8	99.4	
T	0.2	0.1	98.2	0	0	0.1	0.1	0.2	0.1	0.4	0	0	97.7	0.1	0	0.1	0.2	0.1	98	0.2	0.3	0.4	98.2	0	0.1	0	0	94.3	0	0	0	0.3	0.4	

115. A B2M Ex.2 pmSTOP + BE3 unconc. mRNA 1.5 µg

	G_{-10}	A_{-9}	A_{-8}	T_{-7}	G_{-6}	T_{-5}	A_{-4}	A_{-3}	G_{-2}	A_{-1}	C_1	T_2	T_3	A_4	C_5	C_6	C_7	C_8	A_9	C_{10}	T_{11}	T_{12}	A_{13}	A_{14}	C_{15}	T_{16}	A_{17}	T_{18}	C_{19}	T_{20}	T_{21}	G_{22}	G_{23}	Indel
A	1.2	99.3	99.5	0.2	0.1	0.1	99.4	99	0.2	98.9	1	0.5	9.9	99.3	15.6	4.3	8.7	1.5	99.2	0.4	0.2	0.2	99.2	99.6	0.6	0.1	98.8	0.6	0.7	0.3	0.2	0.2	0.1	27.9
C	0.1	0.4	0.3	0	0	0.1	0.4	0.7	0.2	0.8	98.7	0.2	0.5	0.3	35.7	36.6	54.6	80.6	0.6	98.4	0.2	0.2	0.7	0.2	99	0.2	1	0.4	98.8	0.2	0.2	0.1	0.2	
G	98.5	0.2	0.1	0.1	99.8	0	0.1	0.3	99.1	0.2	0.1	0.1	0.1	0.1	0.8	1.8	3.5	0.1	0.1	0.4	0.2	0.2	0.1	0.1	0.1	0.1	0.1	0.2	0.1	0.2	0.2	99.3	99.5	
T	0.2	0.1	0	99.7	0.1	99.8	0.1	0.1	0.4	0.1	0.2	99.2	89.5	0.4	48	57.3	33.3	17.8	0.1	0.8	99.4	99.4	0.1	0.1	0.2	99.6	0.1	98.7	0.3	99.3	99.5	0.4	0.2	

116. C B2M Ex.2 pmSTOP + BE3 unconc. mRNA 1.5 µg

	G_{-10}	A_{-9}	A_{-8}	T_{-7}	G_{-6}	T_{-5}	A_{-4}	A_{-3}	G_{-2}	A_{-1}	C_1	T_2	T_3	A_4	C_5	C_6	C_7	C_8	A_9	C_{10}	T_{11}	T_{12}	A_{13}	A_{14}	C_{15}	T_{16}	A_{17}	T_{18}	C_{19}	T_{20}	T_{21}	G_{22}	G_{23}	Indel
A	0.1	99.8	99.9	0	0.1	0	99.9	99.8	0.2	99.8	0.5	0.4	10	99.6	15.2	3.7	8.1	0.7	99.9	0.1	0	0	99.9	99.9	0	0	99.9	0	0	0	0	0	0	28.9
C	0.1	0	0	0	0	0.1	0	0	0.1	0	99.3	0.1	0.1	0	35.7	36.3	55.4	81.3	0	98.7	0.1	0	0	0	99.8	0.1	0	0.1	99.7	0.1	0	0	0	
G	99.8	0.1	0.1	0	99.9	0	0.1	0.1	99.7	0.2	0.1	0	0	0	0.8	1.6	3.3	0.1	0	0.3	0	0	0	0.1	0	0	0	0	0.1	0	0	99.9	99.9	
T	0	0	0	99.9	0	99.9	0	0.1	0	0	0.2	99.5	89.9	0.3	48.4	58.4	33.3	17.9	0	0.9	99.9	99.9	0	0	0.1	99.9	0	99.9	0.2	99.9	100	0	0	

117. A B2M Ex.2 pmSTOP + BE4 unconc. mRNA 1.5 µg

	G_{-10}	A_{-9}	A_{-8}	T_{-7}	G_{-6}	T_{-5}	A_{-4}	A_{-3}	G_{-2}	A_{-1}	C_1	T_2	T_3	A_4	C_5	C_6	C_7	C_8	A_9	C_{10}	T_{11}	T_{12}	A_{13}	A_{14}	C_{15}	T_{16}	A_{17}	T_{18}	C_{19}	T_{20}	T_{21}	G_{22}	G_{23}	Indel
A	1.2	99.4	99.5	0.1	0.1	0.1	99.5	99.2	0.4	99.1	0.6	0.3	5.2	99.4	8.6	2.8	5	0.9	99.5	0.4	0.1	0.1	99.5	99.7	0.5	0.1	99.2	0.4	0.5	0.2	0.1	0.2	0.1	19.2
C	0.1	0.3	0.3	0	0	0.1	0.4	0.5	0.2	0.6	98.9	0.2	0.2	0.2	35.3	32.3	45.2	61.1	0.4	97.9	0.1	0.1	0.4	0.2	99.2	0.2	0.7	0.4	99	0.2	0.1	0	0.1	
G	98.5	0.2	0.1	0.1	99.8	0	0.1	0.2	99.1	0.2	0.1	0	0.1	0.1	0.3	1.1	2.2	0.1	0.1	0.3	0.1	0.1	0.1	0.1	0.1	0	0	0.2	0.1	0.1	0.1	99.4	99.6	
T	0.2	0.1	0.1	99.8	0.1	99.8	0	0	0.3	0.1	0.4	99.5	94.5	0.3	55.8	63.8	47.7	37.9	0.1	1.4	99.7	99.7	0.1	0	0.2	99.7	0	99.1	0.4	99.5	99.7	0.4	0.2	

118. C B2M Ex.2 pmSTOP + BE4 unconc. mRNA 1.5 µg

	G_{-10}	A_{-9}	A_{-8}	T_{-7}	G_{-6}	T_{-5}	A_{-4}	A_{-3}	G_{-2}	A_{-1}	C_1	T_2	T_3	A_4	C_5	C_6	C_7	C_8	A_9	C_{10}	T_{11}	T_{12}	A_{13}	A_{14}	C_{15}	T_{16}	A_{17}	T_{18}	C_{19}	T_{20}	T_{21}	G_{22}	G_{23}	Indel
A	0.2	99.8	99.8	0	0.1	0	99.9	99.8	0.2	99.8	0.4	0.3	5.4	99.7	8.2	2.4	4.3	0.6	99.9	0.1	0	0	99.9	99.9	0	0	99.9	0	0.1	0	0	0	0	18.8
C	0	0	0	0	0	0.1	0	0	0.1	0	99.2	0.1	0	0	34.9	32.5	46	61.9	0	98.2	0.1	0	0	0	99.8	0.1	0	0.1	99.5	0.1	0	0	0	
G	99.7	0.1	0.1	0	99.9	0	0	0.1	99.7	0.1	0.1	0	0	0	0.5	1.1	1.8	0.1	0	0.3	0	0	0	0.1	0	0	0	0	0.1	0	0	99.9	99.9	
T	0.1	0	0	99.9	0	99.9	0	0	0	0	0.4	99.6	94.6	0.2	56.3	64.1	47.9	37.4	0	1.4	99.9	100	0	0	0.2	99.9	0	99.9	0.4	99.9	100	0.1	0	

119. A B2M Ex.2 pmSTOP + Pulse

	G_{-10}	A_{-9}	A_{-8}	T_{-7}	G_{-6}	T_{-5}	A_{-4}	A_{-3}	G_{-2}	A_{-1}	C_1	T_2	T_3	A_4	C_5	C_6	C_7	C_8	A_9	C_{10}	T_{11}	T_{12}	A_{13}	A_{14}	C_{15}	T_{16}	A_{17}	T_{18}	C_{19}	T_{20}	T_{21}	G_{22}	G_{23}	Indel
A	1	99.4	99.5	0.1	0	0.1	99.6	99.1	0.1	99.3	0.3	0	0	99.6	0.2	1	1.1	0.8	99.1	0.3	0.2	0.1	99.4	99.7	0.6	0.1	99.1	0.5	0.5	0.2	0.1	0.2	0.1	0
C	0	0.4	0.3	0	0	0.1	0.3	0.6	0	0.5	99.6	0.1	0.1	0.2	99.6	98.7	98.5	98.8	0.7	99.7	0.2	0.2	0.5	0.2	99.3	0.2	0.8	0.5	99.3	0.2	0.2	0	0.1	
G	98.8	0.2	0.1	0.1	99.8	0.1	0	0.3	99.4	0.2	0	0	0	0.1	0	0.1	0.1	0	0.1	0	0.2	0.2	0	0.1	0	0.1	0	0.2	0	0.2	0.2	99.5	99.6	
T	0.1	0.1	0	99.8	0.1	99.8	0	0	0.4	0	0.1	99.8	99.8	0	0.1	0.2	0.3	0.4	0.1	0	99.5	99.5	0	0	0.1	99.6	0	98.8	0.1	99.4	99.6	0.3	0.2	

120. A B2M Ex.3 SA + BE3 unconc. mRNA 1.5 µg

	C_{-10}	T_{-9}	T_{-8}	A_{-7}	C_{-6}	A_{-5}	T_{-4}	G_{-3}	T_{-2}	C_{-1}	T_1	C_2	G_3	A_4	T_5	C_6	T_7	A_8	T_9	G_{10}	A_{11}	A_{12}	A_{13}	A_{14}	A_{15}	G_{16}	A_{17}	C_{18}	A_{19}	G_{20}	T_{21}	G_{22}	G_{23}	Indel
A	0.1	0	0	99.8	0.2	99.9	0.1	0.1	0.1	0.5	0	0.5	0.1	99.9	0	10.8	0.1	99.9	0	0.1	99.7	99.9	99.8	99.9	99.7	0.1	99.8	0.1	99.8	0	0	0.1	0.1	3.9
C	99.8	0.2	0.1	0	99.6	0	0.2	0	0.2	92.9	0.2	95.1	0	0	0.1	19.5	0.1	0	0.2	0	0	0	0	0	0	0	0	99.9	0	0	0.1	0	0	
G	0	0.1	0	0.1	0	0.1	0.1	99.9	0.1	0.3	0	0.1	99.8	0	0	38.2	0	0.1	0	99.9	0.2	0	0.2	0	0.2	99.8	0.2	0	0.1	99.9	0.1	99.9	99.9	
T	0.1	99.7	99.8	0	0.2	0	99.7	0.1	99.6	6.2	99.7	4.3	0	0	99.8	31.4	99.8	0	99.7	0.1	0	0	0	0	0	0	0	0	0	0	99.8	0	0	

121. B B2M Ex.3 SA + BE3 unconc. mRNA 1.5 µg

	C_{-10}	T_{-9}	T_{-8}	A_{-7}	C_{-6}	A_{-5}	T_{-4}	G_{-3}	T_{-2}	C_{-1}	T_1	C_2	G_3	A_4	T_5	C_6	T_7	A_8	T_9	G_{10}	A_{11}	A_{12}	A_{13}	A_{14}	A_{15}	G_{16}	A_{17}	C_{18}	A_{19}	G_{20}	T_{21}	G_{22}	G_{23}	Indel
A	0	0	0	99.9	0.1	99.9	0	0.1	0	0.4	0	0.4	0.1	99.9	0.1	11.8	0.1	99.9	0	0	99.8	99.9	99.8	99.9	99.7	0	99.8	0.1	99.8	0	0.1	0	0	3.8
C	99.9	0.2	0.1	0	99.8	0	0.1	0	0.2	93.3	0.1	95.5	0.1	0	0.1	25.1	0.1	0	0.1	0	0	0	0	0	0	0	0	99.9	0	0	0.1	0	0	
G	0	0	0	0.1	0	0	0	99.8	0	0.5	0	0	99.8	0	0	35.3	0	0.1	0	99.9	0.1	0	0.2	0	0.3	99.9	0.2	0	0.1	99.9	0	99.9	99.9	
T	0	99.8	99.9	0	0.1	0	99.8	0.1	99.8	5.8	99.8	4.1	0.1	0.1	99.8	27.8	99.8	0	99.8	0	0	0	0	0	0	0	0	0	0	0	99.8	0	0	

123. A B2M Ex.3 SA + BE4 unconc. mRNA 1.5 µg

	C_{-10}	T_{-9}	T_{-8}	A_{-7}	C_{-6}	A_{-5}	T_{-4}	G_{-3}	T_{-2}	C_{-1}	T_1	C_2	G_3	A_4	T_5	C_6	T_7	A_8	T_9	G_{10}	A_{11}	A_{12}	A_{13}	A_{14}	A_{15}	G_{16}	A_{17}	C_{18}	A_{19}	G_{20}	T_{21}	G_{22}	G_{23}	Indel
A	0.3	0.1	0	99.9	0.5	99.8	0.1	0.1	0.1	0.5	0.2	0.7	0.4	99.9	0.1	4.9	0.1	99.9	0.1	0.2	99.6	99.9	99.8	99.9	99.7	0.2	99.7	0.2	99.8	0.1	0.1	0.2	0.3	2.8
C	99.5	0.2	0.1	0	99.2	0.1	0.1	0	0.2	91.1	0.2	91.1	0	0	0.1	19.7	0.1	0	0.1	0	0.2	0	0	0	0.1	0	0	99.8	0	0	0.1	0	0	
G	0.1	0.2	0.1	0.1	0	0.1	0.2	99.7	0.2	0.4	0.2	0	99.5	0	0.1	15.7	0.1	0.1	0.1	99.8	0.2	0.1	0.2	0	0.2	99.8	0.2	0	0.2	99.9	0.1	99.7	99.6	
T	0.1	99.6	99.8	0	0.4	0	99.5	0.2	99.5	8	99.5	8.2	0	0	99.6	59.7	99.7	0	99.7	0	0	0	0	0	0	0	0	0	0	0	99.6	0	0.1	

124. B B2M Ex.3 SA + BE4 unconc. mRNA 1.5 µg

	C_{-10}	T_{-9}	T_{-8}	A_{-7}	C_{-6}	A_{-5}	T_{-4}	G_{-3}	T_{-2}	C_{-1}	T_1	C_2	G_3	A_4	T_5	C_6	T_7	A_8	T_9	G_{10}	A_{11}	A_{12}	A_{13}	A_{14}	A_{15}	G_{16}	A_{17}	C_{18}	A_{19}	G_{20}	T_{21}	G_{22}	G_{23}	Indel
A	0	0	0	99.9	0.1	99.9	0	0.1	0	0.4	0	0.4	0.1	99.9	0.1	6.8	0	99.9	0	0.1	99.8	99.9	99.8	100	99.7	0.1	99.8	0.1	99.8	0	0.1	0	0.1	2.6
C	99.9	0.1	0.1	0	99.5	0	0.1	0	0.2	91.4	0.2	91.6	0.1	0	0.1	24	0.1	0	0.1	0	0	0	0	0	0	0	0	99.8	0	0	0.1	0	0	
G	0	0	0	0.1	0	0	0	99.8	0	0.3	0	0	99.8	0	0	18.9	0	0.1	0	99.9	0.1	0	0.1	0	0.2	99.8	0.2	0	0.1	100	0	99.9	99.8	
T	0	99.8	99.9	0	0.4	0	99.8	0.1	99.8	7.9	99.8	7.9	0.1	0	99.8	50.4	99.9	0	99.8	0	0	0	0	0	0	0	0	0.1	0	0	99.8	0	0	

125. C B2M Ex.3 SA + BE4 unconc. mRNA 1.5 µg

	C_{-10}	T_{-9}	T_{-8}	A_{-7}	C_{-6}	A_{-5}	T_{-4}	G_{-3}	T_{-2}	C_{-1}	T_1	C_2	G_3	A_4	T_5	C_6	T_7	A_8	T_9	G_{10}	A_{11}	A_{12}	A_{13}	A_{14}	A_{15}	G_{16}	A_{17}	C_{18}	A_{19}	G_{20}	T_{21}	G_{22}	G_{23}	Indel
A	0	0	0	99.9	0.2	99.9	0	0.1	0	0.4	0	0.4	0.1	99.9	0.1	6.2	0	99.9	0	0.1	99.8	99.9	99.8	99.9	99.8	0.2	99.8	0.2	99.8	0	0.1	0	0.1	2.6
C	99.9	0.1	0.1	0	99.5	0	0.2	0	0.2	92.3	0.1	91.7	0.1	0	0.1	26.1	0.1	0	0.1	0	0	0	0	0	0	0	0	99.8	0	0	0.1	0	0	
G	0	0	0	0.1	0	0	0	99.8	0	0.3	0	0.1	99.8	0	0	16.8	0	0	0	99.8	0.1	0	0.2	0	0.2	99.7	0.2	0	0.1	99.9	0	99.8	99.8	
T	0	99.8	99.9	0	0.3	0	99.8	0.1	99.7	7.1	99.8	7.8	0.1	0	99.8	51	99.9	0	99.8	0.1	0	0	0	0	0	0.1	0	0	0	0	99.8	0.1	0.1	

126. A 3G TRAC Ex.3 SA + Pulse

	A_{-10}	A_{-9}	A_{-8}	G_{-7}	T_{-6}	T_{-5}	T_{-4}	A_{-3}	G_{-2}	G_{-1}	T_1	T_2	C_3	G_4	T_5	A_6	T_7	C_8	T_9	G_{10}	T_{11}	A_{12}	A_{13}	A_{14}	A_{15}	C_{16}	C_{17}	A_{18}	A_{19}	G_{20}	A_{21}	G_{22}	G_{23}	Indel
A	99.9	99.9	99.8	0	0	0	0	99.9	0	0.1	0	0	0	0.1	0	99.9	0	0	0	0	0	99.9	99.8	99.8	99.7	0	0	99.9	99.8	0	99.7	0	0.1	0.4
C	0	0	0	0	0.1	0.1	0	0	0	0	0.2	0.1	99.9	0	0.1	0	0.1	99.9	0.1	0	0.1	0	0	0	0	100	100	0	0	0	0	0	0	
G	0	0.1	0.2	100	0.1	0.1	0	0.1	99.9	99.9	0	0	0	99.9	0	0.1	0	0	0	99.9	0	0	0.2	0.1	0.2	0	0	0.1	0.1	99.9	0.2	99.9	99.8	
T	0	0	0	0	99.9	99.8	99.9	0	0	0	99.8	99.8	0.1	0	99.9	0	99.8	0	99.8	0	99.9	0	0	0	0	0	0	0	0	0	0	0	0	

127. A 3G TRAC Ex.3 SA + Pulse

	A_{-10}	A_{-9}	A_{-8}	G_{-7}	T_{-6}	T_{-5}	T_{-4}	A_{-3}	G_{-2}	G_{-1}	T_1	T_2	C_3	G_4	T_5	A_6	T_7	C_8	T_9	G_{10}	T_{11}	A_{12}	A_{13}	A_{14}	A_{15}	C_{16}	C_{17}	A_{18}	A_{19}	G_{20}	A_{21}	G_{22}	G_{23}	Indel
A	99.9	99.9	99.7	0	0	0	0	99.9	0	0	0	0	0	0	0	99.9	0	0	0	0	0	100	99.8	99.8	99.7	0	0	99.9	99.8	0	99.7	0	0	0.1
C	0	0	0	0	0.1	0.2	0	0	0	0	0.2	0.1	99.9	0	0.1	0	0.2	99.8	0.2	0	0.1	0	0	0	0	100	100	0	0	0	0	0	0	
G	0.1	0	0.3	100	0	0	0	0.1	100	100	0	0	0	99.9	0	0.1	0	0	0	100	0	0	0.2	0.1	0.3	0	0	0.1	0.2	100	0.3	100	99.9	
T	0	0	0	0	99.9	99.8	100	0	0	0	99.8	99.8	0.1	0	99.9	0	99.8	0.2	99.8	0	99.9	0	0	0	0	0	0	0	0	0	0	0	0	

128. B 3G TRAC Ex.3 SA + Pulse

	A_{-10}	A_{-9}	A_{-8}	G_{-7}	T_{-6}	T_{-5}	T_{-4}	A_{-3}	G_{-2}	G_{-1}	T_1	T_2	C_3	G_4	T_5	A_6	T_7	C_8	T_9	G_{10}	T_{11}	A_{12}	A_{13}	A_{14}	A_{15}	C_{16}	C_{17}	A_{18}	A_{19}	G_{20}	A_{21}	G_{22}	G_{23}	Indel
A	99.9	99.9	99.8	0	0	0	0	99.9	0	0	0	0	0	0	0	99.9	0	0	0	0	0	100	99.9	99.9	99.7	0	0	99.9	99.8	0	99.7	0	0	0
C	0	0	0	0	0.1	0.1	0	0	0	0	0.2	0.1	99.9	0	0.1	0	0.1	99.8	0.1	0	0	0	0	0	0	100	100	0	0	0	0	0	0	
G	0	0	0.2	99.9	0.1	0.1	0	0.1	99.9	99.9	0	0	0	99.9	0	0	0.1	0	0	100	0	0	0.1	0.1	0.2	0	0	0.1	0.2	99.9	0.2	99.9	99.9	
T	0	0	0	0	99.9	99.8	99.9	0	0	0	99.8	99.8	0.1	0	99.9	0	99.8	0.1	99.8	0	99.9	0	0	0	0	0	0	0	0	0	0	0.1	0.1	

129. C 3G TRAC Ex.3 SA + Pulse

	A_{-10}	A_{-9}	A_{-8}	G_{-7}	T_{-6}	T_{-5}	T_{-4}	A_{-3}	G_{-2}	G_{-1}	T_1	T_2	C_3	G_4	T_5	A_6	T_7	C_8	T_9	G_{10}	T_{11}	A_{12}	A_{13}	A_{14}	A_{15}	C_{16}	C_{17}	A_{18}	A_{19}	G_{20}	A_{21}	G_{22}	G_{23}	Indel
A	99.9	99.9	99.7	0	0	0	0	99.9	0	0	0	0	0	0.1	0	99.9	0	0	0	0	0	99.9	99.8	99.8	99.7	0	0	99.9	99.8	0	99.7	0	0	0.1
C	0	0	0	0	0.1	0.1	0.1	0	0	0	0.2	0.2	99.8	0	0.1	0	0.2	99.7	0.2	0	0.1	0	0	0	0	100	100	0	0	0	0	0	0	
G	0.1	0.1	0.3	100	0	0	0	0.1	100	99.9	0	0	0	99.9	0	0	0	0	0	100	0	0.1	0.2	0.1	0.2	0	0	0.1	0.2	100	0.3	100	99.9	
T	0	0	0	0	99.9	99.9	99.9	0	0	0	99.8	99.8	0.1	0	99.9	0	99.8	0.3	99.8	0	99.9	0	0	0	0	0	0	0	0	0	0	0	0	

130. A 3G TRAC Ex.3 SA + Pulse

	A_{-10}	A_{-9}	A_{-8}	G_{-7}	T_{-6}	T_{-5}	T_{-4}	A_{-3}	G_{-2}	G_{-1}	T_1	T_2	C_3	G_4	T_5	A_6	T_7	C_8	T_9	G_{10}	T_{11}	A_{12}	A_{13}	A_{14}	A_{15}	C_{16}	C_{17}	A_{18}	A_{19}	G_{20}	A_{21}	G_{22}	G_{23}	Indel
A	99.9	99.9	99.7	0	0	0	0	99.9	0	0.1	0	0	0	0	0	99.9	0	0	0	0	0	100	99.8	99.8	99.7	0	0	99.9	99.8	0	99.7	0	0.1	0.2
C	0	0	0	0	0.1	0.1	0	0	0	0	0.2	0.1	99.9	0	0.1	0	0.2	99.8	0.2	0	0.1	0	0	0	0	100	99.9	0	0	0	0	0	0	
G	0.1	0.1	0.2	100	0	0	0	0.1	100	99.9	0	0	0	99.9	0	0.1	0	0	0	100	0	0	0.2	0.1	0.3	0	0	0.1	0.2	100	0.3	100	99.9	
T	0	0	0	0	99.9	99.8	99.9	0	0	0	99.8	99.8	0.1	0	99.9	0	99.8	0.1	99.8	0	99.9	0	0	0	0	0	0	0	0	0	0	0	0	

131. B 3G TRAC Ex.3 SA + Pulse

	A_{-10}	A_{-9}	A_{-8}	G_{-7}	T_{-6}	T_{-5}	T_{-4}	A_{-3}	G_{-2}	G_{-1}	T_1	T_2	C_3	G_4	T_5	A_6	T_7	C_8	T_9	G_{10}	T_{11}	A_{12}	A_{13}	A_{14}	A_{15}	C_{16}	C_{17}	A_{18}	A_{19}	G_{20}	A_{21}	G_{22}	G_{23}	Indel
A	99.9	99.9	99.7	0	0	0	0	99.9	0	0.1	0	0	0	0	0	99.9	0	0	0	0	0	100	99.8	99.8	99.7	0	0	99.9	99.8	0	99.7	0	0.1	0.4
C	0	0	0	0	0.1	0.1	0.1	0	0	0	0.2	0.2	99.6	0	0.1	0	0.2	98.9	0.2	0	0.1	0	0	0	0	100	100	0	0	0	0	0	0	
G	0.1	0.1	0.3	100	0	0	0	0.1	100	99.9	0	0	0	99.9	0	0.1	0	0	0	99.9	0	0	0.2	0.2	0.2	0	0	0.1	0.2	100	0.3	100	99.9	
T	0	0	0	0	99.9	99.8	99.9	0	0	0	99.8	99.8	0.4	0	99.8	0	99.8	1.1	99.8	0	99.9	0	0	0	0	0	0	0	0	0	0	0	0	

132. A 3G TRAC Ex.3 SA + Pulse

	A_{-10}	A_{-9}	A_{-8}	G_{-7}	T_{-6}	T_{-5}	T_{-4}	A_{-3}	G_{-2}	G_{-1}	T_1	T_2	C_3	G_4	T_5	A_6	T_7	C_8	T_9	G_{10}	T_{11}	A_{12}	A_{13}	A_{14}	A_{15}	C_{16}	C_{17}	A_{18}	A_{19}	G_{20}	A_{21}	G_{22}	G_{23}	Indel
A	99.9	99.9	99.7	0	0	0	0	99.9	0	0	0	0	0	0.1	0	99.9	0	0	0	0	0	99.9	99.8	99.8	99.7	0	0	99.9	99.7	0	99.7	0	0.1	0.6
C	0	0	0	0	0.1	0.1	0.1	0	0	0	0.2	0.2	99.7	0	0.1	0	0.2	98.8	0.2	0	0.1	0	0	0	0	100	99.9	0	0	0	0	0	0	
G	0.1	0.1	0.2	100	0	0	0	0.1	100	99.9	0	0	0	99.9	0	0.1	0	0	0	100	0	0	0.2	0.1	0.3	0	0	0.1	0.2	100	0.3	100	99.9	
T	0	0	0	0	99.9	99.8	99.9	0	0	0	99.8	99.8	0.3	0	99.9	0	99.8	1.1	99.8	0	99.9	0	0	0	0	0	0	0	0	0	0	0	0	

133. B 3G TRAC Ex.3 SA + Pulse

	A_{-10}	A_{-9}	A_{-8}	G_{-7}	T_{-6}	T_{-5}	T_{-4}	A_{-3}	G_{-2}	G_{-1}	T_1	T_2	C_3	G_4	T_5	A_6	T_7	C_8	T_9	G_{10}	T_{11}	A_{12}	A_{13}	A_{14}	A_{15}	C_{16}	C_{17}	A_{18}	A_{19}	G_{20}	A_{21}	G_{22}	G_{23}	Indel
A	99.9	99.9	99.7	0	0	0	0	99.9	0	0	0	0	0	0.1	0	99.9	0	0	0	0	0	99.9	99.8	99.8	99.7	0	0	99.9	99.7	0	99.7	0	0	0.4
C	0	0	0	0	0.1	0.1	0	0	0	0	0.2	0.1	99.8	0	0.1	0	0.2	99.6	0.2	0	0	0	0	0	0	100	100	0	0	0	0	0	0	
G	0.1	0.1	0.3	100	0	0	0	0.1	100	100	0	0	0	99.9	0	0.1	0	0	0	100	0	0.1	0.2	0.2	0.3	0	0	0.1	0.2	100	0.3	99.9	99.9	
T	0	0	0	0	99.8	99.8	99.9	0	0	0	99.8	99.8	0.2	0	99.9	0	99.8	0.3	99.8	0	99.9	0	0	0	0	0	0	0	0	0	0	0	0	

134. A 3G TRAC Ex.3 SA + BE3 conc. mRNA 3 µg

	A_{-10}	A_{-9}	A_{-8}	G_{-7}	T_{-6}	T_{-5}	T_{-4}	A_{-3}	G_{-2}	G_{-1}	T_1	T_2	C_3	G_4	T_5	A_6	T_7	C_8	T_9	G_{10}	T_{11}	A_{12}	A_{13}	A_{14}	A_{15}	C_{16}	C_{17}	A_{18}	A_{19}	G_{20}	A_{21}	G_{22}	G_{23}	Indel
A	99.9	99.9	99.7	0	0	0	0	99.9	0	0	0	0	0.9	0.1	0	99.8	0.1	5.1	0	0.1	0	100	99.8	99.8	99.7	0.1	0	99.9	99.8	0	99.7	0	0.1	2.9
C	0	0	0	0	0.1	0.1	0	0	0	0	0.3	0.1	93.8	0	0.1	0	0.1	59.5	0.2	0	0	0	0	0	0	99.8	99.8	0	0	0	0	0	0	
G	0.1	0	0.3	99.9	0.1	0.1	0.1	0.1	99.9	99.9	0	0.1	0.1	99.9	0	0	0.1	14.4	0.1	99.9	0	0	0.2	0.1	0.2	0	0	0.1	0.2	100	0.3	99.9	99.9	
T	0	0	0	0.1	99.8	99.8	99.8	0	0	0	99.6	99.8	5.3	0	99.9	0.1	99.7	20.9	99.8	0	99.9	0	0	0	0	0.1	0.1	0	0	0	0	0	0.1	

135. B 3G TRAC Ex.3 SA + BE3 conc. mRNA 3 µg

	A_{-10}	A_{-9}	A_{-8}	G_{-7}	T_{-6}	T_{-5}	T_{-4}	A_{-3}	G_{-2}	G_{-1}	T_1	T_2	C_3	G_4	T_5	A_6	T_7	C_8	T_9	G_{10}	T_{11}	A_{12}	A_{13}	A_{14}	A_{15}	C_{16}	C_{17}	A_{18}	A_{19}	G_{20}	A_{21}	G_{22}	G_{23}	Indel
A	99.9	99.9	99.7	0	0	0	0	99.8	0	0	0	0	0.9	0.1	0	99.9	0.1	6.5	0	0	0	99.9	99.8	99.8	99.7	0	0	99.9	99.8	0	99.7	0	0	2.9
C	0	0	0	0	0.1	0.1	0.1	0	0	0	0.2	0.2	92	0	0.1	0	0.2	47.9	0.2	0	0.1	0	0	0	0	99.9	100	0	0	0	0	0	0	
G	0.1	0.1	0.3	100	0	0	0	0.2	100	99.9	0	0	0.2	99.9	0	0.1	0	16.4	0	99.9	0	0.1	0.2	0.2	0.3	0	0	0.1	0.2	100	0.3	100	99.9	
T	0	0	0	0	99.8	99.8	99.9	0	0	0	99.7	99.8	6.9	0	99.8	0	99.7	29.2	99.8	0	99.9	0	0	0	0	0.1	0	0	0	0	0	0	0	

136. A 3G TRAC Ex.3 SA + BE4 conc. mRNA 3 µg

	A_{-10}	A_{-9}	A_{-8}	G_{-7}	T_{-6}	T_{-5}	T_{-4}	A_{-3}	G_{-2}	G_{-1}	T_1	T_2	C_3	G_4	T_5	A_6	T_7	C_8	T_9	G_{10}	T_{11}	A_{12}	A_{13}	A_{14}	A_{15}	C_{16}	C_{17}	A_{18}	A_{19}	G_{20}	A_{21}	G_{22}	G_{23}	Indel
A	99.9	99.9	99.7	0	0	0	0	99.9	0	0	0	0	0.3	0.1	0	99.9	0.1	4	0	0	0	99.9	99.8	99.8	99.7	0	0	99.9	99.8	0.1	99.7	0	0.1	2.1
C	0	0	0	0	0.1	0.1	0	0	0	0	0.2	0.1	93	0	0.1	0	0.1	59.1	0.1	0	0	0	0	0	0	99.8	99.9	0	0	0	0	0	0	
G	0.1	0.1	0.2	99.9	0	0.1	0	0.1	99.9	100	0	0	0	99.9	0	0	0	8.7	0	100	0	0	0.2	0.1	0.2	0	0	0.1	0.2	99.9	0.2	99.9	99.9	
T	0	0	0	0	99.9	99.8	99.9	0	0.1	0	99.8	99.8	6.6	0	99.9	0	99.8	28.3	99.8	0	99.9	0	0	0	0	0.2	0	0	0	0	0	0	0.1	

137. B 3G TRAC Ex.3 SA + BE4 conc. mRNA 3 µg

	A_{-10}	A_{-9}	A_{-8}	G_{-7}	T_{-6}	T_{-5}	T_{-4}	A_{-3}	G_{-2}	G_{-1}	T_1	T_2	C_3	G_4	T_5	A_6	T_7	C_8	T_9	G_{10}	T_{11}	A_{12}	A_{13}	A_{14}	A_{15}	C_{16}	C_{17}	A_{18}	A_{19}	G_{20}	A_{21}	G_{22}	G_{23}	Indel
A	99.9	99.9	99.7	0.1	0	0	0	99.9	0	0.1	0	0	0.4	0.1	0	99.9	0.1	3.7	0	0	0	99.9	99.8	99.8	99.7	0	0	99.9	99.8	0	99.7	0	0.1	1.7
C	0	0	0	0	0.1	0.2	0	0	0	0	0.2	0.2	91.9	0	0.1	0	0.2	52.9	0.2	0	0.1	0	0	0	0	99.9	100	0	0	0	0	0	0	
G	0.1	0.1	0.3	99.9	0	0	0	0.1	100	99.9	0	0	0	99.9	0	0.1	0	10.4	0	99.9	0	0	0.2	0.1	0.2	0	0	0.1	0.2	100	0.3	100	99.9	
T	0	0	0	0	99.8	99.8	99.9	0	0	0	99.8	99.8	7.7	0	99.9	0	99.7	32.9	99.8	0	99.9	0	0	0	0	0.1	0	0	0	0	0	0	0	

138. A 3G TRAC Ex.3 SA + BE4 RNP

	A_{-10}	A_{-9}	A_{-8}	G_{-7}	T_{-6}	T_{-5}	T_{-4}	A_{-3}	G_{-2}	G_{-1}	T_1	T_2	C_3	G_4	T_5	A_6	T_7	C_8	T_9	G_{10}	T_{11}	A_{12}	A_{13}	A_{14}	A_{15}	C_{16}	C_{17}	A_{18}	A_{19}	G_{20}	A_{21}	G_{22}	G_{23}	Indel
A	99.9	99.9	99.7	0.1	0	0	0	99.8	0	0.1	0	0	0.4	0	0	99.9	0.1	3.6	0	0	0	100	99.8	99.9	99.7	0	0	99.9	99.8	0	99.7	0	0.1	1.5
C	0	0	0	0	0.1	0.1	0.1	0	0	0	0.2	0.2	75.9	0	0.1	0	0.1	12	0.1	0	0	0	0	0	0	99.7	99.9	0	0	0	0	0	0	
G	0	0.1	0.3	99.9	0	0	0	0.1	99.9	99.9	0	0	0	99.9	0	0	0	8.6	0	99.9	0	0	0.2	0.1	0.3	0	0	0.1	0.2	100	0.3	100	99.9	
T	0	0	0	0	99.8	99.8	99.9	0	0	0	99.8	99.8	23.6	0	99.9	0	99.8	75.8	99.8	0	99.9	0	0	0	0	0.3	0.1	0	0	0	0	0	0	

139. B 3G TRAC Ex.3 SA + BE4 RNP

	A_{-10}	A_{-9}	A_{-8}	G_{-7}	T_{-6}	T_{-5}	T_{-4}	A_{-3}	G_{-2}	G_{-1}	T_1	T_2	C_3	G_4	T_5	A_6	T_7	C_8	T_9	G_{10}	T_{11}	A_{12}	A_{13}	A_{14}	A_{15}	C_{16}	C_{17}	A_{18}	A_{19}	G_{20}	A_{21}	G_{22}	G_{23}	Indel
A	99.9	99.9	99.7	0	0	0	0	99.8	0.1	0.1	0	0	0.4	0	0	99.9	0.1	4.3	0	0	0	99.9	99.8	99.8	99.6	0	0	99.9	99.8	0.1	99.7	0	0	2.2
C	0	0	0	0	0.1	0.2	0.1	0	0	0	0.1	0.2	78.2	0	0.1	0	0.2	16.3	0.1	0	0.1	0	0	0	0	99.8	99.9	0	0	0	0	0	0	
G	0.1	0.1	0.3	99.9	0	0	0	0.1	99.9	99.9	0	0	0	99.9	0	0	0	10.2	0	99.9	0	0	0.2	0.1	0.3	0	0	0.1	0.2	99.9	0.3	100	99.9	
T	0	0	0	0	99.8	99.8	99.9	0	0	0	99.8	99.8	21.3	0	99.9	0	99.8	69.2	99.9	0	99.9	0	0	0	0	0.2	0.1	0	0	0	0	0	0	

140. A 3G TRAC Ex.3 SA + coBE4 mRNA 1.5 µg

	A_{-10}	A_{-9}	A_{-8}	G_{-7}	T_{-6}	T_{-5}	T_{-4}	A_{-3}	G_{-2}	G_{-1}	T_1	T_2	C_3	G_4	T_5	A_6	T_7	C_8	T_9	G_{10}	T_{11}	A_{12}	A_{13}	A_{14}	A_{15}	C_{16}	C_{17}	A_{18}	A_{19}	G_{20}	A_{21}	G_{22}	G_{23}	Indel
A	99.9	99.9	99.8	0	0	0	0	99.9	0	0.1	0	0	0	0.1	0	100	0	0.4	0	0	0	100	99.8	99.8	99.7	0	0	99.9	99.8	0	99.7	0	0	0.7
C	0	0	0	0	0.1	0.1	0.1	0	0	0	0.2	0.1	75.6	0	0.1	0	0.1	11.8	0.1	0	0.1	0	0	0	0	99.8	100	0	0	0	0	0	0	
G	0.1	0.1	0.2	99.9	0	0	0	0.1	100	99.9	0	0	0	99.9	0	0	0	1.1	0	100	0	0	0.2	0.2	0.3	0	0	0.1	0.2	100	0.3	100	99.9	
T	0	0	0	0	99.9	99.8	99.9	0	0	0	99.8	99.8	24.4	0	99.9	0	99.9	86.7	99.9	0	99.9	0	0	0	0	0.2	0	0	0	0	0	0	0	

141. B 3G TRAC Ex.3 SA + coBE4 mRNA 1.5 µg

	A_{-10}	A_{-9}	A_{-8}	G_{-7}	T_{-6}	T_{-5}	T_{-4}	A_{-3}	G_{-2}	G_{-1}	T_1	T_2	C_3	G_4	T_5	A_6	T_7	C_8	T_9	G_{10}	T_{11}	A_{12}	A_{13}	A_{14}	A_{15}	C_{16}	C_{17}	A_{18}	A_{19}	G_{20}	A_{21}	G_{22}	G_{23}	Indel
A	99.9	99.9	99.8	0	0	0	0	99.9	0	0.1	0	0	0	0	0	100	0	0.5	0	0	0	99.9	99.8	99.8	99.7	0	0	99.9	99.8	0	99.7	0	0.1	0.8
C	0	0	0	0	0.1	0.2	0	0	0	0	0.2	0.2	78	0	0.1	0	0.1	12.2	0.1	0	0	0	0	0	0	99.8	99.9	0	0	0	0	0	0	
G	0.1	0.1	0.2	100	0	0	0	0.1	100	99.9	0	0	0	99.9	0	0	0	1.8	0	99.9	0	0	0.2	0.2	0.2	0	0	0.1	0.2	99.9	0.3	100	99.9	
T	0	0	0	0	99.8	99.8	99.9	0	0	0	99.8	99.8	21.9	0	99.9	0	99.8	85.4	99.9	0	99.9	0	0	0	0	0.2	0	0	0	0	0	0	0	

142. A 3G TRAC Ex.3 SA + coBE4 mRNA 4 µg

	A_{-10}	A_{-9}	A_{-8}	G_{-7}	T_{-6}	T_{-5}	T_{-4}	A_{-3}	G_{-2}	G_{-1}	T_1	T_2	C_3	G_4	T_5	A_6	T_7	C_8	T_9	G_{10}	T_{11}	A_{12}	A_{13}	A_{14}	A_{15}	C_{16}	C_{17}	A_{18}	A_{19}	G_{20}	A_{21}	G_{22}	G_{23}	Indel
A	99.9	99.9	99.7	0	0	0	0	99.9	0	0.1	0	0	0	0	0	99.9	0	0.2	0	0	0	99.9	99.8	99.8	99.6	0	0	99.9	99.8	0	99.7	0	0	0.3
C	0	0	0	0	0.1	0.1	0.1	0	0	0	0.2	0.2	77	0	0.1	0	0.1	8.1	0.1	0	0.1	0	0	0	0	99.8	99.9	0	0	0	0	0	0	
G	0.1	0.1	0.3	100	0	0	0	0.1	99.9	99.9	0	0	0	99.9	0	0	0	0.4	0	100	0	0	0.2	0.1	0.3	0	0	0.1	0.2	100	0.2	100	99.9	
T	0	0	0	0	99.9	99.8	99.9	0	0	0	99.8	99.8	23	0	99.9	0	99.9	91.2	99.9	0	99.9	0	0	0	0	0.2	0	0	0	0	0	0	0	

143. A 3G TRAC Ex.3 SA + coBE4 mRNA 4 µg

	A_{-10}	A_{-9}	A_{-8}	G_{-7}	T_{-6}	T_{-5}	T_{-4}	A_{-3}	G_{-2}	G_{-1}	T_1	T_2	C_3	G_4	T_5	A_6	T_7	C_8	T_9	G_{10}	T_{11}	A_{12}	A_{13}	A_{14}	A_{15}	C_{16}	C_{17}	A_{18}	A_{19}	G_{20}	A_{21}	G_{22}	G_{23}	Indel
A	99.9	99.9	99.7	0.1	0	0	0	99.9	0	0	0	0	0	0.1	0	100	0	0.1	0	0	0	99.9	99.8	99.8	99.7	0	0	99.9	99.8	0	99.7	0	0.1	0.5
C	0	0	0	0	0.1	0.2	0.1	0	0	0	0.2	0.2	73.5	0	0.1	0	0.1	6.5	0.1	0	0.1	0	0	0	0	99.7	99.9	0	0	0	0	0	0	
G	0.1	0.1	0.3	99.9	0	0	0	0.1	100	99.9	0	0	0	99.9	0	0	0	0.5	0	100	0	0	0.2	0.2	0.2	0	0	0.1	0.2	100	0.3	100	99.9	
T	0	0	0	0	99.8	99.8	99.9	0	0	0	99.8	99.8	26.5	0	99.9	0	99.9	92.8	99.9	0	99.9	0	0	0	0	0.3	0	0	0	0	0	0	0	

144. B 3G TRAC Ex.3 SA + coBE4 mRNA 4 µg

	A_{-10}	A_{-9}	A_{-8}	G_{-7}	T_{-6}	T_{-5}	T_{-4}	A_{-3}	G_{-2}	G_{-1}	T_1	T_2	C_3	G_4	T_5	A_6	T_7	C_8	T_9	G_{10}	T_{11}	A_{12}	A_{13}	A_{14}	A_{15}	C_{16}	C_{17}	A_{18}	A_{19}	G_{20}	A_{21}	G_{22}	G_{23}	Indel
A	99.9	99.9	99.7	0	0	0	0	99.9	0	0	0	0	0	0	0	100	0	0.2	0	0	0	99.9	99.8	99.8	99.7	0	0	99.9	99.8	0	99.7	0	0	0.4
C	0	0	0	0	0.1	0.1	0.1	0	0	0	0.2	0.1	73.8	0	0.1	0	0.1	6.1	0.1	0	0.1	0	0	0	0	99.8	99.9	0	0	0	0	0	0	
G	0.1	0.1	0.2	100	0	0	0	0.1	100	100	0	0	0	99.9	0	0	0	0.8	0	100	0	0	0.2	0.2	0.2	0	0	0.1	0.2	100	0.3	100	99.9	
T	0	0	0	0	99.9	99.8	99.9	0	0	0	99.8	99.8	26.1	0	99.9	0	99.8	92.9	99.9	0	99.9	0	0	0	0	0.2	0.1	0	0	0	0	0	0	

145. B 3G TRAC Ex.3 SA + coBE4 mRNA 4 µg

	A_{-10}	A_{-9}	A_{-8}	G_{-7}	T_{-6}	T_{-5}	T_{-4}	A_{-3}	G_{-2}	G_{-1}	T_1	T_2	C_3	G_4	T_5	A_6	T_7	C_8	T_9	G_{10}	T_{11}	A_{12}	A_{13}	A_{14}	A_{15}	C_{16}	C_{17}	A_{18}	A_{19}	G_{20}	A_{21}	G_{22}	G_{23}	Indel
A	99.9	99.9	99.8	0	0	0	0	99.9	0.1	0	0	0	0.1	0.1	0	99.9	0	0.3	0	0	0	100	99.9	99.9	99.7	0	0	99.9	99.8	0	99.7	0	0.1	0.2
C	0	0	0	0	0.1	0.1	0.1	0	0	0	0.1	0.1	78.5	0	0.1	0	0.1	17.1	0.1	0	0	0	0	0	0.1	99.8	99.9	0	0	0	0.1	0	0	
G	0.1	0.1	0.2	99.9	0.1	0.1	0	0.1	99.9	99.9	0	0	0	99.9	0	0	0	0.9	0	99.9	0	0	0.1	0.1	0.2	0	0	0.1	0.2	99.9	0.2	99.9	99.8	
T	0	0	0	0	99.9	99.8	99.9	0	0	0	99.8	99.8	21.4	0	99.9	0	99.8	81.7	99.9	0	99.9	0	0	0	0	0.2	0.1	0	0	0	0	0.1	0.1	

146. C 3G TRAC Ex.3 SA + coBE4 mRNA 4 µg

	A_{-10}	A_{-9}	A_{-8}	G_{-7}	T_{-6}	T_{-5}	T_{-4}	A_{-3}	G_{-2}	G_{-1}	T_1	T_2	C_3	G_4	T_5	A_6	T_7	C_8	T_9	G_{10}	T_{11}	A_{12}	A_{13}	A_{14}	A_{15}	C_{16}	C_{17}	A_{18}	A_{19}	G_{20}	A_{21}	G_{22}	G_{23}	Indel
A	100	100	100	0	0	0	0	100	0	0	0	0	0	0	0	100	0	0	0	0	0	100	100	100	100	0	0	100	100	0	100	0	0	0
C	0	0	0	0	0	0	0	0	0	0	0	0	100	0	0	0	0	50	0	0	0	0	0	0	0	100	100	0	0	0	0	0	0	
G	0	0	0	100	0	0	0	0	100	100	0	0	0	100	0	0	0	50	0	100	0	0	0	0	0	0	0	0	0	100	0	100	100	
T	0	0	0	0	100	100	100	0	0	0	100	100	0	0	100	0	100	0	100	0	100	0	0	0	0	0	0	0	0	0	0	0	0	

147. A 3G TRAC Ex.3 SA + Cas9 mRNA 1.5 µg

	A_{-10}	A_{-9}	A_{-8}	G_{-7}	T_{-6}	T_{-5}	T_{-4}	A_{-3}	G_{-2}	G_{-1}	T_1	T_2	C_3	G_4	T_5	A_6	T_7	C_8	T_9	G_{10}	T_{11}	A_{12}	A_{13}	A_{14}	A_{15}	C_{16}	C_{17}	A_{18}	A_{19}	G_{20}	A_{21}	G_{22}	G_{23}	Indel
A	99.8	99.8	99.7	0	0	0	0.1	99.8	0.1	0.1	0	0.1	0.2	0.1	0.2	99.8	0.2	0.1	0.1	0.1	0.1	99.7	99.5	99.6	99.4	0.2	0.3	99.6	99.6	0.1	99.7	0	0.1	94
C	0	0.1	0	0	0.2	0.2	0	0	0	0	0.9	0.2	99.3	0.1	0.1	0	0.3	99.3	0.1	0	0.2	0	0.1	0.1	0.2	99.7	99.1	0.2	0	0	0.1	0	0.1	
G	0.1	0	0.2	99.9	0.1	0.1	0	0.1	99.9	99.8	0	0	0	99.5	0.3	0.1	0.2	0.3	0.2	99.8	0.1	0.2	0.3	0.1	0.3	0	0.6	0.1	0.3	99.7	0.2	99.9	99.8	
T	0	0	0	0.1	99.7	99.7	99.8	0	0	0.1	99.1	99.7	0.5	0.2	99.5	0	99.4	0.3	99.6	0.1	99.7	0.1	0.2	0.2	0.1	0.1	0	0.2	0.1	0.2	0	0.1	0.1	

148. B 3G TRAC Ex.3 SA + Cas9 mRNA 1.5 μg

	A_{-10}	A_{-9}	A_{-8}	G_{-7}	T_{-6}	T_{-5}	T_{-4}	A_{-3}	G_{-2}	G_{-1}	T_1	T_2	C_3	G_4	T_5	A_6	T_7	C_8	T_9	G_{10}	T_{11}	A_{12}	A_{13}	A_{14}	A_{15}	C_{16}	C_{17}	A_{18}	A_{19}	G_{20}	A_{21}	G_{22}	G_{23}	Indel
A	99.8	99.9	99.6	0	0	0	0	99.9	0	0.1	0	0.2	0	0.1	0.2	99.7	0.2	0.1	0.1	0.1	0.1	99.6	99.4	99.6	99.4	0.2	0.4	99.5	99.6	0	99.5	0.1	0.1	91.7
C	0	0	0	0	0.1	0.2	0	0	0	0	0.2	0.1	99.8	0.1	0.2	0	0.2	99.5	0.3	0	0.2	0.1	0.2	0.2	0.2	99.6	98.7	0.2	0	0	0.1	0	0.1	
G	0.1	0.1	0.3	100	0	0.1	0	0.1	99.9	99.8	0.1	0	0	99.7	0.1	0.2	0	0.1	0.1	99.8	0.2	0.2	0.3	0.2	0.3	0.1	0.7	0.2	0.2	99.8	0.3	99.9	99.8	
T	0	0	0	0	99.9	99.8	99.9	0	0	0	99.6	99.7	0.2	0.1	99.5	0.1	99.5	0.2	99.6	0.1	99.5	0.2	0.1	0	0.1	0.1	0.2	0.1	0.2	0.1	0.1	0	0	

149. A 3G TRAC Ex.3 SA + Cas9 mRNA 1.5 μg

	A_{-10}	A_{-9}	A_{-8}	G_{-7}	T_{-6}	T_{-5}	T_{-4}	A_{-3}	G_{-2}	G_{-1}	T_1	T_2	C_3	G_4	T_5	A_6	T_7	C_8	T_9	G_{10}	T_{11}	A_{12}	A_{13}	A_{14}	A_{15}	C_{16}	C_{17}	A_{18}	A_{19}	G_{20}	A_{21}	G_{22}	G_{23}	Indel
A	99.9	99.9	99.7	0	0	0	0	99.9	0.1	0.1	0	0.1	0.1	0.1	0.1	99.7	0.1	0.1	0	0.2	0.1	99.5	99.4	99.5	99.1	0.4	0.8	99.4	99.4	0.2	99.3	0	0.1	91.9
C	0	0	0	0	0.1	0.2	0	0	0.1	0.1	0.2	0.1	99.6	0.1	0.2	0.1	0.2	99.6	0.2	0	0.2	0	0.2	0.1	0.4	99.2	97.6	0.1	0.1	0.1	0.2	0	0	
G	0.1	0.1	0.2	100	0	0	0	0.1	99.8	99.8	0	0	0	99.7	0.1	0.1	0	0.1	0.1	99.7	0.1	0.3	0.2	0.3	0.4	0.1	1.1	0.3	0.3	99.6	0.3	99.8	99.8	
T	0	0	0	0	99.8	99.8	99.9	0	0	0	99.7	99.7	0.3	0.1	99.6	0.1	99.6	0.2	99.7	0.1	99.6	0.2	0.2	0.1	0.1	0.3	0.5	0.2	0.2	0.2	0.2	0.1	0	

150. B 3G TRAC Ex.3 SA + Cas9 mRNA 1.5 μg

	A_{-10}	A_{-9}	A_{-8}	G_{-7}	T_{-6}	T_{-5}	T_{-4}	A_{-3}	G_{-2}	G_{-1}	T_1	T_2	C_3	G_4	T_5	A_6	T_7	C_8	T_9	G_{10}	T_{11}	A_{12}	A_{13}	A_{14}	A_{15}	C_{16}	C_{17}	A_{18}	A_{19}	G_{20}	A_{21}	G_{22}	G_{23}	Indel
A	99.9	99.9	99.7	0	0	0	0.1	99.8	0	0.1	0	0.1	0	0.1	0.1	99.8	0.2	0.1	0.1	0.1	0.1	99.7	99.5	99.7	99.3	0.4	0.7	99.6	99.6	0.2	99.5	0	0.1	92.2
C	0	0	0	0	0.1	0.2	0	0	0	0	0.2	0.1	99.8	0	0.1	0	0.2	99.5	0.3	0	0.1	0	0.1	0	0.3	99.4	98	0.1	0	0.1	0.1	0	0	
G	0.1	0.1	0.3	99.9	0	0	0	0.1	99.9	99.9	0	0	0	99.8	0	0.1	0	0.1	0	99.7	0.1	0.2	0.2	0.2	0.4	0.1	0.9	0.1	0.2	99.6	0.3	99.8	99.8	
T	0	0	0	0	99.8	99.8	99.9	0	0	0	99.8	99.8	0.2	0	99.7	0	99.6	0.4	99.6	0.1	99.6	0.1	0.1	0.1	0.1	0.1	0.4	0.2	0.2	0.1	0.1	0.1	0	

151. A 3G TRAC Ex.3 SA + Cas9 mRNA 4 μg

	A_{-10}	A_{-9}	A_{-8}	G_{-7}	T_{-6}	T_{-5}	T_{-4}	A_{-3}	G_{-2}	G_{-1}	T_1	T_2	C_3	G_4	T_5	A_6	T_7	C_8	T_9	G_{10}	T_{11}	A_{12}	A_{13}	A_{14}	A_{15}	C_{16}	C_{17}	A_{18}	A_{19}	G_{20}	A_{21}	G_{22}	G_{23}	Indel
A	99.8	99.9	99.8	0	0	0	0.1	99.7	0.1	0.1	0	0.1	0.1	0.1	0.2	99.7	0.1	0.1	0.1	0.2	0.2	99.6	99.2	99.6	99.3	0.2	0.6	99.6	99.6	0.1	99.6	0.1	0.1	89.3
C	0	0	0	0	0.1	0.1	0.1	0	0	0	0.2	0.1	99.6	0	0.1	0	0.2	99.5	0.4	0	0.2	0	0.2	0.1	0.4	99.4	99	0.2	0.1	0.1	0	0	0	
G	0.1	0.1	0.2	99.9	0.1	0.2	0	0.2	99.8	99.7	0	0.1	0	99.8	0.1	0.1	0.1	0.1	0.2	99.6	0.1	0.2	0.2	0.2	0.2	0.1	0.3	0.2	0.2	99.7	0.3	99.7	99.8	
T	0.1	0	0	0	99.8	99.7	99.8	0.1	0.1	0.1	99.7	99.7	0.3	0.1	99.6	0.2	99.6	0.2	99.4	0.2	99.6	0.2	0.3	0.1	0.1	0.2	0.1	0.1	0.1	0.1	0.1	0.2	0.1	

152. B 3G TRAC Ex.3 SA + Cas9 mRNA 4 μg

	A_{-10}	A_{-9}	A_{-8}	G_{-7}	T_{-6}	T_{-5}	T_{-4}	A_{-3}	G_{-2}	G_{-1}	T_1	T_2	C_3	G_4	T_5	A_6	T_7	C_8	T_9	G_{10}	T_{11}	A_{12}	A_{13}	A_{14}	A_{15}	C_{16}	C_{17}	A_{18}	A_{19}	G_{20}	A_{21}	G_{22}	G_{23}	Indel
A	99.9	99.9	99.7	0	0.1	0	0.1	99.7	0.1	0.1	0	0.1	0.1	0.1	0	99.7	0.1	0.1	0.1	0.1	0.2	99.5	99	99.5	99.1	0.3	0.5	99.5	99.6	0.1	99.5	0	0.1	92.2
C	0	0	0	0.1	0.2	0.2	0.1	0	0	0	0.3	0.2	99.6	0	0.3	0	0.3	99.5	0.3	0	0.3	0	0.3	0.2	0.4	99.4	99	0.2	0	0	0	0	0	
G	0.1	0.1	0.2	99.9	0.1	0.1	0.1	0.2	99.8	99.8	0.1	0.1	0	99.8	0.1	0.2	0.1	0.1	0.1	99.8	0.1	0.2	0.4	0.3	0.4	0.1	0.3	0.2	0.2	99.7	0.4	99.9	99.8	
T	0	0	0	0	99.7	99.7	99.8	0.1	0.1	0	99.6	99.7	0.3	0.1	99.6	0.1	99.5	0.4	99.6	0	99.4	0.2	0.3	0	0.1	0.2	0.3	0.1	0.2	0.2	0.1	0.1	0	

153. A 3G TRAC Ex.3 SA + Cas9 mRNA 4 μg

	A_{-10}	A_{-9}	A_{-8}	G_{-7}	T_{-6}	T_{-5}	T_{-4}	A_{-3}	G_{-2}	G_{-1}	T_1	T_2	C_3	G_4	T_5	A_6	T_7	C_8	T_9	G_{10}	T_{11}	A_{12}	A_{13}	A_{14}	A_{15}	C_{16}	C_{17}	A_{18}	A_{19}	G_{20}	A_{21}	G_{22}	G_{23}	Indel
A	99.8	99.9	99.7	0	0	0	0	99.8	0	0.1	0.1	0.1	0	0.2	0.2	99.6	0.2	0.2	0.1	0.2	0.2	99.5	99.2	99.6	99.2	0.2	0.7	99.3	99.4	0.2	99.3	0	0.1	90.8
C	0	0	0.1	0.1	0.1	0.2	0.1	0	0	0.1	0.3	0.2	99.3	0.1	0.1	0.1	0.5	97.5	0.3	0	0.2	0	0.2	0.1	0.4	99.5	98	0.2	0.1	0.1	0.1	0	0	
G	0.1	0.1	0.3	99.8	0.1	0.1	0.1	0.1	99.9	99.8	0	0	0	99.7	0.2	0.2	0.1	0.2	0.1	99.6	0.2	0.2	0.4	0.2	0.3	0	0.8	0.2	0.3	99.7	0.4	99.9	99.9	
T	0	0	0	0	99.8	99.8	99.8	0	0	0	99.6	99.7	0.7	0	99.5	0.2	99.2	2	99.5	0.1	99.4	0.2	0.2	0.1	0.1	0.2	0.6	0.3	0.2	0.1	0.2	0.1	0	

154. B 3G TRAC Ex.3 SA + Cas9 mRNA 4 μg

	A_{-10}	A_{-9}	A_{-8}	G_{-7}	T_{-6}	T_{-5}	T_{-4}	A_{-3}	G_{-2}	G_{-1}	T_1	T_2	C_3	G_4	T_5	A_6	T_7	C_8	T_9	G_{10}	T_{11}	A_{12}	A_{13}	A_{14}	A_{15}	C_{16}	C_{17}	A_{18}	A_{19}	G_{20}	A_{21}	G_{22}	G_{23}	Indel
A	99.9	99.9	99.7	0	0	0	0.1	99.8	0	0	0.1	0.1	0.1	0.1	0.2	99.7	0.2	0.2	0	0.1	0.1	99.6	99.3	99.6	99.3	0.2	0.4	99.4	99.3	0.1	99.3	0.1	0.1	95.1
C	0	0	0	0	0.1	0.2	0.1	0	0	0	0.3	0.2	99.4	0	0.2	0	0.3	98.9	0.2	0.1	0.2	0.1	0.2	0.1	0.2	99.5	98.8	0.2	0.1	0	0.1	0.1	0	
G	0.1	0.1	0.3	99.9	0	0	0	0.2	99.9	99.9	0.1	0	0.1	99.8	0.1	0.2	0.1	0.3	0.1	99.8	0.2	0.2	0.3	0.2	0.3	0.1	0.5	0.2	0.4	99.7	0.4	99.8	99.8	
T	0	0	0	0	99.8	99.8	99.8	0	0	0	99.6	99.7	0.4	0.1	99.5	0.1	99.5	0.7	99.7	0.1	99.5	0.1	0.2	0.1	0.1	0.1	0.3	0.2	0.2	0.1	0.2	0.1	0	

155. A 3G TRAC Ex.3 SA + Cas9 RNP

	A_{-10}	A_{-9}	A_{-8}	G_{-7}	T_{-6}	T_{-5}	T_{-4}	A_{-3}	G_{-2}	G_{-1}	T_1	T_2	C_3	G_4	T_5	A_6	T_7	C_8	T_9	G_{10}	T_{11}	A_{12}	A_{13}	A_{14}	A_{15}	C_{16}	C_{17}	A_{18}	A_{19}	G_{20}	A_{21}	G_{22}	G_{23}	Indel
A	99.9	99.9	99.7	0	0	0	0	99.8	0.1	0.1	0	0.1	0.1	0.2	0.2	99.8	0.2	0.1	0.1	0.1	0.1	99.7	99.2	99.5	99.2	1.6	2.9	99.2	99.1	0.4	99.4	0.1	0.1	89.9
C	0	0	0	0	0.1	0.2	0	0	0	0	0.2	0.2	99.6	0	0.1	0	0.2	99.3	0.3	0	0.2	0	0.2	0.1	0.3	97.8	93.7	0.2	0.2	0.1	0.1	0.1	0.1	
G	0.1	0.1	0.2	99.9	0.1	0	0	0.1	99.9	99.9	0	0	0	99.7	0.1	0.1	0.1	0.2	0.1	99.7	0.1	0.1	0.3	0.4	0.4	0.2	2.8	0.3	0.2	99.3	0.4	99.7	99.8	
T	0	0	0	0	99.8	99.8	99.9	0	0	0	99.7	99.7	0.3	0.1	99.6	0.1	99.5	0.4	99.5	0.2	99.6	0.2	0.2	0.1	0.1	0.4	0.6	0.3	0.5	0.2	0.1	0.1	0	

156. B 3G TRAC Ex.3 SA + Cas9 RNP

	A_{-10}	A_{-9}	A_{-8}	G_{-7}	T_{-6}	T_{-5}	T_{-4}	A_{-3}	G_{-2}	G_{-1}	T_{1}	T_{2}	C_{3}	G_{4}	T_{5}	A_{6}	T_{7}	C_{8}	T_{9}	G_{10}	T_{11}	A_{12}	A_{13}	A_{14}	A_{15}	C_{16}	C_{17}	A_{18}	A_{19}	G_{20}	A_{21}	G_{22}	G_{23}	Indel
A	99.9	99.9	99.7	0	0	0	0	99.8	0	0.1	0	0	0	0.1	0.1	99.8	0.2	0.2	0.1	0.2	0.1	99.5	99.2	99.5	99.1	1.3	2.1	99.3	99.4	0.3	99.4	0.1	0.1	87.2
C	0	0	0	0	0.1	0.1	0.1	0	0	0	0.2	0.2	99.6	0.1	0.1	0	0.2	99	0.2	0	0.2	0.1	0.2	0.1	0.4	98.2	95.7	0.2	0.2	0.1	0.2	0	0.1	
G	0.1	0.1	0.3	99.9	0	0.1	0	0.1	99.9	99.9	0	0	0	99.7	0.1	0.1	0.1	0.2	0.1	99.7	0	0.2	0.3	0.3	0.4	0.2	1.9	0.3	0.2	99.5	0.3	99.7	99.8	
T	0	0	0	0	99.8	99.8	99.9	0	0	0	99.8	99.8	0.3	0	99.7	0.1	99.6	0.5	99.6	0.2	99.7	0.2	0.3	0.1	0.1	0.4	0.4	0.2	0.3	0.1	0.1	0.2	0	

157. A 3G PDCD1 Ex.1 SD + Pulse

	C_{-10}	C_{-9}	G_{-8}	C_{-7}	C_{-6}	G_{-5}	A_{-4}	C_{-3}	C_{-2}	C_{-1}	C_{1}	A_{2}	C_{3}	C_{4}	T_{5}	A_{6}	C_{7}	C_{8}	T_{9}	A_{10}	A_{11}	G_{12}	A_{13}	A_{14}	C_{15}	C_{16}	A_{17}	T_{18}	C_{19}	C_{20}	T_{21}	G_{22}	G_{23}	Indel
A	0.1	0.1	0	0	0	0	99.9	0.2	0	0.1	0	99.8	0.2	0	0.1	99	0.2	0.2	0.1	99.3	99.6	0	99.6	99.8	0.1	0	99.4	0.2	0	0.1	0	0	0.2	0.9
C	99.9	99.9	0	100	100	0.1	0.1	99.8	99.7	99.8	100	0.2	99.6	99.8	0	0.3	98.8	99.1	0.2	0.2	0.3	0	0.3	0.2	99.8	100	0.2	0.2	100	99.8	0.2	0	0	
G	0	0	100	0	0	99.8	0	0	0.1	0.1	0	0.1	0	0	0.1	0.5	0	0.1	0.5	0.4	0	99.8	0.1	0	0	0	0.2	0.2	0	0	0.1	99.9	99.8	
T	0	0	0	0	0	0.2	0	0.1	0.2	0	0	0	0.2	0.2	99.8	0.2	1	0.7	99.2	0.1	0.1	0.2	0	0	0.1	0	0.1	99.2	0	0.2	99.7	0.1	0	

158. B 3G PDCD1 Ex.1 SD + Pulse

	C_{-10}	C_{-9}	G_{-8}	C_{-7}	C_{-6}	G_{-5}	A_{-4}	C_{-3}	C_{-2}	C_{-1}	C_{1}	A_{2}	C_{3}	C_{4}	T_{5}	A_{6}	C_{7}	C_{8}	T_{9}	A_{10}	A_{11}	G_{12}	A_{13}	A_{14}	C_{15}	C_{16}	A_{17}	T_{18}	C_{19}	C_{20}	T_{21}	G_{22}	G_{23}	Indel
A	0	0	0.1	0	0	0.1	99.9	0	0	0	0	99.9	0	0.1	0	99.6	0.3	0.2	0	100	99.9	0	99.9	99.9	0	0	99.9	0	0	0	0	0	0	0.4
C	100	99.9	0	99.9	99.9	0	0	100	99.9	99.9	100	0	99.9	99.6	0.1	0	98.2	98.8	0.1	0	0	0	0	0	100	100	0	0.1	99.9	100	0.1	0	0	
G	0	0	99.9	0	0	99.9	0.1	0	0	0	0	0.1	0	0	0	0.4	0.1	0.2	0	0	0.1	99.9	0.1	0	0	0	0.1	0	0	0	0	100	99.9	
T	0	0	0	0	0.1	0	0	0	0.1	0	0	0	0.1	0.3	99.8	0	1.3	0.7	99.9	0	0	0	0	0	0	0	0	99.8	0	0	99.9	0	0	

159. A 3G PDCD1 Ex.1 SD + Pulse

	C_{-10}	C_{-9}	G_{-8}	C_{-7}	C_{-6}	G_{-5}	A_{-4}	C_{-3}	C_{-2}	C_{-1}	C_{1}	A_{2}	C_{3}	C_{4}	T_{5}	A_{6}	C_{7}	C_{8}	T_{9}	A_{10}	A_{11}	G_{12}	A_{13}	A_{14}	C_{15}	C_{16}	A_{17}	T_{18}	C_{19}	C_{20}	T_{21}	G_{22}	G_{23}	Indel
A	0	0	0.1	0	0	0.1	99.9	0	0	0	0	99.9	0	0	0	99.9	0.1	0.1	0	100	99.9	0	99.9	99.9	0	0	99.9	0	0	0	0	0	0	0.1
C	100	99.9	0	99.9	99.9	0	0	100	100	100	100	0	99.8	99.7	0.1	0	98.8	98.9	0.1	0	0	0	0	0	99.9	100	0	0.1	99.9	100	0.1	0	0	
G	0	0	99.9	0	0	99.9	0	0	0	0	0	0.1	0	0	0	0.1	0	0.1	0	0	0.1	99.9	0.1	0.1	0	0	0.1	0	0	0	0	99.9	99.9	
T	0	0	0	0	0.1	0	0	0	0	0	0	0	0.2	0.3	99.9	0	1.1	0.9	99.9	0	0	0	0	0	0	0	0	99.8	0	0	99.8	0	0	

160. B 3G PDCD1 Ex.1 SD + Pulse

	C_{-10}	C_{-9}	G_{-8}	C_{-7}	C_{-6}	G_{-5}	A_{-4}	C_{-3}	C_{-2}	C_{-1}	C_{1}	A_{2}	C_{3}	C_{4}	T_{5}	A_{6}	C_{7}	C_{8}	T_{9}	A_{10}	A_{11}	G_{12}	A_{13}	A_{14}	C_{15}	C_{16}	A_{17}	T_{18}	C_{19}	C_{20}	T_{21}	G_{22}	G_{23}	Indel
A	0	0	0.1	0	0	0.1	99.6	0.1	0	0	0.1	99.6	0.1	0	0	99.4	0	0	0	99.5	99.5	0.1	99.6	99.5	0.1	0.1	99.5	0.1	0	0.1	0	0	0	0.1
C	99.9	99.9	0	99.9	99.9	0	0.3	99.9	99.9	99.9	99.9	0.3	99.8	99.8	0.1	0.4	99.6	99.6	0.1	0.3	0.3	0	0.2	0.3	99.9	99.9	0.3	0.3	99.9	99.9	0.2	0	0	
G	0	0	99.8	0	0	99.7	0.1	0	0	0	0	0.1	0	0	0.3	0.1	0	0	0.4	0.2	0.1	99.7	0.1	0.1	0	0	0.1	0.7	0	0	0.2	99.8	99.9	
T	0	0	0.1	0	0.1	0.2	0	0	0	0	0	0	0.1	0.2	99.5	0.1	0.4	0.4	99.4	0	0	0.2	0	0.1	0	0	0.1	98.9	0	0	99.6	0.1	0.1	

161. C 3G PDCD1 Ex.1 SD + Pulse

	C_{-10}	C_{-9}	G_{-8}	C_{-7}	C_{-6}	G_{-5}	A_{-4}	C_{-3}	C_{-2}	C_{-1}	C_{1}	A_{2}	C_{3}	C_{4}	T_{5}	A_{6}	C_{7}	C_{8}	T_{9}	A_{10}	A_{11}	G_{12}	A_{13}	A_{14}	C_{15}	C_{16}	A_{17}	T_{18}	C_{19}	C_{20}	T_{21}	G_{22}	G_{23}	Indel
A	0	0	0	0	0	0.1	99.9	0	0	0	0	99.9	0	0	0	99.9	0	0	0	100	99.9	0	99.9	99.9	0	0	99.9	0	0	0	0	0	0	0.1
C	100	99.9	0	99.9	100	0	0	100	100	99.9	100	0	99.6	99.1	0.1	0	98.1	98.1	0.1	0	0	0	0	0	99.9	99.9	0	0.1	99.9	100	0.1	0	0	
G	0	0	99.9	0	0	99.9	0.1	0	0	0	0	0.1	0	0	0	0.1	0	0	0	0	0.1	99.9	0.1	0.1	0	0	0.1	0	0	0	0	99.9	99.9	
T	0	0	0	0	0	0	0	0	0	0	0	0	0.4	0.9	99.9	0	1.9	1.9	99.8	0	0	0	0	0	0	0	0	99.8	0	0	99.9	0	0	

162. A 3G PDCD1 Ex.1 SD + Pulse

	C_{-10}	C_{-9}	G_{-8}	C_{-7}	C_{-6}	G_{-5}	A_{-4}	C_{-3}	C_{-2}	C_{-1}	C_{1}	A_{2}	C_{3}	C_{4}	T_{5}	A_{6}	C_{7}	C_{8}	T_{9}	A_{10}	A_{11}	G_{12}	A_{13}	A_{14}	C_{15}	C_{16}	A_{17}	T_{18}	C_{19}	C_{20}	T_{21}	G_{22}	G_{23}	Indel
A	0	0	0.1	0	0	0	99.9	0	0	0	0	99.9	0	0	0	99.7	0.1	0.1	0	99.9	99.9	0	99.9	99.9	0	0	99.9	0	0	0	0	0	0.1	0.4
C	100	99.9	0	99.9	99.9	0	0	100	100	99.9	100	0	99.9	99.9	0.1	0	98.9	99.1	0.1	0	0	0	0	0	100	100	0	0.1	99.9	100	0.1	0	0	
G	0	0	99.9	0	0	99.9	0.1	0	0	0	0	0.1	0	0	0	0.3	0.1	0.2	0	0.1	0.1	100	0.1	0.1	0	0	0.1	0	0	0	0	99.9	99.9	
T	0	0.1	0	0	0.1	0	0	0	0	0.1	0	0	0.1	0.1	99.8	0	0.9	0.6	99.9	0	0	0	0	0	0	0	0	99.8	0	0	99.9	0	0	

163. A 3G PDCD1 Ex.1 SD + Pulse

	C_{-10}	C_{-9}	G_{-8}	C_{-7}	C_{-6}	G_{-5}	A_{-4}	C_{-3}	C_{-2}	C_{-1}	C_{1}	A_{2}	C_{3}	C_{4}	T_{5}	A_{6}	C_{7}	C_{8}	T_{9}	A_{10}	A_{11}	G_{12}	A_{13}	A_{14}	C_{15}	C_{16}	A_{17}	T_{18}	C_{19}	C_{20}	T_{21}	G_{22}	G_{23}	Indel
A	0	0	0.1	0	0	0	99.9	0	0	0	0	99.8	0	0.1	0	99.5	0.1	0.2	0	100	99.9	0	99.9	99.9	0	0	99.9	0	0	0	0	0	0	0.3
C	100	99.9	0	99.9	99.9	0	0	100	100	100	100	0	99.9	99.7	0.1	0	98.8	98.9	0.1	0	0	0	0	0	100	99.9	0	0.2	99.9	100	0.1	0	0	
G	0	0	99.9	0	0	100	0.1	0	0	0	0	0.1	0	0	0	0.4	0.1	0.2	0	0	0.1	99.9	0.1	0.1	0	0	0.1	0	0	0	0	99.9	100	
T	0	0.1	0	0	0.1	0	0	0	0	0	0	0	0.1	0.3	99.9	0	1	0.8	99.9	0	0	0	0	0	0	0	0	99.8	0	0	99.9	0	0	

164. B 3G PDCD1 Ex.1 SD + Pulse

	C_{-10}	C_{-9}	G_{-8}	C_{-7}	C_{-6}	G_{-5}	A_{-4}	C_{-3}	C_{-2}	C_{-1}	C_{1}	A_{2}	C_{3}	C_{4}	T_{5}	A_{6}	C_{7}	C_{8}	T_{9}	A_{10}	A_{11}	G_{12}	A_{13}	A_{14}	C_{15}	C_{16}	A_{17}	T_{18}	C_{19}	C_{20}	T_{21}	G_{22}	G_{23}	Indel
A	0	0	0.1	0	0	0	99.9	0	0	0	0	99.8	0	0.2	0	98.9	0.6	0.2	0	99.9	99.9	0	99.9	99.9	0	0	99.8	0	0	0	0	0	0	0.5
C	100	99.9	0	99.9	99.9	0	0	100	100	99.9	100	0	99.7	99.4	0.1	0	96.6	98	0.1	0	0	0	0	0	99.9	100	0	0.2	99.9	100	0.1	0	0	
G	0	0	99.9	0	0	99.9	0.1	0	0	0	0	0.1	0	0	0	1.1	0.4	0.4	0	0	0.1	99.9	0.1	0	0	0	0.2	0	0	0	0	99.9	100	
T	0	0	0	0	0	0	0	0	0	0.1	0	0	0.3	0.4	99.9	0	2.4	1.4	99.9	0	0	0	0	0	0	0	0	99.8	0	0	99.9	0	0	

165. A 3G PDCD1 Ex.1 SD + Pulse

	C_{-10}	C_{-9}	G_{-8}	C_{-7}	C_{-6}	G_{-5}	A_{-4}	C_{-3}	C_{-2}	C_{-1}	C_{1}	A_{2}	C_{3}	C_{4}	T_{5}	A_{6}	C_{7}	C_{8}	T_{9}	A_{10}	A_{11}	G_{12}	A_{13}	A_{14}	C_{15}	C_{16}	A_{17}	T_{18}	C_{19}	C_{20}	T_{21}	G_{22}	G_{23}	Indel
A	0	0	0.1	0	0	0.1	99.9	0	0	0	0	99.9	0	0	0	99.8	0.1	0	0	100	99.9	0	99.9	99.9	0	0	99.9	0	0	0	0	0	0.1	0.4
C	100	99.9	0	99.9	99.9	0	0	100	100	99.9	100	0	99.9	99.8	0.1	0	99.1	99.3	0.1	0	0	0	0	0	100	99.9	0	0.1	100	100	0.1	0	0	
G	0	0	99.9	0	0	99.9	0.1	0	0	0	0	0.1	0	0	0	0.2	0	0.2	0	0	0.1	99.9	0.1	0.1	0	0	0.1	0	0	0	0	99.9	99.9	
T	0	0.1	0	0	0.1	0	0	0	0	0	0	0	0.1	0.1	99.8	0	0.8	0.5	99.9	0	0	0	0	0	0	0.1	0	99.8	0	0	99.8	0	0	

166. B 3G PDCD1 Ex.1 SD + Pulse

	C_{-10}	C_{-9}	G_{-8}	C_{-7}	C_{-6}	G_{-5}	A_{-4}	C_{-3}	C_{-2}	C_{-1}	C_{1}	A_{2}	C_{3}	C_{4}	T_{5}	A_{6}	C_{7}	C_{8}	T_{9}	A_{10}	A_{11}	G_{12}	A_{13}	A_{14}	C_{15}	C_{16}	A_{17}	T_{18}	C_{19}	C_{20}	T_{21}	G_{22}	G_{23}	Indel
A	0	0	0	0	0	0	99.9	0	0	0	0	99.9	0	0	0	99.8	0.1	0.1	0	100	99.9	0	99.9	99.9	0	0	99.9	0	0	0	0	0.1	0	0.2
C	99.9	99.9	0	100	99.9	0	0	99.9	100	100	100	0	99.9	99.8	0.1	0	99	99.3	0.1	0	0	0	0	0	99.9	100	0	0.1	99.9	100	0.1	0	0	
G	0	0	99.9	0	0	100	0.1	0	0	0	0	0.1	0	0	0	0.2	0.1	0	0	0	0.1	99.9	0.1	0.1	0	0	0.1	0	0	0	0	99.9	99.9	
T	0	0.1	0	0	0	0	0	0	0	0	0	0	0.1	0.2	99.8	0	0.8	0.5	99.9	0	0	0	0	0	0	0	0	99.8	0	0	99.8	0	0	

167. A 3G PDCD1 Ex.1 SD + BE3 conc. mRNA 3 µg

	C_{-10}	C_{-9}	G_{-8}	C_{-7}	C_{-6}	G_{-5}	A_{-4}	C_{-3}	C_{-2}	C_{-1}	C_{1}	A_{2}	C_{3}	C_{4}	T_{5}	A_{6}	C_{7}	C_{8}	T_{9}	A_{10}	A_{11}	G_{12}	A_{13}	A_{14}	C_{15}	C_{16}	A_{17}	T_{18}	C_{19}	C_{20}	T_{21}	G_{22}	G_{23}	Indel
A	0	0	0	0	0	0	99.3	0	0.1	0.1	0.1	99.6	0.2	0.7	0.5	99.3	6	3.7	0	99.5	99.7	0	99.5	99.5	0.1	0	99.2	0.1	0	0	0	0	0	3.2
C	100	99.9	0	99.9	100	0.1	0.4	99.9	99.9	99.9	99.9	0.3	99.1	97.7	0	0.4	67.5	81.1	0.1	0.3	0.3	0	0.2	0.3	99.9	99.8	0.5	0.2	99.8	99.9	0.2	0	0	
G	0	0	99.8	0	0	99.7	0.1	0	0	0	0	0.1	0	0.3	0.4	0.2	2.3	4.6	0.4	0.2	0	99.7	0.1	0.1	0	0	0.1	0.6	0	0	0.2	99.7	99.9	
T	0	0	0.1	0	0	0.2	0.1	0	0	0	0.1	0	0.6	1.4	99.1	0.1	24.2	10.6	99.4	0	0	0.2	0.1	0.1	0	0.1	0.2	99.1	0.2	0	99.6	0.2	0.1	

168. B 3G PDCD1 Ex.1 SD + BE3 conc. mRNA 3 µg

	C_{-10}	C_{-9}	G_{-8}	C_{-7}	C_{-6}	G_{-5}	A_{-4}	C_{-3}	C_{-2}	C_{-1}	C_{1}	A_{2}	C_{3}	C_{4}	T_{5}	A_{6}	C_{7}	C_{8}	T_{9}	A_{10}	A_{11}	G_{12}	A_{13}	A_{14}	C_{15}	C_{16}	A_{17}	T_{18}	C_{19}	C_{20}	T_{21}	G_{22}	G_{23}	Indel
A	0	0	0.1	0	0	0.1	99.8	0	0	0	0	99.7	0.1	0.7	0.1	97	3.7	2.1	0	99.8	99.8	0	99.8	99.9	0	0	99.9	0	0	0	0	0	0.1	4.6
C	99.9	99.8	0	99.8	99.9	0	0	99.9	99.9	99.9	99.9	0	98.5	96	0.2	0	75	83.2	0.1	0	0	0	0	0	99.9	99.9	0	0.2	99.8	99.9	0.2	0	0	
G	0	0	99.9	0	0	99.8	0.1	0	0	0	0	0.2	0	0.1	0	3	1.8	3.3	0	0.1	0.2	99.9	0.2	0.1	0	0	0.1	0	0	0	0	99.9	99.9	
T	0	0.1	0	0.1	0.1	0	0	0.1	0.1	0.1	0.1	0	1.4	3.2	99.7	0	19.5	11.3	99.8	0	0	0	0	0	0.1	0	0	99.8	0.2	0.1	99.8	0	0	

169. A 3G PDCD1 Ex.1 SD + BE4 conc. mRNA 3 µg

	C_{-10}	C_{-9}	G_{-8}	C_{-7}	C_{-6}	G_{-5}	A_{-4}	C_{-3}	C_{-2}	C_{-1}	C_{1}	A_{2}	C_{3}	C_{4}	T_{5}	A_{6}	C_{7}	C_{8}	T_{9}	A_{10}	A_{11}	G_{12}	A_{13}	A_{14}	C_{15}	C_{16}	A_{17}	T_{18}	C_{19}	C_{20}	T_{21}	G_{22}	G_{23}	Indel
A	0.1	0	0.1	0	0	0.1	99.7	0	0	0.1	0	99.8	0.2	1	0	99.6	4.1	3.1	0	99.4	99.8	0	99.9	99.8	0	0	99.7	0.1	0	0	0	0	0	5.1
C	99.9	99.9	0	99.9	99.9	0	0.2	99.9	99.9	99.8	99.9	0.1	97.8	95.4	0.1	0.1	71	78.5	0.1	0.1	0.1	0	0	0.1	99.9	99.9	0.2	0.2	99.7	99.9	0.1	0	0	
G	0	0	99.9	0	0	99.7	0.1	0	0	0	0	0.1	0	0.1	0.1	0.3	1	3	0.1	0.5	0.1	99.9	0.1	0.1	0	0	0	0.1	0	0	0.1	99.9	100	
T	0	0.1	0	0	0.1	0.1	0	0	0	0.1	0.1	0	2	3.6	99.8	0	23.9	15.3	99.8	0	0	0.1	0	0	0.1	0.1	0.1	99.6	0.3	0	99.8	0	0	

170. B 3G PDCD1 Ex.1 SD + BE4 conc. mRNA 3 µg

	C_{-10}	C_{-9}	G_{-8}	C_{-7}	C_{-6}	G_{-5}	A_{-4}	C_{-3}	C_{-2}	C_{-1}	C_{1}	A_{2}	C_{3}	C_{4}	T_{5}	A_{6}	C_{7}	C_{8}	T_{9}	A_{10}	A_{11}	G_{12}	A_{13}	A_{14}	C_{15}	C_{16}	A_{17}	T_{18}	C_{19}	C_{20}	T_{21}	G_{22}	G_{23}	Indel
A	0	0	0	0	0	0	100	0	0	0	0	100	0	0	0	100	0	40	0	100	100	0	100	100	0	0	100	0	0	0	0	0	0	0
C	100	100	0	100	100	0	0	100	100	100	100	0	100	100	0	0	60	60	0	0	0	0	0	0	100	100	0	0	100	100	0	0	0	
G	0	0	100	0	0	100	0	0	0	0	0	0	0	0	0	0	0	0	0	0	0	100	0	0	0	0	0	0	0	0	0	100	100	
T	0	0	0	0	0	0	0	0	0	0	0	0	0	0	100	0	40	0	100	0	0	0	0	0	0	0	0	100	0	0	100	0	0	

171. A 3G PDCD1 Ex.1 SD + BE4 RNP

	C_{-10}	C_{-9}	G_{-8}	C_{-7}	C_{-6}	G_{-5}	A_{-4}	C_{-3}	C_{-2}	C_{-1}	C_{1}	A_{2}	C_{3}	C_{4}	T_{5}	A_{6}	C_{7}	C_{8}	T_{9}	A_{10}	A_{11}	G_{12}	A_{13}	A_{14}	C_{15}	C_{16}	A_{17}	T_{18}	C_{19}	C_{20}	T_{21}	G_{22}	G_{23}	Indel
A	0	0	0.1	0	0	0.2	99.8	0.1	0	0	0	99.9	0.5	1.8	0.2	99.7	4.9	4	0	99.9	99.9	0	99.9	99.9	0	0	99.9	0	0	0	0	0	0	9.7
C	99.9	99.9	0	99.9	99.8	0	0.1	99.7	99.9	99.8	99.8	0	86.7	71.5	0.2	0	31.2	37.2	0	0	0	0	0	0	99.9	99.9	0	0.1	99.6	99.9	0.1	0	0	
G	0	0	99.9	0	0	99.7	0.2	0	0	0	0	0.1	0.1	0.2	0	0.2	2	5.5	0	0.1	0.1	99.9	0.1	0.1	0	0	0.1	0	0	0	0	99.9	99.9	
T	0.1	0.1	0	0	0.1	0	0	0.2	0.1	0.2	0.2	0	12.7	26.6	99.6	0	61.9	53.4	99.9	0	0	0	0	0	0.1	0.1	0	99.8	0.3	0.1	99.9	0	0	

172. B 3G PDCD1 Ex.1 SD + BE4 RNP

	C_{-10}	C_{-9}	G_{-8}	C_{-7}	C_{-6}	G_{-5}	A_{-4}	C_{-3}	C_{-2}	C_{-1}	C_1	A_2	C_3	C_4	T_5	A_6	C_7	C_8	T_9	A_{10}	A_{11}	G_{12}	A_{13}	A_{14}	C_{15}	C_{16}	A_{17}	T_{18}	C_{19}	C_{20}	T_{21}	G_{22}	G_{23}	Indel
A	0	0	0.1	0	0	0.1	99.9	0	0	0	0	99.8	0.2	2.4	0.2	99.7	5.4	4.6	0	99.9	99.9	0	99.7	99.9	0	0	99.9	0	0	0	0	0	0	8.9
C	99.8	99.8	0	99.9	99.9	0	0	99.8	99.9	99.8	99.8	0	86.1	71.4	0.1	0	29.1	35.8	0.1	0	0	0	0	0	99.8	99.9	0	0.1	99.7	100	0.1	0	0	
G	0	0	99.8	0	0	99.8	0.1	0	0	0	0	0.1	0.1	0.2	0	0.2	1.5	5.2	0	0	0.1	99.9	0.2	0.1	0	0	0.1	0	0	0	0	100	99.9	
T	0.1	0.1	0.1	0	0.1	0	0	0.2	0	0.1	0.2	0	13.5	26	99.6	0.1	64	54.4	99.8	0	0	0	0	0	0.2	0	0	99.8	0.3	0	99.8	0	0	

173. A 3G PDCD1 Ex.1 SD + coBE4 mRNA 1.5 μg

	C_{-10}	C_{-9}	G_{-8}	C_{-7}	C_{-6}	G_{-5}	A_{-4}	C_{-3}	C_{-2}	C_{-1}	C_1	A_2	C_3	C_4	T_5	A_6	C_7	C_8	T_9	A_{10}	A_{11}	G_{12}	A_{13}	A_{14}	C_{15}	C_{16}	A_{17}	T_{18}	C_{19}	C_{20}	T_{21}	G_{22}	G_{23}	Indel
A	0	0	0.1	0	0	0.2	99.9	0	0	0	0	99.9	0	0.2	0	99.7	0.6	0.3	0	100	99.9	0	99.9	99.9	0	0	99.9	0	0	0	0	0	0	2.1
C	99.9	99.9	0	99.9	99.9	0	0	99.8	99.9	99.9	99.8	0	85.9	64.9	0.1	0	22.1	22.7	0	0	0	0	0	0	99.9	100	0	0.1	99.7	99.9	0.1	0	0	
G	0	0	99.9	0	0	99.8	0.1	0	0	0	0	0.1	0	0.1	0	0.2	0.3	0.6	0	0	0.1	99.9	0.1	0.1	0	0	0.1	0	0	0	0	99.9	99.9	
T	0.1	0.1	0	0.1	0.1	0	0	0.2	0	0.1	0.2	0	14.1	34.8	99.9	0	77	76.3	99.9	0	0	0	0	0	0.1	0	0	99.8	0.3	0	99.9	0	0	

174. B 3G PDCD1 Ex.1 SD + coBE4 mRNA 1.5 μg

	C_{-10}	C_{-9}	G_{-8}	C_{-7}	C_{-6}	G_{-5}	A_{-4}	C_{-3}	C_{-2}	C_{-1}	C_1	A_2	C_3	C_4	T_5	A_6	C_7	C_8	T_9	A_{10}	A_{11}	G_{12}	A_{13}	A_{14}	C_{15}	C_{16}	A_{17}	T_{18}	C_{19}	C_{20}	T_{21}	G_{22}	G_{23}	Indel
A	0	0	0.1	0	0	0.2	99.9	0	0	0	0	99.9	0	0.5	0	99.6	1.1	0.9	0	100	99.9	0	99.8	99.9	0	0	99.9	0	0	0	0	0	0.1	3
C	99.8	99.9	0	99.9	99.9	0	0	99.7	99.9	99.9	99.8	0	87.3	66.2	0.1	0	20.8	22	0	0	0	0	0	0	99.8	99.9	0	0.1	99.8	99.9	0.1	0	0	
G	0	0	99.9	0	0	99.8	0.1	0	0	0	0	0.1	0	0.1	0	0.4	0.4	1.3	0	0	0.1	99.9	0.1	0.1	0	0	0	0	0	0	0	99.9	99.9	
T	0.2	0.1	0	0.1	0.1	0	0	0.2	0.1	0.1	0.1	0	12.6	33.2	99.8	0	77.6	75.8	99.9	0	0	0	0	0	0.1	0.1	0	99.8	0.2	0	99.9	0	0	

175. A 3G PDCD1 Ex.1 SD + coBE4 mRNA 4 μg

	C_{-10}	C_{-9}	G_{-8}	C_{-7}	C_{-6}	G_{-5}	A_{-4}	C_{-3}	C_{-2}	C_{-1}	C_1	A_2	C_3	C_4	T_5	A_6	C_7	C_8	T_9	A_{10}	A_{11}	G_{12}	A_{13}	A_{14}	C_{15}	C_{16}	A_{17}	T_{18}	C_{19}	C_{20}	T_{21}	G_{22}	G_{23}	Indel
A	0	0	0	0	0	0.2	99.9	0	0	0	0	99.9	0.1	0.2	0	99.9	0.6	0.3	0	99.9	99.9	0	99.9	99.9	0	0	99.9	0	0	0	0	0	0	1.7
C	100	99.9	0	99.9	99.9	0	0	99.8	99.9	99.9	99.9	0	84.5	61.4	0.1	0	14.6	14	0	0	0	0	0	0	99.9	100	0	0.1	99.6	99.8	0.1	0	0	
G	0	0	99.9	0	0	99.8	0.1	0	0	0	0	0.1	0.1	0.1	0	0	0.2	0.4	0	0.1	0.1	99.9	0.1	0.1	0	0	0.1	0	0	0	0	100	99.9	
T	0	0	0	0.1	0.1	0	0	0.2	0.1	0	0.1	0	15.4	38.3	99.9	0	84.6	85.3	99.9	0	0	0	0	0	0.1	0	0	99.8	0.4	0.1	99.8	0	0	

176. B 3G PDCD1 Ex.1 SD + coBE4 mRNA 4 μg

	C_{-10}	C_{-9}	G_{-8}	C_{-7}	C_{-6}	G_{-5}	A_{-4}	C_{-3}	C_{-2}	C_{-1}	C_1	A_2	C_3	C_4	T_5	A_6	C_7	C_8	T_9	A_{10}	A_{11}	G_{12}	A_{13}	A_{14}	C_{15}	C_{16}	A_{17}	T_{18}	C_{19}	C_{20}	T_{21}	G_{22}	G_{23}	Indel
A	0	0	0.2	0	0	0.1	99.9	0	0	0	0	99.9	0	0.1	0	100	1	0.9	0	99.9	99.9	0	99.8	99.9	0	0	99.9	0	0	0	0	0	0	2.5
C	100	99.9	0	99.9	99.9	0	0	99.8	99.9	100	100	0	88.7	66.6	0.1	0	16	16.1	0	0	0	0	0	0	99.9	100	0	0.2	99.8	100	0.1	0	0	
G	0	0	99.8	0	0	99.9	0.1	0	0	0	0	0.1	0	0.2	0	0	0.4	0.5	0	0	0.1	99.9	0.2	0.1	0	0	0.1	0	0	0	0	99.9	99.9	
T	0	0	0	0	0.1	0	0	0.1	0	0	0	0	11.2	33.1	99.9	0	82.6	82.5	99.9	0	0	0	0	0	0.1	0	0	99.8	0.2	0	99.9	0	0	

177. A 3G PDCD1 Ex.1 SD + coBE4 mRNA 4 μg

	C_{-10}	C_{-9}	G_{-8}	C_{-7}	C_{-6}	G_{-5}	A_{-4}	C_{-3}	C_{-2}	C_{-1}	C_1	A_2	C_3	C_4	T_5	A_6	C_7	C_8	T_9	A_{10}	A_{11}	G_{12}	A_{13}	A_{14}	C_{15}	C_{16}	A_{17}	T_{18}	C_{19}	C_{20}	T_{21}	G_{22}	G_{23}	Indel
A	0	0	0.1	0	0	0.1	99.9	0	0	0	0	99.9	0	0.1	0	99.7	0.2	0.2	0	99.9	99.9	0	99.9	99.9	0	0	99.9	0	0	0	0	0	0	1.7
C	99.8	99.9	0	99.9	99.9	0	0	99.7	99.9	99.9	99.8	0	82.9	61.6	0.1	0	14.2	14.1	0	0	0	0	0	0	99.7	99.9	0	0.2	99.5	99.9	0.1	0	0	
G	0	0	99.9	0	0	99.9	0.1	0	0	0	0	0.1	0	0	0	0.3	0.2	0.5	0	0	0.1	99.9	0.1	0.1	0	0	0.1	0	0	0	0	99.9	100	
T	0.2	0.1	0	0	0	0	0	0.3	0	0	0.2	0	17	38.2	99.8	0	85.3	85.2	99.9	0	0	0	0	0	0.2	0.1	0	99.7	0.4	0	99.8	0	0	

178. B 3G PDCD1 Ex.1 SD + coBE4 mRNA 4 μg

	C_{-10}	C_{-9}	G_{-8}	C_{-7}	C_{-6}	G_{-5}	A_{-4}	C_{-3}	C_{-2}	C_{-1}	C_1	A_2	C_3	C_4	T_5	A_6	C_7	C_8	T_9	A_{10}	A_{11}	G_{12}	A_{13}	A_{14}	C_{15}	C_{16}	A_{17}	T_{18}	C_{19}	C_{20}	T_{21}	G_{22}	G_{23}	Indel
A	0	0	0.1	0	0	0.2	99.9	0	0	0	0	99.9	0	0.2	0	99.9	0.5	0.6	0	99.9	99.9	0	99.9	99.9	0	0	99.9	0	0	0	0	0	0	1.8
C	99.9	99.9	0	99.9	99.9	0	0	99.8	99.9	99.9	99.9	0	85.5	63.4	0.1	0	15.4	15.6	0	0	0	0	0	0	99.9	100	0	0.1	99.5	99.9	0.1	0	0	
G	0	0	99.9	0	0	99.8	0.1	0	0	0	0	0.1	0	0.2	0	0.1	0.4	0.7	0.1	0	0.1	100	0.1	0.1	0	0	0.1	0	0	0	0	99.9	99.9	
T	0.1	0.1	0	0.1	0.1	0	0	0.2	0.1	0.1	0.1	0	14.4	36.1	99.9	0	83.7	83.2	99.9	0	0	0	0	0	0.1	0	0	99.8	0.5	0.1	99.8	0	0	

179. B 3G PDCD1 Ex.1 SD + coBE4 mRNA 4 μg

	C_{-10}	C_{-9}	G_{-8}	C_{-7}	C_{-6}	G_{-5}	A_{-4}	C_{-3}	C_{-2}	C_{-1}	C_1	A_2	C_3	C_4	T_5	A_6	C_7	C_8	T_9	A_{10}	A_{11}	G_{12}	A_{13}	A_{14}	C_{15}	C_{16}	A_{17}	T_{18}	C_{19}	C_{20}	T_{21}	G_{22}	G_{23}	Indel
A	0	0	0.1	0	0	0.1	99.8	0	0	0	0	99.8	0	0.4	0	99.9	0.7	0.6	0	99.4	99.8	0	99.9	99.8	0	0	99.8	0.1	0	0	0	0	0	2.3
C	99.8	99.9	0	99.9	99.9	0	0.1	99.8	99.9	99.9	99.9	0.1	88	68.6	0.1	0.1	24.1	24.3	0	0.1	0.1	0	0	0.1	99.9	99.9	0.1	0.2	99.7	99.9	0.1	0	0	
G	0	0	99.9	0	0	99.8	0.1	0	0	0	0	0.1	0	0.1	0.1	0	0.3	0.8	0.1	0.5	0.1	99.9	0.1	0.1	0	0	0.1	0.1	0	0	0	99.9	100	
T	0.2	0.1	0	0	0.1	0.1	0	0.2	0.1	0.1	0.1	0	11.9	30.9	99.8	0	74.9	74.3	99.9	0	0	0	0	0	0.1	0	0	99.7	0.3	0.1	99.8	0	0	

180. A 3G PDCD1 Ex.1 SD + Cas9 mRNA 1.5 μg

	C_{-10}	C_{-9}	G_{-8}	C_{-7}	C_{-6}	G_{-5}	A_{-4}	C_{-3}	C_{-2}	C_{-1}	C_1	A_2	C_3	C_4	T_5	A_6	C_7	C_8	T_9	A_{10}	A_{11}	G_{12}	A_{13}	A_{14}	C_{15}	C_{16}	A_{17}	T_{18}	C_{19}	C_{20}	T_{21}	G_{22}	G_{23}	Indel
A	0	0.1	0	0.2	0.1	0.1	99.8	0	0.1	0	0.1	99.7	0.2	0.1	0.2	99.4	0.3	0.3	0.2	98.6	99.2	0.1	98.9	98.5	0.1	0.5	98.2	0.4	0.2	0.2	0.1	0.1	0.4	81
C	99.8	99.8	0	99.7	99.8	0.1	0.1	99.8	99.8	99.9	99.8	0.2	99.6	99.8	0.2	0.3	98.9	99.3	0.4	0.1	0.2	0.3	0.5	0.7	99.7	98.9	1.1	0.3	99.5	99.8	0.1	0	0.1	
G	0.1	0	99.9	0.1	0	99.7	0.1	0	0	0	0	0.1	0.1	0.1	0.2	0.3	0.1	0	0.2	0.7	0.5	99.5	0.4	0.4	0.2	0.1	0.3	0.4	0.2	0	0.2	99.7	99.3	
T	0	0	0	0	0	0.1	0	0.1	0.1	0	0.1	0	0.1	0.1	99.4	0	0.7	0.4	99.2	0.5	0	0.2	0.2	0.4	0	0.5	0.4	98.9	0.2	0	99.6	0.1	0.2	

181. B 3G PDCD1 Ex.1 SD + Cas9 mRNA 1.5 μg

	C_{-10}	C_{-9}	G_{-8}	C_{-7}	C_{-6}	G_{-5}	A_{-4}	C_{-3}	C_{-2}	C_{-1}	C_1	A_2	C_3	C_4	T_5	A_6	C_7	C_8	T_9	A_{10}	A_{11}	G_{12}	A_{13}	A_{14}	C_{15}	C_{16}	A_{17}	T_{18}	C_{19}	C_{20}	T_{21}	G_{22}	G_{23}	Indel
A	0	0	0.1	0	0	0.2	99.8	0	0	0	0	99.8	0.1	0.6	0.1	97	3.6	2	0	99.8	99.8	0	99.9	99.9	0	0	99.8	0	0	0	0	0	0.1	7.7
C	99.9	99.9	0	99.9	99.9	0	0	99.9	99.9	99.9	99.9	0	98.6	96.4	0.2	0	76.9	84.2	0.1	0	0	0	0	0	99.8	99.9	0	0.3	99.8	99.9	0.2	0	0	
G	0	0	99.8	0	0	99.8	0.2	0	0	0	0	0.2	0	0.1	0	2.9	1.7	3.1	0	0.2	0.2	99.9	0.1	0.1	0	0	0.1	0	0	0	0	99.9	99.9	
T	0	0.1	0	0.1	0.1	0	0	0	0.1	0.1	0.1	0	1.3	3	99.7	0	17.8	10.7	99.8	0	0	0	0	0	0.1	0	0	99.7	0.2	0	99.8	0	0	

182. A 3G PDCD1 Ex.1 SD + Cas9 mRNA 1.5 μg

	C_{-10}	C_{-9}	G_{-8}	C_{-7}	C_{-6}	G_{-5}	A_{-4}	C_{-3}	C_{-2}	C_{-1}	C_1	A_2	C_3	C_4	T_5	A_6	C_7	C_8	T_9	A_{10}	A_{11}	G_{12}	A_{13}	A_{14}	C_{15}	C_{16}	A_{17}	T_{18}	C_{19}	C_{20}	T_{21}	G_{22}	G_{23}	Indel
A	0	0	0.1	0	0	0.1	99.7	0	0	0	0	99.8	0.1	0	0.1	98.9	0.5	0.4	0.2	99	99.2	0	98.4	98.5	0.1	0.4	97.9	0.3	0	0	0.1	0.1	0.1	77.3
C	99.9	99.8	0.1	99.9	99.9	0	0	99.9	99.8	99.9	99.9	0	99.6	99.4	0.3	0.1	97.3	97.9	0.2	0.1	0.2	0.4	0.3	0.6	99.5	99.2	1.1	0.3	99.6	99.8	0.1	0	0	
G	0	0	99.8	0	0	99.8	0.1	0	0	0	0	0.1	0	0	0.2	0.9	0.2	0.4	0.1	0.6	0.6	99.5	0.8	0.4	0.3	0.2	0.5	0.2	0.3	0.1	0.1	99.8	99.8	
T	0	0.1	0	0	0.1	0.1	0.2	0	0.1	0	0.1	0.1	0.2	0.6	99.5	0.1	2	1.3	99.5	0.3	0	0.1	0.5	0.4	0.1	0.2	0.5	99.2	0	0.1	99.6	0	0	

183. B 3G PDCD1 Ex.1 SD + Cas9 mRNA 1.5 μg

	C_{-10}	C_{-9}	G_{-8}	C_{-7}	C_{-6}	G_{-5}	A_{-4}	C_{-3}	C_{-2}	C_{-1}	C_1	A_2	C_3	C_4	T_5	A_6	C_7	C_8	T_9	A_{10}	A_{11}	G_{12}	A_{13}	A_{14}	C_{15}	C_{16}	A_{17}	T_{18}	C_{19}	C_{20}	T_{21}	G_{22}	G_{23}	Indel
A	0	0	0.2	0	0	0.1	99.9	0	0	0.1	0.1	99.8	0.1	0.1	0.1	99.5	0.5	0.1	0.2	98.8	98.8	0.3	98.6	98.5	0.1	0.4	98.3	0.2	0.1	0	0.2	0.3	0.2	85.4
C	99.8	99.8	0.1	99.8	99.8	0	0	99.8	99.9	99.8	99.8	0	99.6	99.5	0.2	0	98	98.7	0.2	0.1	0.3	0.4	0.4	0.9	99.6	98.7	0.8	0.2	99.1	99.5	0.2	0.1	0	
G	0.1	0.1	99.8	0	0	99.8	0.1	0	0	0	0	0.1	0.1	0	0.3	0.4	0.4	0.3	0.1	0.6	0.8	99.3	0.8	0.2	0.2	0.2	0.7	0.6	0.6	0.1	0.2	99.6	99.7	
T	0.1	0.1	0	0.1	0.2	0	0	0.1	0.1	0.1	0.2	0	0.2	0.4	99.4	0	1.2	0.9	99.4	0.5	0	0.1	0.3	0.4	0.1	0.7	0.2	99	0.2	0.4	99.4	0	0.1	

184. A 3G PDCD1 Ex.1 SD + Cas9 mRNA 4 μg

	C_{-10}	C_{-9}	G_{-8}	C_{-7}	C_{-6}	G_{-5}	A_{-4}	C_{-3}	C_{-2}	C_{-1}	C_1	A_2	C_3	C_4	T_5	A_6	C_7	C_8	T_9	A_{10}	A_{11}	G_{12}	A_{13}	A_{14}	C_{15}	C_{16}	A_{17}	T_{18}	C_{19}	C_{20}	T_{21}	G_{22}	G_{23}	Indel
A	0.1	0	0.1	0.1	0.1	0.3	99.5	0.2	0.2	0.1	0.1	99.4	0.1	0	0	99.3	0.2	0.1	0.2	98.9	99	0.3	98	98.1	0.1	0.4	97.5	0.6	0.2	0	0.5	0	0.1	77
C	99.8	99.8	0	99.9	99.7	0	0.3	99.6	99.8	99.9	99.8	0.4	99.7	99.5	0.2	0.5	98.8	98.8	0.4	0.3	0.6	0.4	0.7	1.1	99.8	99.2	1.4	0.7	99.8	99.9	0.2	0	0	
G	0	0	99.8	0	0	99.5	0.1	0	0	0	0	0.1	0.1	0	0.3	0.1	0.2	0.1	0.6	0.5	0.3	99.2	1	0.1	0.1	0.2	0.6	1	0	0	0.4	99.8	99.7	
T	0	0.1	0.1	0	0.2	0.2	0	0.2	0	0	0.1	0	0.1	0.4	99.4	0	0.9	0.9	98.8	0.3	0.1	0.1	0.3	0.6	0	0.3	0.5	97.8	0	0.1	98.9	0.2	0.1	

185. B 3G PDCD1 Ex.1 SD + Cas9 mRNA 4 μg

	C_{-10}	C_{-9}	G_{-8}	C_{-7}	C_{-6}	G_{-5}	A_{-4}	C_{-3}	C_{-2}	C_{-1}	C_1	A_2	C_3	C_4	T_5	A_6	C_7	C_8	T_9	A_{10}	A_{11}	G_{12}	A_{13}	A_{14}	C_{15}	C_{16}	A_{17}	T_{18}	C_{19}	C_{20}	T_{21}	G_{22}	G_{23}	Indel
A	0	0.1	0.1	0	0.1	0	99.4	0	0	0.1	0	99.6	0	0.1	0	98.9	0.2	0.2	0	99	99.4	0.1	99	98.7	0.2	0	98.6	0.2	0	0	0.3	0	0.2	75.5
C	99.9	99.5	0	99.7	99.5	0.1	0.2	99.9	99.9	99.8	99.9	0.2	99.6	99.7	0.2	0.2	97.7	98.3	0.3	0.2	0.2	0.2	0.2	0.9	99.6	99.8	0.9	0.6	99.8	99.9	0.1	0	0	
G	0	0.3	99.8	0.2	0.4	99.8	0.1	0.1	0.1	0	0	0.1	0	0	0.1	0.9	0.3	0.3	0.1	0.6	0.3	99.7	0.4	0.2	0.1	0.1	0.4	0.3	0	0	0.1	99.9	99.8	
T	0	0	0.1	0.1	0.1	0	0.3	0	0	0	0	0.1	0.3	0.2	99.7	0	1.9	1.2	99.5	0.2	0	0	0.5	0.2	0.1	0.1	0.1	98.9	0.1	0	99.4	0	0.1	

186. A 3G PDCD1 Ex.1 SD + Cas9 mRNA 4 μg

	C_{-10}	C_{-9}	G_{-8}	C_{-7}	C_{-6}	G_{-5}	A_{-4}	C_{-3}	C_{-2}	C_{-1}	C_1	A_2	C_3	C_4	T_5	A_6	C_7	C_8	T_9	A_{10}	A_{11}	G_{12}	A_{13}	A_{14}	C_{15}	C_{16}	A_{17}	T_{18}	C_{19}	C_{20}	T_{21}	G_{22}	G_{23}	Indel
A	0	0	0.1	0.1	0	0.1	99.7	0	0.1	0.1	0	99.7	0	0.2	0.1	99.7	0.2	0.2	0.1	99.1	99.2	0	98.6	99	0.1	0.4	98.9	0.3	0	0	0.2	0	0.2	84.7
C	99.9	99.8	0	99.8	99.7	0	0	99.9	99.9	99.8	99.8	0	99.5	99	0.1	0	97.5	97.7	0.2	0.1	0.1	0.3	0.2	0.5	99.6	99.3	0.7	0.5	99.4	99.9	0.1	0	0	
G	0.1	0.2	99.8	0.1	0.2	99.8	0.1	0	0	0	0	0.1	0	0	0.1	0.2	0.2	0.2	0.2	0.4	0.6	99.6	0.8	0.2	0.3	0	0.2	0.3	0.2	0	0.1	99.9	99.7	
T	0	0	0	0.1	0	0.1	0.2	0	0	0	0.1	0.1	0.4	0.8	99.7	0.1	2.2	1.9	99.5	0.4	0.1	0	0.4	0.3	0.1	0.2	0.2	99	0.3	0.1	99.6	0	0.1	

187. B 3G PDCD1 Ex.1 SD + Cas9 mRNA 4 μg

	C_{-10}	C_{-9}	G_{-8}	C_{-7}	C_{-6}	G_{-5}	A_{-4}	C_{-3}	C_{-2}	C_{-1}	C_1	A_2	C_3	C_4	T_5	A_6	C_7	C_8	T_9	A_{10}	A_{11}	G_{12}	A_{13}	A_{14}	C_{15}	C_{16}	A_{17}	T_{18}	C_{19}	C_{20}	T_{21}	G_{22}	G_{23}	Indel
A	0	0	0.2	0	0	0.1	99.6	0	0	0	0	99.8	0	0.1	0.3	99.4	0.2	0.1	0.2	99.1	98.6	0.4	98.3	98.7	0.1	0.4	97.7	0.5	0	0	0.1	0.3	0.3	88.3
C	99.8	99.7	0	99.8	99.7	0	0	99.9	100	99.7	99.7	0	99.7	99.6	0.2	0.1	98.6	98.9	0.2	0.1	0.4	0.3	0.2	0.6	99.3	98.9	0.8	0.3	99.4	99.9	0.1	0	0	
G	0.2	0.1	99.8	0	0.1	99.8	0.2	0	0	0	0.1	0.1	0.2	0.1	0.2	0.4	0.4	0.4	0.1	0.4	1.1	99.4	1.2	0.3	0.5	0.4	0.6	0.3	0.5	0	0.1	99.5	99.6	
T	0	0.2	0	0.1	0.1	0.1	0.2	0	0	0.2	0.1	0.1	0.1	0.2	99.3	0	0.7	0.5	99.5	0.4	0	0	0.4	0.3	0.1	0.4	0.8	98.9	0.1	0.1	99.7	0.2	0.1	

188. A 3G PDCD1 Ex.1 SD + Cas9 RNP

	C_{-10}	C_{-9}	G_{-8}	C_{-7}	C_{-6}	G_{-5}	A_{-4}	C_{-3}	C_{-2}	C_{-1}	C_1	A_2	C_3	C_4	T_5	A_6	C_7	C_8	T_9	A_{10}	A_{11}	G_{12}	A_{13}	A_{14}	C_{15}	C_{16}	A_{17}	T_{18}	C_{19}	C_{20}	T_{21}	G_{22}	G_{23}	Indel
A	0	0	0.1	0	0	0.1	99.8	0.1	0.1	0.1	0.1	99.7	0.1	0.1	0.1	99.6	0.2	0.1	0.1	99.2	98.7	0.4	98	98.7	0.2	0.6	97.3	1.7	0.1	0.1	0.3	0.4	0.4	76.2
C	99.9	99.9	0.1	99.8	99.8	0	0	99.9	99.9	99.8	99.8	0.1	99.8	99.6	0.2	0	99	99.3	0.3	0.1	0.6	0.2	0.2	0.5	99.3	98.6	0.9	0.5	98.6	99.5	0.2	0.1	0.1	
G	0	0	99.8	0	0	99.8	0.1	0	0	0.1	0	0.2	0	0	0.2	0.3	0.2	0.1	0.2	0.6	0.7	99.3	1	0.4	0.4	0	0.7	1.1	0.8	0.2	0.2	99.3	99.5	
T	0	0.1	0.1	0.1	0.2	0.1	0	0	0	0	0	0	0.2	0.3	99.5	0	0.6	0.5	99.5	0.2	0.1	0	0.8	0.4	0.1	0.8	1	96.7	0.4	0.3	99.3	0.2	0.1	

189. B 3G PDCD1 Ex.1 SD + Cas9 RNP

	C_{-10}	C_{-9}	G_{-8}	C_{-7}	C_{-6}	G_{-5}	A_{-4}	C_{-3}	C_{-2}	C_{-1}	C_1	A_2	C_3	C_4	T_5	A_6	C_7	C_8	T_9	A_{10}	A_{11}	G_{12}	A_{13}	A_{14}	C_{15}	C_{16}	A_{17}	T_{18}	C_{19}	C_{20}	T_{21}	G_{22}	G_{23}	Indel
A	0	0	0.2	0	0	0	99.8	0	0	0.1	0	99.7	0.1	0.1	0.1	99.3	0.2	0.1	0	99.2	99	0.2	98.4	98.8	0.1	0.5	98	1.2	0.1	0	0.1	0.4	0.2	70.1
C	99.8	99.8	0	99.9	99.7	0	0.1	100	99.8	99.8	99.8	0.1	99.5	99.6	0.4	0.1	98.8	99.1	0.3	0.1	0.4	0.4	0.3	0.6	99.5	98.6	0.9	0.4	99.4	99.6	0.3	0	0	
G	0.2	0.1	99.8	0	0.1	99.9	0.1	0	0.1	0.1	0	0.2	0.2	0.1	0.1	0.5	0.3	0.1	0.1	0.5	0.5	99.4	0.6	0.3	0.3	0	0.6	0.8	0.3	0.1	0.2	99.5	99.7	
T	0	0.1	0.1	0	0.2	0	0	0	0	0	0	0	0.2	0.2	99.4	0.1	0.7	0.7	99.6	0.2	0	0	0.7	0.3	0.1	0.8	0.6	97.7	0.2	0.3	99.4	0.1	0.1	

190. A 3G B2M Ex.1 SD + Pulse

	G_{-10}	G_{-9}	T_{-8}	A_{-7}	G_{-6}	G_{-5}	A_{-4}	G_{-3}	A_{-2}	G_{-1}	A_1	C_2	T_3	C_4	A_5	C_6	G_7	C_8	T_9	G_{10}	G_{11}	A_{12}	T_{13}	A_{14}	G_{15}	C_{16}	C_{17}	T_{18}	C_{19}	C_{20}	A_{21}	G_{22}	G_{23}	Indel
A	0	0	0	99.8	0	0	99.7	0	99.8	0	99.8	0	0	0	99.9	0	0	0	0	0	0	99.8	0	99.8	0	0	0	0	0	0	99.8	0	0.1	0.7
C	0	0	0.1	0	0	0	0	0	0	0	0	100	0.1	99.8	0	99.8	0	99.9	0.1	0	0	0	0.1	0	0	99.9	99.9	0.1	99.9	99.9	0	0	0	
G	100	100	0	0.1	100	100	0.2	100	0.2	100	0.2	0	0.1	0	0.1	0	100	0	0.1	99.9	99.9	0.2	0	0.1	100	0	0	0.1	0	0	0.1	100	99.9	
T	0	0	99.9	0	0	0	0	0	0	0	0	0	99.8	0.1	0	0.2	0	0	99.8	0.1	0	0	99.9	0	0	0.1	0	99.7	0	0	0	0	0	

191. B 3G B2M Ex.1 SD + Pulse

	G_{-10}	G_{-9}	T_{-8}	A_{-7}	G_{-6}	G_{-5}	A_{-4}	G_{-3}	A_{-2}	G_{-1}	A_1	C_2	T_3	C_4	A_5	C_6	G_7	C_8	T_9	G_{10}	G_{11}	A_{12}	T_{13}	A_{14}	G_{15}	C_{16}	C_{17}	T_{18}	C_{19}	C_{20}	A_{21}	G_{22}	G_{23}	Indel
A	0	0	0	99.9	0	0	99.8	0	99.8	0	99.8	0	0	0	99.9	0	0	0	0	0	0	99.8	0	99.9	0	0	0	0	0	0	99.8	0	0	0
C	0	0	0.1	0	0	0	0	0	0	0	0	100	0.1	99.8	0	99.7	0	99.9	0.1	0	0	0	0.1	0	0	99.9	99.9	0.1	100	100	0	0	0	
G	100	100	0	0.1	100	100	0.2	100	0.2	100	0.2	0	0	0	0.1	0	100	0	0	100	100	0.2	0	0.1	100	0	0	0	0	0	0.1	100	100	
T	0	0	99.9	0	0	0	0	0	0	0	0	0	99.9	0.2	0	0.2	0	0	99.9	0	0	0	99.9	0	0	0	0	99.8	0	0	0	0	0	

192. A 3G B2M Ex.1 SD + Pulse

	G_{-10}	G_{-9}	T_{-8}	A_{-7}	G_{-6}	G_{-5}	A_{-4}	G_{-3}	A_{-2}	G_{-1}	A_1	C_2	T_3	C_4	A_5	C_6	G_7	C_8	T_9	G_{10}	G_{11}	A_{12}	T_{13}	A_{14}	G_{15}	C_{16}	C_{17}	T_{18}	C_{19}	C_{20}	A_{21}	G_{22}	G_{23}	Indel
A	0	0	0	99.9	0.2	0.3	99.7	0.2	99.8	0.2	99.8	0	0	0.1	99.9	0.3	0	0	0	0.1	0	99.8	0	99.9	0	0	0	0	0	0	99.8	0	0.1	1.1
C	0	0	0.1	0	0	0	0	0	0	0	0	99.8	0.1	95.6	0	95.1	0	99.8	0.1	0	0	0	0.1	0	0	99.9	99.9	0.1	99.9	99.9	0	0	0	
G	100	99.9	0	0.1	99.8	99.7	0.2	99.8	0.2	99.8	0.2	0	0	0.2	0.1	0.1	99.9	0	0	99.9	99.9	0.1	0	0.1	99.9	0	0	0	0	0	0.1	100	99.9	
T	0	0	99.9	0	0	0	0	0	0	0	0	0.1	99.9	4.2	0	4.6	0	0.2	99.8	0	0	0	99.8	0	0	0	0	99.8	0.1	0	0	0	0	

193. B 3G B2M Ex.1 SD + Pulse

	G_{-10}	G_{-9}	T_{-8}	A_{-7}	G_{-6}	G_{-5}	A_{-4}	G_{-3}	A_{-2}	G_{-1}	A_1	C_2	T_3	C_4	A_5	C_6	G_7	C_8	T_9	G_{10}	G_{11}	A_{12}	T_{13}	A_{14}	G_{15}	C_{16}	C_{17}	T_{18}	C_{19}	C_{20}	A_{21}	G_{22}	G_{23}	Indel
A	0	0	0	99.9	0	0	99.8	0	99.8	0	99.8	0	0	0	99.8	0	0.1	0	0	0	0	99.8	0	99.9	0	0	0	0	0	0	99.8	0	0.1	0
C	0	0	0.1	0	0	0	0	0	0	0	0	100	0.1	99.7	0	99.6	0	99.9	0.1	0	0	0	0.1	0	0	99.9	99.9	0.1	99.9	100	0	0	0	
G	99.9	100	0	0.1	99.9	99.9	0.2	99.9	0.2	100	0.2	0	0.1	0	0.2	0	99.9	0	0.1	99.9	99.9	0.2	0	0.1	100	0	0	0.1	0	0	0.1	100	99.9	
T	0	0	99.9	0	0	0	0	0	0	0	0	0	99.8	0.2	0	0.3	0	0.1	99.8	0	0	0	99.9	0	0	0	0	99.8	0	0	0	0	0	

194. A 3G B2M Ex.1 SD + Pulse

	G_{-10}	G_{-9}	T_{-8}	A_{-7}	G_{-6}	G_{-5}	A_{-4}	G_{-3}	A_{-2}	G_{-1}	A_1	C_2	T_3	C_4	A_5	C_6	G_7	C_8	T_9	G_{10}	G_{11}	A_{12}	T_{13}	A_{14}	G_{15}	C_{16}	C_{17}	T_{18}	C_{19}	C_{20}	A_{21}	G_{22}	G_{23}	Indel
A	0	0	0	99.8	0	0	99.8	0	99.7	0	99.7	0	0	0	99.8	0	0	0	0	0	0	99.8	0	99.9	0	0	0	0	0	0	99.8	0	0	0
C	0	0	0.1	0	0	0	0	0	0	0	0	100	0.1	99.8	0	99.8	0	99.9	0.1	0	0	0	0.1	0	0	99.9	99.9	0.1	99.9	99.9	0	0	0	
G	99.9	100	0	0.1	100	100	0.2	100	0.2	100	0.2	0	0	0	0.1	0	100	0	0	100	100	0.2	0	0.1	100	0	0	0	0	0	0.1	100	100	
T	0	0	99.9	0	0	0	0	0	0	0	0	0	99.9	0.2	0	0.2	0	0	99.8	0	0	0	99.9	0	0	0	0	99.8	0	0	0	0	0	

195. B 3G B2M Ex.1 SD + Pulse

	G_{-10}	G_{-9}	T_{-8}	A_{-7}	G_{-6}	G_{-5}	A_{-4}	G_{-3}	A_{-2}	G_{-1}	A_1	C_2	T_3	C_4	A_5	C_6	G_7	C_8	T_9	G_{10}	G_{11}	A_{12}	T_{13}	A_{14}	G_{15}	C_{16}	C_{17}	T_{18}	C_{19}	C_{20}	A_{21}	G_{22}	G_{23}	Indel
A	0	0	0	99.9	0.1	0.1	99.8	0.1	99.8	0	99.8	0	0	0	99.8	0	0	0	0	0	0	99.8	0	99.9	0	0	0	0	0	0	99.8	0	0	0.7
C	0	0	0.1	0	0	0	0	0	0	0	0	99.9	0	98.5	0	98.4	0	99.9	0.1	0	0	0	0.1	0	0	99.9	99.9	0.1	99.9	99.9	0	0	0	
G	100	100	0	0.1	99.9	99.8	0.2	99.9	0.2	99.9	0.2	0	0	0	0.1	0	100	0	0	100	100	0.2	0	0.1	100	0	0	0	0	0	0.2	100	100	
T	0	0	99.9	0	0	0	0	0	0	0	0	0	99.9	1.5	0	1.6	0	0.1	99.9	0	0	0	99.9	0	0	0.1	0	99.8	0	0	0	0	0	

196. A 3G B2M Ex.1 SD + Pulse

	G_{-10}	G_{-9}	T_{-8}	A_{-7}	G_{-6}	G_{-5}	A_{-4}	G_{-3}	A_{-2}	G_{-1}	A_1	C_2	T_3	C_4	A_5	C_6	G_7	C_8	T_9	G_{10}	G_{11}	A_{12}	T_{13}	A_{14}	G_{15}	C_{16}	C_{17}	T_{18}	C_{19}	C_{20}	A_{21}	G_{22}	G_{23}	Indel
A	0	0	0	99.9	0	0.1	99.7	0	99.8	0	99.8	0	0	0	99.9	0	0	0	0	0	0	99.8	0	99.9	0	0	0	0	0	0	99.8	0	0	0.2
C	0	0	0.1	0	0	0	0	0	0	0	0	99.9	0	99.3	0	99.2	0	99.9	0.1	0	0	0	0.1	0	0	99.9	99.9	0.1	99.9	99.9	0	0	0	
G	100	100	0	0.1	99.9	99.9	0.2	100	0.2	100	0.2	0	0	0	0.1	0	99.9	0	0	100	100	0.2	0	0.1	100	0	0	0	0	0	0.2	100	100	
T	0	0	99.9	0	0	0	0	0	0	0	0	0	99.9	0.7	0	0.7	0	0	99.9	0	0	0	99.9	0	0	0	0	99.8	0	0	0	0	0	

197. B 3G B2M Ex.1 SD + Pulse

	G_{-10}	G_{-9}	T_{-8}	A_{-7}	G_{-6}	G_{-5}	A_{-4}	G_{-3}	A_{-2}	G_{-1}	A_1	C_2	T_3	C_4	A_5	C_6	G_7	C_8	T_9	G_{10}	G_{11}	A_{12}	T_{13}	A_{14}	G_{15}	C_{16}	C_{17}	T_{18}	C_{19}	C_{20}	A_{21}	G_{22}	G_{23}	Indel
A	0	0	0	99.9	0	0.1	99.8	0.1	99.8	0	99.8	0	0	0	99.9	0	0	0	0	0	0	99.8	0	99.9	0	0	0	0	0	0	99.8	0	0	0.2
C	0	0	0.1	0	0	0	0	0	0	0	0	99.9	0	99.1	0	99	0	99.9	0.1	0	0	0	0.1	0	0	99.9	99.9	0.1	99.9	99.9	0	0	0	
G	100	100	0	0.1	99.9	99.9	0.2	99.9	0.2	100	0.2	0	0	0	0.1	0	99.9	0	0	100	100	0.2	0	0.1	99.9	0	0	0	0	0	0.1	100	100	
T	0	0	99.9	0	0	0	0	0	0	0	0	0	99.9	0.9	0	1	0	0.1	99.9	0	0	0	99.9	0	0	0	0	99.8	0.1	0	0	0	0	

198. A 3G B2M Ex.1 SD + BE3 conc. mRNA 3 μg

	G_{-10}	G_{-9}	T_{-8}	A_{-7}	G_{-6}	G_{-5}	A_{-4}	G_{-3}	A_{-2}	G_{-1}	A_1	C_2	T_3	C_4	A_5	C_6	G_7	C_8	T_9	G_{10}	G_{11}	A_{12}	T_{13}	A_{14}	G_{15}	C_{16}	C_{17}	T_{18}	C_{19}	C_{20}	A_{21}	G_{22}	G_{23}	Indel
A	0	0	0	99.8	0.1	0.4	99.6	0.2	99.7	0.1	99.8	0.2	0.1	1.7	99.5	9.6	0.1	0	0	0.1	0	99.7	0.1	99.9	0	0	0.1	0.1	0.1	0	99.8	0	0	12.5
C	0	0	0.1	0	0	0.1	0.1	0.1	0.1	0	0	99.4	0.2	63.3	0.2	47.1	0	99.3	0.2	0	0	0.1	0.1	0	0	99.9	99.8	0.2	99.7	99.9	0	0	0	
G	99.9	99.9	0	0.1	99.7	99.4	0.2	99.7	0.2	99.8	0.2	0.1	0.1	3.5	0.1	4.3	99.8	0.1	0.1	99.8	99.9	0.2	0	0.1	99.8	0	0	0.1	0	0	0.2	100	99.9	
T	0	0	99.8	0	0.2	0.1	0	0	0	0	0	0.3	99.6	31.5	0.2	39	0	0.5	99.7	0	0	0	99.8	0	0.2	0.1	0	99.6	0.2	0	0	0	0	

199. B 3G B2M Ex.1 SD + BE3 conc. mRNA 3 μg

	G_{-10}	G_{-9}	T_{-8}	A_{-7}	G_{-6}	G_{-5}	A_{-4}	G_{-3}	A_{-2}	G_{-1}	A_1	C_2	T_3	C_4	A_5	C_6	G_7	C_8	T_9	G_{10}	G_{11}	A_{12}	T_{13}	A_{14}	G_{15}	C_{16}	C_{17}	T_{18}	C_{19}	C_{20}	A_{21}	G_{22}	G_{23}	Indel
A	0.1	0	0	99.8	0.2	0.5	99.7	0.3	99.7	0.1	99.7	0.2	0.1	1.8	99.5	10.1	0	0.1	0	0.1	0	99.7	0	99.9	0	0	0	0	0	0	99.8	0	0.1	9.9
C	0	0	0.1	0	0	0.2	0	0.1	0	0.1	0.1	99.4	0.2	56.3	0.1	39.1	0	99.3	0.2	0	0.1	0.1	0.1	0	0	99.9	99.9	0.2	99.6	99.9	0	0	0	
G	99.9	99.9	0	0.1	99.8	99.2	0.2	99.6	0.2	99.8	0.3	0.1	0	4.1	0.1	4.1	99.9	0.1	0	99.8	99.9	0.2	0	0.1	99.8	0	0	0	0.1	0	0.2	100	99.9	
T	0	0	99.9	0	0.1	0.1	0	0	0	0	0	0.3	99.7	37.8	0.3	46.7	0	0.5	99.8	0	0	0	99.8	0	0.1	0.1	0	99.8	0.2	0	0	0	0	

200. A 3G B2M Ex.1 SD + BE4 conc. mRNA 3 μg

	G_{-10}	G_{-9}	T_{-8}	A_{-7}	G_{-6}	G_{-5}	A_{-4}	G_{-3}	A_{-2}	G_{-1}	A_1	C_2	T_3	C_4	A_5	C_6	G_7	C_8	T_9	G_{10}	G_{11}	A_{12}	T_{13}	A_{14}	G_{15}	C_{16}	C_{17}	T_{18}	C_{19}	C_{20}	A_{21}	G_{22}	G_{23}	Indel
A	0	0.1	0	99.8	0.3	1.1	99.5	0.5	99.6	0.4	99.7	0.2	0.1	1.2	99.6	6.4	0	0.1	0	0	0	99.7	0	99.9	0	0.1	0.2	0.1	0.1	0.1	99.8	0	0	7
C	0	0	0.1	0.1	0	0.1	0.2	0.1	0.1	0.1	0.1	99.4	0.2	60.8	0.1	48.1	0	99	0.1	0	0.1	0.1	0.1	0	0	99.8	99.8	0.2	99.5	99.9	0	0	0	
G	99.9	99.9	0.1	0.1	99.6	98.8	0.2	99.4	0.3	99.4	0.2	0	0.2	2.6	0.1	2.1	99.9	0	0.2	99.9	99.9	0.2	0.1	0.1	99.9	0	0	0.2	0.1	0	0.1	100	99.9	
T	0	0	99.8	0.1	0	0	0	0	0	0.1	0	0.4	99.5	35.5	0.2	43.5	0	0.8	99.7	0	0	0	99.8	0	0	0	0	99.5	0.3	0	0	0	0	

201. B 3G B2M Ex.1 SD + BE4 conc. mRNA 3 μg

	G_{-10}	G_{-9}	T_{-8}	A_{-7}	G_{-6}	G_{-5}	A_{-4}	G_{-3}	A_{-2}	G_{-1}	A_1	C_2	T_3	C_4	A_5	C_6	G_7	C_8	T_9	G_{10}	G_{11}	A_{12}	T_{13}	A_{14}	G_{15}	C_{16}	C_{17}	T_{18}	C_{19}	C_{20}	A_{21}	G_{22}	G_{23}	Indel
A	0.1	0.1	0	99.8	0.4	1.1	99.7	0.8	99.8	0.4	99.8	0.2	0	1	99.7	5.8	0	0.1	0	0.1	0	99.7	0	99.9	0	0	0	0	0	0	99.8	0	0	5.8
C	0	0	0.1	0	0	0.1	0	0.1	0	0.1	0	99.3	0.2	59	0	45.7	0	99.1	0.1	0	0.1	0.1	0.1	0	0	99.9	99.9	0.1	99.6	99.9	0	0	0	
G	99.9	99.9	0	0.1	99.6	98.6	0.2	99	0.2	99.5	0.2	0.1	0	2.6	0.1	2.4	99.9	0.1	0	99.9	99.8	0.2	0	0.1	99.9	0	0	0	0	0	0.1	100	99.9	
T	0	0	99.9	0	0	0.1	0	0.1	0	0	0	0.5	99.8	37.5	0.2	46.1	0.1	0.7	99.8	0	0	0	99.8	0	0.1	0.1	0	99.8	0.3	0	0	0	0	

202. A 3G B2M Ex.1 SD + BE4 RNP

	G_{-10}	G_{-9}	T_{-8}	A_{-7}	G_{-6}	G_{-5}	A_{-4}	G_{-3}	A_{-2}	G_{-1}	A_1	C_2	T_3	C_4	A_5	C_6	G_7	C_8	T_9	G_{10}	G_{11}	A_{12}	T_{13}	A_{14}	G_{15}	C_{16}	C_{17}	T_{18}	C_{19}	C_{20}	A_{21}	G_{22}	G_{23}	Indel
A	0.2	0.2	0	99.8	1.6	4.1	99.7	2.9	99.8	1.6	99.8	0.1	0	1	99.8	2.7	0	0.1	0	0.1	0.1	99.8	0	99.8	0	0.1	0.1	0	0	0	99.8	0	0	8
C	0	0	0.1	0	0	0.1	0	0.2	0	0.2	0	98.5	0.1	21.4	0	11.4	0	97.2	0.1	0	0.1	0.1	0.1	0	0	99.8	99.8	0.1	98.8	99.9	0	0	0	
G	99.8	99.8	0	0.1	98.4	95.6	0.2	96.9	0.2	98.1	0.2	0.2	0	2.6	0.1	0.9	99.9	0.2	0	99.9	99.8	0.2	0	0.1	99.9	0	0	0	0.1	0	0.1	100	100	
T	0	0	99.9	0	0	0.1	0	0.1	0	0	0	1.1	99.9	75.1	0.1	85	0	2.5	99.9	0	0	0	99.8	0	0	0.1	0.1	99.8	1.1	0.1	0	0	0	

203. B 3G B2M Ex.1 SD + BE4 RNP

	G_{-10}	G_{-9}	T_{-8}	A_{-7}	G_{-6}	G_{-5}	A_{-4}	G_{-3}	A_{-2}	G_{-1}	A_1	C_2	T_3	C_4	A_5	C_6	G_7	C_8	T_9	G_{10}	G_{11}	A_{12}	T_{13}	A_{14}	G_{15}	C_{16}	C_{17}	T_{18}	C_{19}	C_{20}	A_{21}	G_{22}	G_{23}	Indel
A	0.2	0.1	0	99.8	1.7	4.3	99.7	2.8	99.7	1.8	99.8	0.2	0	1.1	99.8	3.6	0	0.1	0	0.1	0.1	99.8	0	99.8	0	0	0	0	0.1	0	99.8	0	0	4.5
C	0	0	0.1	0	0	0.2	0	0.2	0	0.3	0	98.6	0.1	18.5	0	7.1	0	97	0.1	0	0	0.1	0.1	0	0	99.9	99.8	0.1	98.8	99.9	0	0	0	
G	99.8	99.8	0	0.1	98.2	95.4	0.2	97	0.2	97.9	0.2	0.2	0	2.3	0.1	0.9	99.9	0.2	0	99.9	99.9	0.1	0	0.1	99.9	0	0	0	0.1	0	0.1	100	99.9	
T	0	0	99.9	0	0.1	0.1	0	0	0	0	0	1	99.8	78.1	0.2	88.4	0	2.7	99.9	0	0	0	99.9	0	0.1	0.1	0.1	99.8	1.1	0.1	0	0	0	

204. A 3G B2M Ex.1 SD + coBE4 mRNA 1.5 μg

	G_{-10}	G_{-9}	T_{-8}	A_{-7}	G_{-6}	G_{-5}	A_{-4}	G_{-3}	A_{-2}	G_{-1}	A_1	C_2	T_3	C_4	A_5	C_6	G_7	C_8	T_9	G_{10}	G_{11}	A_{12}	T_{13}	A_{14}	G_{15}	C_{16}	C_{17}	T_{18}	C_{19}	C_{20}	A_{21}	G_{22}	G_{23}	Indel
A	0.2	0.1	0	99.8	2.1	6.3	99.7	3.7	99.8	1.9	99.8	0.1	0	0.1	99.9	0.3	0	0	0	0	0.1	99.8	0	99.8	0	0	0	0	0	0	99.8	0	0	3.5
C	0	0	0.1	0	0	0.1	0	0.1	0	0.1	0	98.1	0.1	14.6	0	8.2	0	96.9	0.1	0	0	0	0.1	0	0	99.9	99.8	0.1	98	99.8	0	0	0	
G	99.8	99.8	0	0.1	97.9	93.6	0.2	96.2	0.2	98	0.2	0.1	0	0.4	0.1	0.2	100	0	0	99.9	99.9	0.1	0	0.1	99.9	0	0	0	0	0	0.2	100	99.9	
T	0	0	99.9	0	0	0	0	0	0	0	0	1.8	99.9	84.8	0	91.3	0	3.1	99.9	0	0	0	99.9	0	0	0.1	0.1	99.8	2	0.2	0	0	0	

205. B 3G B2M Ex.1 SD + coBE4 mRNA 1.5 μg

	G_{-10}	G_{-9}	T_{-8}	A_{-7}	G_{-6}	G_{-5}	A_{-4}	G_{-3}	A_{-2}	G_{-1}	A_1	C_2	T_3	C_4	A_5	C_6	G_7	C_8	T_9	G_{10}	G_{11}	A_{12}	T_{13}	A_{14}	G_{15}	C_{16}	C_{17}	T_{18}	C_{19}	C_{20}	A_{21}	G_{22}	G_{23}	Indel
A	0.2	0.1	0	99.9	1.6	5.4	99.8	2.9	99.8	1.6	99.8	0.1	0	0.2	99.9	0.5	0	0	0	0	0	99.8	0	99.9	0	0	0	0	0	0	99.9	0	0	2
C	0	0	0.1	0	0	0.1	0	0.1	0	0.1	0	98.4	0.1	16.5	0	7.8	0	97.4	0.1	0	0	0	0.1	0	0	99.9	99.9	0.2	98.5	99.9	0	0	0	
G	99.8	99.9	0	0.1	98.4	94.5	0.2	97	0.2	98.3	0.2	0.1	0	0.8	0.1	0.4	100	0.1	0	100	99.9	0.2	0	0.1	99.9	0	0	0	0	0	0.1	99.9	100	
T	0	0	99.9	0	0	0	0	0	0	0	0	1.4	99.9	82.5	0	91.3	0	2.5	99.9	0	0	0	99.9	0	0	0.1	0.1	99.8	1.4	0.1	0	0	0	

206. A 3G B2M Ex.1 SD + coBE4 mRNA 4 μg

	G_{-10}	G_{-9}	T_{-8}	A_{-7}	G_{-6}	G_{-5}	A_{-4}	G_{-3}	A_{-2}	G_{-1}	A_1	C_2	T_3	C_4	A_5	C_6	G_7	C_8	T_9	G_{10}	G_{11}	A_{12}	T_{13}	A_{14}	G_{15}	C_{16}	C_{17}	T_{18}	C_{19}	C_{20}	A_{21}	G_{22}	G_{23}	Indel
A	0.2	0.2	0	99.9	2.3	6.4	99.7	4.1	99.8	2.4	99.7	0	0	0.1	99.9	0.2	0	0	0	0	0	99.8	0	99.9	0	0	0.1	0	0	0	99.8	0	0	2.7
C	0	0	0.1	0	0	0.1	0	0	0	0.1	0	98	0.1	26.9	0	23.3	0	96.9	0.1	0	0	0	0.1	0	0	99.9	99.8	0.2	98.2	99.9	0	0	0	
G	99.7	99.8	0	0.1	97.6	93.5	0.2	95.8	0.2	97.6	0.2	0	0	0.2	0.1	0.1	99.9	0	0	99.9	99.9	0.2	0	0.1	99.9	0	0	0	0	0	0.2	100	100	
T	0	0	99.9	0	0	0	0	0	0	0	0	2	99.9	72.8	0	76.4	0	3	99.9	0	0	0	99.9	0	0	0.1	0.1	99.8	1.7	0.1	0	0	0	

207. B 3G B2M Ex.1 SD + coBE4 mRNA 4 μg

	G_{-10}	G_{-9}	T_{-8}	A_{-7}	G_{-6}	G_{-5}	A_{-4}	G_{-3}	A_{-2}	G_{-1}	A_1	C_2	T_3	C_4	A_5	C_6	G_7	C_8	T_9	G_{10}	G_{11}	A_{12}	T_{13}	A_{14}	G_{15}	C_{16}	C_{17}	T_{18}	C_{19}	C_{20}	A_{21}	G_{22}	G_{23}	Indel
A	0.2	0.2	0	99.8	1.8	6.9	99.7	3.8	99.7	2	99.7	0.2	0	0.2	99.9	0.8	0.1	0	0	0	0	99.7	0	99.8	0	0	0	0	0	0	99.8	0	0	2.2
C	0	0	0.1	0	0	0.1	0	0	0	0.1	0	97.8	0.1	16.4	0	9.2	0	97.2	0.1	0	0	0	0.1	0	0	99.8	99.9	0.2	98.4	99.8	0	0	0	
G	99.8	99.8	0	0.2	98.2	93	0.2	96.1	0.2	97.9	0.3	0.1	0	0.4	0.1	0.1	99.9	0	0	99.9	100	0.2	0	0.1	99.9	0	0	0	0	0	0.2	100	99.9	
T	0	0	99.8	0	0	0	0	0.1	0.1	0	0	1.9	99.8	82.9	0	89.9	0	2.7	99.8	0	0	0	99.9	0	0	0.1	0.1	99.7	1.6	0.2	0	0	0	

208. A 3G B2M Ex.1 SD + coBE4 mRNA 4 μg

	G_{-10}	G_{-9}	T_{-8}	A_{-7}	G_{-6}	G_{-5}	A_{-4}	G_{-3}	A_{-2}	G_{-1}	A_1	C_2	T_3	C_4	A_5	C_6	G_7	C_8	T_9	G_{10}	G_{11}	A_{12}	T_{13}	A_{14}	G_{15}	C_{16}	C_{17}	T_{18}	C_{19}	C_{20}	A_{21}	G_{22}	G_{23}	Indel
A	0.2	0.2	0	99.8	2.6	8.8	99.8	4.8	99.8	2.5	99.8	0	0	0.1	99.9	0.1	0	0	0	0	0	99.8	0	99.9	0	0	0	0	0	0	99.8	0	0	0.8
C	0	0	0.1	0	0	0.1	0	0	0	0.1	0	97.5	0.1	8.4	0	5.1	0	96.2	0.1	0	0	0	0.1	0	0	99.9	99.9	0.1	96.8	99.8	0	0	0	
G	99.8	99.8	0	0.1	97.3	91.1	0.2	95.1	0.2	97.4	0.2	0	0	0.3	0	0.1	100	0	0	100	99.9	0.1	0	0.1	99.9	0	0	0	0	0	0.2	100	99.9	
T	0	0	99.9	0	0	0	0	0	0	0	0	2.5	99.9	91.2	0	94.6	0	3.8	99.9	0	0	0	99.9	0	0	0.1	0.1	99.8	3.1	0.2	0	0	0	

209. B 3G B2M Ex.1 SD + coBE4 mRNA 4 μg

	G_{-10}	G_{-9}	T_{-8}	A_{-7}	G_{-6}	G_{-5}	A_{-4}	G_{-3}	A_{-2}	G_{-1}	A_1	C_2	T_3	C_4	A_5	C_6	G_7	C_8	T_9	G_{10}	G_{11}	A_{12}	T_{13}	A_{14}	G_{15}	C_{16}	C_{17}	T_{18}	C_{19}	C_{20}	A_{21}	G_{22}	G_{23}	Indel
A	0.2	0.2	0	99.9	2.2	6.9	99.7	3.6	99.8	2.1	99.8	0	0	0.1	99.9	0.2	0	0	0	0	0	99.8	0	99.9	0	0	0	0	0	0	99.8	0	0.1	1.5
C	0	0	0.1	0	0	0.1	0	0	0	0.1	0	98	0.1	10	0	4.9	0	96.3	0.1	0	0	0	0.1	0	0	99.9	99.8	0.1	97.6	99.8	0	0	0	
G	99.8	99.8	0	0.1	97.7	93	0.2	96.4	0.2	97.7	0.2	0	0	0.3	0.1	0.1	99.9	0	0	99.9	99.9	0.2	0	0.1	99.9	0	0	0	0	0	0.1	100	99.9	
T	0	0	99.9	0	0	0	0	0	0	0	0	1.9	99.9	89.6	0	94.7	0	3.6	99.8	0	0	0	99.9	0	0	0.1	0.1	99.8	2.4	0.2	0	0	0	

210. B 3G B2M Ex.1 SD + coBE4 mRNA 4 μg

	G_{-10}	G_{-9}	T_{-8}	A_{-7}	G_{-6}	G_{-5}	A_{-4}	G_{-3}	A_{-2}	G_{-1}	A_1	C_2	T_3	C_4	A_5	C_6	G_7	C_8	T_9	G_{10}	G_{11}	A_{12}	T_{13}	A_{14}	G_{15}	C_{16}	C_{17}	T_{18}	C_{19}	C_{20}	A_{21}	G_{22}	G_{23}	Indel
A	0.2	0.2	0	99.9	2.5	7.2	99.6	4	99.7	2.3	99.8	0.1	0	0.2	99.9	0.4	0	0.1	0	0	0	99.8	0	99.9	0	0	0.1	0	0.1	0.1	99.8	0	0	1.2
C	0	0	0	0	0	0	0.2	0	0.1	0	0	98	0.1	20.8	0	14.7	0	97	0.1	0	0	0	0.1	0	0	99.9	99.8	0.2	98.2	99.8	0	0	0	
G	99.8	99.8	0.1	0.1	97.5	92.7	0.2	95.9	0.2	97.6	0.2	0	0.1	0.4	0.1	0.2	99.9	0.1	0.1	99.9	99.9	0.1	0	0.1	100	0	0	0.2	0	0	0.1	100	99.9	
T	0	0	99.8	0	0	0.1	0	0	0	0	0	1.8	99.8	78.6	0	84.7	0	2.8	99.8	0	0	0	99.8	0	0	0.1	0.1	99.6	1.7	0.1	0	0	0	

211. A 3G B2M Ex.1 SD + Cas9 mRNA 1.5 μg

	G_{-10}	G_{-9}	T_{-8}	A_{-7}	G_{-6}	G_{-5}	A_{-4}	G_{-3}	A_{-2}	G_{-1}	A_1	C_2	T_3	C_4	A_5	C_6	G_7	C_8	T_9	G_{10}	G_{11}	A_{12}	T_{13}	A_{14}	G_{15}	C_{16}	C_{17}	T_{18}	C_{19}	C_{20}	A_{21}	G_{22}	G_{23}	Indel
A	0.1	0.1	0.1	99.8	0.1	0.1	99.5	0.1	99.6	0.1	99.5	0.1	0.1	0.2	99.2	0.1	0.1	0.1	0.1	0.3	0.2	99.1	0.3	99.1	0.5	0.7	0.7	0.4	0.2	0.1	99.3	0.1	0.2	95.4
C	0.1	0.1	0.1	0	0	0	0.1	0	0	0.1	0	99.6	0.1	99.4	0.1	99.4	0.1	99.7	0.3	0.1	0.1	0.4	0.2	0.1	0.2	98.5	97.3	0.2	98.8	99.7	0.1	0.1	0.1	
G	99.8	99.8	0	0.1	99.9	99.8	0.3	99.8	0.2	99.8	0.3	0.3	0.1	0	0.2	0.1	99.3	0.1	0.2	99.5	99.6	0.4	0.5	0.4	99	0.2	0.9	0.2	0.2	0.1	0.3	99.7	99.6	
T	0.1	0.1	99.8	0	0	0.1	0.1	0.1	0.1	0	0.2	0	99.6	0.3	0.4	0.4	0.4	0.2	99.4	0.1	0.1	0.2	98.9	0.3	0.3	0.5	1.1	99.2	0.8	0.1	0.4	0.1	0.1	

212. B 3G B2M Ex.1 SD + Cas9 mRNA 1.5 µg

	G_{-10}	G_{-9}	T_{-8}	A_{-7}	G_{-6}	G_{-5}	A_{-4}	G_{-3}	A_{-2}	G_{-1}	A_1	C_2	T_3	C_4	A_5	C_6	G_7	C_8	T_9	G_{10}	G_{11}	A_{12}	T_{13}	A_{14}	G_{15}	C_{16}	C_{17}	T_{18}	C_{19}	C_{20}	A_{21}	G_{22}	G_{23}	Indel
A	0.1	0	0.2	99.8	0.1	0	99.6	0.1	99.6	0.1	99.6	0.1	0	0.2	99.2	0.1	0.1	0	0.1	0.2	0.1	99.3	0.3	99.6	0.4	0.6	0.4	0.2	0.1	0	99.2	0	0	91.6
C	0	0.2	0.1	0	0	0.1	0.1	0	0.1	0.1	0	99.7	0.1	99.5	0.2	99.5	0.2	99.8	0.1	0	0	0.3	0.2	0.2	0.4	98.9	98.1	0.2	99.3	99.8	0.2	0	0.1	
G	99.7	99.7	0	0.1	99.9	99.8	0.2	99.9	0.2	99.8	0.2	0.2	0	0	0.2	0.1	99.4	0.1	0.1	99.7	99.8	0.3	0.2	0.2	99	0.2	0.7	0.2	0.1	0	0.2	99.9	99.8	
T	0.2	0	99.7	0	0	0.1	0.1	0	0.1	0	0.1	0	99.8	0.2	0.4	0.3	0.3	0.1	99.7	0.1	0.1	0.1	99.3	0.1	0.3	0.3	0.7	99.4	0.5	0.1	0.4	0.1	0	

213. A 3G B2M Ex.1 SD + Cas9 mRNA 1.5 µg

	G_{-10}	G_{-9}	T_{-8}	A_{-7}	G_{-6}	G_{-5}	A_{-4}	G_{-3}	A_{-2}	G_{-1}	A_1	C_2	T_3	C_4	A_5	C_6	G_7	C_8	T_9	G_{10}	G_{11}	A_{12}	T_{13}	A_{14}	G_{15}	C_{16}	C_{17}	T_{18}	C_{19}	C_{20}	A_{21}	G_{22}	G_{23}	Indel
A	0.1	0.1	0	99.8	0	0.1	99.6	0.1	99.7	0.1	99.5	0.1	0.1	0.2	99	0.1	0.1	0	0.1	0.4	0.1	99.2	0.3	99.2	0.4	0.6	0.7	0.4	0.1	0.2	99.1	0.2	0.1	95.1
C	0	0	0.1	0	0	0	0	0	0	0	0	99.6	0.1	99.2	0.1	99.2	0.1	99.8	0.2	0.1	0.1	0.3	0.2	0.2	0.3	98.6	96.6	0.3	98.7	99.7	0.1	0.1	0	
G	99.8	99.8	0	0.1	99.9	99.8	0.2	99.8	0.3	99.8	0.3	0.2	0	0.1	0.2	0.1	99.2	0.1	0.1	99.4	99.7	0.4	0.3	0.4	98.8	0.3	1.2	0.1	0.3	0	0.2	99.8	99.9	
T	0	0.1	99.8	0	0	0.1	0.1	0	0.1	0	0.2	0.1	99.8	0.5	0.7	0.6	0.6	0.1	99.6	0.1	0.1	0.1	99.3	0.2	0.4	0.6	1.6	99.3	0.9	0.1	0.6	0	0	

214. B 3G B2M Ex.1 SD + Cas9 mRNA 1.5 µg

	G_{-10}	G_{-9}	T_{-8}	A_{-7}	G_{-6}	G_{-5}	A_{-4}	G_{-3}	A_{-2}	G_{-1}	A_1	C_2	T_3	C_4	A_5	C_6	G_7	C_8	T_9	G_{10}	G_{11}	A_{12}	T_{13}	A_{14}	G_{15}	C_{16}	C_{17}	T_{18}	C_{19}	C_{20}	A_{21}	G_{22}	G_{23}	Indel
A	0	0.1	0.1	99.8	0	0.1	99.6	0.1	99.6	0.1	99.6	0.1	0	0.1	99.3	0.1	0.1	0	0	0.2	0.1	99.2	0.2	99.6	0.3	0.4	0.5	0.4	0	0.1	99.3	0.1	0.1	93
C	0	0.1	0.1	0	0	0.1	0.1	0	0	0	0	99.8	0.1	99.5	0.1	99.4	0	99.8	0.2	0	0	0.4	0.1	0.1	0.2	99.1	97.8	0.1	99.1	99.8	0.1	0	0.1	
G	99.9	99.8	0	0.2	99.9	99.8	0.3	99.8	0.2	99.8	0.3	0.1	0	0	0.2	0.1	99.5	0	0.1	99.7	99.7	0.3	0.2	0.2	99.2	0.2	0.8	0.1	0.1	0	0.2	99.9	99.8	
T	0.1	0.1	99.8	0	0	0	0.1	0	0.1	0.1	0.1	0	99.8	0.3	0.5	0.5	0.4	0.1	99.7	0.1	0.1	0.2	99.4	0.1	0.3	0.3	0.9	99.4	0.7	0	0.5	0	0	

215. A 3G B2M Ex.1 SD + Cas9 mRNA 4 µg

	G_{-10}	G_{-9}	T_{-8}	A_{-7}	G_{-6}	G_{-5}	A_{-4}	G_{-3}	A_{-2}	G_{-1}	A_1	C_2	T_3	C_4	A_5	C_6	G_7	C_8	T_9	G_{10}	G_{11}	A_{12}	T_{13}	A_{14}	G_{15}	C_{16}	C_{17}	T_{18}	C_{19}	C_{20}	A_{21}	G_{22}	G_{23}	Indel
A	0	0.1	0.1	99.8	0	0	99.5	0.1	99.5	0.2	99.5	0.2	0.2	0.3	98.9	0.1	0.1	0.2	0.1	0.3	0.1	99	0.3	99.3	0.7	0.5	0.6	0.4	0.2	0.2	99	0.1	0.1	93.8
C	0.1	0	0	0	0	0.1	0.1	0	0	0	0.1	99.6	0.3	99.5	0.2	99.5	0.1	99.6	0.1	0.2	0.1	0.5	0.2	0.1	0.5	98.6	97.2	0.2	98.8	99.7	0.1	0	0.1	
G	99.8	99.8	0.1	0.2	99.9	99.8	0.4	99.8	0.3	99.8	0.2	0.1	0.2	0	0.2	0.2	99.2	0.1	0.3	99.3	99.6	0.3	0.4	0.3	98.3	0.3	1	0.2	0.2	0	0.2	99.9	99.7	
T	0.1	0.1	99.7	0	0	0.1	0.1	0	0.2	0.1	0.2	0.1	99.3	0.2	0.7	0.2	0.6	0.1	99.5	0.3	0.2	0.2	99.2	0.3	0.5	0.6	1.2	99.2	0.8	0.1	0.7	0	0	

216. B 3G B2M Ex.1 SD + Cas9 mRNA 4 µg

	G_{-10}	G_{-9}	T_{-8}	A_{-7}	G_{-6}	G_{-5}	A_{-4}	G_{-3}	A_{-2}	G_{-1}	A_1	C_2	T_3	C_4	A_5	C_6	G_7	C_8	T_9	G_{10}	G_{11}	A_{12}	T_{13}	A_{14}	G_{15}	C_{16}	C_{17}	T_{18}	C_{19}	C_{20}	A_{21}	G_{22}	G_{23}	Indel
A	0.1	0.2	0.1	99.8	0	0.1	99.6	0.1	99.5	0.1	99.4	0.1	0.1	0.2	99.1	0.1	0.1	0	0	0.2	0.1	99.3	0.4	99.3	0.4	0.9	0.9	0.6	0.2	0.2	99.4	0.1	0.2	96.2
C	0	0	0.1	0	0.1	0	0.1	0.1	0.1	0	0	99.5	0.1	99.6	0.1	99.6	0.1	99.8	0.1	0.1	0.1	0.3	0.1	0.2	0.5	98.4	96.8	0.3	99.3	99.5	0.1	0	0.1	
G	99.8	99.8	0.1	0.1	99.8	99.8	0.3	99.8	0.3	99.9	0.4	0.2	0.1	0	0.3	0	99.5	0	0.3	99.4	99.7	0.3	0.3	0.3	98.5	0.2	0.7	0.3	0.2	0.2	0.3	99.8	99.7	
T	0	0	99.7	0	0	0	0	0	0.1	0	0.2	0.2	99.7	0.1	0.5	0.2	0.3	0.1	99.5	0.2	0.1	0.1	99.2	0.2	0.6	0.6	1.6	98.9	0.4	0.1	0.2	0	0	

217. A 3G B2M Ex.1 SD + Cas9 mRNA 4 µg

	G_{-10}	G_{-9}	T_{-8}	A_{-7}	G_{-6}	G_{-5}	A_{-4}	G_{-3}	A_{-2}	G_{-1}	A_1	C_2	T_3	C_4	A_5	C_6	G_7	C_8	T_9	G_{10}	G_{11}	A_{12}	T_{13}	A_{14}	G_{15}	C_{16}	C_{17}	T_{18}	C_{19}	C_{20}	A_{21}	G_{22}	G_{23}	Indel
A	0.1	0.1	0.1	99.8	0.1	0.2	99.6	0.2	99.7	0.2	99.5	0.1	0.1	0.3	99.1	0.3	0	0	0	0.2	0.1	99.3	0.2	99.4	0.3	0.6	0.5	0.4	0.1	0.1	99.1	0.1	0.1	91.6
C	0	0.1	0.1	0	0	0	0	0.1	0	0.1	0	99.6	0.1	94.7	0.2	93.8	0.1	99.6	0.2	0.1	0	0.3	0.1	0.2	0.2	98.8	97.8	0.2	98.5	99.7	0	0	0.1	
G	99.8	99.8	0	0.1	99.9	99.7	0.2	99.7	0.2	99.8	0.2	0.2	0	0.2	0.2	0.1	99.4	0.1	0.2	99.7	99.7	0.3	0.3	0.2	98.9	0.3	0.9	0.2	0.3	0.1	0.3	99.8	99.8	
T	0	0.1	99.8	0	0	0.1	0.1	0	0.1	0	0.2	0.1	99.8	4.9	0.5	5.8	0.5	0.3	99.6	0.1	0.1	0.1	99.3	0.1	0.5	0.4	0.8	99.2	1.1	0.2	0.5	0	0	

218. B 3G B2M Ex.1 SD + Cas9 mRNA 4 µg

	G_{-10}	G_{-9}	T_{-8}	A_{-7}	G_{-6}	G_{-5}	A_{-4}	G_{-3}	A_{-2}	G_{-1}	A_1	C_2	T_3	C_4	A_5	C_6	G_7	C_8	T_9	G_{10}	G_{11}	A_{12}	T_{13}	A_{14}	G_{15}	C_{16}	C_{17}	T_{18}	C_{19}	C_{20}	A_{21}	G_{22}	G_{23}	Indel
A	0.1	0.1	0.1	99.8	0.1	0.1	99.5	0.1	99.6	0.1	99.5	0.1	0	0.2	99.1	0.1	0.1	0	0.1	0.2	0.1	99.1	0.3	99.4	0.5	0.5	0.6	0.4	0.1	0.1	99.3	0.1	0.1	95
C	0	0	0.1	0	0	0.1	0.1	0.1	0.1	0	0	99.6	0.1	99	0.1	98.8	0	99.7	0.2	0	0	0.4	0.2	0.2	0.4	98.9	97.7	0.2	99	99.8	0.1	0	0	
G	99.8	99.8	0	0.1	99.9	99.7	0.3	99.8	0.3	99.9	0.3	0.2	0	0	0.2	0.1	99.3	0.1	0.2	99.7	99.8	0.3	0.3	0.2	98.8	0.3	0.5	0.1	0.2	0	0.2	99.9	99.8	
T	0	0	99.8	0	0	0.1	0.1	0	0.1	0	0.2	0.1	99.8	0.8	0.5	0.9	0.5	0.1	99.6	0.2	0.1	0.2	99.3	0.2	0.3	0.3	1.2	99.2	0.6	0.1	0.5	0	0.1	

219. A 3G B2M Ex.1 SD + Cas9 RNP

	G_{-10}	G_{-9}	T_{-8}	A_{-7}	G_{-6}	G_{-5}	A_{-4}	G_{-3}	A_{-2}	G_{-1}	A_1	C_2	T_3	C_4	A_5	C_6	G_7	C_8	T_9	G_{10}	G_{11}	A_{12}	T_{13}	A_{14}	G_{15}	C_{16}	C_{17}	T_{18}	C_{19}	C_{20}	A_{21}	G_{22}	G_{23}	Indel
A	0.1	0.1	0.1	99.8	0	0	99.7	0.1	99.7	0.1	99.5	0.1	0.1	0.2	99.1	0.1	0.1	0.1	0	0.2	0.1	99.3	0.4	99.5	0.4	1.2	0.9	0.8	0.1	0.2	99.1	0.1	0.1	90.2
C	0	0.1	0.1	0	0	0	0.1	0	0	0	0	99.7	0.1	99.3	0.1	99.2	0.1	99.7	0.1	0	0.1	0.4	0.1	0.1	0.3	97.9	97	0.2	98.5	99.7	0.1	0	0.1	
G	99.8	99.8	0	0.2	99.9	99.9	0.2	99.9	0.2	99.9	0.3	0.1	0	0.1	0.2	0.1	99.3	0	0.1	99.6	99.7	0.3	0.4	0.2	98.8	0.4	1	0.2	0.4	0.1	0.2	99.9	99.8	
T	0.1	0.1	99.8	0	0	0	0.1	0	0.1	0	0.2	0.1	99.8	0.4	0.6	0.6	0.5	0.2	99.8	0.1	0.1	0.1	99	0.3	0.6	0.5	1.1	98.8	1.1	0.1	0.6	0	0.1	

220. B 3G B2M Ex.1 SD + Cas9 RNP

	G_{-10}	G_{-9}	T_{-8}	A_{-7}	G_{-6}	G_{-5}	A_{-4}	G_{-3}	A_{-2}	G_{-1}	A_1	C_2	T_3	C_4	A_5	C_6	G_7	C_8	T_9	G_{10}	G_{11}	A_{12}	T_{13}	A_{14}	G_{15}	C_{16}	C_{17}	T_{18}	C_{19}	C_{20}	A_{21}	G_{22}	G_{23}	Indel
A	0.1	0.1	0.1	99.8	0	0.1	99.6	0.1	99.6	0.1	99.6	0.1	0.1	0.3	99	0.1	0.1	0	0.1	0.2	0.1	99.1	0.4	99.5	0.4	1.4	0.9	0.6	0.1	0.2	99.1	0.1	0.1	92.5
C	0	0	0.1	0	0	0	0.1	0	0.1	0.1	0	99.6	0.1	98.8	0.1	98.8	0.1	99.6	0.2	0	0.1	0.4	0.2	0.1	0.3	97.9	96.7	0.2	98.7	99.6	0.1	0	0.1	
G	99.9	99.8	0	0.1	99.9	99.8	0.2	99.8	0.3	99.8	0.2	0.2	0.1	0.1	0.2	0.1	99.3	0.1	0.2	99.7	99.7	0.3	0.4	0.2	98.9	0.3	1.1	0.2	0.3	0.1	0.2	99.8	99.8	
T	0	0.1	99.8	0	0	0.1	0.1	0	0.1	0	0.2	0.1	99.7	0.8	0.6	1	0.5	0.2	99.5	0.1	0.1	0.2	99.1	0.2	0.4	0.5	1.3	98.9	0.9	0.1	0.6	0	0	

221. A 3G B2M Ex.1 SD + Pulse

	G_{-10}	G_{-9}	T_{-8}	A_{-7}	G_{-6}	G_{-5}	A_{-4}	G_{-3}	A_{-2}	G_{-1}	A_1	C_2	T_3	C_4	A_5	C_6	G_7	C_8	T_9	G_{10}	G_{11}	A_{12}	T_{13}	A_{14}	G_{15}	C_{16}	C_{17}	T_{18}	C_{19}	C_{20}	A_{21}	G_{22}	G_{23}	Indel
A	0	0	0	99.9	0	0	99.8	0	99.8	0	99.8	0	0	0	99.8	0.1	0	0	0	0	0	99.8	0	99.9	0	0	0	0	0	0	99.8	0	0	0.1
C	0	0	0.1	0	0	0	0	0	0	0	0	99.9	0.1	99.4	0	99.3	0	99.9	0.1	0	0	0	0.1	0	0	99.9	99.9	0.1	99.9	99.9	0	0	0	
G	99.9	99.9	0	0.1	99.9	99.9	0.2	100	0.2	100	0.2	0	0.1	0	0.2	0	100	0	0	99.9	100	0.2	0	0.1	100	0	0	0.1	0	0	0.1	100	99.9	
T	0	0	99.9	0	0	0	0	0	0	0	0	0	99.8	0.5	0	0.6	0	0	99.9	0	0	0	99.9	0	0	0.1	0	99.8	0	0	0	0	0	

222. B 3G B2M Ex.1 SD + Pulse

	G_{-10}	G_{-9}	T_{-8}	A_{-7}	G_{-6}	G_{-5}	A_{-4}	G_{-3}	A_{-2}	G_{-1}	A_1	C_2	T_3	C_4	A_5	C_6	G_7	C_8	T_9	G_{10}	G_{11}	A_{12}	T_{13}	A_{14}	G_{15}	C_{16}	C_{17}	T_{18}	C_{19}	C_{20}	A_{21}	G_{22}	G_{23}	Indel
A	0	0	0	99.9	0.1	0.1	99.8	0.1	99.8	0	99.8	0	0.1	0.1	99.8	0.3	0	0	0	0	0	99.8	0	99.9	0	0	0	0	0	0	99.8	0	0.1	0.4
C	0	0	0.1	0	0	0	0	0	0	0	0	99.9	0.1	97.3	0	96.5	0	99.8	0.1	0	0	0	0.1	0	0	99.9	99.9	0.1	99.9	99.9	0	0	0	
G	100	99.9	0	0.1	99.9	99.8	0.2	99.9	0.2	100	0.2	0	0	0.1	0.1	0.2	99.9	0	0	99.9	99.9	0.2	0	0.1	100	0	0	0.1	0	0	0.1	100	99.9	
T	0	0	99.9	0	0	0	0	0	0	0	0	0	99.8	2.4	0	3	0	0.1	99.9	0	0	0	99.9	0	0	0	0	99.8	0.1	0	0	0	0	

223. A 3G B2M Ex.1 SD + BE4 RNP

	G_{-10}	G_{-9}	T_{-8}	A_{-7}	G_{-6}	G_{-5}	A_{-4}	G_{-3}	A_{-2}	G_{-1}	A_1	C_2	T_3	C_4	A_5	C_6	G_7	C_8	T_9	G_{10}	G_{11}	A_{12}	T_{13}	A_{14}	G_{15}	C_{16}	C_{17}	T_{18}	C_{19}	C_{20}	A_{21}	G_{22}	G_{23}	Indel
A	0.3	0.2	0	99.8	2.5	5.2	99.7	3.4	99.8	2.4	99.7	0.2	0	1.1	99.8	3.5	0	0.1	0	0	0.1	99.8	0	99.9	0	0	0	0	0	0	99.8	0	0.1	5.3
C	0	0	0.1	0	0	0.1	0	0.1	0	0.3	0	98.4	0.1	18.3	0.1	7.1	0	97.3	0.1	0	0	0	0.1	0	0	99.9	99.9	0.1	98.9	99.9	0	0	0	
G	99.7	99.8	0	0.1	97.4	94.7	0.2	96.4	0.2	97.3	0.2	0.2	0.1	2.4	0.1	1	99.9	0.2	0.1	99.9	99.9	0.1	0	0.1	99.9	0	0	0.1	0.1	0	0.1	100	99.9	
T	0	0	99.8	0	0.1	0.1	0	0.1	0	0	0	1.1	99.8	78.3	0.1	88.4	0	2.4	99.8	0	0	0	99.9	0	0	0	0	99.8	1	0.1	0	0	0	

224. B 3G B2M Ex.1 SD + BE4 RNP

	G_{-10}	G_{-9}	T_{-8}	A_{-7}	G_{-6}	G_{-5}	A_{-4}	G_{-3}	A_{-2}	G_{-1}	A_1	C_2	T_3	C_4	A_5	C_6	G_7	C_8	T_9	G_{10}	G_{11}	A_{12}	T_{13}	A_{14}	G_{15}	C_{16}	C_{17}	T_{18}	C_{19}	C_{20}	A_{21}	G_{22}	G_{23}	Indel
A	0.1	0.1	0	99.8	1.7	4.3	99.6	2.5	99.7	1.8	99.7	0.2	0.1	0.9	99.7	3	0	0.1	0	0	0.1	99.8	0	99.9	0	0	0.1	0.1	0.1	0.1	99.8	0	0	4.8
C	0	0	0.1	0	0	0.2	0.1	0.2	0.1	0.1	0	98.3	0.1	21.4	0.1	10.9	0	97.5	0.1	0	0	0	0.1	0	0	99.9	99.8	0.1	98.6	99.9	0	0	0	
G	99.8	99.8	0.1	0.2	98.2	95.4	0.2	97.1	0.2	98	0.2	0.2	0.2	2.6	0.1	1.3	99.9	0.1	0.1	99.9	99.8	0.2	0.1	0.1	99.9	0	0	0.2	0	0	0.2	100	99.9	
T	0.1	0	99.8	0	0.1	0.1	0.1	0.1	0	0	0	1.4	99.7	75.2	0.1	84.8	0	2.3	99.8	0	0.1	0	99.8	0	0	0.1	0.1	99.6	1.3	0.1	0	0	0	

225. A 3G TRAC Ex.3 SA + Pulse

	A_{-10}	A_{-9}	A_{-8}	G_{-7}	T_{-6}	T_{-5}	T_{-4}	A_{-3}	G_{-2}	G_{-1}	T_1	T_2	C_3	G_4	T_5	A_6	T_7	C_8	T_9	G_{10}	T_{11}	A_{12}	A_{13}	A_{14}	A_{15}	C_{16}	C_{17}	A_{18}	A_{19}	G_{20}	A_{21}	G_{22}	G_{23}	Indel
A	99.9	99.9	99.8	0	0	0	0	99.9	0	0	0	0	0.1	0.1	0	99.9	0	0.1	0	0	0	100	99.8	99.9	99.7	0	0	99.9	99.7	0	99.7	0	0	0.1
C	0	0	0	0	0.1	0.1	0	0	0	0	0.2	0.1	99.6	0	0.1	0	0.1	98.8	0.1	0	0	0	0	0	0.1	100	100	0	0	0	0	0	0	
G	0	0	0.2	99.9	0.1	0.1	0	0.1	100	99.9	0	0	0	99.9	0	0	0.1	0.3	0	100	0	0	0.2	0.1	0.2	0	0	0.1	0.2	99.9	0.2	99.9	99.9	
T	0	0	0	0.1	99.9	99.8	99.9	0	0	0	99.8	99.8	0.3	0	99.8	0	99.7	0.8	99.8	0	99.9	0	0	0	0	0	0	0	0	0	0	0	0.1	

226. B 3G TRAC Ex.3 SA + Pulse

	A_{-10}	A_{-9}	A_{-8}	G_{-7}	T_{-6}	T_{-5}	T_{-4}	A_{-3}	G_{-2}	G_{-1}	T_1	T_2	C_3	G_4	T_5	A_6	T_7	C_8	T_9	G_{10}	T_{11}	A_{12}	A_{13}	A_{14}	A_{15}	C_{16}	C_{17}	A_{18}	A_{19}	G_{20}	A_{21}	G_{22}	G_{23}	Indel
A	99.9	99.9	99.7	0	0	0	0	99.9	0	0	0	0	0.1	0	0	99.9	0	0.2	0	0	0	99.9	99.8	99.8	99.7	0	0	99.9	99.7	0	99.7	0	0.1	0
C	0	0	0	0	0.1	0.1	0	0	0	0	0.3	0.1	99.3	0	0.1	0	0.1	97.9	0.1	0	0	0	0	0	0.1	100	100	0	0.1	0	0	0	0	
G	0	0	0.2	99.9	0.1	0.1	0	0.1	99.9	99.9	0	0	0	99.9	0	0	0	0.3	0.1	99.9	0	0	0.2	0.1	0.2	0	0	0.1	0.2	100	0.3	99.9	99.9	
T	0	0	0	0.1	99.8	99.8	99.9	0	0	0	99.7	99.8	0.6	0.1	99.9	0	99.8	1.6	99.8	0	99.9	0	0	0	0	0	0	0	0	0	0	0.1	0	

227. A 3G TRAC Ex.3 SA + BE4 RNP

	A_{-10}	A_{-9}	A_{-8}	G_{-7}	T_{-6}	T_{-5}	T_{-4}	A_{-3}	G_{-2}	G_{-1}	T_1	T_2	C_3	G_4	T_5	A_6	T_7	C_8	T_9	G_{10}	T_{11}	A_{12}	A_{13}	A_{14}	A_{15}	C_{16}	C_{17}	A_{18}	A_{19}	G_{20}	A_{21}	G_{22}	G_{23}	Indel
A	99.9	99.9	99.7	0.2	0	0	0	99.9	0.1	0.1	0	0	0.4	0.1	0	99.9	0.1	3.6	0	0	0	99.9	99.8	99.8	99.7	0.1	0	100	99.8	0	99.7	0	0.1	2.1
C	0	0	0	0	0.1	0.1	0	0	0	0	0.2	0.1	74.6	0	0.1	0	0.1	15.5	0.1	0	0.1	0	0	0	0.1	99.6	99.9	0	0	0	0	0	0	
G	0	0	0.2	99.8	0	0.1	0.1	0	99.9	99.8	0	0	0.1	99.9	0.1	0	0.1	7.6	0	99.9	0	0	0.1	0.1	0.2	0	0	0	0.2	99.9	0.2	99.9	99.8	
T	0	0	0	0.1	99.9	99.8	99.9	0	0	0	99.8	99.8	24.9	0	99.8	0	99.7	73.3	99.9	0	99.9	0	0	0	0	0.3	0	0	0	0	0	0	0.1	

228. B 3G TRAC Ex.3 SA + BE4 RNP

	A_{-10}	A_{-9}	A_{-8}	G_{-7}	T_{-6}	T_{-5}	T_{-4}	A_{-3}	G_{-2}	G_{-1}	T_1	T_2	C_3	G_4	T_5	A_6	T_7	C_8	T_9	G_{10}	T_{11}	A_{12}	A_{13}	A_{14}	A_{15}	C_{16}	C_{17}	A_{18}	A_{19}	G_{20}	A_{21}	G_{22}	G_{23}	Indel
A	99.9	99.9	99.8	0	0	0	0	99.9	0.1	0	0	0	0.6	0	0	99.9	0.1	3.7	0	0.1	0	99.9	99.7	99.9	99.7	0	0	99.9	99.8	0	99.6	0	0	2.1
C	0	0	0	0	0.1	0.1	0	0	0	0	0.2	0.2	76.8	0	0.2	0	0.1	17.4	0.1	0	0.1	0	0	0	0	99.8	99.9	0	0	0	0	0	0	
G	0	0	0.2	99.9	0.1	0.1	0	0	99.9	99.9	0	0	0.1	99.9	0	0.1	0	9.2	0	99.9	0	0	0.2	0.1	0.2	0	0	0.1	0.2	99.9	0.4	99.9	99.8	
T	0.1	0	0	0.1	99.8	99.8	100	0	0	0	99.8	99.8	22.5	0	99.8	0.1	99.8	69.6	99.8	0	99.8	0	0	0	0	0.2	0	0	0	0	0	0	0.1	

229. A 3G TRAC Ex.3 SA + Pulse

	C_{-10}	C_{-9}	G_{-8}	C_{-7}	C_{-6}	G_{-5}	A_{-4}	C_{-3}	C_{-2}	C_{-1}	C_1	A_2	C_3	C_4	T_5	A_6	C_7	C_8	T_9	A_{10}	A_{11}	G_{12}	A_{13}	A_{14}	C_{15}	C_{16}	A_{17}	T_{18}	C_{19}	C_{20}	T_{21}	G_{22}	G_{23}	Indel
A	0	0	0.1	0.1	0	0	99.5	0.1	0	0	0	99.6	0.1	0	0	99.3	0	0	0	99.4	99.6	0	99.6	99.6	0.1	0	99.2	0.1	0	0.1	0	0	0	0.1
C	99.9	99.9	0	99.9	99.9	0	0.3	99.9	99.9	99.9	99.9	0.2	99.9	99.9	0.1	0.5	99.8	99.8	0.1	0.2	0.3	0	0.3	0.2	99.9	99.9	0.5	0.2	99.9	99.9	0.1	0	0	
G	0	0	99.8	0	0	99.6	0.1	0	0	0	0	0.1	0	0	0.3	0.1	0	0	0.4	0.3	0.1	99.7	0.1	0.1	0	0	0.1	0.6	0	0	0.2	99.8	99.9	
T	0	0	0.1	0	0	0.3	0	0	0	0	0	0	0	0.1	99.6	0.1	0.2	0.1	99.5	0	0	0.2	0	0.1	0	0	0.2	99.1	0	0	99.7	0.2	0	

230. B 3G TRAC Ex.3 SA + Pulse

	C_{-10}	C_{-9}	G_{-8}	C_{-7}	C_{-6}	G_{-5}	A_{-4}	C_{-3}	C_{-2}	C_{-1}	C_1	A_2	C_3	C_4	T_5	A_6	C_7	C_8	T_9	A_{10}	A_{11}	G_{12}	A_{13}	A_{14}	C_{15}	C_{16}	A_{17}	T_{18}	C_{19}	C_{20}	T_{21}	G_{22}	G_{23}	Indel
A	0	0	0	0	0	0	99.8	0	0	0	0	99.8	0.1	0	0	99.8	0.1	0.1	0	99.5	99.9	0	99.9	99.8	0	0	99.7	0.1	0	0	0	0	0	0.1
C	99.9	99.9	0	99.9	99.9	0	0.1	100	99.9	99.9	99.9	0.1	99.7	99.3	0.1	0.1	98.6	98.7	0.1	0.1	0.1	0	0	0.1	99.9	99.9	0.2	0.1	99.9	99.9	0.1	0	0	
G	0	0	99.9	0	0	99.9	0.1	0	0	0	0	0.1	0	0	0.1	0	0	0.1	0.1	0.4	0	99.9	0	0	0	0	0	0.1	0	0	0	99.9	100	
T	0	0	0	0	0	0.1	0	0	0	0	0	0	0.3	0.6	99.8	0	1.3	1.1	99.8	0	0	0	0	0	0	0	0	99.7	0	0	99.9	0	0	

231. A 3G TRAC Ex.3 SA + BE4 RNP

	C_{-10}	C_{-9}	G_{-8}	C_{-7}	C_{-6}	G_{-5}	A_{-4}	C_{-3}	C_{-2}	C_{-1}	C_1	A_2	C_3	C_4	T_5	A_6	C_7	C_8	T_9	A_{10}	A_{11}	G_{12}	A_{13}	A_{14}	C_{15}	C_{16}	A_{17}	T_{18}	C_{19}	C_{20}	T_{21}	G_{22}	G_{23}	Indel
A	0	0	0.1	0	0	0.3	99.8	0.1	0	0	0.1	99.8	0.3	1.6	0.2	99.9	4.4	4.5	0	99.4	99.8	0	99.9	99.8	0.1	0	99.8	0.1	0.1	0	0	0	0	9.3
C	99.8	99.8	0	99.9	99.9	0	0	99.8	99.9	99.9	99.8	0.1	86.4	70.6	0.1	0	27.6	33.4	0	0	0.1	0	0	0.1	99.8	99.9	0.1	0.1	99.6	99.9	0.1	0	0	
G	0	0	99.9	0	0	99.6	0.1	0	0	0	0	0.1	0.1	0.2	0	0	1.5	5.2	0.1	0.5	0.1	99.9	0.1	0	0	0	0	0.1	0	0	0	99.9	99.9	
T	0.2	0.1	0	0	0.1	0.1	0	0.2	0	0.1	0.1	0	13.2	27.5	99.7	0.1	66.5	56.9	99.9	0	0	0.1	0	0	0.2	0	0	99.7	0.3	0.1	99.9	0	0	

232. B 3G TRAC Ex.3 SA + BE4 RNP

	C_{-10}	C_{-9}	G_{-8}	C_{-7}	C_{-6}	G_{-5}	A_{-4}	C_{-3}	C_{-2}	C_{-1}	C_1	A_2	C_3	C_4	T_5	A_6	C_7	C_8	T_9	A_{10}	A_{11}	G_{12}	A_{13}	A_{14}	C_{15}	C_{16}	A_{17}	T_{18}	C_{19}	C_{20}	T_{21}	G_{22}	G_{23}	Indel
A	0.1	0	0.1	0	0.1	0.3	99.8	0.2	0.1	0	0	99.8	0.4	1.4	0.2	99.8	4.7	5.1	0	99.4	99.9	0.1	99.8	99.8	0	0	99.7	0	0	0	0	0.1	0	8.9
C	99.9	99.9	0	99.9	99.8	0	0.1	99.5	99.9	99.9	99.8	0.1	87.2	71	0.1	0.1	27.9	34.4	0	0.1	0.1	0	0	0.1	99.9	99.9	0.2	0.1	99.6	99.9	0.1	0	0	
G	0	0	99.9	0	0	99.6	0.1	0	0	0	0	0.1	0	0.4	0	0	2.2	5.7	0.1	0.5	0	99.8	0.1	0	0	0	0.1	0.1	0	0	0.1	99.8	100	
T	0.1	0	0	0	0.2	0.1	0.1	0.3	0	0.1	0.1	0	12.4	27.2	99.7	0	65.3	54.9	99.9	0	0	0.1	0	0	0.1	0	0	99.7	0.3	0.1	99.8	0	0	

233. A B2M Ex.1 SD OnT + Pulse

	G_{-10}	G_{-9}	T_{-8}	A_{-7}	G_{-6}	G_{-5}	A_{-4}	G_{-3}	A_{-2}	G_{-1}	A_1	C_2	T_3	C_4	A_5	C_6	G_7	C_8	T_9	G_{10}	G_{11}	A_{12}	T_{13}	A_{14}	G_{15}	C_{16}	C_{17}	T_{18}	C_{19}	C_{20}	A_{21}	G_{22}	G_{23}	Indel
A	0	0	0	99.9	0	0	99.5	0	99.7	0	99.7	0.3	0.2	0.3	99.8	0.2	0	0.1	0	0.1	0	99.7	0.1	99.8	0	0.1	0.2	0.1	0.1	0.2	99.8	0	0.1	0
C	0	0	0.1	0	0	0	0.2	0	0.1	0	0.1	99.7	0.2	99.7	0.1	99.7	0	99.9	0.1	0	0	0	0.1	0.1	0	99.8	99.8	0.2	99.9	99.8	0	0	0	
G	99.9	99.9	0.3	0.1	100	99.9	0.2	99.9	0.2	99.9	0.2	0	0.5	0	0.1	0	99.9	0	0.4	99.9	100	0.2	0.2	0.1	99.9	0	0	0.3	0	0	0.1	99.9	99.9	
T	0	0	99.6	0	0	0.1	0	0	0	0	0	0	99.2	0	0	0.1	0	0	99.5	0	0	0	99.7	0	0	0	0	99.3	0	0	0	0	0	

234. A B2M Ex.1 SD OT1 + Pulse

	A_{-10}	T_{-9}	T_{-8}	C_{-7}	A_{-6}	G_{-5}	C_{-4}	A_{-3}	T_{-2}	C_{-1}	T_1	C_2	T_3	G_4	C_5	C_6	C_7	C_8	T_9	G_{10}	G_{11}	A_{12}	T_{13}	A_{14}	G_{15}	C_{16}	C_{17}	T_{18}	C_{19}	C_{20}	T_{21}	G_{22}	G_{23}	Indel
A	99.9	0	0	0.1	99.8	0	0	99.9	0	0	0	0.1	0	0	0	0.1	0.1	0	0	0	0	99.8	0	99.9	0	0	0	0	0	0	0	0	0	0
C	0	0	0	99.9	0	0	99.9	0	0.1	99.9	0.1	99.9	0.1	0	99.9	99.8	99.9	99.9	0.1	0	0	0.1	0	0	0	99.9	99.9	0.2	99.9	99.9	0.1	0	0	
G	0	0.1	0	0	0.1	99.9	0	0	0	0	0	0	0	99.9	0	0	0	0	0	99.9	100	0.1	0	0.1	99.9	0	0	0	0	0	0	99.9	99.9	
T	0	99.9	99.9	0	0	0	0	0	99.9	0	99.9	0	99.9	0	0	0	0	0	99.8	0.1	0	0	99.9	0	0	0	0	99.7	0	0	99.9	0.1	0.1	

235. A B2M Ex.1 SD OT2 + Pulse

	T_{-10}	C_{-9}	G_{-8}	G_{-7}	T_{-6}	C_{-5}	C_{-4}	A_{-3}	C_{-2}	G_{-1}	A_1	C_2	T_3	C_4	A_5	C_6	C_7	T_8	T_9	C_{10}	C_{11}	A_{12}	T_{13}	A_{14}	G_{15}	C_{16}	C_{17}	T_{18}	C_{19}	C_{20}	A_{21}	G_{22}	G_{23}	Indel
A	0	0	0	0	0	0	0	99.8	0	0	99.8	0	0	0.1	99.8	0	0	0	0	0	0	99.8	0	99.9	0	0	0	0	0.1	0.1	99.9	0	0.1	0
C	0.1	99.9	0	0	0.1	99.9	99.9	0	99.9	0	0	99.9	0.2	99.9	0.1	99.5	100	0.2	0.1	99.9	99.9	0	0.1	0	0	99.9	99.9	0.1	99.9	99.9	0	0	0	
G	0.1	0	99.9	99.9	0.1	0	0	0.1	0	99.9	0.1	0	0	0	0.1	0	0	0.1	0	0	0	0.1	0	0.1	99.9	0	0	0.1	0	0	0.1	99.9	99.8	
T	99.8	0	0	0.1	99.8	0	0	0	0.1	0.1	0	0	99.8	0	0	0.5	0	99.7	99.9	0	0	0	99.9	0	0	0	0	99.8	0	0	0	0	0.1	

236. A B2M Ex.1 SD OT3 + Pulse

	T_{-10}	C_{-9}	T_{-8}	T_{-7}	G_{-6}	G_{-5}	C_{-4}	A_{-3}	G_{-2}	A_{-1}	G_{1}	C_{2}	T_{3}	C_{4}	C_{5}	T_{6}	G_{7}	C_{8}	T_{9}	G_{10}	C_{11}	A_{12}	T_{13}	A_{14}	G_{15}	C_{16}	C_{17}	T_{18}	C_{19}	C_{20}	T_{21}	G_{22}	G_{23}	Indel
A	0	0	0	0	0	0.1	0.1	99.7	0	99.4	0.1	0.1	0	0	0	0	0	0	0	0	0	99.7	0	99.8	0.1	0.1	0	0	0.1	0	0	0	0	0
C	0	99.9	0.1	0.1	0	0	99.8	0.2	0	0.2	0	99.8	0.3	99.9	100	0.1	0	99.9	0.1	0	99.9	0.1	0	0.1	0	99.8	99.9	0.2	99.9	100	0.1	0	0	
G	0	0	0.1	0	99.7	99.8	0	0.1	99.8	0.3	99.9	0	0.1	0	0	0.1	99.8	0	0.1	99.8	0	0.1	0	0.1	99.8	0	0	0.1	0	0	0	99.8	99.9	
T	99.9	0.1	99.8	99.9	0.2	0.1	0	0	0.1	0.1	0.1	0.1	99.6	0	0	99.8	0.2	0	99.8	0.1	0	0	99.9	0	0.1	0	0	99.6	0	0	99.9	0.1	0.1	

237. A B2M Ex.1 SD OT4 + Pulse

	A_{-10}	A_{-9}	A_{-8}	T_{-7}	G_{-6}	C_{-5}	A_{-4}	G_{-3}	T_{-2}	T_{-1}	T_{1}	C_{2}	T_{3}	C_{4}	A_{5}	C_{6}	T_{7}	G_{8}	T_{9}	G_{10}	G_{11}	T_{12}	T_{13}	A_{14}	G_{15}	C_{16}	C_{17}	T_{18}	C_{19}	C_{20}	T_{21}	G_{22}	G_{23}	Indel
A	100	99.9	99.8	0	0	0	99.9	0	0	0	0	0	0	0	99.8	0	0	0	0	0	0	0	0	99.9	0	0	0.1	0	0	0	0	0.1	0	0
C	0	0	0	0	0	99.9	0	0	0.2	0	0.1	100	0.1	99.9	0	100	0.2	0	0.1	0	0	0.1	0.1	0	0	99.9	99.9	0.2	100	100	0.1	0	0	
G	0	0	0.1	0	99.9	0	0.1	99.9	0	0.1	0	0	0	0	0.1	0	0	99.9	0	99.9	99.9	0	0	0.1	99.9	0	0	0	0	0	0	99.9	99.9	
T	0	0	0	99.9	0	0.1	0	0	99.8	99.9	99.9	0	99.8	0	0	0	99.8	0.1	99.9	0.1	0.1	99.8	99.9	0	0	0.1	0	99.8	0	0	99.8	0	0.1	

238. A B2M Ex.1 SD OT5 + Pulse

	T_{-10}	T_{-9}	T_{-8}	T_{-7}	A_{-6}	T_{-5}	G_{-4}	G_{-3}	C_{-2}	A_{-1}	T_{1}	C_{2}	T_{3}	C_{4}	T_{5}	C_{6}	A_{7}	C_{8}	T_{9}	G_{10}	G_{11}	A_{12}	T_{13}	A_{14}	G_{15}	C_{16}	C_{17}	T_{18}	A_{19}	C_{20}	T_{21}	G_{22}	G_{23}	Indel
A	0	0	0	0	99.9	0	0	0	0.1	99.9	0	0	0	0	0	0	99.8	0	0	0	0	99.8	0	99.9	0	0.1	0.1	0	99.9	0	0	0	0	0
C	0.2	0	0	0	0	0.6	0	0	99.9	0	0.1	99.9	0.2	100	0.2	99.9	0	99.9	0.1	0	0	0	0	0	0	99.8	99.4	0	0	99.9	0.1	0	0	
G	0.1	0.1	0.1	0	0	0	99.9	99.9	0	0	0	0	0	0	0.1	0	0.1	0	0.1	99.9	99.9	0.1	0	0.1	99.9	0	0	0	0.1	0	0	99.9	99.9	
T	99.7	99.8	99.9	100	0	99.3	0	0	0	0	99.8	0	99.8	0	99.8	0	0	0	99.8	0.1	0	0	99.9	0	0	0.1	0.5	99.9	0	0	99.9	0	0	

239. A B2M Ex.1 SD OT6 + Pulse

	T_{-10}	T_{-9}	T_{-8}	T_{-7}	A_{-6}	T_{-5}	G_{-4}	G_{-3}	C_{-2}	A_{-1}	T_{1}	C_{2}	T_{3}	C_{4}	T_{5}	C_{6}	A_{7}	C_{8}	T_{9}	G_{10}	G_{11}	A_{12}	T_{13}	A_{14}	G_{15}	C_{16}	C_{17}	T_{18}	A_{19}	C_{20}	T_{21}	G_{22}	G_{23}	Indel
A	0	0	0	0	99.9	0	0	0	0.1	99.9	0	0	0	0	0	0	99.8	0	0	0	0	99.8	0	99.9	0	0.1	0.1	0	99.9	0	0	0	0	0
C	0.2	0	0	0	0	0.6	0	0	99.8	0	0.1	99.9	0.2	100	0.2	99.9	0	99.9	0.1	0	0	0	0.1	0	0	99.8	99.3	0	0	99.9	0.1	0	0	
G	0.1	0.2	0.1	0	0	0	99.9	99.9	0	0	0	0	0.1	0	0.1	0	0.1	0	0.1	99.9	99.9	0.1	0	0.1	99.9	0	0	0.1	0.1	0	0	99.9	99.9	
T	99.7	99.8	99.9	99.9	0	99.3	0	0	0.1	0	99.8	0	99.8	0	99.8	0	0	0	99.8	0.1	0	0	99.9	0	0	0	0.6	99.9	0	0	99.9	0.1	0	

240. A B2M Ex.1 SD OT7 + Pulse

	T_{-10}	T_{-9}	T_{-8}	T_{-7}	A_{-6}	T_{-5}	G_{-4}	G_{-3}	C_{-2}	A_{-1}	T_{1}	C_{2}	T_{3}	C_{4}	T_{5}	C_{6}	A_{7}	C_{8}	T_{9}	G_{10}	G_{11}	A_{12}	T_{13}	A_{14}	G_{15}	C_{16}	C_{17}	T_{18}	A_{19}	C_{20}	T_{21}	G_{22}	G_{23}	Indel
A	0	0	0	0	99.9	0	0	0	0.1	99.9	0	0.1	0	0	0	0.1	99.9	0.1	0	0	0	99.8	0	99.9	0	0.2	0.2	0	99.9	0.1	0	0	0	0
C	0.2	0	0	0	0	0.6	0	0	99.8	0	0.1	99.8	0.2	99.9	0.2	99.8	0	99.9	0.1	0	0	0	0	0	0	99.8	99.3	0	0	99.9	0.1	0	0	
G	0.2	0.2	0.1	0.1	0	0	99.9	99.9	0	0	0.1	0	0.1	0	0.1	0	0.1	0	0.1	99.9	99.9	0.1	0	0.1	99.9	0	0	0	0.1	0	0	99.9	99.9	
T	99.6	99.7	99.8	99.9	0	99.3	0.1	0	0.1	0	99.8	0	99.7	0	99.7	0	0	0	99.8	0.1	0	0	99.9	0	0	0	0.5	99.9	0	0	99.9	0.1	0.1	

241. A B2M Ex.1 SD OT8 + Pulse

	T_{-10}	A_{-9}	G_{-8}	C_{-7}	T_{-6}	C_{-5}	G_{-4}	G_{-3}	G_{-2}	C_{-1}	A_{1}	G_{2}	C_{3}	C_{4}	A_{5}	C_{6}	C_{7}	C_{8}	T_{9}	G_{10}	G_{11}	A_{12}	G_{13}	A_{14}	G_{15}	C_{16}	C_{17}	T_{18}	C_{19}	C_{20}	G_{21}	G_{22}	G_{23}	Indel
A	0	99.8	0	0	0	0	0	0.1	0	0	99.9	0	0	0	99.9	0	0	0	0	0.1	0	99.7	0	99.5	0.1	0	0	0	0	0	0.1	0	0	0
C	0.1	0	0	99.9	0.1	99.9	0	0	0	99.9	0	0	99.9	100	0	100	100	100	0.1	0	0	0	0	0.1	0	99.9	99.9	0	100	100	0	0	0	
G	0.1	0.2	100	0	0.1	0	100	99.9	100	0	0.1	100	0	0	0	0	0	0	0	99.9	100	0.3	100	0.4	99.9	0	0	0.2	0	0	99.9	100	99.9	
T	99.8	0	0	0	99.7	0.1	0	0	0	0	0	0	0	0	0	0	0	0	99.8	0	0	0	0	0	0	0	0	99.7	0	0	0	0	0	

242. A B2M Ex.1 SD OT9 + Pulse

	A_{-10}	C_{-9}	C_{-8}	A_{-7}	A_{-6}	A_{-5}	A_{-4}	G_{-3}	C_{-2}	A_{-1}	A_{1}	C_{2}	T_{3}	C_{4}	C_{5}	C_{6}	G_{7}	C_{8}	T_{9}	G_{10}	G_{11}	A_{12}	A_{13}	A_{14}	G_{15}	C_{16}	C_{17}	T_{18}	G_{19}	C_{20}	A_{21}	G_{22}	G_{23}	Indel
A	99.7	0.2	0.2	99.8	99.9	99.9	99.8	0.1	0.7	99.9	99.6	0.4	0.3	0	0.1	0.2	0.1	0.1	0.1	0.1	0.1	99.9	99.9	99.7	0.2	0.2	0.4	0.1	0.1	0.5	99.8	0	0	0
C	0.2	99.8	99.7	0.1	0	0	0.1	0	99.2	0	0.2	99.6	2	99.9	99.9	99.7	0.1	99.8	0.2	0	0	0	0	0	0	99.7	99.5	0.2	0	99.4	0	0	0	
G	0.1	0	0	0	0	0	0.1	99.8	0	0	0.1	0	0.9	0	0	0	99.8	0	0.5	99.8	99.8	0.1	0	0.2	99.7	0.1	0	1.2	99.9	0	0.1	99.9	99.9	
T	0	0	0	0	0	0	0	0.1	0	0	0	0	96.8	0	0	0.1	0.1	0	99.3	0.1	0.1	0	0	0	0.1	0.1	0	98.5	0	0	0	0	0.1	

243. A B2M Ex.1 SD OT10 + Pulse

	T_{-10}	C_{-9}	T_{-8}	C_{-7}	C_{-6}	T_{-5}	T_{-4}	T_{-3}	G_{-2}	A_{-1}	C_{1}	C_{2}	T_{3}	C_{4}	C_{5}	C_{6}	G_{7}	C_{8}	T_{9}	G_{10}	T_{11}	G_{12}	T_{13}	A_{14}	G_{15}	C_{16}	C_{17}	T_{18}	C_{19}	C_{20}	T_{21}	G_{22}	G_{23}	Indel
A	0	0	0	0	0	0	0	0	0	99.6	0	0	0	0	0	0	0.1	0	0	0	0	0	0	99.8	0	0	0.1	0.1	0	0	0	0	0	0
C	0.3	99.9	0.2	99.9	99.9	0.1	0.1	0	0	0.2	99.9	99.9	0.2	99.9	100	99.9	0	99.9	0.1	0	0.1	0	0.1	0	0	99.9	99.8	0.2	99.9	100	0.1	0	0	
G	0	0	0.1	0	0	0	0	0	99.8	0.2	0	0	0.1	0	0	0	99.8	0	0.3	99.9	0.2	99.9	0	0.1	99.9	0	0	0.1	0	0	0.1	99.9	99.8	
T	99.7	0	99.6	0	0	99.9	99.9	99.9	0.1	0	0	0	99.7	0	0	0.1	0.1	0.1	99.6	0.1	99.7	0.1	99.9	0	0	0.1	0.1	99.6	0	0	99.8	0.1	0.2	

244. A B2M Ex.1 SD OnT + Cas9 mRNA

	G_{-10}	G_{-9}	T_{-8}	A_{-7}	G_{-6}	G_{-5}	A_{-4}	G_{-3}	A_{-2}	G_{-1}	A_{1}	C_{2}	T_{3}	C_{4}	A_{5}	C_{6}	G_{7}	C_{8}	T_{9}	G_{10}	G_{11}	A_{12}	T_{13}	A_{14}	G_{15}	C_{16}	C_{17}	T_{18}	C_{19}	C_{20}	A_{21}	G_{22}	G_{23}	Indel
A	0.1	0	0	99.8	0	0	99.6	0	99.7	0.1	99.7	0.1	0	0.1	99.6	0.1	0.1	0	0	0.1	0	99.5	0.1	99.9	0	1	0.8	0.2	0.2	0.2	99.2	0.2	0.1	91.1
C	0	0	0.1	0	0	0.1	0.1	0	0	0	0	99.8	0.1	99.9	0	99.8	0.1	99.9	0.1	0	0.1	0.2	0.1	0	0.1	97.2	97.3	0.4	99.1	99.7	0.1	0.1	0.1	
G	99.9	99.9	0.1	0.1	99.9	99.9	0.2	99.9	0.2	99.9	0.2	0	0.1	0	0.2	0	99.8	0	0.2	99.8	99.8	0.2	0.2	0.1	99.5	1	0.5	0.2	0.1	0	0.2	99.7	99.7	
T	0	0	99.8	0	0	0	0.1	0	0.1	0	0	0	99.7	0	0.1	0.1	0	0.1	99.7	0	0	0.1	99.7	0	0.4	0.7	1.4	99.2	0.6	0.1	0.5	0	0.1	

245. A B2M Ex.1 SD OT1 + Cas9 mRNA

	A_{-10}	T_{-9}	T_{-8}	C_{-7}	A_{-6}	G_{-5}	C_{-4}	A_{-3}	T_{-2}	C_{-1}	T_{1}	C_{2}	T_{3}	G_{4}	C_{5}	C_{6}	C_{7}	C_{8}	T_{9}	G_{10}	G_{11}	A_{12}	T_{13}	A_{14}	G_{15}	C_{16}	C_{17}	T_{18}	C_{19}	C_{20}	T_{21}	G_{22}	G_{23}	Indel
A	99.9	0	0	0.1	99.8	0	0	100	0	0.1	0	0.1	0	0	0	0.1	0.1	0	0	0	0	99.7	0	99.9	0	0	0	0	0.1	0	0	0	0.1	0
C	0	0	0	99.8	0	0	99.9	0	0.1	99.9	0.1	99.9	0	0	99.9	99.8	99.8	99.9	0.1	0	0	0.1	0	0	0	99.9	99.9	0.1	99.9	99.9	0.1	0	0	
G	0	0.1	0	0	0.1	99.9	0	0	0	0	0	0	0	99.9	0	0	0	0	0	99.9	100	0.1	0	0.1	99.9	0	0	0	0	0	0	99.9	99.8	
T	0	99.9	99.9	0	0	0	0	0	99.9	0	99.8	0	99.9	0	0.1	0	0.1	0	99.8	0.1	0	0	99.9	0	0	0	0	99.8	0	0	99.8	0.1	0.1	

246. A B2M Ex.1 SD OT2 + Cas9 mRNA

	T_{-10}	C_{-9}	G_{-8}	G_{-7}	T_{-6}	C_{-5}	C_{-4}	A_{-3}	C_{-2}	G_{-1}	A_{1}	C_{2}	T_{3}	C_{4}	A_{5}	C_{6}	C_{7}	T_{8}	T_{9}	C_{10}	C_{11}	A_{12}	T_{13}	A_{14}	G_{15}	C_{16}	C_{17}	T_{18}	C_{19}	C_{20}	A_{21}	G_{22}	G_{23}	Indel
A	0	0	0.1	0	0	0	0	99.9	0	0	99.9	0	0	0	99.8	0	0	0	0	0	0	99.9	0	99.9	0	0	0	0	0	0	99.9	0	0	0
C	0.1	99.9	0	0	0.1	100	99.9	0	99.9	0	0	100	0.1	99.9	0	99.5	99.9	0.1	0.1	100	100	0	0	0	0	99.9	100	0.1	99.9	99.9	0	0	0	
G	0	0	99.9	99.9	0	0	0	0.1	0	99.9	0.1	0	0	0	0.1	0	0	0	0	0	0	0.1	0	0.1	100	0	0	0.1	0	0	0.1	100	99.9	
T	99.8	0	0	0	99.8	0	0	0	0.1	0	0	0	99.9	0	0	0.4	0	99.8	99.9	0	0	0	99.9	0	0	0	0	99.8	0	0	0	0	0.1	

247. A B2M Ex.1 SD OT3 + Cas9 mRNA

	T_{-10}	C_{-9}	T_{-8}	T_{-7}	G_{-6}	G_{-5}	C_{-4}	A_{-3}	G_{-2}	A_{-1}	G_{1}	C_{2}	T_{3}	C_{4}	C_{5}	T_{6}	G_{7}	C_{8}	T_{9}	G_{10}	C_{11}	A_{12}	T_{13}	A_{14}	G_{15}	C_{16}	C_{17}	T_{18}	C_{19}	C_{20}	T_{21}	G_{22}	G_{23}	Indel
A	0	0	0	0	0	0	0.1	99.8	0	99.5	0	0	0	0	0	0	0	0	0	0.1	0	99.8	0	99.9	0	0.1	0	0	0.1	0	0	0	0	0
C	0	99.9	0.1	0.1	0	0	99.9	0.1	0	0.2	0	99.9	0.3	99.9	100	0.1	0	99.9	0.2	0	99.9	0.1	0	0	0	99.9	99.9	0.2	99.9	100	0.1	0	0	
G	0	0	0	0	99.8	99.9	0	0.1	99.9	0.2	99.9	0	0.1	0	0	0	99.8	0	0.1	99.8	0	0.1	0	0.1	99.9	0	0	0.1	0	0	0	99.9	99.9	
T	100	0	99.9	99.9	0.2	0.1	0	0	0.1	0.1	0	0	99.6	0.1	0	99.8	0.1	0	99.8	0.1	0	0	99.9	0	0.1	0	0	99.7	0	0	99.9	0.1	0.1	

248. A B2M Ex.1 SD OT4 + Cas9 mRNA

	A_{-10}	A_{-9}	A_{-8}	T_{-7}	G_{-6}	C_{-5}	A_{-4}	G_{-3}	T_{-2}	T_{-1}	T_{1}	C_{2}	T_{3}	C_{4}	A_{5}	C_{6}	T_{7}	G_{8}	T_{9}	G_{10}	G_{11}	T_{12}	T_{13}	A_{14}	G_{15}	C_{16}	C_{17}	T_{18}	C_{19}	C_{20}	T_{21}	G_{22}	G_{23}	Indel
A	99.9	99.9	99.8	0	0	0.1	99.9	0	0	0	0	0.1	0	0.1	99.8	0.1	0	0	0	0	0	0	0	99.9	0	0.1	0.1	0	0	0	0	0	0	0
C	0	0	0.1	0	0	99.8	0	0	0.2	0	0.1	99.9	0.1	99.8	0.1	99.9	0.1	0	0.2	0	0	0.1	0	0	0	99.8	99.8	0.2	99.9	100	0.1	0	0	
G	0	0	0	0.1	99.9	0	0.1	99.9	0.1	0	0	0	0	0	0.1	0	0	99.8	0	99.9	99.9	0.1	0	0.1	99.9	0	0	0	0	0	0	99.9	99.8	
T	0	0	0	99.9	0.1	0.1	0	0	99.8	99.9	99.9	0	99.8	0	0	0	99.9	0.1	99.8	0.1	0.1	99.8	99.9	0	0	0.1	0	99.8	0	0	99.8	0.1	0.1	

249. A B2M Ex.1 SD OT5 + Cas9 mRNA

	T_{-10}	T_{-9}	T_{-8}	T_{-7}	A_{-6}	T_{-5}	G_{-4}	G_{-3}	C_{-2}	A_{-1}	T_{1}	C_{2}	T_{3}	C_{4}	T_{5}	C_{6}	A_{7}	C_{8}	T_{9}	G_{10}	G_{11}	A_{12}	T_{13}	A_{14}	G_{15}	C_{16}	C_{17}	T_{18}	A_{19}	C_{20}	T_{21}	G_{22}	G_{23}	Indel
A	0	0	0	0	99.9	0	0	0	0.2	99.9	0.1	0.1	0	0.1	0	0.1	99.8	0.1	0	0	0	99.8	0	99.9	0	0.1	0.2	0	99.9	0.1	0	0	0	0
C	0.2	0	0	0	0	1.2	0	0	99.8	0	0.2	99.8	0.2	99.9	0.2	99.8	0	99.9	0.1	0	0	0	0.1	0	0	99.8	98.8	0.1	0	99.9	0.1	0	0	
G	0.3	0.3	0.2	0.1	0	0	99.9	99.9	0	0	0	0	0.1	0	0.1	0	0.1	0	0.1	99.9	99.9	0.1	0	0.1	100	0	0	0	0	0	0	99.9	99.9	
T	99.5	99.7	99.8	99.9	0	98.7	0.1	0	0.1	0	99.7	0	99.7	0	99.7	0	0	0	99.8	0.1	0	0	99.9	0	0	0.1	1.1	99.9	0	0	99.9	0.1	0.1	

250. A B2M Ex.1 SD OT6 + Cas9 mRNA

	T_{-10}	T_{-9}	T_{-8}	T_{-7}	A_{-6}	T_{-5}	G_{-4}	G_{-3}	C_{-2}	A_{-1}	T_{1}	C_{2}	T_{3}	C_{4}	T_{5}	C_{6}	A_{7}	C_{8}	T_{9}	G_{10}	G_{11}	A_{12}	T_{13}	A_{14}	G_{15}	C_{16}	C_{17}	T_{18}	A_{19}	C_{20}	T_{21}	G_{22}	G_{23}	Indel
A	0	0	0	0	99.9	0	0	0	0.1	99.9	0	0	0	0	0	0	99.9	0	0	0	0	99.8	0	99.9	0	0.1	0.1	0	99.9	0	0	0	0	0
C	0.2	0	0	0	0	1.6	0	0	99.9	0	0.1	99.9	0.2	99.9	0.1	99.9	0	99.9	0.1	0	0	0	0	0	0	99.8	98.4	0	0	99.9	0.1	0	0	
G	0.1	0.1	0.1	0	0	0	99.9	99.9	0	0.1	0	0	0	0	0.1	0	0.1	0	0.1	99.9	99.9	0.1	0	0.1	100	0	0	0	0	0	0	99.9	99.9	
T	99.7	99.8	99.9	99.9	0	98.3	0	0	0	0	99.8	0	99.7	0	99.8	0	0	0	99.8	0	0	0	99.9	0	0	0	1.5	99.9	0	0	99.9	0.1	0	

251. A B2M Ex.1 SD OT7 + Cas9 mRNA

	T_{-10}	T_{-9}	T_{-8}	T_{-7}	A_{-6}	T_{-5}	G_{-4}	G_{-3}	C_{-2}	A_{-1}	T_{1}	C_{2}	T_{3}	C_{4}	T_{5}	C_{6}	A_{7}	C_{8}	T_{9}	G_{10}	G_{11}	A_{12}	T_{13}	A_{14}	G_{15}	C_{16}	C_{17}	T_{18}	A_{19}	C_{20}	T_{21}	G_{22}	G_{23}	Indel
A	0	0	0	0	99.9	0	0	0	0.1	99.9	0	0.1	0	0	0	0.1	99.8	0.1	0	0	0	99.8	0	99.9	0	0.1	0.1	0	99.9	0	0	0	0	0
C	0.2	0	0	0	0	1.1	0	0	99.8	0	0.2	99.9	0.2	99.9	0.2	99.9	0	99.9	0.1	0	0	0	0.1	0	0	99.8	98.9	0	0	99.9	0.1	0	0	
G	0.2	0.2	0.1	0.1	0	0	99.9	99.9	0	0.1	0	0	0.1	0	0.1	0	0.1	0	0.1	99.9	99.9	0.1	0	0.1	99.9	0	0	0.1	0.1	0	0	99.9	99.9	
T	99.7	99.8	99.9	99.9	0	98.9	0.1	0	0.1	0	99.8	0	99.7	0	99.8	0	0	0	99.8	0.1	0	0	99.9	0	0	0	0.9	99.9	0	0	99.9	0.1	0.1	

252. A B2M Ex.1 SD OT8 + Cas9 mRNA

	T_{-10}	A_{-9}	G_{-8}	C_{-7}	T_{-6}	C_{-5}	G_{-4}	G_{-3}	G_{-2}	C_{-1}	A_{1}	G_{2}	C_{3}	C_{4}	A_{5}	C_{6}	C_{7}	C_{8}	T_{9}	G_{10}	G_{11}	A_{12}	G_{13}	A_{14}	G_{15}	C_{16}	C_{17}	T_{18}	C_{19}	C_{20}	G_{21}	G_{22}	G_{23}	Indel
A	0	99.8	0	0	0	0	0	0	0	0	99.9	0.1	0	0	99.9	0	0	0	0	0	0	99.7	0	99.7	0	0	0	0	0	0	0	0	0	0
C	0.1	0	0	100	0.1	99.9	0	0	0	99.9	0	0	99.9	100	0	99.9	99.9	100	0.1	0	0	0	0	0	0	99.9	99.9	0.1	100	100	0	0	0	
G	0	0.1	100	0	0.1	0	99.9	99.9	100	0	0.1	99.9	0	0	0.1	0	0	0	0	100	100	0.2	100	0.2	100	0	0	0.2	0	0	99.9	100	100	
T	99.9	0	0	0	99.8	0	0	0	0	0	0	0	0.1	0	0	0	0	0	99.9	0	0	0	0	0	0	0	0	99.7	0	0	0	0	0	

253. A B2M Ex.1 SD OT9 + Cas9 mRNA

	A_{-10}	C_{-9}	C_{-8}	A_{-7}	A_{-6}	A_{-5}	A_{-4}	G_{-3}	C_{-2}	A_{-1}	A_{1}	C_{2}	T_{3}	C_{4}	C_{5}	C_{6}	G_{7}	C_{8}	T_{9}	G_{10}	G_{11}	A_{12}	A_{13}	A_{14}	G_{15}	C_{16}	C_{17}	T_{18}	G_{19}	C_{20}	A_{21}	G_{22}	G_{23}	Indel
A	99.6	0.1	0.3	99.6	99.7	99.7	99.8	0.1	0.2	100	99.7	0.1	0.1	0	0	0.1	0	0.1	0	0.1	0	99.9	100	99.7	0.4	0.5	1.1	0.1	0	0	99.8	0	0	0
C	0.3	99.8	99.7	0.3	0.2	0.3	0.1	0	99.8	0	0.1	99.9	0.5	100	99.9	99.9	0	99.9	0.2	0	0	0	0	0	0.1	99.4	98.8	0.2	0	99.9	0	0	0	
G	0.1	0	0	0.1	0	0.1	0.1	99.9	0	0	0.2	0	0	0	0	0	99.9	0	0	99.9	99.9	0.1	0	0.3	99.6	0.1	0	0.2	99.9	0	0.1	99.9	99.9	
T	0	0	0	0	0	0	0	0	0	0	0	0	99.4	0	0.1	0	0	0	99.7	0	0	0	0	0	0	0	0	99.5	0	0.1	0	0	0	

254. A B2M Ex.1 SD OT10 + Cas9 mRNA

	T_{-10}	C_{-9}	T_{-8}	C_{-7}	C_{-6}	T_{-5}	T_{-4}	T_{-3}	G_{-2}	A_{-1}	C_{1}	C_{2}	T_{3}	C_{4}	C_{5}	C_{6}	G_{7}	C_{8}	T_{9}	G_{10}	T_{11}	G_{12}	T_{13}	A_{14}	G_{15}	C_{16}	C_{17}	T_{18}	C_{19}	C_{20}	T_{21}	G_{22}	G_{23}	Indel
A	0	0	0	0.1	0.1	0	0	0	0	99.8	0	0	0	0	0	0	0	0	0	0	0	0.1	0	99.8	0.1	0	0.1	0	0	0	0	0	0	0
C	0.3	100	0.3	99.9	99.9	0	0.1	0	0	0.1	99.9	100	0.2	100	99.9	99.9	0	99.9	0.1	0	0.2	0	0.1	0	0	100	99.8	0.1	100	100	0.2	0	0	
G	0	0	0.1	0	0	0.1	0	0	99.9	0.1	0	0	0.1	0	0	0	99.9	0.1	0.3	99.8	0.2	99.9	0	0.1	99.9	0	0	0.2	0	0	0.1	99.9	99.9	
T	99.7	0	99.6	0	0	99.9	99.9	99.9	0.1	0	0.1	0	99.7	0	0	0	0.1	0	99.6	0.1	99.6	0	99.9	0	0	0	0	99.7	0	0	99.7	0	0.1	

255. A B2M Ex.1 SD OnT + BE4 mRNA

	G_{-10}	G_{-9}	T_{-8}	A_{-7}	G_{-6}	G_{-5}	A_{-4}	G_{-3}	A_{-2}	G_{-1}	A_{1}	C_{2}	T_{3}	C_{4}	A_{5}	C_{6}	G_{7}	C_{8}	T_{9}	G_{10}	G_{11}	A_{12}	T_{13}	A_{14}	G_{15}	C_{16}	C_{17}	T_{18}	C_{19}	C_{20}	A_{21}	G_{22}	G_{23}	Indel
A	0.2	0.2	0	99.8	2.2	6.8	99.7	4.6	99.6	2.6	99.7	0.3	0.1	1.6	99.8	7.9	0	0.2	0	0	0.1	99.5	0	99.8	0	0	0	0	0.1	0	99.8	0	0	8.4
C	0	0	0.1	0	0	0.2	0	0.2	0.2	0.3	0.1	97.7	0.1	22	0	15.2	0	96.3	0.1	0	0.1	0.3	0.2	0	0	99.8	99.8	0.2	98	99.7	0	0	0	
G	99.8	99.8	0	0.1	97.8	92.9	0.2	95.1	0.2	97.1	0.2	0.5	0	4.2	0.1	2.4	99.9	0.3	0	99.9	99.7	0.2	0	0.1	99.8	0	0	0	0.1	0	0.2	99.9	99.9	
T	0	0	99.9	0	0	0.1	0	0.1	0	0	0	1.5	99.8	72.1	0.1	74.5	0	3.1	99.9	0	0.1	0	99.7	0	0.1	0.1	0.1	99.8	1.8	0.2	0	0	0	

256. A B2M Ex.1 SD OT1 + BE4 mRNA

	A_{-10}	T_{-9}	T_{-8}	C_{-7}	A_{-6}	G_{-5}	C_{-4}	A_{-3}	T_{-2}	C_{-1}	T_{1}	C_{2}	T_{3}	G_{4}	C_{5}	C_{6}	C_{7}	C_{8}	T_{9}	G_{10}	G_{11}	A_{12}	T_{13}	A_{14}	G_{15}	C_{16}	C_{17}	T_{18}	C_{19}	C_{20}	T_{21}	G_{22}	G_{23}	Indel
A	99.9	0	0.1	0.3	99.8	0	0.2	100	0.1	0.2	0	0.5	0.1	0.1	0.2	0.3	0.2	0.1	0	0.1	0	99.9	0	100	0.1	0.1	0.1	0	0.3	0.1	0	0	0	0
C	0	0	0	99.6	0.1	0	99.7	0	0	99.8	0	99.5	0.1	0	99.8	99.7	99.7	99.9	0.2	0	0	0.1	0	0	0	99.9	99.9	0.2	99.7	99.8	0	0	0	
G	0	0.2	0	0	0.1	99.9	0	0	0.1	0	0.1	0	0.1	99.9	0	0	0	0	0.1	99.8	100	0.1	0	0	99.9	0	0	0.1	0	0	0.1	100	99.8	
T	0	99.8	99.9	0.1	0	0	0	0	99.8	0	99.9	0	99.8	0	0	0.1	0.1	0	99.7	0.1	0	0	99.9	0	0	0	0.1	99.7	0	0.1	99.9	0	0.1	

257. A B2M Ex.1 SD OT2 + BE4 mRNA

	T_{-10}	C_{-9}	G_{-8}	G_{-7}	T_{-6}	C_{-5}	C_{-4}	A_{-3}	C_{-2}	G_{-1}	A_{1}	C_{2}	T_{3}	C_{4}	A_{5}	C_{6}	C_{7}	T_{8}	T_{9}	C_{10}	C_{11}	A_{12}	T_{13}	A_{14}	G_{15}	C_{16}	C_{17}	T_{18}	C_{19}	C_{20}	A_{21}	G_{22}	G_{23}	Indel
A	0	0	0	0	0	0	0	99.9	0	0	99.9	0	0	0	99.9	0	0	0	0	0	0	100	0	99.9	0	0	0	0	0	0	100	0	0	0
C	0	100	0	0	0.1	100	100	0	100	0	0	100	0.1	100	0	100	100	0.1	0	100	100	0	0	0	0	100	100	0	100	100	0	0	0	
G	0	0	100	99.9	0	0	0	0.1	0	100	0	0	0	0	0.1	0	0	0	0	0	0	0	0	0.1	99.9	0	0	0	0	0	0	100	99.9	
T	100	0	0	0	99.9	0	0	0	0	0	0	0	99.9	0	0	0	0	99.9	100	0	0	0	100	0	0	0	0	99.9	0	0	0	0	0	

258. A B2M Ex.1 SD OT3 + BE4 mRNA

	T_{-10}	C_{-9}	T_{-8}	T_{-7}	G_{-6}	G_{-5}	C_{-4}	A_{-3}	G_{-2}	A_{-1}	G_{1}	C_{2}	T_{3}	C_{4}	C_{5}	T_{6}	G_{7}	C_{8}	T_{9}	G_{10}	C_{11}	A_{12}	T_{13}	A_{14}	G_{15}	C_{16}	C_{17}	T_{18}	C_{19}	C_{20}	T_{21}	G_{22}	G_{23}	Indel
A	0	0	0	0	0	0	0	99.8	0	99.6	0	0.1	0	0	0	0	0	0	0	0	0	99.8	0	99.9	0	0.1	0	0	0.1	0	0	0	0	0
C	0	99.9	0	0	0	0	99.9	0.2	0	0.2	0	99.9	0.2	99.9	100	0.1	0	99.9	0.1	0	99.9	0.1	0	0	0	99.9	99.9	0.1	99.9	100	0.1	0	0	
G	0	0	0	0	99.8	99.9	0	0	99.9	0.2	99.9	0	0.1	0	0	0	99.8	0	0.1	99.8	0	0	0	0	99.9	0	0	0.1	0	0	0	99.9	99.9	
T	100	0	99.9	100	0.2	0.1	0	0	0.1	0.1	0.1	0	99.7	0	0	99.9	0.1	0	99.8	0.1	0	0	99.9	0	0.1	0	0	99.7	0	0	99.9	0.1	0.1	

259. A B2M Ex.1 SD OT4 + BE4 mRNA

	A_{-10}	A_{-9}	A_{-8}	T_{-7}	G_{-6}	C_{-5}	A_{-4}	G_{-3}	T_{-2}	T_{-1}	T_{1}	C_{2}	T_{3}	C_{4}	A_{5}	C_{6}	T_{7}	G_{8}	T_{9}	G_{10}	G_{11}	T_{12}	T_{13}	A_{14}	G_{15}	C_{16}	C_{17}	T_{18}	C_{19}	C_{20}	T_{21}	G_{22}	G_{23}	Indel
A	100	99.7	99.5	0	0.1	0	99.9	0	0	0	0	0	0	0	99.9	0	0	0	0	0	0	0	0	99.9	0	0	0	0	0	0	0	0	0	0
C	0	0.2	0.4	0.1	0	99.9	0	0	0.2	0.1	0.1	100	0.2	100	0	100	0.2	0	0.1	0	0	0.2	0.1	0	0	99.9	100	0.2	100	100	0.2	0	0	
G	0	0.1	0.1	0	99.9	0	0.1	100	0	0.1	0	0	0	0	0.1	0	0	99.9	0	100	99.9	0	0	0.1	100	0	0	0	0	0	0	100	100	
T	0	0	0	99.9	0	0.1	0	0	99.7	99.8	99.9	0	99.8	0	0	0	99.8	0.1	99.8	0	0	99.8	99.9	0	0	0.1	0	99.8	0	0	99.8	0	0	

260. A B2M Ex.1 SD OT5 + BE4 mRNA

	T_{-10}	T_{-9}	T_{-8}	T_{-7}	A_{-6}	T_{-5}	G_{-4}	G_{-3}	C_{-2}	A_{-1}	T_{1}	C_{2}	T_{3}	C_{4}	T_{5}	C_{6}	A_{7}	C_{8}	T_{9}	G_{10}	G_{11}	A_{12}	T_{13}	A_{14}	G_{15}	C_{16}	C_{17}	T_{18}	A_{19}	C_{20}	T_{21}	G_{22}	G_{23}	Indel
A	0	0	0	0	100	0	0	0	0.1	100	0	0	0	0	0	0	99.9	0.1	0	0	0	99.9	0	99.9	0	0.1	0.1	0	99.9	0	0	0	0	0
C	0.1	0	0	0	0	0.3	0	0	99.8	0	0.1	99.9	0.1	99.9	0.1	99.9	0	99.9	0	0	0	0	0	0	0	99.8	99.6	0	0	99.9	0.1	0	0	
G	0.1	0.1	0.1	0	0	0	99.9	99.9	0	0	0	0	0.1	0	0.1	0	0.1	0	0.1	99.9	100	0.1	0	0.1	99.9	0	0	0.1	0	0	0	99.9	99.9	
T	99.7	99.8	99.9	100	0	99.6	0	0	0.1	0	99.9	0	99.8	0	99.8	0	0	0	99.9	0.1	0	0	99.9	0	0	0.1	0.3	99.9	0	0	99.9	0.1	0.1	

261. A B2M Ex.1 SD OT6 + BE4 mRNA

	T_{-10}	T_{-9}	T_{-8}	T_{-7}	A_{-6}	T_{-5}	G_{-4}	G_{-3}	C_{-2}	A_{-1}	T_{1}	C_{2}	T_{3}	C_{4}	T_{5}	C_{6}	A_{7}	C_{8}	T_{9}	G_{10}	G_{11}	A_{12}	T_{13}	A_{14}	G_{15}	C_{16}	C_{17}	T_{18}	A_{19}	C_{20}	T_{21}	G_{22}	G_{23}	Indel
A	0	0	0	0	100	0	0	0.1	0.1	100	0.1	0	0	0	0	0.1	99.9	0	0	0.1	0	99.9	0	99.9	0	0.2	0.2	0	99.9	0.1	0	0	0	0
C	0.1	0	0	0	0	0.3	0	0	99.8	0	0.1	99.9	0.1	99.9	0.1	99.9	0	99.9	0	0	0	0	0	0	0	99.7	99.6	0	0	99.8	0	0	0	
G	0.3	0.3	0.2	0.1	0	0	99.9	99.9	0	0	0	0	0.1	0	0.1	0	0.1	0	0.1	99.8	100	0.1	0	0.1	100	0.1	0	0	0	0	0	99.9	99.9	
T	99.6	99.7	99.8	99.9	0	99.7	0	0	0	0	99.8	0	99.8	0	99.8	0	0	0	99.9	0.1	0	0	100	0	0	0.1	0.3	99.9	0	0	99.9	0.1	0.1	

262. A B2M Ex.1 SD OT7 + BE4 mRNA

	T_{-10}	T_{-9}	T_{-8}	T_{-7}	A_{-6}	T_{-5}	G_{-4}	G_{-3}	C_{-2}	A_{-1}	T_{1}	C_{2}	T_{3}	C_{4}	T_{5}	C_{6}	A_{7}	C_{8}	T_{9}	G_{10}	G_{11}	A_{12}	T_{13}	A_{14}	G_{15}	C_{16}	C_{17}	T_{18}	A_{19}	C_{20}	T_{21}	G_{22}	G_{23}	Indel
A	0	0	0	0	100	0	0	0	0	100	0	0	0	0	0	0	99.9	0	0	0	0	99.9	0	99.9	0	0.1	0.1	0	99.9	0	0	0	0	0
C	0.1	0	0	0	0	0.4	0	0	99.9	0	0.1	99.9	0.1	100	0.1	99.9	0	100	0	0	0	0	0	0	0	99.9	99.5	0	0	99.9	0	0	0	
G	0.1	0.1	0.1	0	0	0	99.9	99.9	0	0	0	0	0	0	0	0	0.1	0	0	99.9	100	0.1	0	0.1	100	0	0	0	0	0	0	99.9	99.9	
T	99.8	99.9	99.9	100	0	99.5	0	0	0.1	0	99.9	0	99.9	0	99.9	0	0	0	99.9	0	0	0	99.9	0	0	0	0.4	99.9	0	0	99.9	0	0	

263. A B2M Ex.1 SD OT8 + BE4 mRNA

	T_{-10}	A_{-9}	G_{-8}	C_{-7}	T_{-6}	C_{-5}	G_{-4}	G_{-3}	G_{-2}	C_{-1}	A_{1}	G_{2}	C_{3}	C_{4}	A_{5}	C_{6}	C_{7}	C_{8}	T_{9}	G_{10}	G_{11}	A_{12}	G_{13}	A_{14}	G_{15}	C_{16}	C_{17}	T_{18}	C_{19}	C_{20}	G_{21}	G_{22}	G_{23}	Indel
A	0	99.9	0	0	0	0	0	0	0	0	99.9	0	0	0	99.9	0	0	0	0	0	0	99.7	0	99.7	0	0	0	0	0	0	0	0	0	0
C	0.1	0	0	99.9	0.1	99.9	0	0	0	99.9	0	0	99.9	100	0	100	100	100	0.1	0	0	0	0	0	0	99.9	99.9	0.1	100	99.9	0	0	0	
G	0	0.1	100	0	0.1	0	99.9	99.9	99.9	0	0.1	99.9	0	0	0.1	0	0	0	0	100	100	0.2	100	0.2	100	0	0	0.2	0	0	99.9	100	99.9	
T	99.9	0	0	0	99.8	0.1	0	0	0	0	0	0	0.1	0	0	0	0	0	99.9	0	0	0	0	0	0	0	0	99.7	0	0.1	0	0	0	

264. A B2M Ex.1 SD OT9 + BE4 mRNA

	A_{-10}	C_{-9}	C_{-8}	A_{-7}	A_{-6}	A_{-5}	A_{-4}	G_{-3}	C_{-2}	A_{-1}	A_{1}	C_{2}	T_{3}	C_{4}	C_{5}	C_{6}	G_{7}	C_{8}	T_{9}	G_{10}	G_{11}	A_{12}	A_{13}	A_{14}	G_{15}	C_{16}	C_{17}	T_{18}	G_{19}	C_{20}	A_{21}	G_{22}	G_{23}	Indel
A	99.7	0.1	0.2	99.6	99.6	99.7	99.7	0.1	0.2	100	99.6	0	0.1	0	0	0	0.1	0.1	0	0	0	99.9	99.9	99.7	0.3	0.4	1	0.1	0.1	0	99.9	0	0	0
C	0.3	99.9	99.8	0.4	0.3	0.2	0.2	0	99.8	0	0.2	99.9	0.5	100	99.9	99.9	0	99.9	0.2	0	0	0	0	0	0	99.4	98.9	0.2	0	99.9	0	0	0	
G	0.1	0	0	0.1	0	0.1	0.1	99.9	0	0	0.2	0	0	0	0	0	99.8	0	0	100	99.9	0.1	0.1	0.3	99.6	0.2	0	0.1	99.9	0	0.1	100	100	
T	0	0	0	0	0	0	0	0	0	0	0	0	99.4	0	0	0.1	0	0	99.8	0	0	0	0	0	0	0	0	99.6	0	0.1	0	0	0	

265. A B2M Ex.1 SD OT10 + BE4 mRNA

	T_{-10}	C_{-9}	T_{-8}	C_{-7}	C_{-6}	T_{-5}	T_{-4}	T_{-3}	G_{-2}	A_{-1}	C_{1}	C_{2}	T_{3}	C_{4}	C_{5}	C_{6}	G_{7}	C_{8}	T_{9}	G_{10}	T_{11}	G_{12}	T_{13}	A_{14}	G_{15}	C_{16}	C_{17}	T_{18}	C_{19}	C_{20}	T_{21}	G_{22}	G_{23}	Indel
A	0	0	0	0	0	0	0	0	0	99.7	0	0	0	0	0	0	0.1	0	0	0.1	0	0	0	99.8	0	0	0	0	0	0	0	0	0	0
C	0.2	100	0.2	100	100	0.2	0.1	0.1	0	0.1	100	100	0.2	100	100	99.9	0	100	0.2	0	0.2	0	0.1	0	0	99.9	99.9	0.2	100	100	0.1	0	0	
G	0	0	0	0	0	0	0	0	99.9	0.2	0	0	0	0	0	0	99.9	0	0	99.9	0	99.9	0	0.1	99.9	0	0	0	0	0	0	100	99.8	
T	99.8	0	99.8	0	0	99.8	99.9	99.9	0	0	0	0	99.8	0	0	0.1	0	0	99.8	0	99.8	0	99.9	0	0	0.1	0	99.7	0	0	99.9	0	0.1	

266. A TRAC Ex.3 SA OnT + Pulse

	A_{-10}	A_{-9}	A_{-8}	G_{-7}	T_{-6}	T_{-5}	T_{-4}	A_{-3}	G_{-2}	G_{-1}	T_{1}	T_{2}	C_{3}	G_{4}	T_{5}	A_{6}	T_{7}	C_{8}	T_{9}	G_{10}	T_{11}	A_{12}	A_{13}	A_{14}	A_{15}	C_{16}	C_{17}	A_{18}	A_{19}	G_{20}	A_{21}	G_{22}	G_{23}	Indel
A	99.9	99.9	99.8	0	0	0	0	99.9	0	0	0	0	0	0	0	99.9	0	0	0	0	0	100	99.9	99.9	99.8	0	0	99.9	99.8	0	99.6	0	0.1	0
C	0	0.1	0	0	0	0.1	0	0	0	0	0.1	0	99.9	0	0.1	0	0.1	100	0.1	0	0	0	0	0	0	100	99.9	0	0.1	0	0.1	0	0	
G	0	0	0.1	100	0.1	0.1	0	0	99.9	100	0	0	0	99.9	0	0	0.1	0	0	99.9	0	0	0.1	0	0.2	0	0	0	0.2	99.9	0.3	99.9	99.9	
T	0	0	0	0	99.8	99.9	100	0	0	0	99.8	99.9	0.1	0	99.9	0	99.8	0	99.9	0	99.9	0	0	0	0	0	0	0	0	0	0	0	0	

267. A TRAC Ex.3 SA OT1 + Pulse

	T_{-10}	T_{-9}	T_{-8}	T_{-7}	C_{-6}	C_{-5}	T_{-4}	T_{-3}	A_{-2}	C_{-1}	T_{1}	T_{2}	G_{3}	G_{4}	G_{5}	A_{6}	T_{7}	C_{8}	T_{9}	T_{10}	T_{11}	A_{12}	A_{13}	A_{14}	A_{15}	C_{16}	C_{17}	A_{18}	A_{19}	G_{20}	G_{21}	G_{22}	G_{23}	Indel
A	0	0	0	0	0.1	0	0	0	100	0	0	0	0	0	0	99.8	0	0.1	0	0	0	99.9	99.9	99.8	99.7	0	0	99.9	99.8	0	0	0	0	0
C	0.1	0.2	0.1	0	99.9	99.9	0.1	0	0	100	0.1	0	0	0	0	0	0.1	99.9	0.1	0	0	0	0	0.1	0	100	99.9	0	0	0	0	0	0	
G	0	0	0	0	0	0	0	0	0	0	0	0	99.8	99.9	99.9	0.1	0.1	0	0.1	0	0	0	0.1	0.1	0.2	0	0	0	0.1	99.9	99.9	99.9	100	
T	99.8	99.8	99.9	100	0	0	99.8	100	0	0	99.9	99.9	0.1	0.1	0	0	99.8	0	99.8	100	99.9	0	0	0	0	0	0	0	0	0	0	0.1	0	

268. A TRAC Ex.3 SA OT2 + Pulse

	A$_{-10}$	G$_{-9}$	T$_{-8}$	A$_{-7}$	G$_{-6}$	G$_{-5}$	T$_{-4}$	A$_{-3}$	T$_{-2}$	C$_{-1}$	T$_{1}$	G$_{2}$	A$_{3}$	G$_{4}$	C$_{5}$	A$_{6}$	T$_{7}$	C$_{8}$	T$_{9}$	G$_{10}$	T$_{11}$	A$_{12}$	A$_{13}$	A$_{14}$	A$_{15}$	C$_{16}$	C$_{17}$	A$_{18}$	A$_{19}$	G$_{20}$	T$_{21}$	G$_{22}$	G$_{23}$	Indel
A	99.8	0	0	99.9	0	0	0	100	0	0	0	0	99.8	0	0.1	99.9	0	0	0	0.1	0	100	99.9	99.9	99.8	0	0.1	99.9	99.9	0	0	0	0	0
C	0	0	0	0	0	0	0.1	0	0	99.9	0.1	0	0	0	99.9	0	0.1	100	0	0	0.1	0	0	0	0.1	99.9	99.9	0	0	0	0.1	0	0	
G	0.2	99.9	0	0.1	99.8	99.9	0	0	0	0	0	100	0.2	99.9	0	0	0	0	0	99.9	0	0	0.1	0.1	0.2	0	0	0.1	0.1	99.9	0.1	99.9	99.9	
T	0	0.1	100	0	0.1	0.1	99.9	0	99.9	0	99.9	0	0	0	0	0	99.9	0	99.9	0	99.8	0	0	0	0	0	0	0	0	0	99.8	0	0.1	

269. A TRAC Ex.3 SA OT3 + Pulse

	G$_{-10}$	G$_{-9}$	C$_{-8}$	T$_{-7}$	T$_{-6}$	C$_{-5}$	A$_{-4}$	G$_{-3}$	C$_{-2}$	C$_{-1}$	T$_{1}$	T$_{2}$	C$_{3}$	T$_{4}$	A$_{5}$	A$_{6}$	T$_{7}$	C$_{8}$	T$_{9}$	C$_{10}$	T$_{11}$	A$_{12}$	A$_{13}$	A$_{14}$	A$_{15}$	C$_{16}$	C$_{17}$	A$_{18}$	A$_{19}$	G$_{20}$	T$_{21}$	G$_{22}$	G$_{23}$	Indel
A	0	0	0	0	0	0	99.9	0	0.1	0	0	0	0	0	100	100	0	0	0.1	0	0	100	99.9	99.9	99.8	0	0	99.9	99.9	0	0	0	0.1	0
C	0	0	100	0.1	0	99.9	0	0	99.9	99.9	0.2	0	100	0	0	0	0.1	100	0.1	99.9	0	0	0	0.1	0	100	100	0	0	0	0.1	0	0	
G	99.8	99.9	0	0	0	0	0.1	99.8	0	0	0	0	0	0	0	0	0	0	0.2	0	0	0	0	0	0.2	0	0	0.1	0.1	99.9	0	99.8	99.8	
T	0.2	0.1	0	99.8	99.9	0	0	0.2	0	0	99.8	100	0	100	0	0	99.9	0	99.6	0	99.9	0	0	0	0	0	0	0	0	0	99.9	0.2	0.1	

270. A TRAC Ex.3 SA OT4 + Pulse

	A$_{-10}$	C$_{-9}$	A$_{-8}$	G$_{-7}$	T$_{-6}$	G$_{-5}$	T$_{-4}$	T$_{-3}$	T$_{-2}$	A$_{-1}$	G$_{1}$	T$_{2}$	G$_{3}$	G$_{4}$	T$_{5}$	A$_{6}$	T$_{7}$	C$_{8}$	T$_{9}$	G$_{10}$	C$_{11}$	A$_{12}$	A$_{13}$	A$_{14}$	A$_{15}$	C$_{16}$	C$_{17}$	A$_{18}$	A$_{19}$	G$_{20}$	G$_{21}$	G$_{22}$	G$_{23}$	Indel
A	99.9	0	99.9	0	0	0	0	0	0	100	0	0	0	0	0	99.9	0	0	0	0.1	0	100	99.8	100	99.8	0	0	100	99.8	0	0	0.1	0	0
C	0	99.9	0	0	0.1	0	0.1	0	0	0	0	0.1	0	0	0	0	0.1	99.9	0.1	0	99.9	0	0	0	0	99.9	100	0	0	0	0	0	0	
G	0.1	0	0	100	0.1	100	0.1	0	0	0	100	0	99.9	99.9	0	0	0.1	0	0	99.9	0	0	0.1	0	0.1	0	0	0	0.2	99.9	99.9	99.8	99.9	
T	0	0	0	0	99.8	0	99.8	99.9	100	0	0	99.8	0	0	100	0	99.8	0	99.9	0	0	0	0	0	0	0	0	0	0	0	0.1	0.1	0	

271. A TRAC Ex.3 SA OT5 + Pulse

	G$_{-10}$	G$_{-9}$	C$_{-8}$	C$_{-7}$	T$_{-6}$	C$_{-5}$	A$_{-4}$	G$_{-3}$	T$_{-2}$	T$_{-1}$	T$_{1}$	C$_{2}$	C$_{3}$	T$_{4}$	C$_{5}$	A$_{6}$	T$_{7}$	G$_{8}$	T$_{9}$	G$_{10}$	T$_{11}$	A$_{12}$	A$_{13}$	A$_{14}$	A$_{15}$	C$_{16}$	C$_{17}$	A$_{18}$	A$_{19}$	G$_{20}$	A$_{21}$	G$_{22}$	G$_{23}$	Indel
A	0.1	0	0	0	0	0	99.9	0	0	0	0	0	0	0	0	99.8	0	0	0	0	0	99.9	99.8	99.8	99.7	0	0	99.9	99.8	0	99.7	0	0	0
C	0	0	99.9	99.9	0.2	100	0	0	0.1	0.2	0.1	100	99.9	0.2	100	0	0.1	0	0.1	0	0.1	0	0	0.1	0	99.9	99.9	0	0	0	0	0	0	
G	99.9	99.9	0	0	0	0	0.1	99.9	0	0	0	0	0	0	0	0.1	0	99.9	0	99.9	0	0	0.2	0.1	0.2	0	0	0.1	0.2	100	0.2	99.9	99.9	
T	0.1	0.1	0	0	99.8	0	0	0	99.8	99.8	99.9	0	0	99.8	0	0	99.9	0	99.8	0	99.9	0	0	0	0	0	0	0	0	0	0	0	0.1	

272. A TRAC Ex.3 SA OT6 + Pulse

	G$_{-10}$	A$_{-9}$	T$_{-8}$	A$_{-7}$	A$_{-6}$	G$_{-5}$	A$_{-4}$	A$_{-3}$	G$_{-2}$	T$_{-1}$	T$_{1}$	C$_{2}$	T$_{3}$	G$_{4}$	C$_{5}$	A$_{6}$	T$_{7}$	C$_{8}$	T$_{9}$	T$_{10}$	T$_{11}$	A$_{12}$	A$_{13}$	A$_{14}$	A$_{15}$	C$_{16}$	C$_{17}$	A$_{18}$	A$_{19}$	G$_{20}$	T$_{21}$	G$_{22}$	G$_{23}$	Indel
A	0	99.9	0	100	99.8	0	100	99.9	0	0	0	0.2	0	0.1	0.1	100	0	0.1	0	0	0	100	100	99.9	99.8	0.1	0.3	100	99.8	0	0	0	0	0
C	0	0	0	0	0	0	0	0	0	0.1	0.1	99.8	0.1	0	99.8	0	0.1	99.9	0.1	0	0	0	0	0	0	99.9	99.7	0	0	0	0.1	0	0	
G	99.9	0.1	0	0	0.1	100	0	0	99.9	0	0.1	0	0.1	99.8	0	0	0	0	0.1	0	0	0	0	0	0.1	0	0	0	0.1	100	0.1	99.7	99.9	
T	0	0	100	0	0	0	0	0	0	99.8	99.8	0	99.8	0.1	0	0	99.9	0	99.8	99.9	99.9	0	0	0	0	0	0	0	0	0	99.7	0.3	0	

273. A TRAC Ex.3 SA OT7 + Pulse

	C$_{-10}$	A$_{-9}$	T$_{-8}$	A$_{-7}$	T$_{-6}$	G$_{-5}$	T$_{-4}$	C$_{-3}$	T$_{-2}$	T$_{-1}$	T$_{1}$	T$_{2}$	T$_{3}$	G$_{4}$	T$_{5}$	A$_{6}$	T$_{7}$	C$_{8}$	T$_{9}$	T$_{10}$	T$_{11}$	A$_{12}$	A$_{13}$	A$_{14}$	A$_{15}$	C$_{16}$	C$_{17}$	A$_{18}$	T$_{19}$	G$_{20}$	T$_{21}$	G$_{22}$	G$_{23}$	Indel
A	0.4	99.9	0	99.9	0	0	0	0.6	0	0	0	0	0	0	0	99.9	0.1	0.1	0	0	0	100	99.9	99.9	99.7	0.2	0.3	99.9	0	0	0	0	0	0
C	99.5	0	0.1	0	0.1	0	0.2	99.3	0.2	0	0	0	0	0	0.1	0	0.1	99.8	0.2	0	0	0	0	0	0	99.8	99.7	0	0.1	0	0.1	0	0	
G	0	0	0	0	0	99.8	0	0	0.2	0.1	0.1	0	0	99.8	0	0	0.1	0	0.1	0	0	0	0.1	0	0.2	0	0	0.1	0	99.8	0	99.6	99.8	
T	0.1	0	99.9	0	99.9	0.1	99.7	0.1	99.6	99.8	99.9	99.9	99.9	0.1	99.9	0	99.7	0	99.7	100	99.9	0	0	0	0	0	0	0	99.8	0.1	99.8	0.3	0.1	

274. A TRAC Ex.3 SA OT8 + Pulse

	T$_{-10}$	G$_{-9}$	G$_{-8}$	A$_{-7}$	G$_{-6}$	A$_{-5}$	T$_{-4}$	G$_{-3}$	C$_{-2}$	T$_{-1}$	T$_{1}$	T$_{2}$	C$_{3}$	T$_{4}$	T$_{5}$	A$_{6}$	T$_{7}$	G$_{8}$	T$_{9}$	G$_{10}$	T$_{11}$	G$_{12}$	A$_{13}$	A$_{14}$	A$_{15}$	C$_{16}$	C$_{17}$	A$_{18}$	A$_{19}$	G$_{20}$	G$_{21}$	G$_{22}$	G$_{23}$	Indel
A	0	0	0	99.7	0	99.8	0	0	0	0	0	0	0	0	0	99.9	0	0	0	0	0	0	99.9	99.9	99.8	0	0.1	99.9	99.8	0	0.1	0	0.1	0
C	0	0	0	0	0	0	0.1	0	99.9	0.1	0	0.1	100	0.1	0	0	0	0	0.2	0	0.1	0	0	0	0	99.9	99.9	0	0	0	0	0	0	
G	0	99.9	99.9	0.2	99.9	0.1	0	99.9	0	0.1	0.1	0.1	0	0	0	0.1	0	99.9	0	99.9	0	99.9	0.1	0	0.2	0	0	0	0.1	100	99.8	99.8	99.8	
T	99.9	0.1	0	0.1	0	0	99.9	0	0	99.7	99.8	99.8	0	99.8	100	0	99.9	0	99.8	0	99.9	0.1	0	0	0	0	0	0	0	0	0.1	0.1	0.1	

275. A TRAC Ex.3 SA OT9 + Pulse

	G$_{-10}$	G$_{-9}$	A$_{-8}$	C$_{-7}$	T$_{-6}$	G$_{-5}$	G$_{-4}$	C$_{-3}$	A$_{-2}$	T$_{-1}$	C$_{1}$	T$_{2}$	C$_{3}$	T$_{4}$	C$_{5}$	T$_{6}$	T$_{7}$	C$_{8}$	T$_{9}$	G$_{10}$	T$_{11}$	A$_{12}$	A$_{13}$	A$_{14}$	A$_{15}$	C$_{16}$	C$_{17}$	A$_{18}$	A$_{19}$	G$_{20}$	A$_{21}$	G$_{22}$	G$_{23}$	Indel
A	0	0	99.8	0	0	0	0.1	0	99.9	0	0	0	0	0	0	0	0	0	0	0	0	100	99.9	99.9	99.8	0	0	99.9	99.8	0	99.8	0	0	0
C	0	0	0	99.9	0.1	0	0	99.9	0	0.2	100	0.2	100	0.2	100	0.2	0	100	0.1	0	0	0	0	0	0	100	99.9	0	0	0	0	0	0	
G	99.8	99.9	0.1	0	0	99.8	99.9	0	0	0	0	0	0	0.1	0	0	0	0	0	99.9	0	0	0.1	0	0.2	0	0	0	0.1	100	0.2	99.9	99.9	
T	0.1	0.1	0	0	99.8	0.1	0	0	0	99.8	0	99.8	0	99.7	0	99.8	99.9	0	99.9	0	99.9	0	0	0	0	0	0	0	0	0	0	0.1	0	

276. A TRAC Ex.3 SA OT10 + Pulse

	T_{-10}	G_{-9}	G_{-8}	C_{-7}	T_{-6}	C_{-5}	T_{-4}	T_{-3}	T_{-2}	A_{-1}	T_{1}	G_{2}	G_{3}	G_{4}	T_{5}	T_{6}	T_{7}	C_{8}	T_{9}	T_{10}	T_{11}	A_{12}	A_{13}	A_{14}	A_{15}	C_{16}	C_{17}	A_{18}	A_{19}	G_{20}	G_{21}	G_{22}	G_{23}	Indel
A	0	0	0	0	0	0	0	0	0	100	0	0	0	0	0	0	0	0	0	0	0	100	99.9	99.9	99.8	0.1	0	100	99.9	0	0	0	0	0
C	0	0	0	99.9	0.1	99.9	0.1	0	0	0	0	0	0	0	0.1	0	0	99.9	0.1	0	0.1	0	0	0	0	99.9	99.9	0	0	0	0	0	0	
G	0	99.9	99.9	0	0.1	0	0.1	0	0	0	0	99.9	99.9	99.9	0	0	0	0	0	0	0	0	0	0	0.1	0	0	0	0.1	100	100	99.9	100	
T	100	0.1	0	0	99.8	0	99.8	99.9	100	0	99.9	0.1	0.1	0	99.9	100	100	0	99.8	100	99.9	0	0	0	0	0	0	0	0	0	0	0.1	0	

277. A TRAC Ex.3 SA OnT + Cas9 mRNA

	A_{-10}	A_{-9}	A_{-8}	G_{-7}	T_{-6}	T_{-5}	T_{-4}	A_{-3}	G_{-2}	G_{-1}	T_{1}	T_{2}	C_{3}	G_{4}	T_{5}	A_{6}	T_{7}	C_{8}	T_{9}	G_{10}	T_{11}	A_{12}	A_{13}	A_{14}	A_{15}	C_{16}	C_{17}	A_{18}	A_{19}	G_{20}	A_{21}	G_{22}	G_{23}	Indel
A	99.9	99.9	99.7	0	0	0	0	99.7	0	0.1	0	0.2	0.1	0.1	0.3	99.8	0.1	0.1	0.1	0.1	0	99.7	99.7	99.7	99.6	0.9	1	99.5	99.2	0.2	99.3	0.1	0.2	85.5
C	0	0	0.1	0	0	0.1	0	0	0	0	0.1	0	99.8	0	0	0	0.2	99.7	0.1	0	0.1	0	0	0.1	0.2	98	98.3	0.2	0.2	0.1	0.1	0.1	0.1	
G	0	0	0.2	99.9	0.1	0.1	0	0.2	99.9	99.8	0	0	0	99.8	0	0.1	0.1	0.1	0.1	99.8	0.1	0.1	0.1	0.2	0.1	0.6	0.4	0.1	0.4	99.5	0.3	99.6	99.6	
T	0	0	0	0	99.8	99.8	99.9	0	0.1	0.1	99.8	99.8	0.1	0.2	99.7	0.1	99.6	0.1	99.7	0.1	99.8	0.1	0.2	0.1	0.1	0.5	0.3	0.2	0.3	0.2	0.4	0.1	0.1	

278. A TRAC Ex.3 SA OT1 + Cas9 mRNA

	T_{-10}	T_{-9}	T_{-8}	T_{-7}	C_{-6}	C_{-5}	T_{-4}	T_{-3}	A_{-2}	C_{-1}	T_{1}	T_{2}	G_{3}	G_{4}	G_{5}	A_{6}	T_{7}	C_{8}	T_{9}	T_{10}	T_{11}	A_{12}	A_{13}	A_{14}	A_{15}	C_{16}	C_{17}	A_{18}	A_{19}	G_{20}	G_{21}	G_{22}	G_{23}	Indel
A	0	0	0	0	0	0	0	0	100	0	0	0	0	0	0	99.9	0	0	0	0	0	100	99.9	99.8	99.8	0	0	100	99.9	0	0	0	0.1	0
C	0.1	0.2	0	0	100	100	0.1	0.1	0	100	0.1	0	0	0	0	0	0	99.9	0.1	0	0	0	0	0	0	100	100	0	0	0	0	0	0	
G	0	0	0	0	0	0	0	0	0	0	0	0	99.9	99.9	100	0.1	0	0	0	0	0	0	0.1	0.2	0.2	0	0	0	0.1	100	99.9	99.9	99.9	
T	99.9	99.7	99.9	100	0	0	99.8	99.9	0	0	99.9	100	0.1	0	0	0	99.9	0	99.9	100	100	0	0	0	0	0	0	0	0	0	0.1	0.1	0	

279. A TRAC Ex.3 SA OT2 + Cas9 mRNA

	A_{-10}	G_{-9}	T_{-8}	A_{-7}	G_{-6}	G_{-5}	T_{-4}	A_{-3}	T_{-2}	C_{-1}	T_{1}	G_{2}	A_{3}	G_{4}	C_{5}	A_{6}	T_{7}	C_{8}	T_{9}	G_{10}	T_{11}	A_{12}	A_{13}	A_{14}	A_{15}	C_{16}	C_{17}	A_{18}	A_{19}	G_{20}	T_{21}	G_{22}	G_{23}	Indel
A	99.5	0.1	0	99.9	0.1	0.1	0.1	99.9	0	0	0	0.1	99.7	0	0.1	99.9	0	0.1	0	0.1	0	100	100	99.9	99.8	0.1	0.2	99.9	99.8	0	0	0	0	0
C	0	0	0	0	0.1	0	0.1	0	0.2	100	0.3	0	0.1	0	99.8	0	0.1	99.9	0	0.1	0	0	0	0.1	0.1	99.9	99.8	0	0.1	0	0	0	0	
G	0.4	99.7	0	0	99.8	99.8	0.1	0.1	0.1	0	0	99.9	0.1	100	0	0.1	0	0	0.1	99.7	0.1	0	0	0	0.1	0	0	0.1	0.1	100	0.1	100	100	
T	0.1	0.2	100	0.1	0	0.1	99.7	0	99.7	0	99.7	0	0.1	0	0.1	0	99.9	0	99.9	0.1	99.9	0	0	0	0	0	0	0	0	0	99.9	0	0	

280. A TRAC Ex.3 SA OT3 + Cas9 mRNA

	G_{-10}	G_{-9}	C_{-8}	T_{-7}	T_{-6}	C_{-5}	A_{-4}	G_{-3}	C_{-2}	C_{-1}	T_{1}	T_{2}	C_{3}	T_{4}	A_{5}	A_{6}	T_{7}	C_{8}	T_{9}	C_{10}	T_{11}	A_{12}	A_{13}	A_{14}	A_{15}	C_{16}	C_{17}	A_{18}	A_{19}	G_{20}	T_{21}	G_{22}	G_{23}	Indel
A	0	0	0	0	0	0.1	99.8	0	0	0.1	0	0	0	0	100	100	0	0.1	0	0.1	0	100	100	100	99.9	0	0	100	99.9	0	0	0	0	0
C	0	0	99.9	0.2	0.1	99.9	0	0	99.9	99.9	0.3	0	99.9	0	0	0	0.1	99.8	0.2	99.9	0	0	0	0	0	99.9	99.9	0	0	0	0.1	0	0	
G	99.8	99.9	0	0.1	0	0	0.1	99.7	0	0	0	0	0	0	0	0	0	0	0.2	0	0	0	0	0	0.1	0	0	0	0.1	100	0	99.9	99.8	
T	0.2	0	0	99.7	99.8	0	0	0.3	0.1	0	99.7	99.9	0.1	100	0	0	99.8	0	99.7	0	100	0	0	0	0	0.1	0	0	0	0	99.8	0.1	0.1	

281. A TRAC Ex.3 SA OT4 + Cas9 mRNA

	A_{-10}	C_{-9}	A_{-8}	G_{-7}	T_{-6}	G_{-5}	T_{-4}	T_{-3}	T_{-2}	A_{-1}	G_{1}	T_{2}	G_{3}	G_{4}	T_{5}	A_{6}	T_{7}	C_{8}	T_{9}	G_{10}	C_{11}	A_{12}	A_{13}	A_{14}	A_{15}	C_{16}	C_{17}	A_{18}	A_{19}	G_{20}	G_{21}	G_{22}	G_{23}	Indel
A	99.7	0	99.9	0	0	0	0	0	0	99.9	0	0	0	0	0	99.9	0	0	0	0.1	0	99.9	99.8	99.9	99.8	0	0	99.9	99.8	0	0.1	0.1	0.1	0.2
C	0	100	0	0	0.2	0	0.2	0.1	0	0	0	0.1	0	0	0.1	0	0.1	100	0.1	0	99.9	0	0	0	0	99.9	100	0	0	0	0	0	0	
G	0.2	0	0.1	100	0	100	0	0	0	0	100	0	99.9	100	0	0.1	0	0	0	99.9	0	0.1	0.2	0.1	0.2	0	0	0.1	0.1	100	99.9	99.9	99.9	
T	0	0	0	0	99.8	0	99.8	99.9	99.9	0	0	99.8	0	0	99.9	0	99.9	0	99.8	0	0.1	0	0	0	0	0	0	0	0	0	0	0	0	

282. A TRAC Ex.3 SA OT5 + Cas9 mRNA

	G_{-10}	G_{-9}	C_{-8}	C_{-7}	T_{-6}	C_{-5}	A_{-4}	G_{-3}	T_{-2}	T_{-1}	T_{1}	C_{2}	C_{3}	T_{4}	C_{5}	A_{6}	T_{7}	G_{8}	T_{9}	G_{10}	T_{11}	A_{12}	A_{13}	A_{14}	A_{15}	C_{16}	C_{17}	A_{18}	A_{19}	G_{20}	A_{21}	G_{22}	G_{23}	Indel
A	0.1	0.1	0.1	0.1	0	0.1	99.9	0	0	0	0	0	0.1	0	0	99.8	0	0	0	0	0	99.9	99.8	99.8	99.7	0.1	0	99.9	99.8	0	99.7	0	0	0
C	0	0	99.8	99.8	0.2	99.9	0	0	0.2	0.2	0.1	99.9	99.9	0.2	99.9	0	0.1	0	0.2	0	0.1	0	0	0.1	0.1	99.9	99.9	0	0	0	0.1	0	0	
G	99.7	99.8	0	0	0.1	0	0.1	99.9	0	0	0	0	0	0.1	0	0.1	0.1	99.9	0	99.9	0	0.1	0.1	0.1	0.2	0	0	0.1	0.2	99.9	0.2	99.9	99.8	
T	0.2	0.2	0.1	0	99.7	0	0	0.1	99.8	99.8	99.9	0	0	99.7	0	0	99.8	0.1	99.8	0	99.9	0	0	0	0	0	0	0	0	0.1	0	0.1	0.2	

283. A TRAC Ex.3 SA OT6 + Cas9 mRNA

	G_{-10}	A_{-9}	T_{-8}	A_{-7}	A_{-6}	G_{-5}	A_{-4}	A_{-3}	G_{-2}	T_{-1}	T_{1}	C_{2}	T_{3}	G_{4}	C_{5}	A_{6}	T_{7}	C_{8}	T_{9}	T_{10}	T_{11}	A_{12}	A_{13}	A_{14}	A_{15}	C_{16}	C_{17}	A_{18}	A_{19}	G_{20}	T_{21}	G_{22}	G_{23}	Indel
A	0.1	100	0	100	99.9	0	100	99.9	0	0	0	0.2	0.1	0	0.1	100	0	0.1	0	0	0	100	100	99.9	99.9	0.2	0.3	100	99.8	0	0	0.1	0.1	0
C	0	0	0	0	0	0	0	0	0	0.1	0.1	99.8	0.1	0	99.9	0	0.1	99.9	0.2	0.1	0	0	0	0.1	0	99.8	99.7	0	0.1	0	0.1	0	0	
G	99.9	0	0	0	0.1	99.9	0	0.1	99.8	0	0.2	0	0	99.9	0	0	0	0	0.1	0.1	0	0	0	0	0	0	0	0	0.1	99.9	0	99.7	99.9	
T	0.1	0	100	0	0	0.1	0	0	0.2	99.9	99.8	0.1	99.8	0.1	0	0	99.8	0	99.7	99.8	99.9	0	0	0	0	0.1	0	0	0	0	99.9	0.2	0	

284. A TRAC Ex.3 SA OT7 + Cas9 mRNA

	C_{-10}	A_{-9}	T_{-8}	A_{-7}	T_{-6}	G_{-5}	T_{-4}	C_{-3}	T_{-2}	T_{-1}	T_{1}	T_{2}	T_{3}	G_{4}	T_{5}	A_{6}	T_{7}	C_{8}	T_{9}	T_{10}	T_{11}	A_{12}	A_{13}	A_{14}	A_{15}	C_{16}	C_{17}	A_{18}	T_{19}	G_{20}	T_{21}	G_{22}	G_{23}	Indel
A	0.5	100	0	100	0	0.1	0	0.5	0	0	0	0	0	0.1	0	100	0.1	0.2	0.1	0	0	100	99.9	99.9	99.7	0.2	0.4	99.9	0.1	0.1	0	0.1	0	0
C	99.4	0	0	0	0.1	0	0.1	99.4	0.1	0.1	0	0	0	0	0.1	0	0.1	99.8	0.1	0	0	0	0	0	0	99.8	99.6	0	0.1	0	0.1	0	0	
G	0	0	0	0	0	99.8	0	0	0.2	0.1	0.1	0	0	99.7	0	0	0.2	0	0.1	0	0	0	0.1	0.1	0.2	0	0	0.1	0	99.8	0.1	99.5	99.8	
T	0.1	0	99.9	0	99.9	0.1	99.8	0.1	99.7	99.8	99.8	99.9	99.9	0.2	99.9	0	99.7	0	99.7	99.9	100	0	0	0	0	0	0	0	99.8	0.1	99.8	0.3	0.1	

285. A TRAC Ex.3 SA OT8 + Cas9 mRNA

	T_{-10}	G_{-9}	G_{-8}	A_{-7}	G_{-6}	A_{-5}	T_{-4}	G_{-3}	C_{-2}	T_{-1}	T_{1}	T_{2}	C_{3}	T_{4}	T_{5}	A_{6}	T_{7}	G_{8}	T_{9}	G_{10}	T_{11}	G_{12}	A_{13}	A_{14}	A_{15}	C_{16}	C_{17}	A_{18}	A_{19}	G_{20}	G_{21}	G_{22}	G_{23}	Indel
A	0	0	0	99.8	0	99.8	0	0	0	0	0	0	0	0	0	99.9	0	0	0	0	0	0	99.9	100	99.7	0	0	99.9	99.8	0	0	0	0.1	0
C	0	0	0	0	0	0	0.1	0	100	0.2	0	0.1	100	0.1	0	0	0.1	0	0.2	0	0.1	0	0	0	0	99.9	99.9	0	0	0	0	0	0	
G	0	99.9	100	0.1	100	0.1	0	100	0	0	0	0	0	0	0	0.1	0	100	0	100	0	100	0.1	0	0.2	0	0	0.1	0.2	100	99.9	99.9	99.9	
T	99.9	0	0	0	0	0	99.9	0	0	99.8	99.9	99.8	0	99.8	99.9	0	99.9	0	99.8	0	99.9	0	0	0	0	0	0	0	0	0	0	0	0	

286. A TRAC Ex.3 SA OT9 + Cas9 mRNA

	G_{-10}	G_{-9}	A_{-8}	C_{-7}	T_{-6}	G_{-5}	G_{-4}	C_{-3}	A_{-2}	T_{-1}	C_{1}	T_{2}	C_{3}	T_{4}	C_{5}	T_{6}	T_{7}	C_{8}	T_{9}	G_{10}	T_{11}	A_{12}	A_{13}	A_{14}	A_{15}	C_{16}	C_{17}	A_{18}	A_{19}	G_{20}	A_{21}	G_{22}	G_{23}	Indel
A	0	0	99.8	0	0	0	0	0	99.9	0	0	0	0	0	0	0	0	0	0	0	0	100	99.9	99.9	99.8	0	0	99.9	99.9	0	99.8	0	0	0
C	0	0	0	100	0.1	0	0	99.9	0	0.2	100	0.2	100	0.2	100	0.1	0	100	0.1	0	0	0	0	0	0	100	99.9	0	0	0	0	0	0	
G	99.8	99.9	0.1	0	0	99.9	99.9	0	0	0	0	0	0	0	0	0	0	0	0	100	0	0	0	0	0.1	0	0	0	0.1	100	0.2	99.9	99.9	
T	0.1	0.1	0	0	99.8	0.1	0	0	0	99.8	0	99.7	0	99.8	0	99.8	99.9	0	99.9	0	99.9	0	0	0	0	0	0	0	0	0	0	0	0	

287. A TRAC Ex.3 SA OT10 + Cas9 mRNA

	T_{-10}	G_{-9}	G_{-8}	C_{-7}	T_{-6}	C_{-5}	T_{-4}	T_{-3}	T_{-2}	A_{-1}	T_{1}	G_{2}	G_{3}	G_{4}	T_{5}	T_{6}	T_{7}	C_{8}	T_{9}	T_{10}	T_{11}	A_{12}	A_{13}	A_{14}	A_{15}	C_{16}	C_{17}	A_{18}	A_{19}	G_{20}	G_{21}	G_{22}	G_{23}	Indel
A	0	0	0.1	0.1	0	0	0	0	0	100	0	0	0	0	0	0	0	0	0	0	0	100	99.9	99.9	99.8	0.1	0	100	99.9	0	0	0.1	0	0
C	0	0	0	99.9	0.2	99.9	0.1	0	0	0	0	0	0	0	0.1	0	0	100	0.1	0	0.2	0	0	0	0	99.9	99.9	0	0	0	0	0	0	
G	0	99.9	99.9	0	0.1	0	0.2	0	0	0	0	99.9	99.9	99.9	0	0	0	0	0	0	0	0	0.1	0	0.1	0	0	0	0.1	100	99.9	99.9	100	
T	100	0.1	0	0	99.7	0	99.7	99.9	100	0	99.9	0.1	0.1	0	99.9	100	99.9	0	99.8	99.9	99.8	0	0	0	0	0	0	0	0	0	0	0.1	0	

288. A TRAC Ex.3 SA OnT + BE4 mRNA

	A_{-10}	A_{-9}	A_{-8}	G_{-7}	T_{-6}	T_{-5}	T_{-4}	A_{-3}	G_{-2}	G_{-1}	T_{1}	T_{2}	C_{3}	G_{4}	T_{5}	A_{6}	T_{7}	C_{8}	T_{9}	G_{10}	T_{11}	A_{12}	A_{13}	A_{14}	A_{15}	C_{16}	C_{17}	A_{18}	A_{19}	G_{20}	A_{21}	G_{22}	G_{23}	Indel
A	99.9	99.9	99.8	0	0	0	0	99.9	0	0	0	0	0.6	0	0	100	0	5.8	0	0	0	100	99.8	99.9	99.7	0	0.1	99.9	99.8	0	99.7	0	0.1	2.8
C	0	0	0	0	0.1	0.1	0.1	0	0	0	0.1	0.1	89.3	0	0.1	0	0.1	32.5	0.1	0	0.1	0	0	0	0	99.8	99.8	0	0	0	0	0	0	
G	0	0.1	0.2	99.9	0	0	0	0.1	100	99.9	0	0	0.1	99.9	0	0	0	16.9	0	99.9	0	0	0.2	0.1	0.2	0	0	0	0.2	99.9	0.3	100	99.9	
T	0	0	0	0	99.9	99.8	99.9	0	0	0	99.9	99.8	10	0	99.9	0	99.8	44.7	99.9	0	99.9	0	0	0	0	0.2	0.1	0	0	0	0	0	0	

289. A TRAC Ex.3 SA OT1 + BE4 mRNA

	T_{-10}	T_{-9}	T_{-8}	T_{-7}	C_{-6}	C_{-5}	T_{-4}	T_{-3}	A_{-2}	C_{-1}	T_{1}	T_{2}	G_{3}	G_{4}	G_{5}	A_{6}	T_{7}	C_{8}	T_{9}	T_{10}	T_{11}	A_{12}	A_{13}	A_{14}	A_{15}	C_{16}	C_{17}	A_{18}	A_{19}	G_{20}	G_{21}	G_{22}	G_{23}	Indel
A	0	0	0	0	0	0	0	0	100	0	0	0	0	0	0	99.9	0	0	0	0	0	100	99.9	99.9	99.8	0	0	99.9	99.9	0	0	0	0	0
C	0.1	0	0.1	0	99.9	100	0.2	0	0	100	0.1	0	0	0	0	0	0.1	100	0.1	0	0	0	0	0	0	100	100	0	0	0	0	0	0	
G	0	0	0	0	0	0	0	0	0	0	0	0	99.9	99.9	99.9	0.1	0	0	0	0	0	0	0	0	0.1	0	0	0.1	0.1	100	100	100	100	
T	99.9	100	99.9	100	0	0	99.8	100	0	0	99.9	100	0	0	0	0	99.9	0	99.9	100	100	0	0	0	0	0	0	0	0	0	0	0	0	

290. A TRAC Ex.3 SA OT2 + BE4 mRNA

	A_{-10}	G_{-9}	T_{-8}	A_{-7}	G_{-6}	G_{-5}	T_{-4}	A_{-3}	T_{-2}	C_{-1}	T_{1}	G_{2}	A_{3}	G_{4}	C_{5}	A_{6}	T_{7}	C_{8}	T_{9}	G_{10}	T_{11}	A_{12}	A_{13}	A_{14}	A_{15}	C_{16}	C_{17}	A_{18}	A_{19}	G_{20}	T_{21}	G_{22}	G_{23}	Indel
A	99.8	0	0	99.9	0	0	0	99.9	0	0	0	0	99.8	0	0	100	0	0	0	0	0	100	99.9	99.9	99.8	0	0	99.9	99.9	0	0	0	0	0
C	0	0	0	0	0	0	0.1	0	0.1	99.9	0.1	0	0.1	0	99.9	0	0	99.9	0.1	0	0	0	0	0	0	100	100	0	0	0	0	0	0	
G	0.2	100	0	0.1	100	100	0	0	0	0	0	100	0.1	100	0	0	0	0	0	100	0	0	0.1	0	0.2	0	0	0	0.1	100	0	100	100	
T	0	0	100	0	0	0	99.9	0	99.9	0.1	99.9	0	0	0	0	0	99.9	0.1	99.9	0	99.9	0	0	0	0	0	0	0	0	0	99.9	0	0	

291. A TRAC Ex.3 SA OT3 + BE4 mRNA

	G_{-10}	G_{-9}	C_{-8}	T_{-7}	T_{-6}	C_{-5}	A_{-4}	G_{-3}	C_{-2}	C_{-1}	T_{1}	T_{2}	C_{3}	T_{4}	A_{5}	A_{6}	T_{7}	C_{8}	T_{9}	C_{10}	T_{11}	A_{12}	A_{13}	A_{14}	A_{15}	C_{16}	C_{17}	A_{18}	A_{19}	G_{20}	T_{21}	G_{22}	G_{23}	Indel
A	0	0	0	0	0	0	99.9	0.2	0	0	0	0	0.1	0	100	100	0	0	0.1	0	0	100	100	100	99.9	0	0	99.9	99.9	0	0	0.1	0.1	0.1
C	0	0	100	0.1	0	100	0	0	100	100	0.1	0.1	99.9	0	0	0	0	100	0.4	100	0	0	0	0	0	100	100	0	0	0	0.1	0	0	
G	99.5	99.7	0	0	0	0	0	99.4	0	0	0	0	0	0	0	0	0	0	0	0	0	0	0	0	0	0	0	0	0.1	100	0.1	99.6	99.8	
T	0.4	0.3	0	99.8	100	0	0	0.4	0	0	99.8	99.8	0	99.9	0	0	100	0	99.5	0	100	0	0	0	0	0	0	0	0	0	99.8	0.3	0.2	

292. A TRAC Ex.3 SA OT4 + BE4 mRNA

	A_{-10}	C_{-9}	A_{-8}	G_{-7}	T_{-6}	G_{-5}	T_{-4}	T_{-3}	T_{-2}	A_{-1}	G_1	T_2	G_3	G_4	T_5	A_6	T_7	C_8	T_9	G_{10}	C_{11}	A_{12}	A_{13}	A_{14}	A_{15}	C_{16}	C_{17}	A_{18}	A_{19}	G_{20}	G_{21}	G_{22}	G_{23}	Indel
A	99.9	0	99.9	0	0	0	0	0	0	100	0	0	0	0.1	0	100	0	0	0	0	0	100	99.8	99.9	99.8	0.1	0	99.8	99.9	0	0.1	0	0.1	0.1
C	0	99.9	0	0	0.2	0	0.2	0.1	0.1	0	0	0.1	0	0	0	0	0.1	100	0.2	0	99.9	0	0	0	0	99.9	100	0	0	0	0	0	0	
G	0.1	0	0.1	100	0.1	100	0.1	0.1	0	0	100	0	99.8	99.9	0	0	0	0	0	99.9	0	0	0.2	0	0.2	0	0	0.1	0.1	99.8	99.8	99.8	99.8	
T	0.1	0	0	0	99.8	0	99.8	99.8	99.9	0	0	99.8	0.1	0	99.9	0	99.9	0	99.8	0.1	0.1	0	0	0	0	0	0	0	0	0.1	0.2	0.1	0.1	

293. A TRAC Ex.3 SA OT5 + BE4 mRNA

	G_{-10}	G_{-9}	C_{-8}	C_{-7}	T_{-6}	C_{-5}	A_{-4}	G_{-3}	T_{-2}	T_{-1}	T_1	C_2	C_3	T_4	C_5	A_6	T_7	G_8	T_9	G_{10}	T_{11}	A_{12}	A_{13}	A_{14}	A_{15}	C_{16}	C_{17}	A_{18}	A_{19}	G_{20}	A_{21}	G_{22}	G_{23}	Indel
A	0.1	0	0	0.1	0	0	99.9	0	0	0	0	0	0	0	0	99.8	0	0	0	0	0	99.9	99.8	99.8	99.8	0	0	99.9	99.8	0	99.7	0	0	0
C	0	0	99.9	99.9	0.2	100	0	0	0.1	0.2	0.1	100	99.9	0.2	100	0	0.1	0	0.1	0	0.1	0	0	0.1	0	100	100	0	0	0	0	0	0	
G	99.9	99.9	0	0	0	0	0.1	99.9	0	0	0	0	0	0	0	0.1	0	99.9	0	99.9	0	0	0.2	0.1	0.2	0	0	0.1	0.2	99.9	0.2	99.9	99.9	
T	0	0.1	0	0	99.8	0	0	0	99.8	99.8	99.9	0	0	99.8	0	0	99.9	0	99.8	0	99.9	0	0	0	0	0	0	0	0	0	0	0.1	0.1	

294. A TRAC Ex.3 SA OT6 + BE4 mRNA

	G_{-10}	A_{-9}	T_{-8}	A_{-7}	A_{-6}	G_{-5}	A_{-4}	A_{-3}	G_{-2}	T_{-1}	T_1	C_2	T_3	G_4	C_5	A_6	T_7	C_8	T_9	T_{10}	T_{11}	A_{12}	A_{13}	A_{14}	A_{15}	C_{16}	C_{17}	A_{18}	A_{19}	G_{20}	T_{21}	G_{22}	G_{23}	Indel
A	0	99.9	0	100	99.9	0	100	99.9	0	0	0.1	0	0	0	0	100	0	0	0	0	0	100	100	99.9	99.8	0	0	99.9	99.9	0	0	0	0	0
C	0	0	0	0	0	0	0	0	0	0.1	0	99.9	0.1	0	99.9	0	0.1	100	0.1	0	0	0	0	0	0	100	99.9	0	0	0	0.1	0	0	
G	99.9	0	0	0	0.1	100	0	0.1	99.9	0	0	0	0.1	99.9	0	0	0	0	0	0	0	0	0	0	0.1	0	0	0	0.1	99.9	0	99.9	99.9	
T	0	0	100	0	0	0	0	0	0	99.9	99.8	0	99.8	0.1	0	0	99.9	0	99.8	99.9	99.9	0	0	0	0	0	0	0	0	0	99.9	0.1	0.1	

295. A TRAC Ex.3 SA OT7 + BE4 mRNA

	C_{-10}	A_{-9}	T_{-8}	A_{-7}	T_{-6}	G_{-5}	T_{-4}	C_{-3}	T_{-2}	T_{-1}	T_1	T_2	T_3	G_4	T_5	A_6	T_7	C_8	T_9	T_{10}	T_{11}	A_{12}	A_{13}	A_{14}	A_{15}	C_{16}	C_{17}	A_{18}	T_{19}	G_{20}	T_{21}	G_{22}	G_{23}	Indel
A	0.5	99.9	0	100	0	0	0	0.5	0.1	0	0	0	0	0.1	0	99.9	0.1	0.2	0.1	0	0	100	99.9	99.9	99.8	0.2	0.4	99.9	0.1	0.1	0	0.2	0.1	0
C	99.3	0	0	0	0.1	0	0.2	99.4	0.2	0	0	0	0	0	0.1	0	0.1	99.8	0.1	0	0	0	0	0	0	99.8	99.5	0	0.1	0	0	0	0	
G	0	0	0	0	0	99.8	0	0	0.2	0.1	0.1	0	0	99.6	0	0	0.1	0	0.1	0	0	0	0	0	0.1	0	0	0.1	0	99.8	0.1	99.4	99.8	
T	0.1	0	99.9	0	99.9	0.1	99.8	0.1	99.6	99.8	99.9	99.9	99.9	0.3	99.9	0	99.7	0	99.7	99.9	100	0	0	0	0	0	0.1	0	99.8	0.1	99.8	0.4	0.1	

296. A TRAC Ex.3 SA OT8 + BE4 mRNA

	T_{-10}	G_{-9}	G_{-8}	A_{-7}	G_{-6}	A_{-5}	T_{-4}	G_{-3}	C_{-2}	T_{-1}	T_1	T_2	C_3	T_4	T_5	A_6	T_7	G_8	T_9	G_{10}	T_{11}	G_{12}	A_{13}	A_{14}	A_{15}	C_{16}	C_{17}	A_{18}	A_{19}	G_{20}	G_{21}	G_{22}	G_{23}	Indel
A	0	0	0	99.8	0	100	0	0.1	0	0	0	0	0.1	0	0	100	0	0	0	0	0	0	99.9	100	99.8	0	0.1	99.9	99.9	0	0	0.1	0	0
C	0.1	0	0	0	0	0	0	0	100	0.2	0	0	99.9	0.1	0	0	0	0	0	0	0.2	0	0	0	0	99.9	99.9	0	0	0	0	0	0	
G	0.1	99.8	99.9	0.1	100	0	0	99.6	0	0.1	0.1	0	0	0	0	0	0	100	0	99.9	0	99.9	0.1	0	0.1	0	0	0	0	100	99.9	99.7	99.9	
T	99.7	0.2	0	0.1	0	0	99.9	0.2	0	99.7	99.8	100	0	99.8	100	0	100	0	100	0.1	99.8	0.1	0	0	0	0	0	0	0	0	0.1	0.2	0.1	

297. A TRAC Ex.3 SA OT9 + BE4 mRNA

	G_{-10}	G_{-9}	A_{-8}	C_{-7}	T_{-6}	G_{-5}	G_{-4}	C_{-3}	A_{-2}	T_{-1}	C_1	T_2	C_3	T_4	C_5	T_6	T_7	C_8	T_9	G_{10}	T_{11}	A_{12}	A_{13}	A_{14}	A_{15}	C_{16}	C_{17}	A_{18}	A_{19}	G_{20}	A_{21}	G_{22}	G_{23}	Indel
A	0.2	0.1	99.7	0	0	0.1	0.1	0.1	99.9	0	0.1	0	0.1	0	0.1	0	0	0	0	0	0	99.9	99.8	99.8	99.7	0	0.1	99.9	99.8	0	99.8	0.1	0.1	0
C	0	0	0.1	99.9	0.1	0	0	99.9	0	0.2	99.9	0.2	99.9	0.2	99.9	0.1	0	99.9	0.1	0	0	0	0.1	0.1	0.1	99.9	99.9	0	0.1	0	0.1	0	0	
G	99.5	99.7	0.2	0	0	99.6	99.8	0	0	0	0	0	0	0.1	0	0	0	0	0	99.9	0.1	0	0.1	0	0.2	0	0	0	0.1	99.9	0.1	99.8	99.8	
T	0.4	0.3	0	0	99.8	0.3	0.1	0	0	99.8	0	99.8	0	99.6	0	99.8	99.9	0	99.9	0	99.9	0	0	0	0	0	0	0	0	0.1	0	0.1	0.1	

298. A TRAC Ex.3 SA OT10 + BE4 mRNA

	T_{-10}	G_{-9}	G_{-8}	C_{-7}	T_{-6}	C_{-5}	T_{-4}	T_{-3}	T_{-2}	A_{-1}	T_1	G_2	G_3	G_4	T_5	T_6	T_7	C_8	T_9	T_{10}	T_{11}	A_{12}	A_{13}	A_{14}	A_{15}	C_{16}	C_{17}	A_{18}	A_{19}	G_{20}	G_{21}	G_{22}	G_{23}	Indel
A	0	0	0.1	0.1	0	0.1	0	0	0	100	0	0	0	0	0	0	0	0.1	0	0	0	99.9	99.9	99.9	99.8	0.2	0.1	99.9	99.8	0	0	0.1	0	0
C	0	0	0	99.8	0.2	99.9	0.1	0	0	0	0.1	0	0	0	0.1	0	0	99.9	0.1	0	0.2	0	0	0	0	99.8	99.9	0	0	0	0	0	0	
G	0	99.8	99.8	0.1	0.1	0	0.2	0.1	0	0	0	99.8	99.8	99.9	0.1	0	0	0	0.1	0	0	0	0.1	0.1	0.1	0	0	0.1	0.1	99.9	99.9	99.8	99.9	
T	99.9	0.1	0.1	0	99.7	0	99.7	99.9	100	0	99.9	0.1	0.1	0.1	99.8	99.9	99.9	0	99.8	99.9	99.8	0	0	0	0	0	0	0	0	0	0.1	0.1	0	

299. A PDCD1 Ex.1 SD OnT + Pulse

	C_{-10}	C_{-9}	G_{-8}	C_{-7}	C_{-6}	G_{-5}	A_{-4}	C_{-3}	C_{-2}	C_{-1}	C_1	A_2	C_3	C_4	T_5	A_6	C_7	C_8	T_9	A_{10}	A_{11}	G_{12}	A_{13}	A_{14}	C_{15}	C_{16}	A_{17}	T_{18}	C_{19}	C_{20}	T_{21}	G_{22}	G_{23}	Indel
A	0	0	0	0	0	0	99.6	0	0	0	0.1	99.8	0	0	0	99.9	0	0	0	99.9	99.8	0	99.8	99.8	0	0	99.7	0	0	0	0	0	0	0
C	100	99.9	0	99.9	99.9	0	0.2	99.9	99.9	100	99.9	0.1	100	100	0	0.1	99.9	99.9	0	0.1	0.1	0	0.1	0.1	100	100	0.2	0.1	100	99.9	0	0	0	
G	0	0	99.9	0	0	99.9	0.1	0	0	0	0	0.1	0	0	0	0	0	0	0.1	0	0.1	99.9	0.1	0	0	0	0.1	0.2	0	0	0.1	100	99.9	
T	0	0	0	0	0	0	0.1	0	0	0	0	0	0	0	99.9	0	0	0	99.9	0	0	0	0	0	0	0	0	99.7	0	0	99.9	0	0	

300. A PDCD1 Ex.1 SD OT1 + Pulse

	G_{-10}	T_{-9}	G_{-8}	T_{-7}	C_{-6}	C_{-5}	A_{-4}	T_{-3}	G_{-2}	C_{-1}	C_{1}	G_{2}	G_{3}	C_{4}	C_{5}	A_{6}	C_{7}	C_{8}	T_{9}	G_{10}	A_{11}	G_{12}	A_{13}	A_{14}	C_{15}	C_{16}	A_{17}	T_{18}	C_{19}	C_{20}	T_{21}	G_{22}	G_{23}	Indel
A	0	0	0.1	0	0.1	0	96.5	0	0.1	0.1	0	0.1	0.1	0.1	0	98.6	0.2	0	0	0.1	98	0.2	99.5	99.7	0.6	0.1	99.3	0.4	0.1	0	0	0.2	0.1	0
C	0.1	0.1	0.1	0.3	99.8	99.9	3.5	0	0.1	99.8	99.9	0.6	0	99.8	99.9	1.2	99.8	99.9	0.3	0.1	0.9	0.1	0.3	0.2	99.3	99.9	0.5	0.2	99.8	100	0.3	0.2	0.1	
G	98.1	0.5	97.2	0.2	0	0	0	0.1	98.3	0	0	99	99.7	0	0	0.1	0	0	0.2	98.4	0.7	98.8	0.1	0.1	0	0	0.1	0.2	0	0	0.1	96.9	99.3	
T	1.8	99.4	2.6	99.5	0.2	0	0	99.8	1.5	0.1	0.1	0.4	0.1	0	0	0.1	0	0	99.6	1.4	0.4	1	0	0	0	0	0.1	99.3	0.1	0	99.6	2.8	0.4	

301. A PDCD1 Ex.1 SD OT2 + Pulse

	G_{-10}	T_{-9}	C_{-8}	T_{-7}	G_{-6}	A_{-5}	C_{-4}	T_{-3}	G_{-2}	C_{-1}	A_{1}	C_{2}	C_{3}	T_{4}	G_{5}	A_{6}	C_{7}	C_{8}	T_{9}	A_{10}	A_{11}	G_{12}	A_{13}	A_{14}	C_{15}	C_{16}	A_{17}	T_{18}	C_{19}	C_{20}	A_{21}	G_{22}	G_{23}	Indel
A	0	0	0	0	0	99.8	0	0	0	0	99.9	0	0	0	0	99.8	0	0	0	100	99.9	0	99.8	99.9	0	0	99.8	0	0	0	99.9	0.1	0	0
C	0	0.2	100	0.1	0	0	100	0.1	0	99.9	0	99.9	100	0.1	0	0	99.9	100	0.1	0	0	0	0	0	99.9	100	0	0.1	99.9	99.9	0	0	0	
G	100	0	0	0	99.9	0.2	0	0	99.9	0	0.1	0	0	0	99.9	0.2	0	0	0	0	0.1	100	0.1	0.1	0	0	0.1	0	0	0	0.1	99.9	99.9	
T	0	99.8	0	99.9	0	0	0	99.8	0	0	0	0	0	99.8	0.1	0	0	0	99.9	0	0	0	0	0	0	0	0	99.9	0	0	0	0	0	

302. A PDCD1 Ex.1 SD OT3 + Pulse

	A_{-10}	A_{-9}	C_{-8}	T_{-7}	C_{-6}	T_{-5}	A_{-4}	G_{-3}	C_{-2}	C_{-1}	C_{1}	T_{2}	T_{3}	C_{4}	T_{5}	A_{6}	T_{7}	C_{8}	T_{9}	C_{10}	A_{11}	G_{12}	A_{13}	A_{14}	C_{15}	C_{16}	A_{17}	T_{18}	C_{19}	C_{20}	A_{21}	G_{22}	G_{23}	Indel
A	99.9	99.9	0	0	0	0	99.9	0	0	0	0	0	0	0	0	100	0	0	0.1	0	99.9	0	99.8	99.9	0	0	99.9	0	0	0	99.8	0	0	0
C	0	0	100	0.1	100	0.1	0	0	99.9	100	100	0.1	0.1	100	0.1	0	0.2	100	0.2	99.9	0	0	0	0	100	99.9	0	0.1	99.9	99.9	0	0	0	
G	0.1	0.1	0	0	0	0	0.1	99.9	0	0	0	0	0	0	0	0	0.1	0	0.1	0	0.1	99.9	0.1	0.1	0	0	0.1	0	0	0	0.2	99.9	99.9	
T	0	0	0	99.9	0	99.9	0	0	0	0	0	99.8	99.9	0	99.9	0	99.7	0	99.6	0	0	0.1	0	0	0	0	0	99.9	0	0	0	0	0	

303. A PDCD1 Ex.1 SD OT4 + Pulse

	G_{-10}	A_{-9}	A_{-8}	G_{-7}	T_{-6}	G_{-5}	C_{-4}	C_{-3}	C_{-2}	A_{-1}	T_{1}	A_{2}	C_{3}	C_{4}	C_{5}	A_{6}	G_{7}	C_{8}	T_{9}	C_{10}	A_{11}	G_{12}	A_{13}	A_{14}	C_{15}	C_{16}	A_{17}	T_{18}	C_{19}	C_{20}	T_{21}	G_{22}	G_{23}	Indel
A	0	99.8	99.8	0	0	0.1	0	0	0	99.9	0	99.9	0.1	0	0.1	99.8	0.1	0	0	0	99.9	0	99.8	99.8	0	0.1	99.9	0.1	0.1	0	0	0	0	0
C	0	0	0	0	0	0	99.9	99.9	99.9	0	0	0	99.9	99.9	99.9	0	0	99.9	0.2	100	0	0	0	0.1	99.9	99.9	0	0.1	99.9	100	0.1	0	0	
G	99.9	0.2	0.2	100	0	99.7	0	0	0	0.1	0	0.1	0	0	0	0.1	99.9	0	0.1	0	0.1	99.9	0.1	0.2	0	0	0.1	0	0	0	0	99.9	99.8	
T	0.1	0	0	0	99.9	0.2	0.1	0	0	0	99.9	0	0	0	0	0	0	0	99.7	0	0	0	0	0	0	0	0	99.8	0	0	99.8	0.1	0.1	

304. A PDCD1 Ex.1 SD OT5 + Pulse

	G_{-10}	T_{-9}	T_{-8}	C_{-7}	C_{-6}	C_{-5}	A_{-4}	T_{-3}	C_{-2}	A_{-1}	C_{1}	A_{2}	C_{3}	T_{4}	C_{5}	A_{6}	C_{7}	T_{8}	T_{9}	A_{10}	A_{11}	G_{12}	T_{13}	A_{14}	C_{15}	C_{16}	A_{17}	T_{18}	C_{19}	C_{20}	T_{21}	G_{22}	G_{23}	Indel
A	0	0	0	0.1	0.1	0.1	99.9	0	0.1	99.8	0.1	99.9	0	0	0.2	99.8	0	0	0	99.9	99.8	0	0	99.9	0.1	0.3	99.9	0	0.1	0.1	0	0	0	0
C	0	0.1	0.2	99.8	99.9	99.8	0	0.1	99.9	0	99.9	0	99.9	0.2	99.8	0	99.9	0.1	0	0	0	0	0.1	0	99.8	99.6	0	0.1	99.9	99.8	0.1	0	0	
G	99.7	0	0	0	0	0	0.1	0	0	0.2	0	0.1	0	0	0	0.1	0	0	0	0.1	0.1	99.9	0	0	0	0	0.1	0	0	0	0	99.8	99.7	
T	0.2	99.9	99.8	0.1	0	0	0	99.8	0	0	0	0	0	99.8	0	0	0	99.9	99.9	0	0	0.1	99.8	0	0	0.1	0	99.9	0	0	99.9	0.2	0.2	

305. A PDCD1 Ex.1 SD OT6 + Pulse

	C_{-10}	T_{-9}	T_{-8}	G_{-7}	G_{-6}	A_{-5}	A_{-4}	A_{-3}	A_{-2}	A_{-1}	C_{1}	A_{2}	C_{3}	A_{4}	A_{5}	A_{6}	C_{7}	C_{8}	T_{9}	G_{10}	A_{11}	G_{12}	A_{13}	A_{14}	C_{15}	C_{16}	A_{17}	T_{18}	C_{19}	G_{20}	A_{21}	G_{22}	G_{23}	Indel
A	0.1	0	0	0.1	0	99.7	99.8	99.8	99.8	99.7	0	99.8	0	99.8	99.8	99.8	0.1	0.1	0	0	99.5	0	99.7	99.8	0	0.1	99.8	0	0.1	0.1	99.7	0	0	0
C	99.8	0.1	0.1	0	0	0	0	0	0	0.1	99.9	0	99.9	0	0	0	99.9	99.9	0.2	0	0.1	0	0	0	0	99.9	0	0.1	99.8	0	0	0	0	
G	0	0.1	0	99.9	100	0.2	0.2	0.2	0.2	0.2	0	0.2	0	0.2	0.2	0.2	0	0	0.1	99.9	0.4	99.9	0.2	0.2	99.9	0	0.1	0.1	0	99.9	0.2	99.9	100	
T	0	99.8	99.8	0	0	0	0	0	0	0	0	0	0	0	0	0	0	0	99.7	0.1	0.1	0	0	0	0	0	0	99.8	0.1	0	0	0	0	

306. A PDCD1 Ex.1 SD OT7 + Pulse

	T_{-10}	C_{-9}	C_{-8}	A_{-7}	T_{-6}	C_{-5}	T_{-4}	C_{-3}	A_{-2}	C_{-1}	C_{1}	A_{2}	C_{3}	C_{4}	T_{5}	C_{6}	C_{7}	A_{8}	T_{9}	T_{10}	T_{11}	G_{12}	A_{13}	A_{14}	C_{15}	C_{16}	A_{17}	T_{18}	C_{19}	C_{20}	T_{21}	G_{22}	G_{23}	Indel
A	0	0	0	99.9	0	0	0	0	99.8	0	0	99.8	0	0	0	0	0	99.9	0	0	0	0	99.8	99.9	0	0	99.9	0	0	0	0	0	0	0
C	0.1	100	100	0	0.2	100	0.1	100	0	100	99.9	0	100	100	0.2	99.6	100	0	0.1	0	0	0	0	0	100	100	0	0.1	100	100	0.1	0	0.1	
G	0	0	0	0.1	0	0	0.1	0	0.1	0	0	0.1	0	0	0.1	0	0	0	0	0	0	99.9	0.1	0.1	0	0	0	0	0	0	0	99.9	99.8	
T	99.8	0	0	0	99.8	0	99.8	0	0	0	0	0	0	0	99.8	0.4	0	0	99.9	99.9	99.9	0.1	0	0	0	0	0	99.9	0	0	99.9	0.1	0.1	

307. A PDCD1 Ex.1 SD OT8 + Pulse

	A_{-10}	A_{-9}	A_{-8}	A_{-7}	A_{-6}	G_{-5}	C_{-4}	A_{-3}	A_{-2}	C_{-1}	C_{1}	A_{2}	C_{3}	C_{4}	C_{5}	A_{6}	C_{7}	C_{8}	T_{9}	A_{10}	A_{11}	G_{12}	C_{13}	A_{14}	C_{15}	C_{16}	A_{17}	T_{18}	C_{19}	T_{20}	A_{21}	G_{22}	G_{23}	Indel
A	100	100	99.9	99.9	99.8	0	0	99.8	99.8	0	0.1	99.9	0	0.1	0.2	99.9	0	0	0	100	99.9	0	0	99.9	0	0	99.9	0	0	0	99.9	0	0	0
C	0	0	0.1	0	0	0	99.9	0	0	99.9	99.9	0	99.9	99.9	99.8	0	99.9	100	0	0	0	0	99.9	0	100	100	0	0.1	100	0	0	0	0	
G	0	0	0	0	0.2	99.9	0	0.1	0.1	0	0	0.1	0	0	0	0.1	0	0	0	0	0.1	99.9	0	0.1	0	0	0	0	0	0	0	99.9	99.9	
T	0	0	0	0	0	0	0	0	0	0	0	0	0	0	0	0	0	0	99.9	0	0	0	0	0	0	0	0	99.8	0	99.9	0	0.1	0	

308. A PDCD1 Ex.1 SD OT9 + Pulse

	G_{-10}	A_{-9}	T_{-8}	G_{-7}	G_{-6}	A_{-5}	T_{-4}	C_{-3}	A_{-2}	C_{-1}	C_{1}	A_{2}	C_{3}	C_{4}	T_{5}	T_{6}	C_{7}	A_{8}	T_{9}	C_{10}	A_{11}	G_{12}	A_{13}	A_{14}	C_{15}	C_{16}	A_{17}	T_{18}	C_{19}	T_{20}	G_{21}	G_{22}	G_{23}	Indel
A	0	99.8	0	0	0	99.6	0	0	99.8	0.1	0.1	99.8	0	0	0	0	0	99.9	0	0	99.9	0	99.9	99.8	0	0	99.9	0	0	0	0	0	0.1	0
C	0	0	0	0	0	0.1	0	99.9	0.1	99.9	99.9	0.1	99.9	99.9	0.1	0.1	99.9	0	0.1	100	0	0	0	0	99.9	99.9	0	0.1	99.9	0	0	0	0	
G	99.9	0.1	0.1	99.8	99.8	0.2	0.1	0	0.1	0	0	0.2	0	0	0.1	0.1	0	0	0.1	0	0.1	99.9	0.1	0.1	0	0	0	0.1	0	0	99.8	99.9	99.8	
T	0	0.1	99.8	0.2	0.2	0.2	99.8	0	0	0	0	0	0	0	99.8	99.8	0	0	99.8	0	0	0.1	0	0	0	0	0	99.8	0	99.9	0.1	0.1	0.1	

309. A PDCD1 Ex.1 SD OT10 + Pulse

	G_{-10}	T_{-9}	T_{-8}	C_{-7}	C_{-6}	A_{-5}	G_{-4}	A_{-3}	A_{-2}	C_{-1}	C_{1}	A_{2}	G_{3}	C_{4}	T_{5}	A_{6}	T_{7}	C_{8}	T_{9}	C_{10}	A_{11}	G_{12}	A_{13}	A_{14}	C_{15}	C_{16}	T_{17}	T_{18}	C_{19}	C_{20}	T_{21}	G_{22}	G_{23}	Indel
A	0	0	0	0	0	99.9	0	99.8	99.8	0	0.1	99.8	0	0	0	99.9	0	0	0	0	99.8	0	99.9	99.9	0	0	0	0	0	0	0	0	0	0
C	0	0.1	0.1	100	99.9	0	0	0	0	99.9	99.9	0	0	100	0.1	0	0.2	100	0.2	100	0	0	0	0	100	99.6	0.1	0.1	99.9	100	0.1	0	0	
G	99.9	0	0	0	0	0.1	100	0.1	0.2	0	0	0.1	99.9	0	0	0	0	0	0	0	0.1	100	0.1	0.1	0	0	0	0	0	0	0	99.9	100	
T	0	99.9	99.9	0	0	0	0	0	0	0	0	0	0	0	99.9	0	99.8	0	99.8	0	0	0	0	0	0	0.4	99.8	99.9	0	0	99.8	0	0	

310. A PDCD1 Ex.1 SD OnT + Cas9 mRNA

	C_{-10}	C_{-9}	G_{-8}	C_{-7}	C_{-6}	G_{-5}	A_{-4}	C_{-3}	C_{-2}	C_{-1}	C_{1}	A_{2}	C_{3}	C_{4}	T_{5}	A_{6}	C_{7}	C_{8}	T_{9}	A_{10}	A_{11}	G_{12}	A_{13}	A_{14}	C_{15}	C_{16}	A_{17}	T_{18}	C_{19}	C_{20}	T_{21}	G_{22}	G_{23}	Indel
A	0	0	0	0	0	0	99.3	0	0	0	0	99.9	0	0	0	99.5	0.1	0	0	99.8	99.5	0	99.4	99.5	0.2	0.4	98	0.2	0	0	0.1	0.1	0	84.6
C	100	99.9	0	100	100	0	0.6	99.9	100	99.9	99.9	0	99.8	99.7	0	0.3	99.2	99.3	0.1	0	0.2	0	0.2	0.4	99.6	99.4	1.2	0.3	99.5	99.6	0.2	0	0.1	
G	0	0	99.9	0	0	99.9	0.1	0	0	0	0	0	0	0	0	0	0	0.1	0	0	0.2	99.9	0.1	0.1	0.3	0.1	0.5	0.3	0.3	0.2	0.2	99.7	99.9	
T	0	0.1	0	0	0	0	0	0	0	0	0	0	0.2	0.2	99.9	0.2	0.6	0.6	99.9	0.1	0.2	0	0.3	0.1	0	0.2	0.4	99.2	0.2	0.2	99.5	0.1	0	

311. A PDCD1 Ex.1 SD OT1 + Cas9 mRNA

	G_{-10}	T_{-9}	G_{-8}	T_{-7}	C_{-6}	C_{-5}	A_{-4}	T_{-3}	G_{-2}	C_{-1}	C_{1}	G_{2}	G_{3}	C_{4}	C_{5}	A_{6}	C_{7}	C_{8}	T_{9}	G_{10}	A_{11}	G_{12}	A_{13}	A_{14}	C_{15}	C_{16}	A_{17}	T_{18}	C_{19}	C_{20}	T_{21}	G_{22}	G_{23}	Indel
A	0	0	0	0	0	0	98.7	0	0.1	0	0	0.1	0	0	0	99.7	0	0	0	0.1	99.7	0	99.6	99.8	0.1	0	99.8	0	0	0	0	0.1	0.1	0
C	0	0.1	0	0.2	100	100	1.2	0.1	0.1	99.9	99.9	0.3	0	99.9	99.9	0.2	100	100	0.2	0	0.1	0	0.2	0.1	99.8	100	0.2	0.2	100	100	0.1	0.1	0.2	
G	99.6	0	99.3	0	0	0	0.1	0	99.5	0	0	99.5	99.8	0	0	0.1	0	0	0	99.7	0.2	99.9	0.2	0.1	0	0	0.1	0	0	0	0	98.5	99.3	
T	0.4	99.8	0.6	99.7	0	0	0	99.9	0.3	0.1	0.1	0.1	0.2	0.1	0	0	0	0	99.8	0.2	0	0.1	0	0	0	0	0	99.8	0	0	99.9	1.3	0.4	

312. A PDCD1 Ex.1 SD OT2 + Cas9 mRNA

	G_{-10}	T_{-9}	C_{-8}	T_{-7}	G_{-6}	A_{-5}	C_{-4}	T_{-3}	G_{-2}	C_{-1}	A_{1}	C_{2}	C_{3}	T_{4}	G_{5}	A_{6}	C_{7}	C_{8}	T_{9}	A_{10}	A_{11}	G_{12}	A_{13}	A_{14}	C_{15}	C_{16}	A_{17}	T_{18}	C_{19}	C_{20}	A_{21}	G_{22}	G_{23}	Indel
A	0	0	0	0	0	99.8	0	0	0	0.1	99.9	0.1	0.1	0	0	99.8	0	0	0	99.9	99.8	0	99.8	99.9	0.1	0.1	99.6	0.2	0	0	99.9	0.1	0	13.1
C	0	0.1	100	0	0	0	100	0.1	0	99.8	0	99.8	99.9	0.1	0	0	99.9	99.9	0.1	0	0	0	0.1	0	99.8	99.8	0.2	0	99.8	99.9	0	0	0	
G	100	0.1	0	0	100	0.2	0	0	99.9	0	0	0	0	0	99.9	0.1	0	0	0	0.1	0.1	100	0.2	0.1	0	0.1	0.1	0.1	0	0	0	99.8	99.9	
T	0	99.8	0	99.9	0	0	0	99.9	0	0.1	0	0.1	0	99.9	0.1	0	0	0	99.9	0	0	0	0	0	0.1	0	0	99.7	0.2	0	0	0.1	0	

313. A PDCD1 Ex.1 SD OT3 + Cas9 mRNA

	A_{-10}	A_{-9}	C_{-8}	T_{-7}	C_{-6}	T_{-5}	A_{-4}	G_{-3}	C_{-2}	C_{-1}	C_{1}	T_{2}	T_{3}	C_{4}	T_{5}	A_{6}	T_{7}	C_{8}	T_{9}	C_{10}	A_{11}	G_{12}	A_{13}	A_{14}	C_{15}	C_{16}	A_{17}	T_{18}	C_{19}	C_{20}	A_{21}	G_{22}	G_{23}	Indel
A	99.9	99.9	0	0	0	0	99.9	0	0	0.1	0	0	0	0	0	99.9	0	0	0	0	99.9	0	99.8	99.8	0.1	0	99.9	0	0.1	0.1	99.8	0	0	0
C	0	0	100	0.1	99.9	0.1	0	0	99.9	99.9	99.9	0.2	0.1	100	0.1	0	0.1	100	0.2	99.9	0	0	0	0	99.9	99.9	0	0.1	99.9	99.9	0	0	0	
G	0.1	0.1	0	0	0	0	0	99.8	0	0	0	0.1	0	0	0	0	0.1	0	0.1	0	0.1	99.9	0.1	0.2	0	0	0.1	0.1	0	0	0.1	99.9	99.9	
T	0	0	0	99.9	0	99.9	0	0.1	0	0	0	99.8	99.8	0	99.9	0	99.7	0	99.7	0	0	0.1	0	0	0	0	0	99.8	0	0	0	0	0	

314. A PDCD1 Ex.1 SD OT4 + Cas9 mRNA

	G_{-10}	A_{-9}	A_{-8}	G_{-7}	T_{-6}	G_{-5}	C_{-4}	C_{-3}	C_{-2}	A_{-1}	T_{1}	A_{2}	C_{3}	C_{4}	C_{5}	A_{6}	G_{7}	C_{8}	T_{9}	C_{10}	A_{11}	G_{12}	A_{13}	A_{14}	C_{15}	C_{16}	A_{17}	T_{18}	C_{19}	C_{20}	T_{21}	G_{22}	G_{23}	Indel
A	0	99.8	99.8	0	0	0.1	0.1	0	0.1	99.9	0	99.9	0.1	0	0.1	99.9	0	0	0	0	99.9	0	99.8	99.8	0.1	0.1	99.8	0.1	0.1	0	0	0	0	0
C	0	0	0	0	0	0	99.8	99.9	99.9	0	0	0	99.8	99.9	99.9	0	0	99.9	0.2	99.9	0	0	0	0.1	99.9	99.9	0.1	0.1	99.9	100	0.2	0	0	
G	99.9	0.1	0.2	100	0	99.7	0	0	0	0.1	0	0.1	0	0	0	0.1	99.9	0	0.1	0	0	99.9	0.2	0.1	0	0	0	0	0	0	0	99.9	99.9	
T	0	0	0	0	99.9	0.2	0.1	0	0	0	99.9	0	0.1	0	0	0	0.1	0	99.7	0	0	0	0.1	0.1	0	0	0	99.8	0	0	99.8	0	0.1	

315. A PDCD1 Ex.1 SD OT5 + Cas9 mRNA

	G_{-10}	T_{-9}	T_{-8}	C_{-7}	C_{-6}	C_{-5}	A_{-4}	T_{-3}	C_{-2}	A_{-1}	C_{1}	A_{2}	C_{3}	T_{4}	C_{5}	A_{6}	C_{7}	T_{8}	T_{9}	A_{10}	A_{11}	G_{12}	T_{13}	A_{14}	C_{15}	C_{16}	A_{17}	T_{18}	C_{19}	C_{20}	T_{21}	G_{22}	G_{23}	Indel
A	0.1	0	0	0.1	0.1	0.1	99.9	0	0.1	99.8	0.1	99.9	0	0	0.1	99.9	0	0	0	99.9	99.9	0	0	100	0.1	0.3	99.9	0	0.1	0.1	0	0	0.1	0
C	0	0.2	0.1	99.8	99.8	99.8	0	0.2	99.9	0	99.9	0	99.9	0.2	99.8	0	100	0.1	0	0	0	0	0	0	99.8	99.7	0	0	99.9	99.8	0.1	0	0	
G	99.7	0	0	0	0	0	0.1	0	0	0.1	0	0.1	0	0	0	0.1	0	0	0	0	0.1	99.9	0	0	0	0	0.1	0	0	0	0	99.8	99.8	
T	0.2	99.8	99.8	0.1	0	0	0	99.8	0	0	0	0	0	99.8	0	0	0	99.9	99.9	0	0	0.1	99.9	0	0	0	0	99.9	0	0	99.8	0.2	0.2	

316. A PDCD1 Ex.1 SD OT6 + Cas9 mRNA

	C_{-10}	T_{-9}	T_{-8}	G_{-7}	G_{-6}	A_{-5}	A_{-4}	A_{-3}	A_{-2}	A_{-1}	C_{1}	A_{2}	C_{3}	A_{4}	A_{5}	A_{6}	C_{7}	C_{8}	T_{9}	G_{10}	A_{11}	G_{12}	A_{13}	A_{14}	C_{15}	C_{16}	A_{17}	T_{18}	C_{19}	G_{20}	A_{21}	G_{22}	G_{23}	Indel
A	0.1	0	0	0	0	99.8	99.8	99.8	99.8	99.7	0	99.8	0	99.8	99.8	99.8	0.1	0.1	0	0	99.6	0	99.7	99.7	0	0	99.9	0	0.1	0.1	99.7	0	0	0
C	99.9	0.1	0.1	0	0	0	0	0	0	0.1	99.9	0	99.9	0	0	0	99.9	99.9	0.2	0	0.1	0	0	0	0	99.9	0	0.1	99.8	0	0	0	0	
G	0	0.1	0	99.9	100	0.2	0.2	0.2	0.1	0.2	0	0.2	0	0.1	0.2	0.2	0	0	0.1	99.9	0.3	99.9	0.2	0.2	99.9	0	0.1	0.1	0	99.9	0.2	99.9	100	
T	0	99.8	99.8	0	0	0	0	0	0	0	0	0	0	0	0	0	0	0	99.8	0.1	0	0.1	0	0	0	0	0	99.8	0	0	0	0	0	

317. A PDCD1 Ex.1 SD OT7 + Cas9 mRNA

	T_{-10}	C_{-9}	C_{-8}	A_{-7}	T_{-6}	C_{-5}	T_{-4}	C_{-3}	A_{-2}	C_{-1}	C_{1}	A_{2}	C_{3}	C_{4}	T_{5}	C_{6}	C_{7}	A_{8}	T_{9}	T_{10}	T_{11}	G_{12}	A_{13}	A_{14}	C_{15}	C_{16}	A_{17}	T_{18}	C_{19}	C_{20}	T_{21}	G_{22}	G_{23}	Indel
A	0	0	0	99.9	0	0	0	0	99.9	0	0	99.9	0	0	0	0	0	99.9	0	0	0	0	99.8	99.8	0	0	99.9	0	0	0	0	0	0	0
C	0.1	100	99.9	0	0.2	100	0.1	100	0	100	99.9	0	100	99.9	0.2	99.7	100	0	0.1	0	0	0	0	0	100	100	0	0.1	100	99.9	0.1	0	0.1	
G	0.1	0	0	0.1	0	0	0.1	0	0.1	0	0	0.1	0	0	0	0	0	0	0	0	0	99.9	0.1	0.1	0	0	0	0	0	0	0	99.9	99.8	
T	99.8	0	0	0	99.8	0	99.8	0	0	0	0	0	0	0	99.8	0.3	0	0	99.9	99.9	100	0.1	0	0	0	0	0.1	99.9	0	0	99.8	0.1	0.1	

318. A PDCD1 Ex.1 SD OT8 + Cas9 mRNA

	A_{-10}	A_{-9}	A_{-8}	A_{-7}	A_{-6}	G_{-5}	C_{-4}	A_{-3}	A_{-2}	C_{-1}	C_{1}	A_{2}	C_{3}	C_{4}	C_{5}	A_{6}	C_{7}	C_{8}	T_{9}	A_{10}	A_{11}	G_{12}	C_{13}	A_{14}	C_{15}	C_{16}	A_{17}	T_{18}	C_{19}	T_{20}	A_{21}	G_{22}	G_{23}	Indel
A	100	100	99.9	99.9	99.8	0	0	99.9	99.8	0	0.1	99.9	0	0.1	0.1	99.9	0	0	0	100	99.9	0	0	99.9	0	0	99.9	0	0	0	99.9	0	0	0
C	0	0	0	0	0	0	99.9	0	0	100	99.9	0	99.9	99.9	99.8	0	100	100	0	0	0	0	99.9	0	100	100	0	0.1	100	0	0	0	0	
G	0	0	0	0	0.2	99.9	0	0.1	0.1	0	0	0.1	0	0	0	0.1	0	0	0	0	0.1	99.9	0	0.1	0	0	0.1	0	0	0	0	99.9	99.9	
T	0	0	0	0	0	0	0	0	0	0	0	0	0	0	0	0	0	0	99.9	0	0	0	0	0	0	0	0	99.8	0	99.9	0	0	0	

319. A PDCD1 Ex.1 SD OT9 + Cas9 mRNA

	G_{-10}	A_{-9}	T_{-8}	G_{-7}	G_{-6}	A_{-5}	T_{-4}	C_{-3}	A_{-2}	C_{-1}	C_{1}	A_{2}	C_{3}	C_{4}	T_{5}	T_{6}	C_{7}	A_{8}	T_{9}	C_{10}	A_{11}	G_{12}	A_{13}	A_{14}	C_{15}	C_{16}	A_{17}	T_{18}	C_{19}	T_{20}	G_{21}	G_{22}	G_{23}	Indel
A	0	99.8	0	0	0	99.7	0	0.1	99.7	0.1	0.1	99.8	0	0	0	0	0	99.9	0	0	99.8	0	99.9	99.9	0	0	99.9	0	0.1	0	0	0	0.1	0
C	0	0	0	0	0	0.1	0.1	99.9	0.1	99.9	99.9	0.1	99.9	100	0.2	0.1	99.9	0	0.1	99.9	0	0	0	0	99.9	99.9	0.1	0.1	99.9	0	0	0	0	
G	99.9	0.1	0.1	99.8	99.8	0.1	0.1	0	0.2	0	0	0.1	0	0	0.1	0.1	0	0	0.1	0	0.1	99.9	0.1	0.1	0	0	0.1	0.1	0	0	99.8	99.9	99.8	
T	0	0	99.8	0.2	0.1	0.1	99.8	0	0	0	0	0	0	0	99.7	99.8	0	0	99.8	0	0	0.1	0	0	0	0	0	99.8	0	99.9	0.1	0.1	0.1	

320. A PDCD1 Ex.1 SD OT10 + Cas9 mRNA

	G_{-10}	T_{-9}	T_{-8}	C_{-7}	C_{-6}	A_{-5}	G_{-4}	A_{-3}	A_{-2}	C_{-1}	C_{1}	A_{2}	G_{3}	C_{4}	T_{5}	A_{6}	T_{7}	C_{8}	T_{9}	C_{10}	A_{11}	G_{12}	A_{13}	A_{14}	C_{15}	C_{16}	T_{17}	T_{18}	C_{19}	C_{20}	T_{21}	G_{22}	G_{23}	Indel
A	0	0	0	0	0.1	99.8	0	99.8	99.8	0.1	0.1	99.8	0	0	0	99.9	0	0.1	0	0	99.9	0	99.9	99.9	0.1	0.1	0	0	0.1	0.1	0	0	0	0
C	0	0.1	0.1	99.9	99.8	0	0	0	0	99.9	99.8	0	0	99.9	0.1	0	0.2	99.9	0.1	99.9	0	0	0	0	99.9	99.7	0.1	0.1	99.9	99.9	0.2	0	0	
G	99.9	0	0	0	0	0.1	99.9	0.2	0.2	0	0	0.1	99.9	0	0	0	0	0	0	0	0.1	100	0.1	0.1	0	0	0	0	0	0	0	99.9	99.9	
T	0	99.9	99.9	0	0	0	0	0	0	0	0	0	0	0	99.8	0	99.8	0	99.8	0	0	0	0	0	0	0.2	99.8	99.9	0	0	99.8	0.1	0	

321. A PDCD1 Ex.1 SD OnT + BE4 mRNA

	C_{-10}	C_{-9}	G_{-8}	C_{-7}	C_{-6}	G_{-5}	A_{-4}	C_{-3}	C_{-2}	C_{-1}	C_{1}	A_{2}	C_{3}	C_{4}	T_{5}	A_{6}	C_{7}	C_{8}	T_{9}	A_{10}	A_{11}	G_{12}	A_{13}	A_{14}	C_{15}	C_{16}	A_{17}	T_{18}	C_{19}	C_{20}	T_{21}	G_{22}	G_{23}	Indel
A	0.1	0.2	0	0.2	0.2	0.3	99.5	0.2	0.1	0.1	0.4	99.2	0.3	1.6	0.1	99.6	5.7	5.7	0.1	99.7	99.6	0	99.7	99.5	0.1	0.1	99.2	0.1	0.1	0	0	0.1	0	7.3
C	99.8	99.7	0	99.8	99.7	0.1	0.3	99.7	99.8	99.8	99.5	0.7	88	74	0.1	0.3	24.1	29.3	0	0.2	0.4	0	0.2	0.4	99.8	99.8	0.6	0.1	99.7	99.9	0.1	0	0	
G	0	0	99.8	0	0	99.4	0.1	0	0	0	0	0.1	0	0.4	0.2	0	3	8	0.1	0.1	0	99.9	0.1	0	0	0	0.1	0.4	0	0	0.1	99.9	99.9	
T	0.1	0.1	0.1	0.1	0.1	0.2	0.1	0.1	0.1	0.1	0	0.1	11.7	24.1	99.6	0	67.3	57	99.8	0	0	0	0	0	0.1	0	0.1	99.4	0.3	0	99.8	0	0	

322. A PDCD1 Ex.1 SD OT1 + BE4 mRNA

	G_{-10}	T_{-9}	G_{-8}	T_{-7}	C_{-6}	C_{-5}	A_{-4}	T_{-3}	G_{-2}	C_{-1}	C_{1}	G_{2}	G_{3}	C_{4}	C_{5}	A_{6}	C_{7}	C_{8}	T_{9}	G_{10}	A_{11}	G_{12}	A_{13}	A_{14}	C_{15}	C_{16}	A_{17}	T_{18}	C_{19}	C_{20}	T_{21}	G_{22}	G_{23}	Indel
A	0	0	0	0	0.1	0	97.2	0.1	0.1	0	0	0.1	0	0.1	0	99.1	0.1	0	0.1	0	98.6	0.2	99.8	99.8	0.6	0	99.4	0.3	0.1	0	0	0.2	0.1	0
C	0.1	0.2	0	0.2	99.8	99.9	2.7	0	0.1	99.9	99.9	0.3	0	99.8	99.9	0.7	99.9	100	0.3	0.1	0.5	0	0.1	0.1	99.3	99.9	0.5	0.2	99.8	99.9	0.3	0.1	0.1	
G	98.7	0.4	98	0.1	0	0	0.1	0.1	98.8	0	0	99.3	99.8	0	0	0.1	0	0	0.2	99	0.8	99.4	0.1	0.1	0	0	0	0.2	0	0	0	98	99.6	
T	1.2	99.4	2	99.7	0	0.1	0	99.8	1	0.1	0.1	0.2	0.1	0.2	0	0.1	0	0	99.4	0.9	0.2	0.4	0	0	0	0	0.1	99.3	0.1	0.1	99.7	1.8	0.3	

323. A PDCD1 Ex.1 SD OT2 + BE4 mRNA

	G_{-10}	T_{-9}	C_{-8}	T_{-7}	G_{-6}	A_{-5}	C_{-4}	T_{-3}	G_{-2}	C_{-1}	A_{1}	C_{2}	C_{3}	T_{4}	G_{5}	A_{6}	C_{7}	C_{8}	T_{9}	A_{10}	A_{11}	G_{12}	A_{13}	A_{14}	C_{15}	C_{16}	A_{17}	T_{18}	C_{19}	C_{20}	A_{21}	G_{22}	G_{23}	Indel
A	0	0	0	0	0.3	99.8	0	0	0.1	0	99.9	0.1	0	0	0	99.8	0.2	0.1	0	100	99.9	0	99.8	99.9	0	0	99.9	0	0	0	99.9	0.1	0	0.2
C	0	0.2	99	0.1	0.1	0	99.9	0.1	0	99.9	0	99.8	99.9	0.1	0	0	98.7	99.2	0.1	0	0	0	0	0	99.9	100	0	0.1	99.9	99.9	0	0	0	
G	99.9	0	0.2	0	99.6	0.2	0	0	99.9	0	0.1	0	0	0	99.8	0.1	0.2	0.1	0	0	0.1	99.9	0.1	0.1	0	0	0.1	0	0	0	0.1	99.8	99.9	
T	0	99.8	0.8	99.9	0.1	0	0.1	99.8	0	0.1	0	0.1	0.1	99.8	0.1	0	0.9	0.6	99.9	0	0	0.1	0	0	0	0	0	99.8	0.1	0	0	0.1	0	

324. A PDCD1 Ex.1 SD OT3 + BE4 mRNA

	A_{-10}	A_{-9}	C_{-8}	T_{-7}	C_{-6}	T_{-5}	A_{-4}	G_{-3}	C_{-2}	C_{-1}	C_{1}	T_{2}	T_{3}	C_{4}	T_{5}	A_{6}	T_{7}	C_{8}	T_{9}	C_{10}	A_{11}	G_{12}	A_{13}	A_{14}	C_{15}	C_{16}	A_{17}	T_{18}	C_{19}	C_{20}	A_{21}	G_{22}	G_{23}	Indel
A	99.9	99.9	0	0	0	0	99.9	0.1	0	0	0	0	0	0	0	100	0	0	0.1	0	99.9	0	99.8	99.9	0	0	99.8	0	0	0.1	99.9	0	0	0
C	0	0	100	0.1	100	0.1	0	0	99.9	100	100	0.1	0.1	100	0.1	0	0.1	100	0.2	99.9	0	0	0	0	99.9	99.9	0.1	0.1	99.9	99.9	0	0	0	
G	0.1	0.1	0	0	0	0	0.1	99.9	0	0	0	0	0	0	0	0	0.1	0	0.1	0	0.1	99.9	0.1	0.1	0	0	0.1	0.1	0	0	0.1	99.9	99.9	
T	0	0	0	99.8	0	99.9	0	0	0	0	0	99.9	99.8	0	99.9	0	99.8	0	99.6	0	0	0.1	0	0	0	0	0	99.8	0	0	0	0	0	

325. A PDCD1 Ex.1 SD OT4 + BE4 mRNA

	G_{-10}	A_{-9}	A_{-8}	G_{-7}	T_{-6}	G_{-5}	C_{-4}	C_{-3}	C_{-2}	A_{-1}	T_{1}	A_{2}	C_{3}	C_{4}	C_{5}	A_{6}	G_{7}	C_{8}	T_{9}	C_{10}	A_{11}	G_{12}	A_{13}	A_{14}	C_{15}	C_{16}	A_{17}	T_{18}	C_{19}	C_{20}	T_{21}	G_{22}	G_{23}	Indel
A	0	99.8	99.8	0	0	0.1	0	0	0	99.9	0	99.9	0.1	0	0.1	99.8	0.1	0	0	0	99.9	0	99.8	99.8	0.1	0.1	99.9	0.1	0.1	0	0	0	0	0
C	0	0	0	0	0	0	99.9	99.9	99.9	0	0	0	99.9	99.9	99.9	0	0	99.9	0.2	99.9	0	0	0	0.1	99.9	99.9	0	0.1	99.9	99.9	0.1	0	0	
G	99.9	0.1	0.2	99.9	0	99.7	0	0	0	0.1	0	0.1	0	0	0	0.1	99.9	0	0.1	0	0.1	99.9	0.1	0.1	0	0	0.1	0	0	0	0	99.9	99.8	
T	0.1	0	0	0	99.9	0.2	0.1	0	0	0	99.9	0	0	0	0	0	0	0	99.7	0	0	0.1	0	0	0	0	0	99.8	0	0	99.8	0.1	0.1	

326. A PDCD1 Ex.1 SD OT5 + BE4 mRNA

	G_{-10}	T_{-9}	T_{-8}	C_{-7}	C_{-6}	C_{-5}	A_{-4}	T_{-3}	C_{-2}	A_{-1}	C_{1}	A_{2}	C_{3}	T_{4}	C_{5}	A_{6}	C_{7}	T_{8}	T_{9}	A_{10}	A_{11}	G_{12}	T_{13}	A_{14}	C_{15}	C_{16}	A_{17}	T_{18}	C_{19}	C_{20}	T_{21}	G_{22}	G_{23}	Indel
A	0.1	0	0	0	0	0	99.9	0	0	99.8	0	99.9	0	0	0	99.9	0	0	0	99.9	99.8	0	0	99.9	0	0.1	99.9	0	0	0.1	0	0	0	0
C	0	0.1	0.1	99.9	99.9	99.9	0	0.2	99.9	0	99.9	0	100	0.2	99.9	0	100	0.1	0	0	0	0	0.1	0	99.9	99.9	0	0.1	99.9	99.9	0.1	0	0	
G	99.8	0	0	0	0	0	0.1	0	0	0.1	0	0.1	0	0	0	0.1	0	0	0	0	0.1	99.9	0	0	0	0	0.1	0	0	0	0	99.9	99.9	
T	0.1	99.8	99.8	0	0	0	0	99.8	0	0	0	0	0	99.8	0	0	0	99.9	100	0	0	0	99.9	0	0	0	0	99.9	0	0	99.9	0.1	0.1	

327. A PDCD1 Ex.1 SD OT6 + BE4 mRNA

	C_{-10}	T_{-9}	T_{-8}	G_{-7}	G_{-6}	A_{-5}	A_{-4}	A_{-3}	A_{-2}	A_{-1}	C_{1}	A_{2}	C_{3}	A_{4}	A_{5}	A_{6}	C_{7}	C_{8}	T_{9}	G_{10}	A_{11}	G_{12}	A_{13}	A_{14}	C_{15}	C_{16}	A_{17}	T_{18}	C_{19}	G_{20}	A_{21}	G_{22}	G_{23}	Indel
A	0	0	0	0	0	99.8	99.8	99.8	99.8	99.7	0	99.8	0	99.9	99.8	99.8	0	0	0	0	99.6	0	99.7	99.8	0	0	99.9	0	0	0.1	99.8	0	0	0
C	99.9	0.1	0.1	0	0	0	0	0	0	0.1	99.9	0	99.9	0	0	0	99.9	100	0.2	0	0	0	0	0	0	99.9	0	0	99.9	0	0	0	0	
G	0	0	0	99.9	100	0.2	0.2	0.1	0.1	0.2	0	0.2	0	0.1	0.1	0.1	0	0	0.1	99.9	0.3	99.9	0.2	0.2	99.9	0	0.1	0	0	99.9	0.2	99.9	100	
T	0	99.8	99.8	0	0	0	0	0	0	0	0	0	0	0	0	0	0	0	99.8	0	0	0	0	0	0	0	0	99.9	0.1	0	0	0	0	

328. A PDCD1 Ex.1 SD OT7 + BE4 mRNA

	T_{-10}	C_{-9}	C_{-8}	A_{-7}	T_{-6}	C_{-5}	T_{-4}	C_{-3}	A_{-2}	C_{-1}	C_{1}	A_{2}	C_{3}	C_{4}	T_{5}	C_{6}	C_{7}	A_{8}	T_{9}	T_{10}	T_{11}	G_{12}	A_{13}	A_{14}	C_{15}	C_{16}	A_{17}	T_{18}	C_{19}	C_{20}	T_{21}	G_{22}	G_{23}	Indel
A	0	0.1	0.1	99.8	0	0.1	0	0.2	99.8	0.1	0.2	99.7	0	0.1	0.1	0	0	99.9	0	0	0	0	99.8	99.8	0	0.1	99.8	0	0	0.1	0	0	0.1	0
C	0.1	99.9	99.9	0.1	0.3	99.8	0.2	99.8	0.2	99.9	99.8	0.2	100	99.9	0.1	99.9	99.9	0.1	0.1	0	0	0	0.1	0.1	99.9	99.9	0.1	0.1	99.9	99.9	0.1	0	0.1	
G	0	0	0	0.1	0	0	0.1	0	0	0	0	0.1	0	0	0.1	0	0	0	0.1	0.1	0	99.7	0.1	0.1	0	0	0	0	0	0	0	99.8	99.6	
T	99.9	0	0	0	99.7	0	99.7	0	0	0	0	0	0	0	99.7	0.1	0	0	99.8	99.9	100	0.3	0	0	0	0	0.1	99.8	0	0	99.9	0.2	0.2	

329. A PDCD1 Ex.1 SD OT8 + BE4 mRNA

	A_{-10}	A_{-9}	A_{-8}	A_{-7}	A_{-6}	G_{-5}	C_{-4}	A_{-3}	A_{-2}	C_{-1}	C_{1}	A_{2}	C_{3}	C_{4}	C_{5}	A_{6}	C_{7}	C_{8}	T_{9}	A_{10}	A_{11}	G_{12}	C_{13}	A_{14}	C_{15}	C_{16}	A_{17}	T_{18}	C_{19}	T_{20}	A_{21}	G_{22}	G_{23}	Indel
A	99.9	100	99.9	99.9	99.8	0	0.1	99.9	99.8	0	0.2	99.9	0	0.1	0.2	99.9	0	0	0	99.9	99.9	0	0.1	99.8	0	0	99.9	0	0	0	99.9	0	0	0
C	0	0	0.1	0	0	0	99.9	0	0	99.9	99.8	0	99.9	99.8	99.7	0	99.9	99.9	0	0	0	0	99.9	0.1	99.9	99.9	0	0.1	100	0	0	0	0	
G	0	0	0	0	0.2	99.9	0	0.1	0.2	0	0	0.1	0	0	0	0.1	0	0	0	0	0.1	99.9	0	0.1	0	0	0	0.1	0	0	0.1	99.9	99.9	
T	0	0	0	0	0	0	0.1	0	0	0	0	0	0	0	0	0	0	0	99.9	0	0	0	0	0	0	0	0	99.8	0	99.9	0	0.1	0.1	

330. A PDCD1 Ex.1 SD OT9 + BE4 mRNA

	G_{-10}	A_{-9}	T_{-8}	G_{-7}	G_{-6}	A_{-5}	T_{-4}	C_{-3}	A_{-2}	C_{-1}	C_{1}	A_{2}	C_{3}	C_{4}	T_{5}	T_{6}	C_{7}	A_{8}	T_{9}	C_{10}	A_{11}	G_{12}	A_{13}	A_{14}	C_{15}	C_{16}	A_{17}	T_{18}	C_{19}	T_{20}	G_{21}	G_{22}	G_{23}	Indel
A	0	99.9	0	0	0	99.7	0	0	99.8	0	0	99.8	0	0	0	0	0	99.9	0	0	99.8	0.1	99.9	99.9	0	0	99.9	0	0	0	0	0	0.1	0
C	0	0	0	0	0	0	0	99.9	0	99.9	99.9	0	100	100	0.1	0.1	99.9	0	0.1	100	0	0	0	0	100	100	0	0.1	99.9	0	0	0	0	
G	100	0.1	0.1	99.8	99.9	0.1	0.1	0	0.1	0	0	0.1	0	0	0.1	0.1	0	0	0	0	0.1	99.9	0.1	0.1	0	0	0	0.1	0	0	99.9	99.9	99.8	
T	0	0	99.9	0.1	0.1	0.1	99.8	0	0	0	0	0	0	0	99.8	99.8	0	0	99.9	0	0	0	0	0	0	0	0	99.9	0	99.9	0.1	0	0.1	

331. A PDCD1 Ex.1 SD OT10 + BE4 mRNA

	G_{-10}	T_{-9}	T_{-8}	C_{-7}	C_{-6}	A_{-5}	G_{-4}	A_{-3}	A_{-2}	C_{-1}	C_{1}	A_{2}	G_{3}	C_{4}	T_{5}	A_{6}	T_{7}	C_{8}	T_{9}	C_{10}	A_{11}	G_{12}	A_{13}	A_{14}	C_{15}	C_{16}	T_{17}	T_{18}	C_{19}	C_{20}	T_{21}	G_{22}	G_{23}	Indel
A	0	0	0	0	0	99.8	0	99.8	99.8	0	0	99.9	0	0	0	99.9	0	0	0	0	99.9	0	99.9	99.9	0	0	0	0	0	0	0	0	0	0
C	0	0.1	0	100	99.9	0	0	0	0	99.9	99.9	0	0	100	0.1	0	0.2	100	0.1	100	0	0	0	0	99.9	99.6	0.1	0.1	99.9	100	0.2	0	0	
G	100	0	0	0	0	0.1	100	0.1	0.2	0	0	0.1	99.9	0	0	0	0	0	0	0	0.1	99.9	0.1	0.1	0	0	0	0	0	0	0	99.9	99.9	
T	0	99.8	99.9	0	0	0	0	0	0	0	0	0	0	0	99.8	0	99.8	0	99.8	0	0	0	0	0	0	0.4	99.9	99.9	0	0	99.8	0	0	
