# Supplementary material for: Highly efficient multiplex human T cell engineering without double-strand breaks using Cas9 base editors
Source: Nat Commun. 2019 Nov 19;10:5222. doi: 10.1038/s41467-019-13007-6 (PMC6864045; doi:10.1038/s41467-019-13007-6)

# Highly efficient multiplex human T cell engineering without double-strand breaks using Cas9 base editors

## Appendix III - Indel Distribution Plots of Next Generation Sequencing

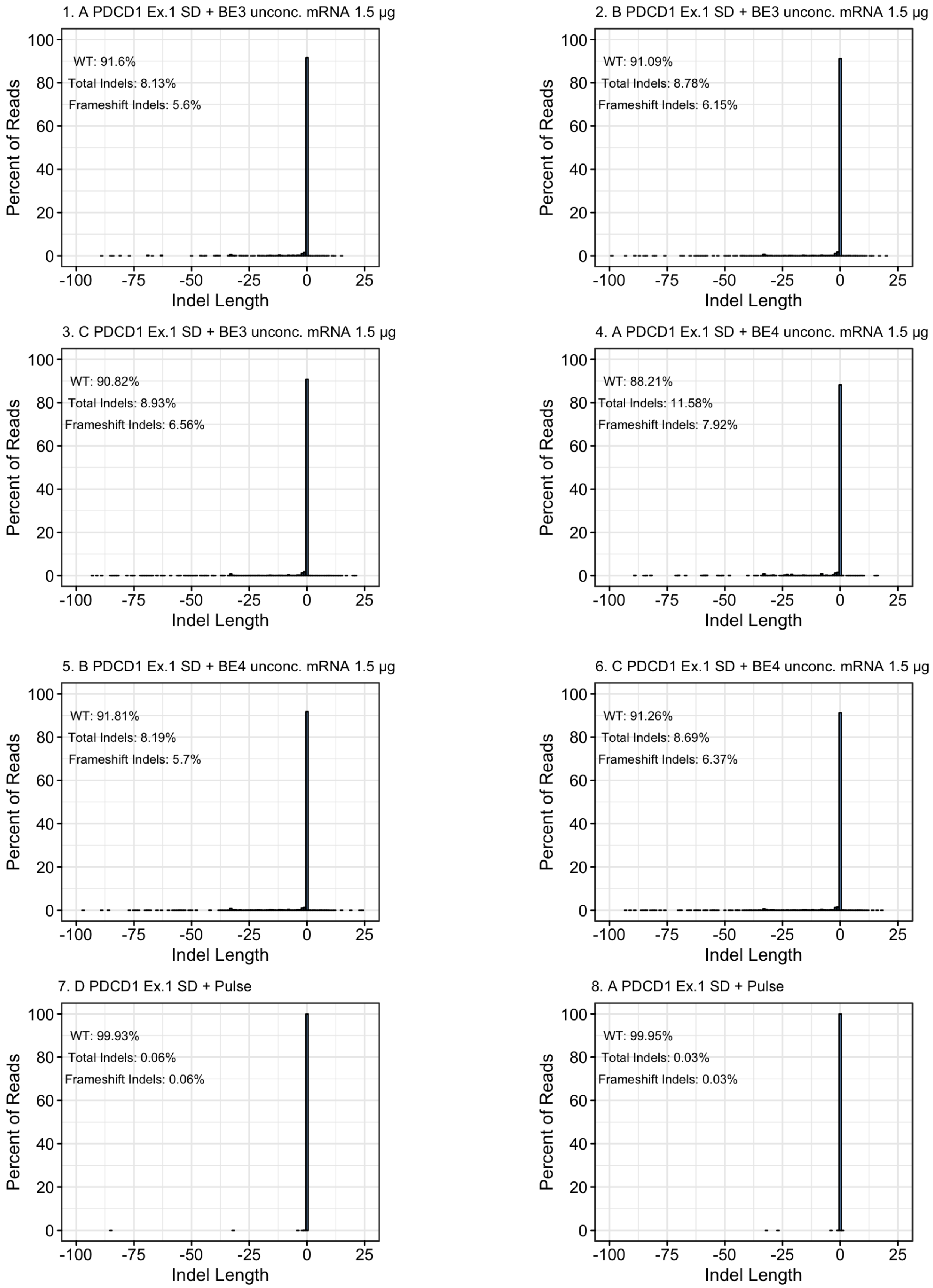
1. A PDCD1 Ex.1 SD + BE3 unconc. mRNA 1.5 µg
WT: 91.6%
Total Indels: 8.13%
Frameshift Indels: 5.6%
2. B PDCD1 Ex.1 SD + BE3 unconc. mRNA 1.5 µg
WT: 91.09%
Total Indels: 8.78%
Frameshift Indels: 6.15%
3. C PDCD1 Ex.1 SD + BE3 unconc. mRNA 1.5 µg
WT: 90.82%
Total Indels: 8.93%
Frameshift Indels: 6.56%
4. A PDCD1 Ex.1 SD + BE4 unconc. mRNA 1.5 µg
WT: 88.21%
Total Indels: 11.58%
Frameshift Indels: 7.92%
5. B PDCD1 Ex.1 SD + BE4 unconc. mRNA 1.5 µg
WT: 91.81%
Total Indels: 8.19%
Frameshift Indels: 5.7%
6. C PDCD1 Ex.1 SD + BE4 unconc. mRNA 1.5 µg
WT: 91.26%
Total Indels: 8.69%
Frameshift Indels: 6.37%
7. D PDCD1 Ex.1 SD + Pulse
WT: 99.93%
Total Indels: 0.06%
Frameshift Indels: 0.06%
8. A PDCD1 Ex.1 SD + Pulse
WT: 99.95%
Total Indels: 0.03%
Frameshift Indels: 0.03%
Percent of Reads
Indel Length
0
20
40
60
80
100
-100
-75
-50
-25
0
25

9. A PDCD1 Ex.2 SA + BE3 unconc. mRNA 1.5 µg

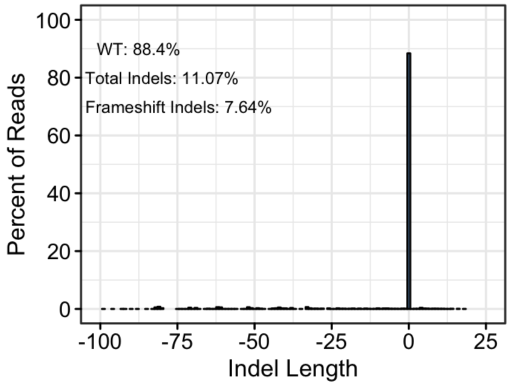


10. B PDCD1 Ex.2 SA + BE3 unconc. mRNA 1.5 µg

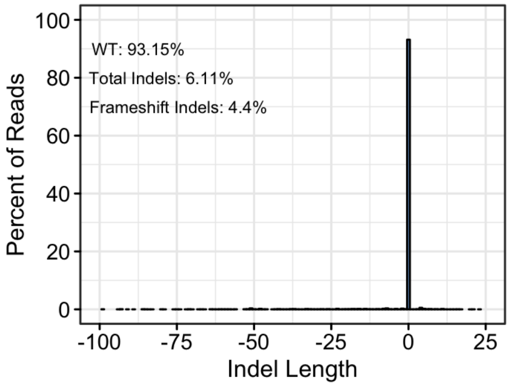


11. C PDCD1 Ex.2 SA + BE3 unconc. mRNA 1.5 µg

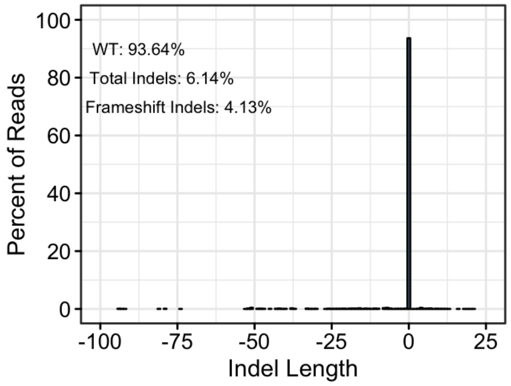


12. A PDCD1 Ex.2 SA + BE4 unconc. mRNA 1.5 µg

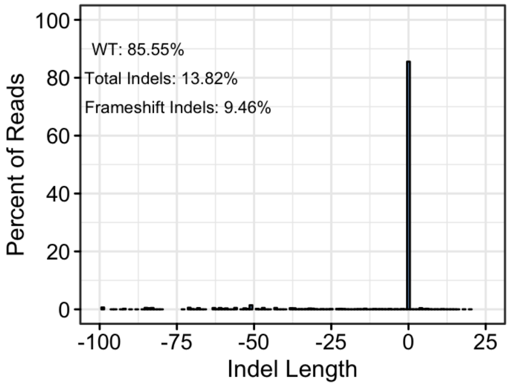


13. B PDCD1 Ex.2 SA + BE4 unconc. mRNA 1.5 µg

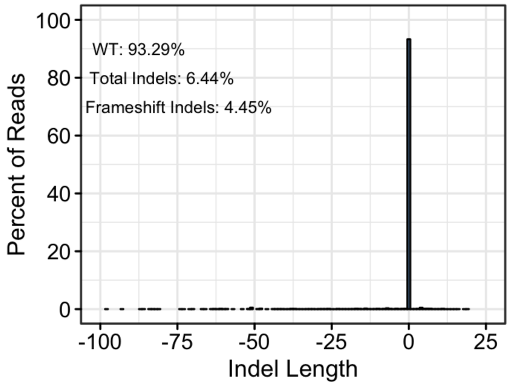


14. C PDCD1 Ex.2 SA + BE4 unconc. mRNA 1.5 µg

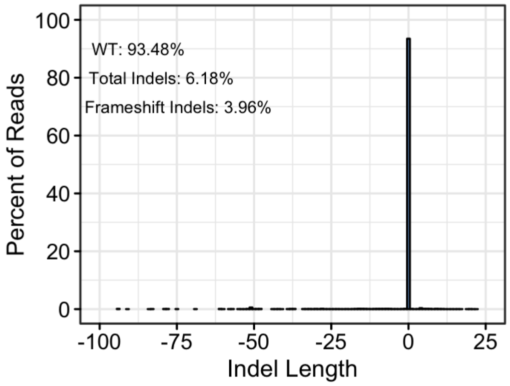


15. D PDCD1 Ex.2 SA + Pulse

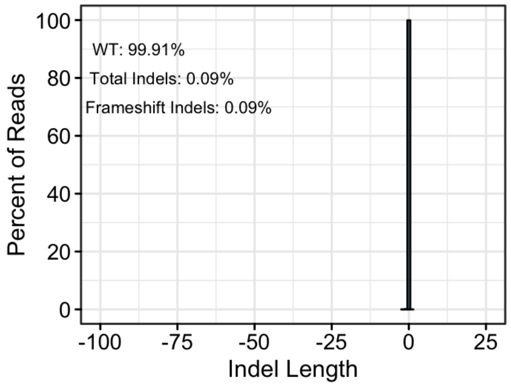


16. A PDCD1 Ex.2 SA + Pulse

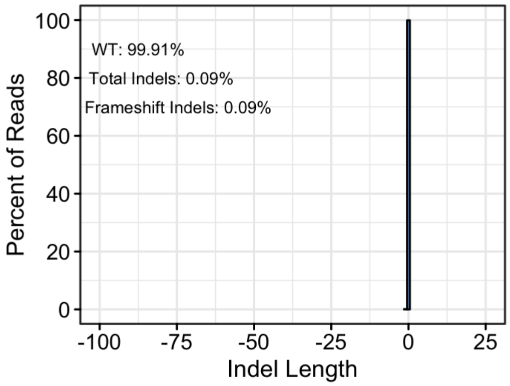

17. A PDCD1 Ex.3 SA + BE3 unconc. mRNA 1.5 µg

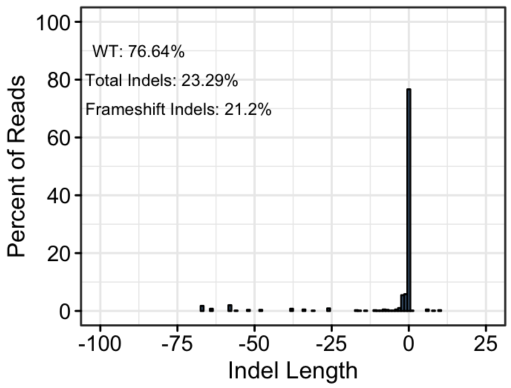


18. B PDCD1 Ex.3 SA + BE3 unconc. mRNA 1.5 µg

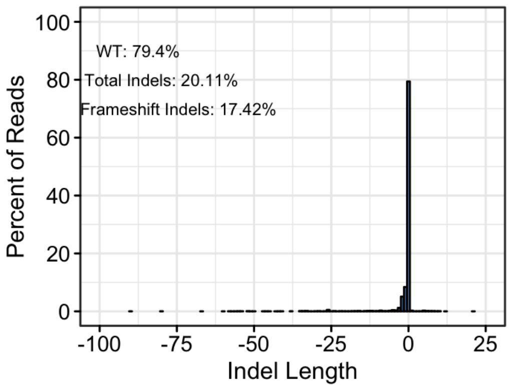


19. C PDCD1 Ex.3 SA + BE3 unconc. mRNA 1.5 µg

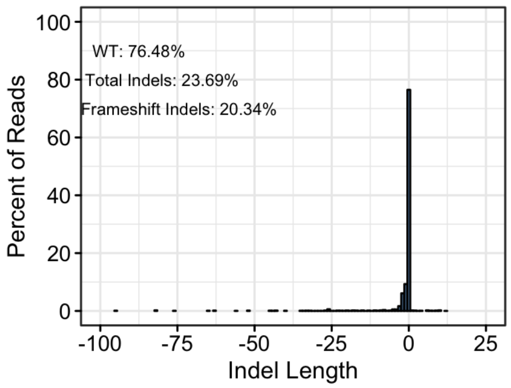


20. A PDCD1 Ex.3 SA + BE4 unconc. mRNA 1.5 µg

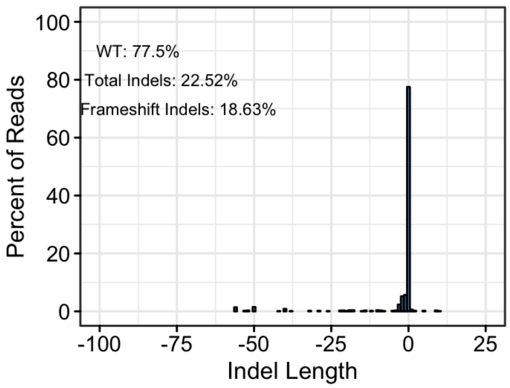


21. B PDCD1 Ex.3 SA + BE4 unconc. mRNA 1.5 µg

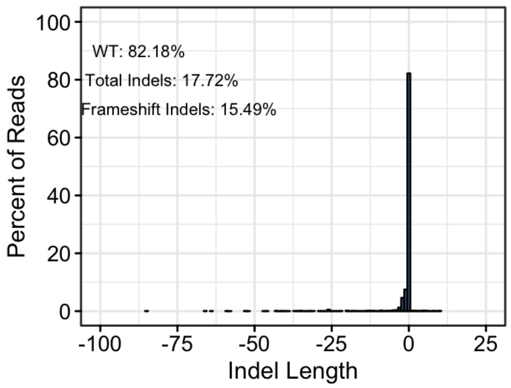


22. C PDCD1 Ex.3 SA + BE4 unconc. mRNA 1.5 µg

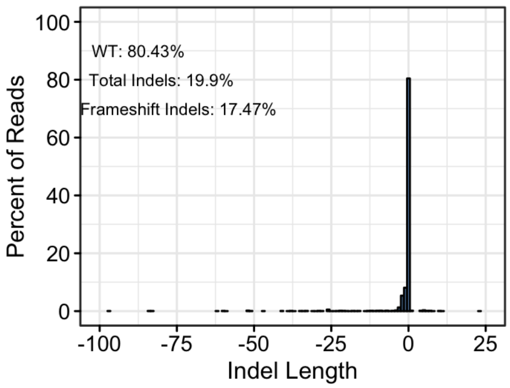


23. D PDCD1 Ex.3 SA + Pulse

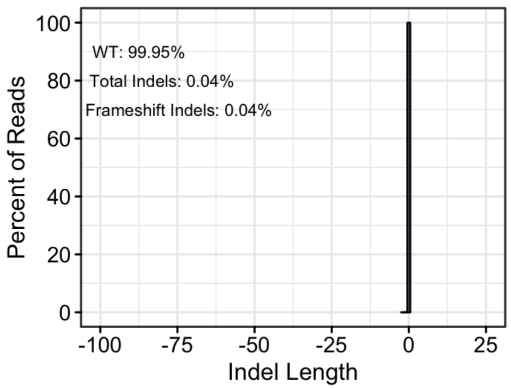


24. A PDCD1 Ex.3 SA + Pulse

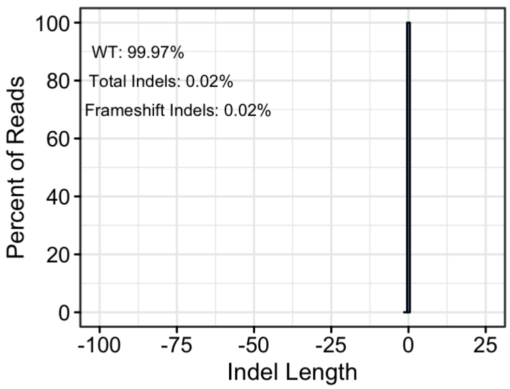

25. A PDCD1 Ex.3 SD + BE3 unconc. mRNA 1.5 μg

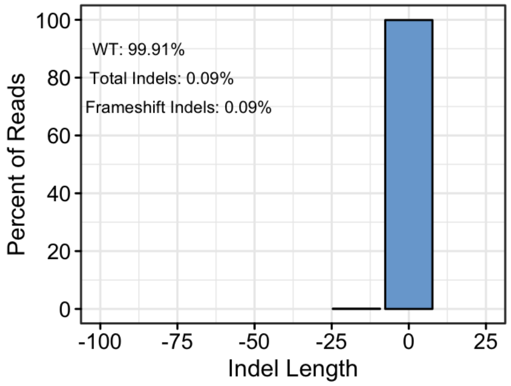


26. B PDCD1 Ex.3 SD + BE3 unconc. mRNA 1.5 μg

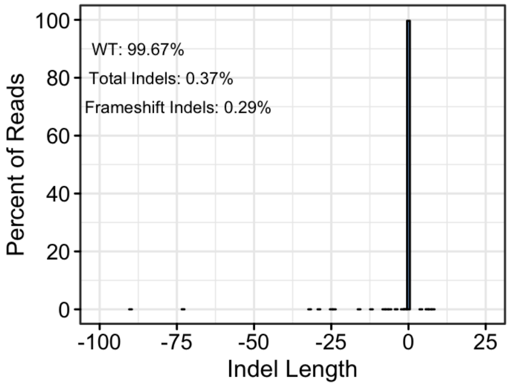


27. C PDCD1 Ex.3 SD + BE3 unconc. mRNA 1.5 μg

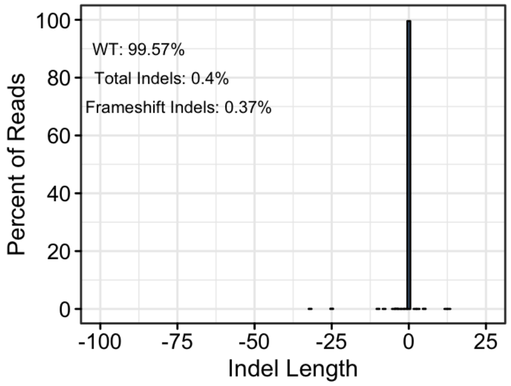


28. A PDCD1 Ex.3 SD + BE4 unconc. mRNA 1.5 μg

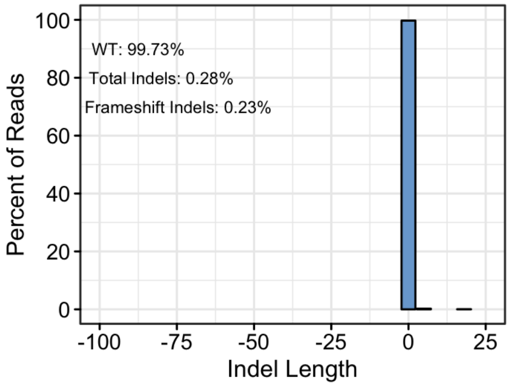


29. B PDCD1 Ex.3 SD + BE4 unconc. mRNA 1.5 μg

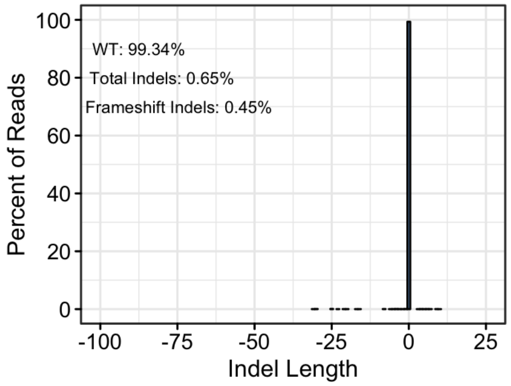


30. C PDCD1 Ex.3 SD + BE4 unconc. mRNA 1.5 μg

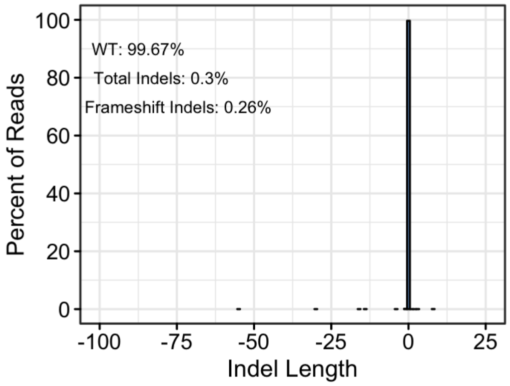


31. A PDCD1 Ex.4 SA + BE3 unconc. mRNA 1.5 μg

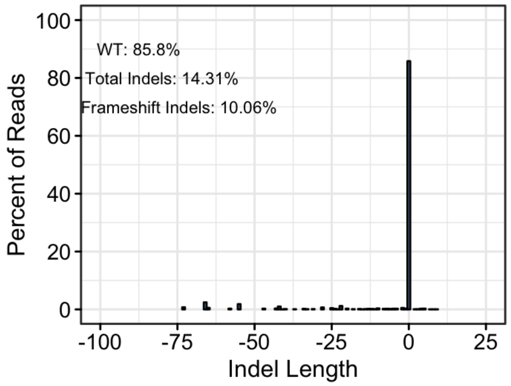


32. B PDCD1 Ex.4 SA + BE3 unconc. mRNA 1.5 μg

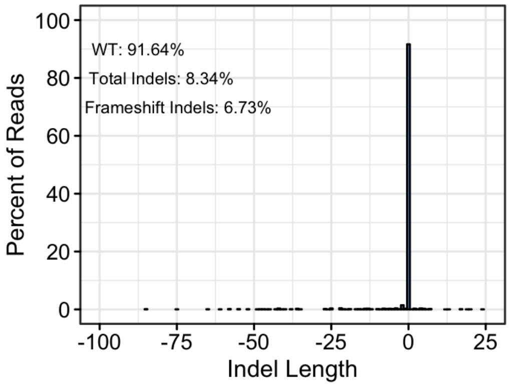

33. C PDCD1 Ex.4 SA + BE3 unconc. mRNA 1.5 µg

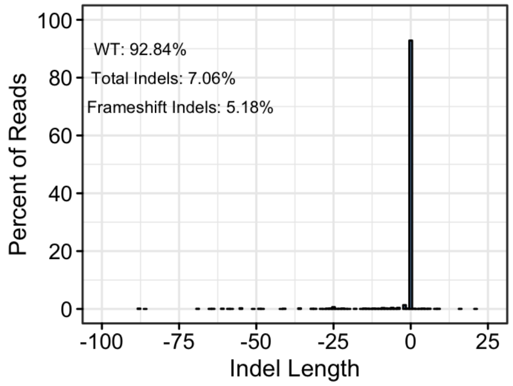


34. A PDCD1 Ex.4 SA + BE4 unconc. mRNA 1.5 µg

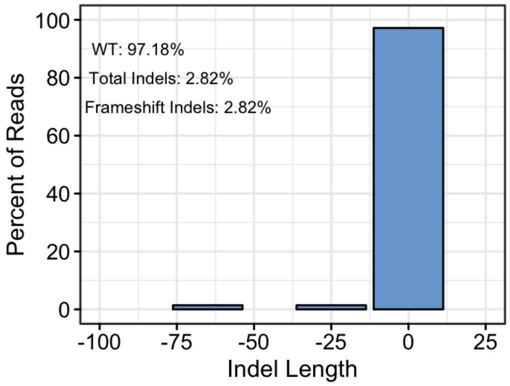


35. B PDCD1 Ex.4 SA + BE4 unconc. mRNA 1.5 µg

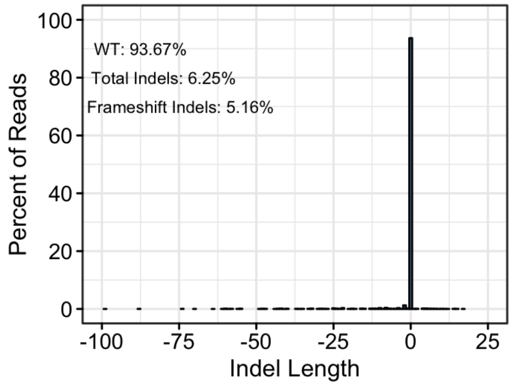


36. C PDCD1 Ex.4 SA + BE4 unconc. mRNA 1.5 µg

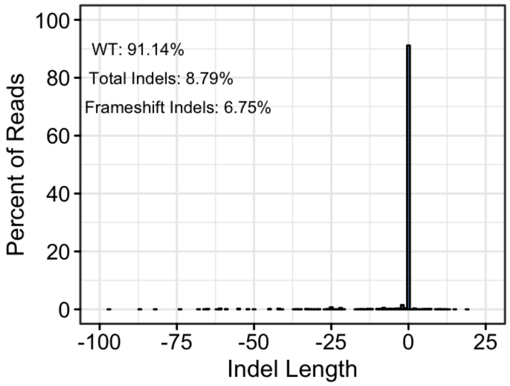


37. D PDCD1 Ex.4 SA + Pulse

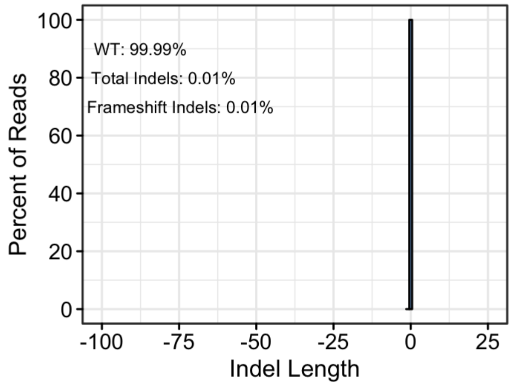


38. A PDCD1 Ex.4 SA + Pulse

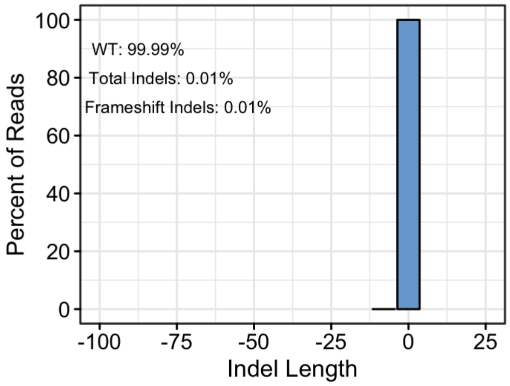


39. A PDCD1 Ex.2 pmSTOP + BE3 unconc. mRNA 1.5 µg

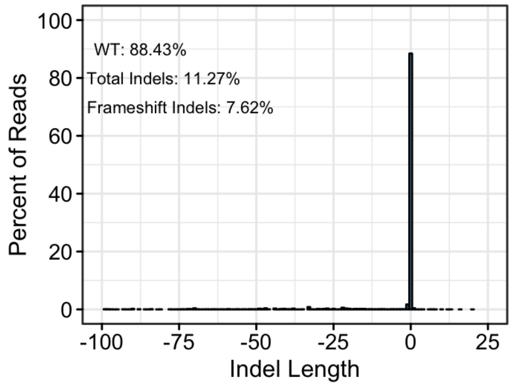


40. B PDCD1 Ex.2 pmSTOP + BE3 unconc. mRNA 1.5 µg

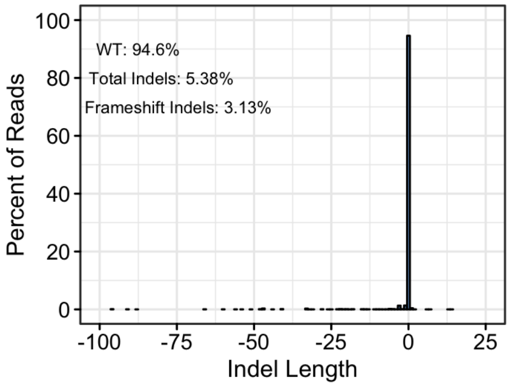

41. C PDCD1 Ex.2 pmSTOP + BE3 unconc. mRNA 1.5 µg

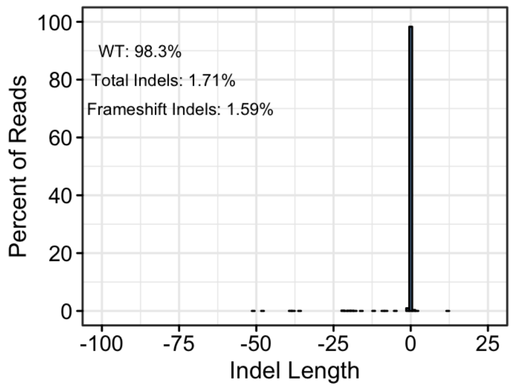


42. A PDCD1 Ex.2 pmSTOP + BE4 unconc. mRNA 1.5 µg

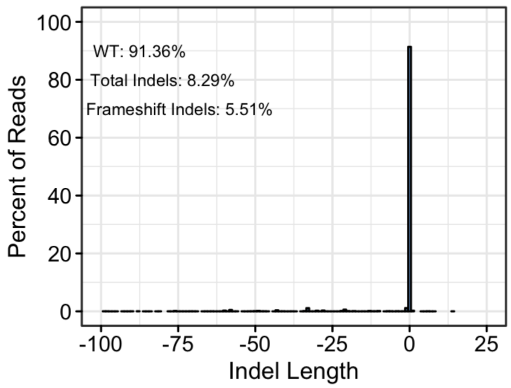


43. B PDCD1 Ex.2 pmSTOP + BE4 unconc. mRNA 1.5 µg

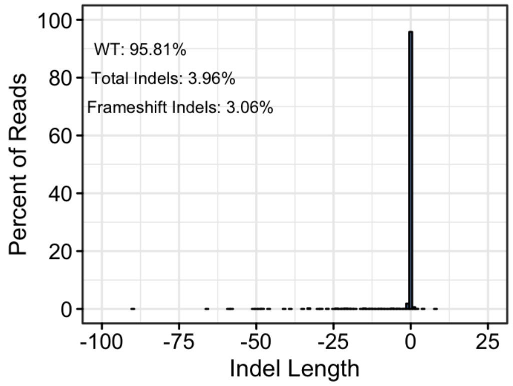


44. C PDCD1 Ex.2 pmSTOP + BE4 unconc. mRNA 1.5 µg

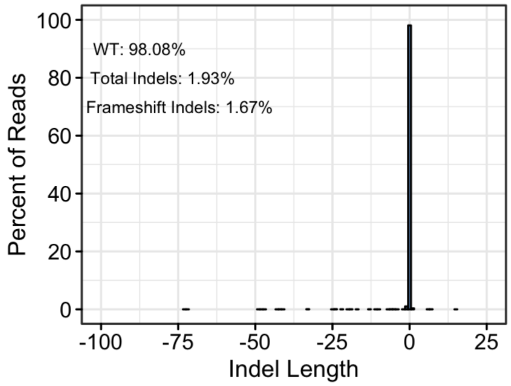


45. A PDCD1 Ex.2 pmSTOP + Cas9 mRNA 1.5 µg

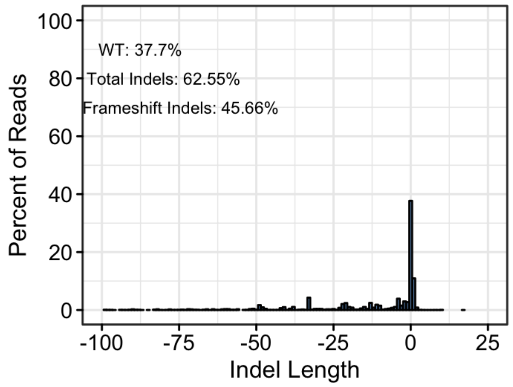


46. B PDCD1 Ex.2 pmSTOP + Cas9 mRNA 1.5 µg

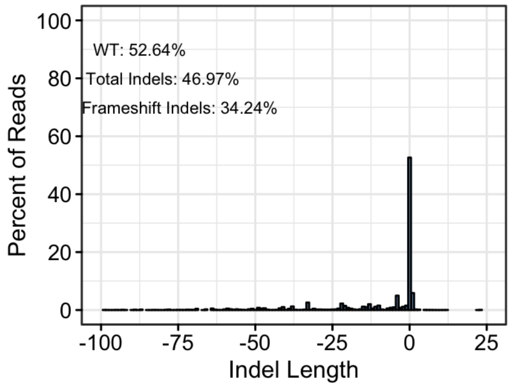


47. D PDCD1 Ex.2 pmSTOP + Pulse

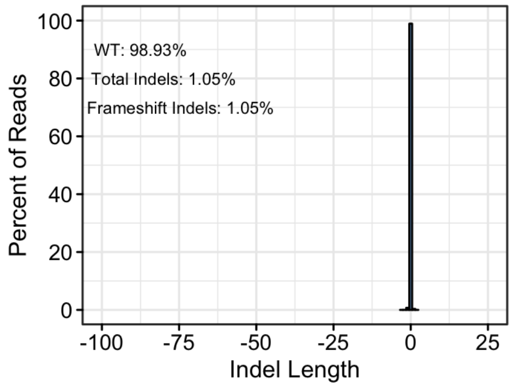


48. D PDCD1 Ex.2 pmSTOP + Pulse

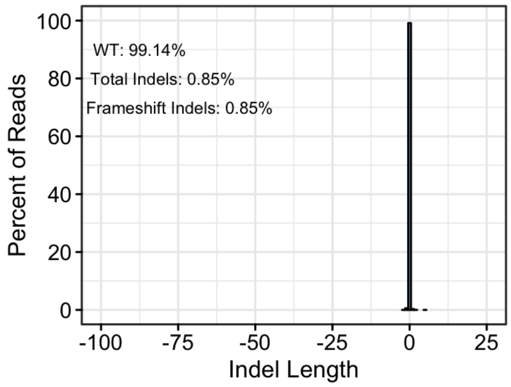

49. A PDCD1 Ex.2 pmSTOP + Pulse

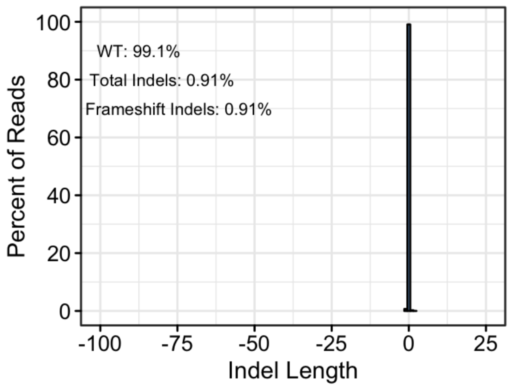


50. A PDCD1 Ex.2 pmSTOP + Pulse

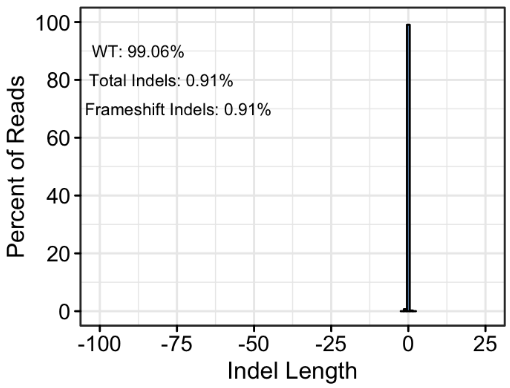


51. A PDCD1 Ex.3 pmSTOP_1 + BE3 unconc. mRNA 1.5 µg

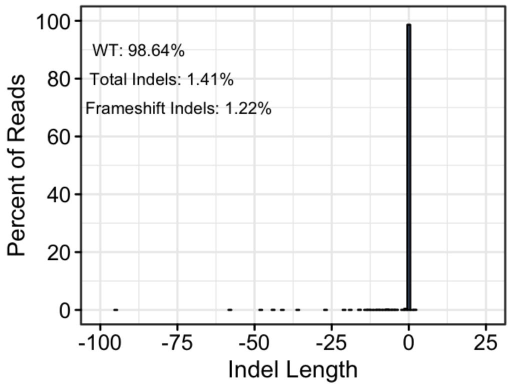


52. B PDCD1 Ex.3 pmSTOP_1 + BE3 unconc. mRNA 1.5 µg

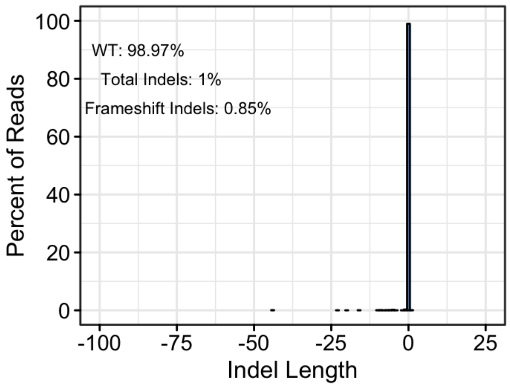


54. D PDCD1 Ex.3 pmSTOP_1 + BE3 unconc. mRNA 1.5 µg

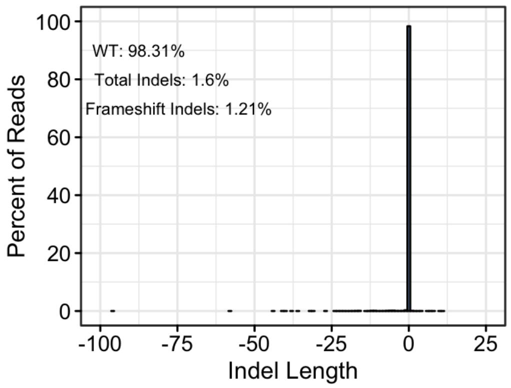


55. A PDCD1 Ex.3 pmSTOP_1 + BE4 unconc. mRNA 1.5 µg

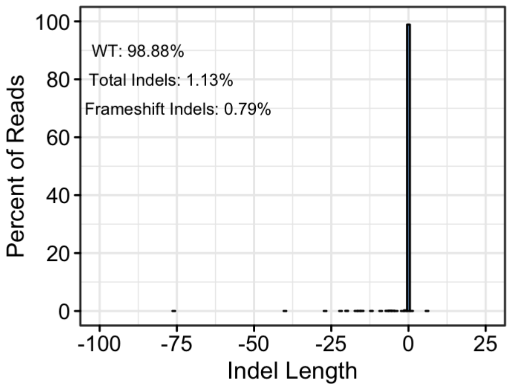


56. B PDCD1 Ex.3 pmSTOP_1 + BE4 unconc. mRNA 1.5 µg

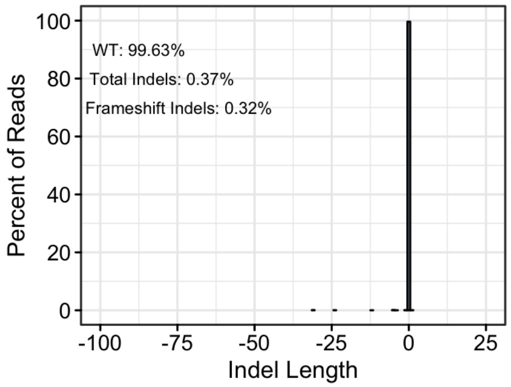


58. D PDCD1 Ex.3 pmSTOP_1 + BE4 unconc. mRNA 1.5 µg

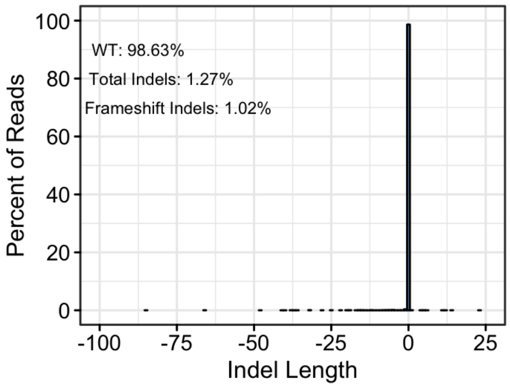

59. D PDCD1 Ex.3 pmSTOP_1 + Pulse

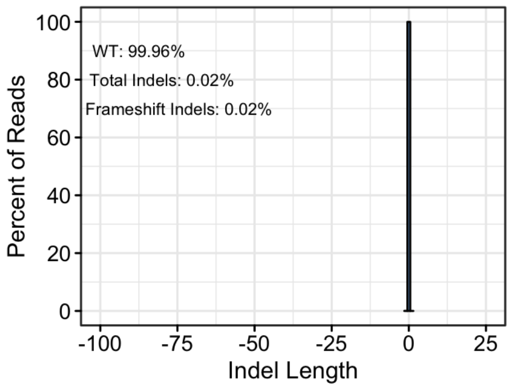


60. A PDCD1 Ex.3 pmSTOP_1 + Pulse

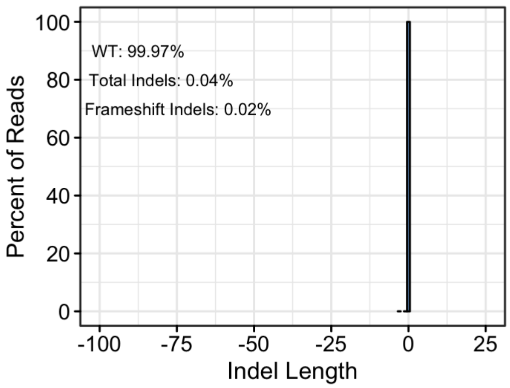


61. A PDCD1 Ex.3 pmSTOP_2 + BE3 unconc. mRNA 1.5 µg

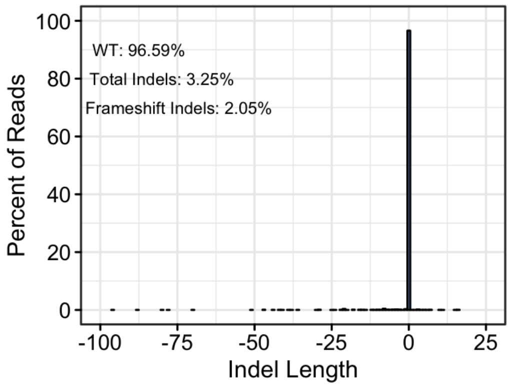


62. B PDCD1 Ex.3 pmSTOP_2 + BE3 unconc. mRNA 1.5 µ

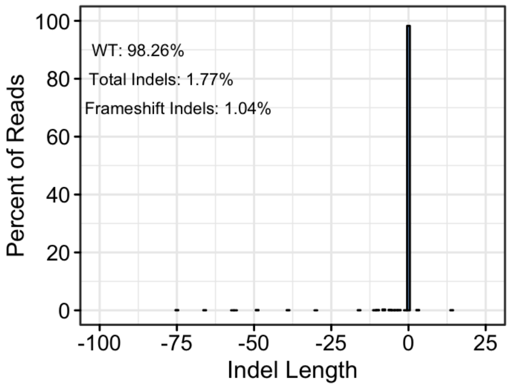


63. C PDCD1 Ex.3 pmSTOP_2 + BE3 unconc. mRNA 1.5 µg

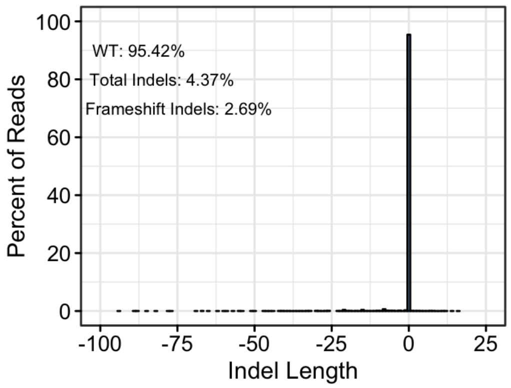


64. A PDCD1 Ex.3 pmSTOP_2 + BE4 unconc. mRNA 1.5 µ

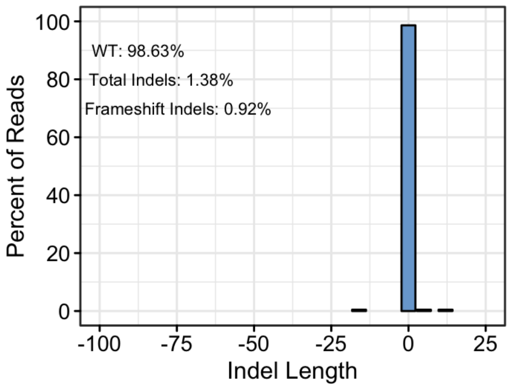


65. B PDCD1 Ex.3 pmSTOP_2 + BE4 unconc. mRNA 1.5 µg

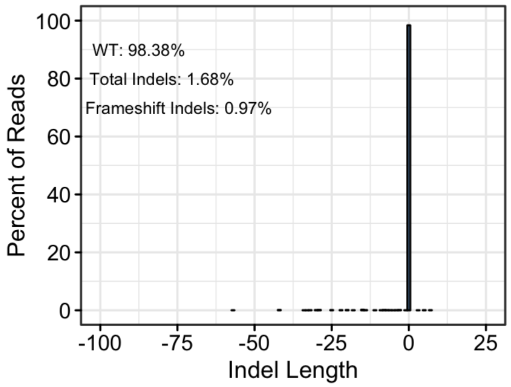


66. C PDCD1 Ex.3 pmSTOP_2 + BE4 unconc. mRNA 1.5 µ

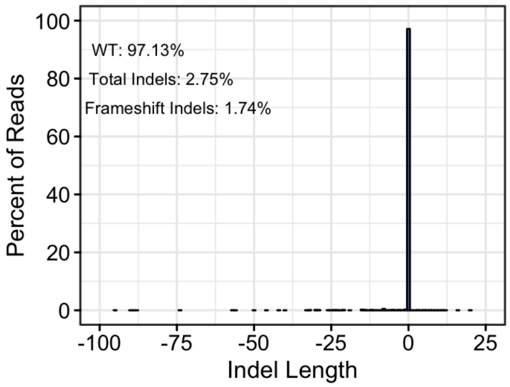

67. D PDCD1 Ex.3 pmSTOP_2 + Pulse

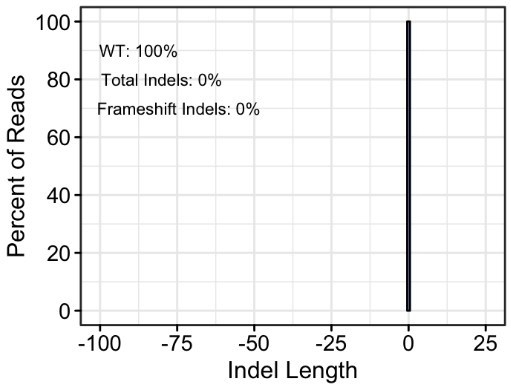


68. A PDCD1 Ex.3 pmSTOP_2 + Pulse

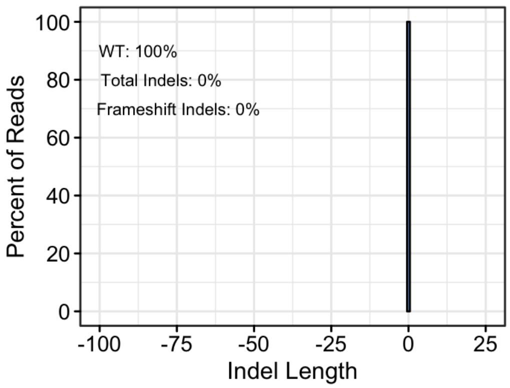


69. A TRAC Ex.1 SD + BE3 unconc. mRNA 1.5 µg

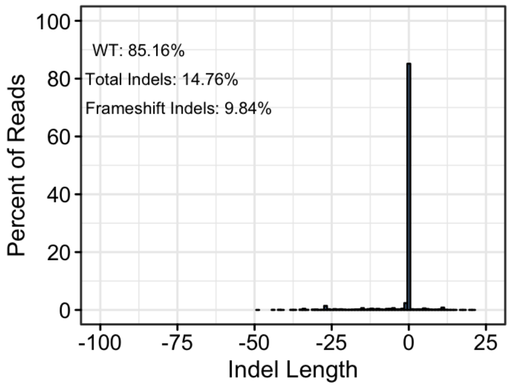


70. B TRAC Ex.1 SD + BE3 unconc. mRNA 1.5 µg

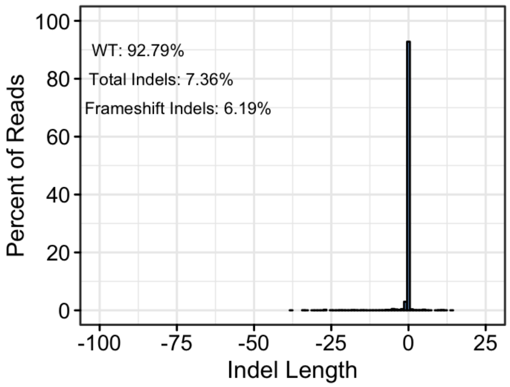


71. C TRAC Ex.1 SD + BE3 unconc. mRNA 1.5 µg

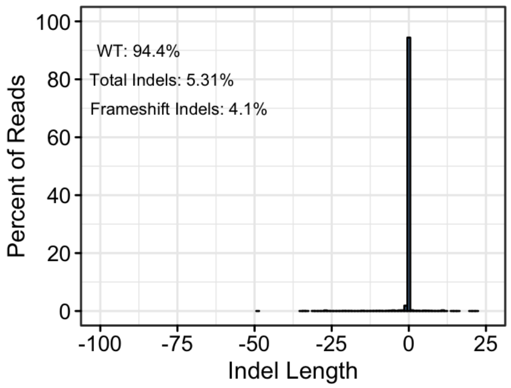


72. D TRAC Ex.1 SD + BE3 unconc. mRNA 1.5 µg

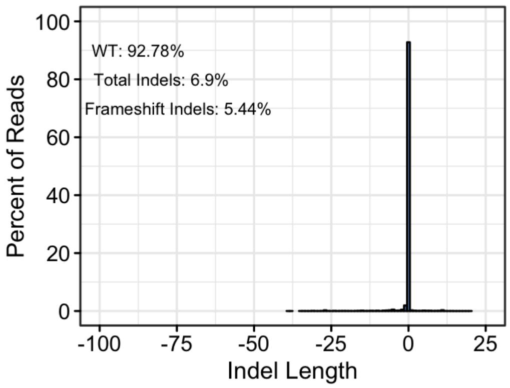


73. A TRAC Ex.1 SD + BE4 unconc. mRNA 1.5 µg

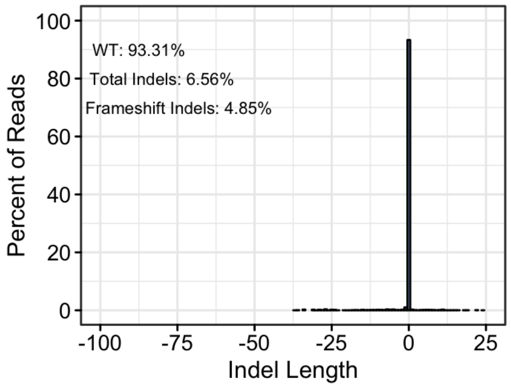


74. B TRAC Ex.1 SD + BE4 unconc. mRNA 1.5 µg

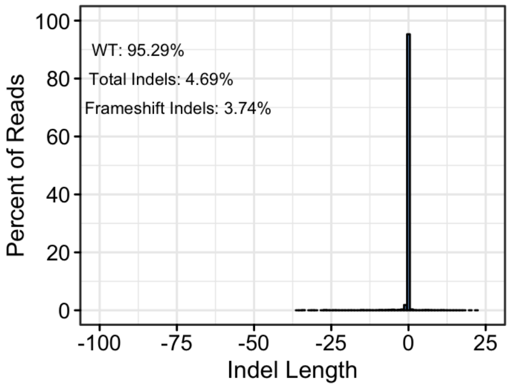

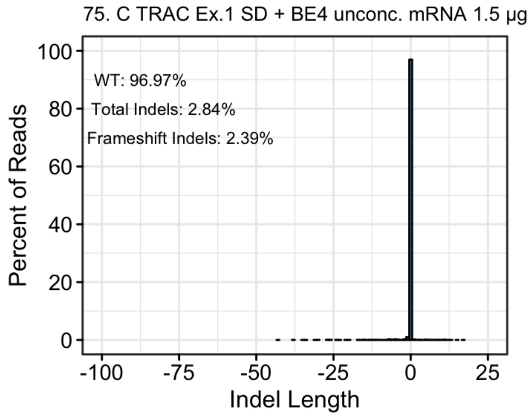
75. C TRAC Ex.1 SD + BE4 unconc. mRNA 1.5 µg
WT: 96.97%
Total Indels: 2.84%
Frameshift Indels: 2.39%
Percent of Reads
Indel Length

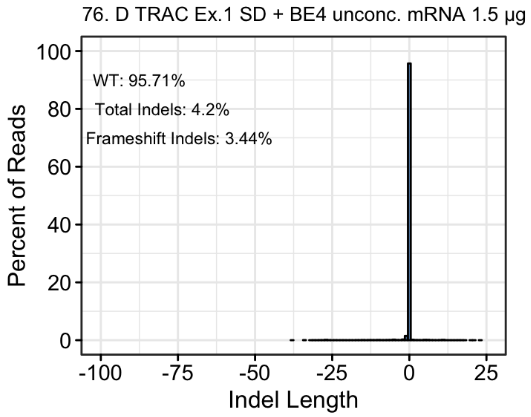
76. D TRAC Ex.1 SD + BE4 unconc. mRNA 1.5 µg
WT: 95.71%
Total Indels: 4.2%
Frameshift Indels: 3.44%
Percent of Reads
Indel Length

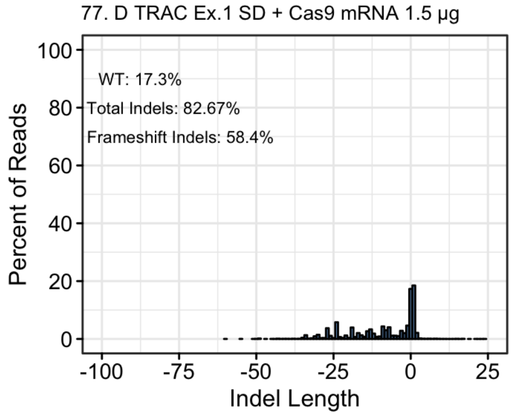
77. D TRAC Ex.1 SD + Cas9 mRNA 1.5 µg
WT: 17.3%
Total Indels: 82.67%
Frameshift Indels: 58.4%
Percent of Reads
Indel Length

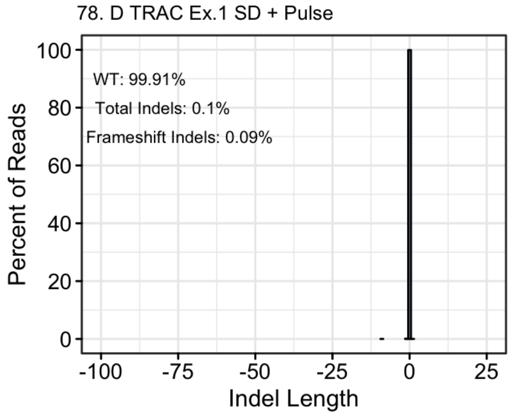
78. D TRAC Ex.1 SD + Pulse
WT: 99.91%
Total Indels: 0.1%
Frameshift Indels: 0.09%
Percent of Reads
Indel Length

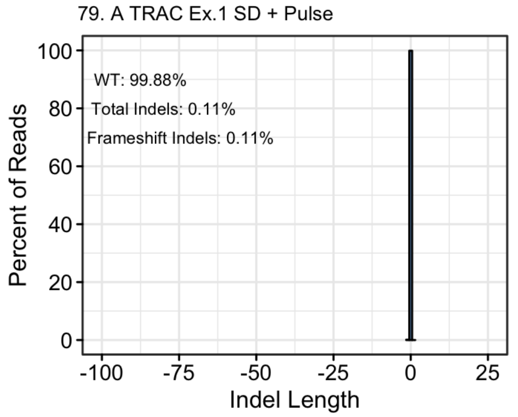
79. A TRAC Ex.1 SD + Pulse
WT: 99.88%
Total Indels: 0.11%
Frameshift Indels: 0.11%
Percent of Reads
Indel Length

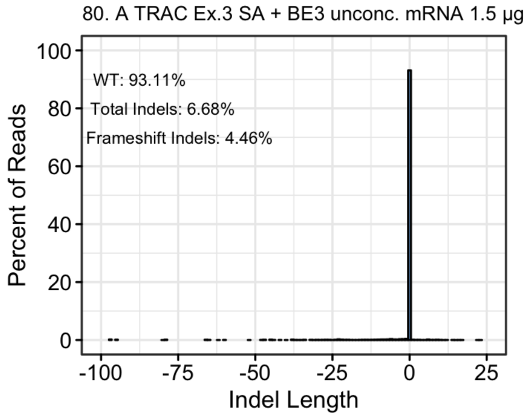
80. A TRAC Ex.3 SA + BE3 unconc. mRNA 1.5 µg
WT: 93.11%
Total Indels: 6.68%
Frameshift Indels: 4.46%
Percent of Reads
Indel Length

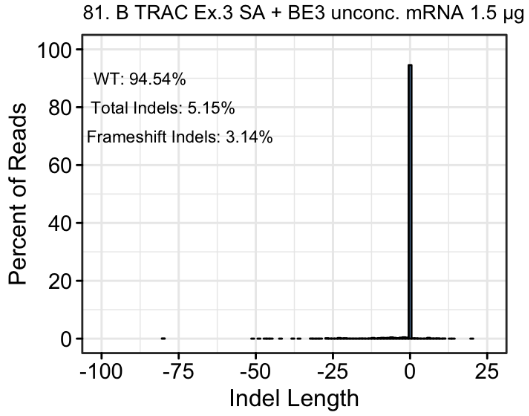
81. B TRAC Ex.3 SA + BE3 unconc. mRNA 1.5 µg
WT: 94.54%
Total Indels: 5.15%
Frameshift Indels: 3.14%
Percent of Reads
Indel Length

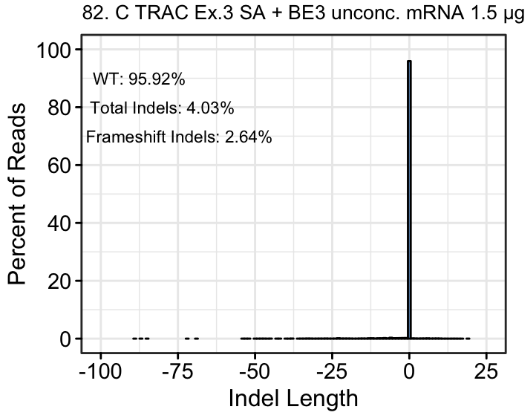
82. C TRAC Ex.3 SA + BE3 unconc. mRNA 1.5 µg
WT: 95.92%
Total Indels: 4.03%
Frameshift Indels: 2.64%
Percent of Reads
Indel Length

83. D TRAC Ex.3 SA + BE3 unconc. mRNA 1.5 µg

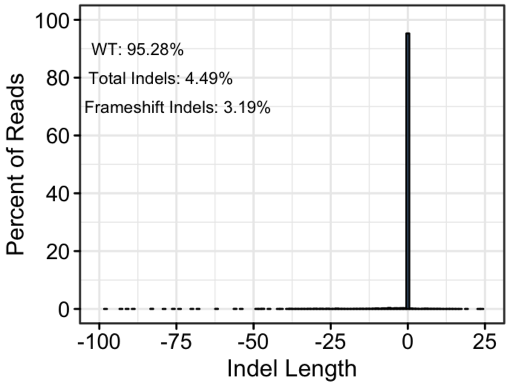


84. A TRAC Ex.3 SA + BE4 unconc. mRNA 1.5 µg

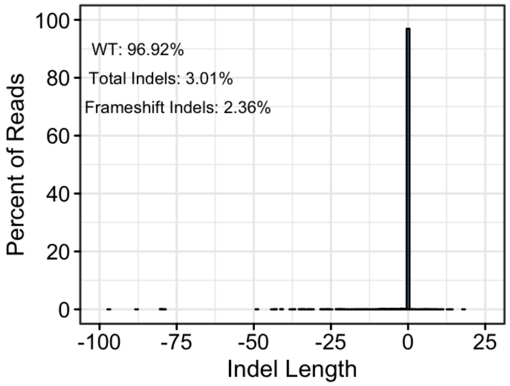


85. B TRAC Ex.3 SA + BE4 unconc. mRNA 1.5 µg

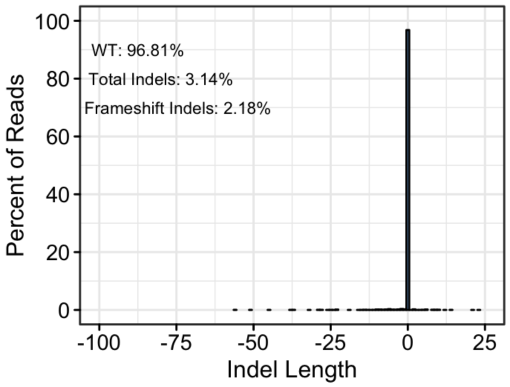


86. C TRAC Ex.3 SA + BE4 unconc. mRNA 1.5 µg

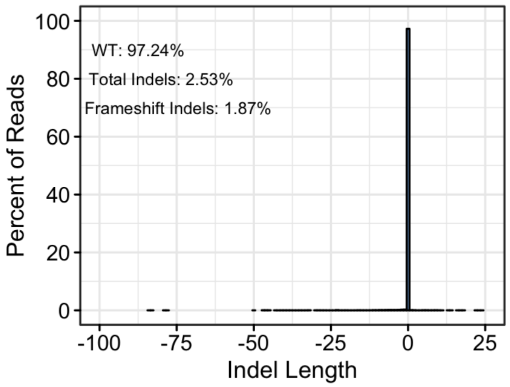


87. D TRAC Ex.3 SA + BE4 unconc. mRNA 1.5 µg

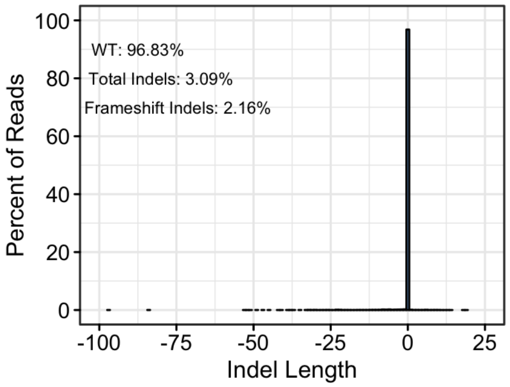


88. A TRAC Ex.3 SA + Cas9 mRNA 1.5 µg

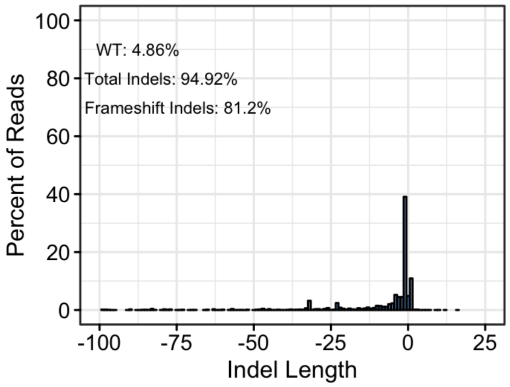


89. A TRAC Ex.3 SA + Pulse

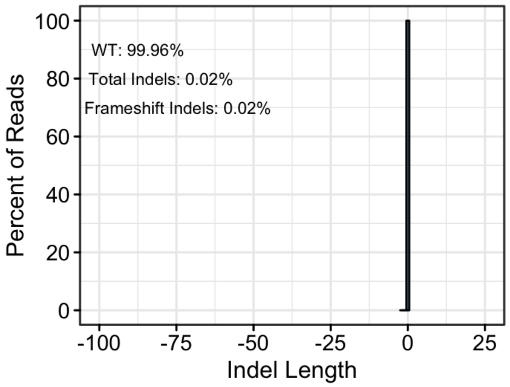


90. D TRAC Ex.3 SA + Pulse

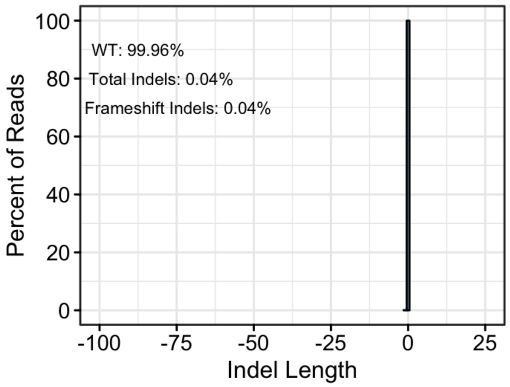

91. A TRAC Ex.3 pmSTOP_1 + BE3 unconc. mRNA 1.5 μg

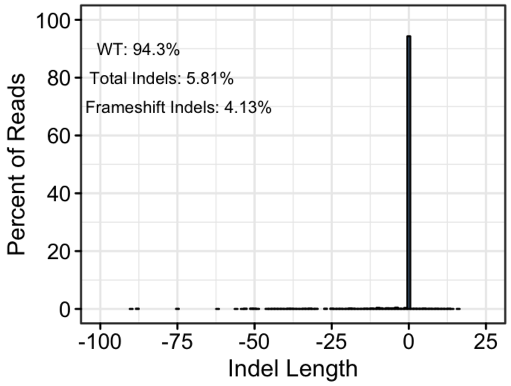


92. B TRAC Ex.3 pmSTOP_1 + BE3 unconc. mRNA 1.5 μg

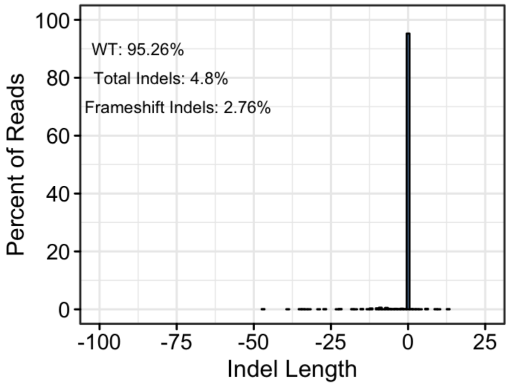


93. C TRAC Ex.3 pmSTOP_1 + BE3 unconc. mRNA 1.5 μg

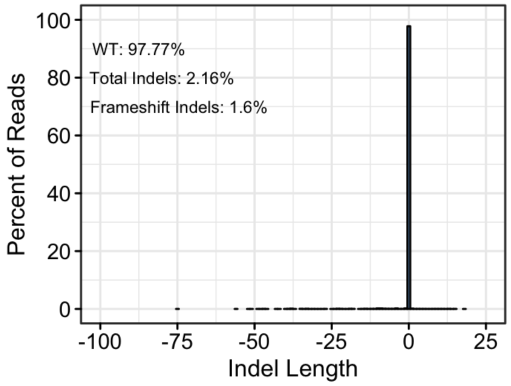


94. A TRAC Ex.3 pmSTOP_1 + BE4 unconc. mRNA 1.5 μg

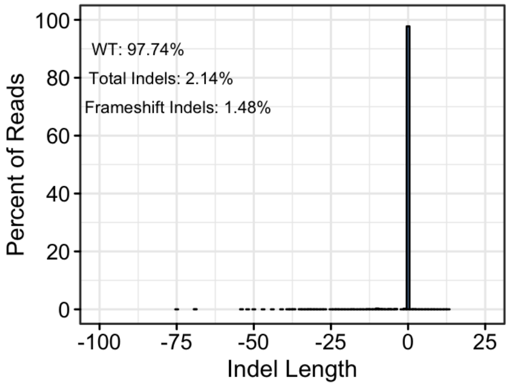


95. B TRAC Ex.3 pmSTOP_1 + BE4 unconc. mRNA 1.5 μg

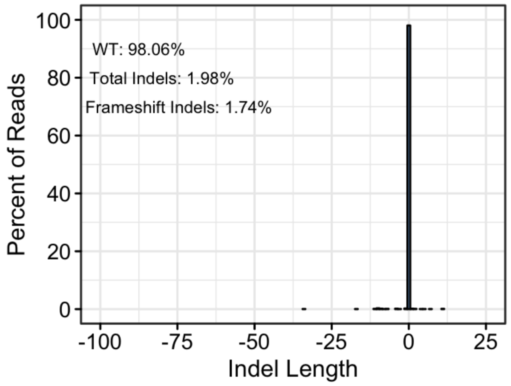


96. C TRAC Ex.3 pmSTOP_1 + BE4 unconc. mRNA 1.5 μg

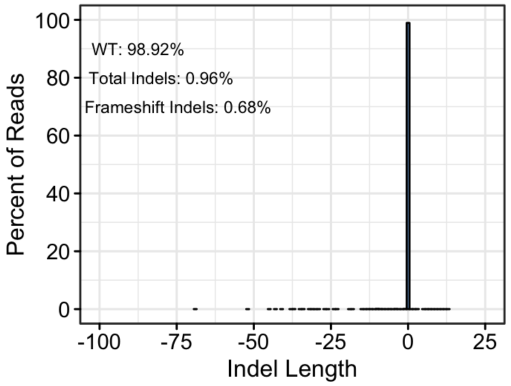


97. D TRAC Ex.3 pmSTOP_1 + Pulse

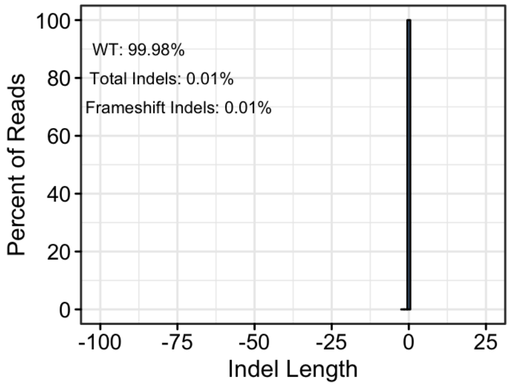


98. A TRAC Ex.3 pmSTOP_1 + Pulse

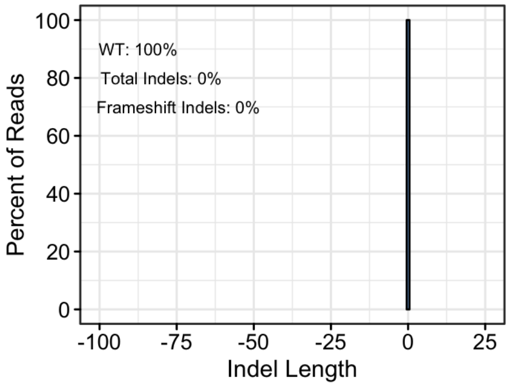

99. A TRAC Ex.3 pmSTOP_2 + BE3 unconc. mRNA 1.5 µg

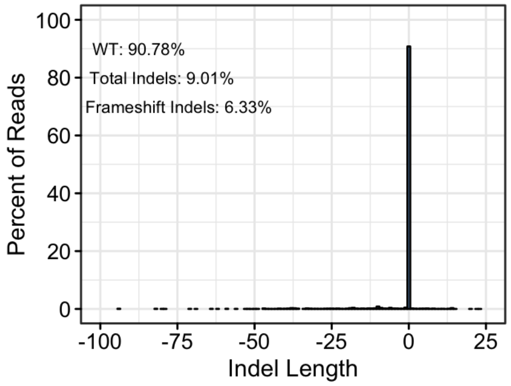


100. B TRAC Ex.3 pmSTOP_2 + BE3 unconc. mRNA 1.5 µg

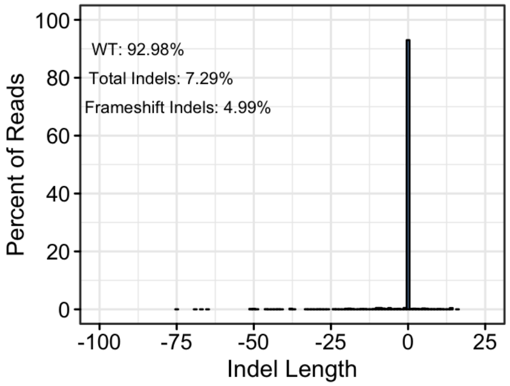


101. C TRAC Ex.3 pmSTOP_2 + BE3 unconc. mRNA 1.5 µg

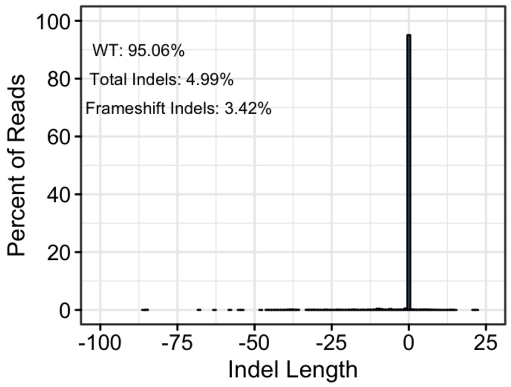


102. A TRAC Ex.3 pmSTOP_2 + BE4 unconc. mRNA 1.5 µg

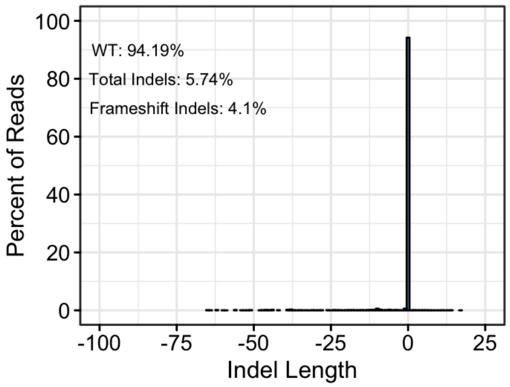


103. B TRAC Ex.3 pmSTOP_2 + BE4 unconc. mRNA 1.5 µg

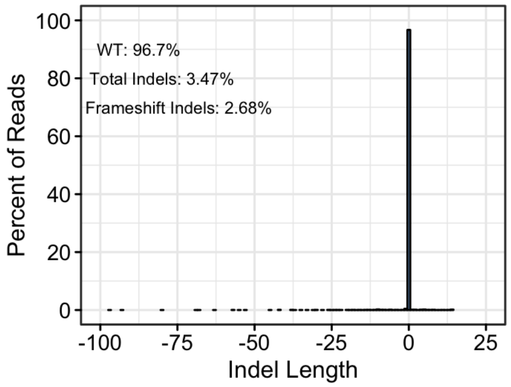


104. C TRAC Ex.3 pmSTOP_2 + BE4 unconc. mRNA 1.5 µg

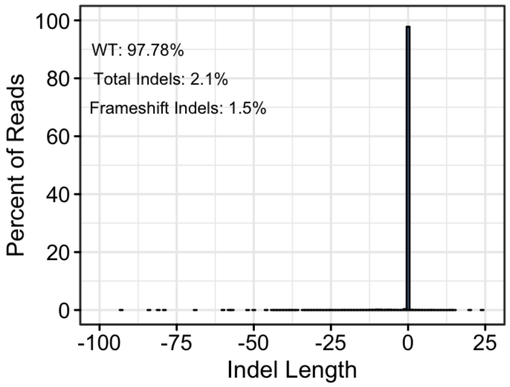


105. D TRAC Ex.3 pmSTOP_2 + Pulse

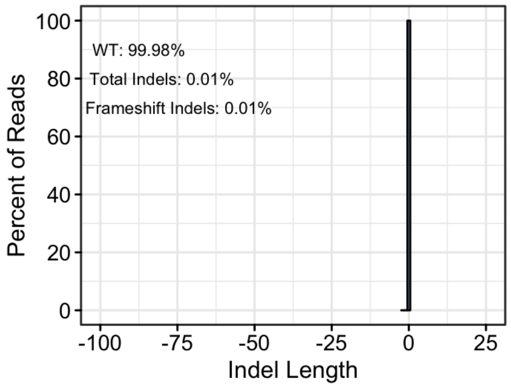


106. A TRAC Ex.3 pmSTOP_2 + Pulse

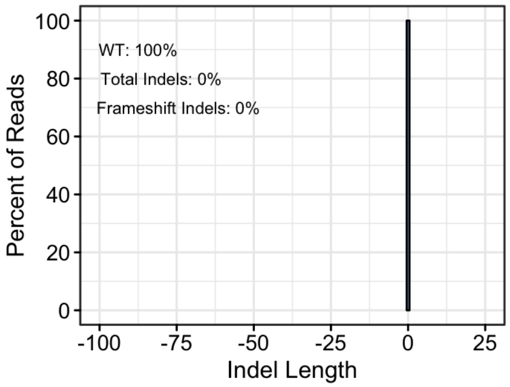

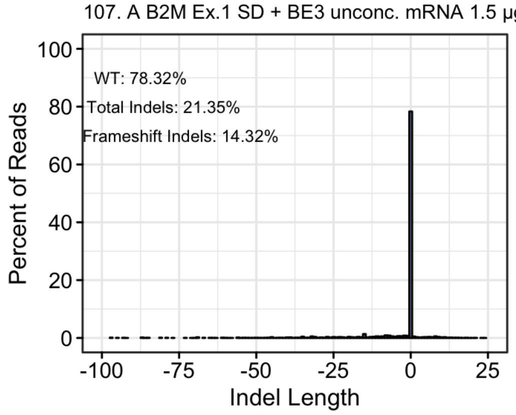
107. A B2M Ex.1 SD + BE3 unconc. mRNA 1.5 µg
WT: 78.32%
Total Indels: 21.35%
Frameshift Indels: 14.32%
Percent of Reads
Indel Length
100
80
60
40
20
0
-100
-75
-50
-25
0
25

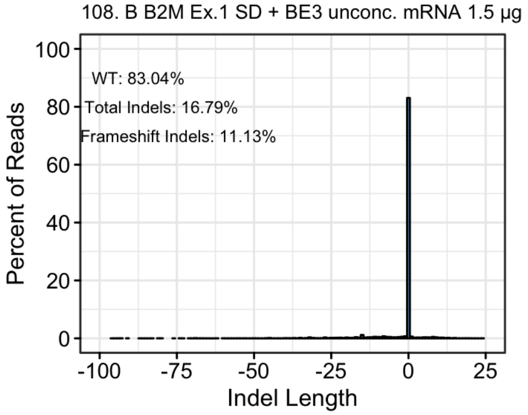
108. B B2M Ex.1 SD + BE3 unconc. mRNA 1.5 µg
WT: 83.04%
Total Indels: 16.79%
Frameshift Indels: 11.13%
Percent of Reads
Indel Length
100
80
60
40
20
0
-100
-75
-50
-25
0
25

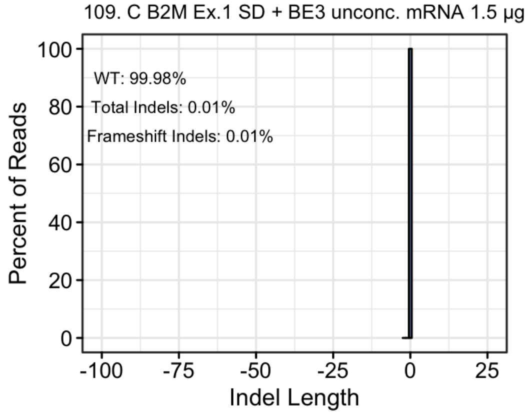
109. C B2M Ex.1 SD + BE3 unconc. mRNA 1.5 µg
WT: 99.98%
Total Indels: 0.01%
Frameshift Indels: 0.01%
Percent of Reads
Indel Length
100
80
60
40
20
0
-100
-75
-50
-25
0
25

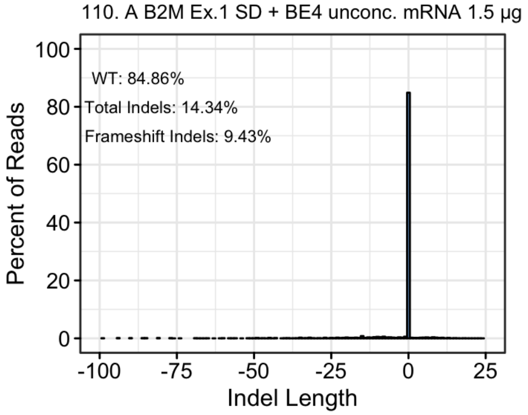
110. A B2M Ex.1 SD + BE4 unconc. mRNA 1.5 µg
WT: 84.86%
Total Indels: 14.34%
Frameshift Indels: 9.43%
Percent of Reads
Indel Length
100
80
60
40
20
0
-100
-75
-50
-25
0
25

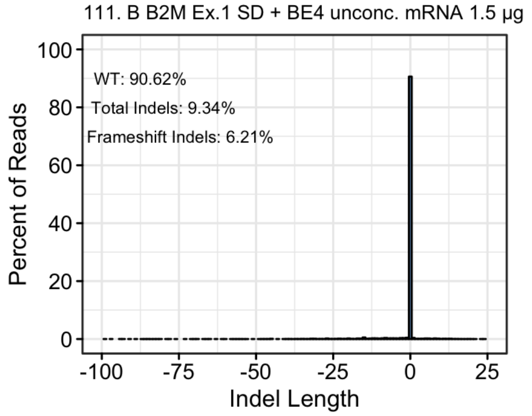
111. B B2M Ex.1 SD + BE4 unconc. mRNA 1.5 µg
WT: 90.62%
Total Indels: 9.34%
Frameshift Indels: 6.21%
Percent of Reads
Indel Length
100
80
60
40
20
0
-100
-75
-50
-25
0
25

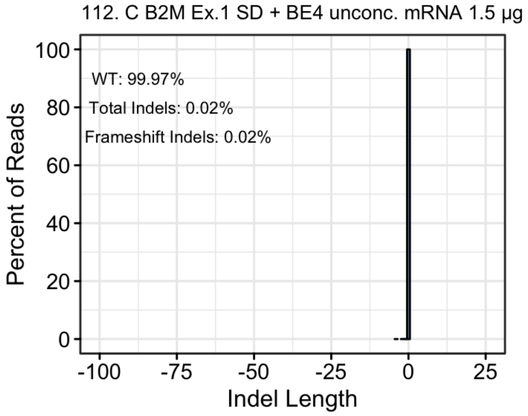
112. C B2M Ex.1 SD + BE4 unconc. mRNA 1.5 µg
WT: 99.97%
Total Indels: 0.02%
Frameshift Indels: 0.02%
Percent of Reads
Indel Length
100
80
60
40
20
0
-100
-75
-50
-25
0
25

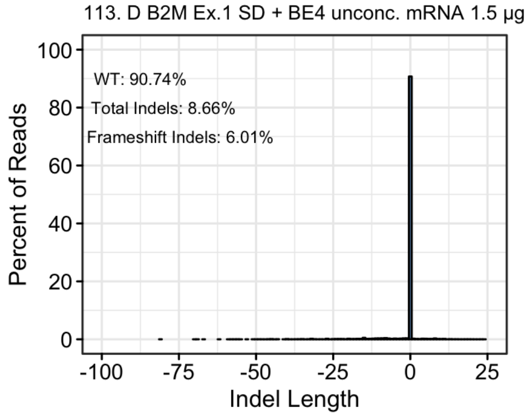
113. D B2M Ex.1 SD + BE4 unconc. mRNA 1.5 µg
WT: 90.74%
Total Indels: 8.66%
Frameshift Indels: 6.01%
Percent of Reads
Indel Length
100
80
60
40
20
0
-100
-75
-50
-25
0
25

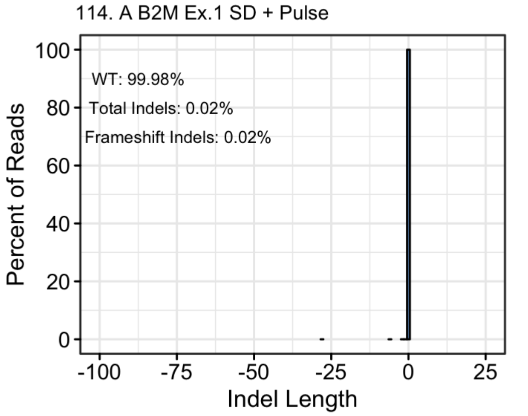
114. A B2M Ex.1 SD + Pulse
WT: 99.98%
Total Indels: 0.02%
Frameshift Indels: 0.02%
Percent of Reads
Indel Length
100
80
60
40
20
0
-100
-75
-50
-25
0
25

115. A B2M Ex.2 pmSTOP + BE3 unconc. mRNA 1.5 µg

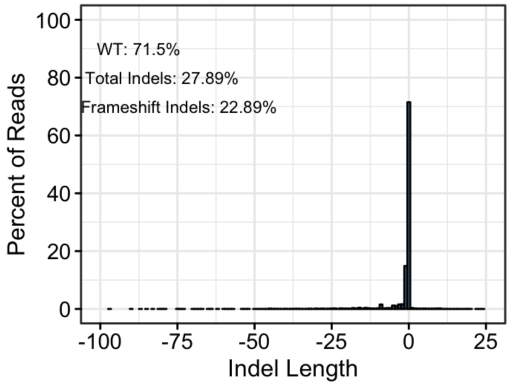


116. C B2M Ex.2 pmSTOP + BE3 unconc. mRNA 1.5 µg

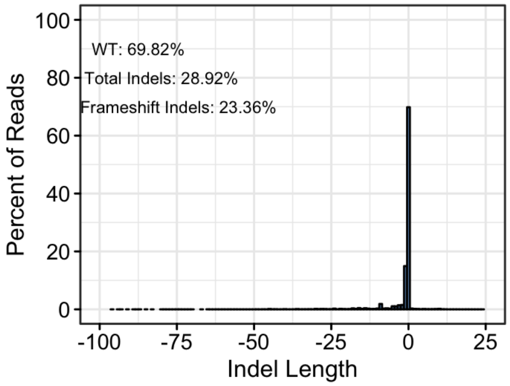


117. A B2M Ex.2 pmSTOP + BE4 unconc. mRNA 1.5 µg

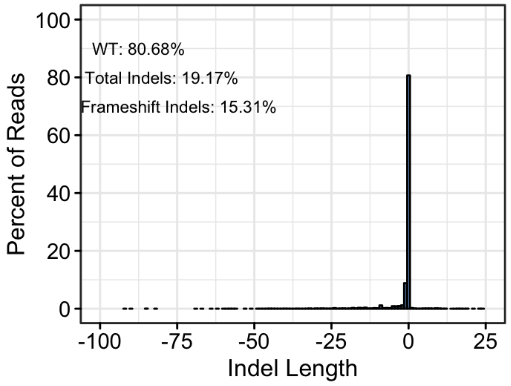


118. C B2M Ex.2 pmSTOP + BE4 unconc. mRNA 1.5 µg

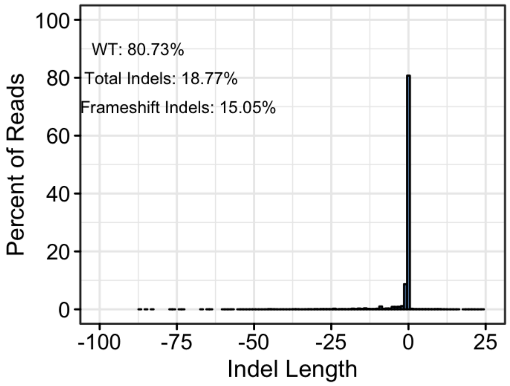


119. A B2M Ex.2 pmSTOP + Pulse

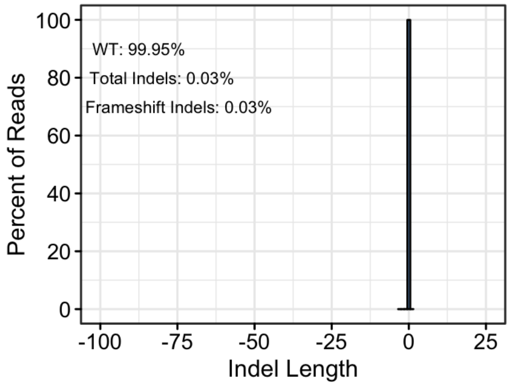


120. A B2M Ex.3 SA + BE3 unconc. mRNA 1.5 µg

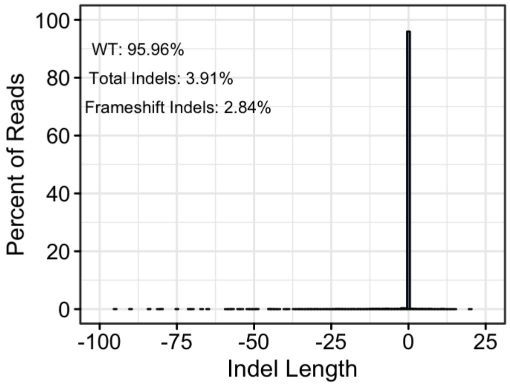


121. B B2M Ex.3 SA + BE3 unconc. mRNA 1.5 µg

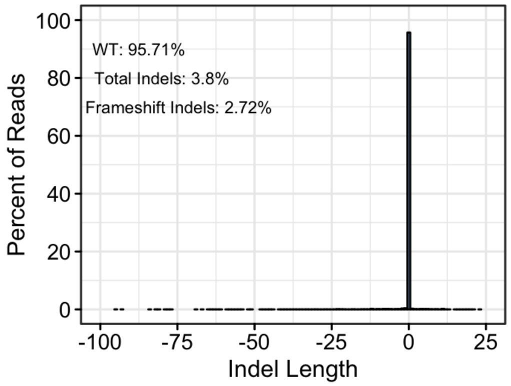


123. A B2M Ex.3 SA + BE4 unconc. mRNA 1.5 µg

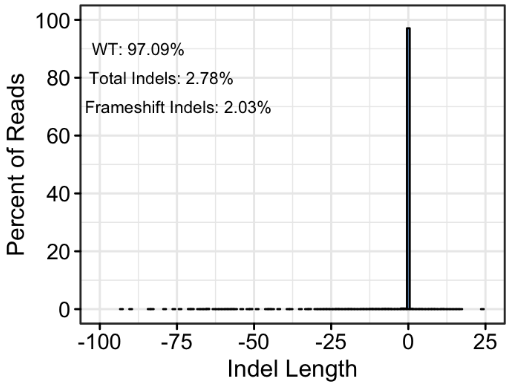

124. B B2M Ex.3 SA + BE4 unconc. mRNA 1.5 µg

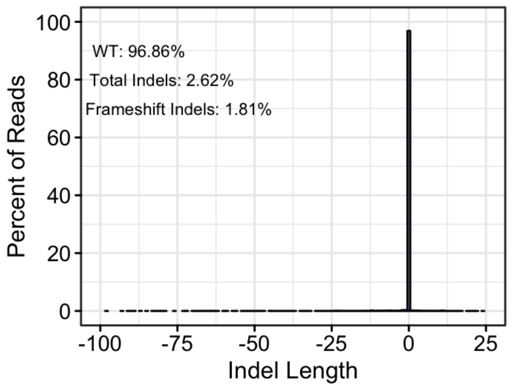


125. C B2M Ex.3 SA + BE4 unconc. mRNA 1.5 µg

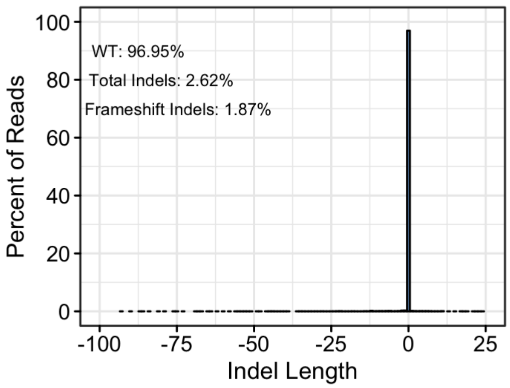


126. A 3G TRAC Ex.3 SA + Pulse

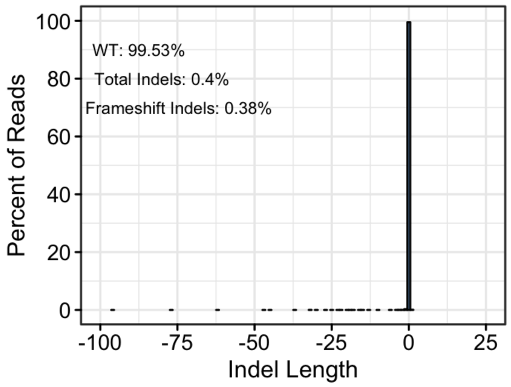


127. A 3G TRAC Ex.3 SA + Pulse

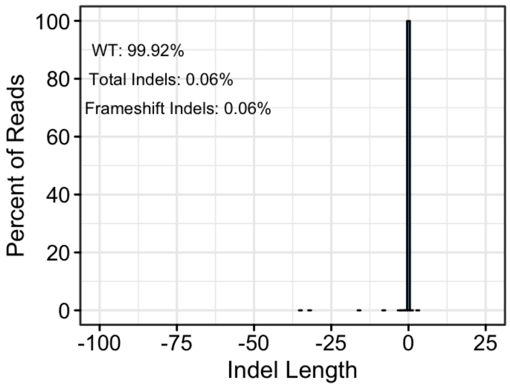


128. B 3G TRAC Ex.3 SA + Pulse

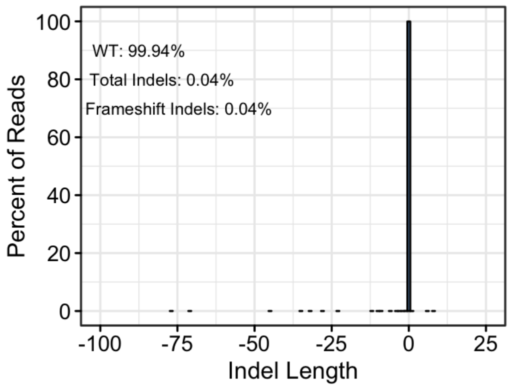


129. C 3G TRAC Ex.3 SA + Pulse

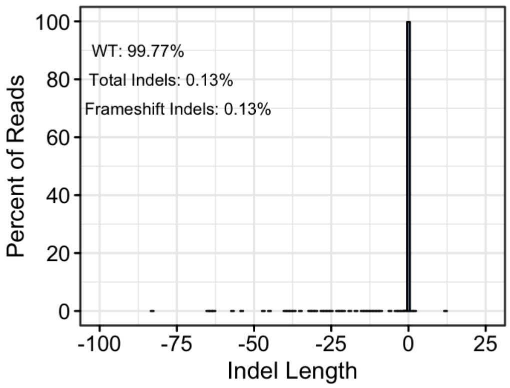


130. A 3G TRAC Ex.3 SA + Pulse

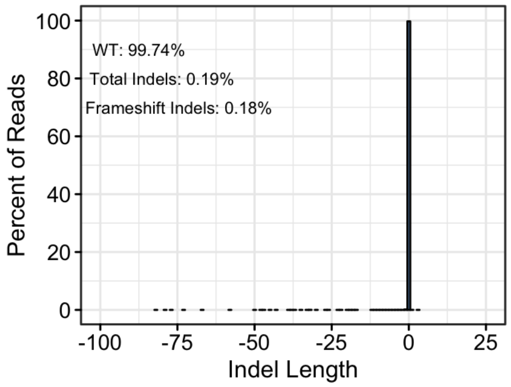


131. B 3G TRAC Ex.3 SA + Pulse

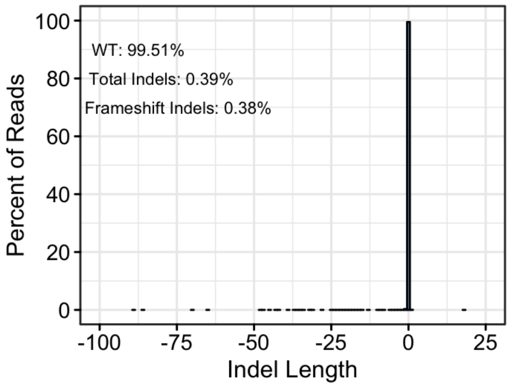

132. A 3G TRAC Ex.3 SA + Pulse

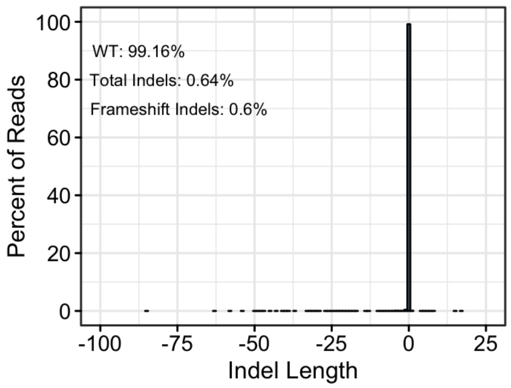


133. B 3G TRAC Ex.3 SA + Pulse

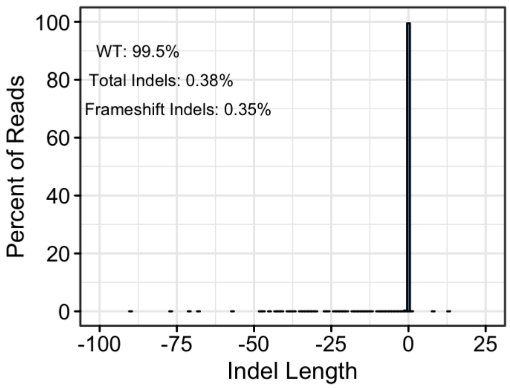


134. A 3G TRAC Ex.3 SA + BE3 conc. mRNA 3 μg

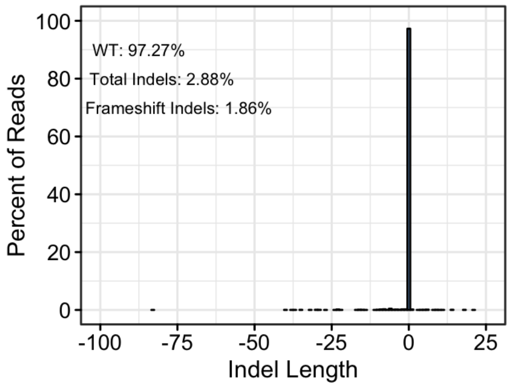


135. B 3G TRAC Ex.3 SA + BE3 conc. mRNA 3 μg

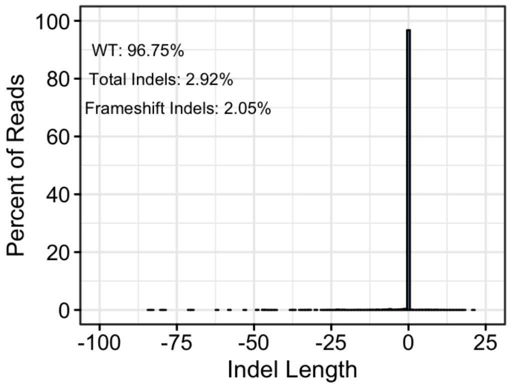


136. A 3G TRAC Ex.3 SA + BE4 conc. mRNA 3 μg

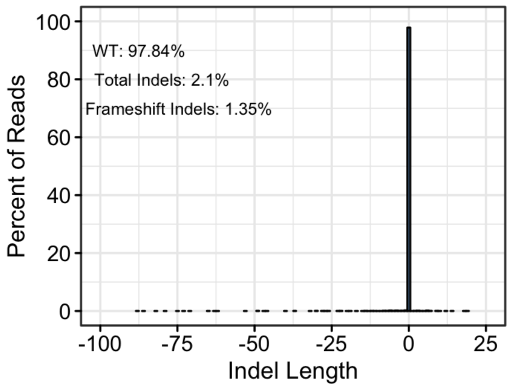


137. B 3G TRAC Ex.3 SA + BE4 conc. mRNA 3 μg

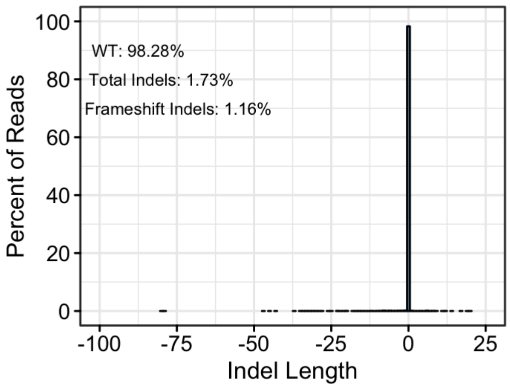


138. A 3G TRAC Ex.3 SA + BE4 RNP

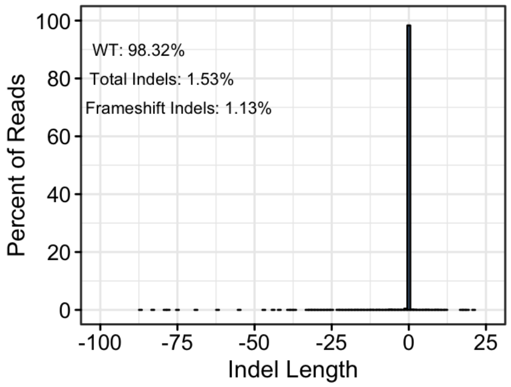


139. B 3G TRAC Ex.3 SA + BE4 RNP

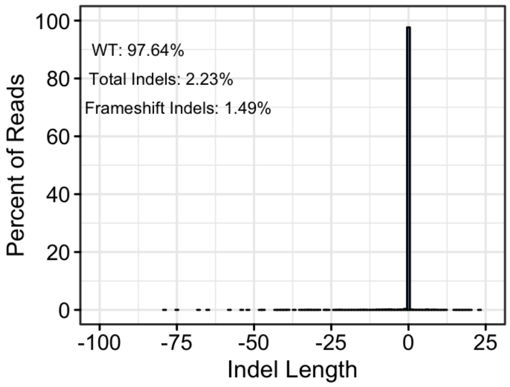

140. A 3G TRAC Ex.3 SA + coBE4 mRNA 1.5 µg

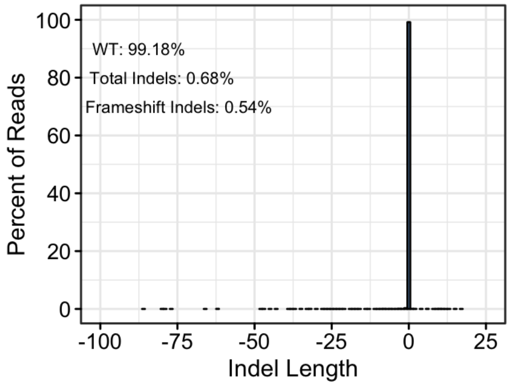


141. B 3G TRAC Ex.3 SA + coBE4 mRNA 1.5 µg

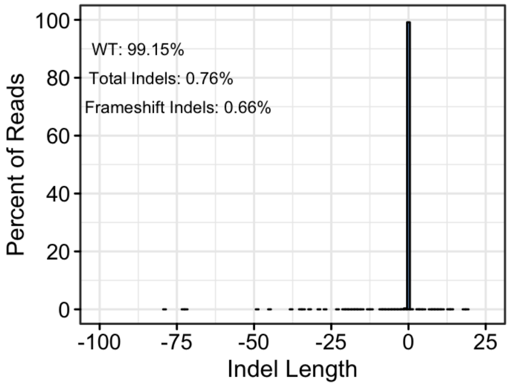


142. A 3G TRAC Ex.3 SA + coBE4 mRNA 4 µg

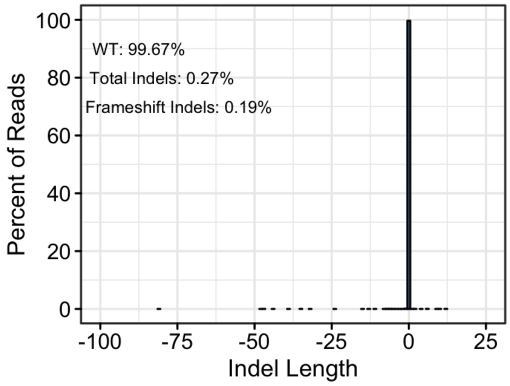


143. A 3G TRAC Ex.3 SA + coBE4 mRNA 4 µg

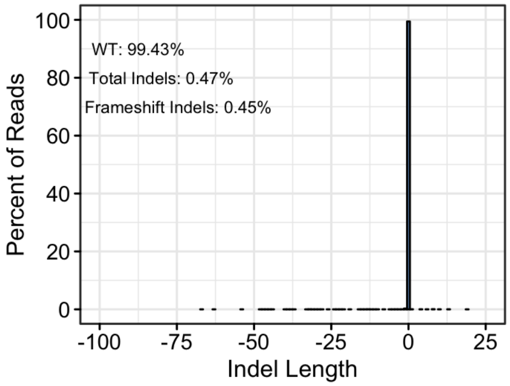


144. B 3G TRAC Ex.3 SA + coBE4 mRNA 4 µg

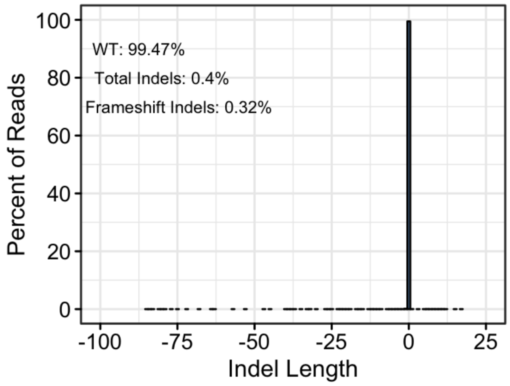


145. B 3G TRAC Ex.3 SA + coBE4 mRNA 4 µg

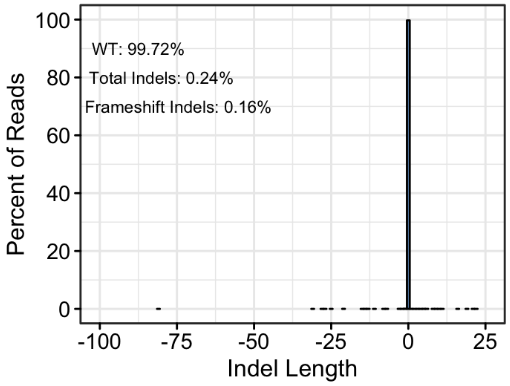


146. C 3G TRAC Ex.3 SA + coBE4 mRNA 4 µg

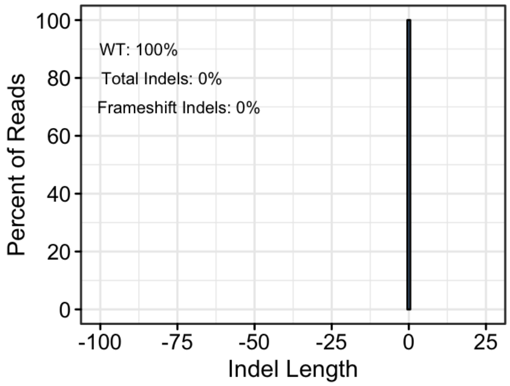


147. A 3G TRAC Ex.3 SA + Cas9 mRNA 1.5 µg

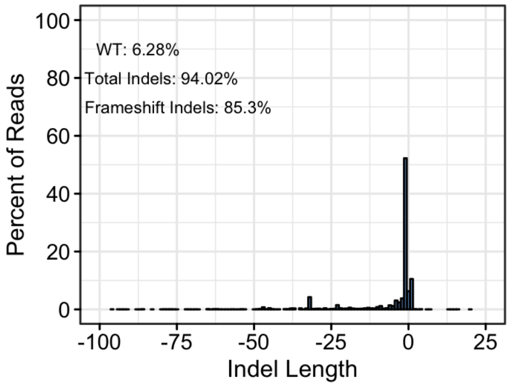

148. B 3G TRAC Ex.3 SA + Cas9 mRNA 1.5 µg

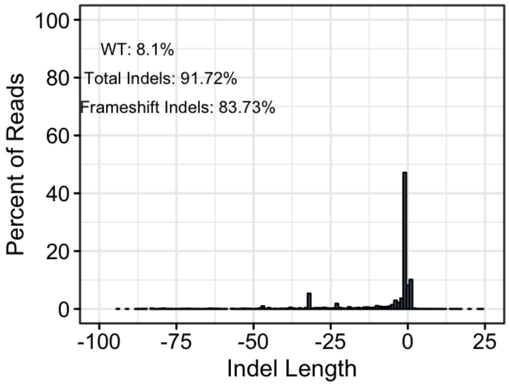


149. A 3G TRAC Ex.3 SA + Cas9 mRNA 1.5 µg

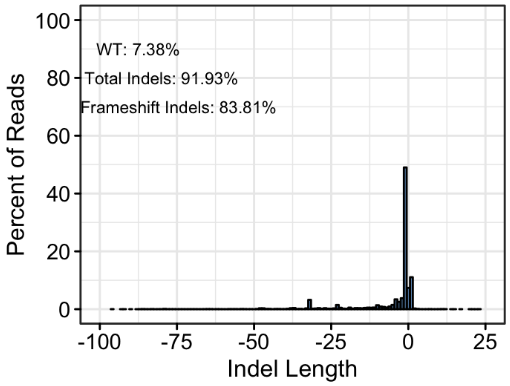


150. B 3G TRAC Ex.3 SA + Cas9 mRNA 1.5 µg

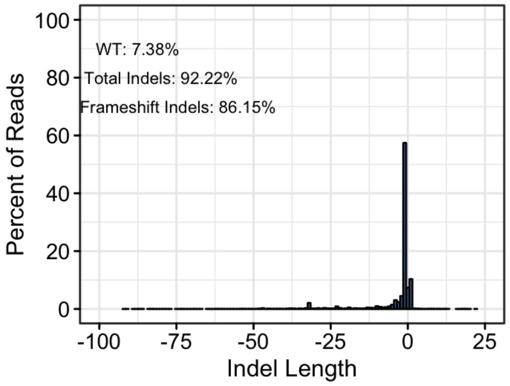


151. A 3G TRAC Ex.3 SA + Cas9 mRNA 4 µg

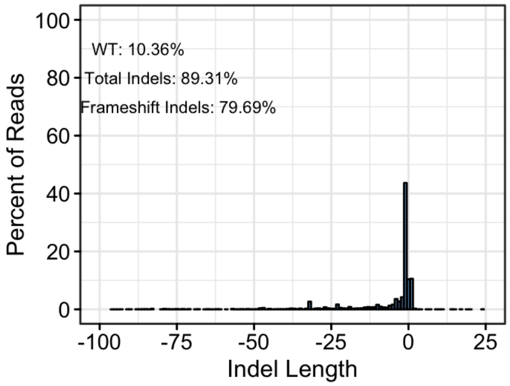


152. B 3G TRAC Ex.3 SA + Cas9 mRNA 4 µg

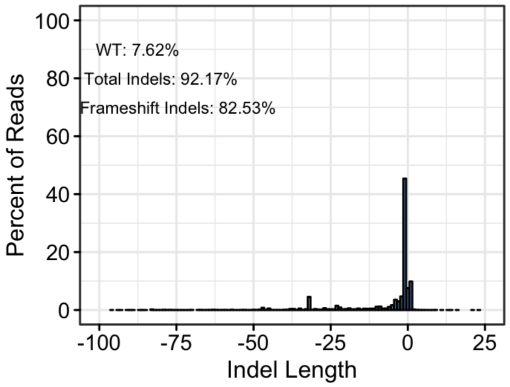


153. A 3G TRAC Ex.3 SA + Cas9 mRNA 4 µg

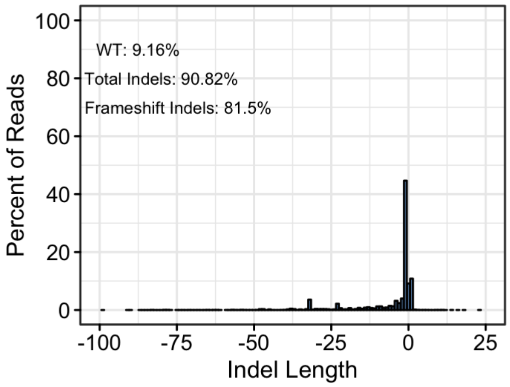


154. B 3G TRAC Ex.3 SA + Cas9 mRNA 4 µg

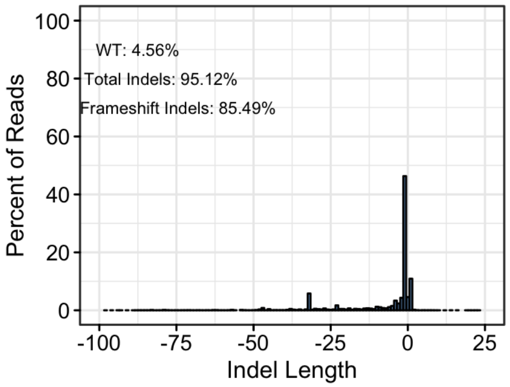


155. A 3G TRAC Ex.3 SA + Cas9 RNP

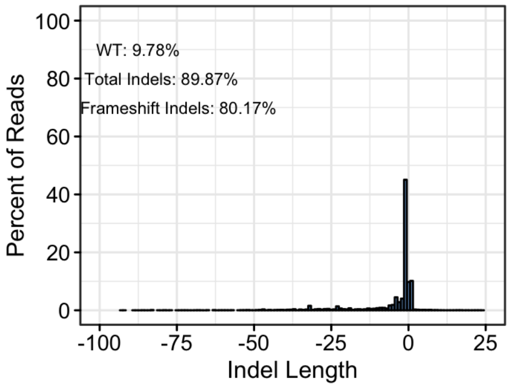

156. B 3G TRAC Ex.3 SA + Cas9 RNP

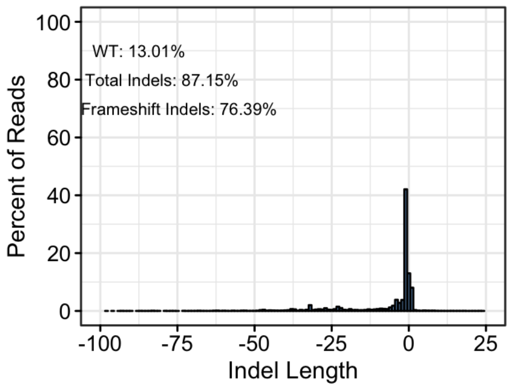


157. A 3G PDCD1 Ex.1 SD + Pulse

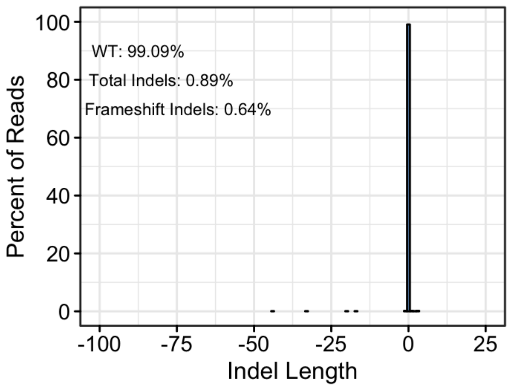


158. B 3G PDCD1 Ex.1 SD + Pulse

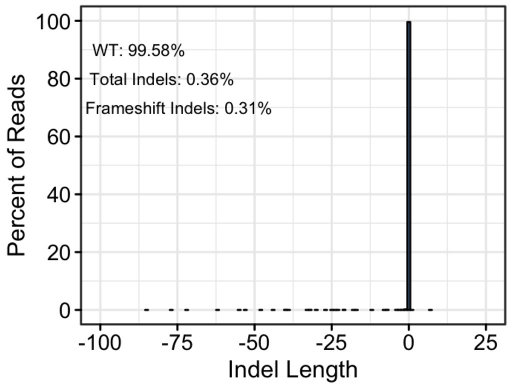


159. A 3G PDCD1 Ex.1 SD + Pulse

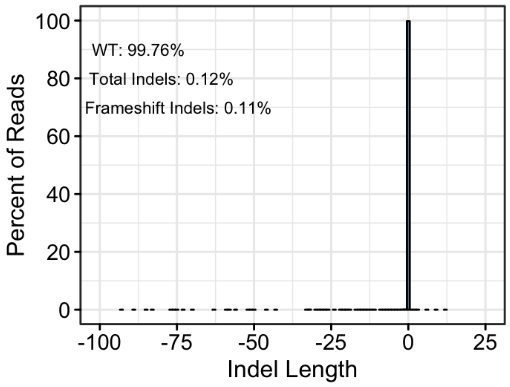


160. B 3G PDCD1 Ex.1 SD + Pulse

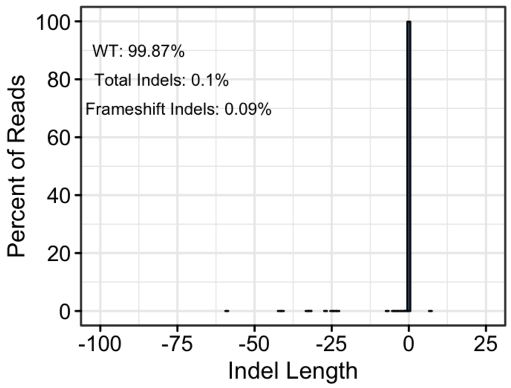


161. C 3G PDCD1 Ex.1 SD + Pulse

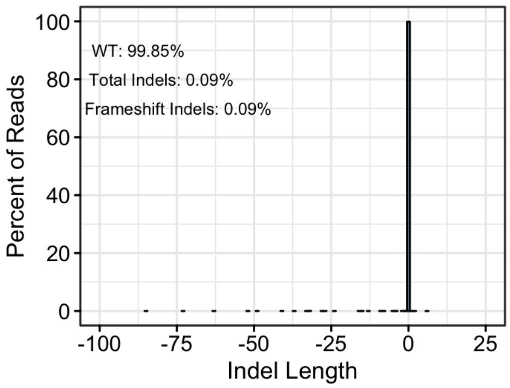


162. A 3G PDCD1 Ex.1 SD + Pulse

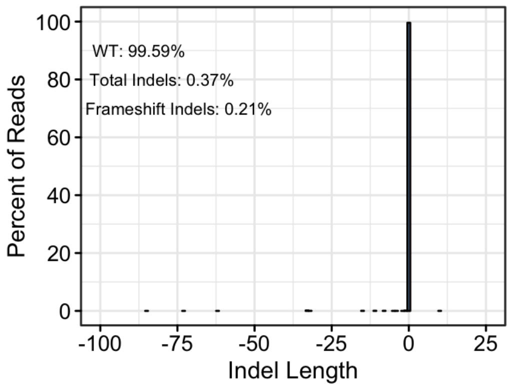


163. A 3G PDCD1 Ex.1 SD + Pulse

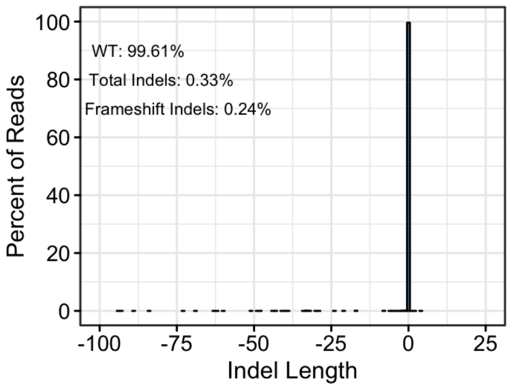

164. B 3G PDCD1 Ex.1 SD + Pulse

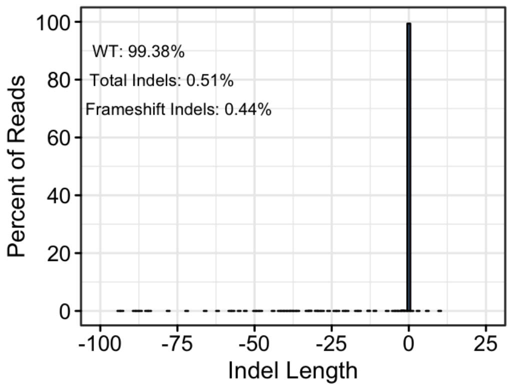


165. A 3G PDCD1 Ex.1 SD + Pulse

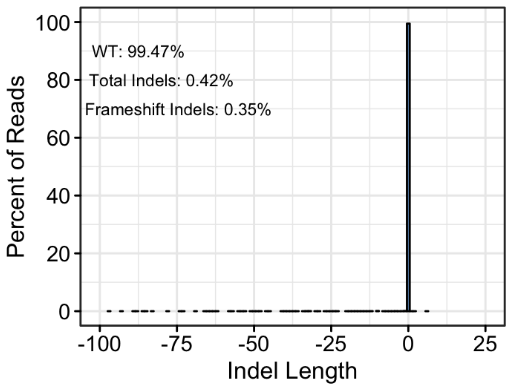


166. B 3G PDCD1 Ex.1 SD + Pulse

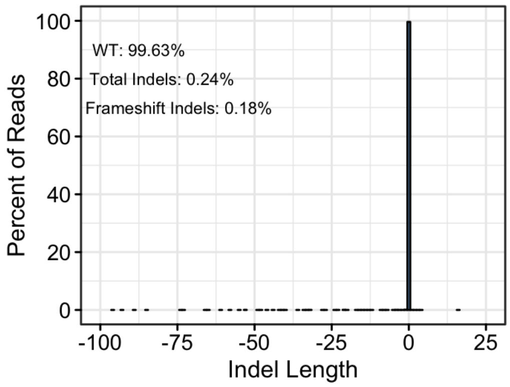


167. A 3G PDCD1 Ex.1 SD + BE3 conc. mRNA 3 μg

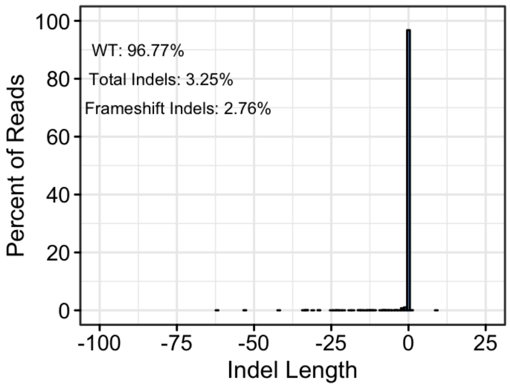


168. B 3G PDCD1 Ex.1 SD + BE3 conc. mRNA 3 μg

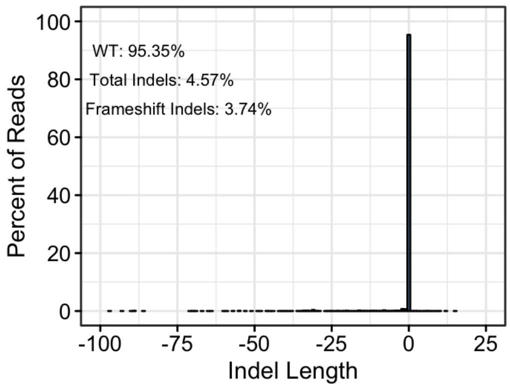


169. A 3G PDCD1 Ex.1 SD + BE4 conc. mRNA 3 μg

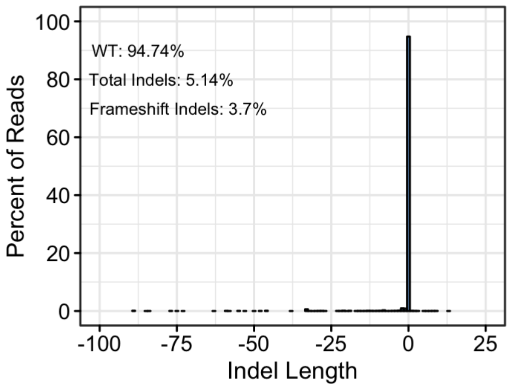


170. B 3G PDCD1 Ex.1 SD + BE4 conc. mRNA 3 μg

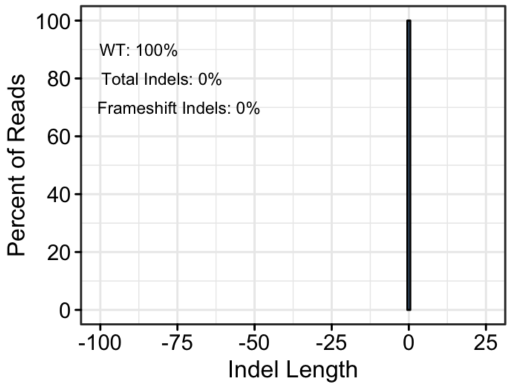


171. A 3G PDCD1 Ex.1 SD + BE4 RNP

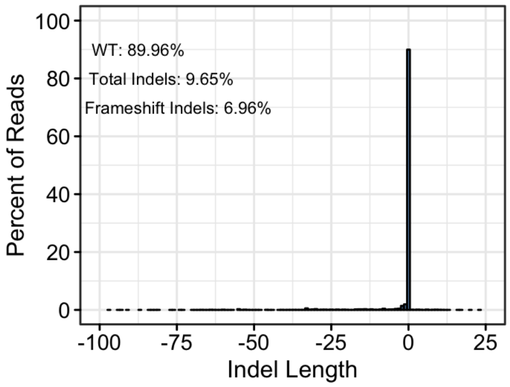

172. B 3G PDCD1 Ex.1 SD + BE4 RNP

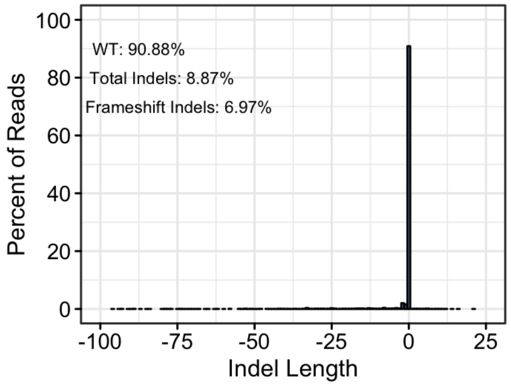


173. A 3G PDCD1 Ex.1 SD + coBE4 mRNA 1.5 μg

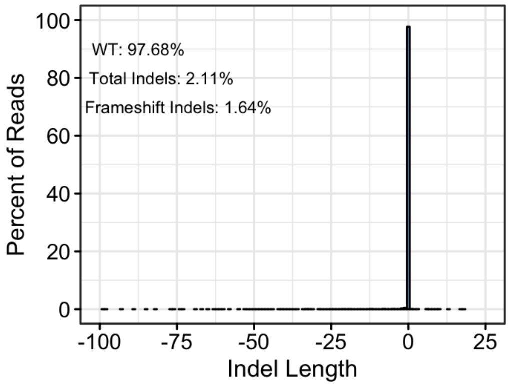


174. B 3G PDCD1 Ex.1 SD + coBE4 mRNA 1.5 μg

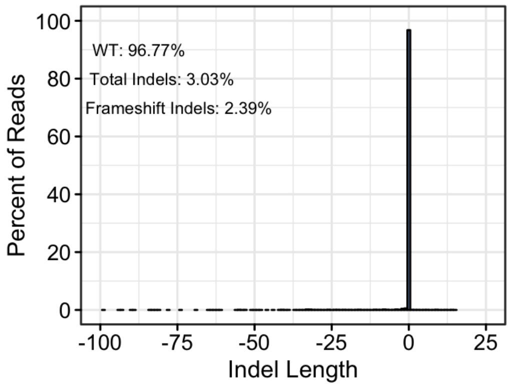


175. A 3G PDCD1 Ex.1 SD + coBE4 mRNA 4 μg

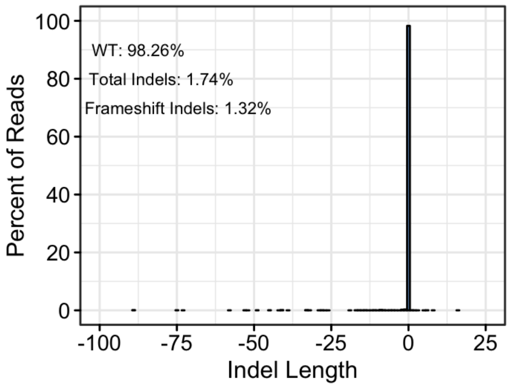


176. B 3G PDCD1 Ex.1 SD + coBE4 mRNA 4 μg

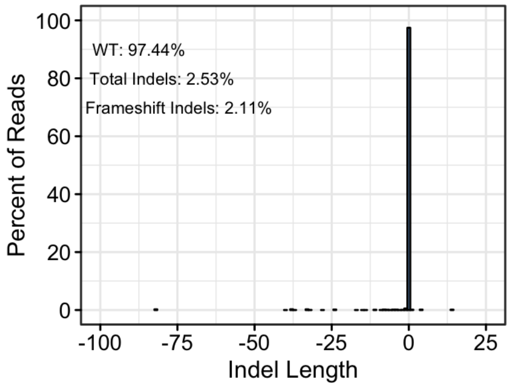


177. A 3G PDCD1 Ex.1 SD + coBE4 mRNA 4 μg

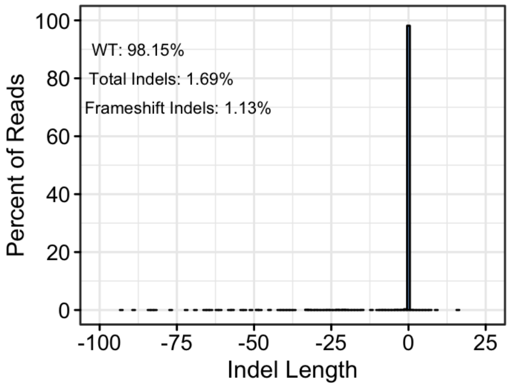


178. B 3G PDCD1 Ex.1 SD + coBE4 mRNA 4 μg

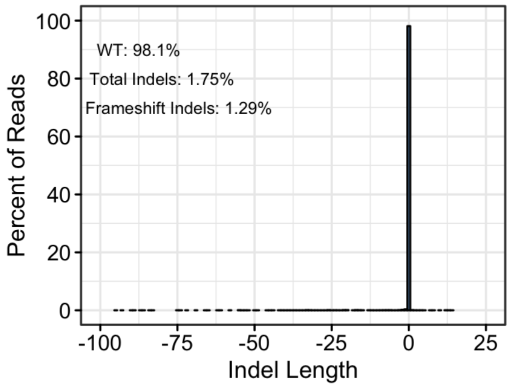


179. B 3G PDCD1 Ex.1 SD + coBE4 mRNA 4 μg

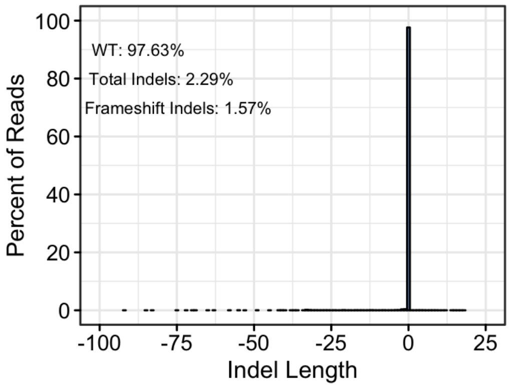

180. A 3G PDCD1 Ex.1 SD + Cas9 mRNA 1.5 µg

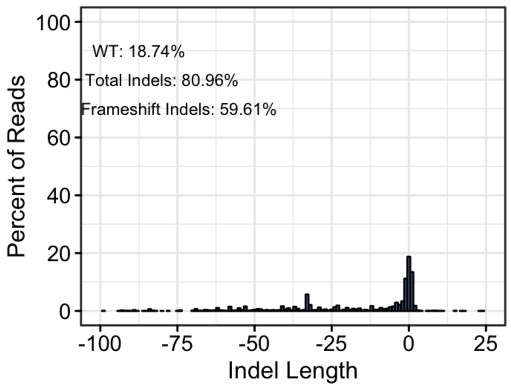


181. B 3G PDCD1 Ex.1 SD + Cas9 mRNA 1.5 µg

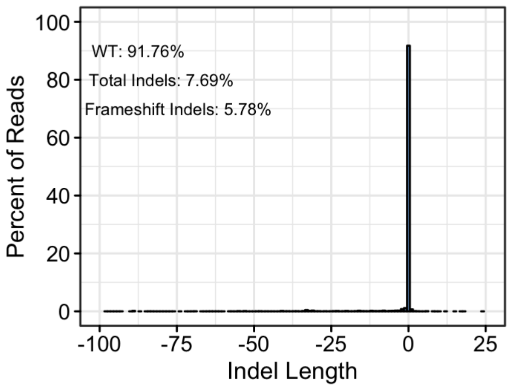


182. A 3G PDCD1 Ex.1 SD + Cas9 mRNA 1.5 µg

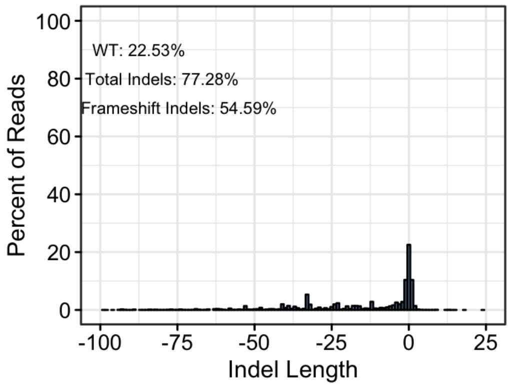


183. B 3G PDCD1 Ex.1 SD + Cas9 mRNA 1.5 µg

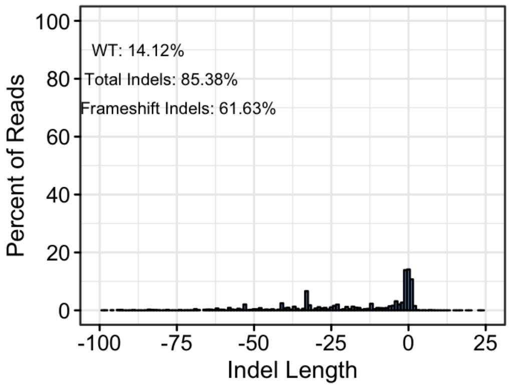


184. A 3G PDCD1 Ex.1 SD + Cas9 mRNA 4 µg

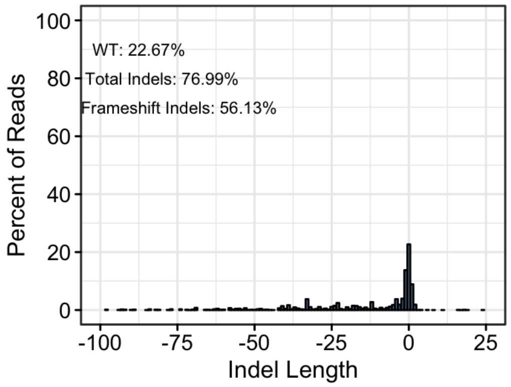


185. B 3G PDCD1 Ex.1 SD + Cas9 mRNA 4 µg

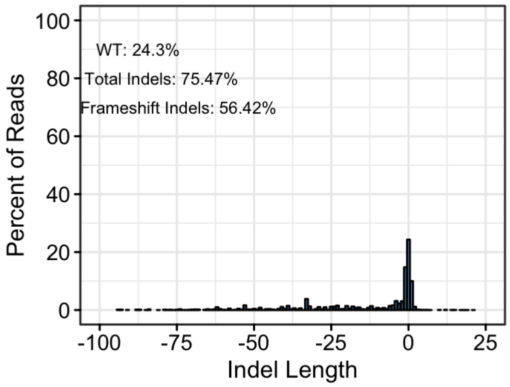


186. A 3G PDCD1 Ex.1 SD + Cas9 mRNA 4 µg

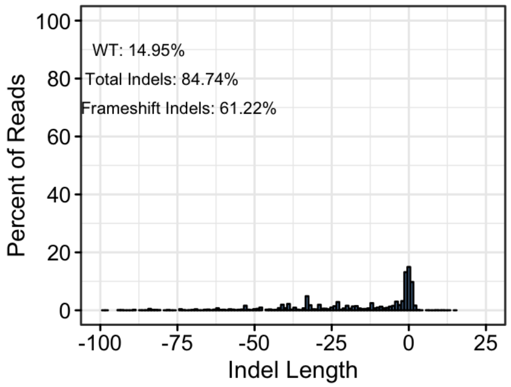


187. B 3G PDCD1 Ex.1 SD + Cas9 mRNA 4 µg

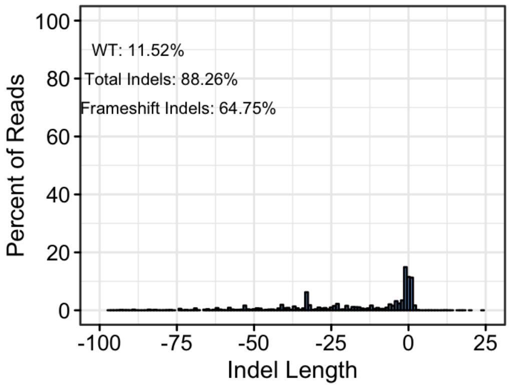

188. A 3G PDCD1 Ex.1 SD + Cas9 RNP

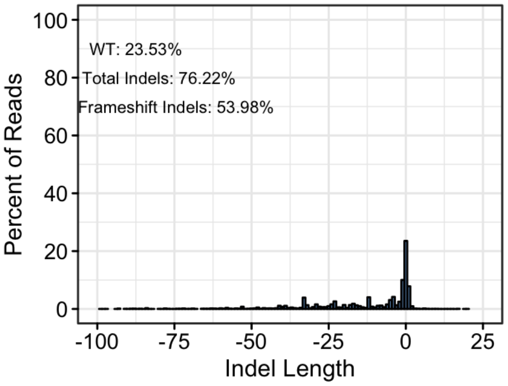


189. B 3G PDCD1 Ex.1 SD + Cas9 RNP

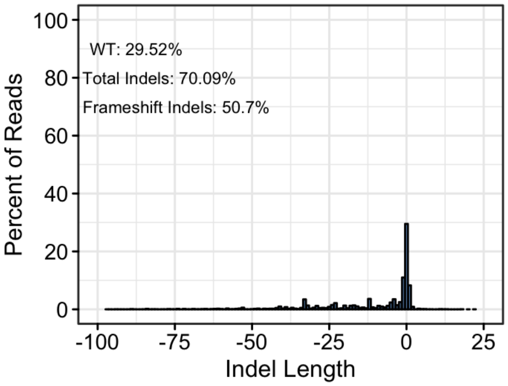


190. A 3G B2M Ex.1 SD + Pulse

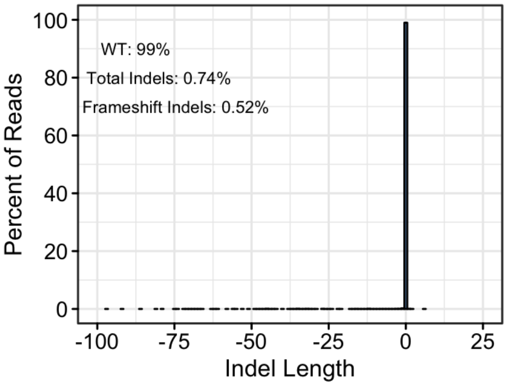


191. B 3G B2M Ex.1 SD + Pulse

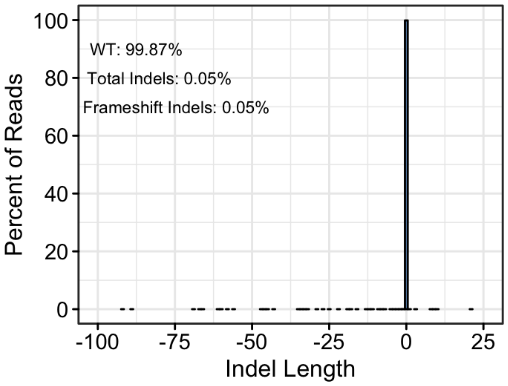


192. A 3G B2M Ex.1 SD + Pulse

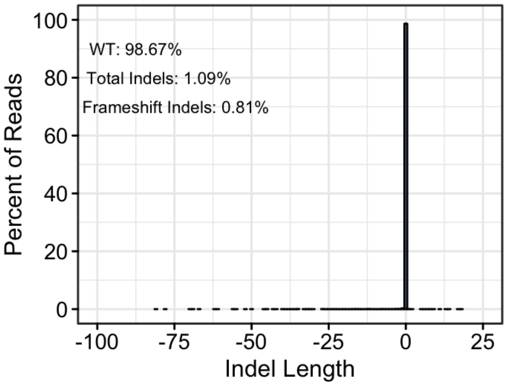


193. B 3G B2M Ex.1 SD + Pulse

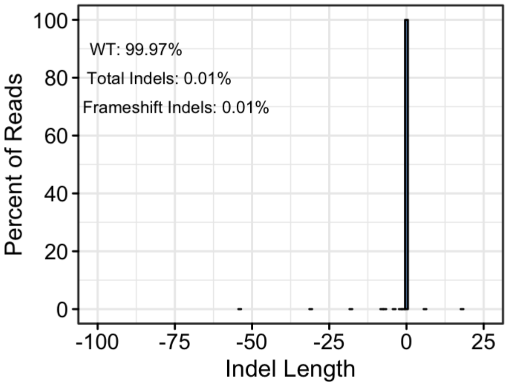


194. A 3G B2M Ex.1 SD + Pulse

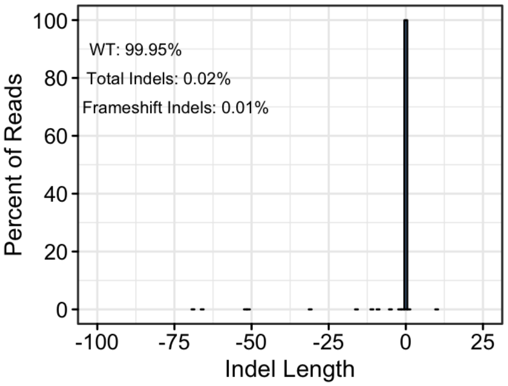


195. B 3G B2M Ex.1 SD + Pulse

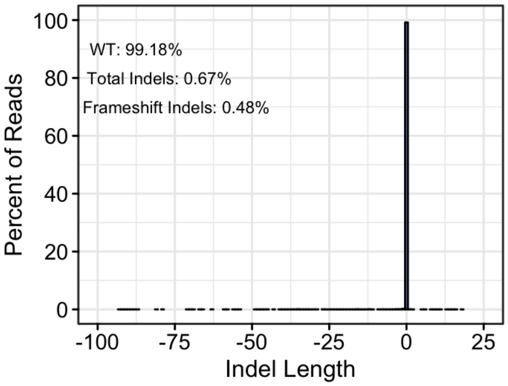

196. A 3G B2M Ex.1 SD + Pulse

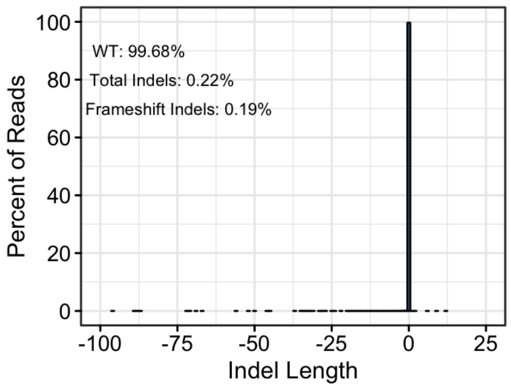


197. B 3G B2M Ex.1 SD + Pulse

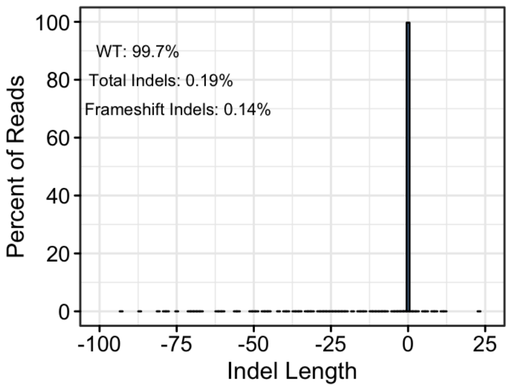


198. A 3G B2M Ex.1 SD + BE3 conc. mRNA 3 μg

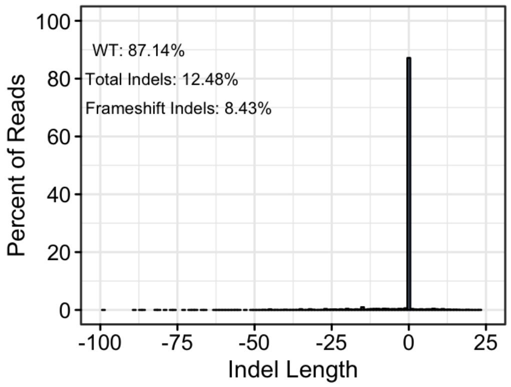


199. B 3G B2M Ex.1 SD + BE3 conc. mRNA 3 μg

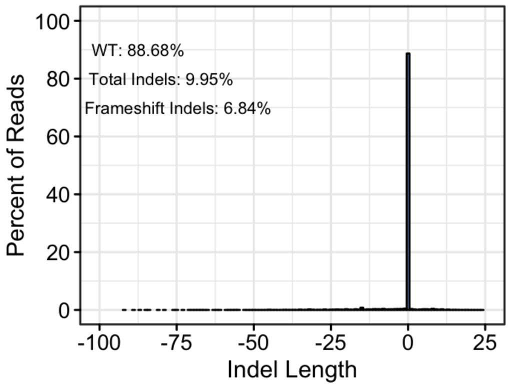


200. A 3G B2M Ex.1 SD + BE4 conc. mRNA 3 μg

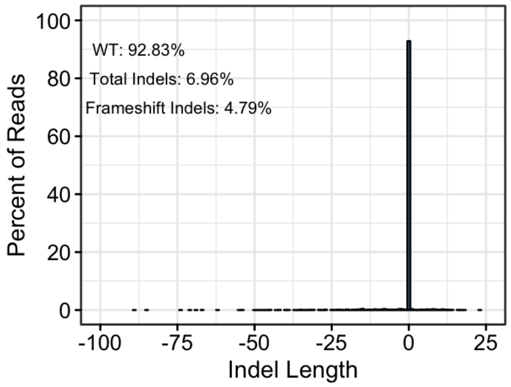


201. B 3G B2M Ex.1 SD + BE4 conc. mRNA 3 μg

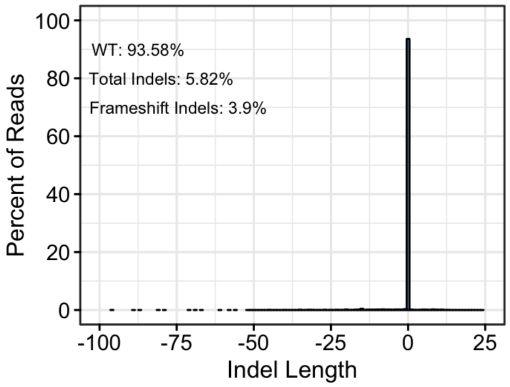


202. A 3G B2M Ex.1 SD + BE4 RNP

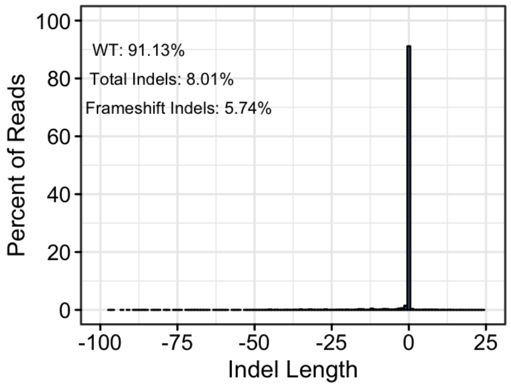


203. B 3G B2M Ex.1 SD + BE4 RNP

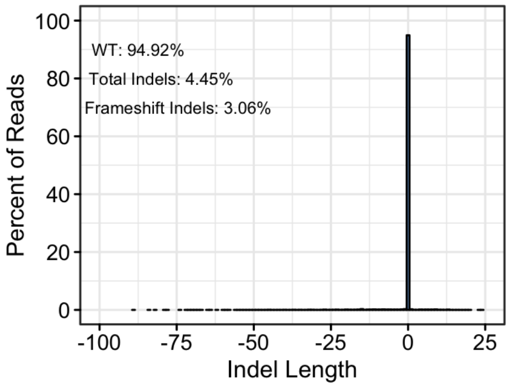

204. A 3G B2M Ex.1 SD + coBE4 mRNA 1.5 µg

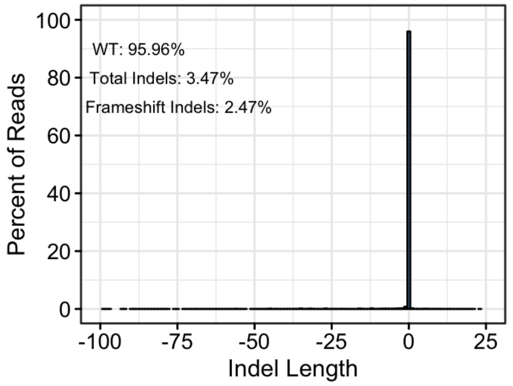


205. B 3G B2M Ex.1 SD + coBE4 mRNA 1.5 µg

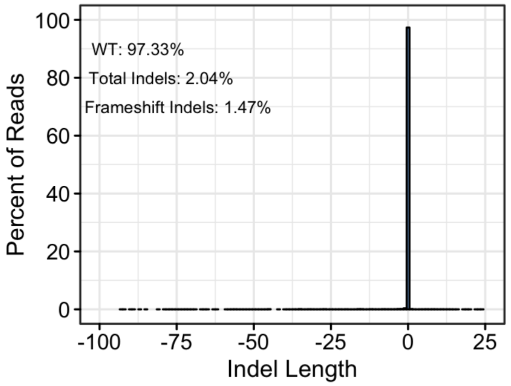


206. A 3G B2M Ex.1 SD + coBE4 mRNA 4 µg

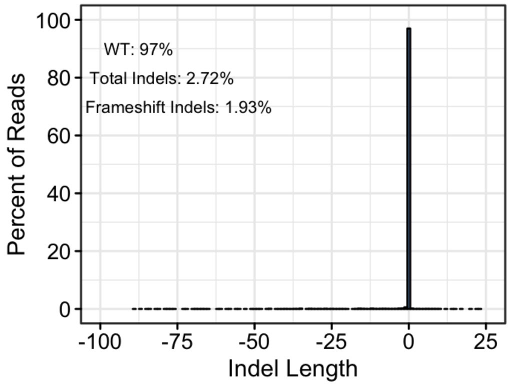


207. B 3G B2M Ex.1 SD + coBE4 mRNA 4 µg

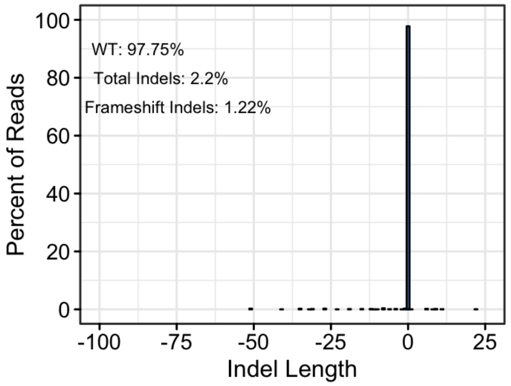


208. A 3G B2M Ex.1 SD + coBE4 mRNA 4 µg

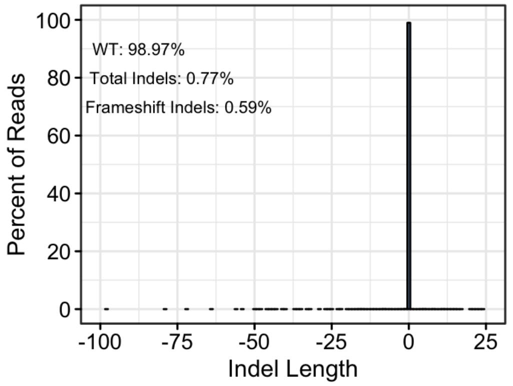


209. B 3G B2M Ex.1 SD + coBE4 mRNA 4 µg

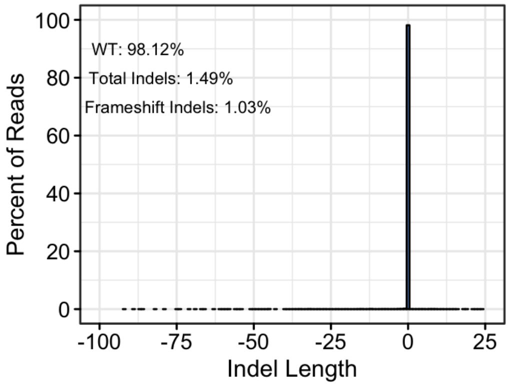


210. B 3G B2M Ex.1 SD + coBE4 mRNA 4 µg

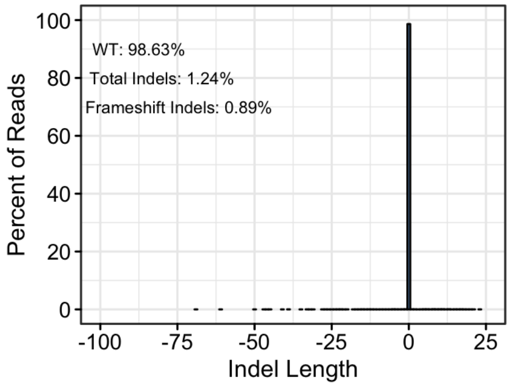


211. A 3G B2M Ex.1 SD + Cas9 mRNA 1.5 µg

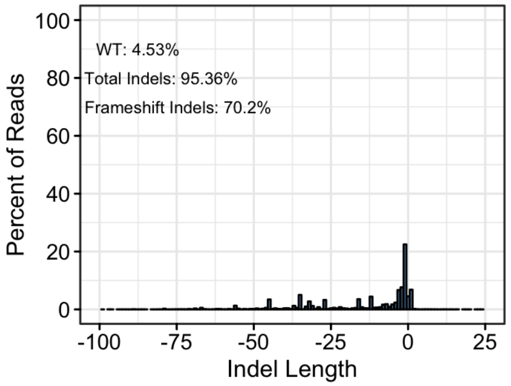

212. B 3G B2M Ex.1 SD + Cas9 mRNA 1.5 µg

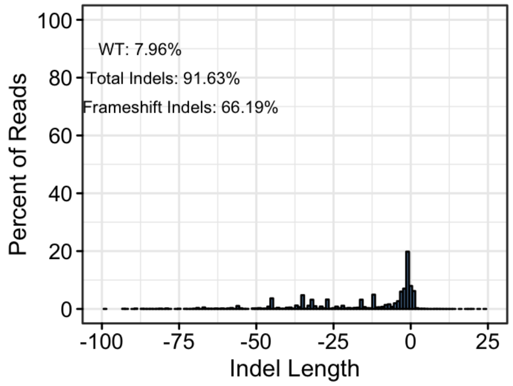


213. A 3G B2M Ex.1 SD + Cas9 mRNA 1.5 µg

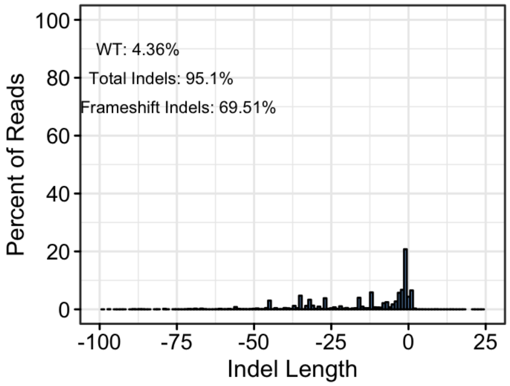


214. B 3G B2M Ex.1 SD + Cas9 mRNA 1.5 µg

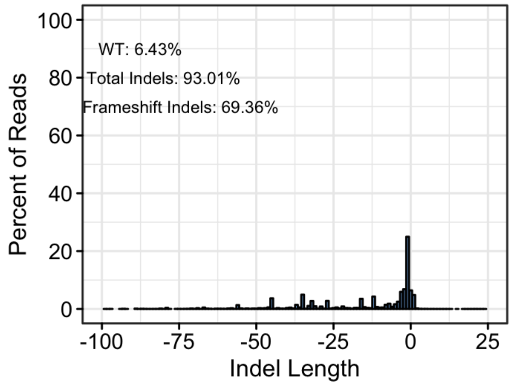


215. A 3G B2M Ex.1 SD + Cas9 mRNA 4 µg

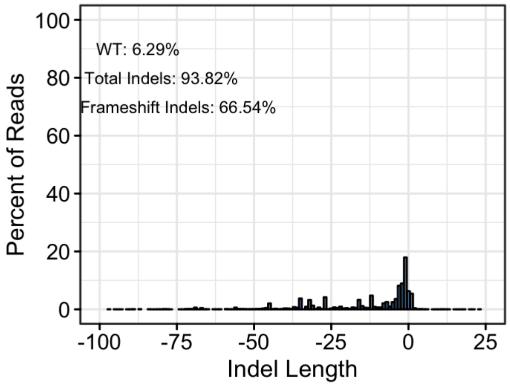


216. B 3G B2M Ex.1 SD + Cas9 mRNA 4 µg

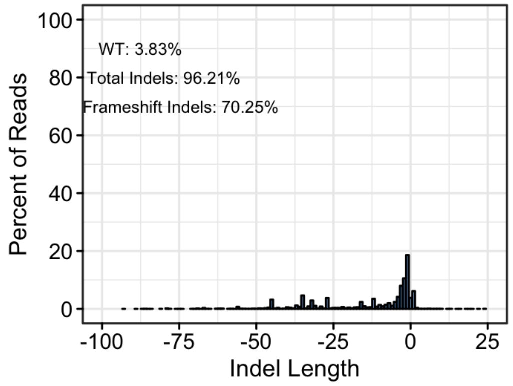


217. A 3G B2M Ex.1 SD + Cas9 mRNA 4 µg

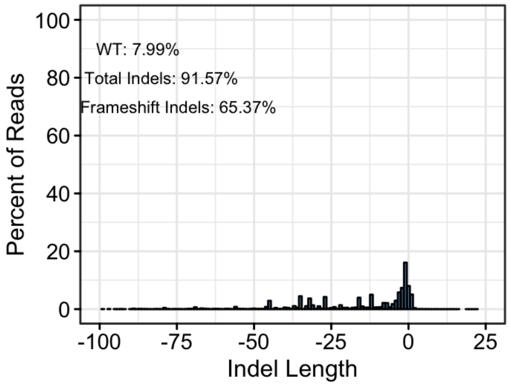


218. B 3G B2M Ex.1 SD + Cas9 mRNA 4 µg

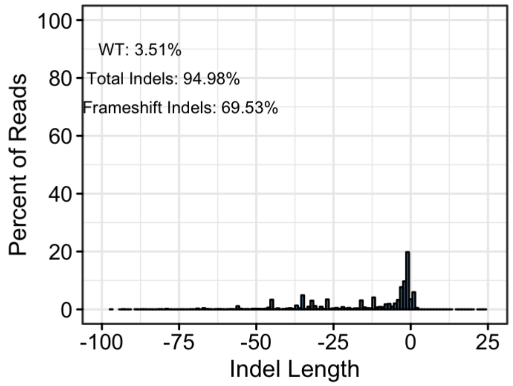


219. A 3G B2M Ex.1 SD + Cas9 RNP

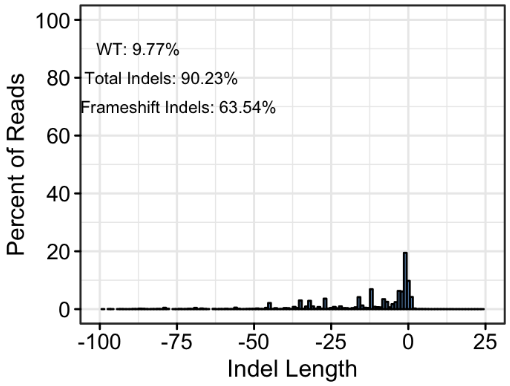

220. B 3G B2M Ex.1 SD + Cas9 RNP

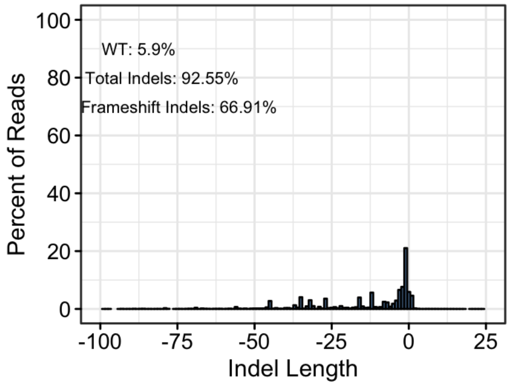


221. A 3G B2M Ex.1 SD + Pulse

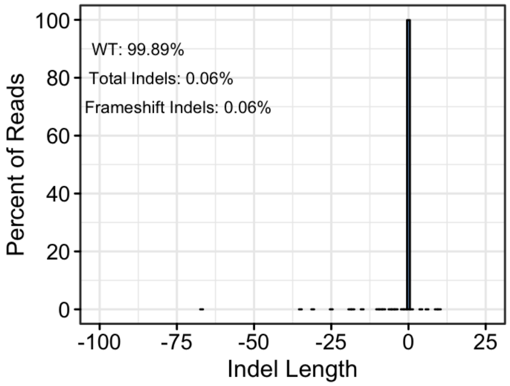


222. B 3G B2M Ex.1 SD + Pulse

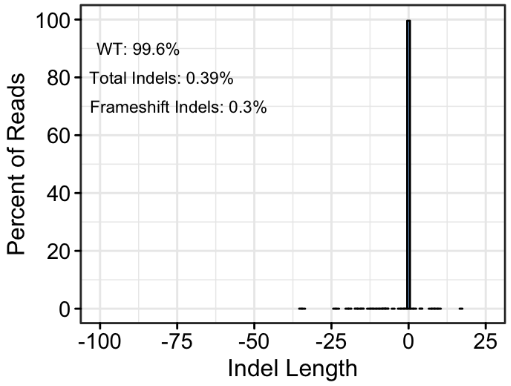


223. A 3G B2M Ex.1 SD + BE4 RNP

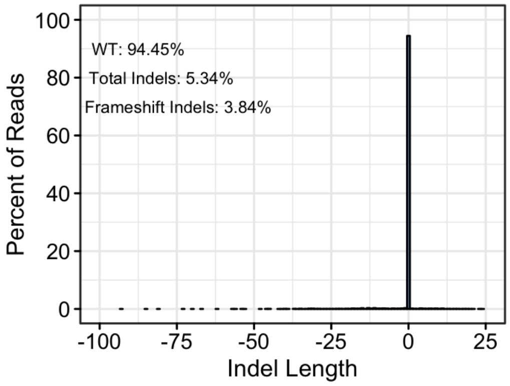


224. B 3G B2M Ex.1 SD + BE4 RNP

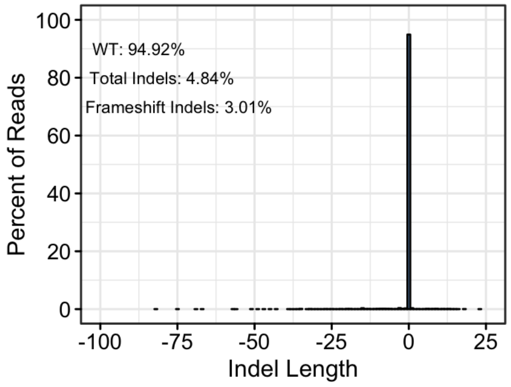


225. A 3G TRAC Ex.3 SA + Pulse

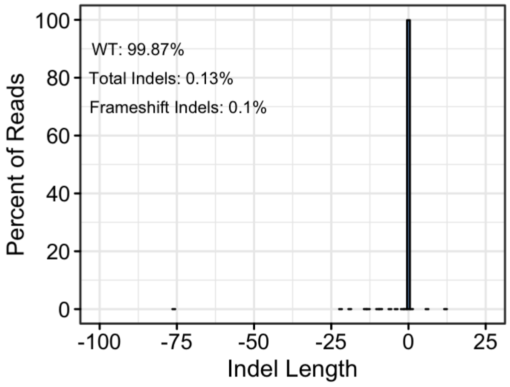


226. B 3G TRAC Ex.3 SA + Pulse

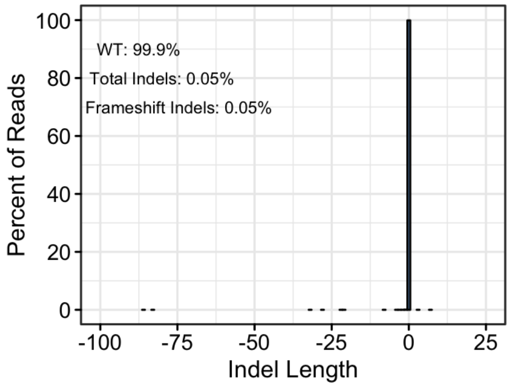


227. A 3G TRAC Ex.3 SA + BE4 RNP

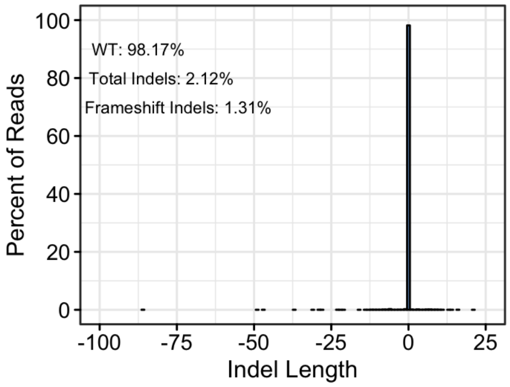

228. B 3G TRAC Ex.3 SA + BE4 RNP

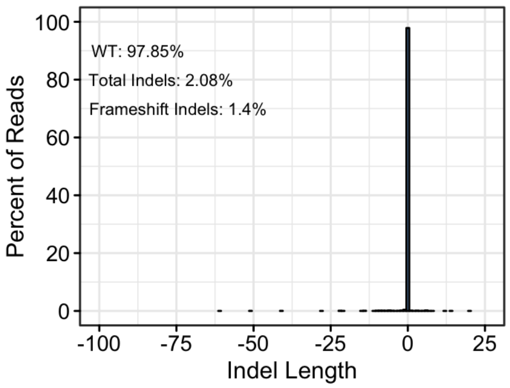


229. A 3G TRAC Ex.3 SA + Pulse

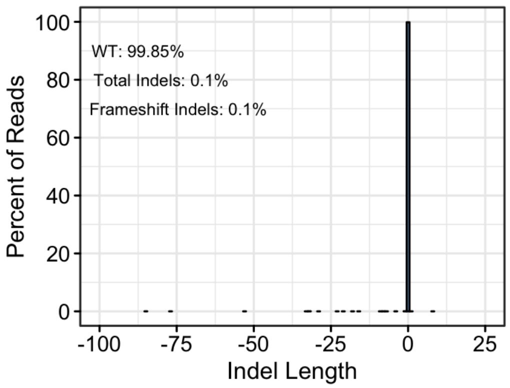


230. B 3G TRAC Ex.3 SA + Pulse

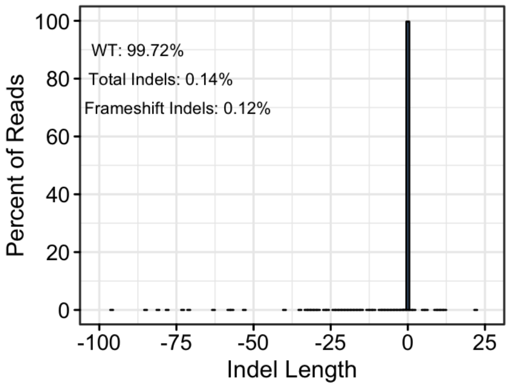


231. A 3G TRAC Ex.3 SA + BE4 RNP

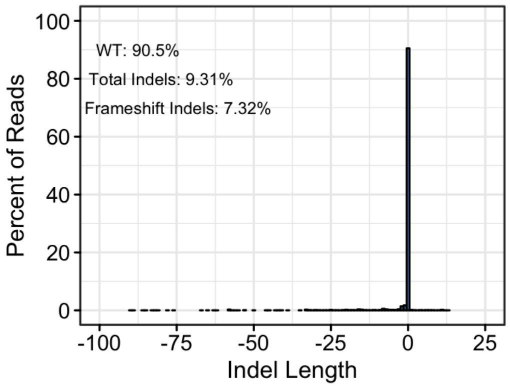


232. B 3G TRAC Ex.3 SA + BE4 RNP

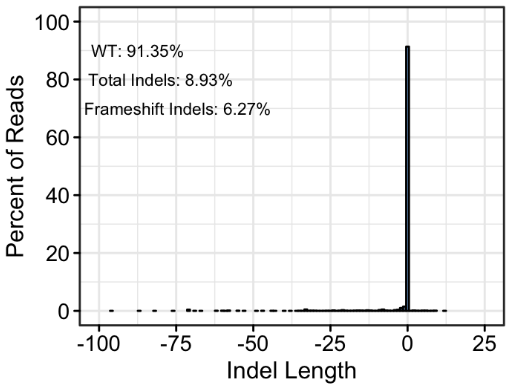


233. A B2M Ex.1 SD OnT + Pulse

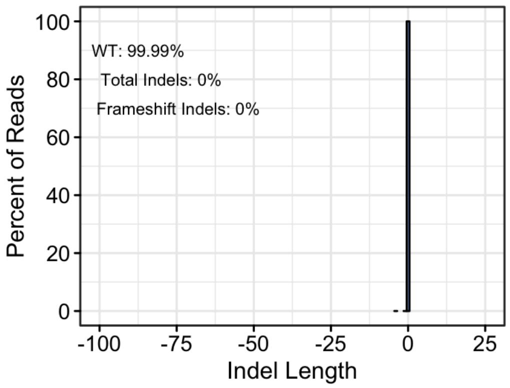


234. A B2M Ex.1 SD OT1 + Pulse

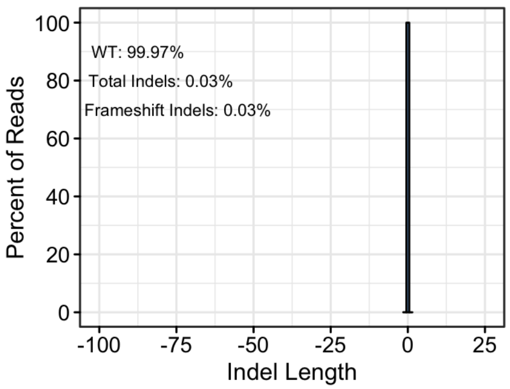


235. A B2M Ex.1 SD OT2 + Pulse

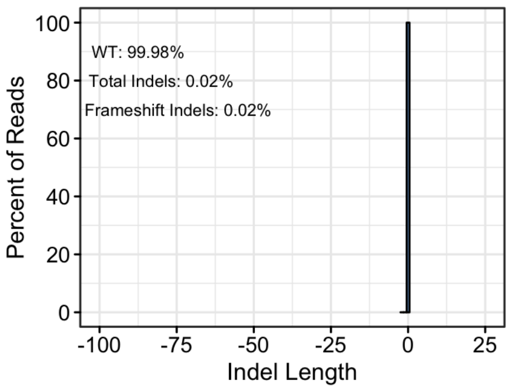

236. A B2M Ex.1 SD OT3 + Pulse

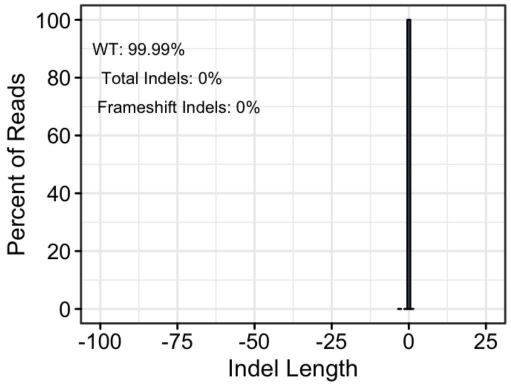


237. A B2M Ex.1 SD OT4 + Pulse

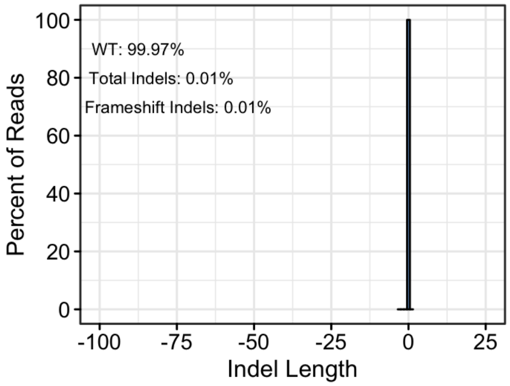


238. A B2M Ex.1 SD OT5 + Pulse

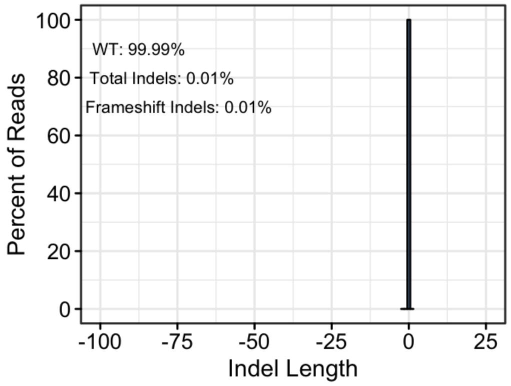


239. A B2M Ex.1 SD OT6 + Pulse

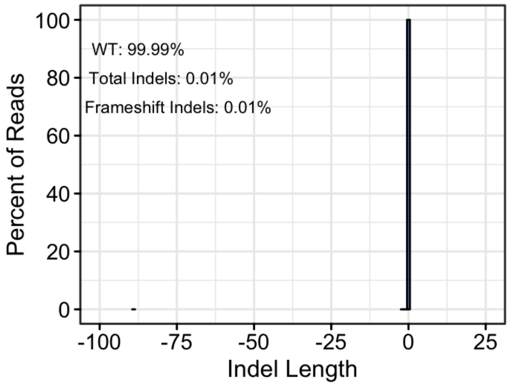


240. A B2M Ex.1 SD OT7 + Pulse

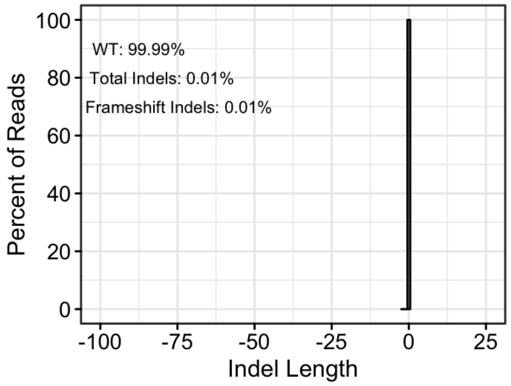


241. A B2M Ex.1 SD OT8 + Pulse

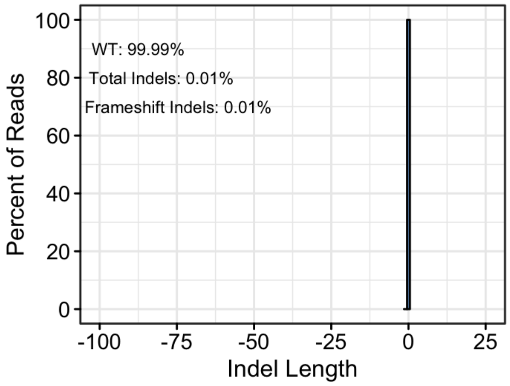


242. A B2M Ex.1 SD OT9 + Pulse

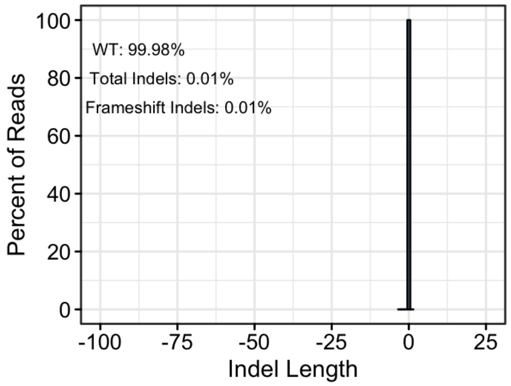


243. A B2M Ex.1 SD OT10 + Pulse

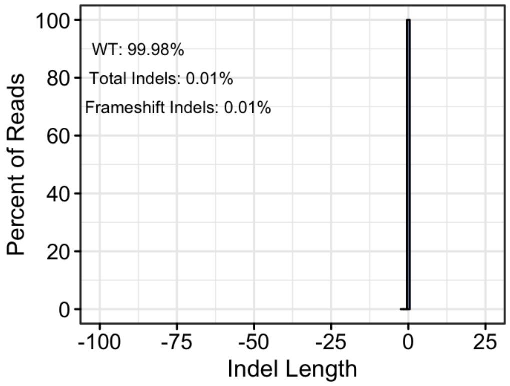

244. A B2M Ex.1 SD OnT + Cas9 mRNA

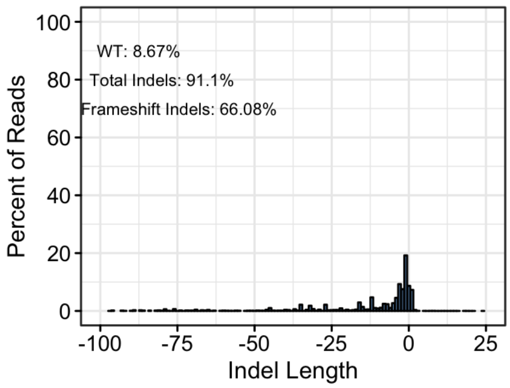


245. A B2M Ex.1 SD OT1 + Cas9 mRNA

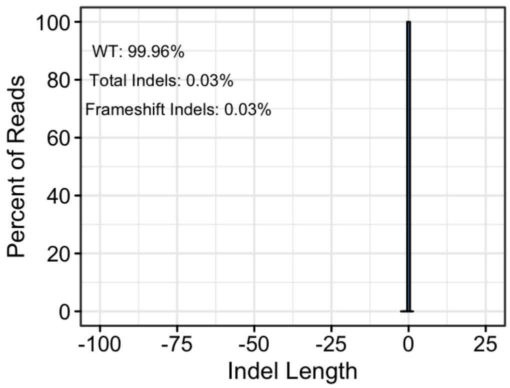


246. A B2M Ex.1 SD OT2 + Cas9 mRNA

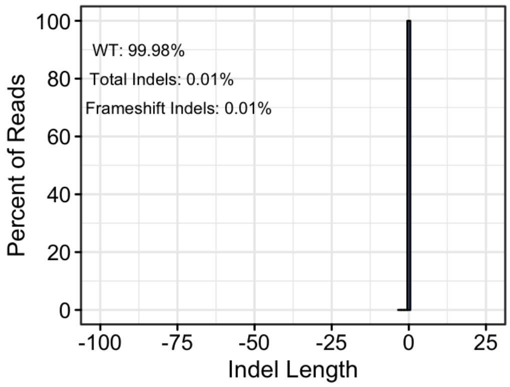


247. A B2M Ex.1 SD OT3 + Cas9 mRNA

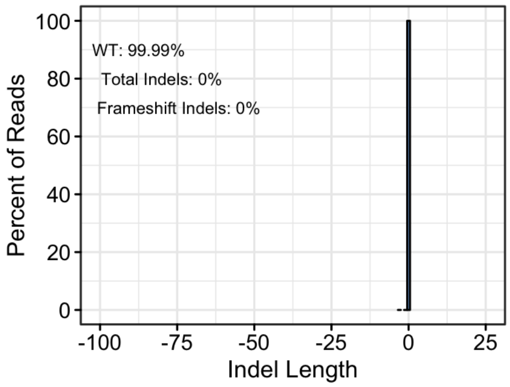


248. A B2M Ex.1 SD OT4 + Cas9 mRNA

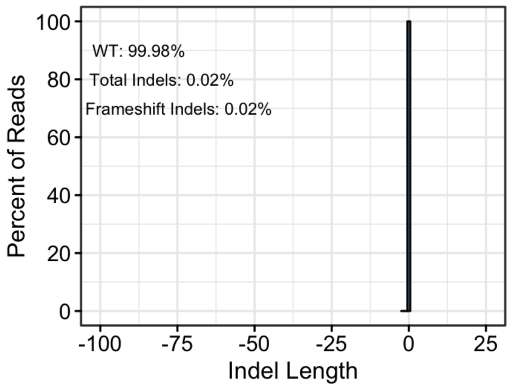


249. A B2M Ex.1 SD OT5 + Cas9 mRNA

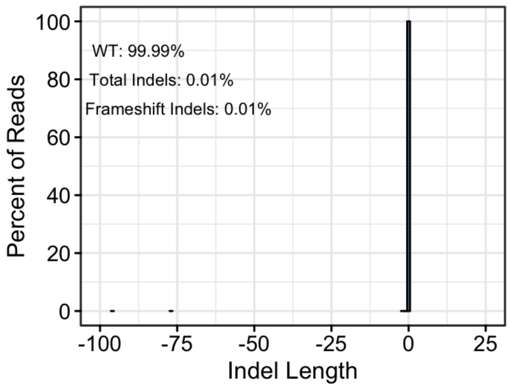


250. A B2M Ex.1 SD OT6 + Cas9 mRNA

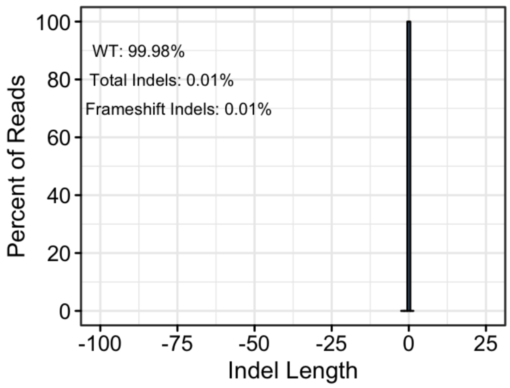


251. A B2M Ex.1 SD OT7 + Cas9 mRNA

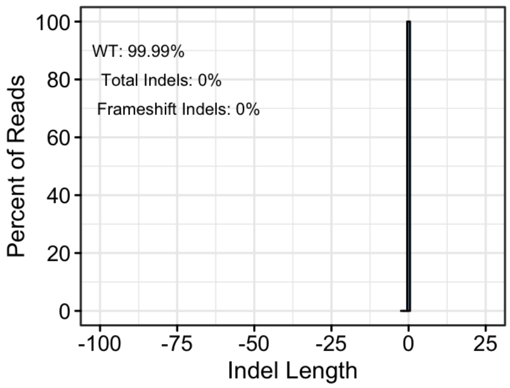

252. A B2M Ex.1 SD OT8 + Cas9 mRNA

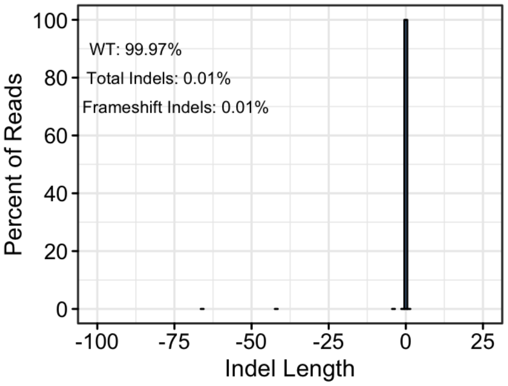


253. A B2M Ex.1 SD OT9 + Cas9 mRNA

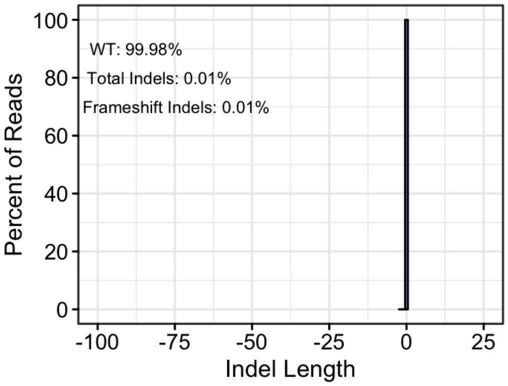


254. A B2M Ex.1 SD OT10 + Cas9 mRNA

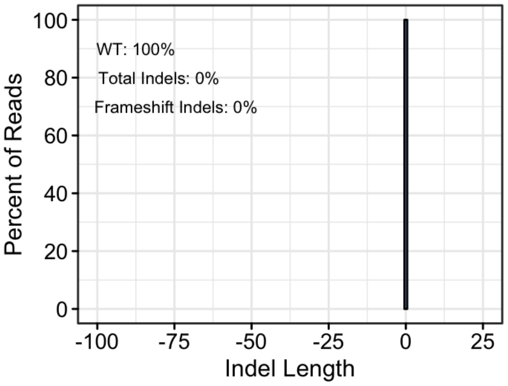


255. A B2M Ex.1 SD OnT + BE4 mRNA

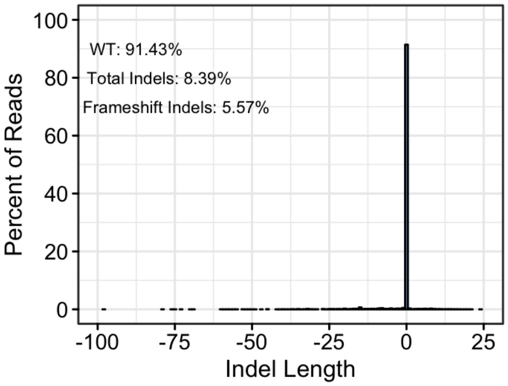


256. A B2M Ex.1 SD OT1 + BE4 mRNA

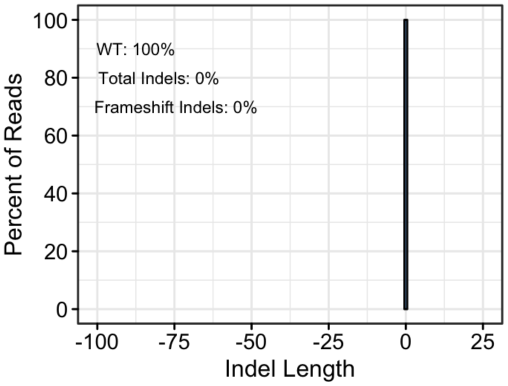


257. A B2M Ex.1 SD OT2 + BE4 mRNA

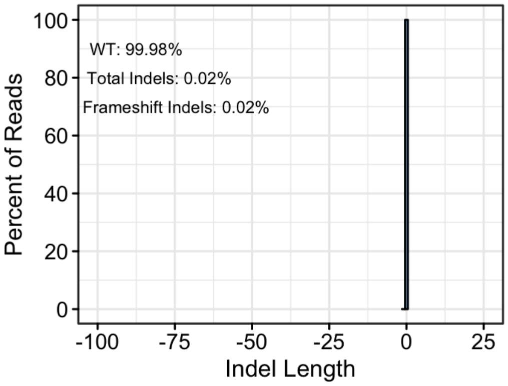


258. A B2M Ex.1 SD OT3 + BE4 mRNA

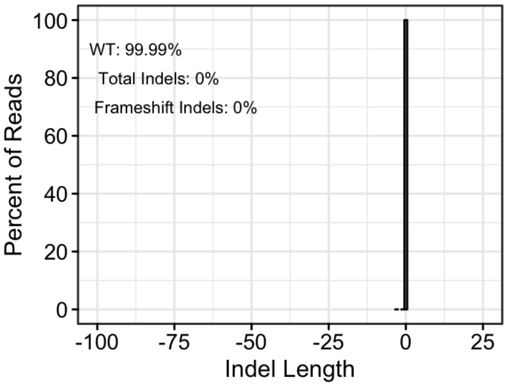


259. A B2M Ex.1 SD OT4 + BE4 mRNA

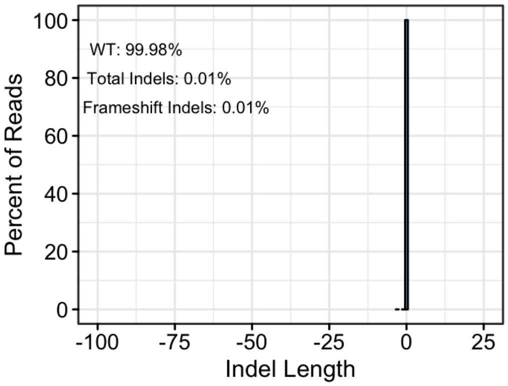

260. A B2M Ex.1 SD OT5 + BE4 mRNA

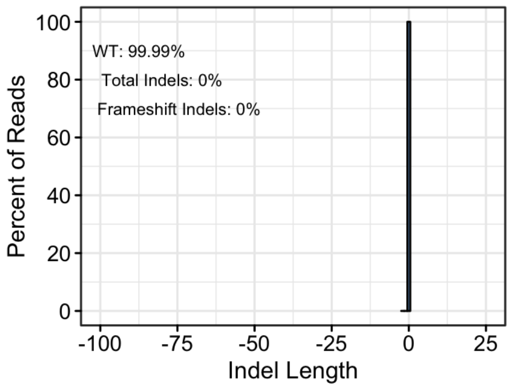


261. A B2M Ex.1 SD OT6 + BE4 mRNA

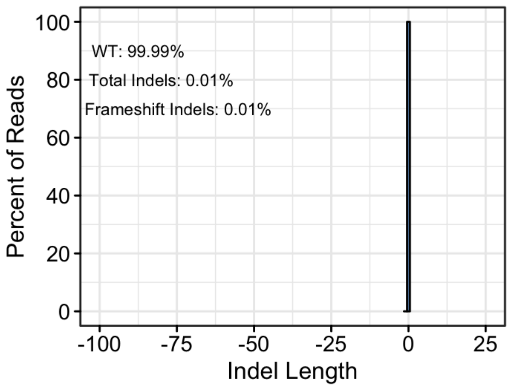


262. A B2M Ex.1 SD OT7 + BE4 mRNA

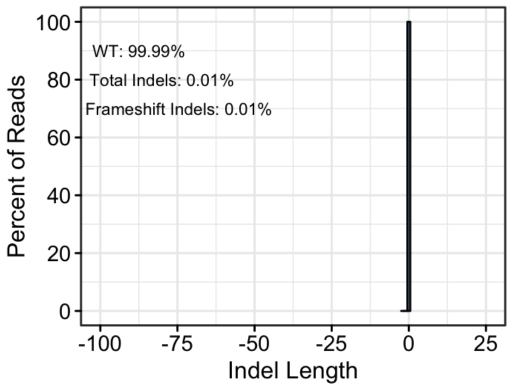


263. A B2M Ex.1 SD OT8 + BE4 mRNA

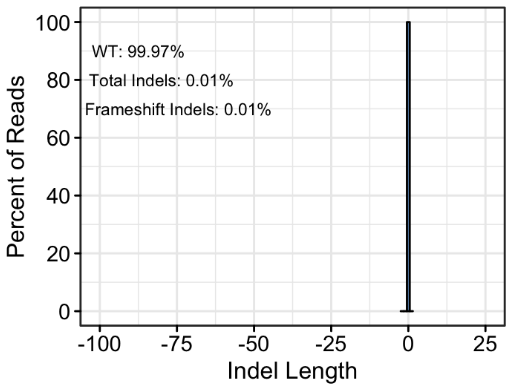


264. A B2M Ex.1 SD OT9 + BE4 mRNA

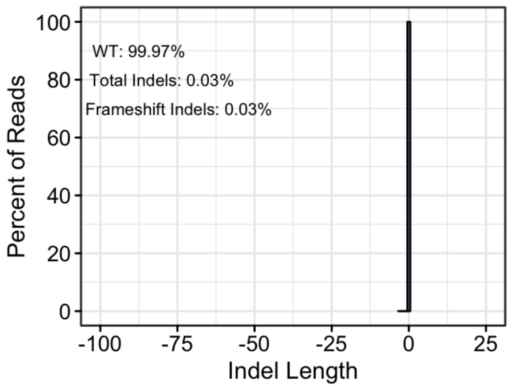


265. A B2M Ex.1 SD OT10 + BE4 mRNA

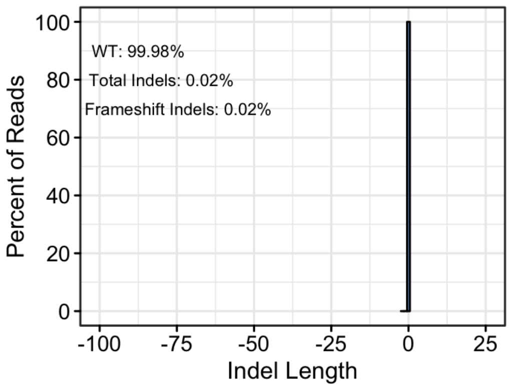


266. A TRAC Ex.3 SA OnT + Pulse

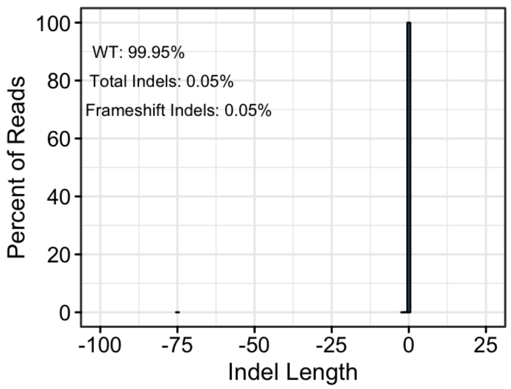


267. A TRAC Ex.3 SA OT1 + Pulse

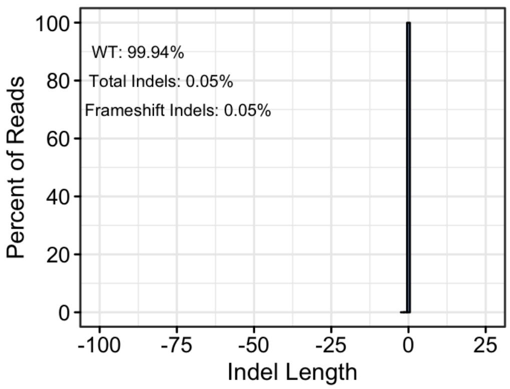

268. A TRAC Ex.3 SA OT2 + Pulse

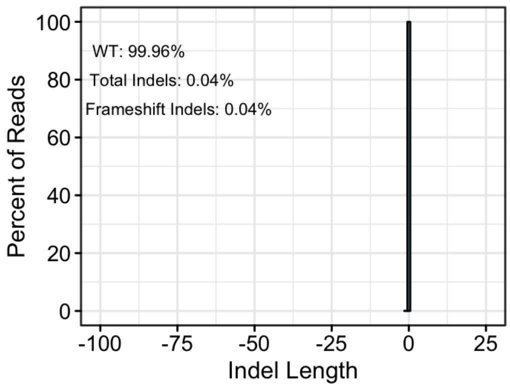


269. A TRAC Ex.3 SA OT3 + Pulse

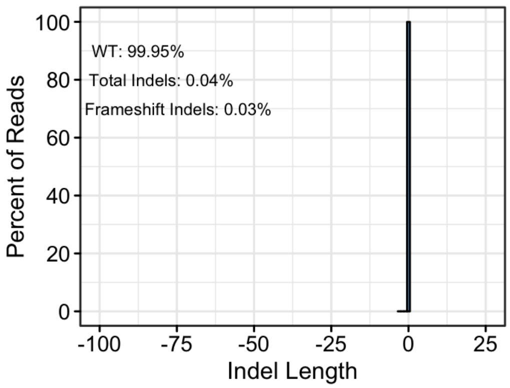


270. A TRAC Ex.3 SA OT4 + Pulse

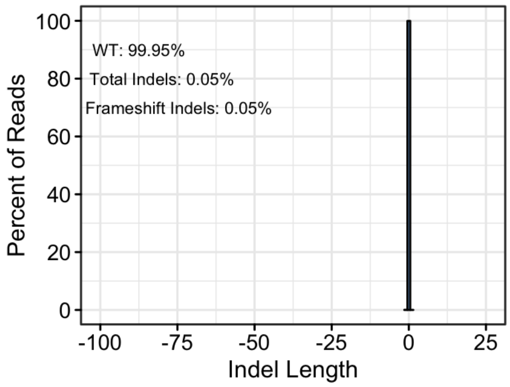


271. A TRAC Ex.3 SA OT5 + Pulse

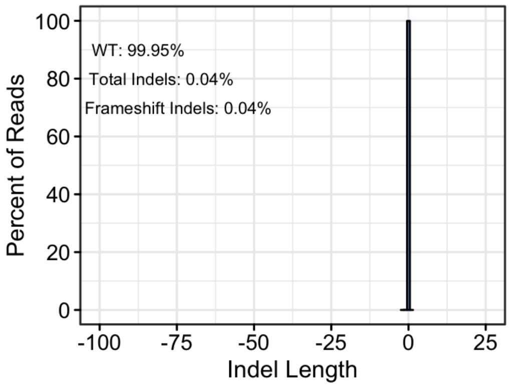


272. A TRAC Ex.3 SA OT6 + Pulse

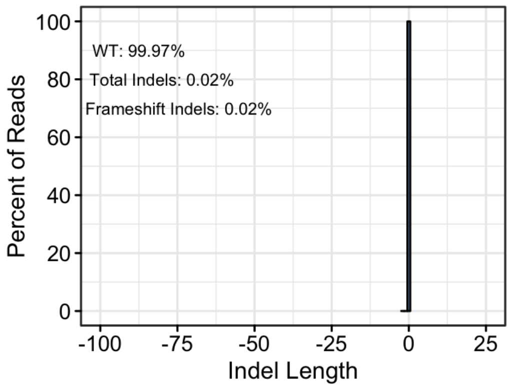


273. A TRAC Ex.3 SA OT7 + Pulse

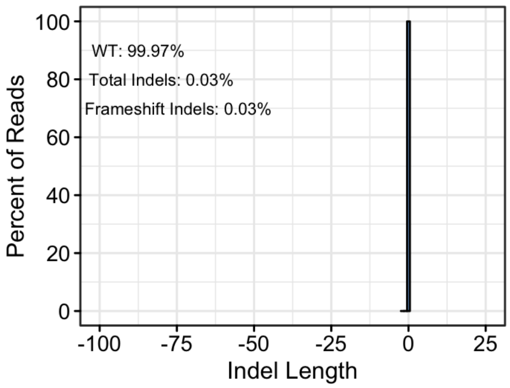


274. A TRAC Ex.3 SA OT8 + Pulse

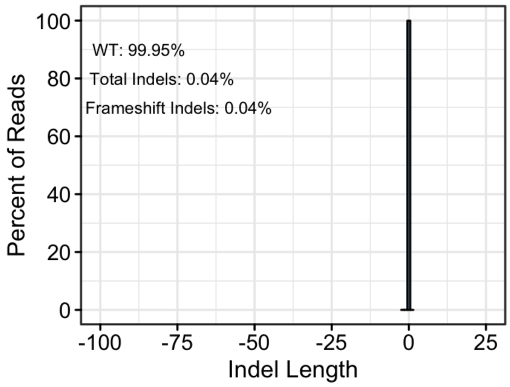


275. A TRAC Ex.3 SA OT9 + Pulse

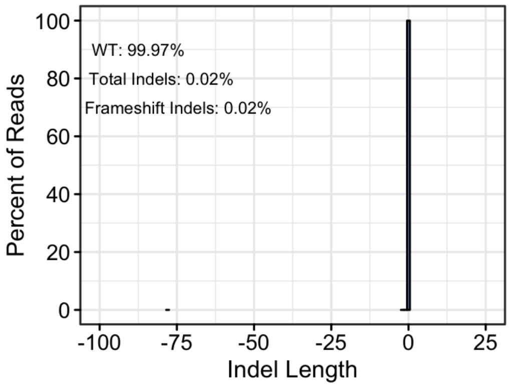

276. A TRAC Ex.3 SA OT10 + Pulse

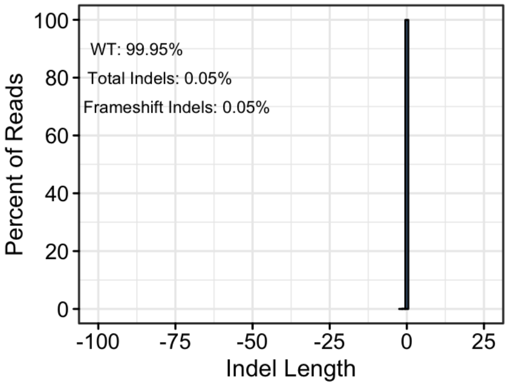


277. A TRAC Ex.3 SA OnT + Cas9 mRNA

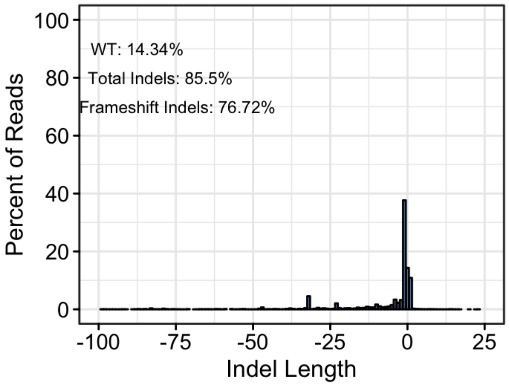


278. A TRAC Ex.3 SA OT1 + Cas9 mRNA

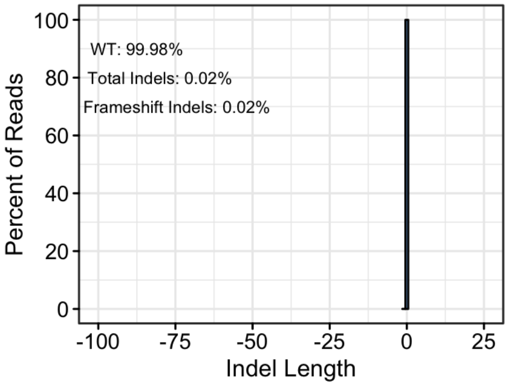


279. A TRAC Ex.3 SA OT2 + Cas9 mRNA

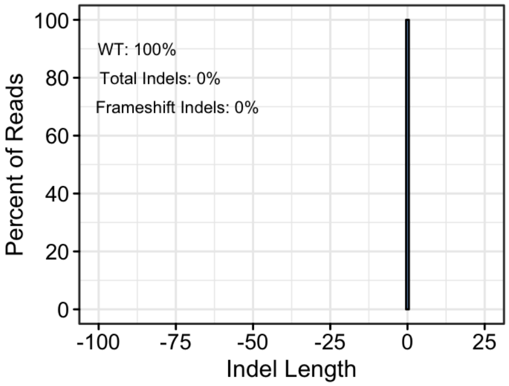


280. A TRAC Ex.3 SA OT3 + Cas9 mRNA

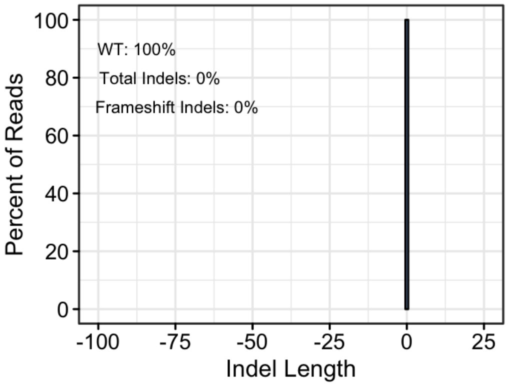


281. A TRAC Ex.3 SA OT4 + Cas9 mRNA

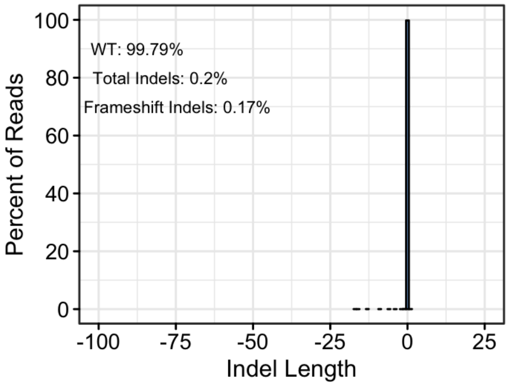


282. A TRAC Ex.3 SA OT5 + Cas9 mRNA

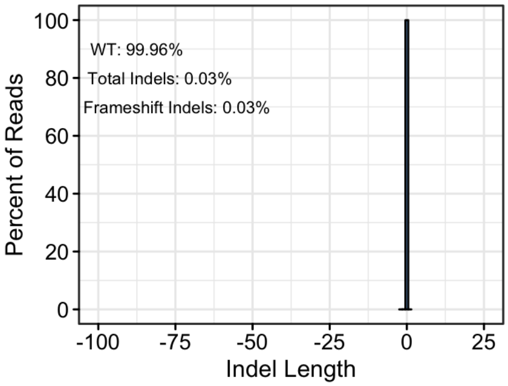


283. A TRAC Ex.3 SA OT6 + Cas9 mRNA

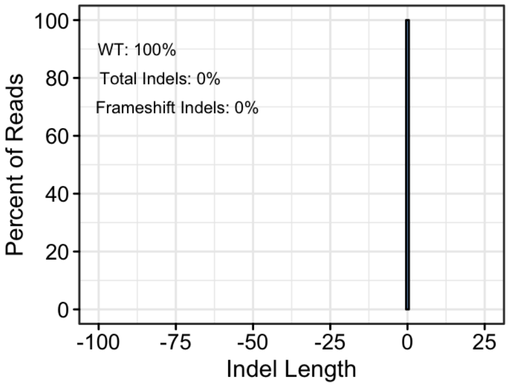

284. A TRAC Ex.3 SA OT7 + Cas9 mRNA

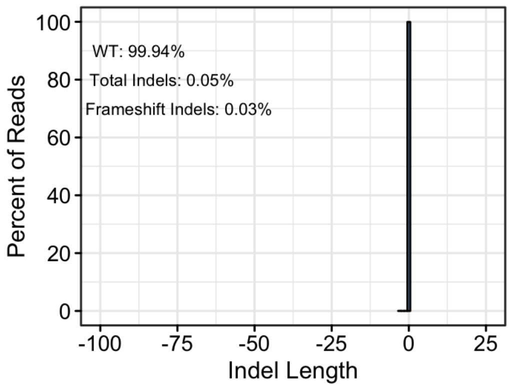


285. A TRAC Ex.3 SA OT8 + Cas9 mRNA

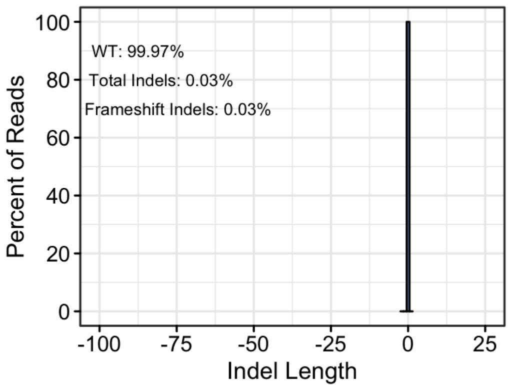


286. A TRAC Ex.3 SA OT9 + Cas9 mRNA

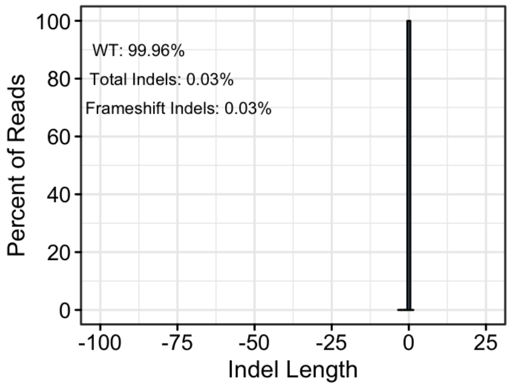


287. A TRAC Ex.3 SA OT10 + Cas9 mRNA

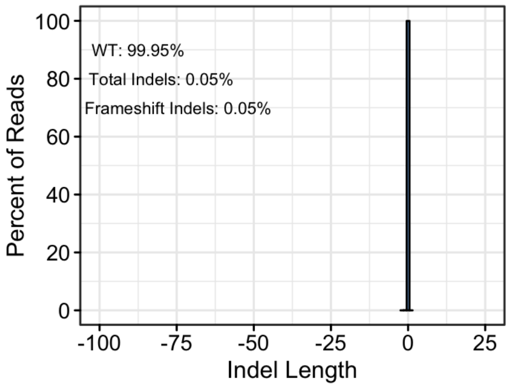


288. A TRAC Ex.3 SA OnT + BE4 mRNA

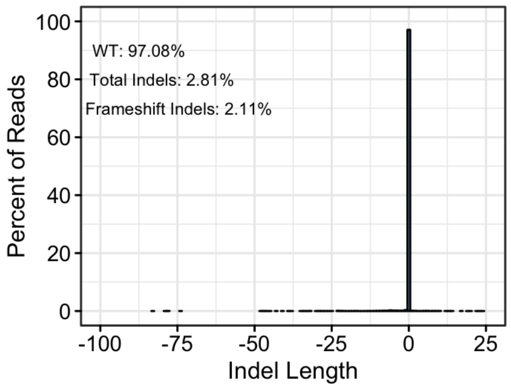


289. A TRAC Ex.3 SA OT1 + BE4 mRNA

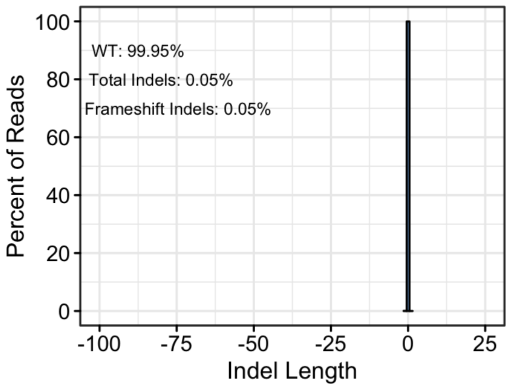


290. A TRAC Ex.3 SA OT2 + BE4 mRNA

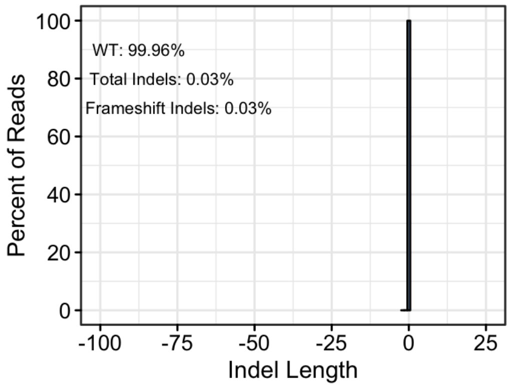


291. A TRAC Ex.3 SA OT3 + BE4 mRNA

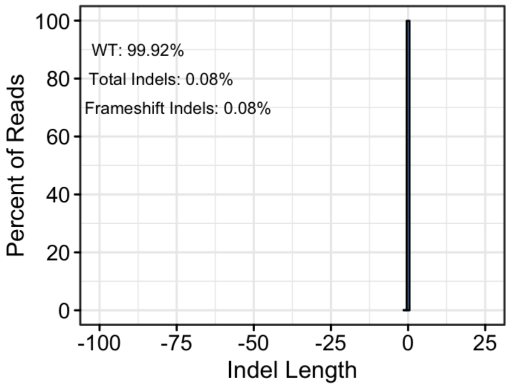

292. A TRAC Ex.3 SA OT4 + BE4 mRNA

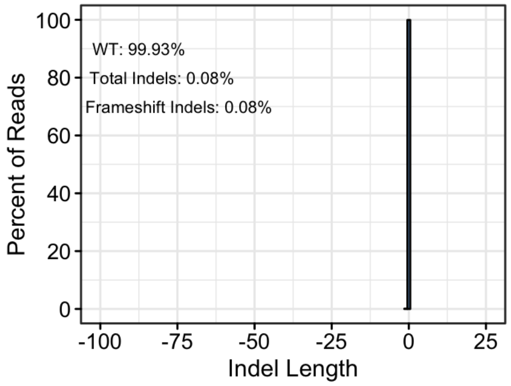


293. A TRAC Ex.3 SA OT5 + BE4 mRNA

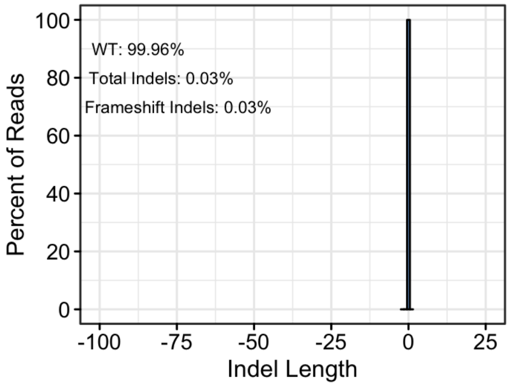


294. A TRAC Ex.3 SA OT6 + BE4 mRNA

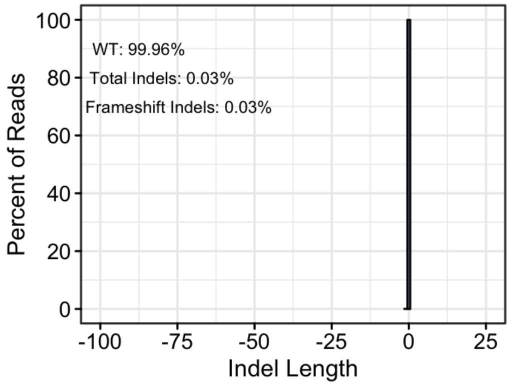


295. A TRAC Ex.3 SA OT7 + BE4 mRNA

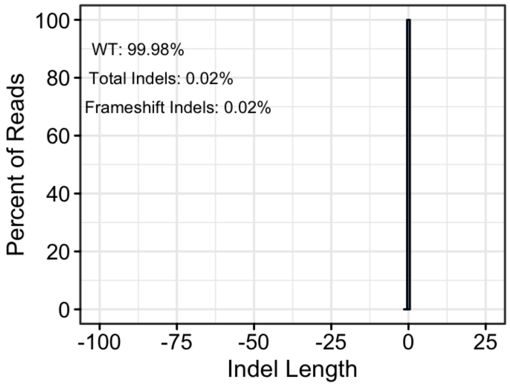


296. A TRAC Ex.3 SA OT8 + BE4 mRNA

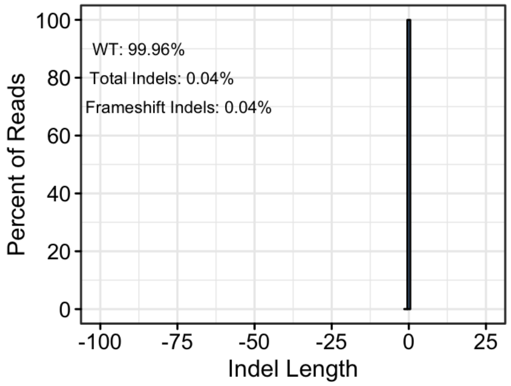


297. A TRAC Ex.3 SA OT9 + BE4 mRNA

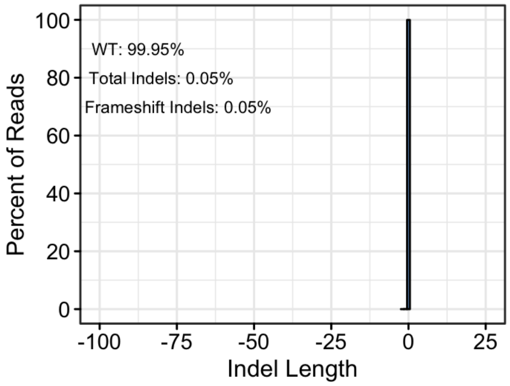


298. A TRAC Ex.3 SA OT10 + BE4 mRNA

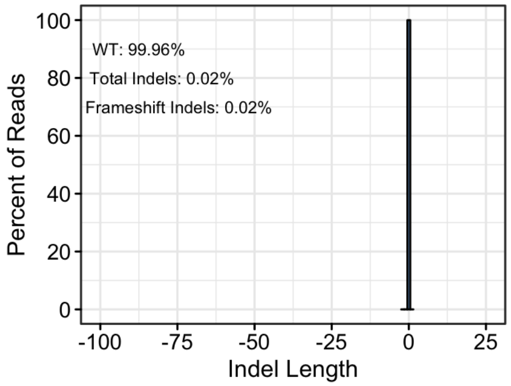


299. A PDCD1 Ex.1 SD OnT + Pulse

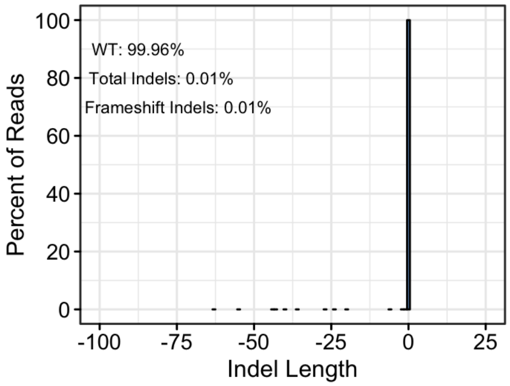

300. A PDCD1 Ex.1 SD OT1 + Pulse

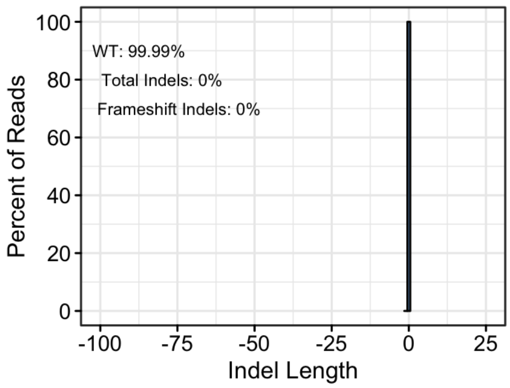


301. A PDCD1 Ex.1 SD OT2 + Pulse

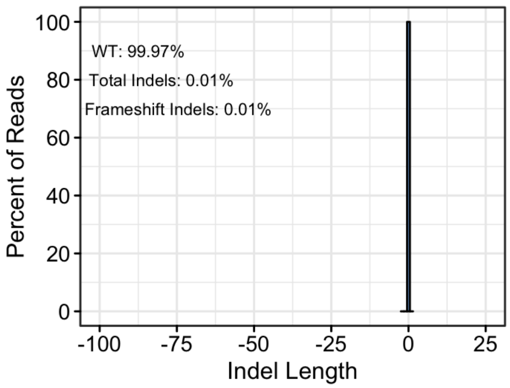


302. A PDCD1 Ex.1 SD OT3 + Pulse

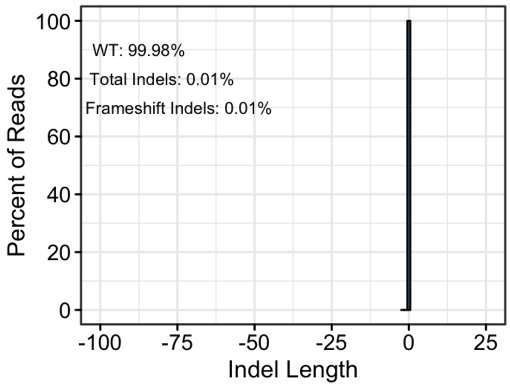


303. A PDCD1 Ex.1 SD OT4 + Pulse

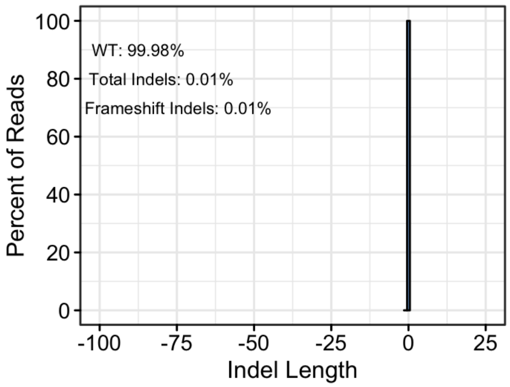


304. A PDCD1 Ex.1 SD OT5 + Pulse

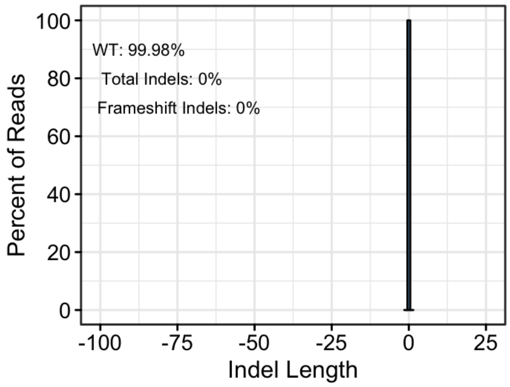


305. A PDCD1 Ex.1 SD OT6 + Pulse

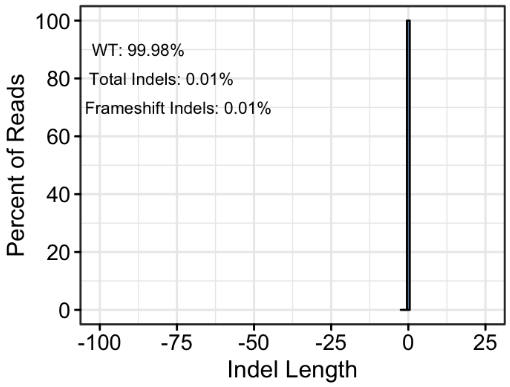


306. A PDCD1 Ex.1 SD OT7 + Pulse

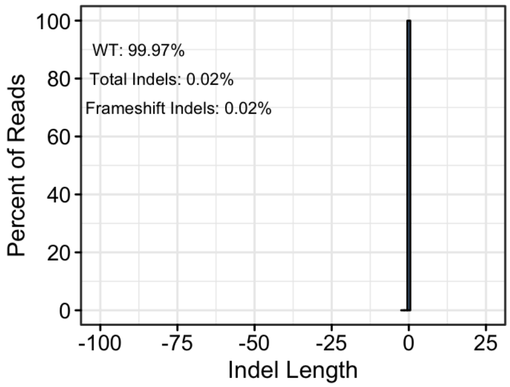


307. A PDCD1 Ex.1 SD OT8 + Pulse

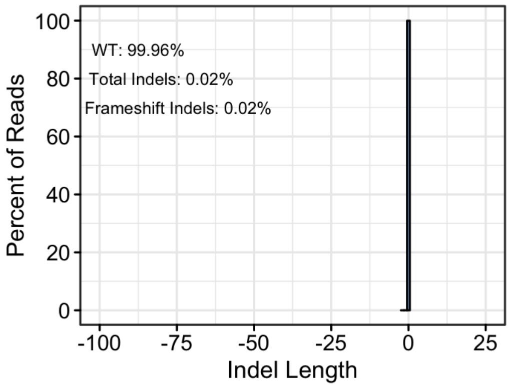

308. A PDCD1 Ex.1 SD OT9 + Pulse

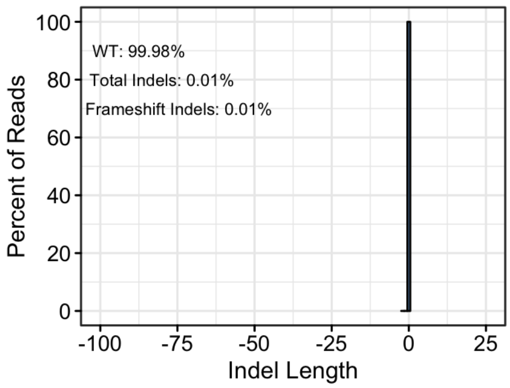


309. A PDCD1 Ex.1 SD OT10 + Pulse

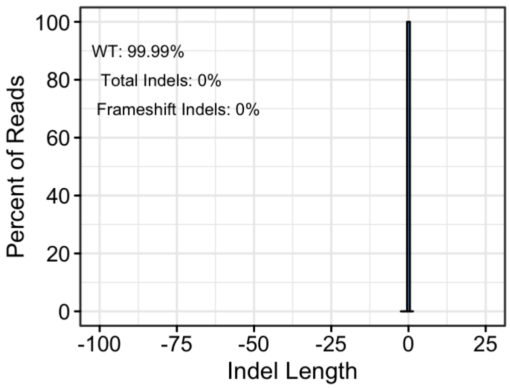


310. A PDCD1 Ex.1 SD OnT + Cas9 mRNA

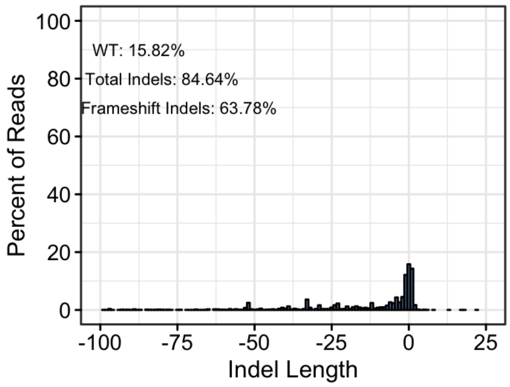


311. A PDCD1 Ex.1 SD OT1 + Cas9 mRNA

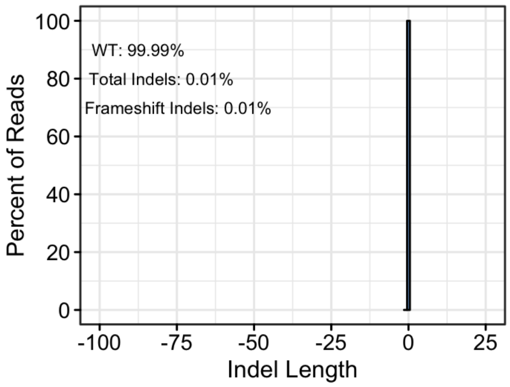


312. A PDCD1 Ex.1 SD OT2 + Cas9 mRNA

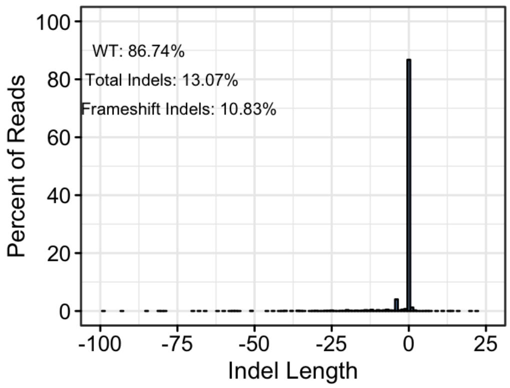


313. A PDCD1 Ex.1 SD OT3 + Cas9 mRNA

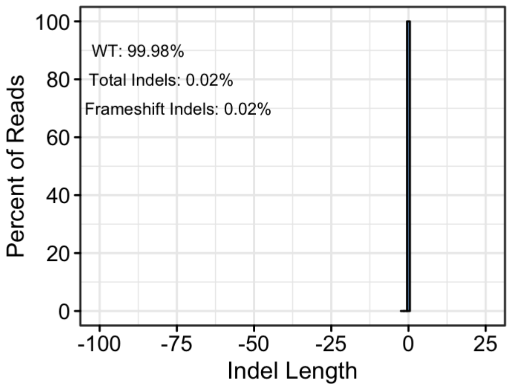


314. A PDCD1 Ex.1 SD OT4 + Cas9 mRNA

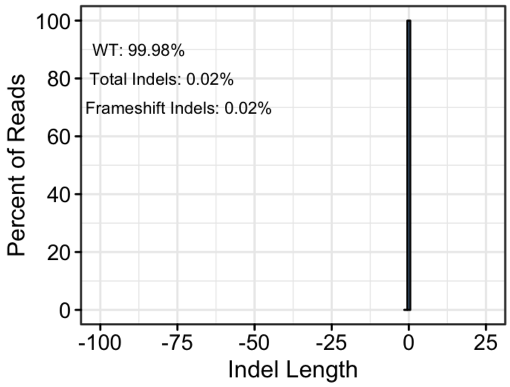


315. A PDCD1 Ex.1 SD OT5 + Cas9 mRNA

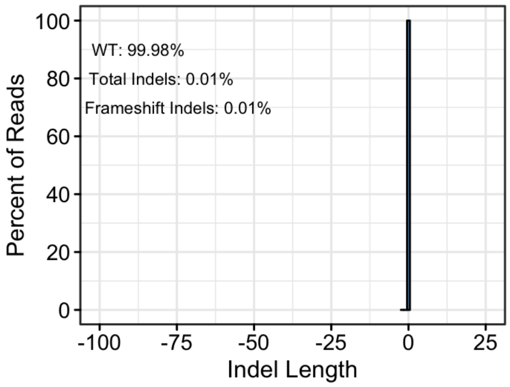

316. A PDCD1 Ex.1 SD OT6 + Cas9 mRNA

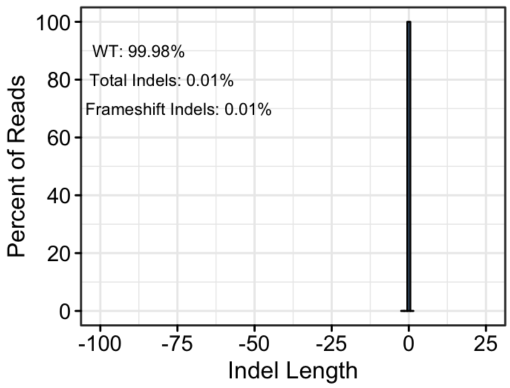


317. A PDCD1 Ex.1 SD OT7 + Cas9 mRNA

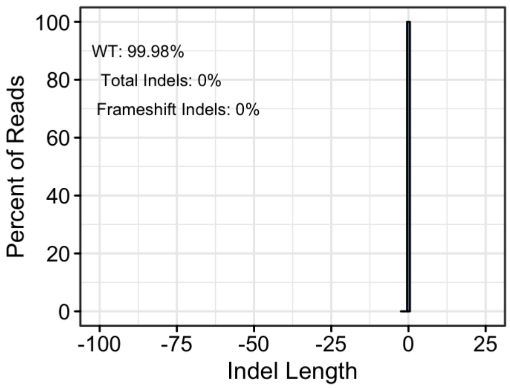


318. A PDCD1 Ex.1 SD OT8 + Cas9 mRNA

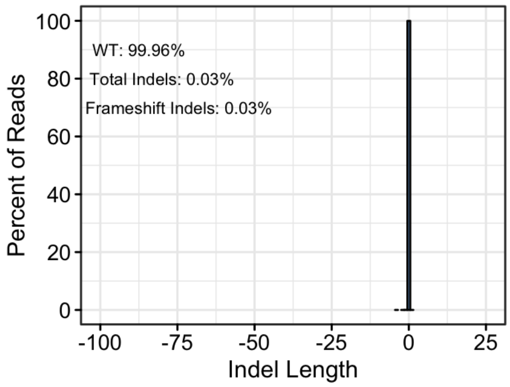


319. A PDCD1 Ex.1 SD OT9 + Cas9 mRNA

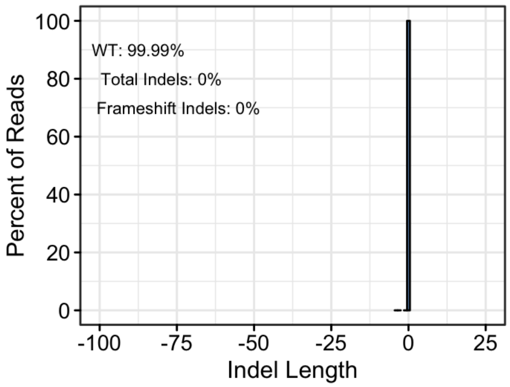


320. A PDCD1 Ex.1 SD OT10 + Cas9 mRNA

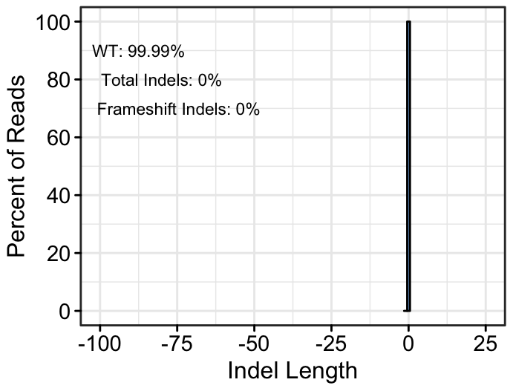


321. A PDCD1 Ex.1 SD OnT + BE4 mRNA

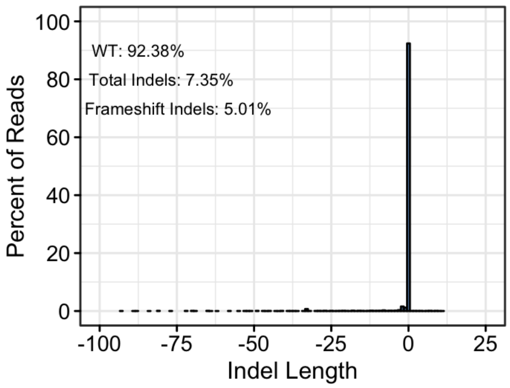


322. A PDCD1 Ex.1 SD OT1 + BE4 mRNA

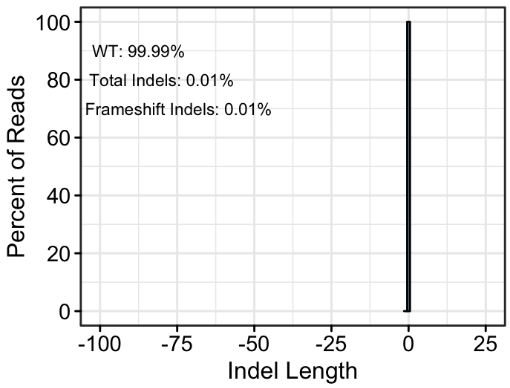


323. A PDCD1 Ex.1 SD OT2 + BE4 mRNA

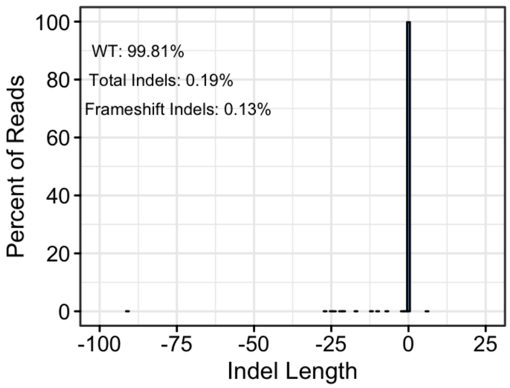

324. A PDCD1 Ex.1 SD OT3 + BE4 mRNA

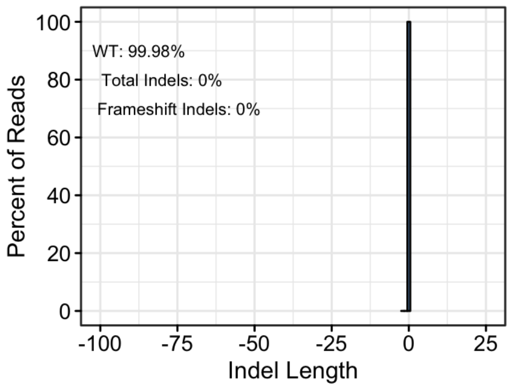


325. A PDCD1 Ex.1 SD OT4 + BE4 mRNA

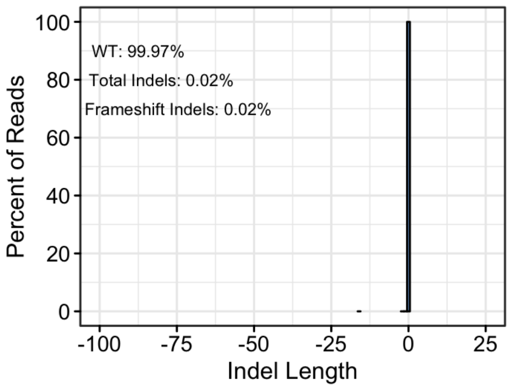


326. A PDCD1 Ex.1 SD OT5 + BE4 mRNA

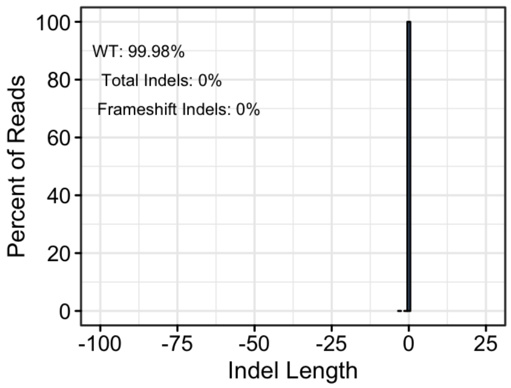


327. A PDCD1 Ex.1 SD OT6 + BE4 mRNA

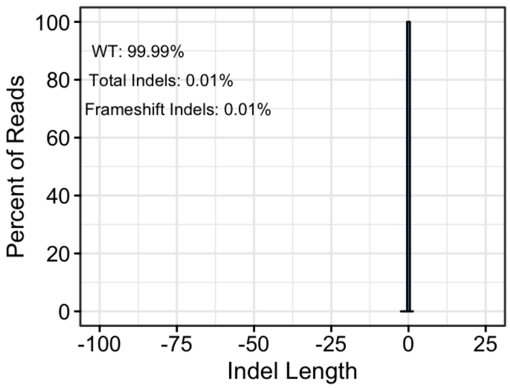


328. A PDCD1 Ex.1 SD OT7 + BE4 mRNA

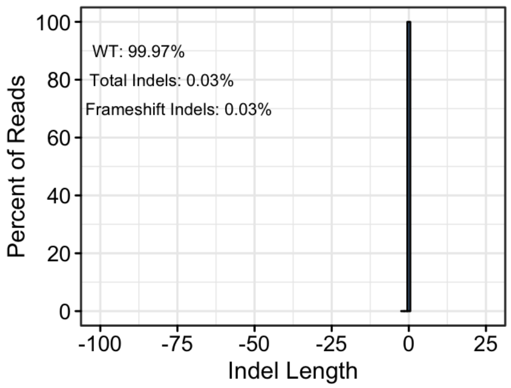


329. A PDCD1 Ex.1 SD OT8 + BE4 mRNA

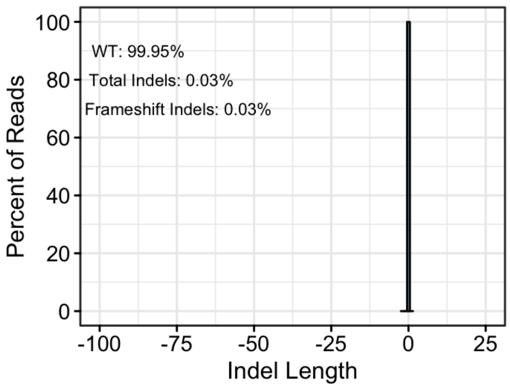


330. A PDCD1 Ex.1 SD OT9 + BE4 mRNA

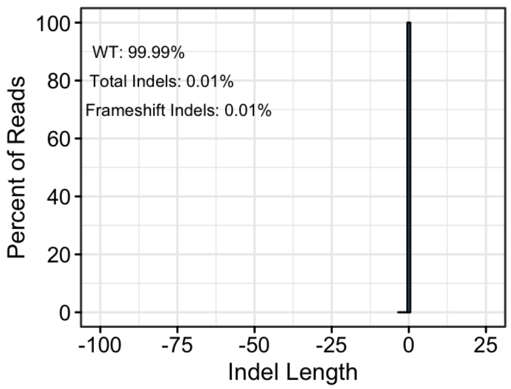


331. A PDCD1 Ex.1 SD OT10 + BE4 mRNA

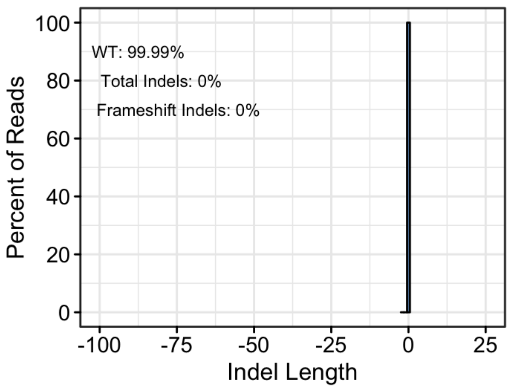

Supplement: Supplementary file 3 — Dataset 3 [file 41467_2019_13007_MOESM3_ESM.pdf]
