# Supplementary material for: Highly efficient multiplex human T cell engineering without double-strand breaks using Cas9 base editors
Source: Nat Commun. 2019 Nov 19;10:5222. doi: 10.1038/s41467-019-13007-6 (PMC6864045; doi:10.1038/s41467-019-13007-6)

Highly efficient multiplex human T cell engineering without double-strand breaks using Cas9 base editors

Appendix II - Bar Plots of Next Generation Sequencing

1. A PDCD1 Ex.1 SD + BE3 unconc. mRNA 1.5 µg

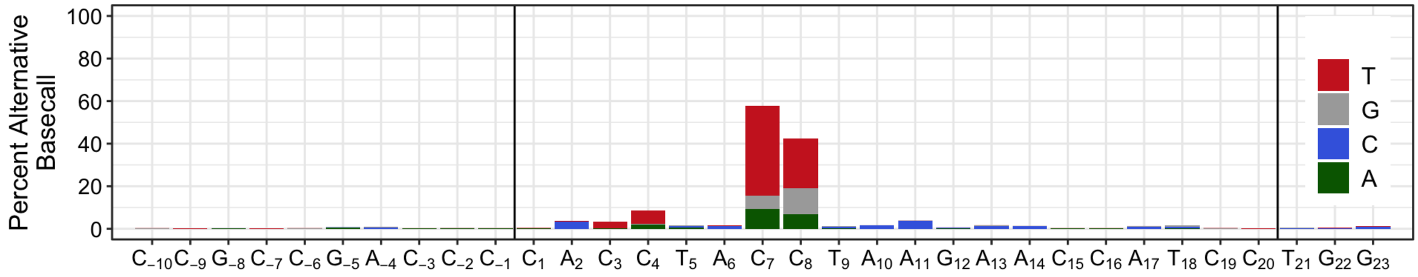

2. B PDCD1 Ex.1 SD + BE3 unconc. mRNA 1.5 µg

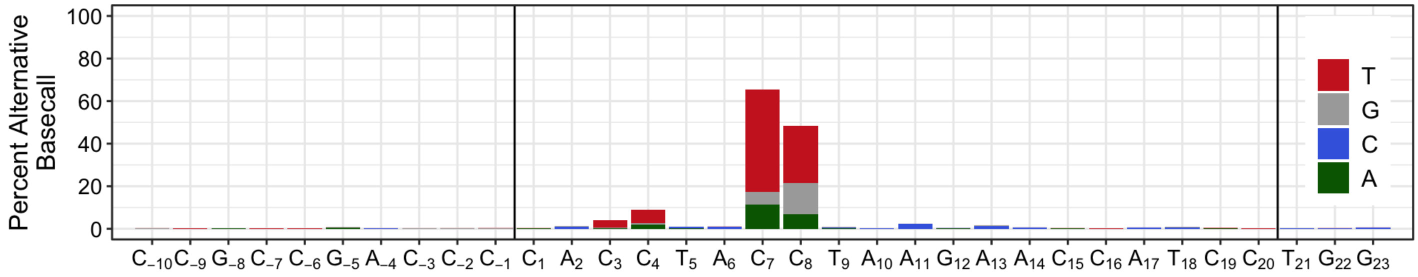

3. C PDCD1 Ex.1 SD + BE3 unconc. mRNA 1.5 µg

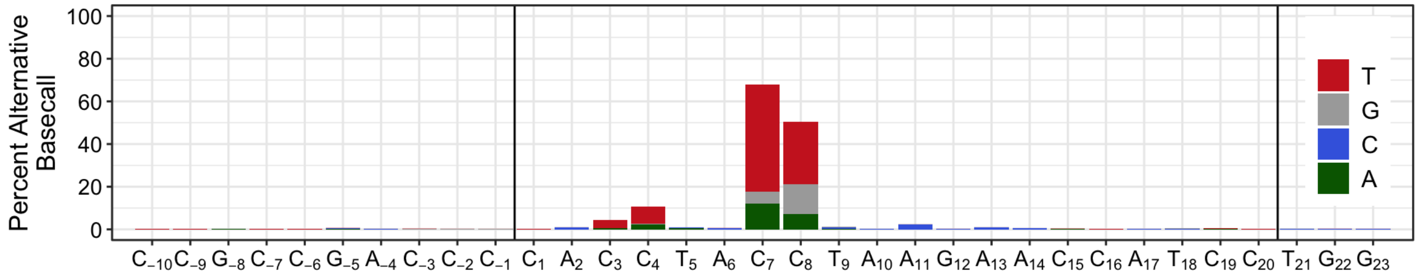

4. A PDCD1 Ex.1 SD + BE4 unconc. mRNA 1.5 µg

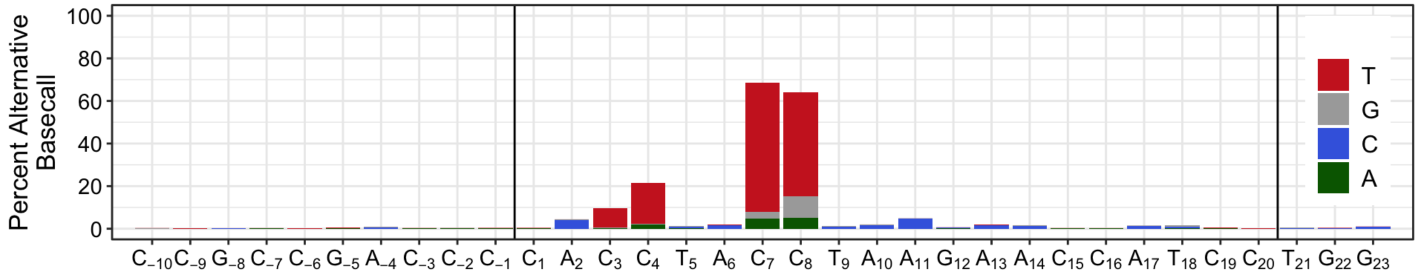

5. B PDCD1 Ex.1 SD + BE4 unconc. mRNA 1.5 µg

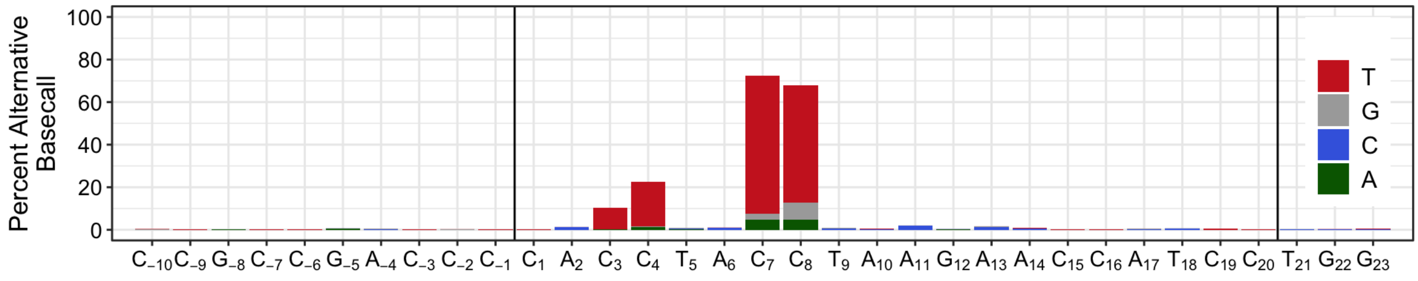

6. C PDCD1 Ex.1 SD + BE4 unconc. mRNA 1.5 µg

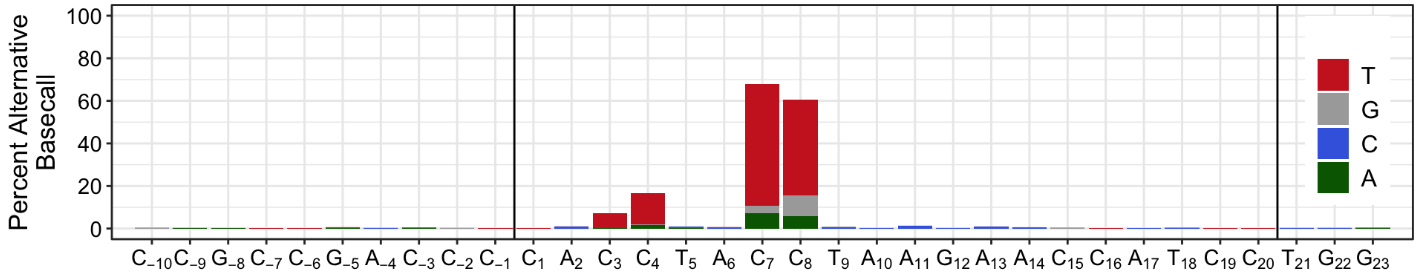

7. D PDCD1 Ex.1 SD + Pulse

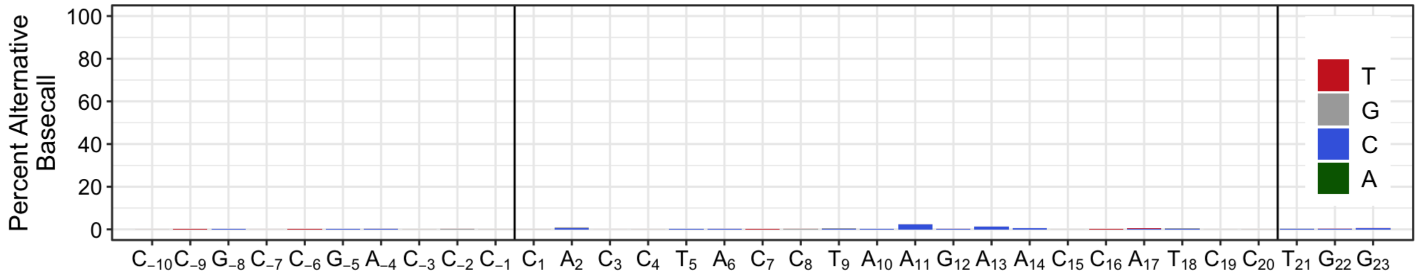

8. A PDCD1 Ex.1 SD + Pulse

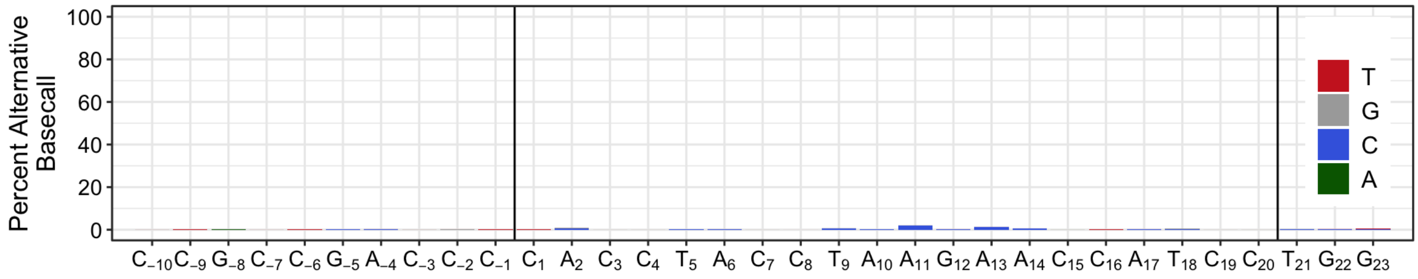

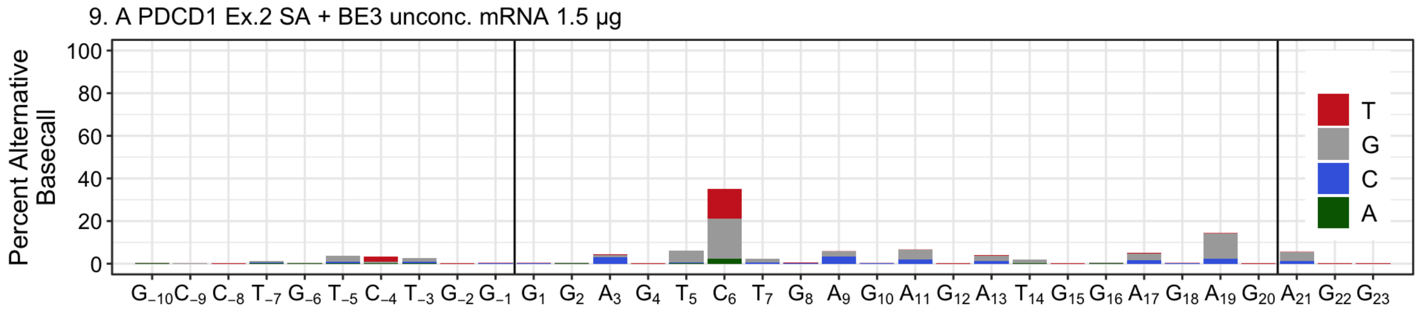
9. A PDCD1 Ex.2 SA + BE3 unconc. mRNA 1.5 µg
Percent Alternative Basecall
T
G
C
A

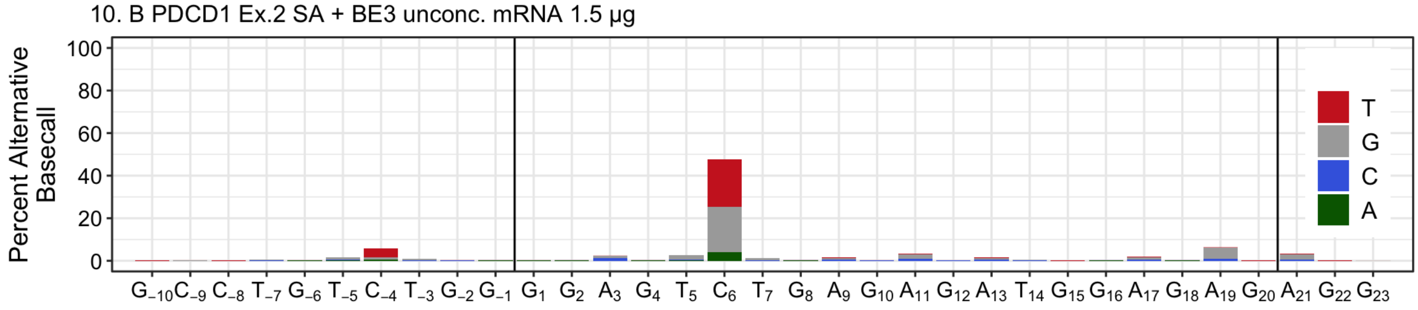
10. B PDCD1 Ex.2 SA + BE3 unconc. mRNA 1.5 µg
Percent Alternative Basecall
T
G
C
A

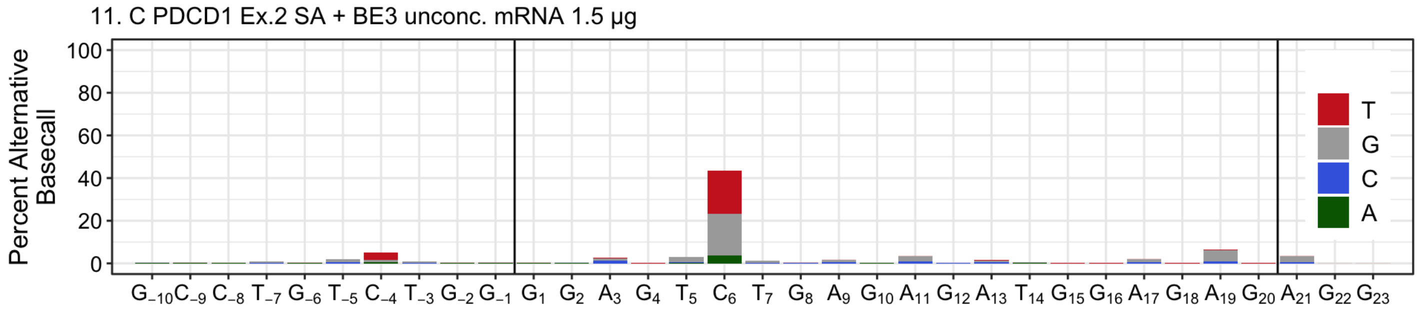
11. C PDCD1 Ex.2 SA + BE3 unconc. mRNA 1.5 µg
Percent Alternative Basecall
T
G
C
A

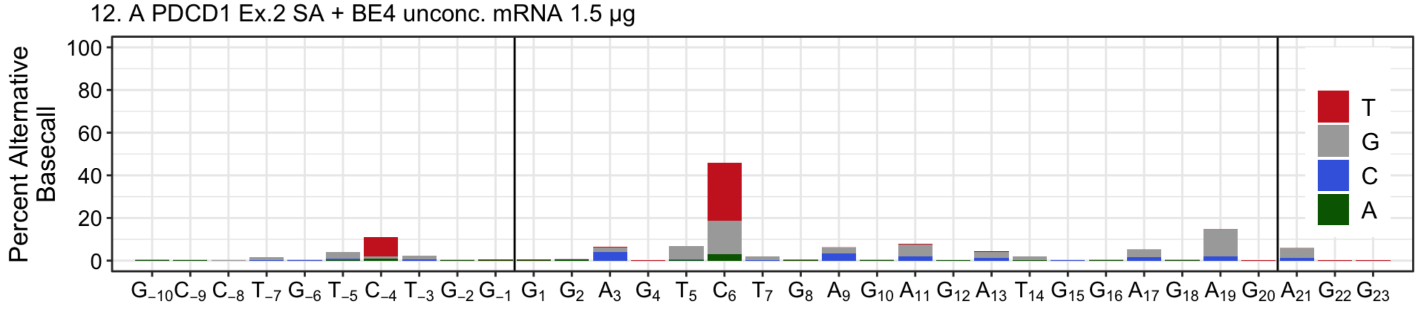
12. A PDCD1 Ex.2 SA + BE4 unconc. mRNA 1.5 µg
Percent Alternative Basecall
T
G
C
A

13. B PDCD1 Ex.2 SA + BE4 unconc. mRNA 1.5 µg

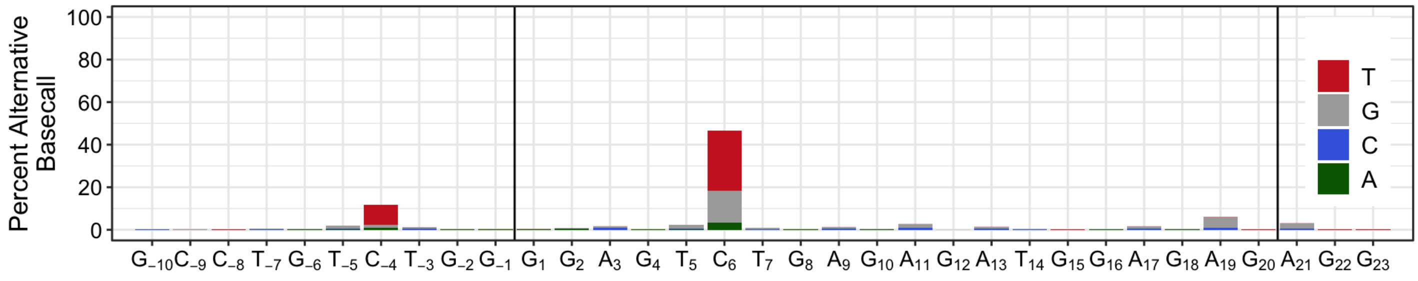

14. C PDCD1 Ex.2 SA + BE4 unconc. mRNA 1.5 µg

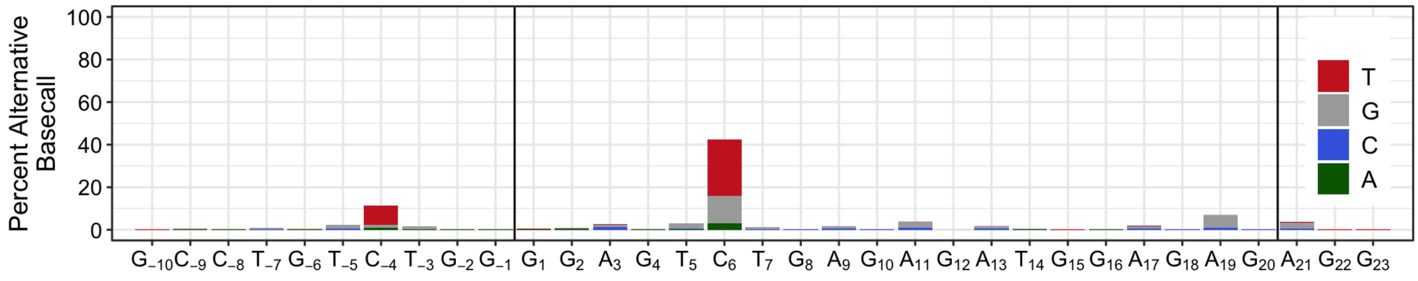

15. D PDCD1 Ex.2 SA + Pulse

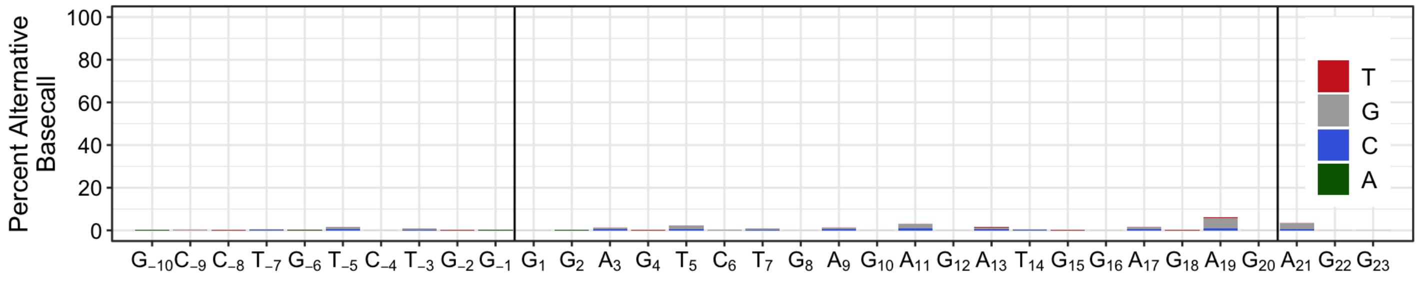

16. A PDCD1 Ex.2 SA + Pulse

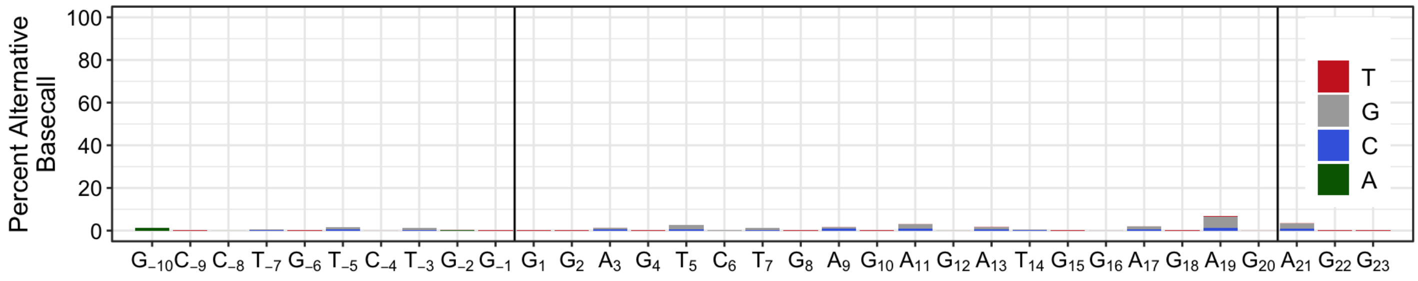

17. A PDCD1 Ex.3 SA + BE3 unconc. mRNA 1.5 µg

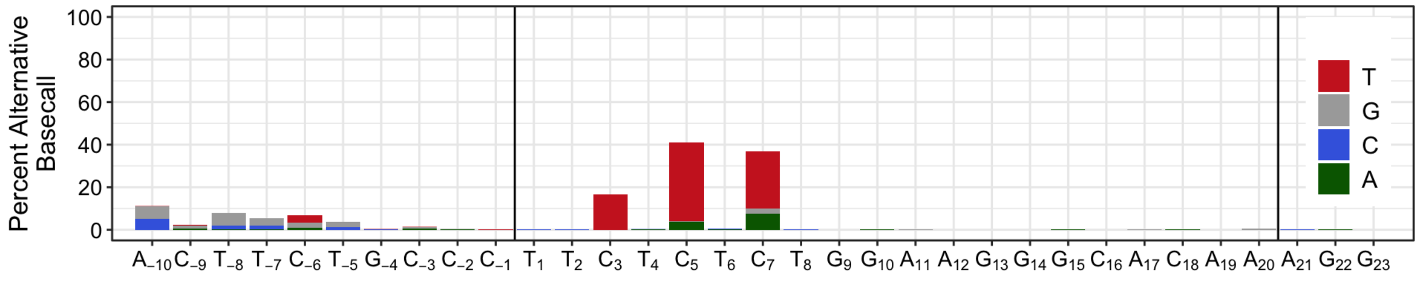

18. B PDCD1 Ex.3 SA + BE3 unconc. mRNA 1.5 µg

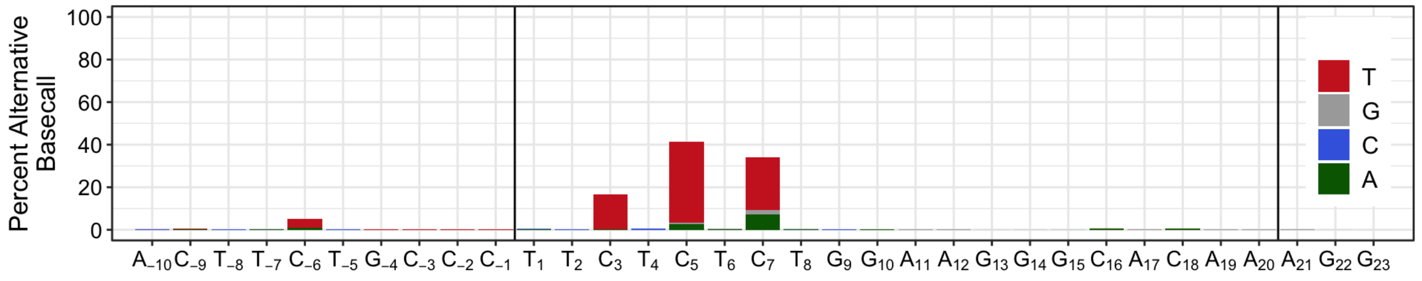

19. C PDCD1 Ex.3 SA + BE3 unconc. mRNA 1.5 µg

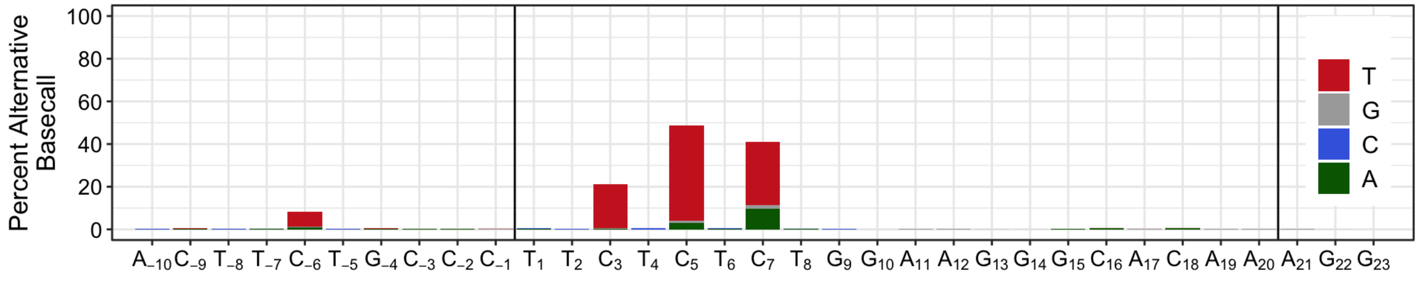

20. A PDCD1 Ex.3 SA + BE4 unconc. mRNA 1.5 µg

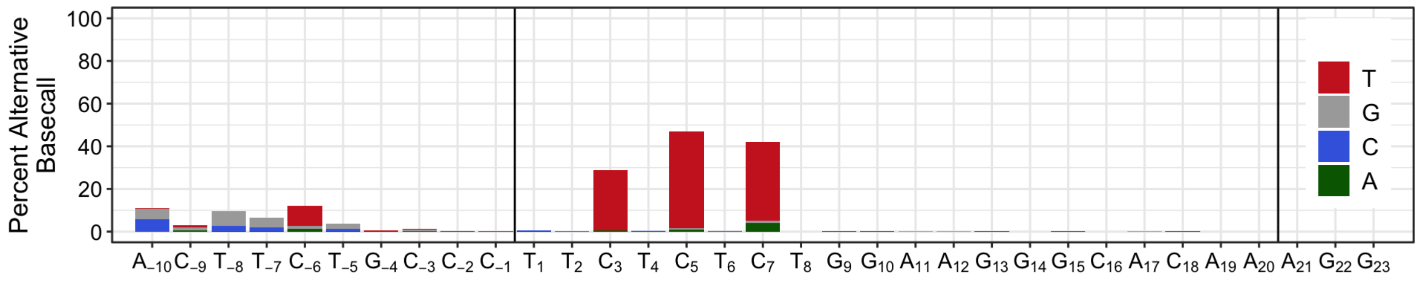

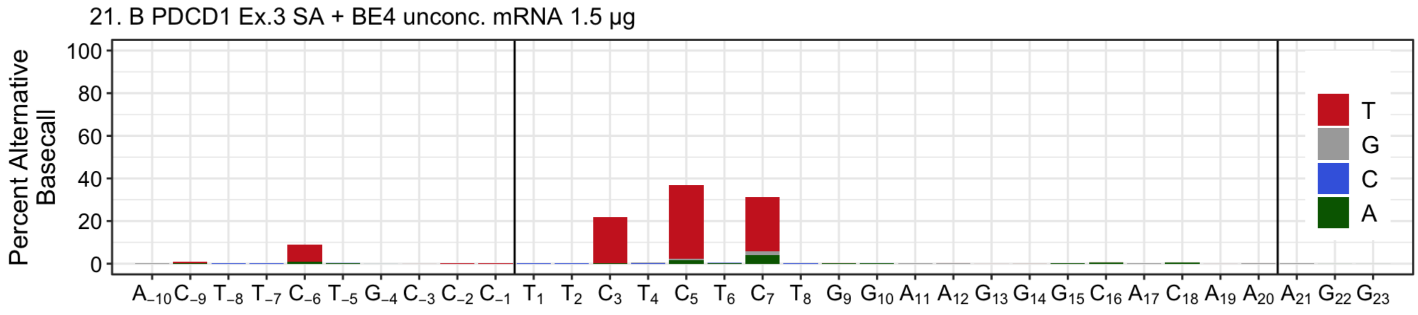
21. B PDCD1 Ex.3 SA + BE4 unconc. mRNA 1.5 µg
Percent Alternative Basecall
T
G
C
A

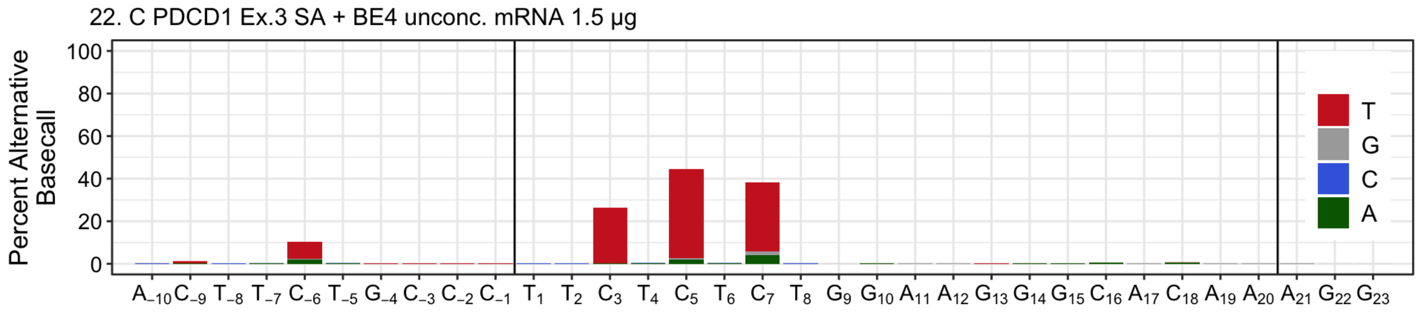
22. C PDCD1 Ex.3 SA + BE4 unconc. mRNA 1.5 µg
Percent Alternative Basecall
T
G
C
A

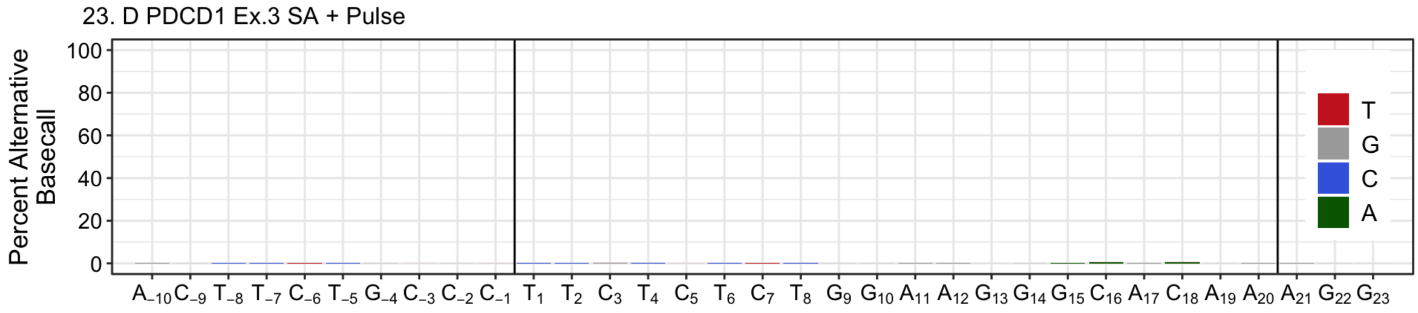
23. D PDCD1 Ex.3 SA + Pulse
Percent Alternative Basecall
T
G
C
A

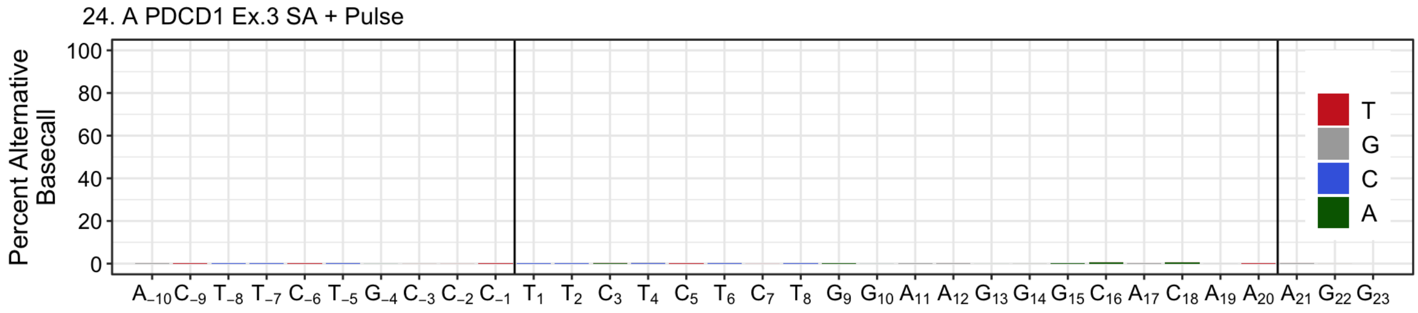
24. A PDCD1 Ex.3 SA + Pulse
Percent Alternative Basecall
T
G
C
A

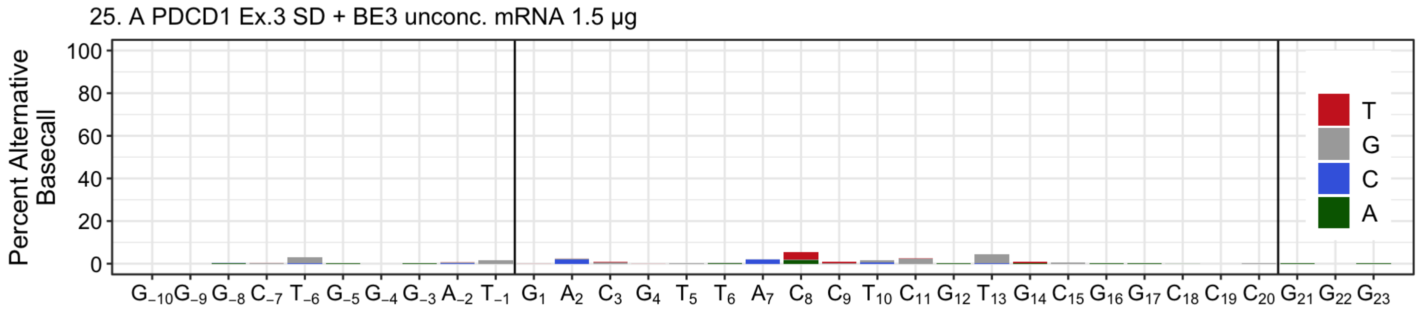
25. A PDCD1 Ex.3 SD + BE3 unconc. mRNA 1.5 µg
Percent Alternative Basecall
T
G
C
A

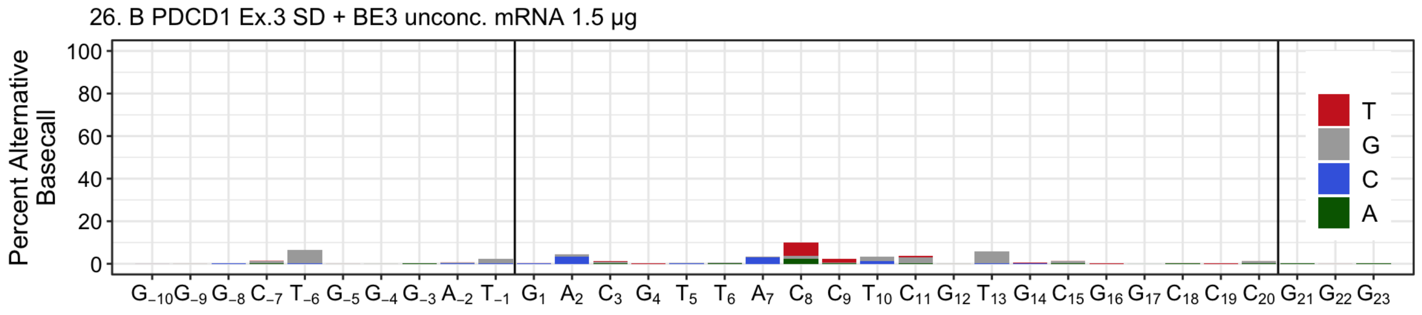
26. B PDCD1 Ex.3 SD + BE3 unconc. mRNA 1.5 µg
Percent Alternative Basecall
T
G
C
A

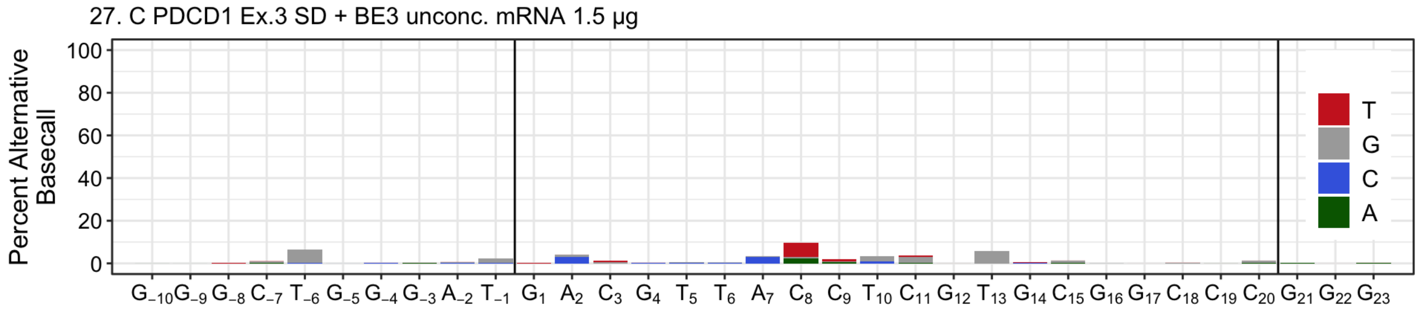
27. C PDCD1 Ex.3 SD + BE3 unconc. mRNA 1.5 µg
Percent Alternative Basecall
T
G
C
A

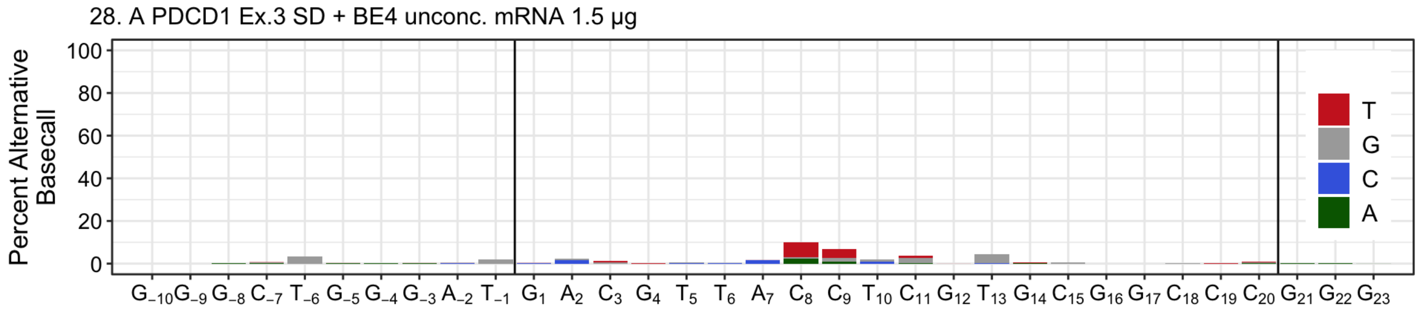
28. A PDCD1 Ex.3 SD + BE4 unconc. mRNA 1.5 µg
Percent Alternative Basecall
T
G
C
A

29. B PDCD1 Ex.3 SD + BE4 unconc. mRNA 1.5 µg

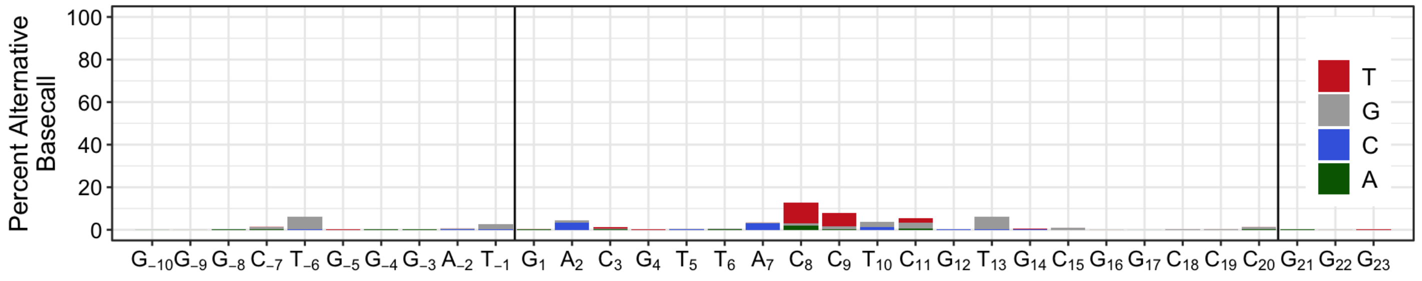

30. C PDCD1 Ex.3 SD + BE4 unconc. mRNA 1.5 µg

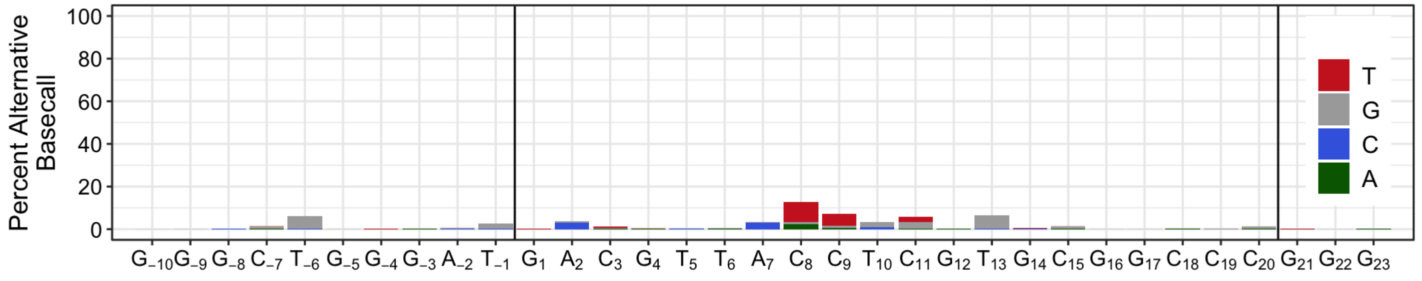

31. A PDCD1 Ex.4 SA + BE3 unconc. mRNA 1.5 µg

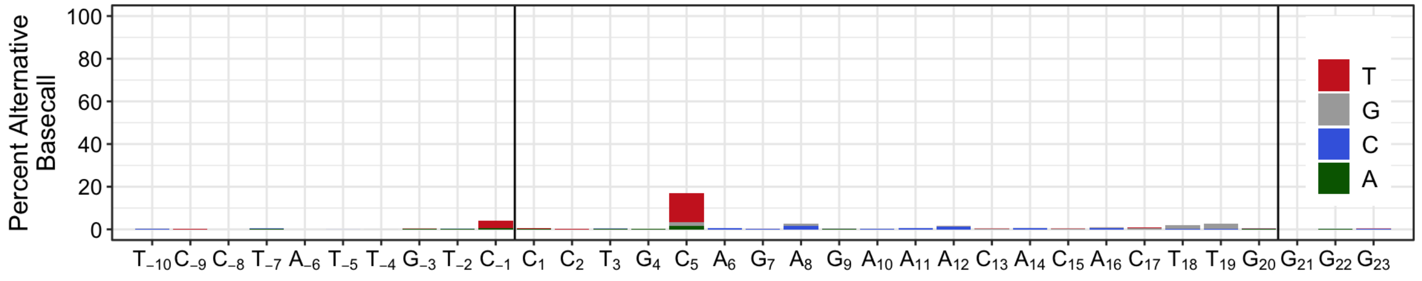

32. B PDCD1 Ex.4 SA + BE3 unconc. mRNA 1.5 µg

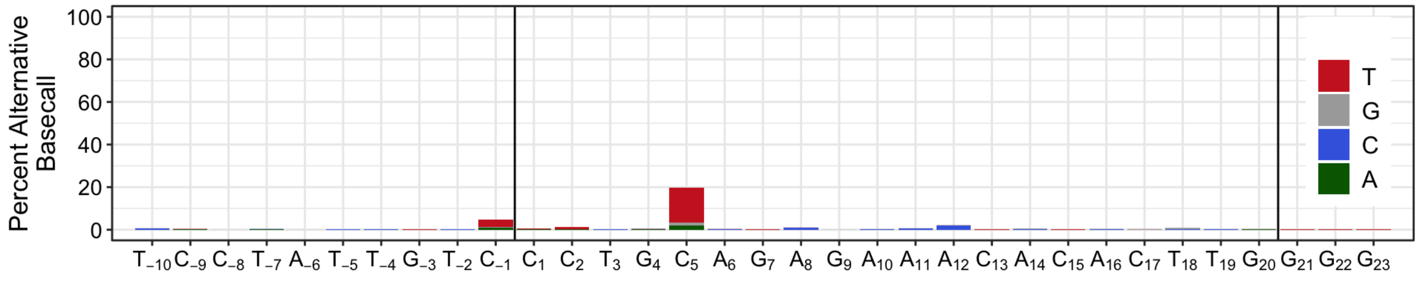

33. C PDCD1 Ex.4 SA + BE3 unconc. mRNA 1.5 μg

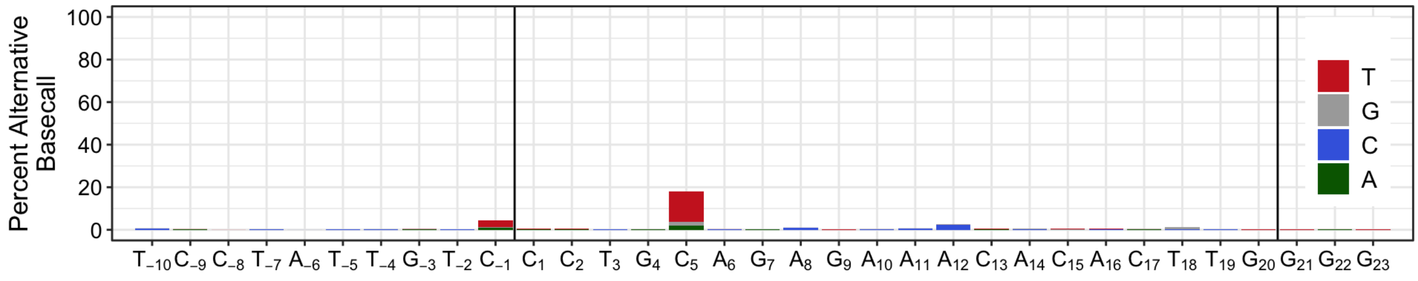

34. A PDCD1 Ex.4 SA + BE4 unconc. mRNA 1.5 μg

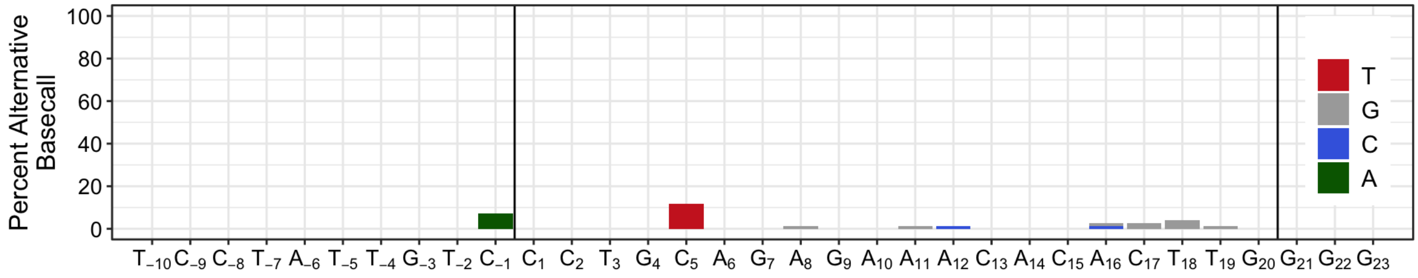

35. B PDCD1 Ex.4 SA + BE4 unconc. mRNA 1.5 μg

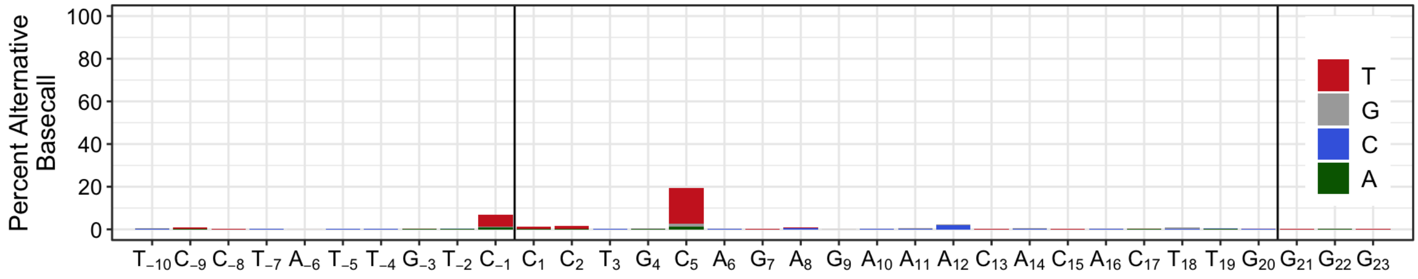

36. C PDCD1 Ex.4 SA + BE4 unconc. mRNA 1.5 μg

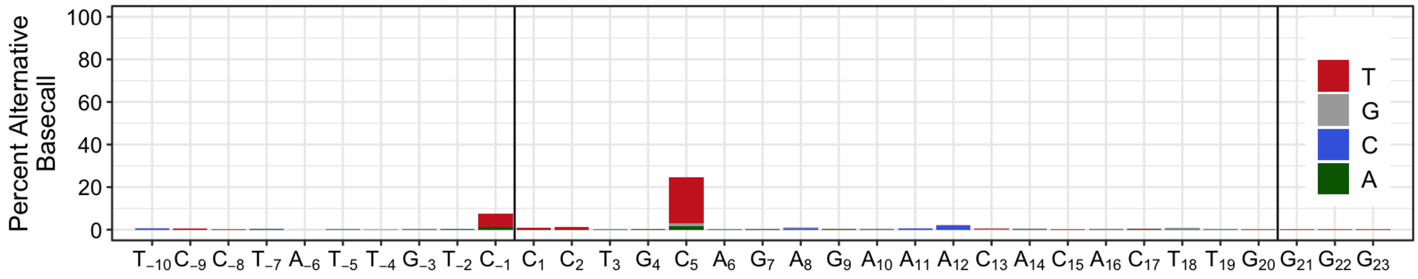

37. D PDCD1 Ex.4 SA + Pulse

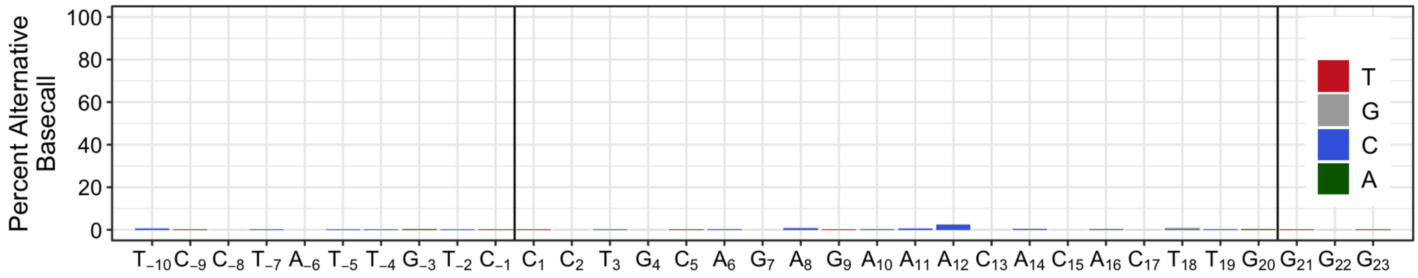

38. A PDCD1 Ex.4 SA + Pulse

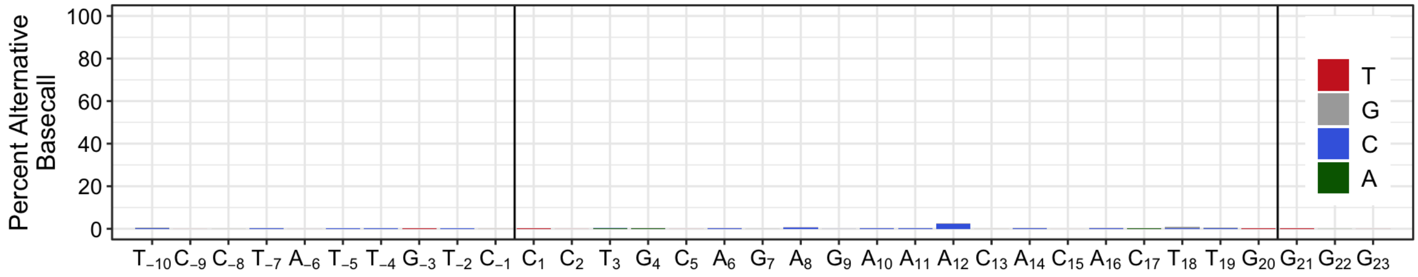

39. A PDCD1 Ex.2 pmSTOP + BE3 unconc. mRNA 1.5 µg

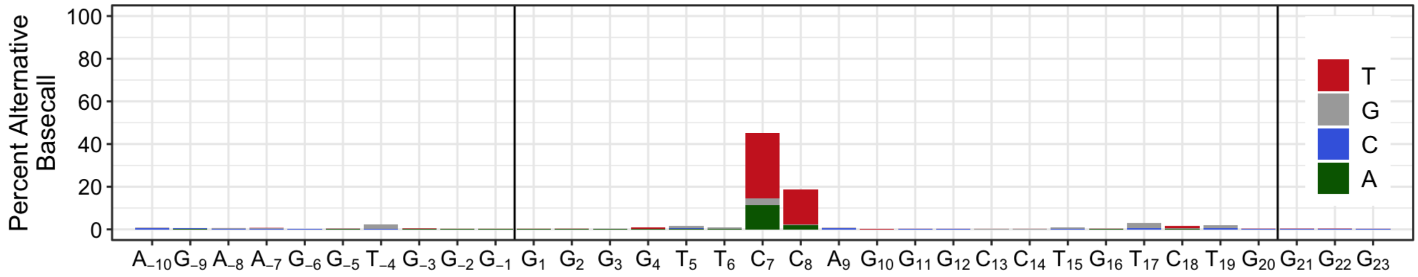

40. B PDCD1 Ex.2 pmSTOP + BE3 unconc. mRNA 1.5 µg

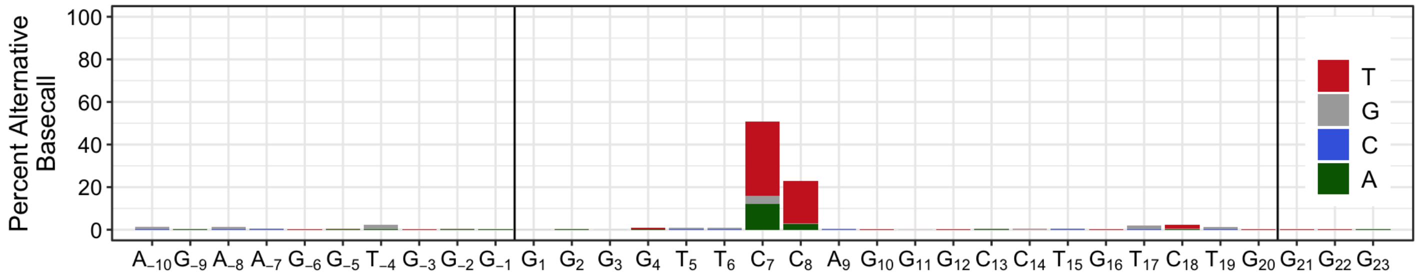

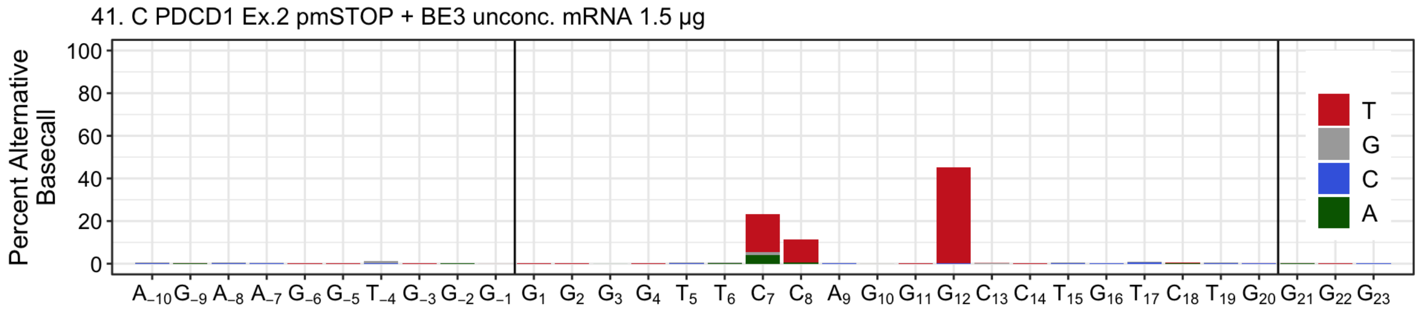
41. C PDCD1 Ex.2 pmSTOP + BE3 unconc. mRNA 1.5 µg
Percent Alternative Basecall
T
G
C
A

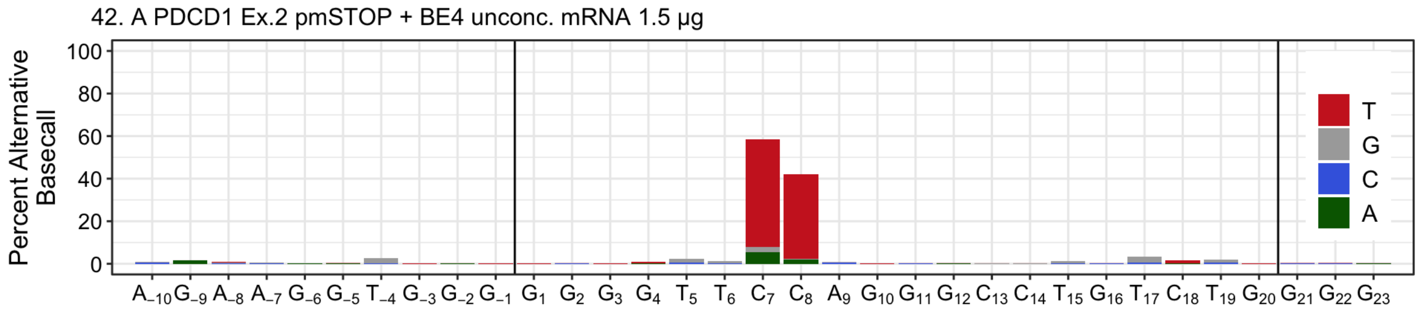
42. A PDCD1 Ex.2 pmSTOP + BE4 unconc. mRNA 1.5 µg
Percent Alternative Basecall
T
G
C
A

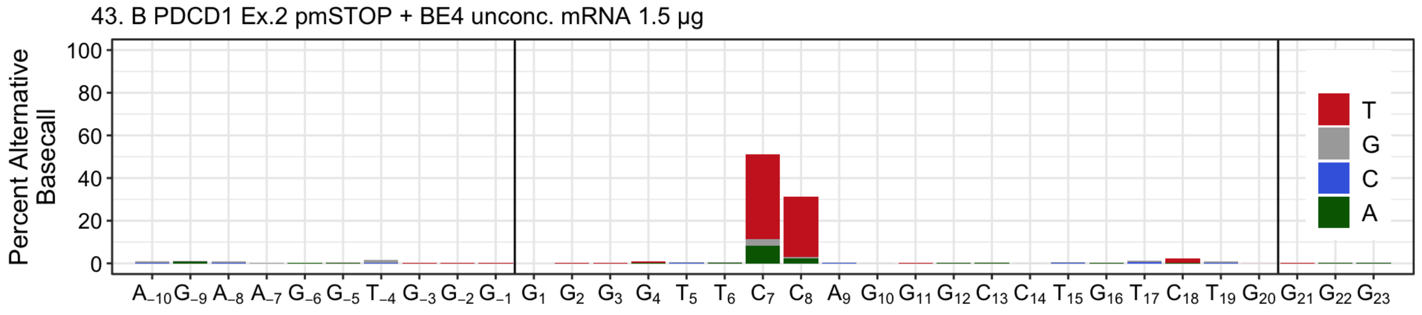
43. B PDCD1 Ex.2 pmSTOP + BE4 unconc. mRNA 1.5 µg
Percent Alternative Basecall
T
G
C
A

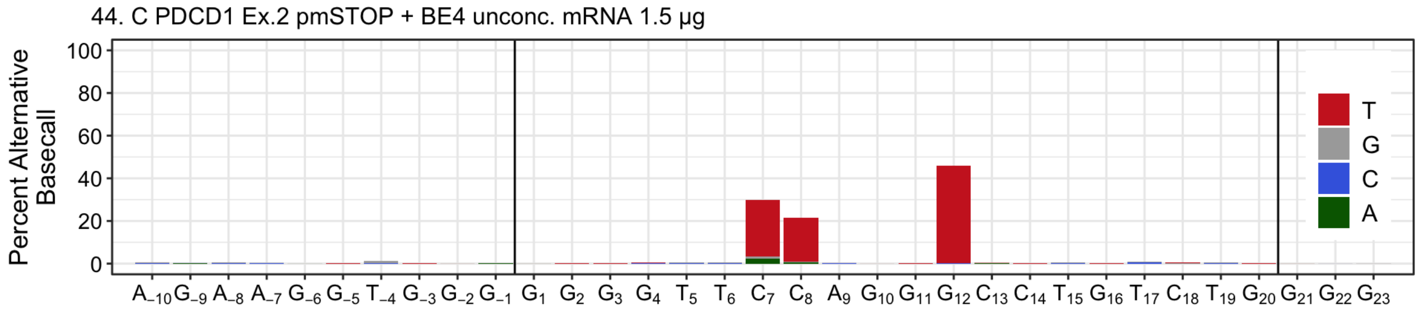
44. C PDCD1 Ex.2 pmSTOP + BE4 unconc. mRNA 1.5 µg
Percent Alternative Basecall
T
G
C
A

45. A PDCD1 Ex.2 pmSTOP + Cas9 mRNA 1.5 µg

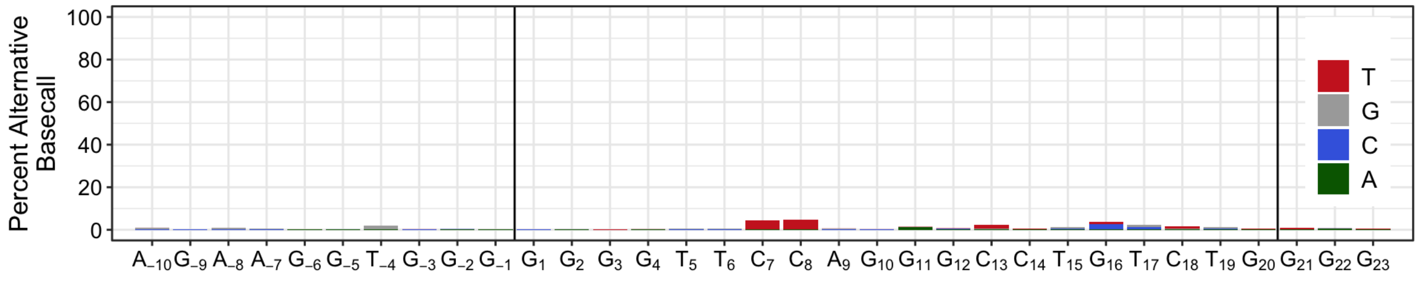

46. B PDCD1 Ex.2 pmSTOP + Cas9 mRNA 1.5 µg

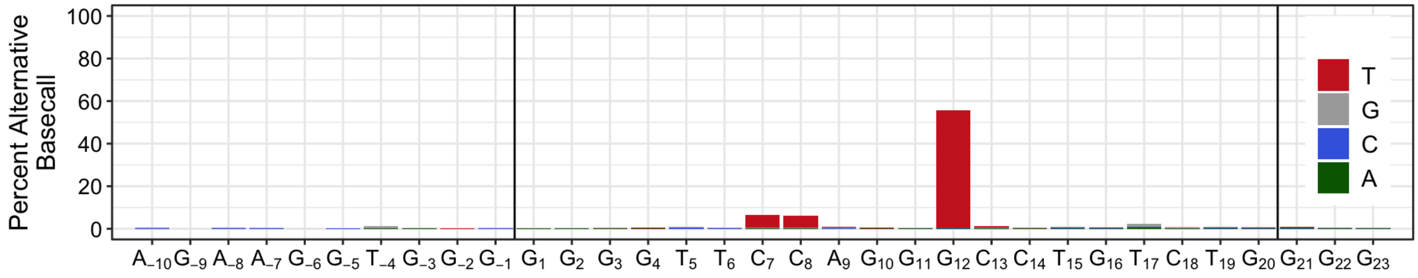

47. D PDCD1 Ex.2 pmSTOP + Pulse

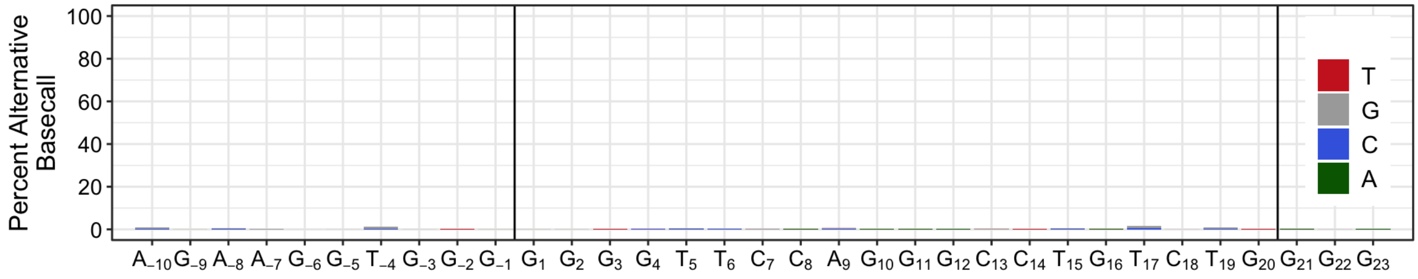

48. D PDCD1 Ex.2 pmSTOP + Pulse

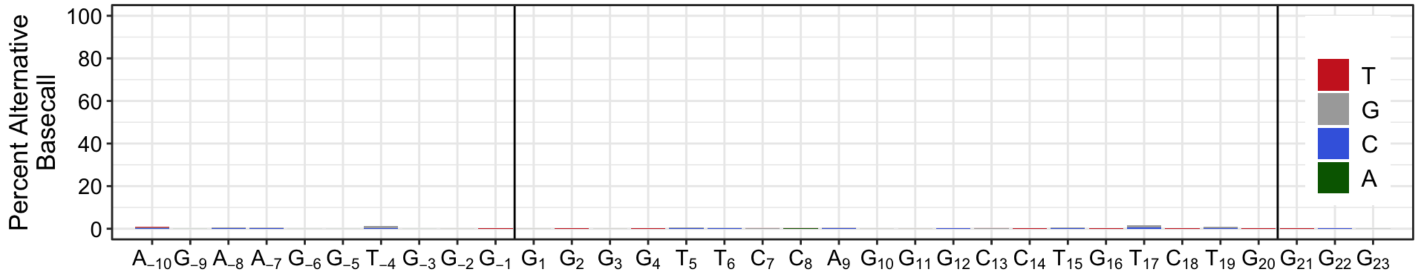

49. A PDCD1 Ex.2 pmSTOP + Pulse

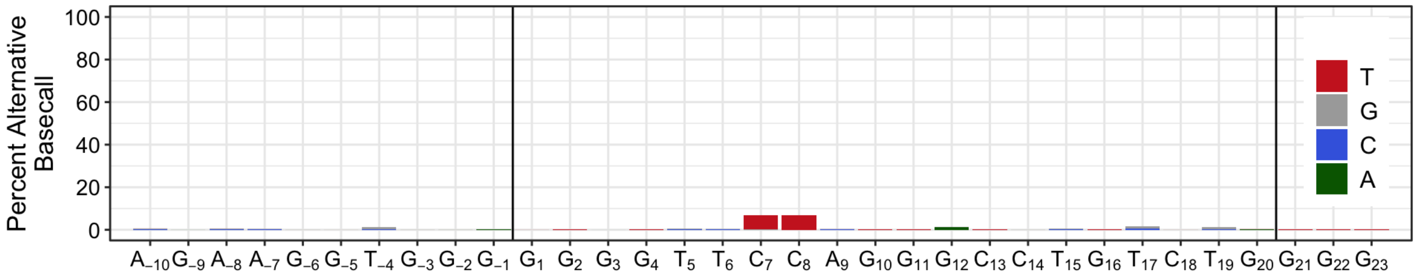

50. A PDCD1 Ex.2 pmSTOP + Pulse

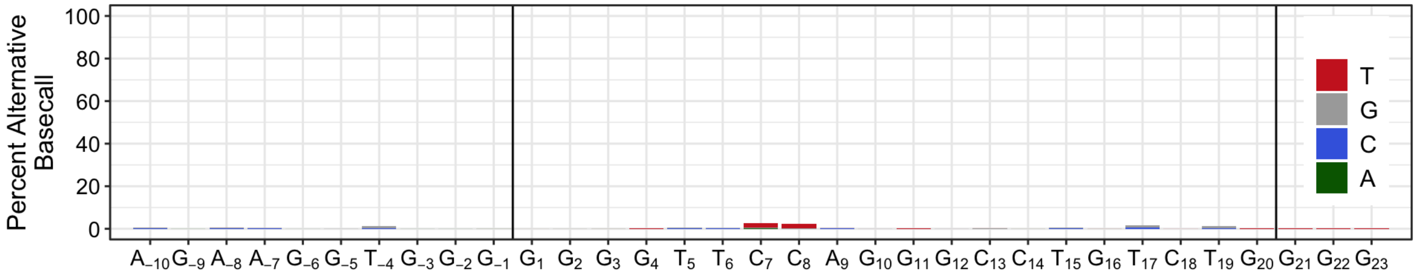

51. A PDCD1 Ex.3 pmSTOP_1 + BE3 unconc. mRNA 1.5 µg

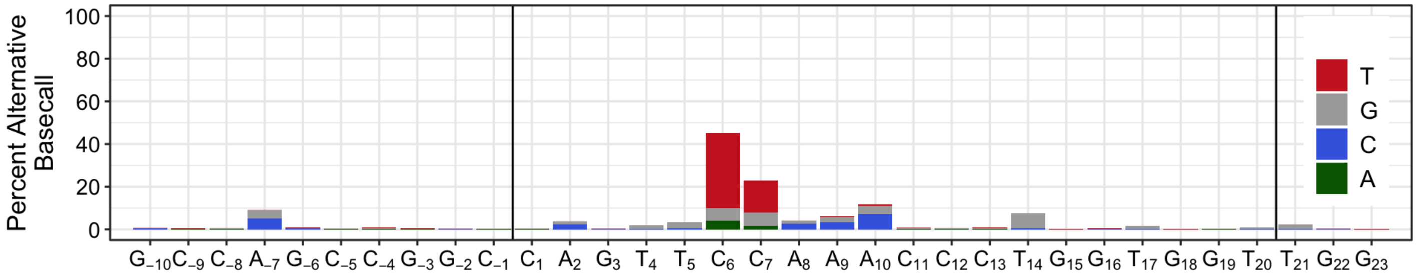

52. B PDCD1 Ex.3 pmSTOP_1 + BE3 unconc. mRNA 1.5 µg

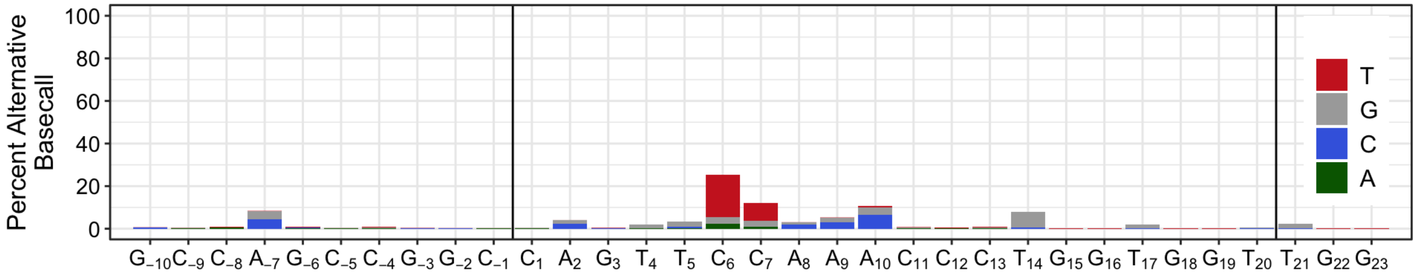

54. D PDCD1 Ex.3 pmSTOP_1 + BE3 unconc. mRNA 1.5 µg

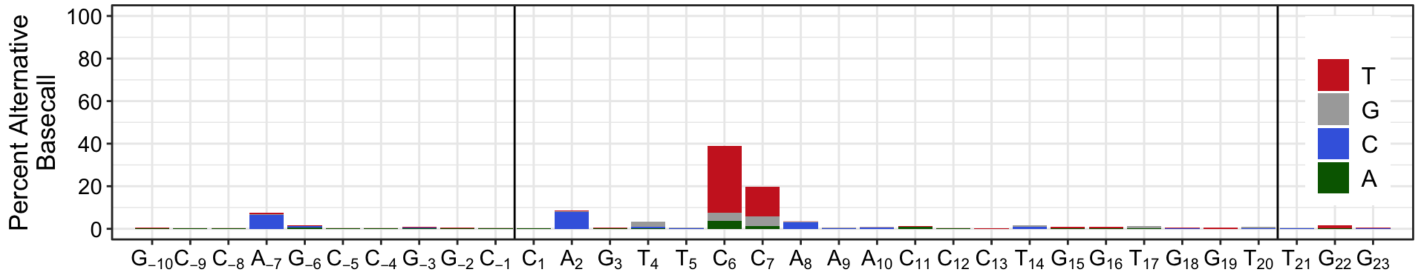

55. A PDCD1 Ex.3 pmSTOP_1 + BE4 unconc. mRNA 1.5 µg

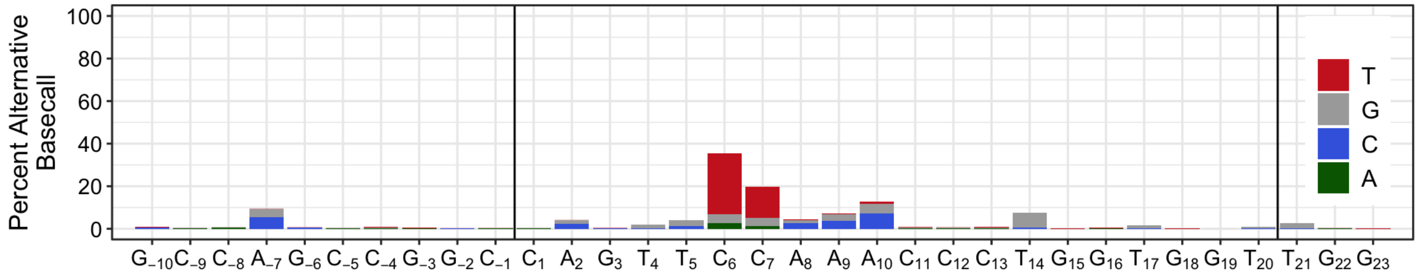

56. B PDCD1 Ex.3 pmSTOP_1 + BE4 unconc. mRNA 1.5 µg

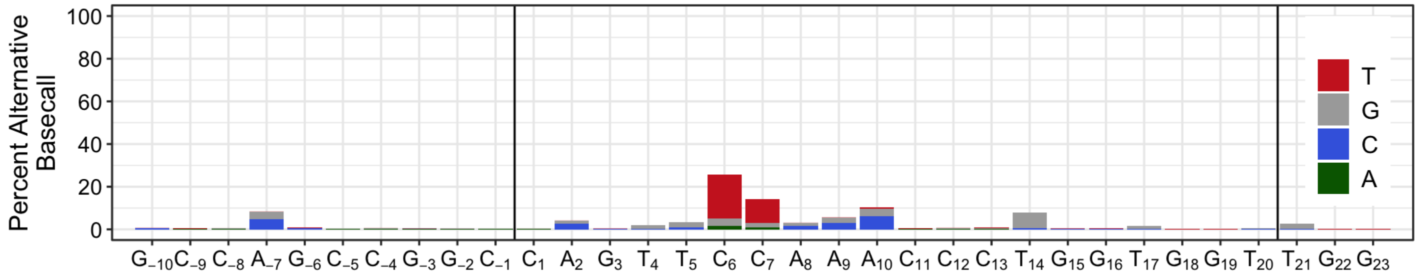

58. D PDCD1 Ex.3 pmSTOP_1 + BE4 unconc. mRNA 1.5 µg

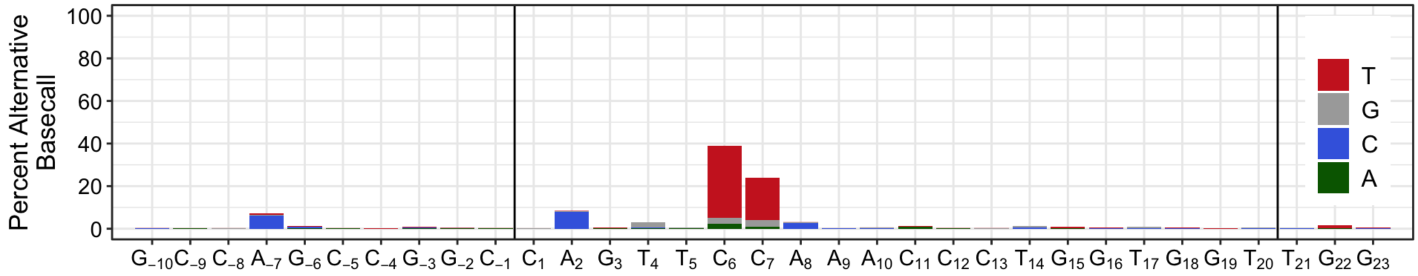

59. D PDCD1 Ex.3 pmSTOP_1 + Pulse

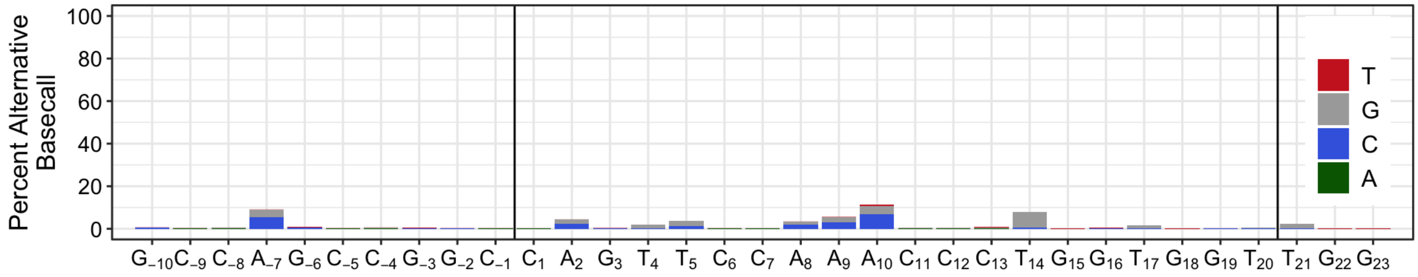

60. A PDCD1 Ex.3 pmSTOP_1 + Pulse

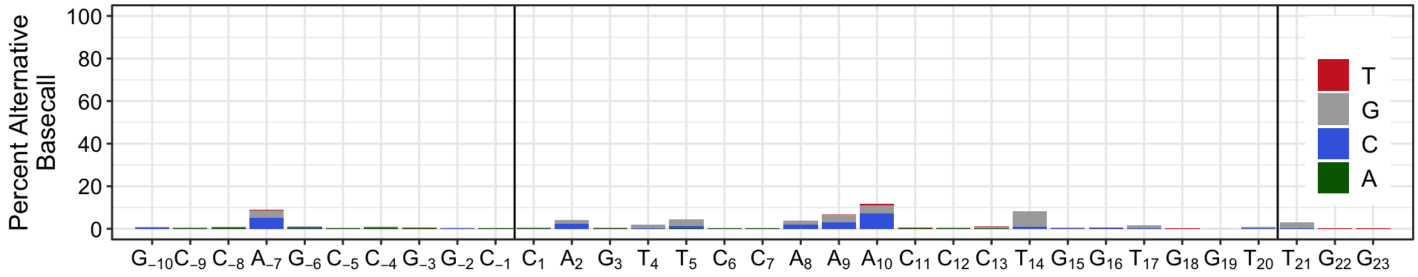

61. A PDCD1 Ex.3 pmSTOP_2 + BE3 unconc. mRNA 1.5 µg

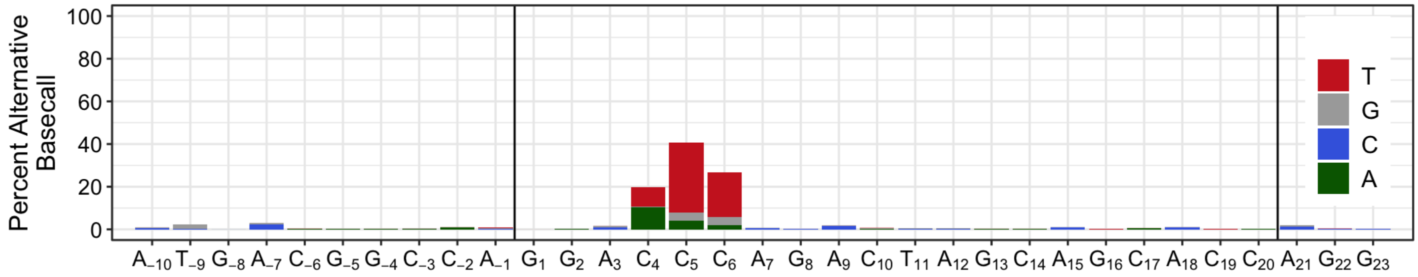

62. B PDCD1 Ex.3 pmSTOP_2 + BE3 unconc. mRNA 1.5 µg

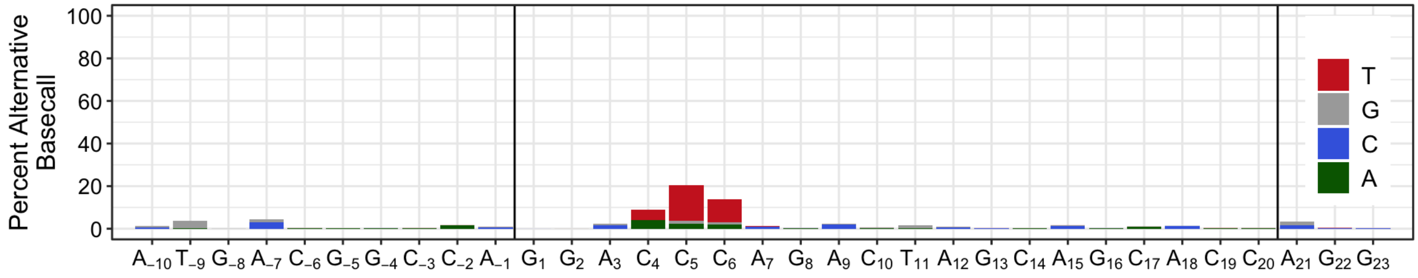

63. C PDCD1 Ex.3 pmSTOP_2 + BE3 unconc. mRNA 1.5 µg

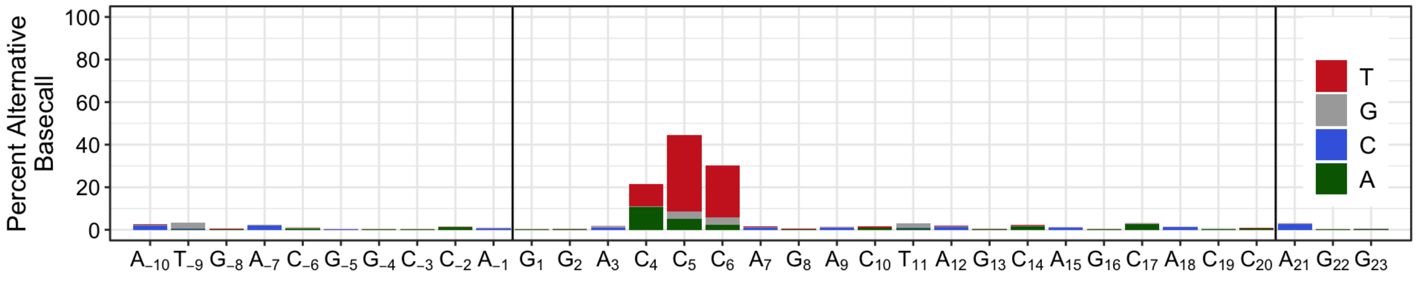

64. A PDCD1 Ex.3 pmSTOP_2 + BE4 unconc. mRNA 1.5 µg

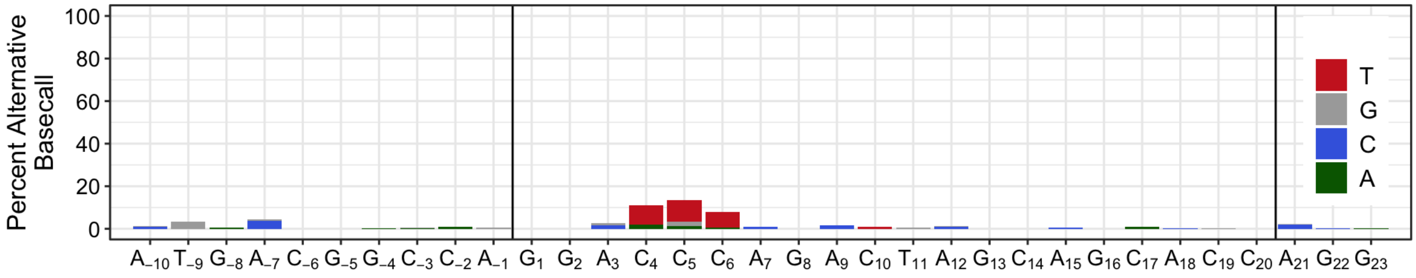

65. B PDCD1 Ex.3 pmSTOP_2 + BE4 unconc. mRNA 1.5 µg

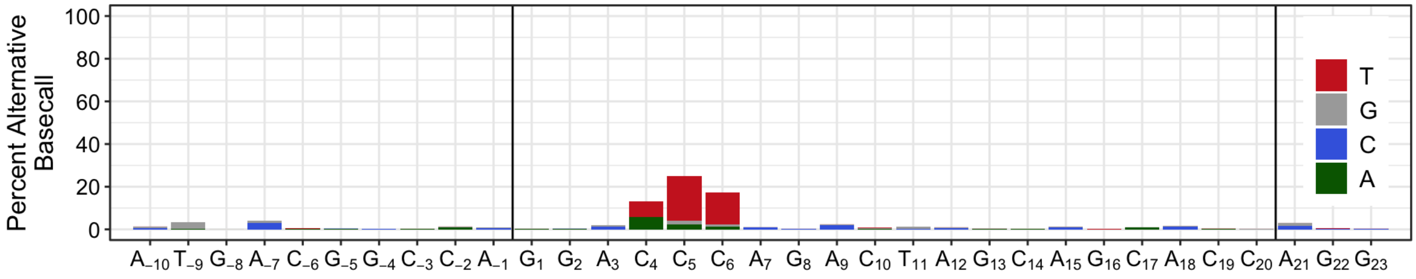

66. C PDCD1 Ex.3 pmSTOP_2 + BE4 unconc. mRNA 1.5 µg

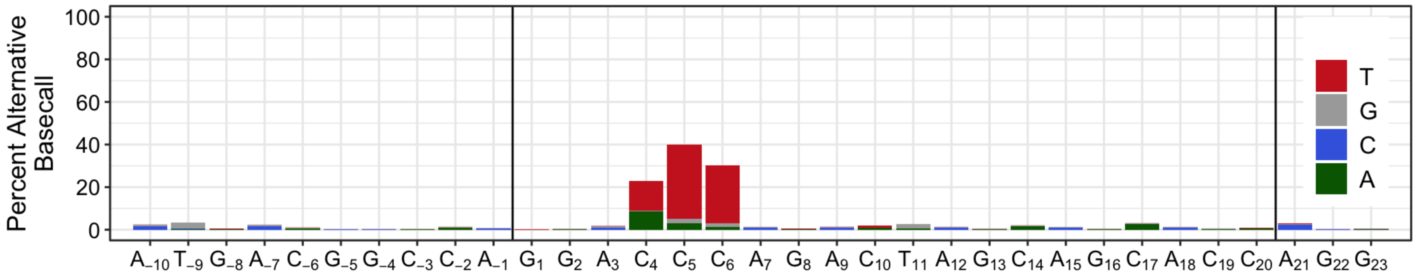

67. D PDCD1 Ex.3 pmSTOP_2 + Pulse

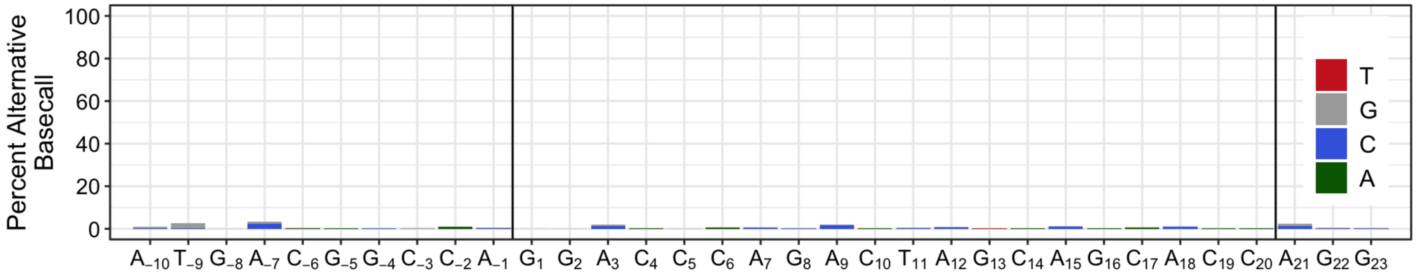

68. A PDCD1 Ex.3 pmSTOP_2 + Pulse

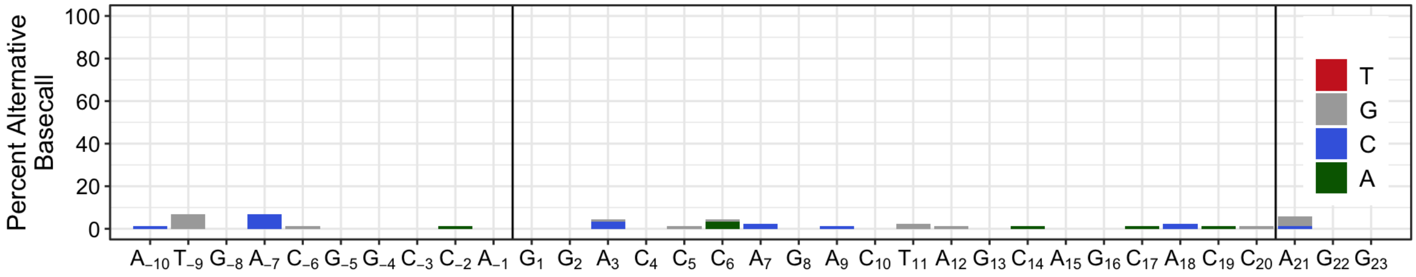

69. A TRAC Ex.1 SD + BE3 unconc. mRNA 1.5 µg

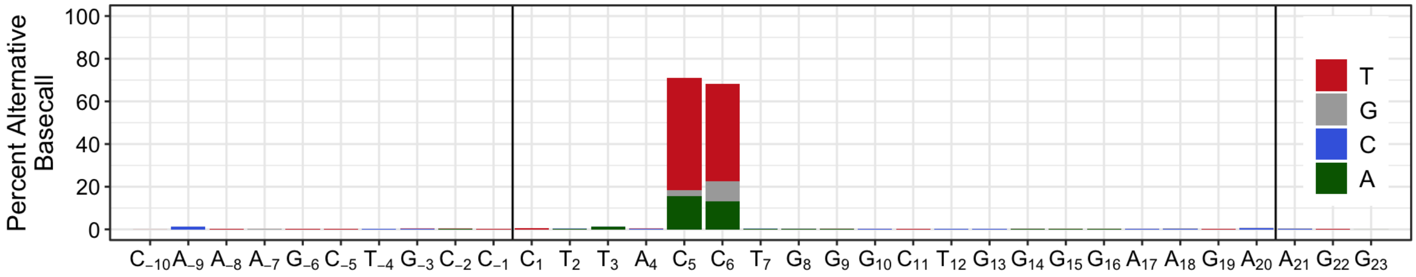

70. B TRAC Ex.1 SD + BE3 unconc. mRNA 1.5 µg

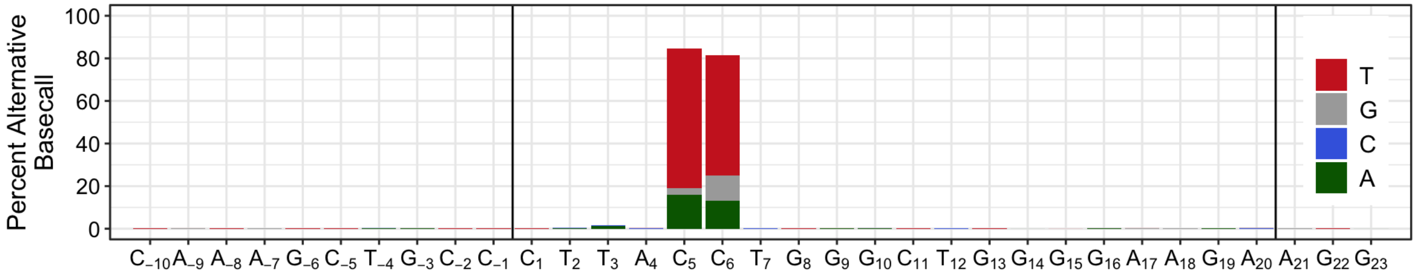

71. C TRAC Ex.1 SD + BE3 unconc. mRNA 1.5 µg

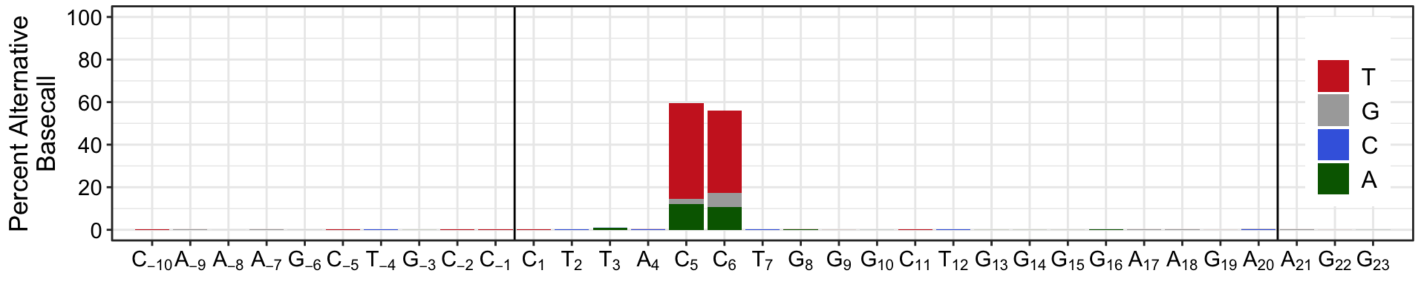

72. D TRAC Ex.1 SD + BE3 unconc. mRNA 1.5 µg

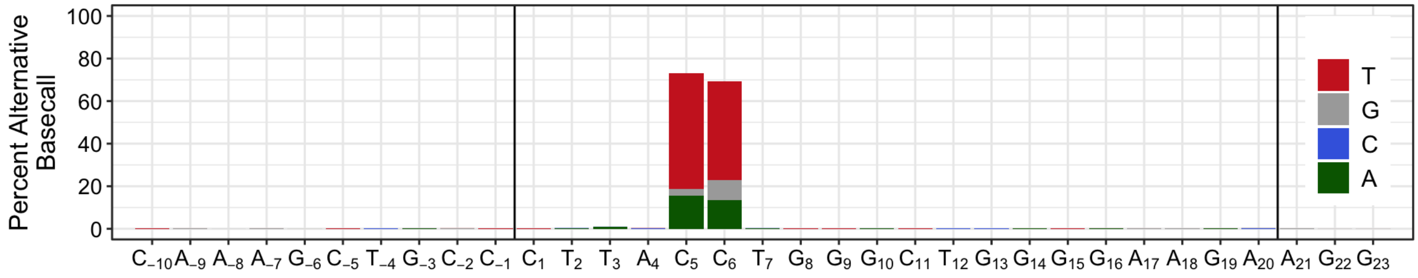

73. A TRAC Ex.1 SD + BE4 unconc. mRNA 1.5 µg

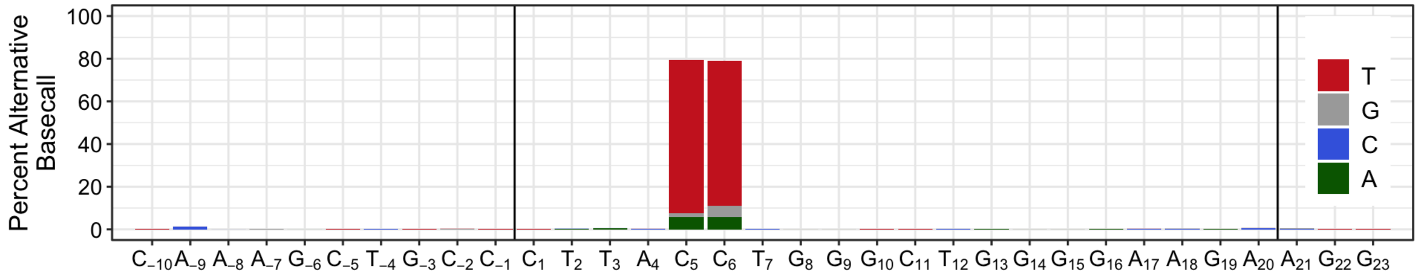

74. B TRAC Ex.1 SD + BE4 unconc. mRNA 1.5 µg

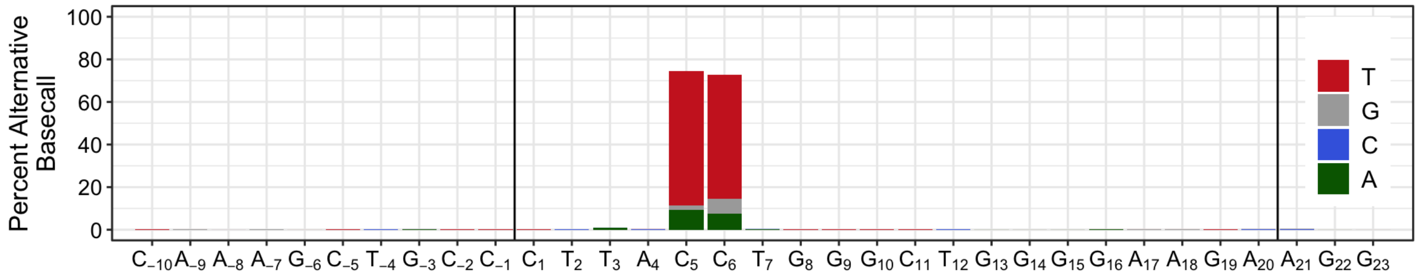

75. C TRAC Ex.1 SD + BE4 unconc. mRNA 1.5 µg

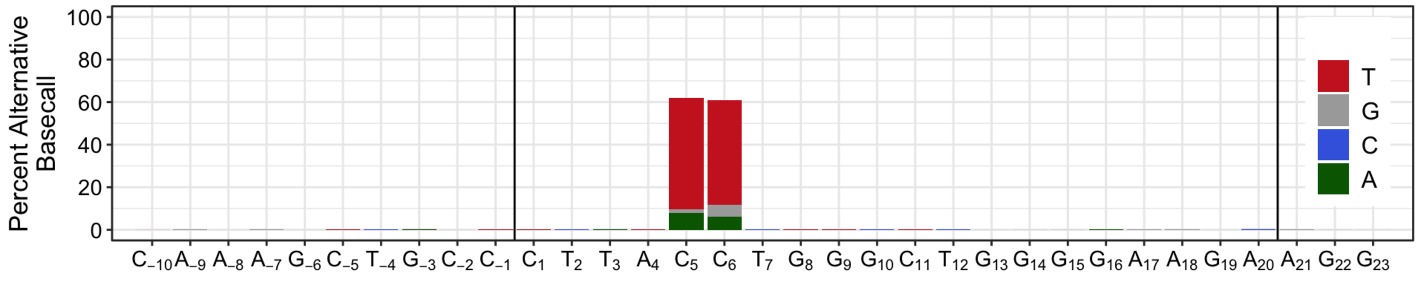

76. D TRAC Ex.1 SD + BE4 unconc. mRNA 1.5 µg

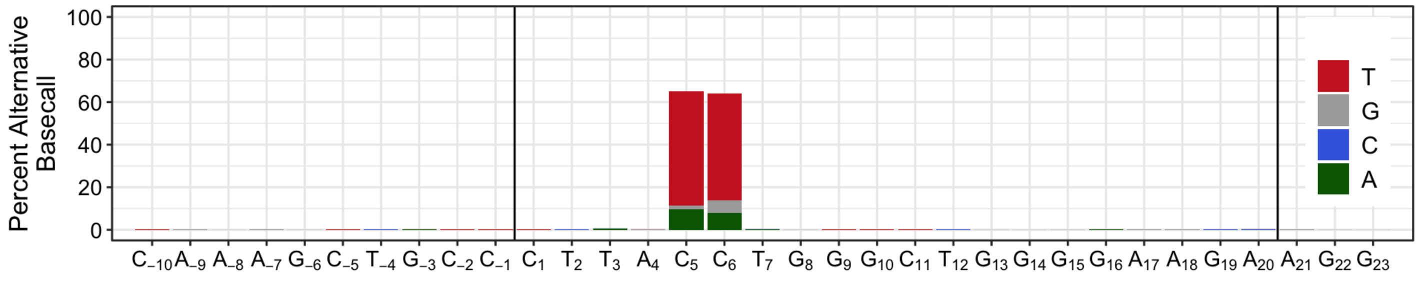

77. D TRAC Ex.1 SD + Cas9 mRNA 1.5 µg

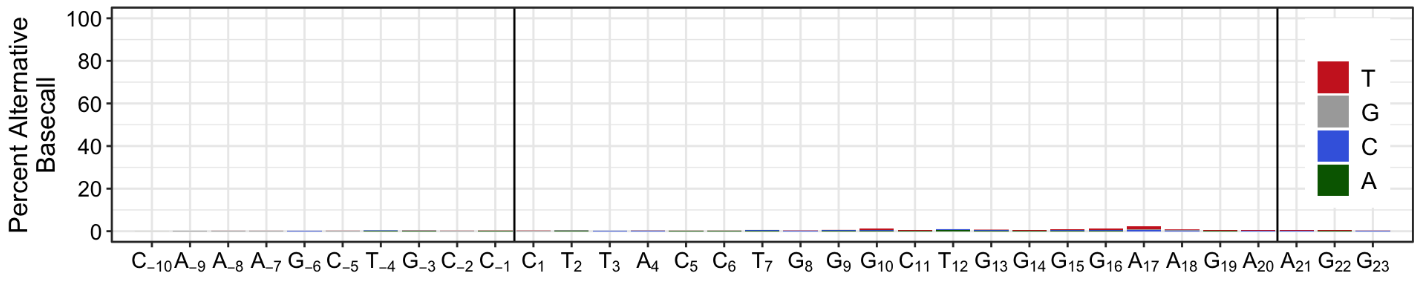

78. D TRAC Ex.1 SD + Pulse

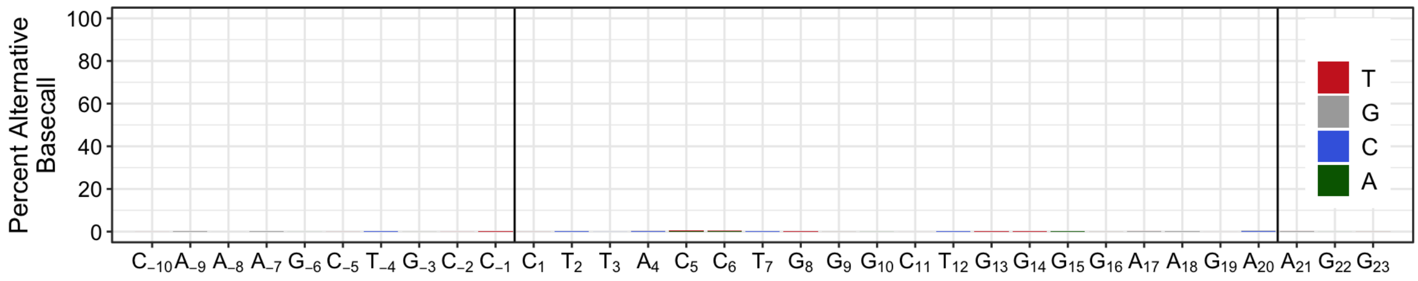

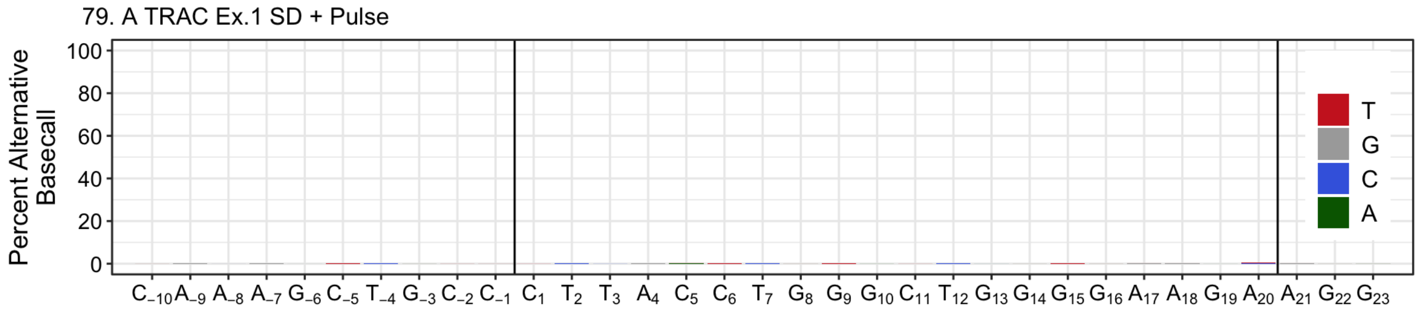
79. A TRAC Ex.1 SD + Pulse
Percent Alternative Basecall
T
G
C
A

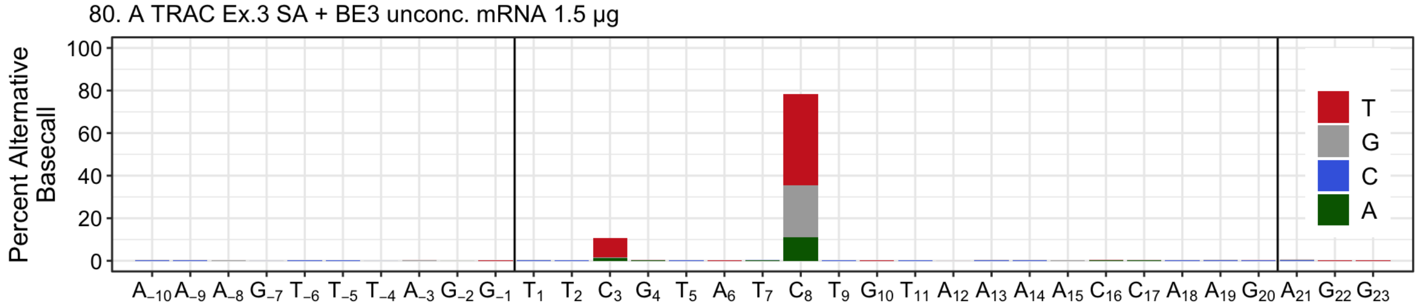
80. A TRAC Ex.3 SA + BE3 unconc. mRNA 1.5 µg
Percent Alternative Basecall
T
G
C
A

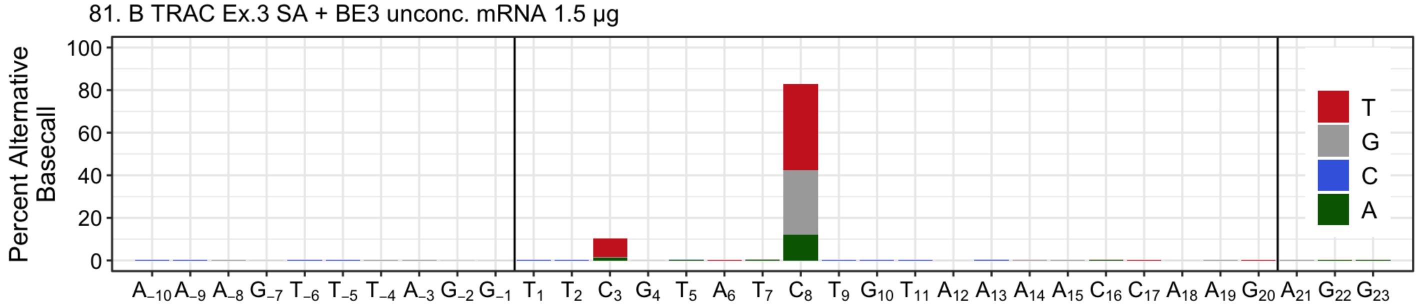
81. B TRAC Ex.3 SA + BE3 unconc. mRNA 1.5 µg
Percent Alternative Basecall
T
G
C
A

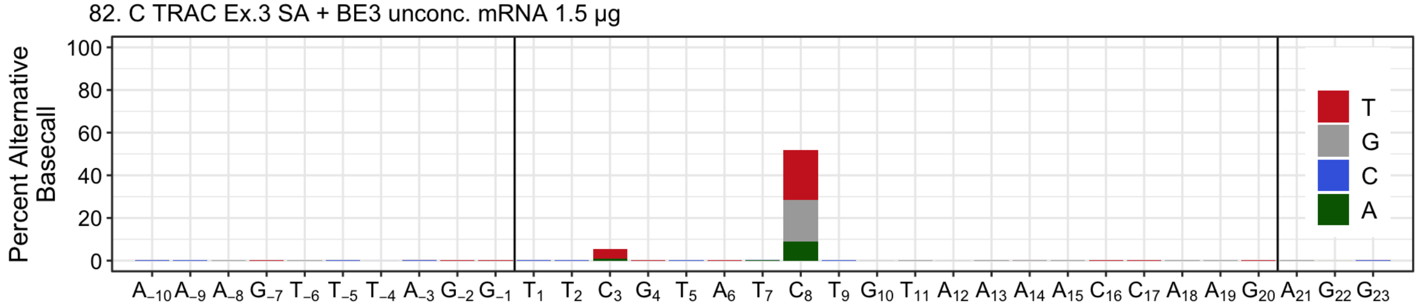
82. C TRAC Ex.3 SA + BE3 unconc. mRNA 1.5 µg
Percent Alternative Basecall
T
G
C
A

83. D TRAC Ex.3 SA + BE3 unconc. mRNA 1.5 µg

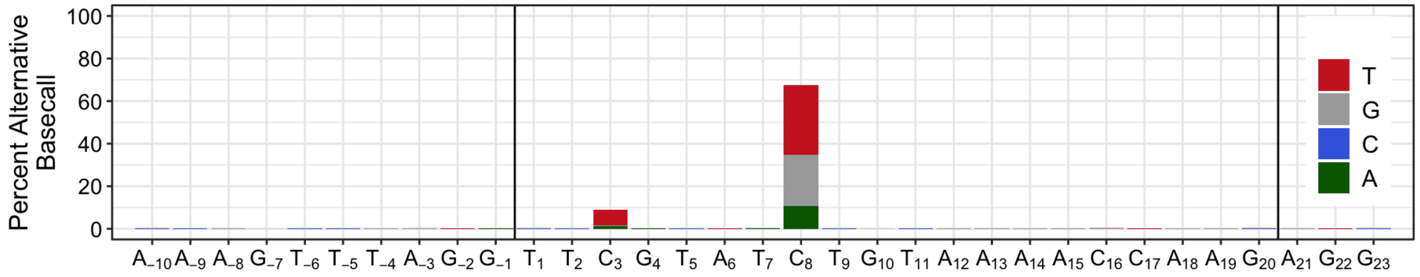

84. A TRAC Ex.3 SA + BE4 unconc. mRNA 1.5 µg

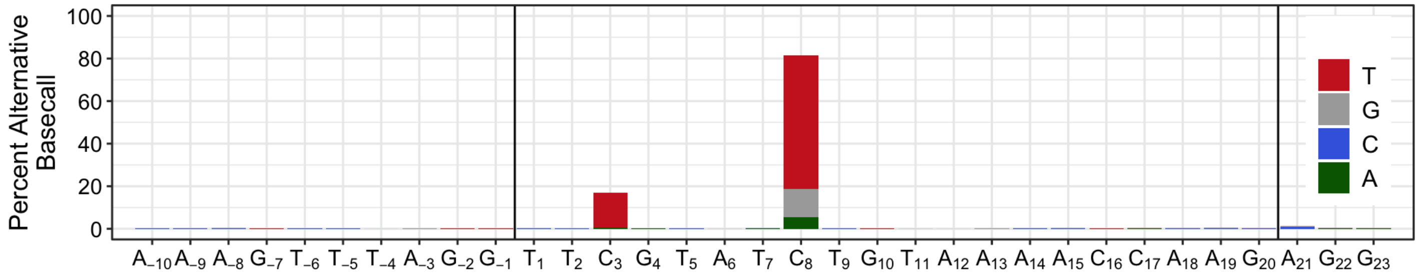

85. B TRAC Ex.3 SA + BE4 unconc. mRNA 1.5 µg

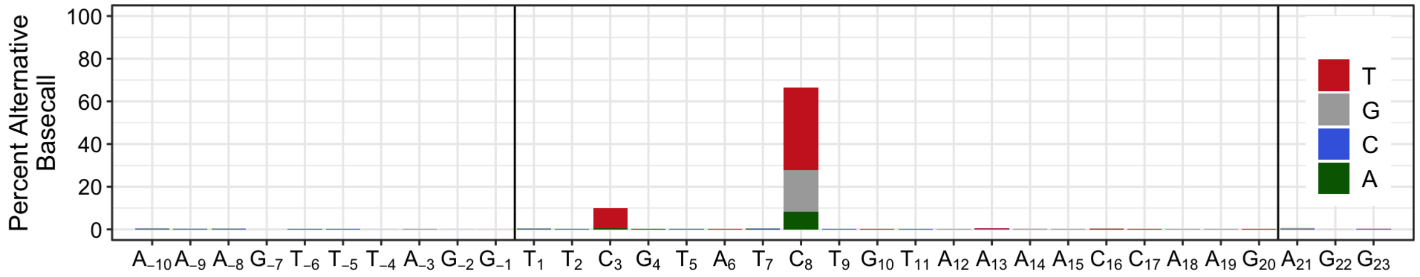

86. C TRAC Ex.3 SA + BE4 unconc. mRNA 1.5 µg

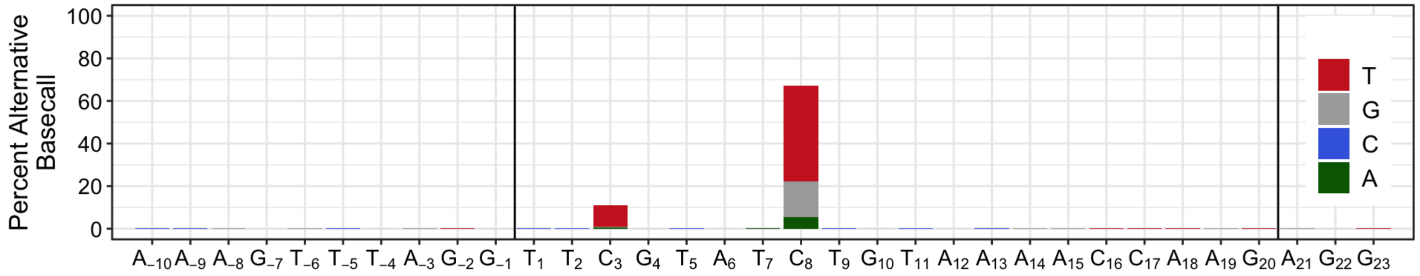

87. D TRAC Ex.3 SA + BE4 unconc. mRNA 1.5 µg

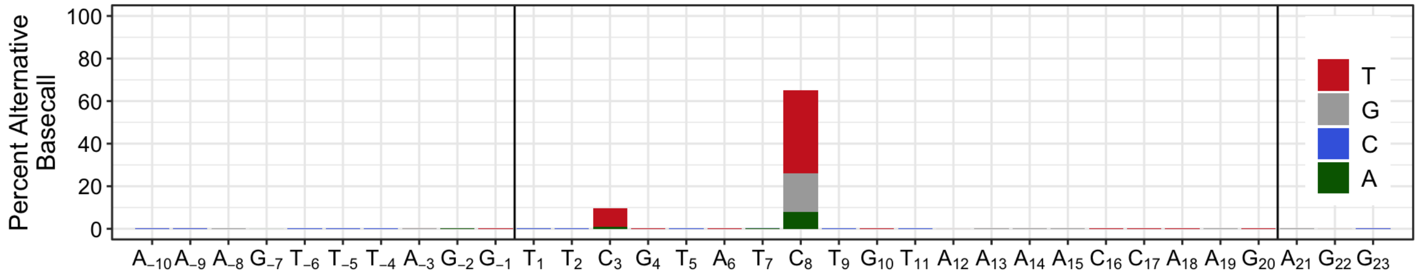

88. A TRAC Ex.3 SA + Cas9 mRNA 1.5 µg

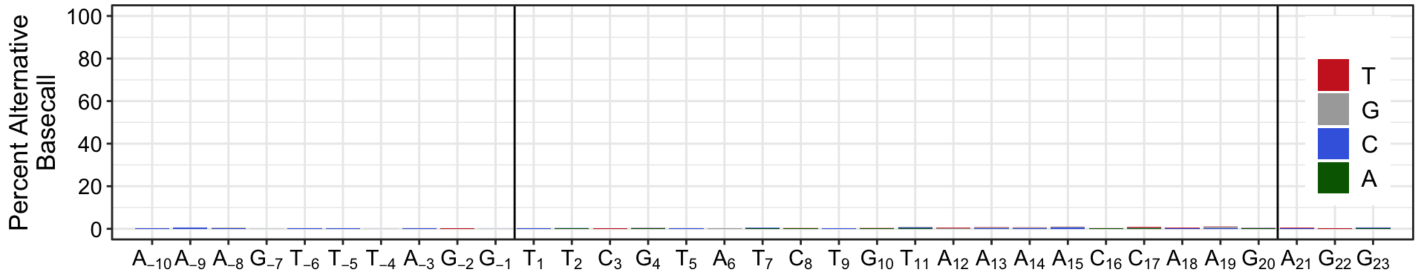

89. A TRAC Ex.3 SA + Pulse

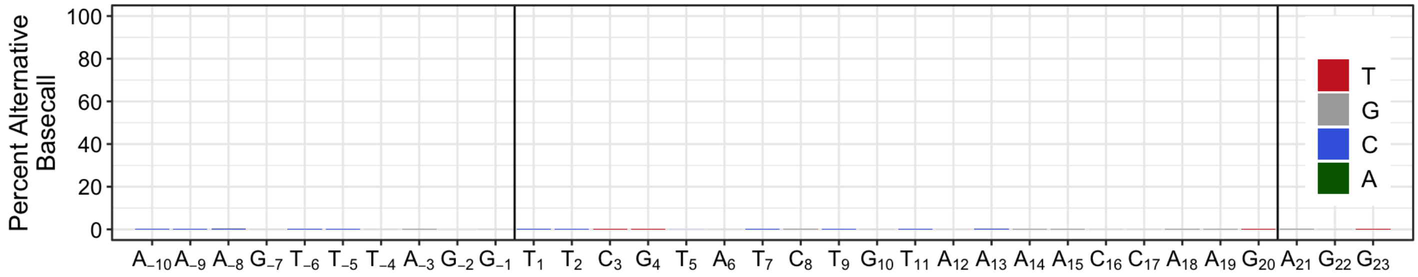

90. D TRAC Ex.3 SA + Pulse

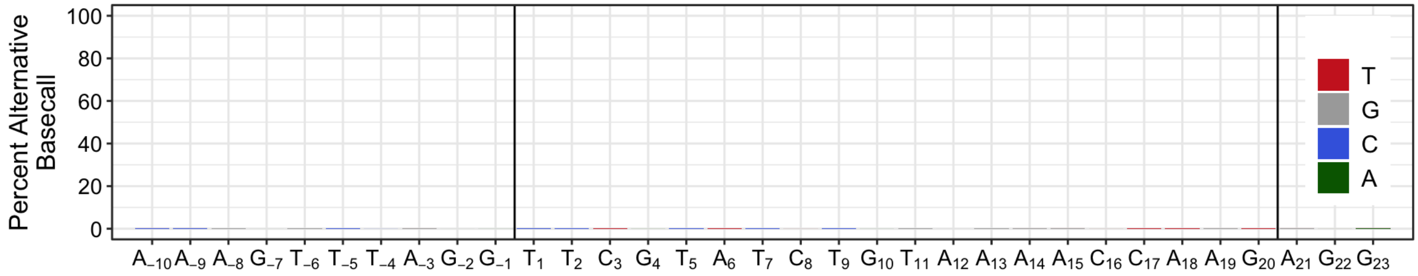

91. A TRAC Ex.3 pmSTOP_1 + BE3 unconc. mRNA 1.5 µg

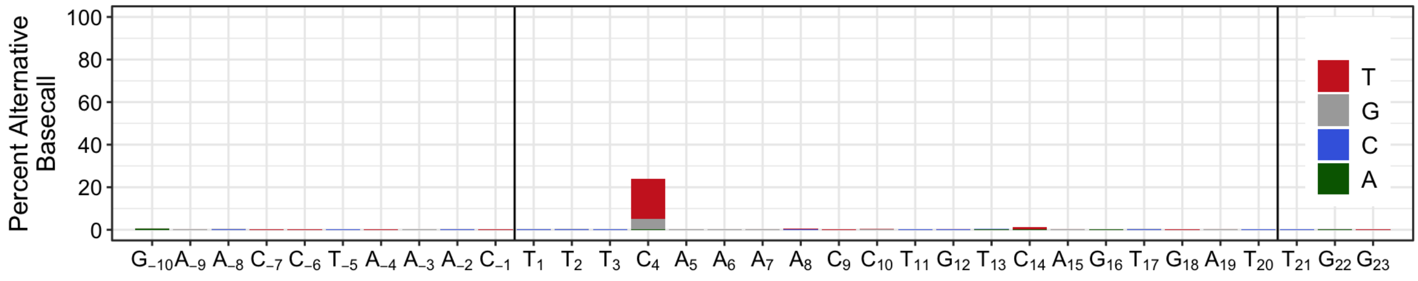

92. B TRAC Ex.3 pmSTOP_1 + BE3 unconc. mRNA 1.5 µg

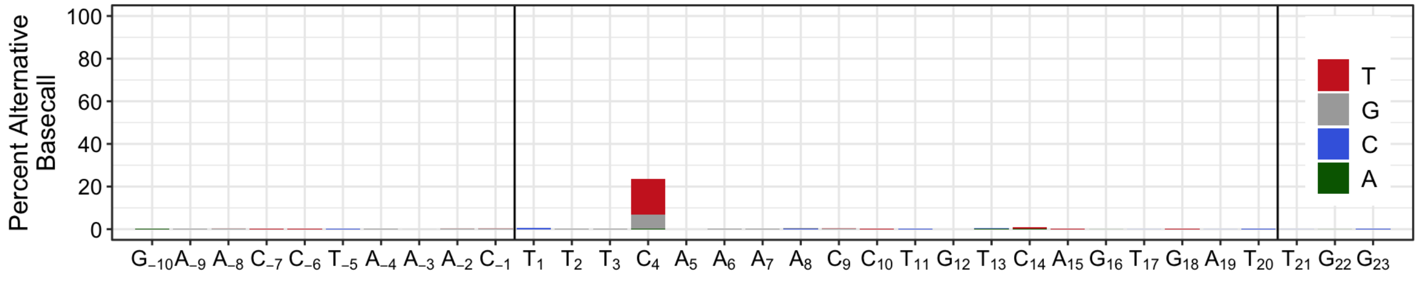

93. C TRAC Ex.3 pmSTOP_1 + BE3 unconc. mRNA 1.5 µg

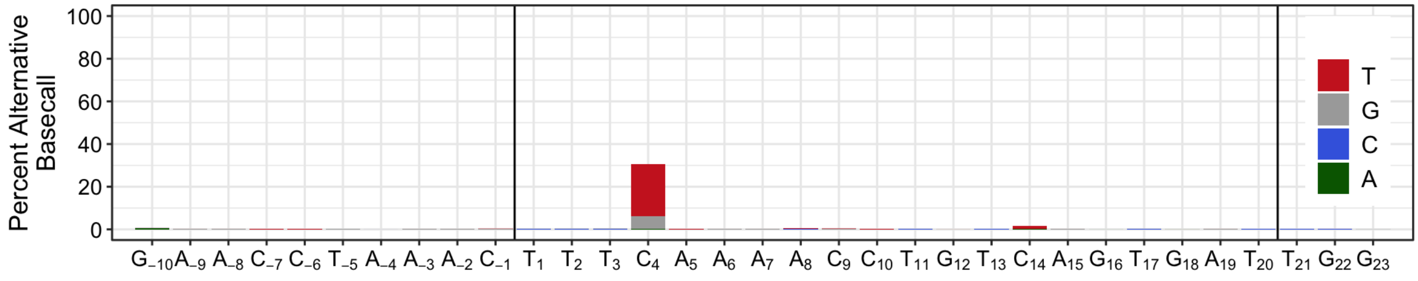

94. A TRAC Ex.3 pmSTOP_1 + BE4 unconc. mRNA 1.5 µg

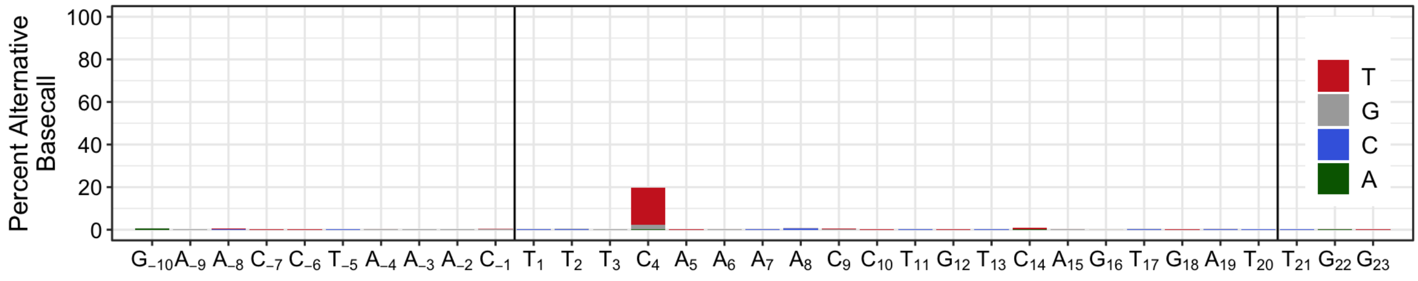

95. B TRAC Ex.3 pmSTOP_1 + BE4 unconc. mRNA 1.5 µg

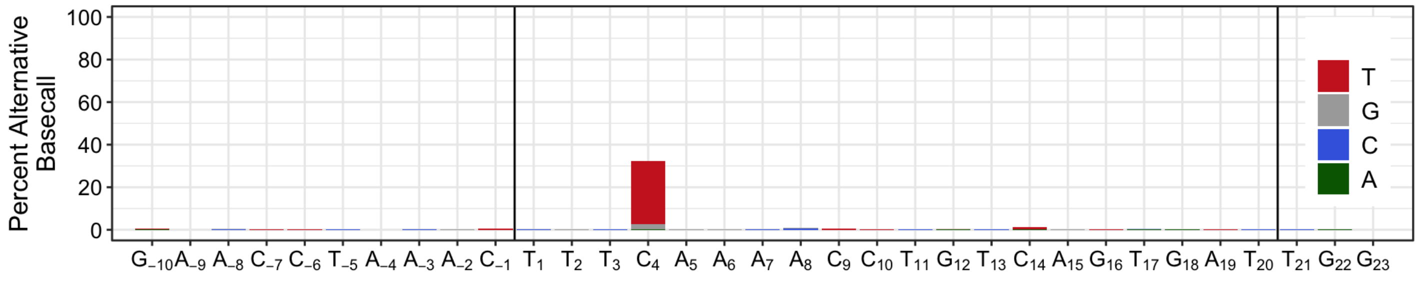

96. C TRAC Ex.3 pmSTOP_1 + BE4 unconc. mRNA 1.5 µg

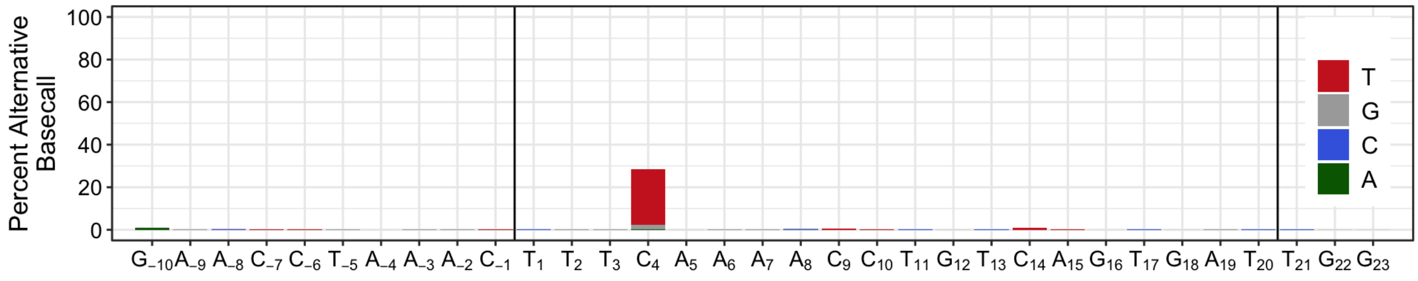

97. D TRAC Ex.3 pmSTOP_1 + Pulse

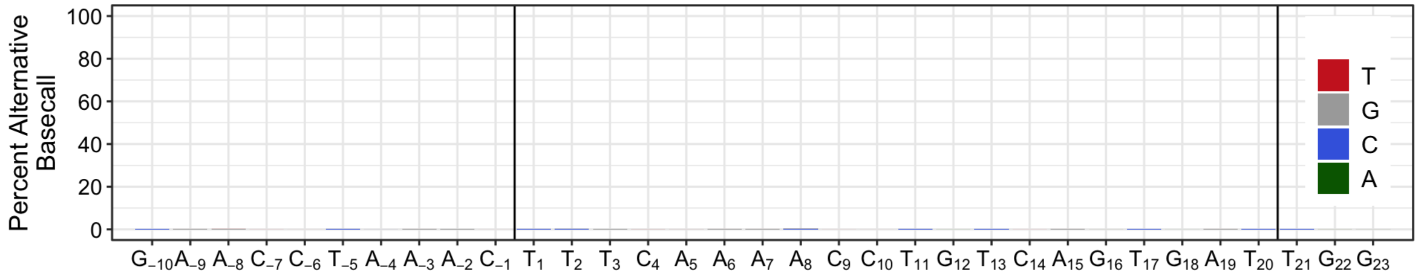

98. A TRAC Ex.3 pmSTOP_1 + Pulse

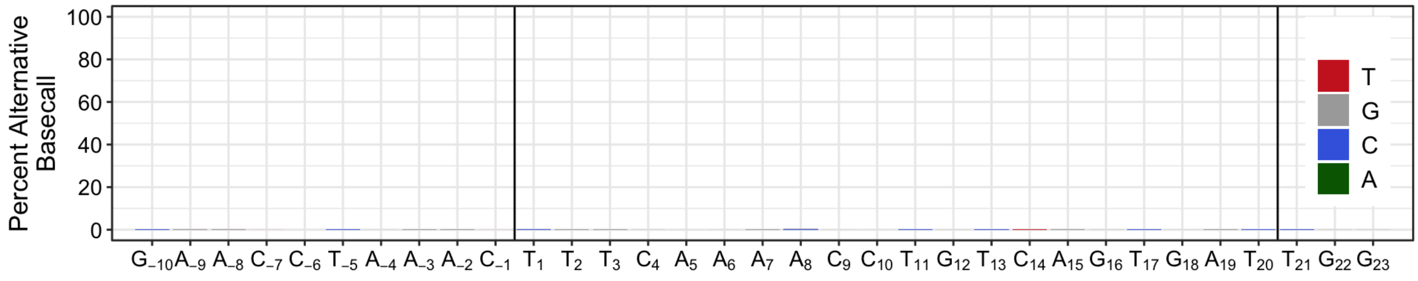

99. A TRAC Ex.3 pmSTOP_2 + BE3 unconc. mRNA 1.5 µg

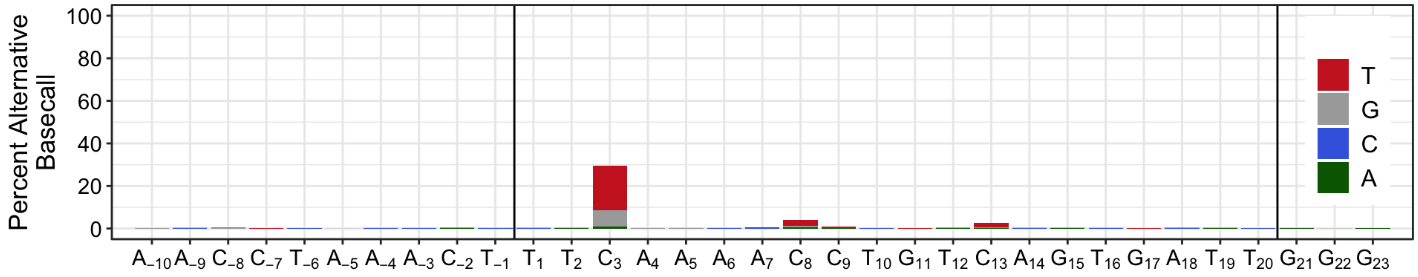

100. B TRAC Ex.3 pmSTOP_2 + BE3 unconc. mRNA 1.5 µg

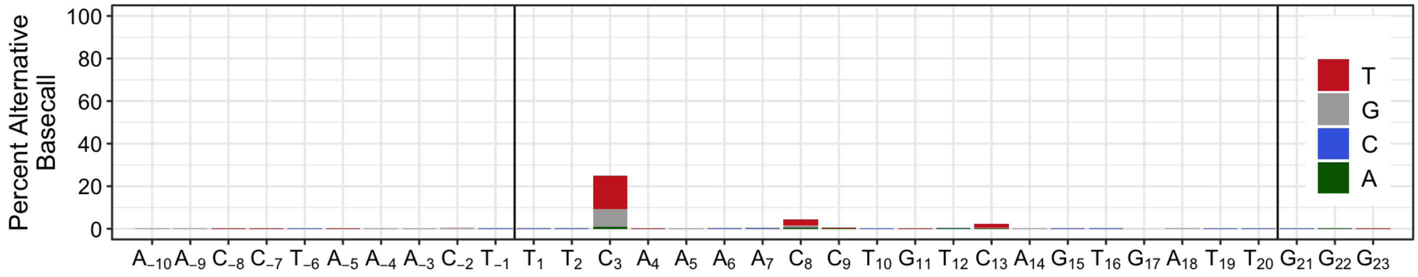

101. C TRAC Ex.3 pmSTOP_2 + BE3 unconc. mRNA 1.5 µg

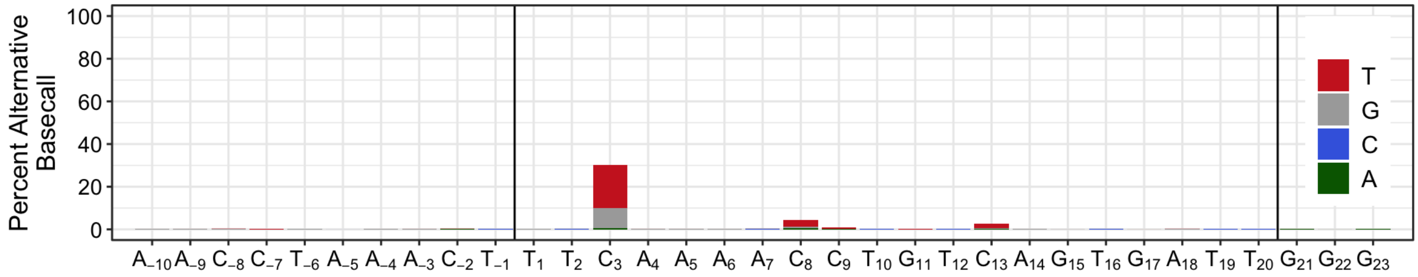

102. A TRAC Ex.3 pmSTOP_2 + BE4 unconc. mRNA 1.5 µg

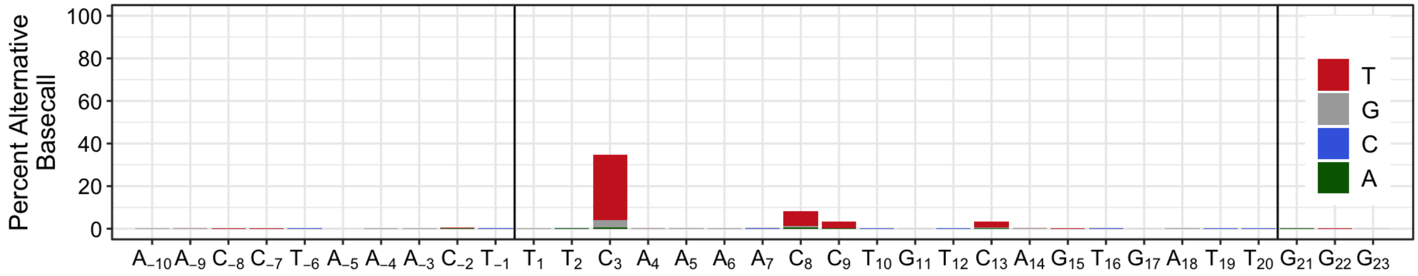

103. B TRAC Ex.3 pmSTOP_2 + BE4 unconc. mRNA 1.5 µg

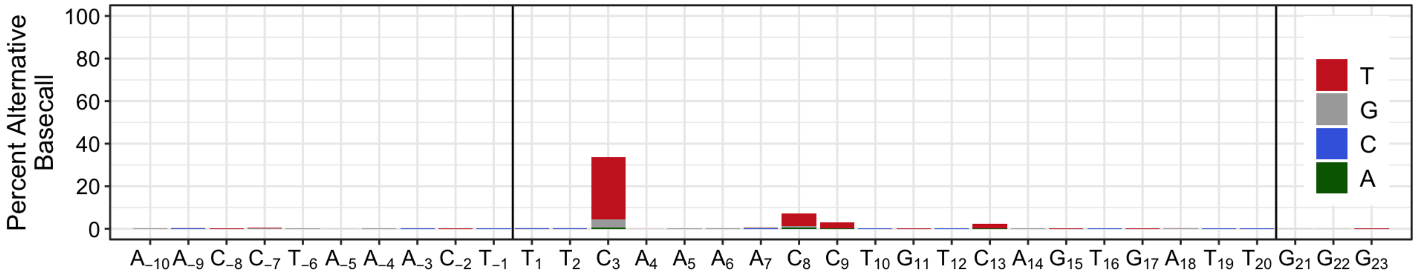

104. C TRAC Ex.3 pmSTOP_2 + BE4 unconc. mRNA 1.5 µg

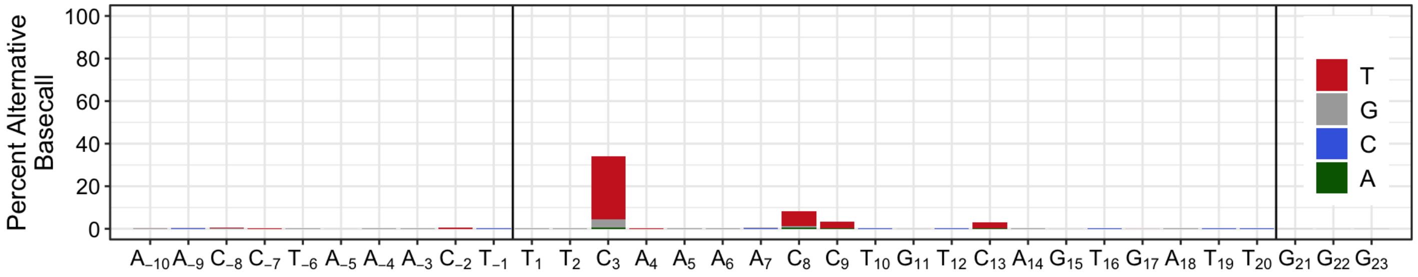

105. D TRAC Ex.3 pmSTOP_2 + Pulse

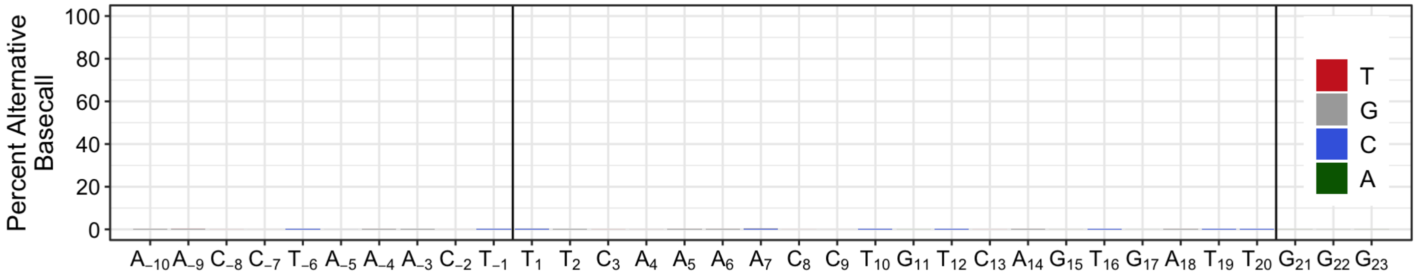

106. A TRAC Ex.3 pmSTOP_2 + Pulse

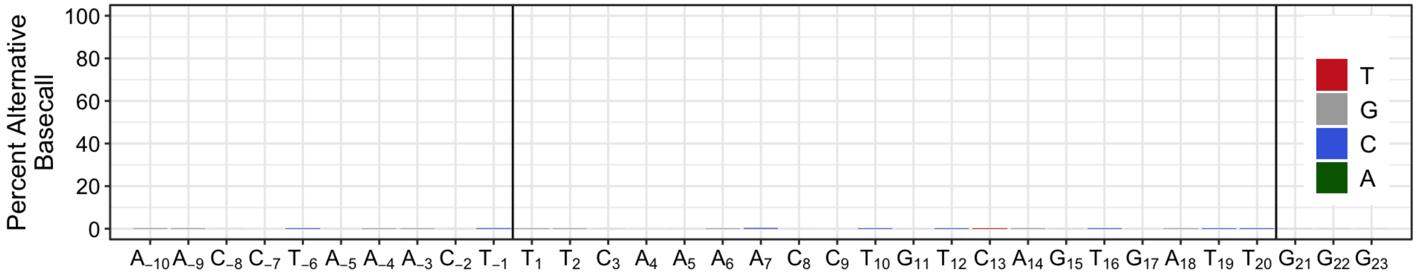

107. A B2M Ex.1 SD + BE3 unconc. mRNA 1.5 µg

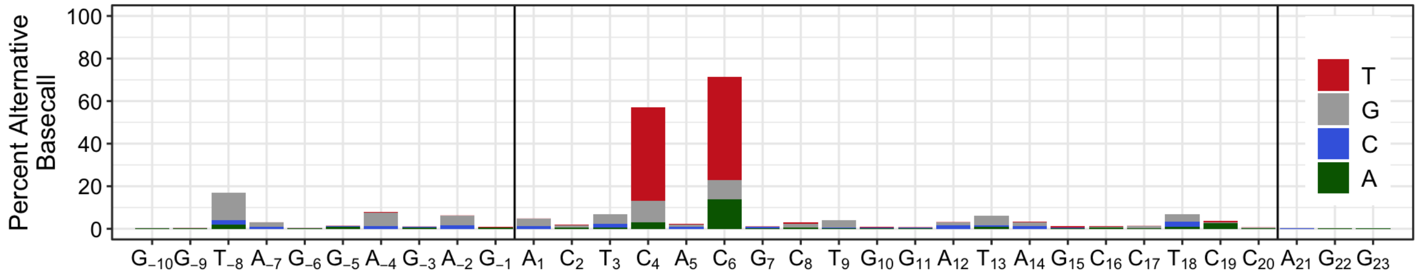

108. B B2M Ex.1 SD + BE3 unconc. mRNA 1.5 µg

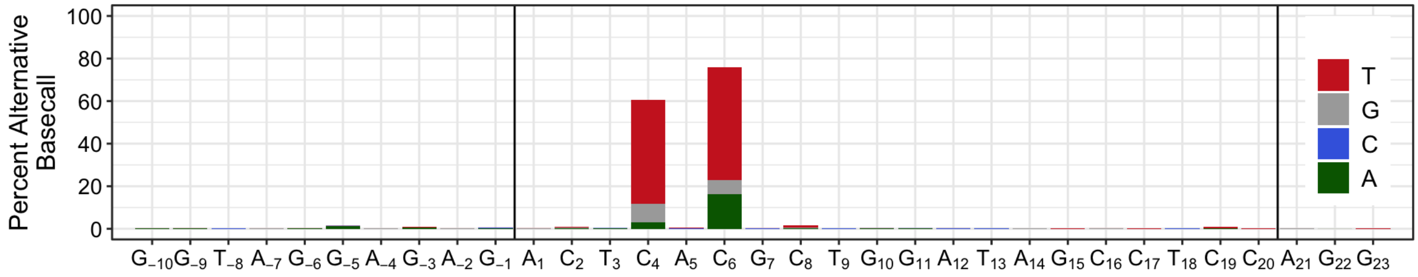

109. C B2M Ex.1 SD + BE3 unconc. mRNA 1.5 µg

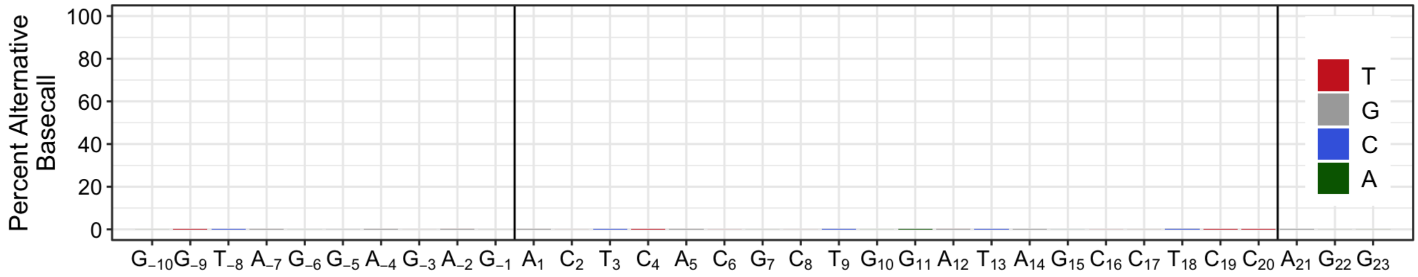

110. A B2M Ex.1 SD + BE4 unconc. mRNA 1.5 µg

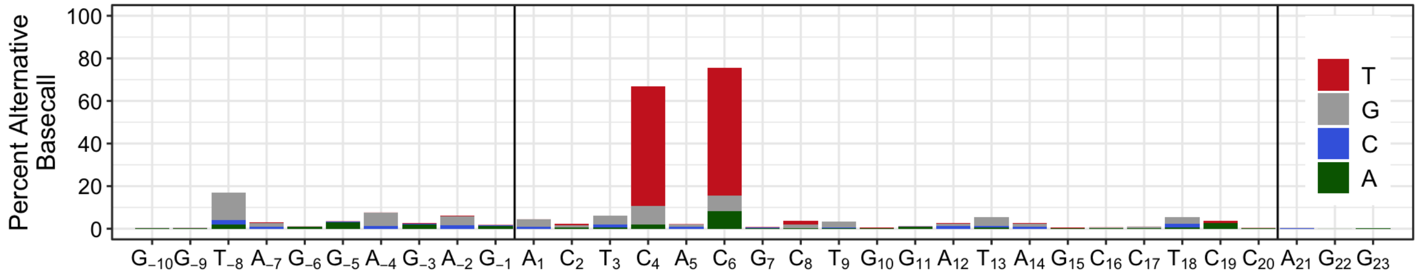

111. B B2M Ex.1 SD + BE4 unconc. mRNA 1.5 µg

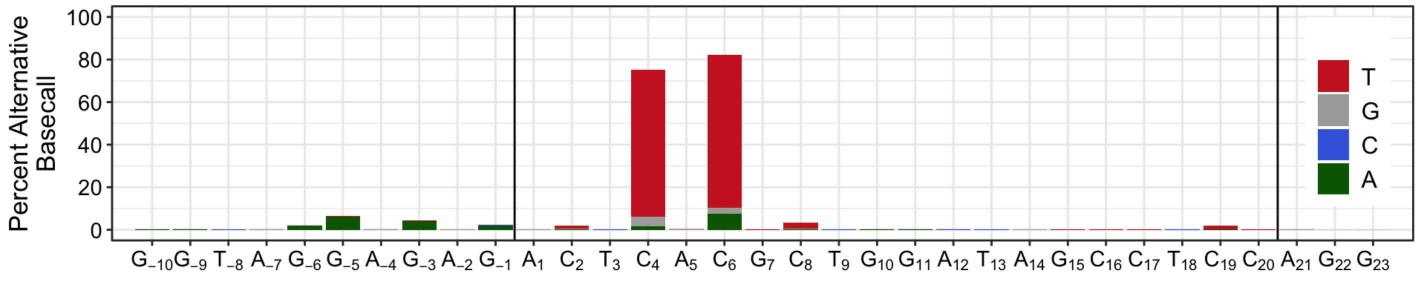

112. C B2M Ex.1 SD + BE4 unconc. mRNA 1.5 µg

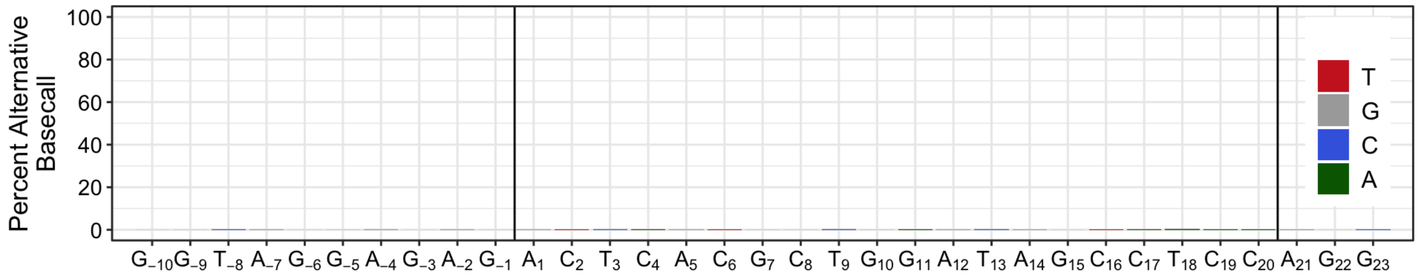

113. D B2M Ex.1 SD + BE4 unconc. mRNA 1.5 µg

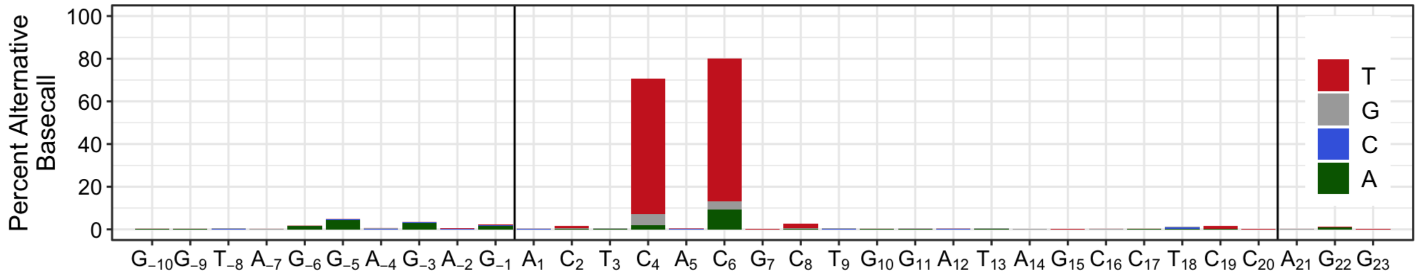

114. A B2M Ex.1 SD + Pulse

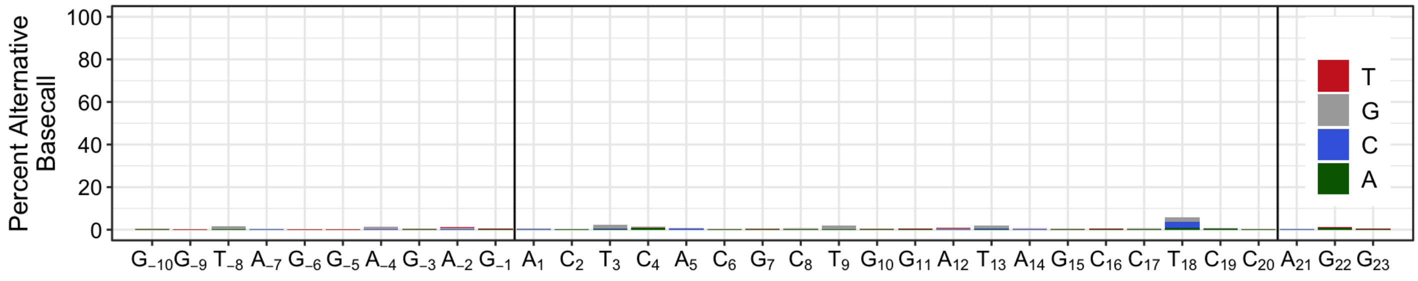

115. A B2M Ex.2 pmSTOP + BE3 unconc. mRNA 1.5 µg

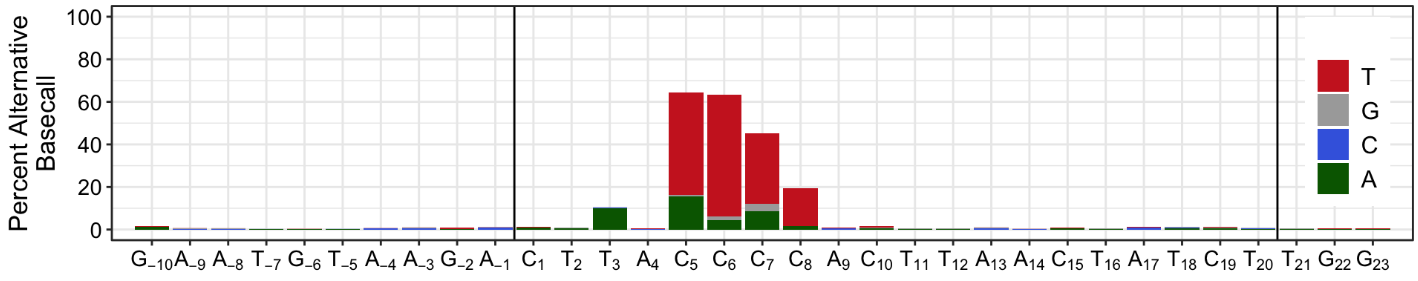

116. C B2M Ex.2 pmSTOP + BE3 unconc. mRNA 1.5 µg

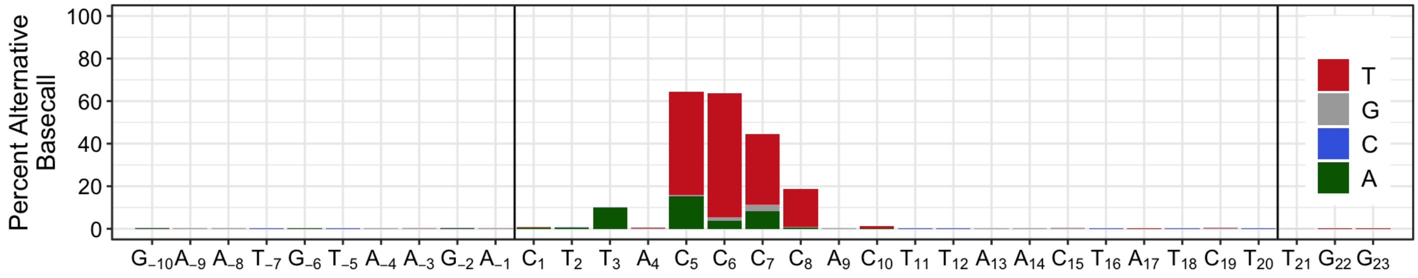

117. A B2M Ex.2 pmSTOP + BE4 unconc. mRNA 1.5 µg

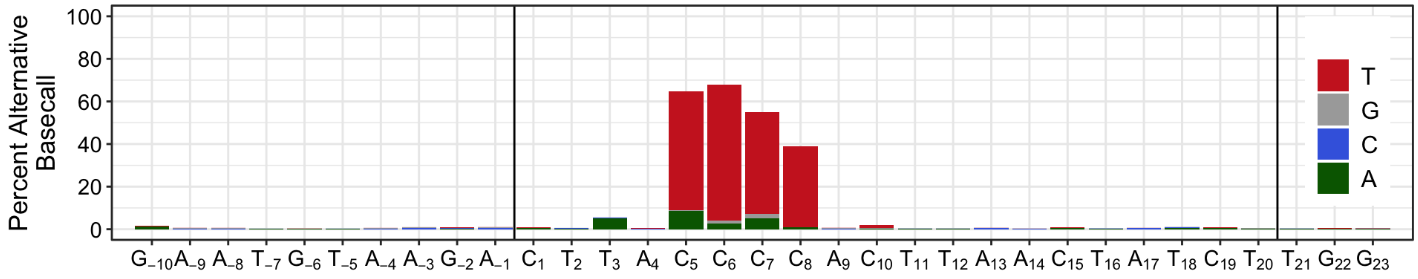

118. C B2M Ex.2 pmSTOP + BE4 unconc. mRNA 1.5 µg

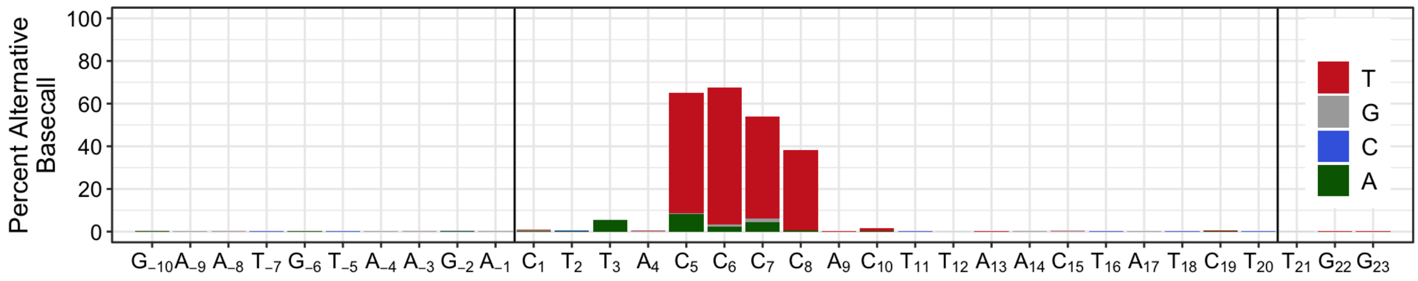

119. A B2M Ex.2 pmSTOP + Pulse

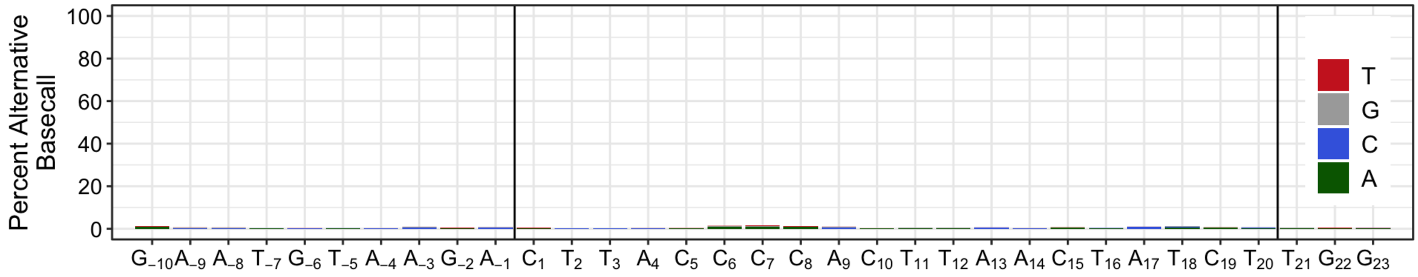

120. A B2M Ex.3 SA + BE3 unconc. mRNA 1.5 µg

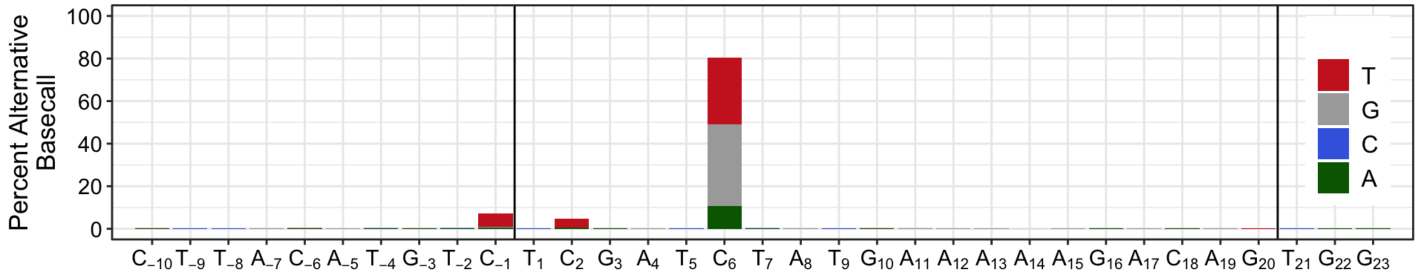

121. B B2M Ex.3 SA + BE3 unconc. mRNA 1.5 µg

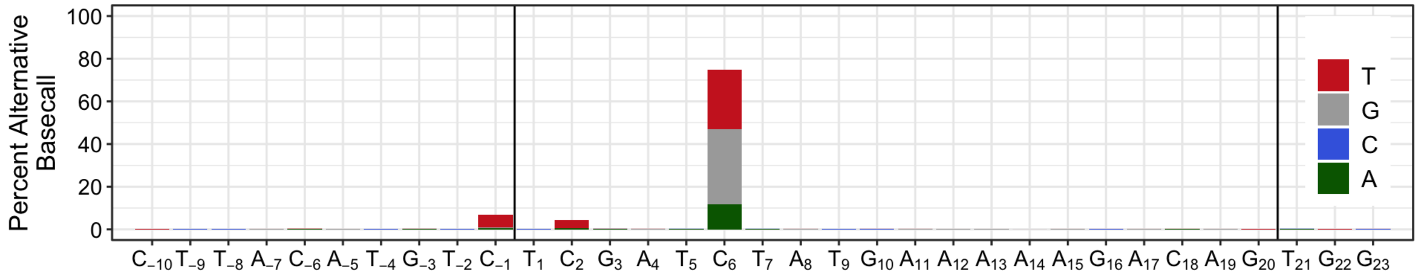

123. A B2M Ex.3 SA + BE4 unconc. mRNA 1.5 µg

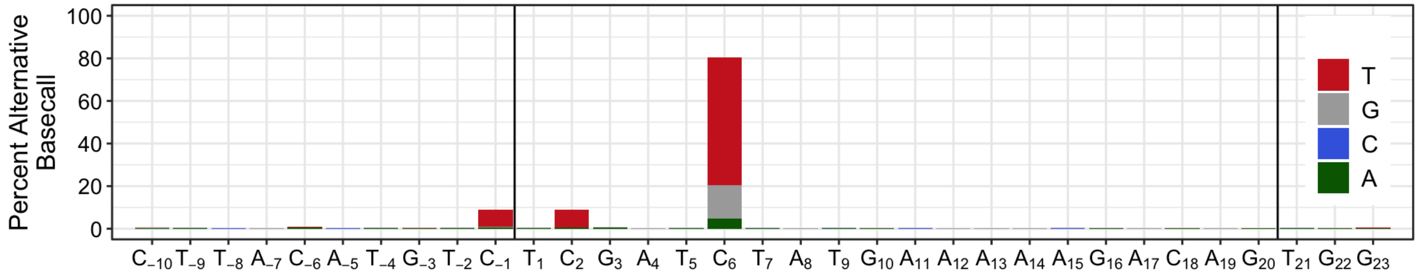

124. B B2M Ex.3 SA + BE4 unconc. mRNA 1.5 µg

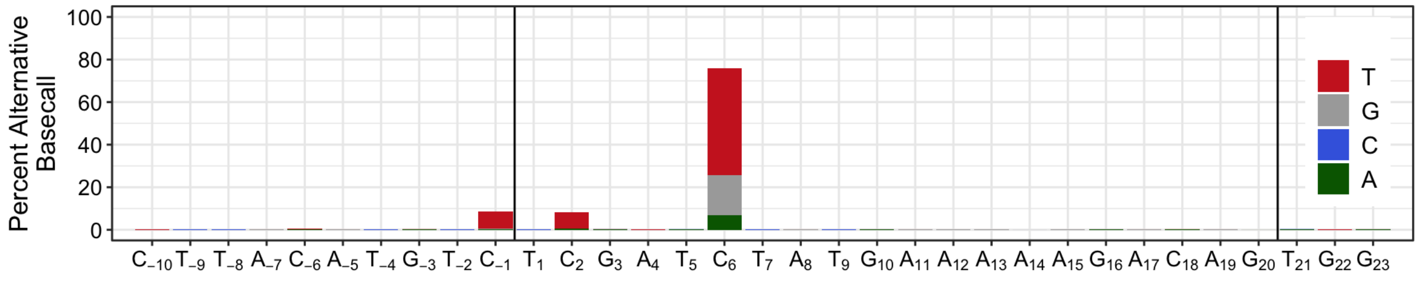

125. C B2M Ex.3 SA + BE4 unconc. mRNA 1.5 µg

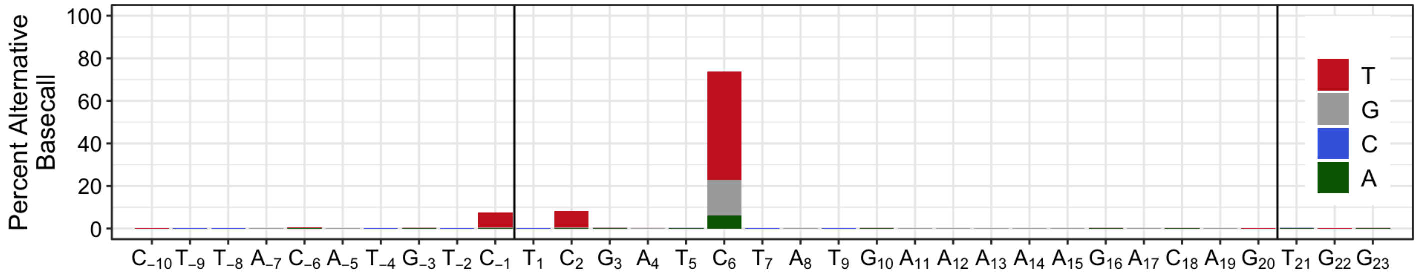

126. A 3G TRAC Ex.3 SA + Pulse

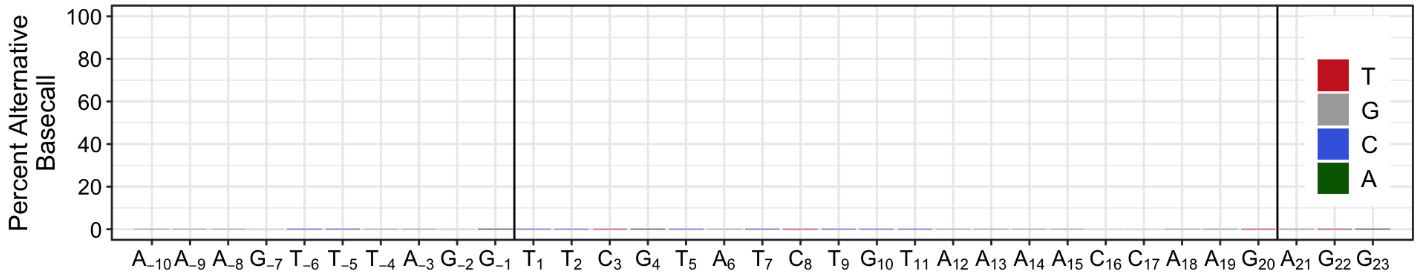

127. A 3G TRAC Ex.3 SA + Pulse

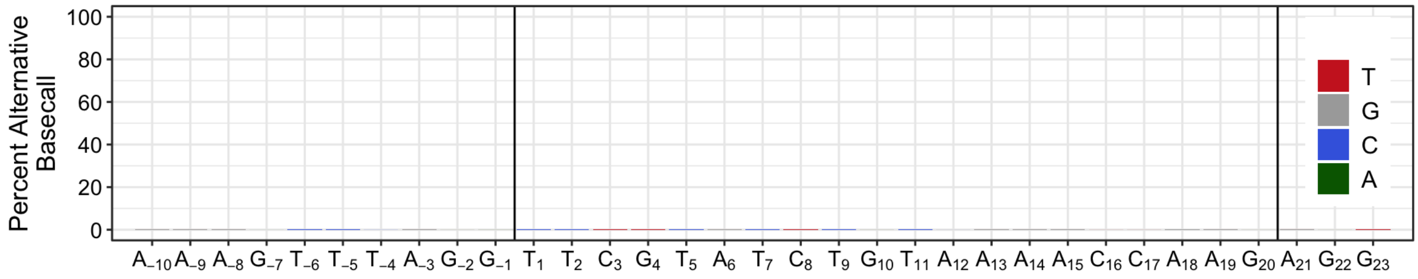

128. B 3G TRAC Ex.3 SA + Pulse

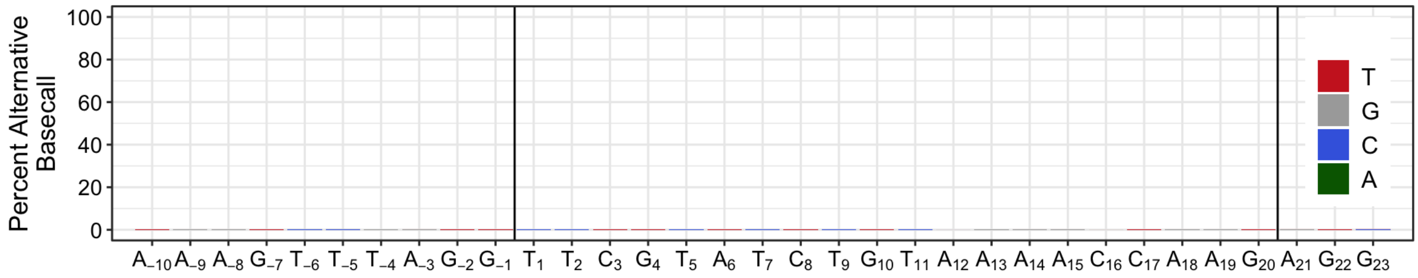

129. C 3G TRAC Ex.3 SA + Pulse

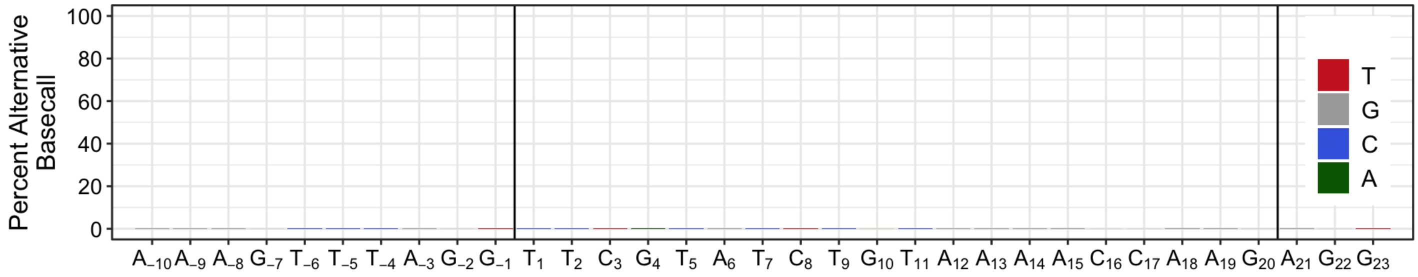

130. A 3G TRAC Ex.3 SA + Pulse

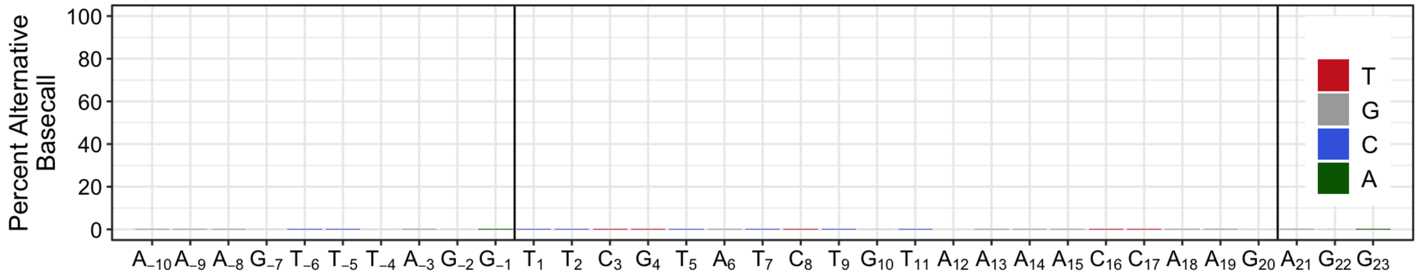

131. B 3G TRAC Ex.3 SA + Pulse

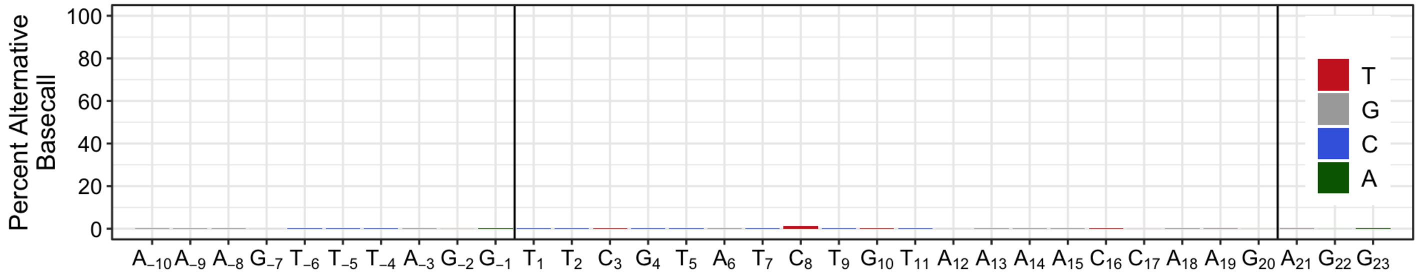

132. A 3G TRAC Ex.3 SA + Pulse

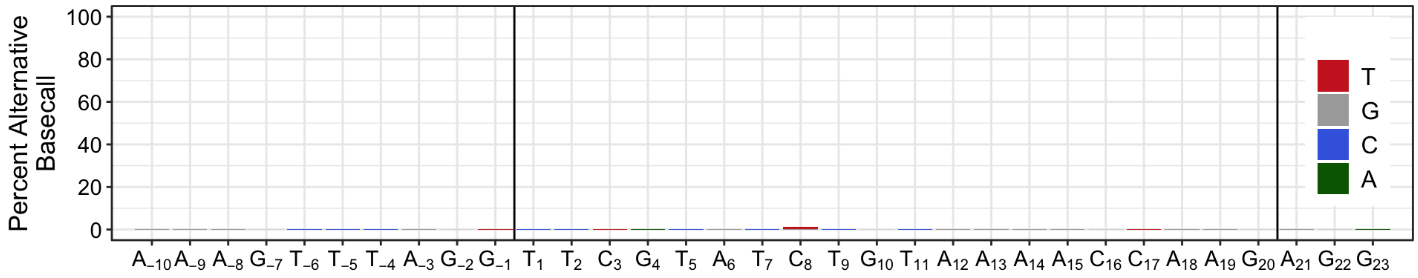

133. B 3G TRAC Ex.3 SA + Pulse

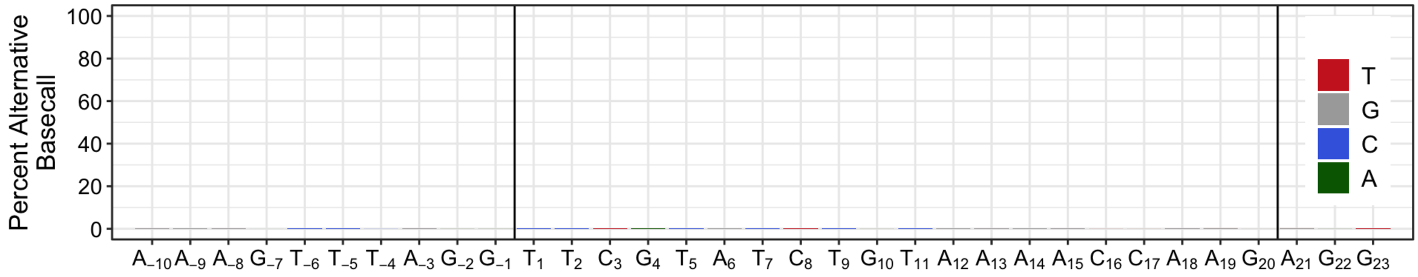

134. A 3G TRAC Ex.3 SA + BE3 conc. mRNA 3 μg

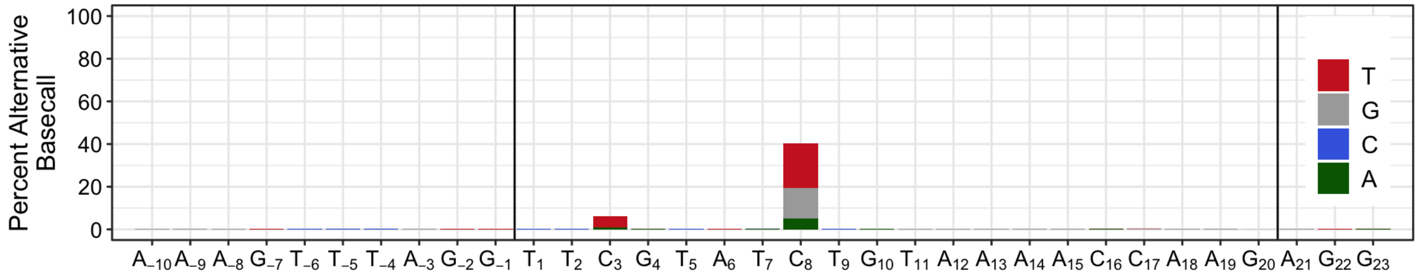

135. B 3G TRAC Ex.3 SA + BE3 conc. mRNA 3 μg

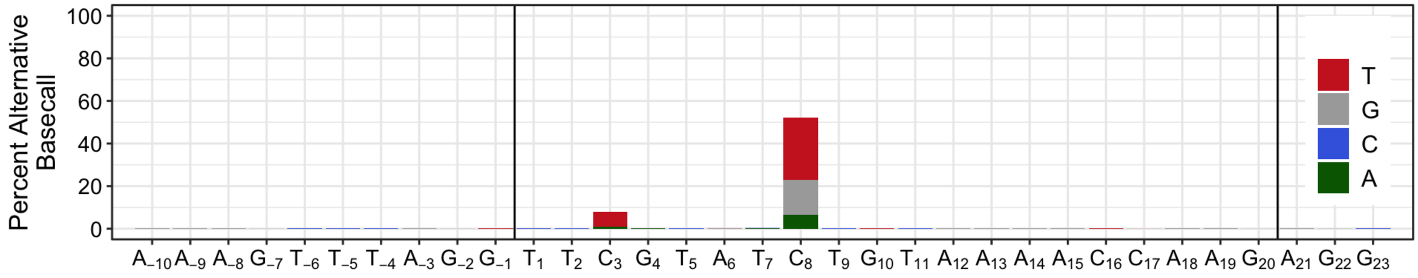

136. A 3G TRAC Ex.3 SA + BE4 conc. mRNA 3 µg

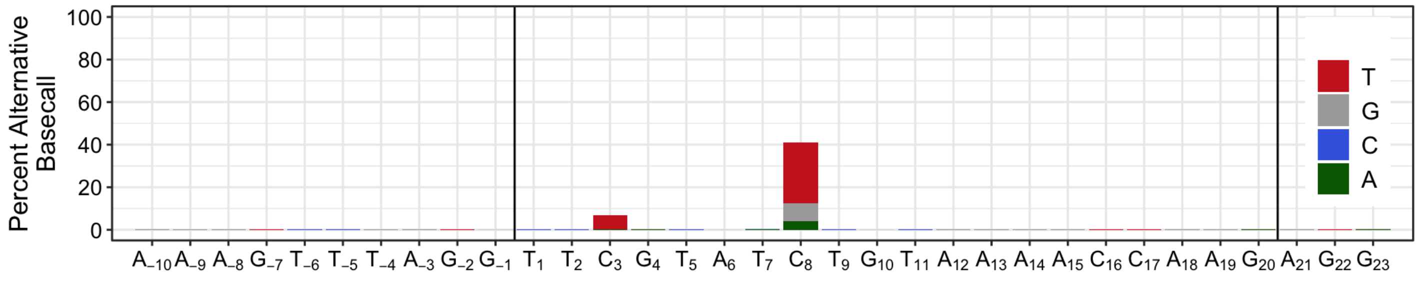

137. B 3G TRAC Ex.3 SA + BE4 conc. mRNA 3 µg

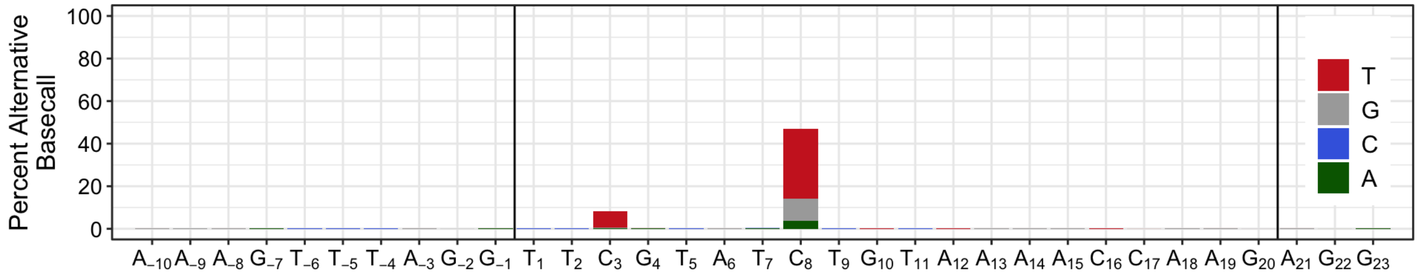

138. A 3G TRAC Ex.3 SA + BE4 RNP

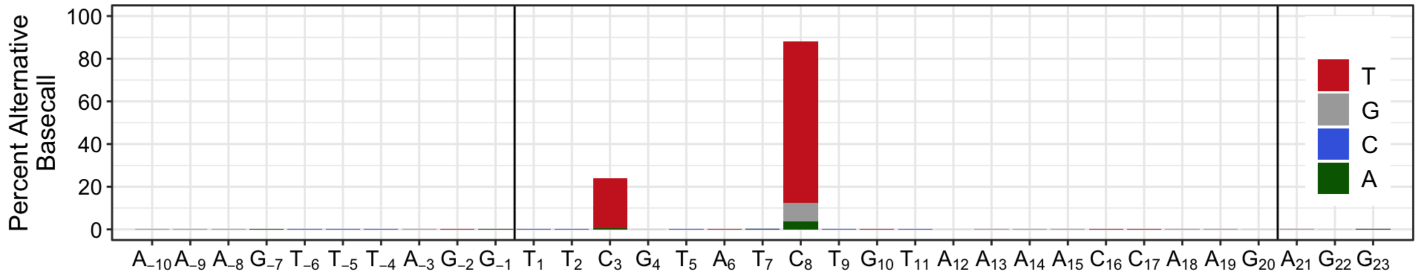

139. B 3G TRAC Ex.3 SA + BE4 RNP

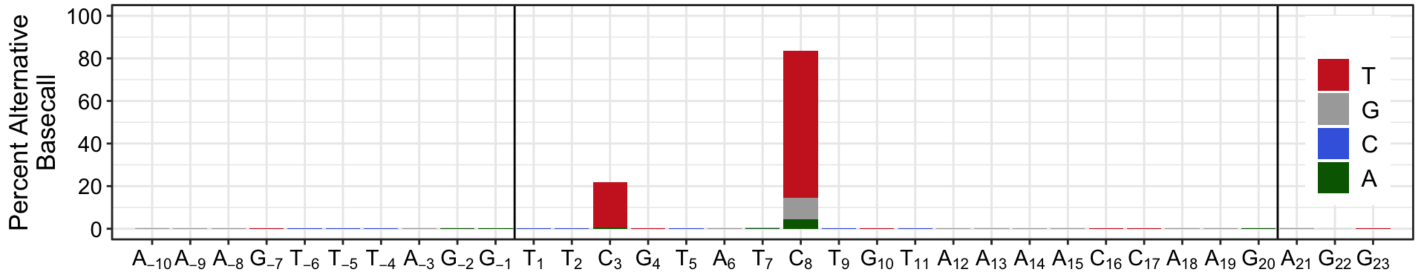

140. A 3G TRAC Ex.3 SA + coBE4 mRNA 1.5 µg

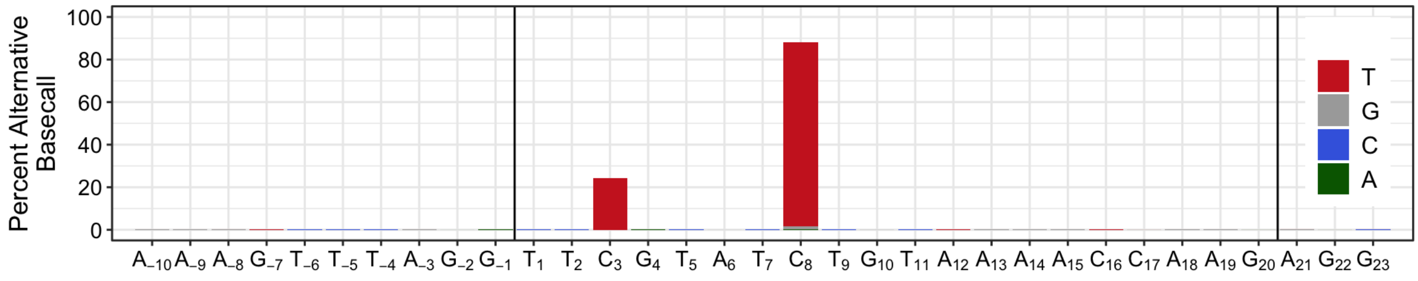

141. B 3G TRAC Ex.3 SA + coBE4 mRNA 1.5 µg

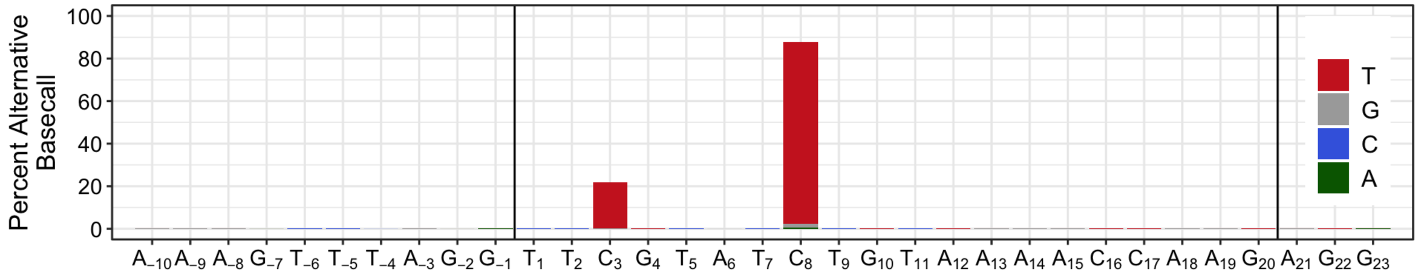

142. A 3G TRAC Ex.3 SA + coBE4 mRNA 4 µg

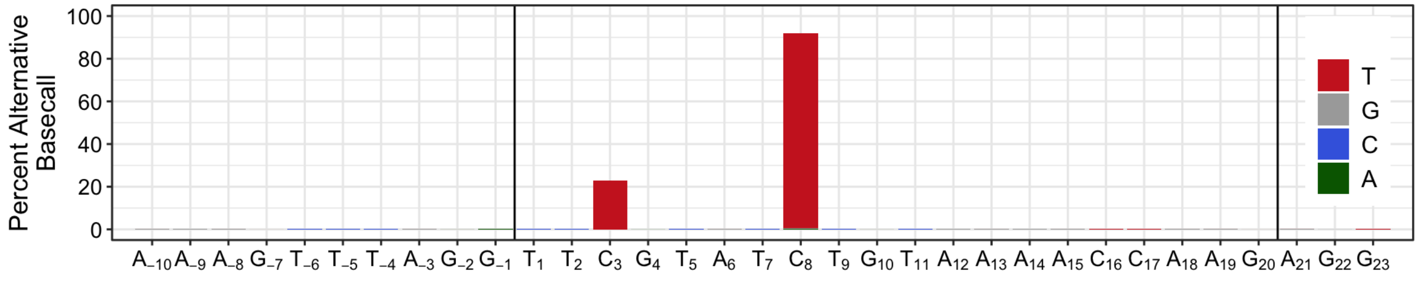

143. A 3G TRAC Ex.3 SA + coBE4 mRNA 4 µg

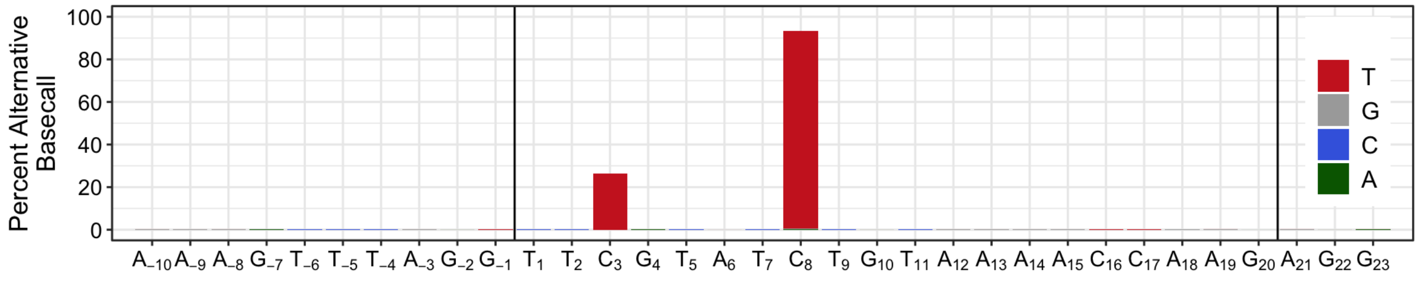

144. B 3G TRAC Ex.3 SA + coBE4 mRNA 4 μg

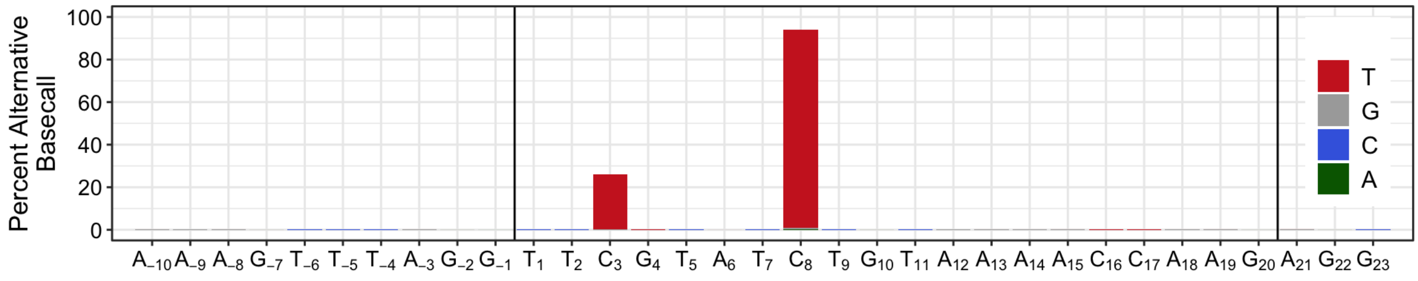

145. B 3G TRAC Ex.3 SA + coBE4 mRNA 4 μg

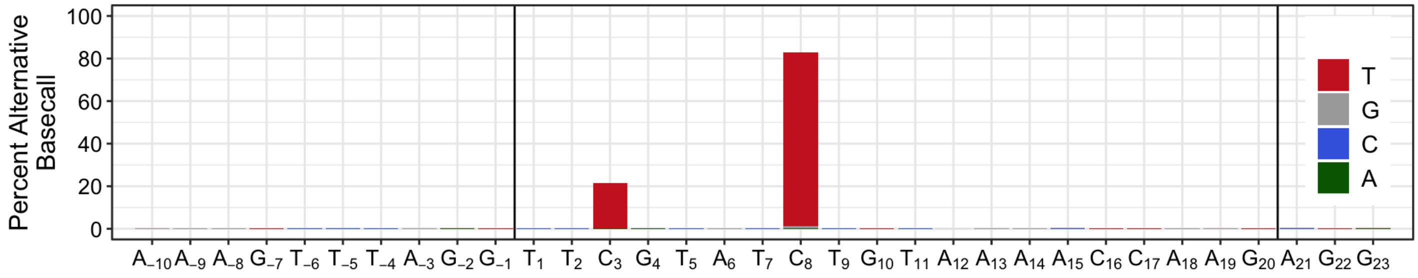

146. C 3G TRAC Ex.3 SA + coBE4 mRNA 4 μg

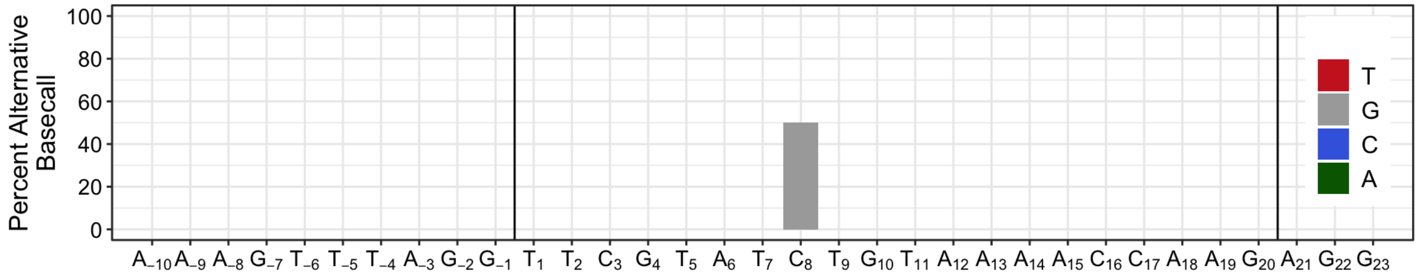

147. A 3G TRAC Ex.3 SA + Cas9 mRNA 1.5 μg

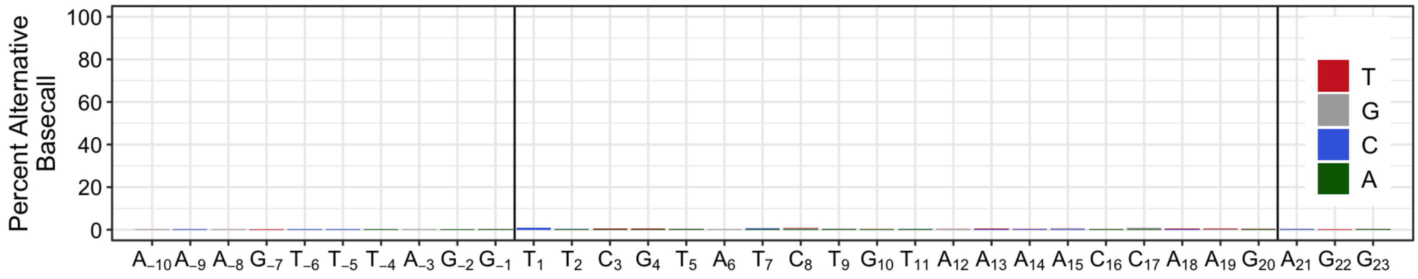

148. B 3G TRAC Ex.3 SA + Cas9 mRNA 1.5 µg

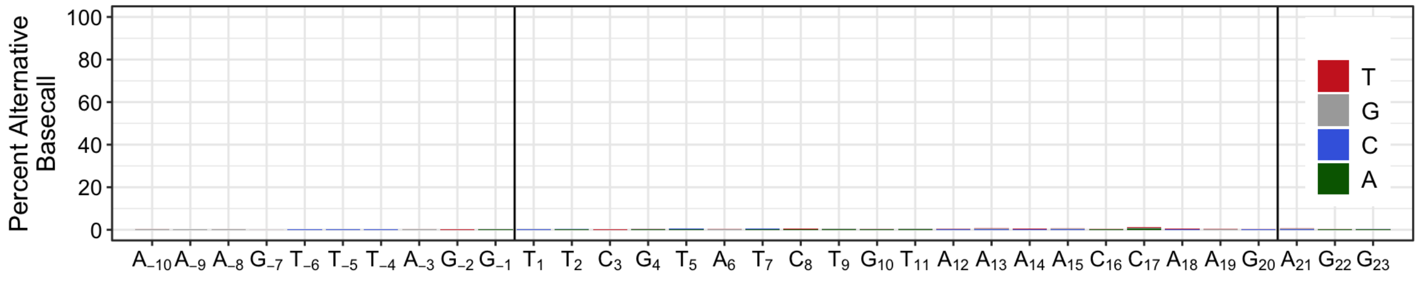

149. A 3G TRAC Ex.3 SA + Cas9 mRNA 1.5 µg

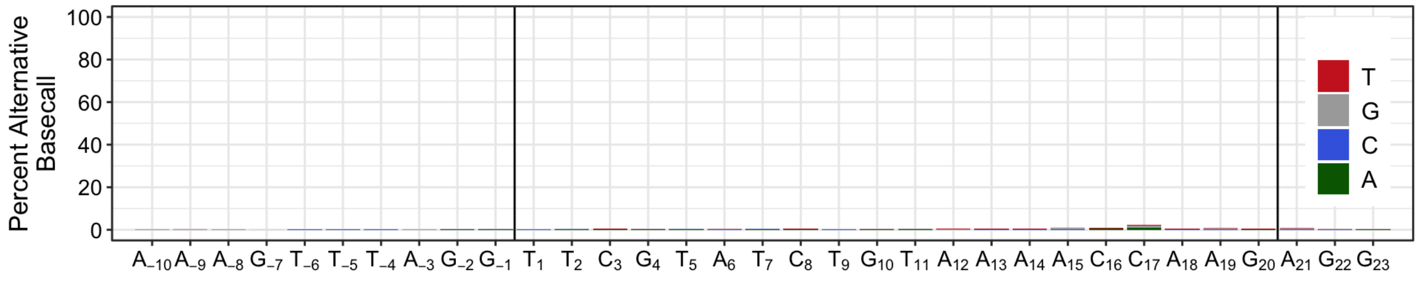

150. B 3G TRAC Ex.3 SA + Cas9 mRNA 1.5 µg

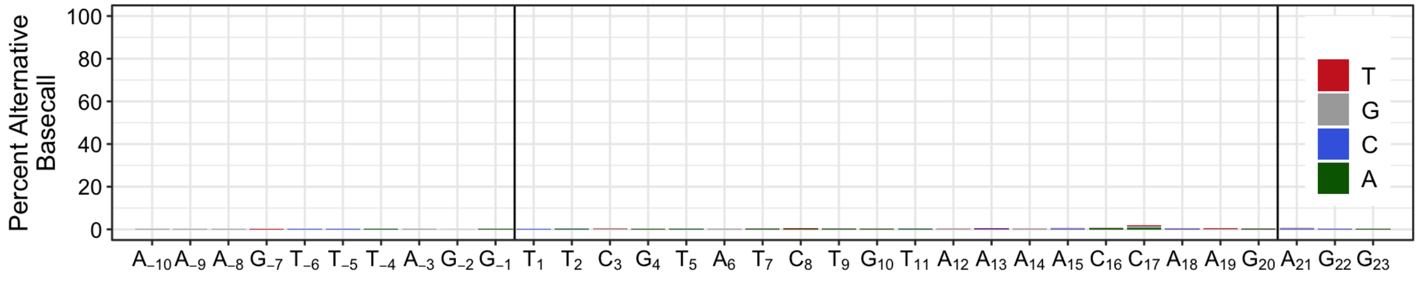

151. A 3G TRAC Ex.3 SA + Cas9 mRNA 4 µg

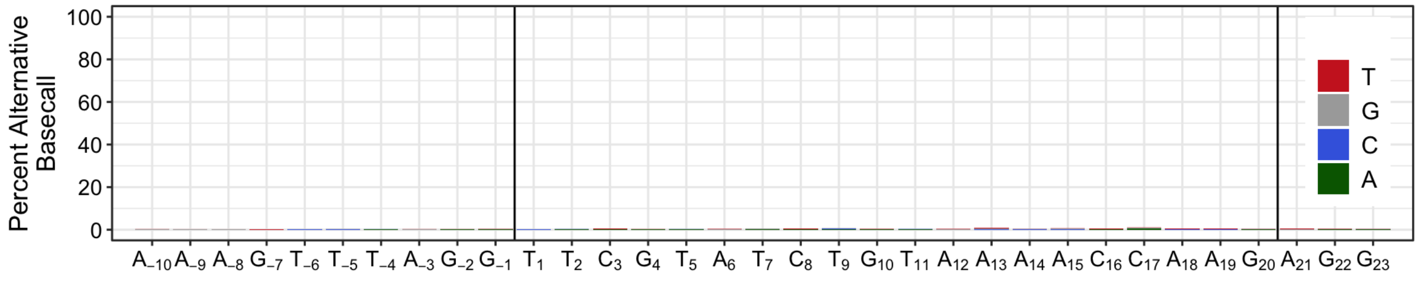

152. B 3G TRAC Ex.3 SA + Cas9 mRNA 4 µg

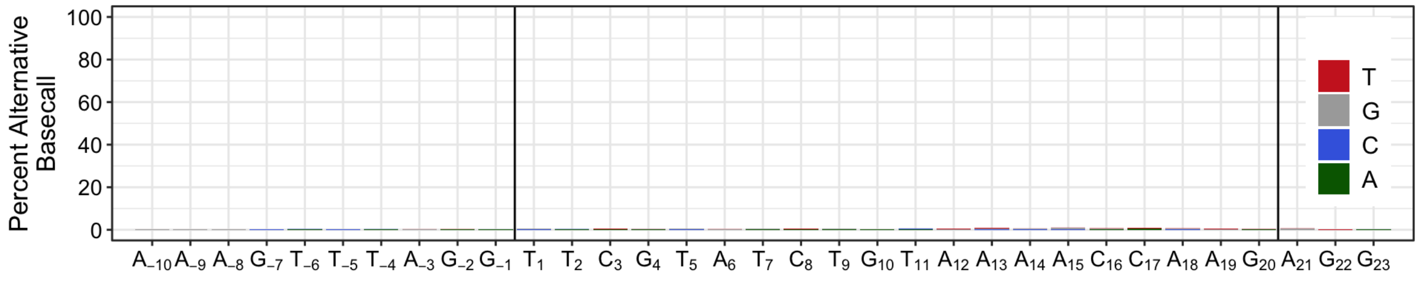

153. A 3G TRAC Ex.3 SA + Cas9 mRNA 4 µg

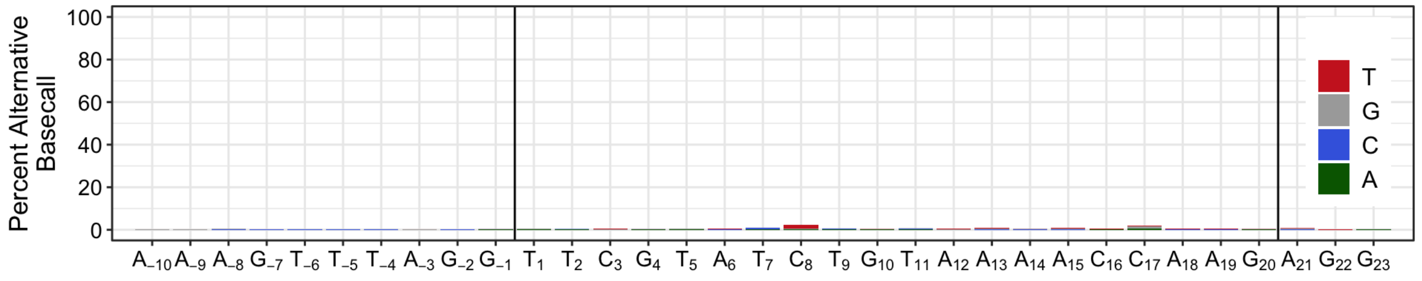

154. B 3G TRAC Ex.3 SA + Cas9 mRNA 4 µg

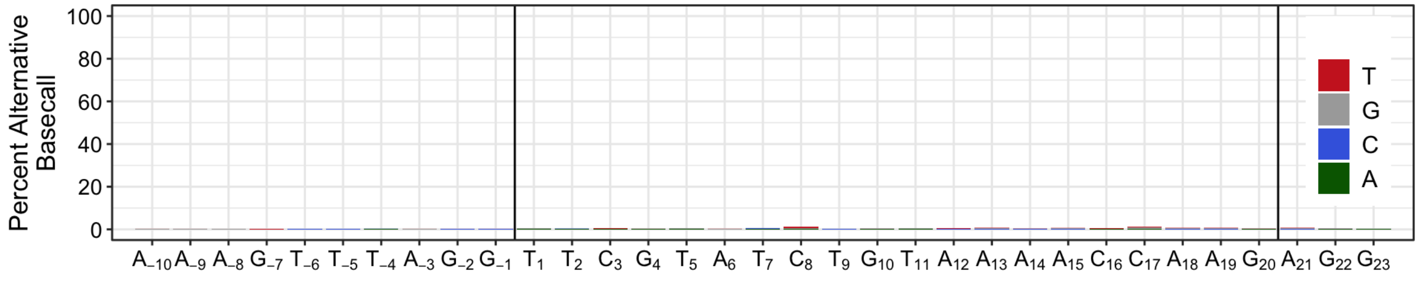

155. A 3G TRAC Ex.3 SA + Cas9 RNP

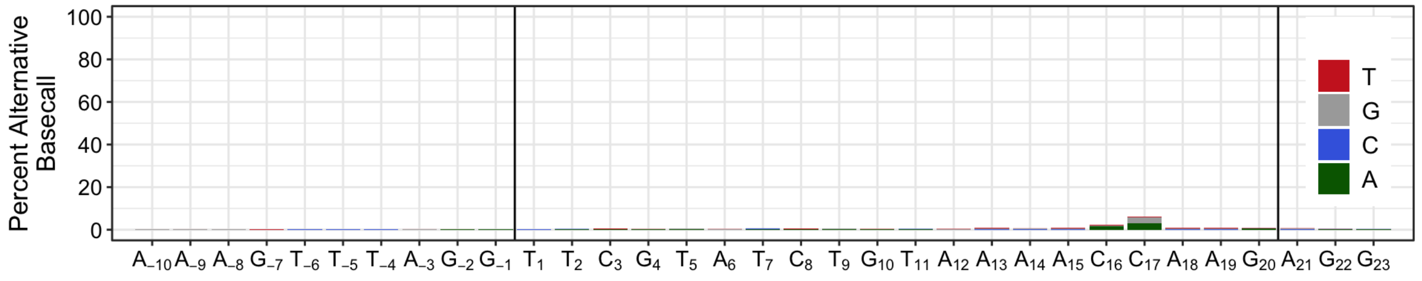

156. B 3G TRAC Ex.3 SA + Cas9 RNP

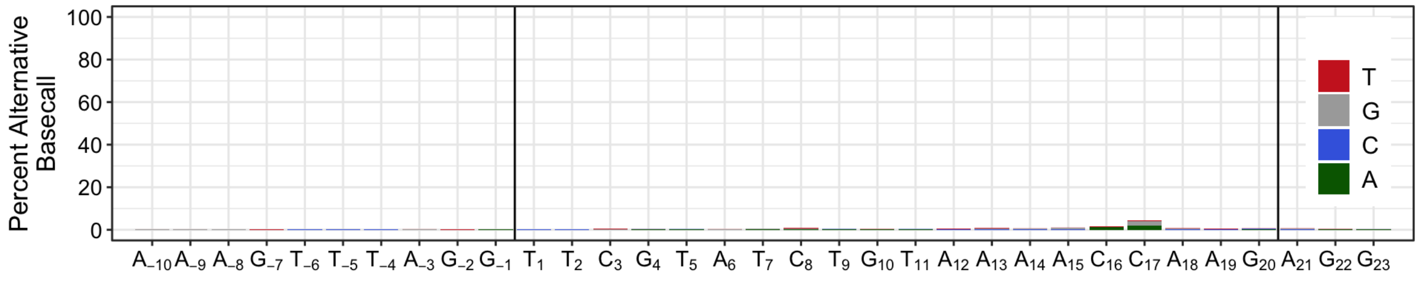

157. A 3G PDCD1 Ex.1 SD + Pulse

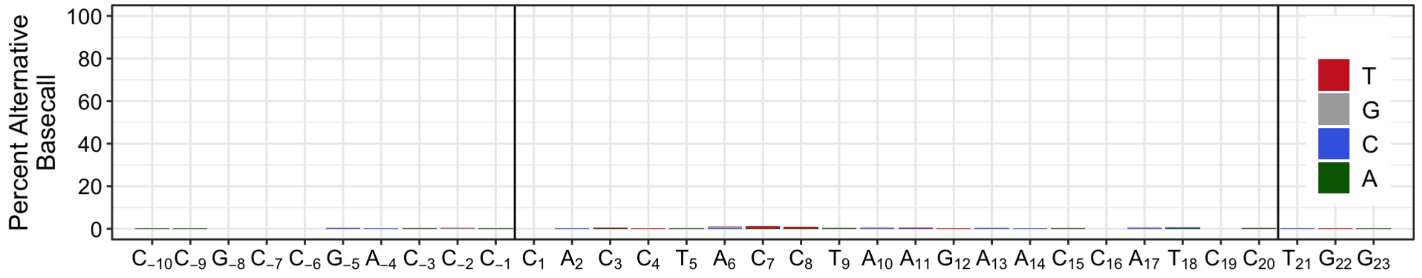

158. B 3G PDCD1 Ex.1 SD + Pulse

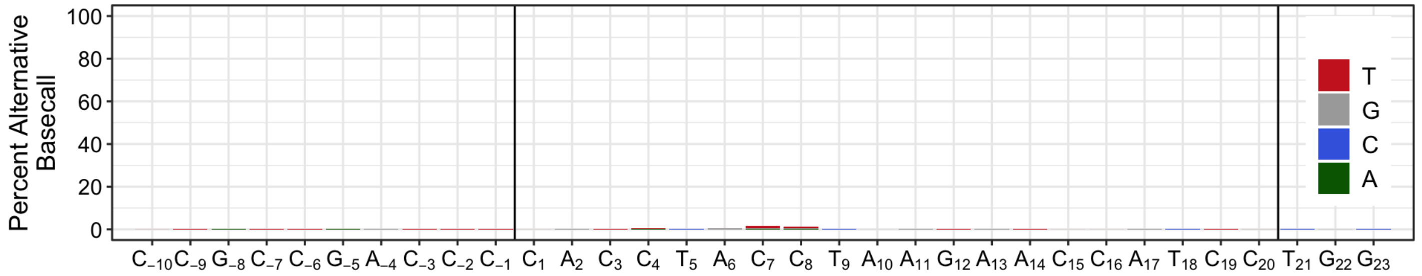

159. A 3G PDCD1 Ex.1 SD + Pulse

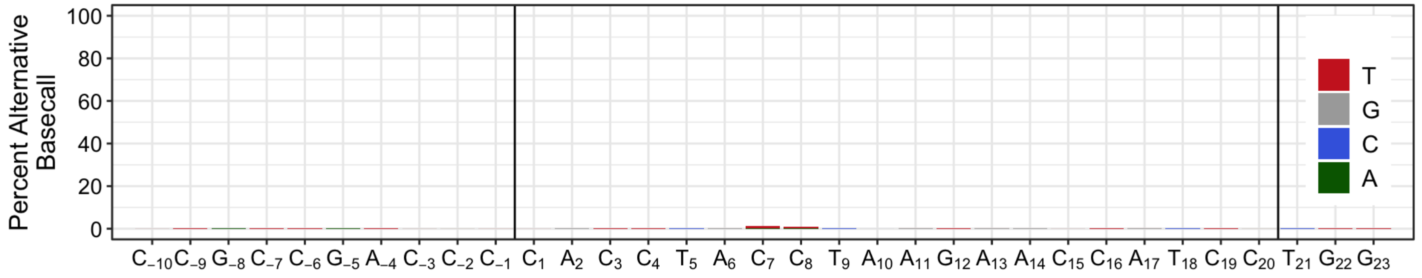

160. B 3G PDCD1 Ex.1 SD + Pulse

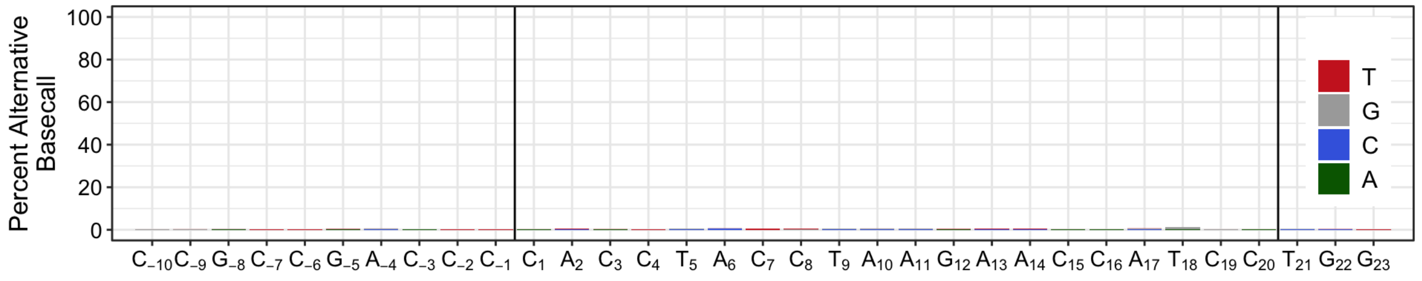

161. C 3G PDCD1 Ex.1 SD + Pulse

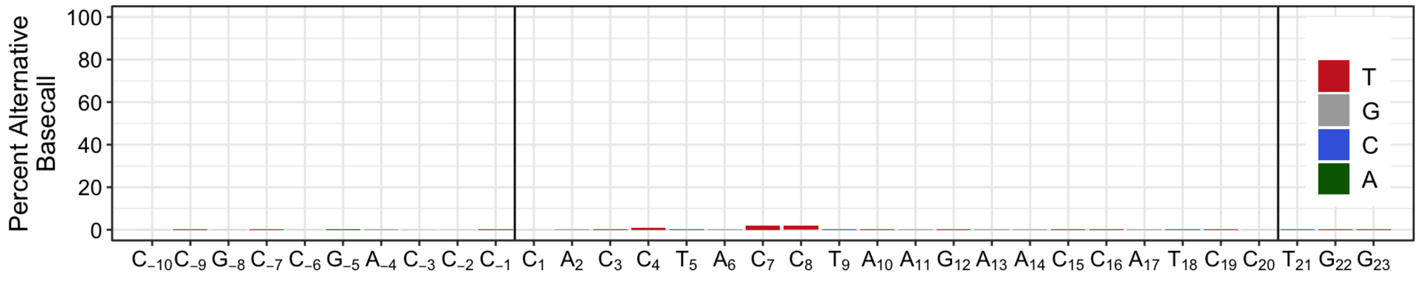

162. A 3G PDCD1 Ex.1 SD + Pulse

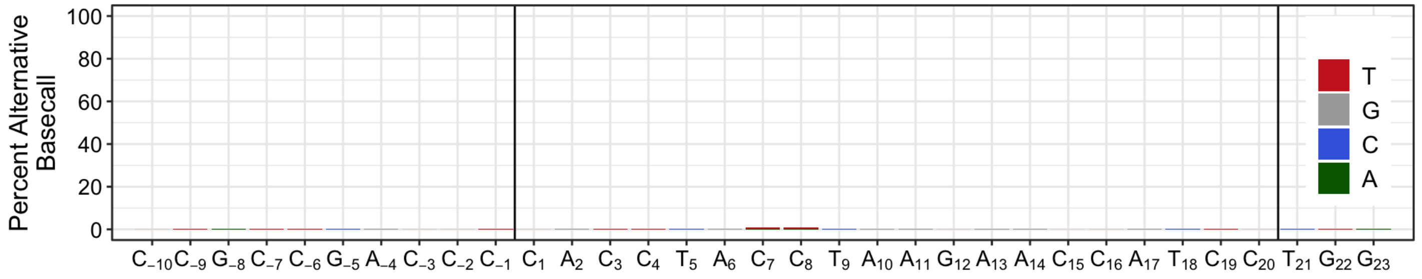

163. A 3G PDCD1 Ex.1 SD + Pulse

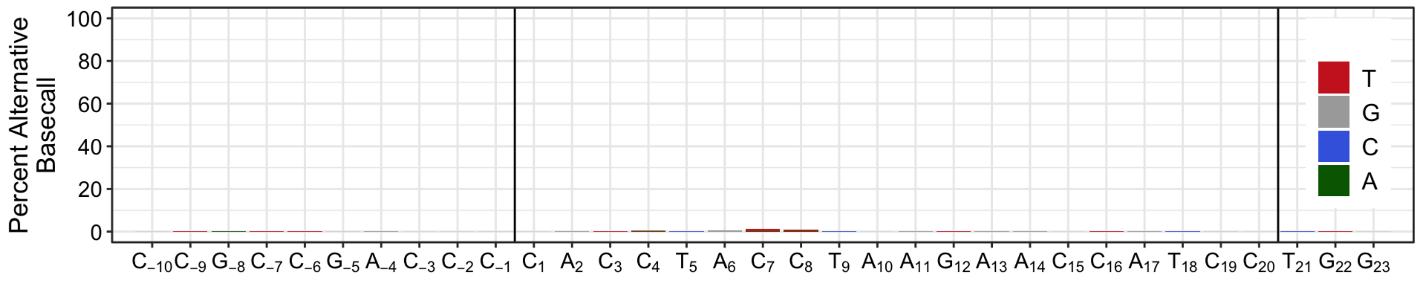

164. B 3G PDCD1 Ex.1 SD + Pulse

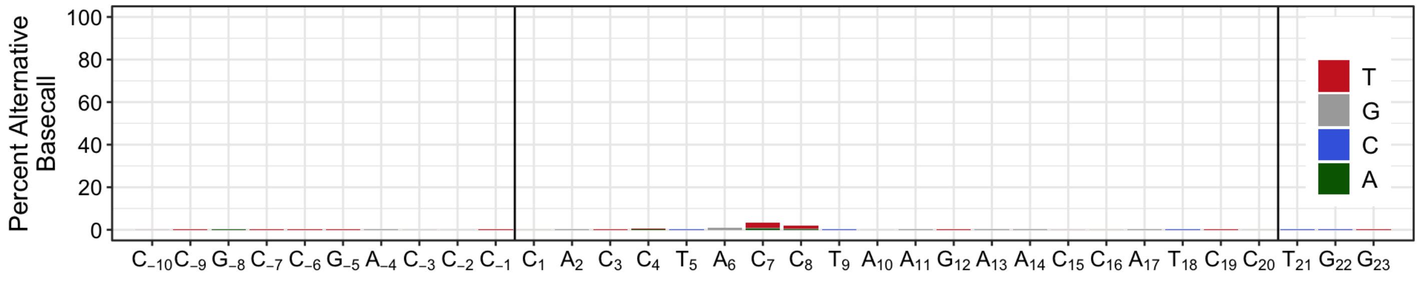

165. A 3G PDCD1 Ex.1 SD + Pulse

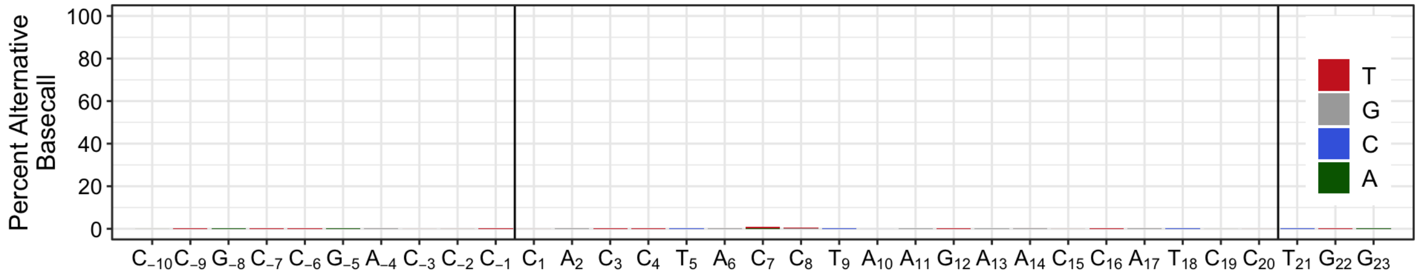

166. B 3G PDCD1 Ex.1 SD + Pulse

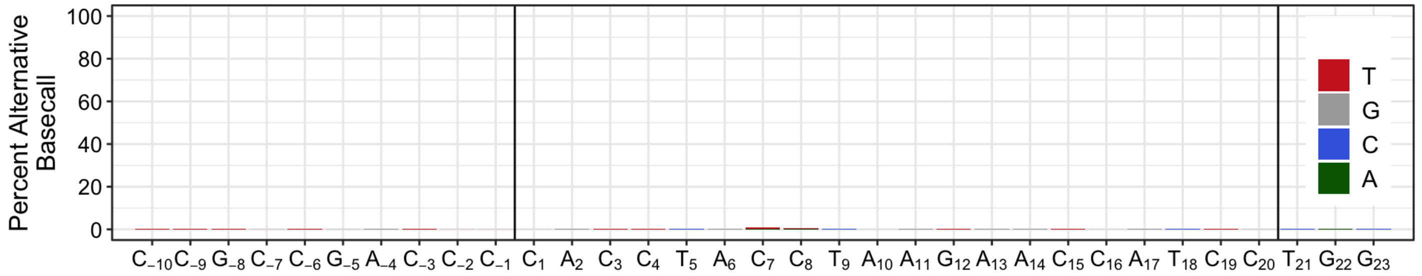

167. A 3G PDCD1 Ex.1 SD + BE3 conc. mRNA 3 µg

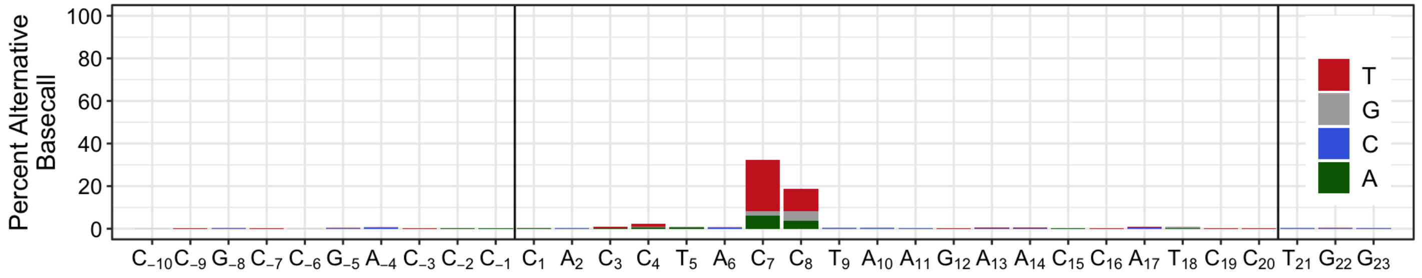

168. B 3G PDCD1 Ex.1 SD + BE3 conc. mRNA 3 μg

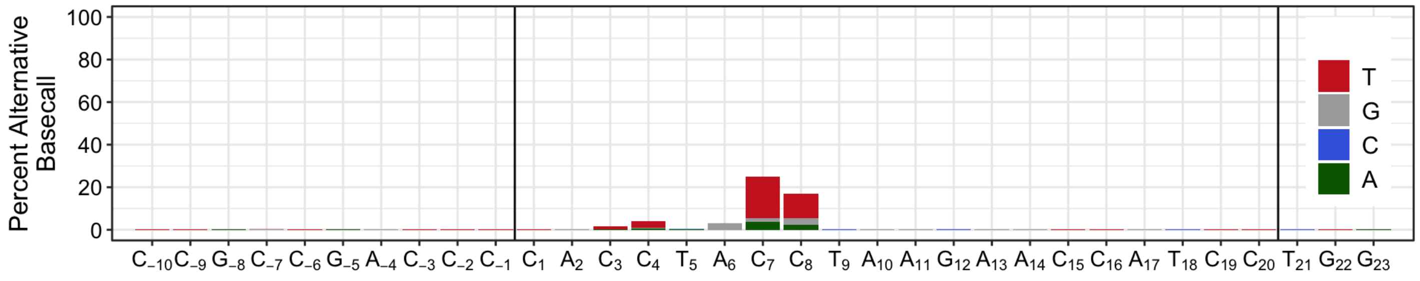

169. A 3G PDCD1 Ex.1 SD + BE4 conc. mRNA 3 μg

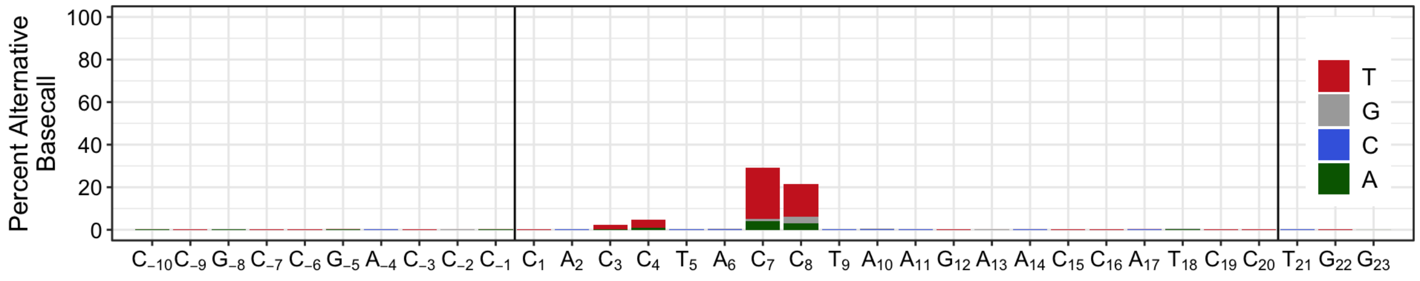

170. B 3G PDCD1 Ex.1 SD + BE4 conc. mRNA 3 μg

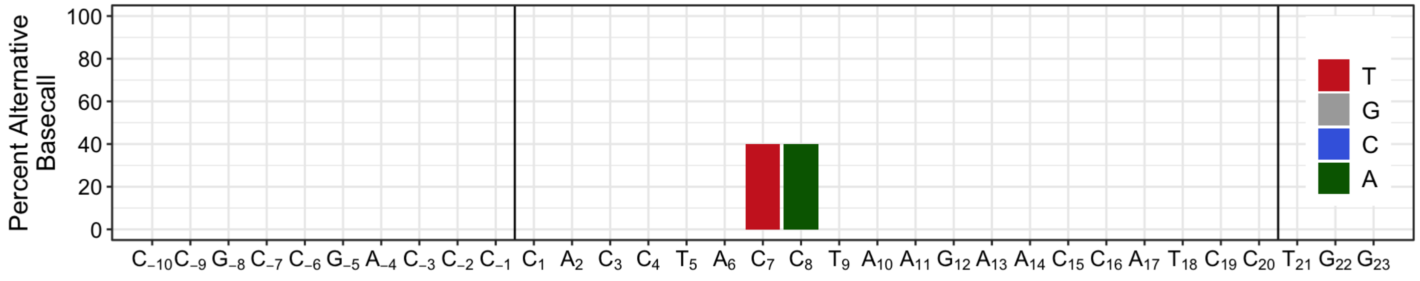

171. A 3G PDCD1 Ex.1 SD + BE4 RNP

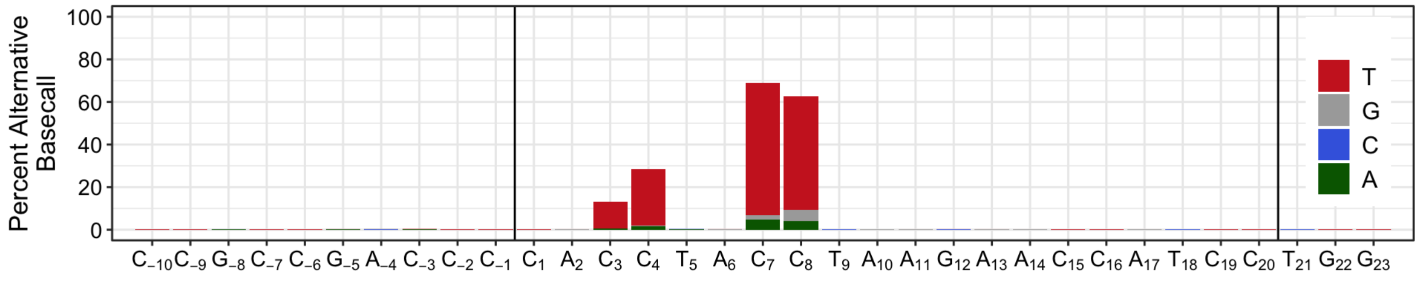

172. B 3G PDCD1 Ex.1 SD + BE4 RNP

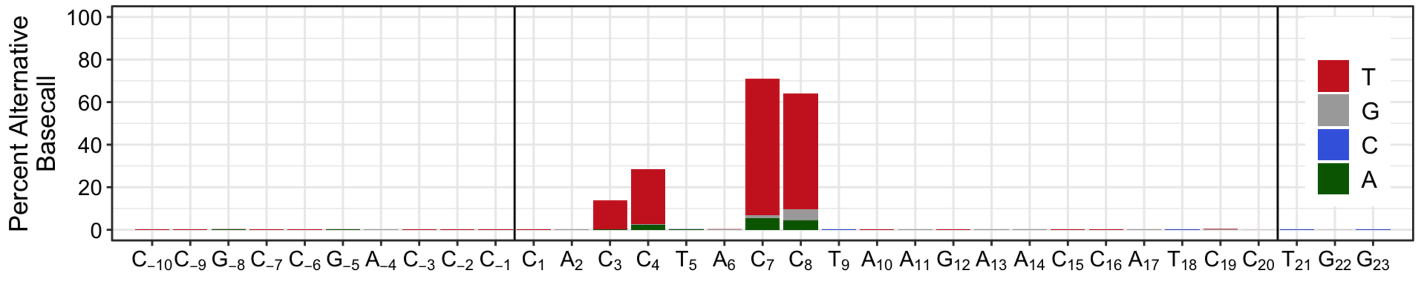

173. A 3G PDCD1 Ex.1 SD + coBE4 mRNA 1.5 µg

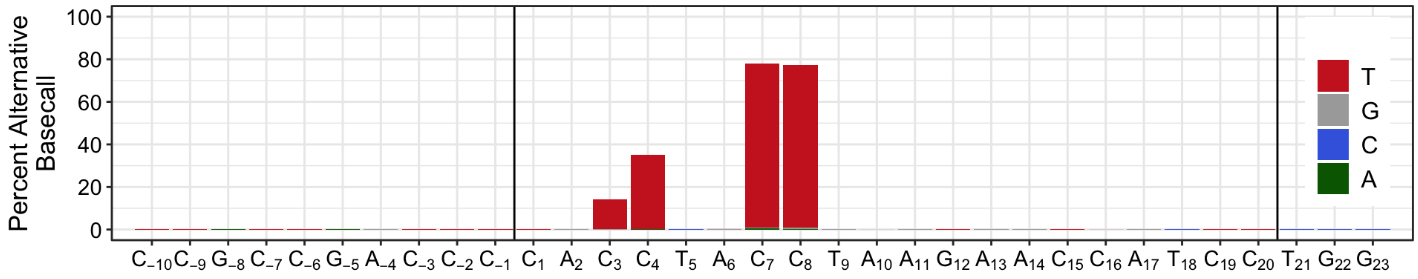

174. B 3G PDCD1 Ex.1 SD + coBE4 mRNA 1.5 µg

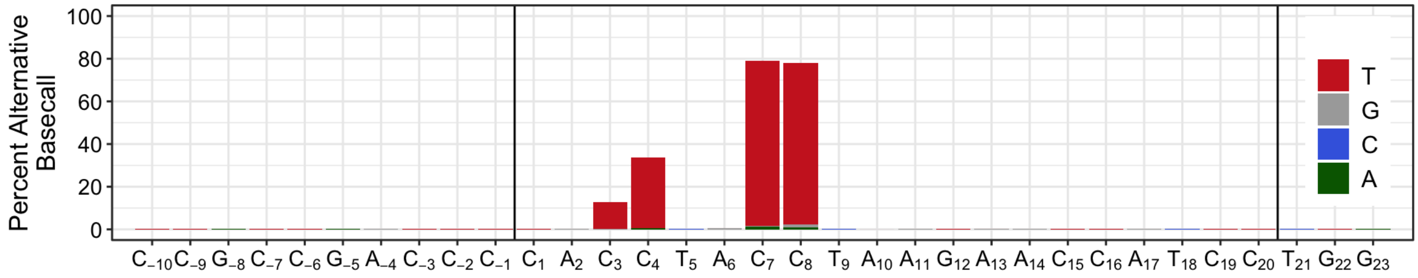

175. A 3G PDCD1 Ex.1 SD + coBE4 mRNA 4 µg

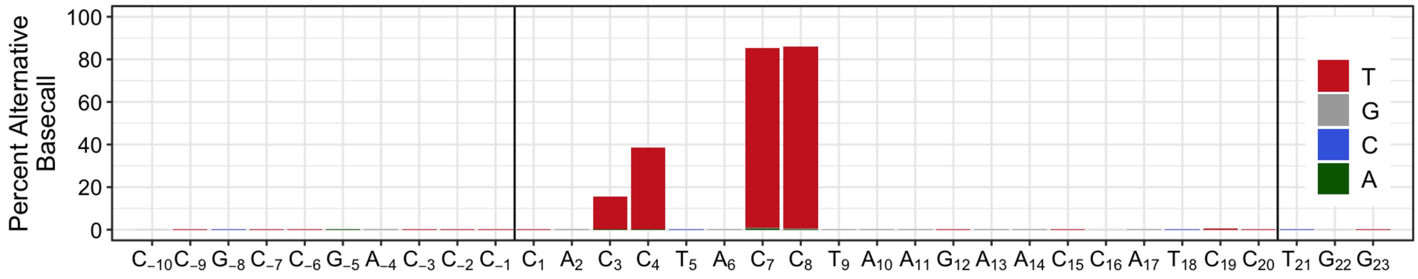

176. B 3G PDCD1 Ex.1 SD + coBE4 mRNA 4 μg

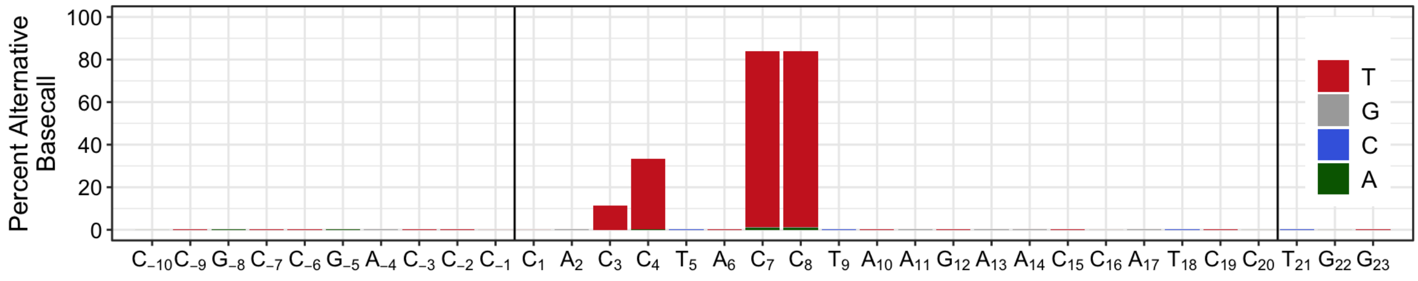

177. A 3G PDCD1 Ex.1 SD + coBE4 mRNA 4 μg

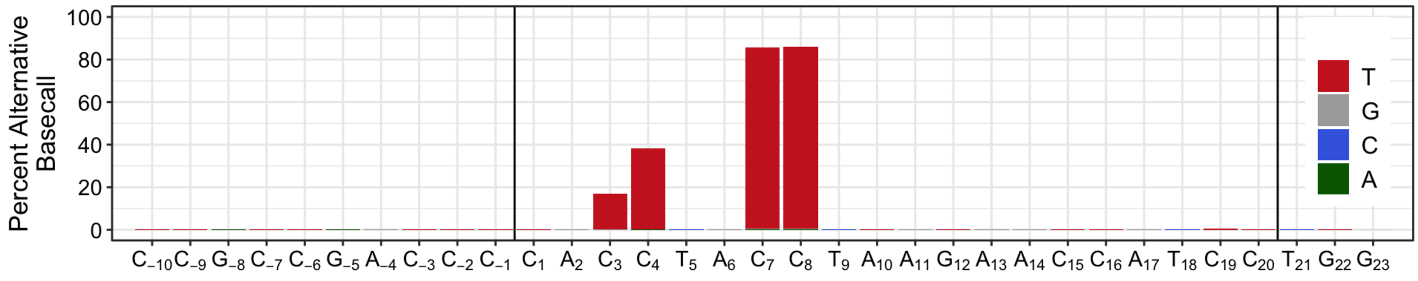

178. B 3G PDCD1 Ex.1 SD + coBE4 mRNA 4 μg

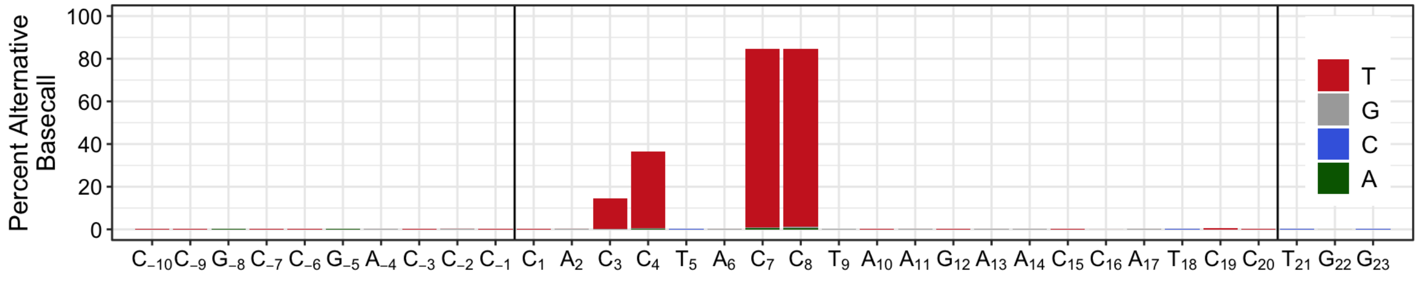

179. B 3G PDCD1 Ex.1 SD + coBE4 mRNA 4 μg

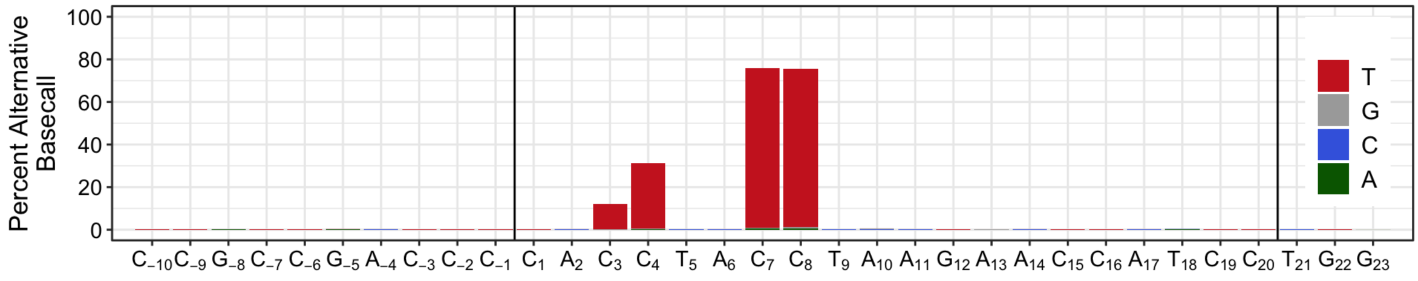

180. A 3G PDCD1 Ex.1 SD + Cas9 mRNA 1.5 μg

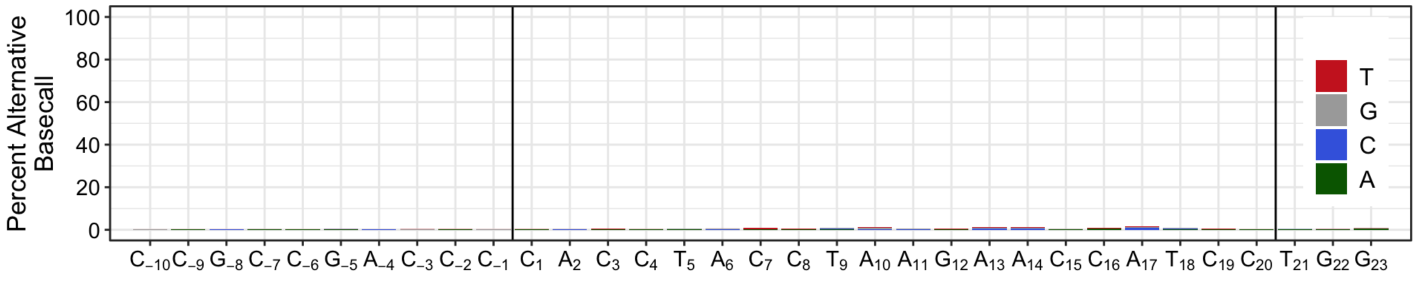

181. B 3G PDCD1 Ex.1 SD + Cas9 mRNA 1.5 μg

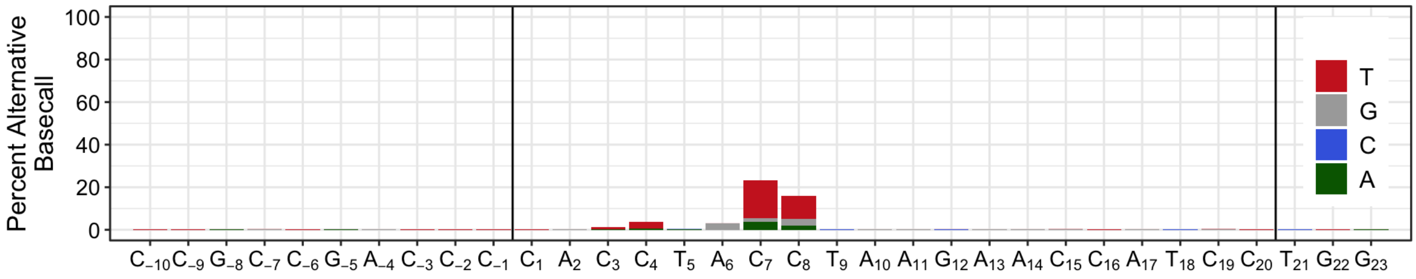

182. A 3G PDCD1 Ex.1 SD + Cas9 mRNA 1.5 μg

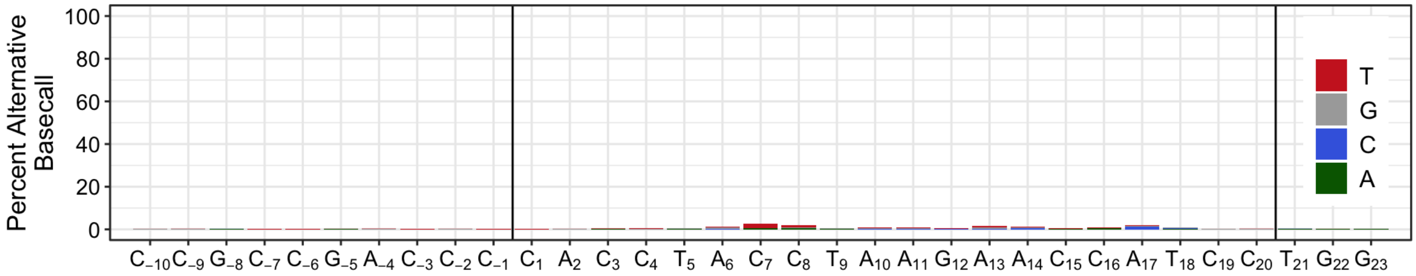

183. B 3G PDCD1 Ex.1 SD + Cas9 mRNA 1.5 μg

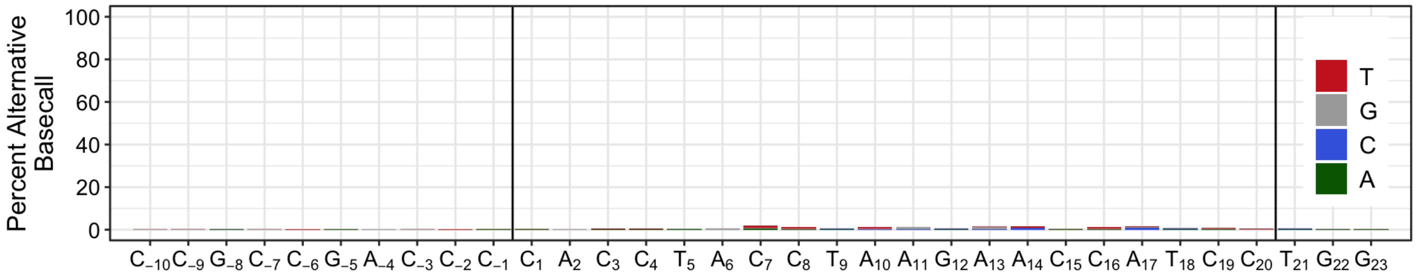

184. A 3G PDCD1 Ex.1 SD + Cas9 mRNA 4 µg

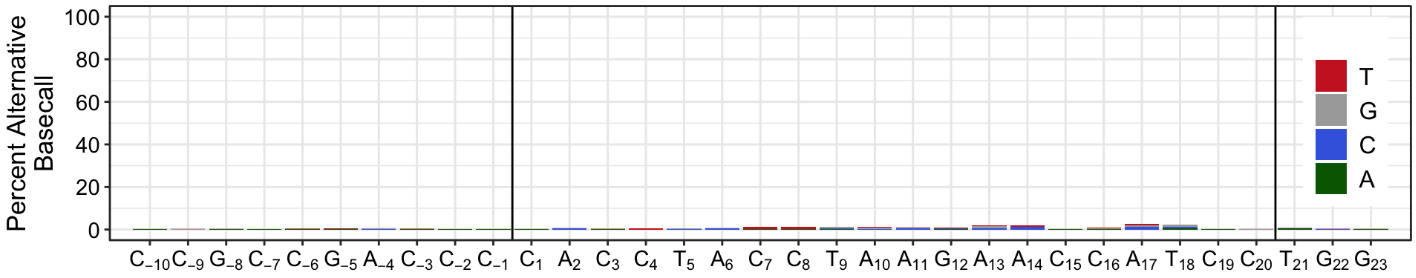

185. B 3G PDCD1 Ex.1 SD + Cas9 mRNA 4 µg

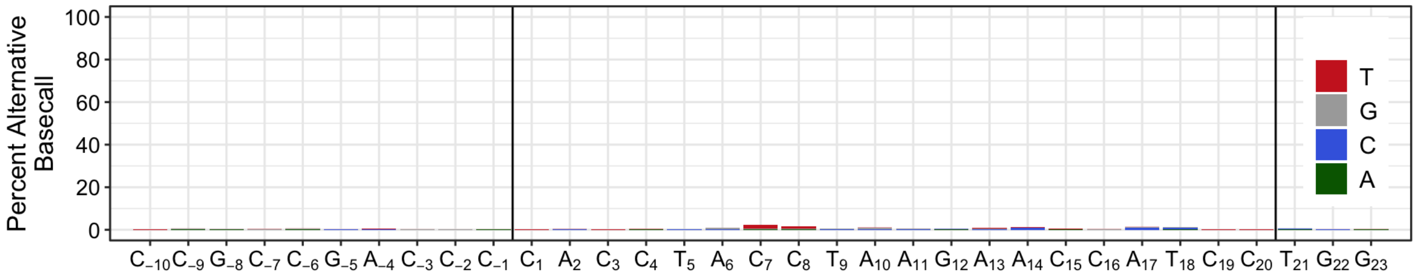

186. A 3G PDCD1 Ex.1 SD + Cas9 mRNA 4 µg

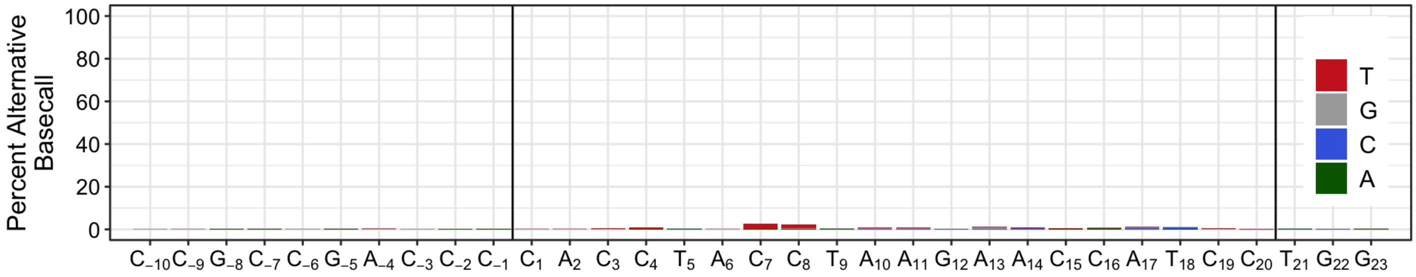

187. B 3G PDCD1 Ex.1 SD + Cas9 mRNA 4 µg

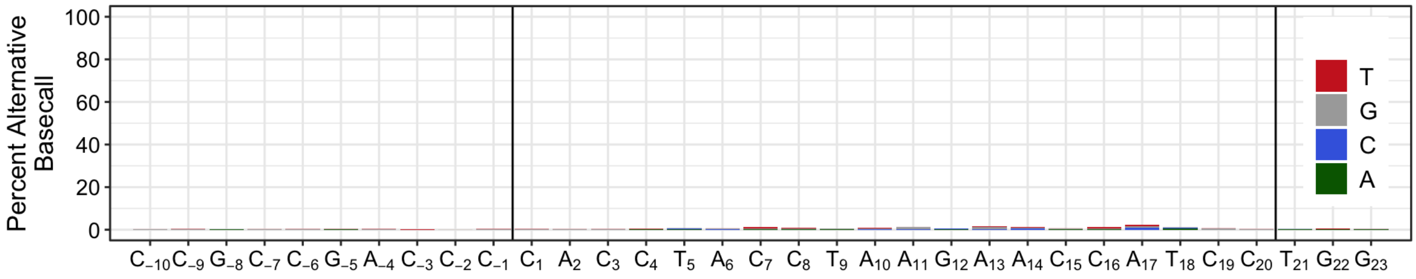

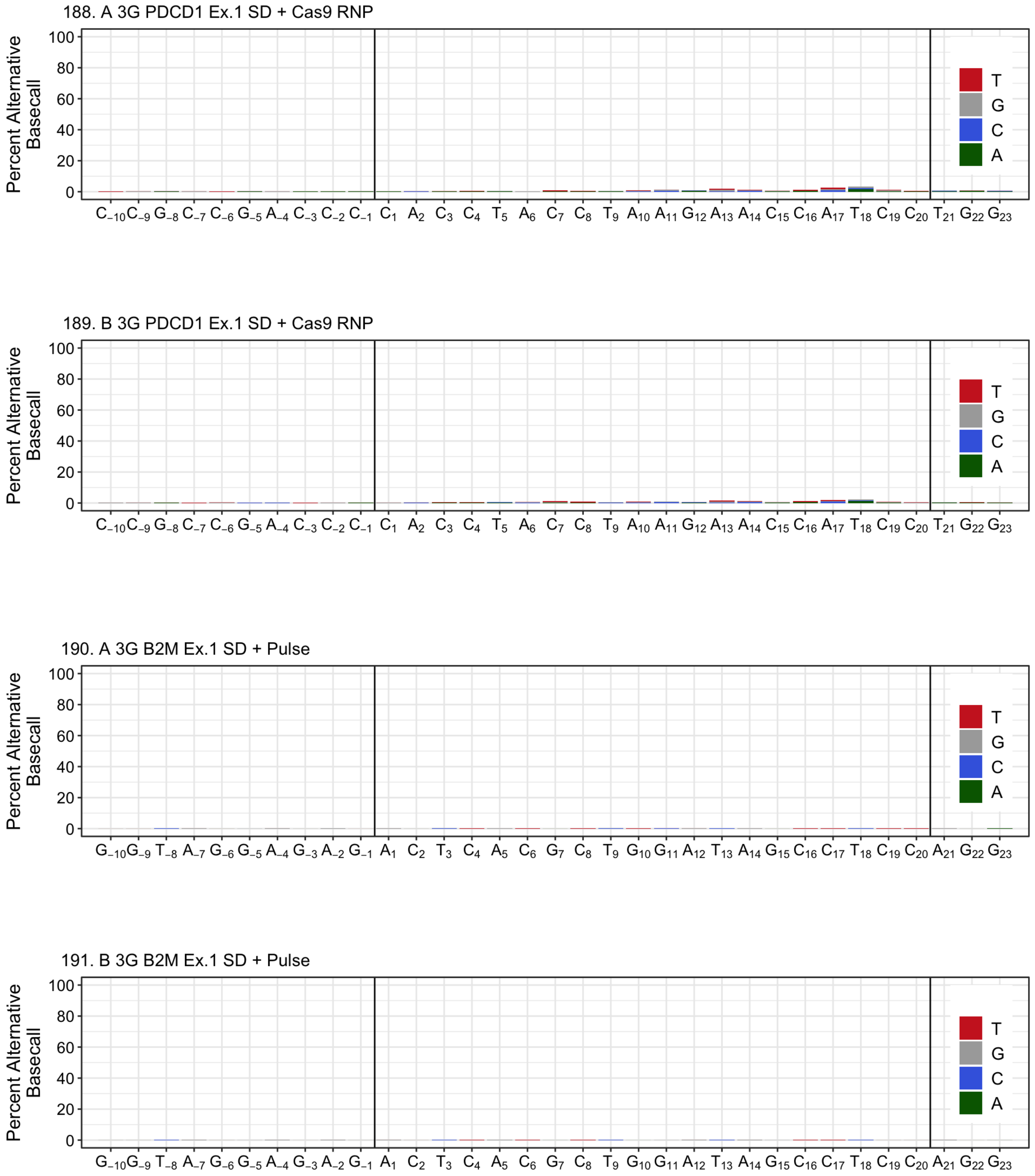
188. A 3G PDCD1 Ex.1 SD + Cas9 RNP
Percent Alternative Basecall
189. B 3G PDCD1 Ex.1 SD + Cas9 RNP
190. A 3G B2M Ex.1 SD + Pulse
191. B 3G B2M Ex.1 SD + Pulse
T
G
C
A

192. A 3G B2M Ex.1 SD + Pulse

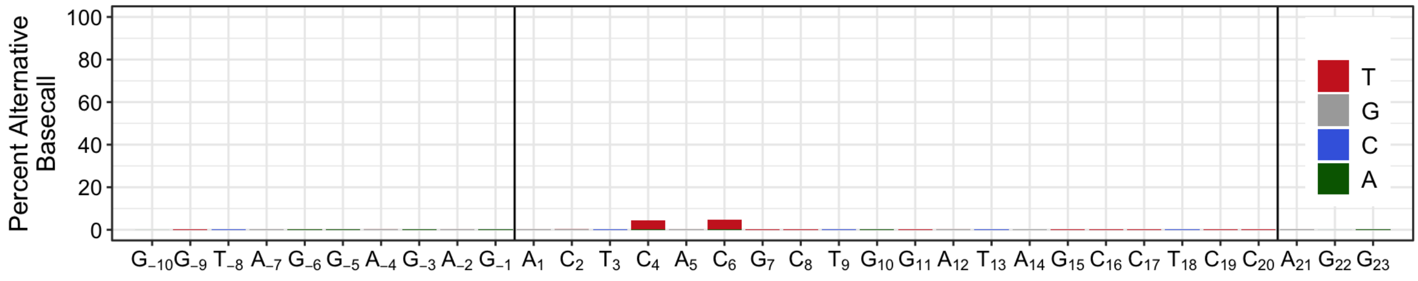

193. B 3G B2M Ex.1 SD + Pulse

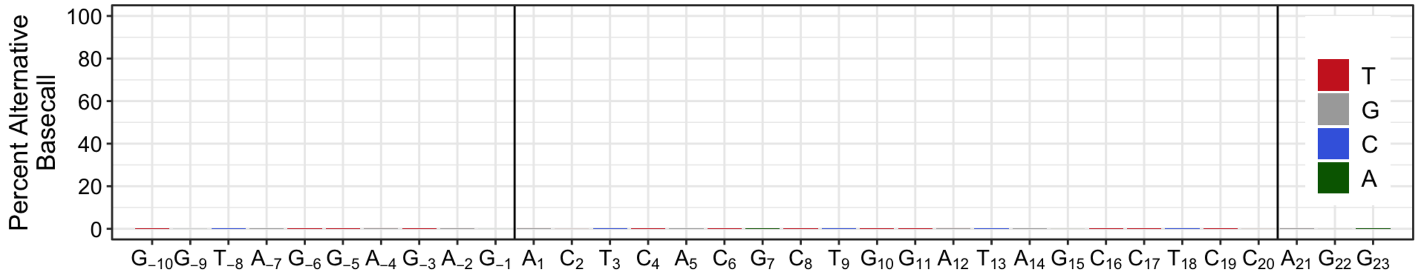

194. A 3G B2M Ex.1 SD + Pulse

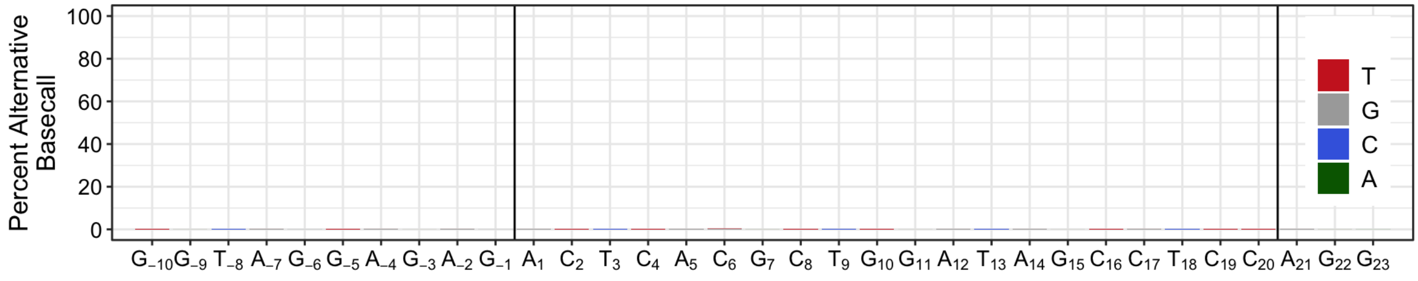

195. B 3G B2M Ex.1 SD + Pulse

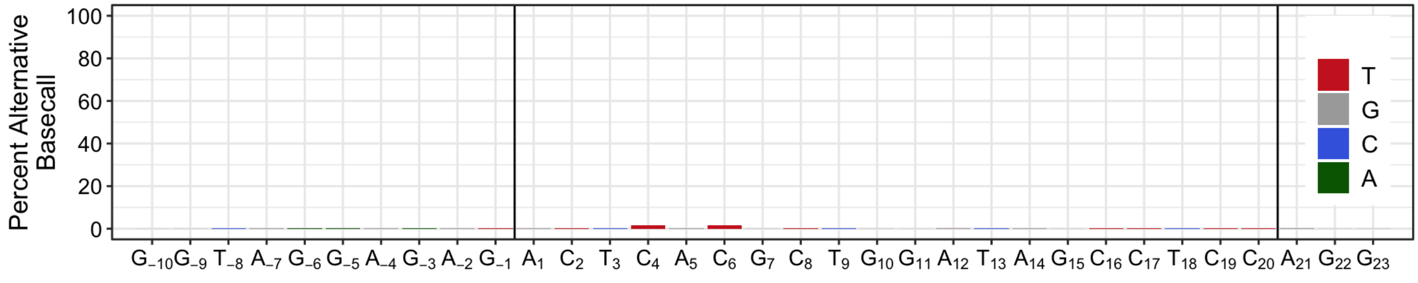

196. A 3G B2M Ex.1 SD + Pulse

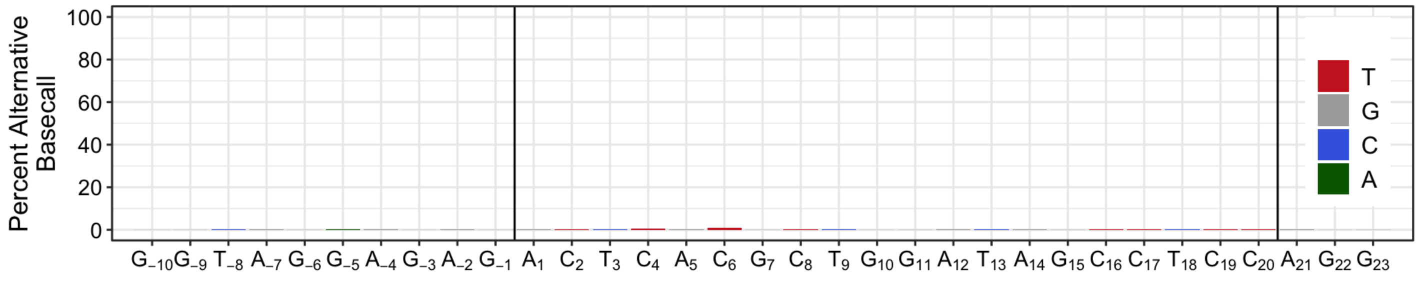

197. B 3G B2M Ex.1 SD + Pulse

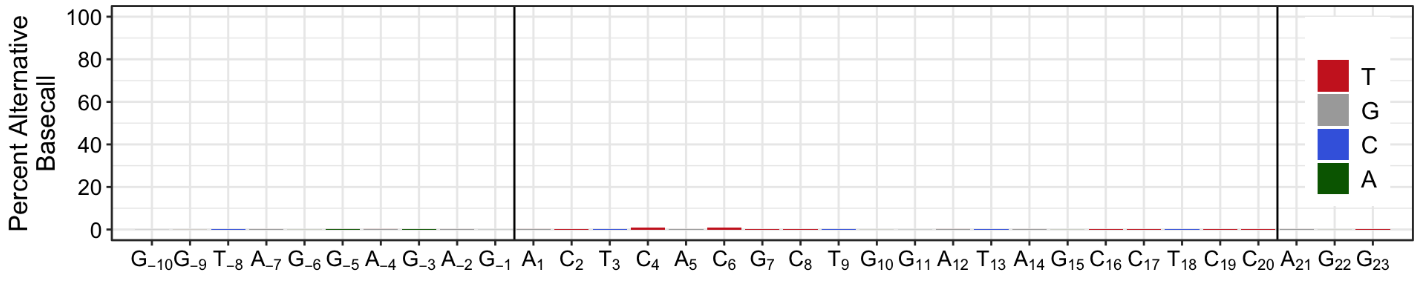

198. A 3G B2M Ex.1 SD + BE3 conc. mRNA 3 µg

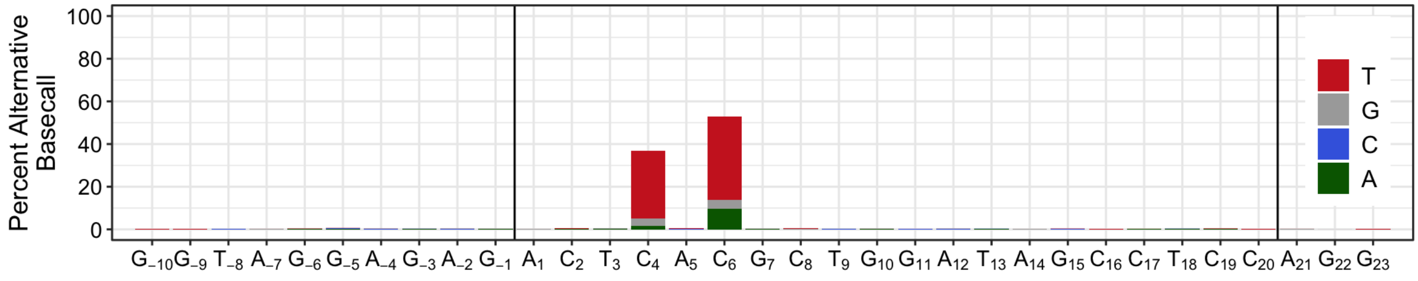

199. B 3G B2M Ex.1 SD + BE3 conc. mRNA 3 µg

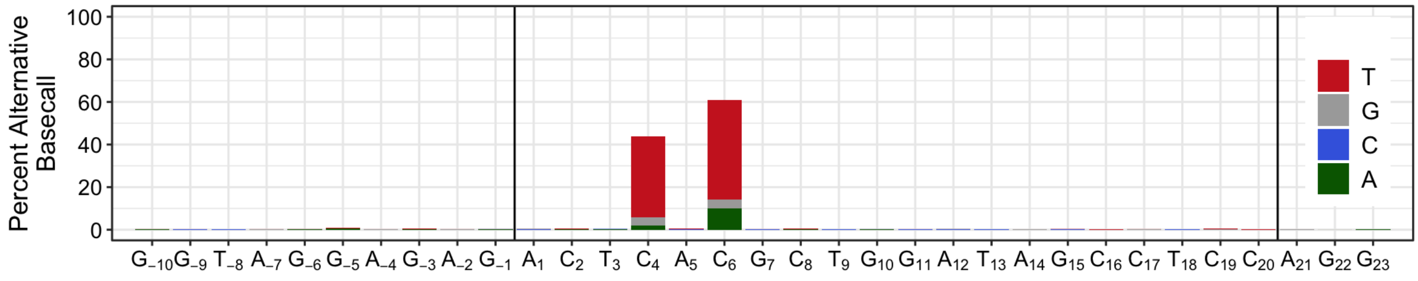

200. A 3G B2M Ex.1 SD + BE4 conc. mRNA 3 µg

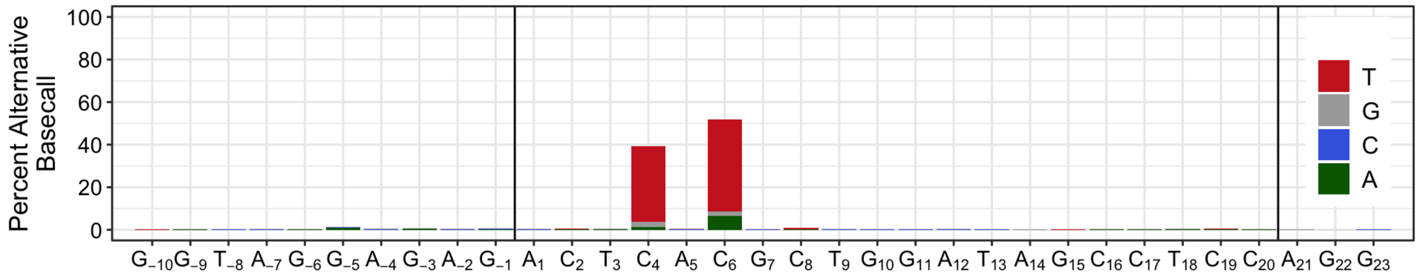

201. B 3G B2M Ex.1 SD + BE4 conc. mRNA 3 µg

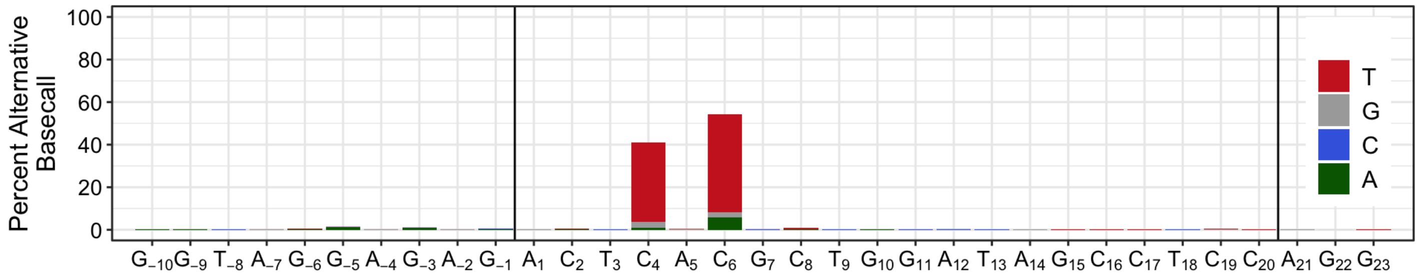

202. A 3G B2M Ex.1 SD + BE4 RNP

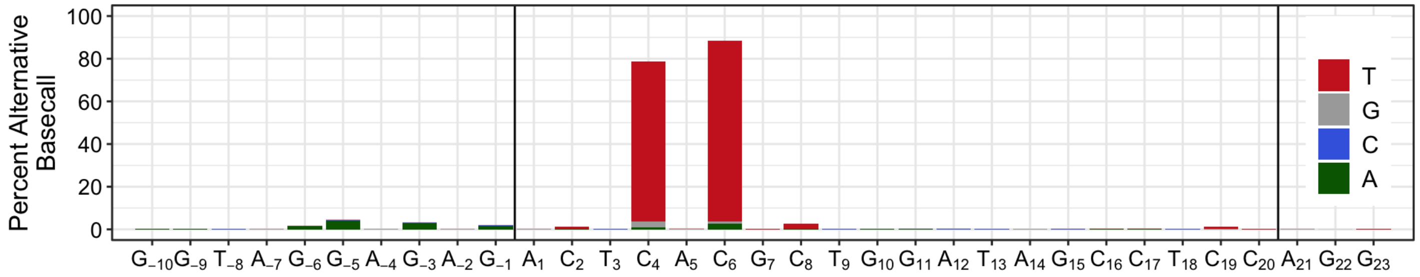

203. B 3G B2M Ex.1 SD + BE4 RNP

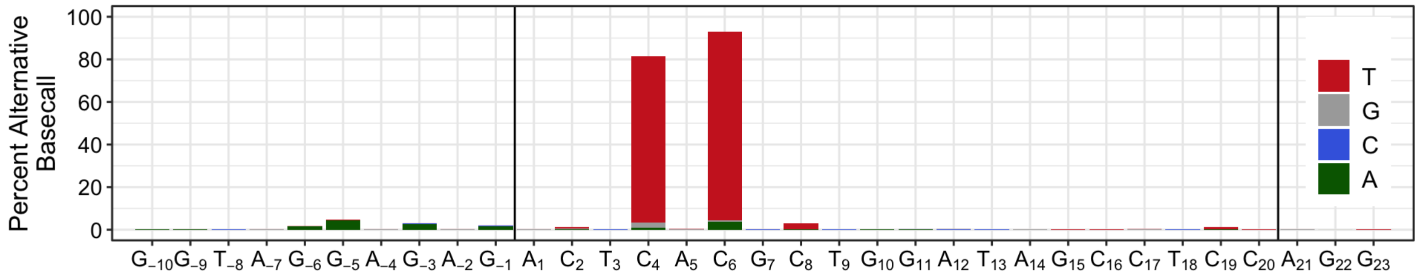

204. A 3G B2M Ex.1 SD + coBE4 mRNA 1.5 µg

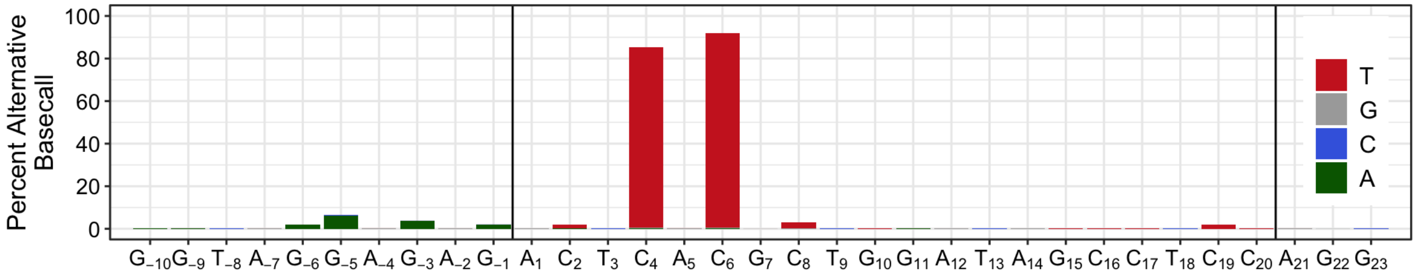

205. B 3G B2M Ex.1 SD + coBE4 mRNA 1.5 µg

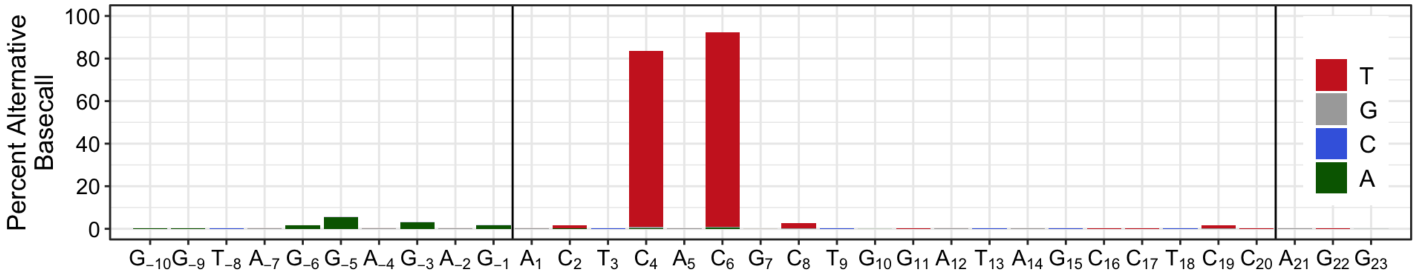

206. A 3G B2M Ex.1 SD + coBE4 mRNA 4 µg

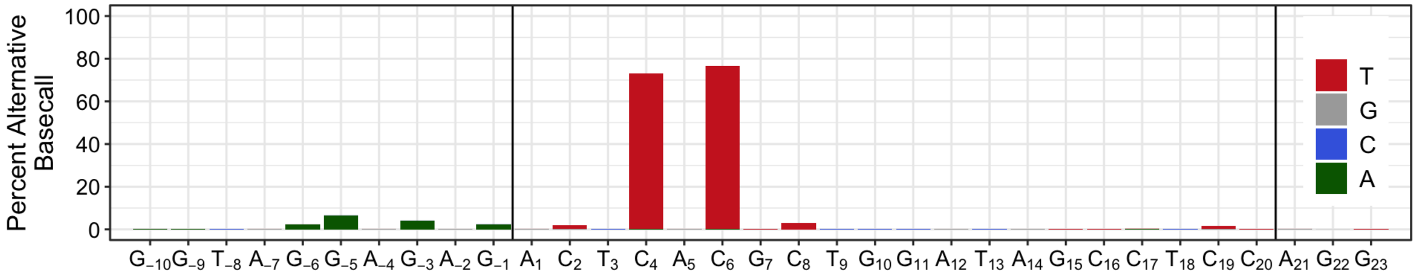

207. B 3G B2M Ex.1 SD + coBE4 mRNA 4 µg

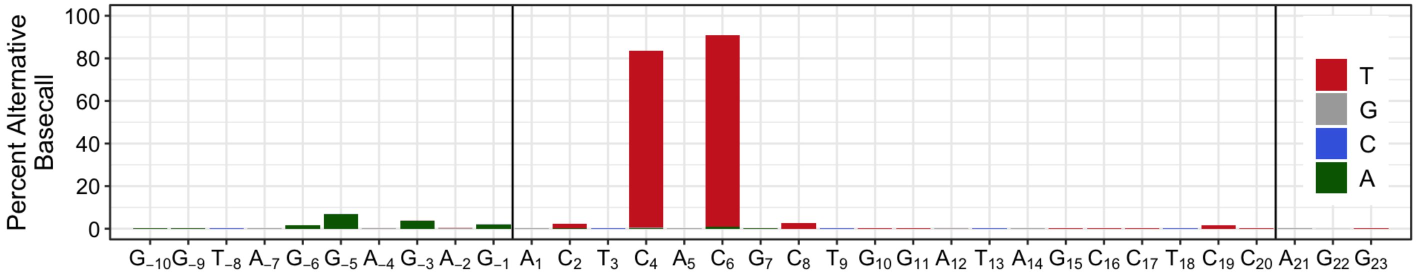

208. A 3G B2M Ex.1 SD + coBE4 mRNA 4 µg

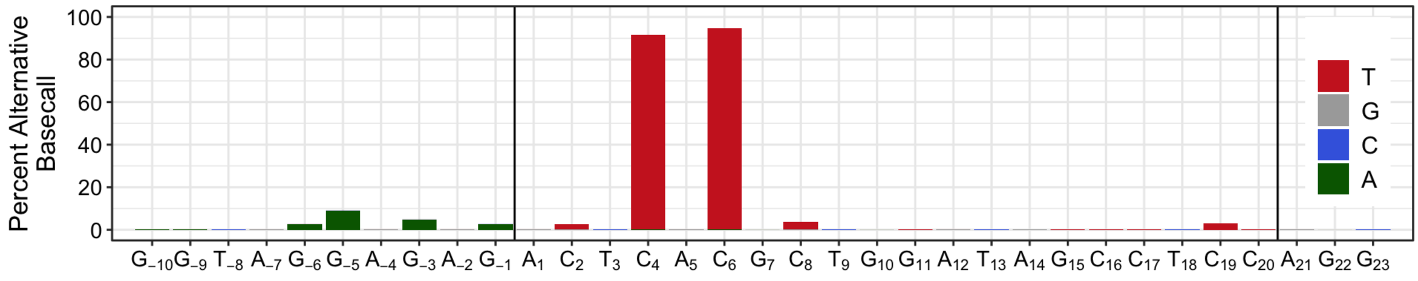

209. B 3G B2M Ex.1 SD + coBE4 mRNA 4 µg

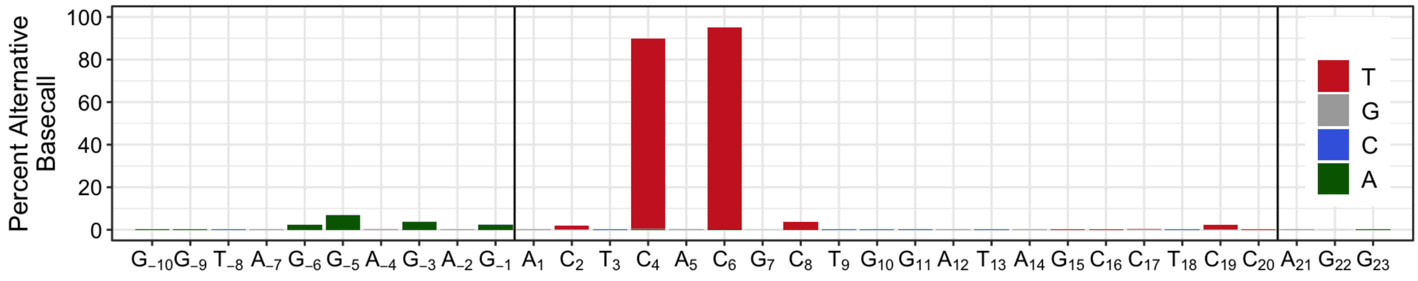

210. B 3G B2M Ex.1 SD + coBE4 mRNA 4 µg

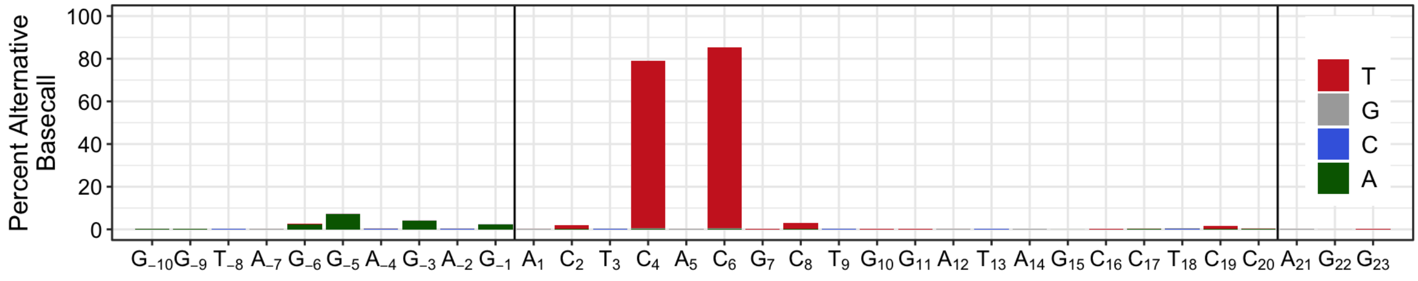

211. A 3G B2M Ex.1 SD + Cas9 mRNA 1.5 µg

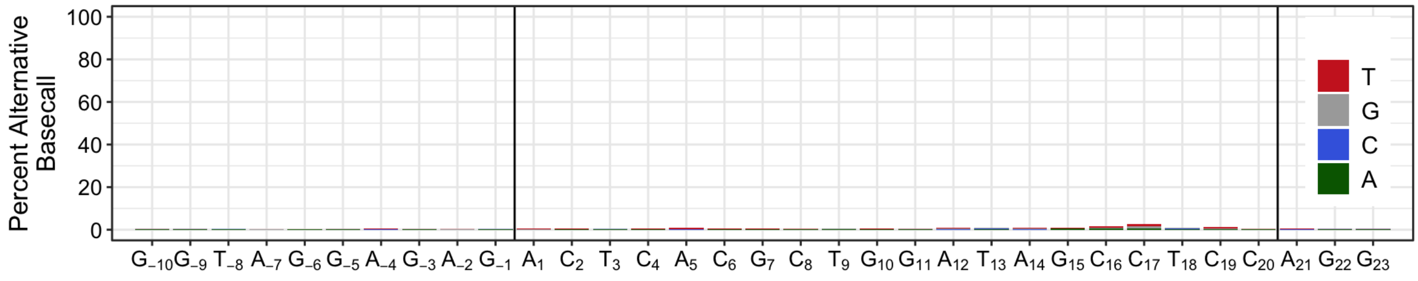

212. B 3G B2M Ex.1 SD + Cas9 mRNA 1.5 µg

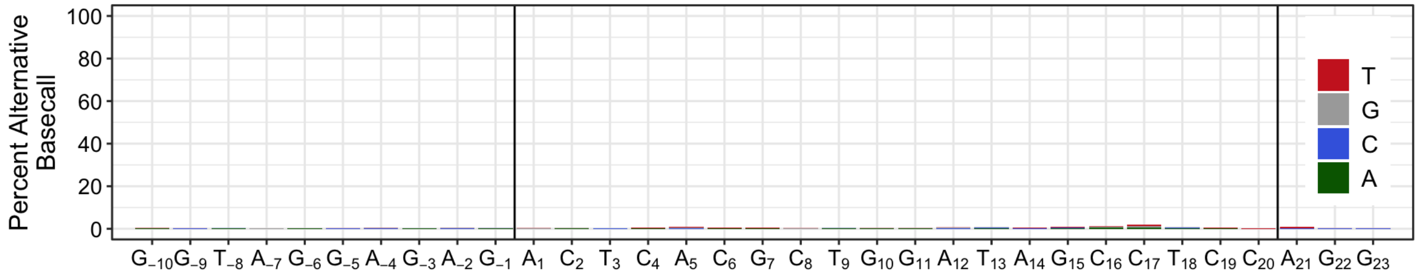

213. A 3G B2M Ex.1 SD + Cas9 mRNA 1.5 µg

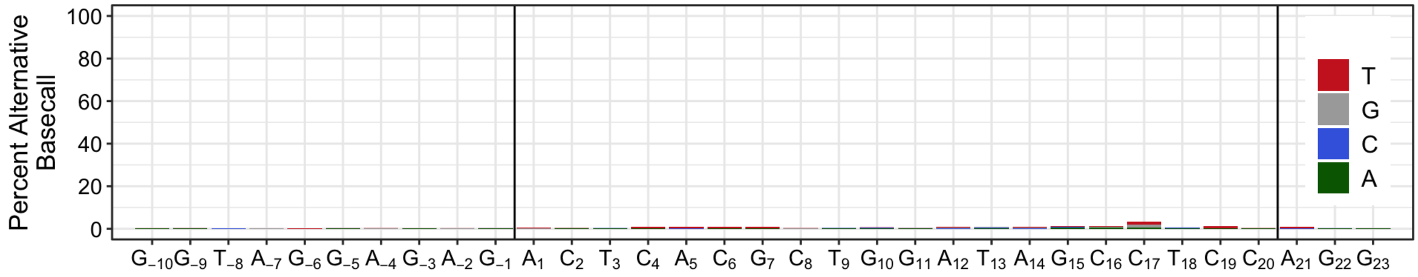

214. B 3G B2M Ex.1 SD + Cas9 mRNA 1.5 µg

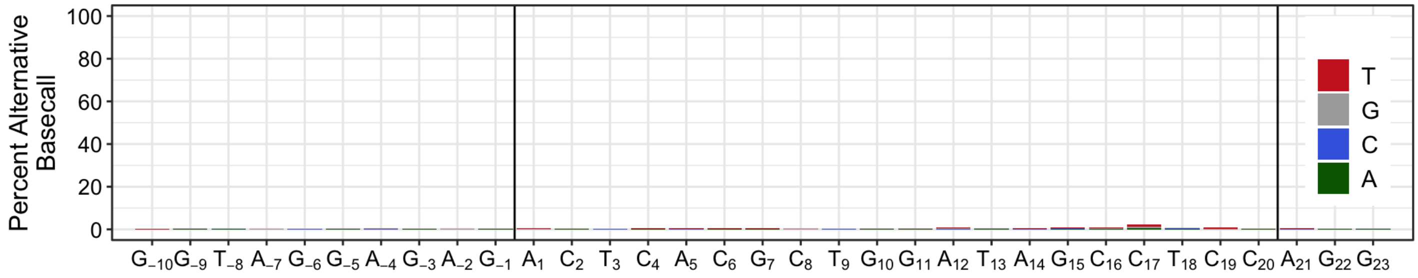

215. A 3G B2M Ex.1 SD + Cas9 mRNA 4 µg

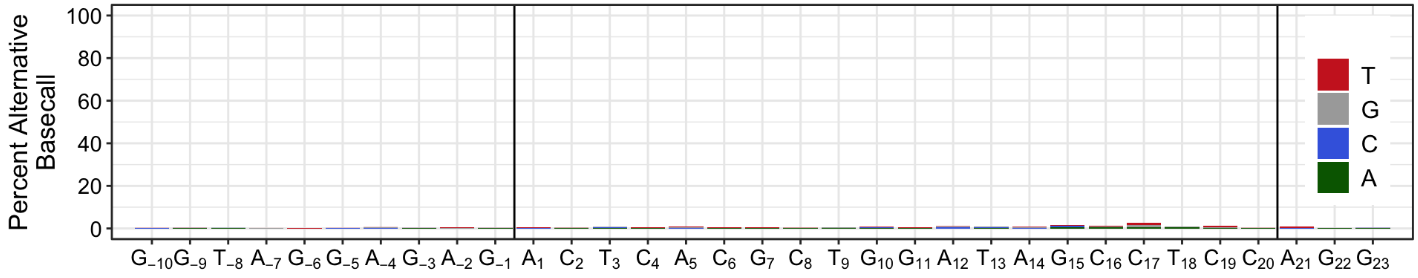

216. B 3G B2M Ex.1 SD + Cas9 mRNA 4 µg

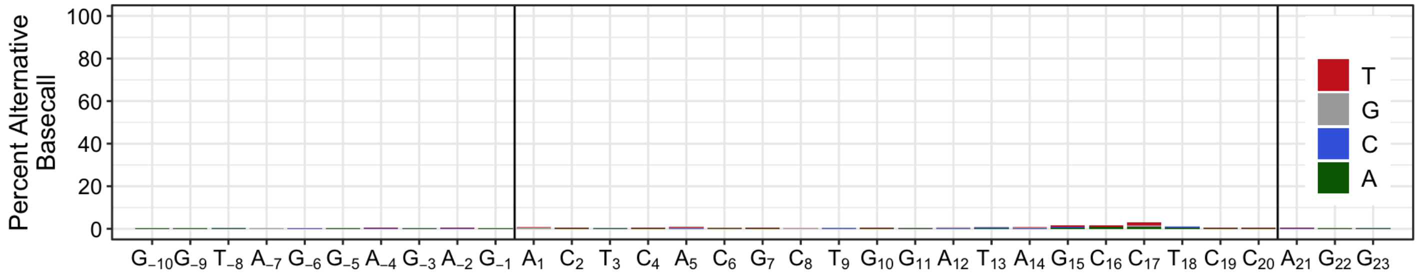

217. A 3G B2M Ex.1 SD + Cas9 mRNA 4 µg

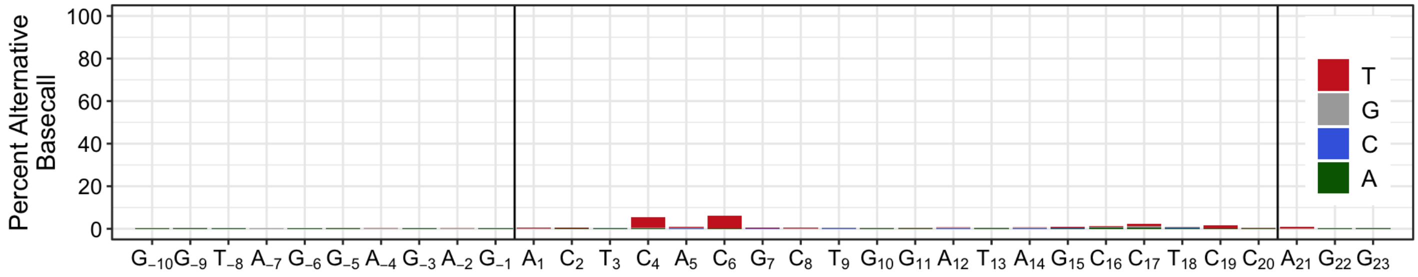

218. B 3G B2M Ex.1 SD + Cas9 mRNA 4 µg

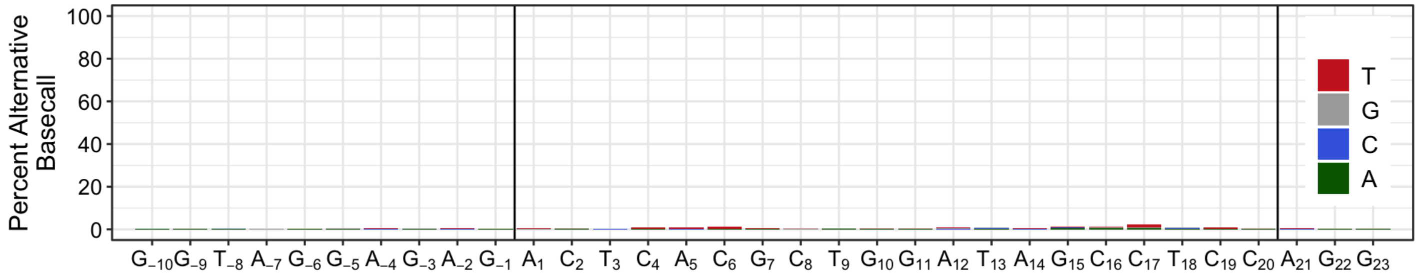

219. A 3G B2M Ex.1 SD + Cas9 RNP

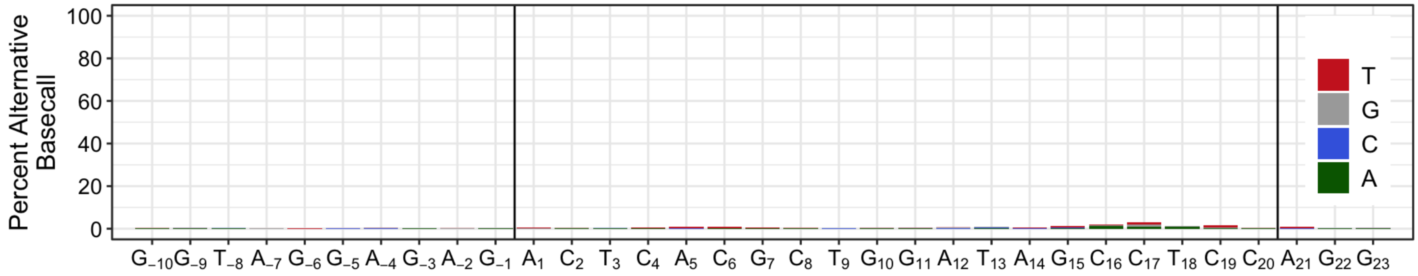

220. B 3G B2M Ex.1 SD + Cas9 RNP

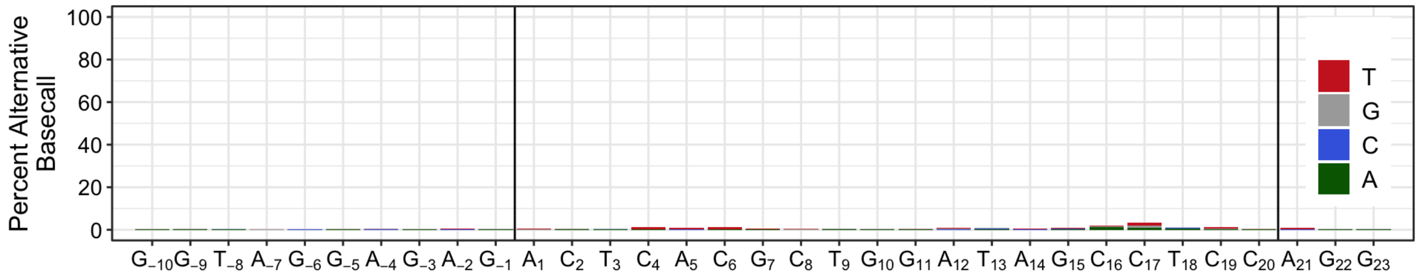

221. A 3G B2M Ex.1 SD + Pulse

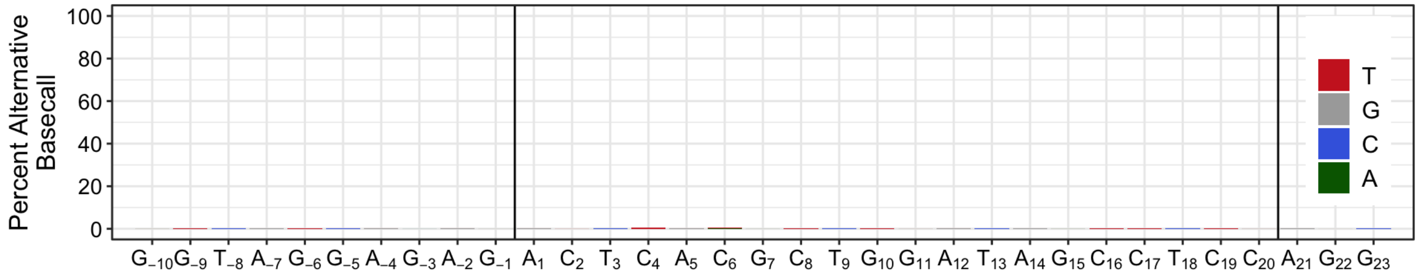

222. B 3G B2M Ex.1 SD + Pulse

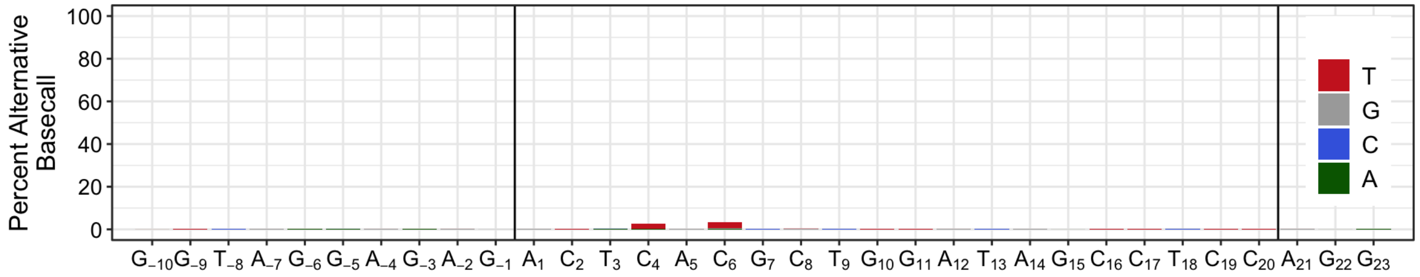

223. A 3G B2M Ex.1 SD + BE4 RNP

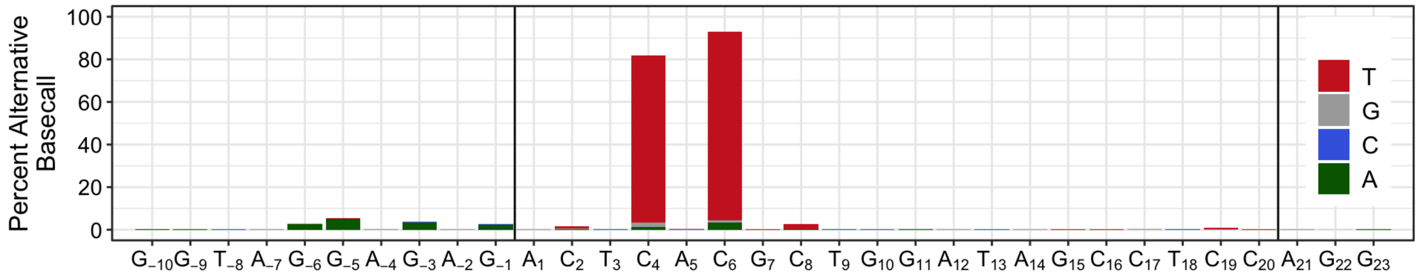

224. B 3G B2M Ex.1 SD + BE4 RNP

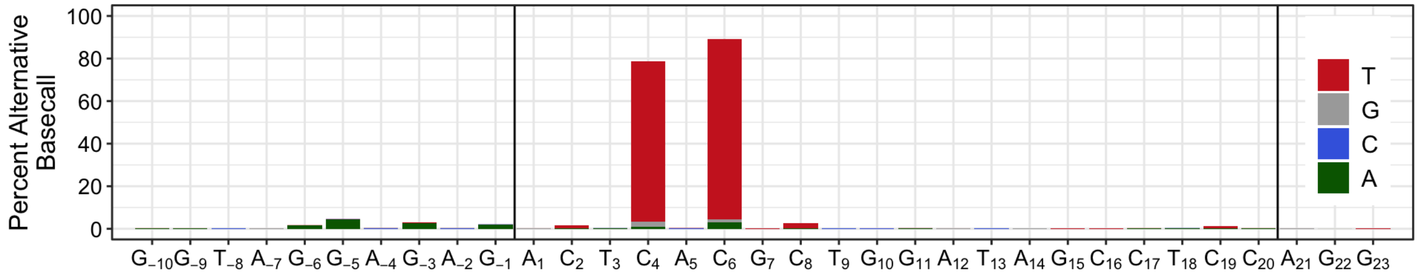

225. A 3G TRAC Ex.3 SA + Pulse

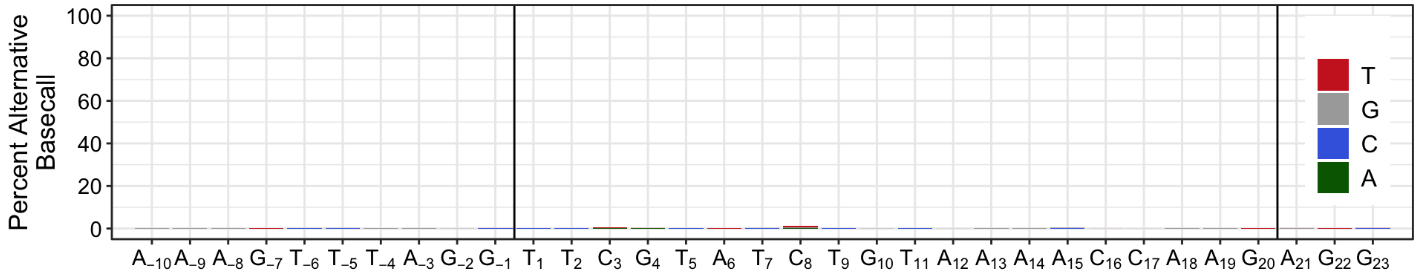

226. B 3G TRAC Ex.3 SA + Pulse

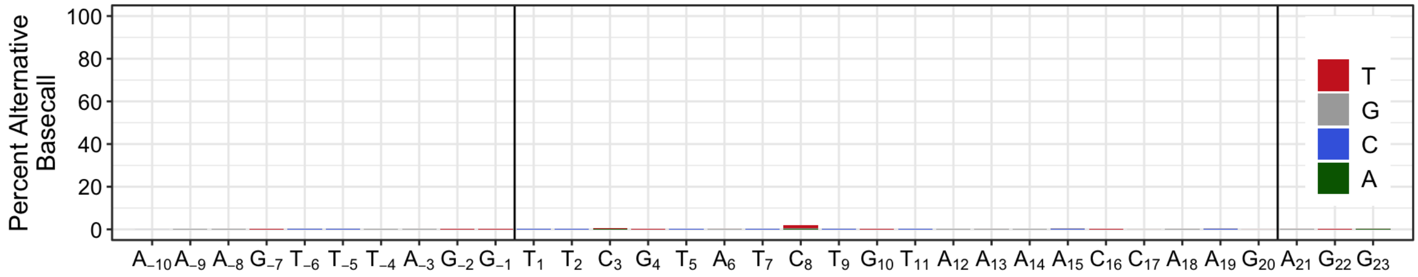

227. A 3G TRAC Ex.3 SA + BE4 RNP

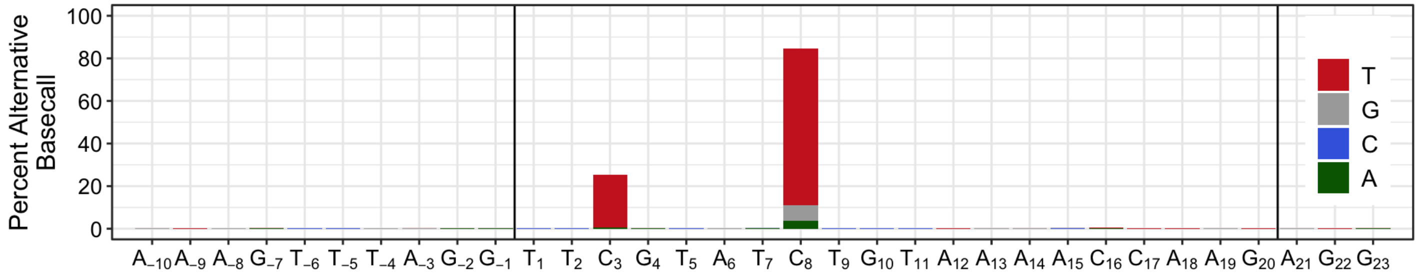

228. B 3G TRAC Ex.3 SA + BE4 RNP

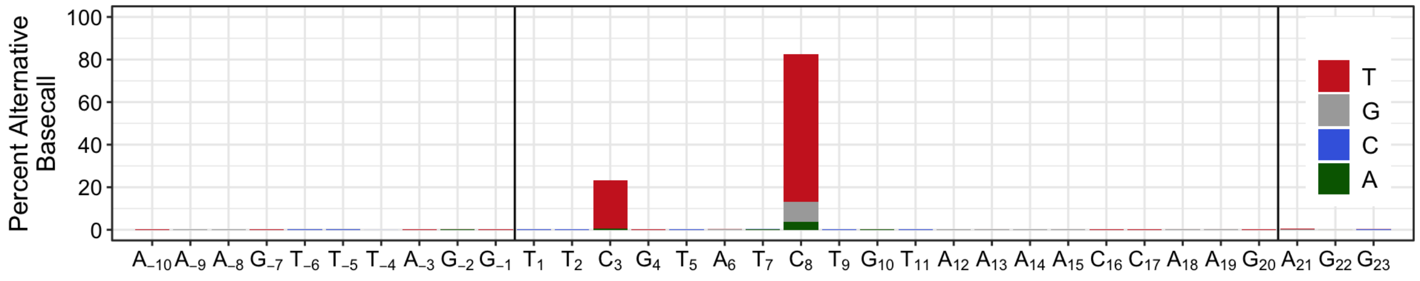

229. A 3G TRAC Ex.3 SA + Pulse

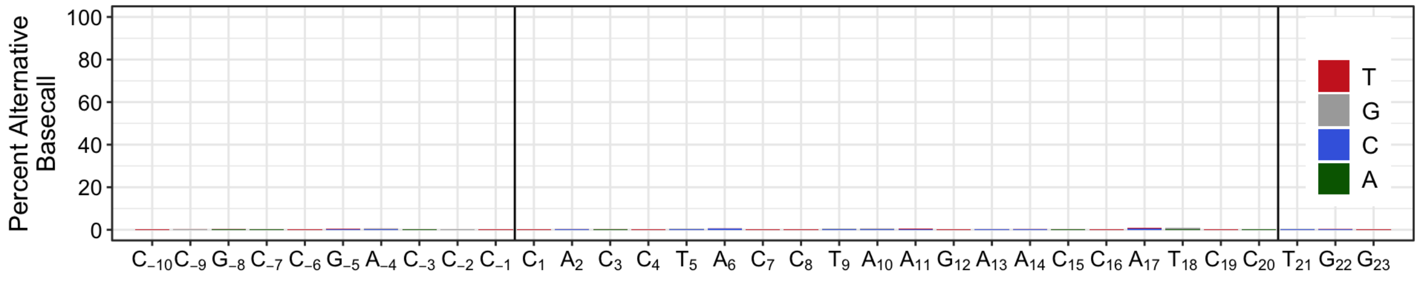

230. B 3G TRAC Ex.3 SA + Pulse

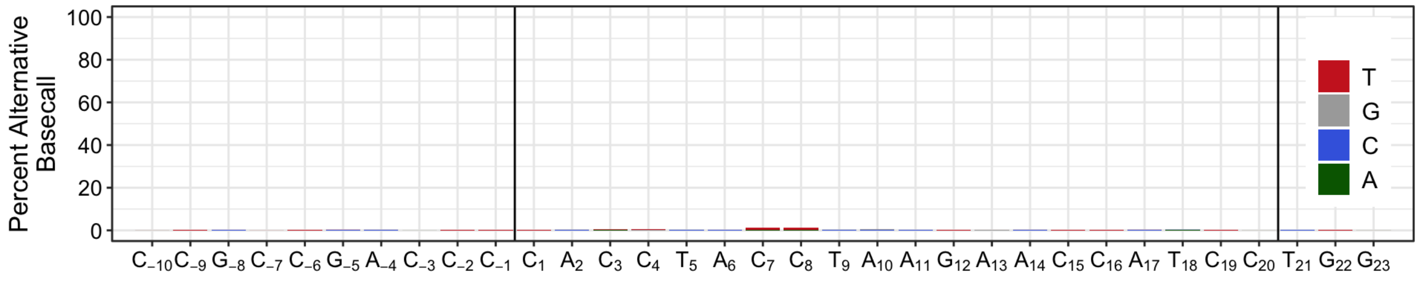

231. A 3G TRAC Ex.3 SA + BE4 RNP

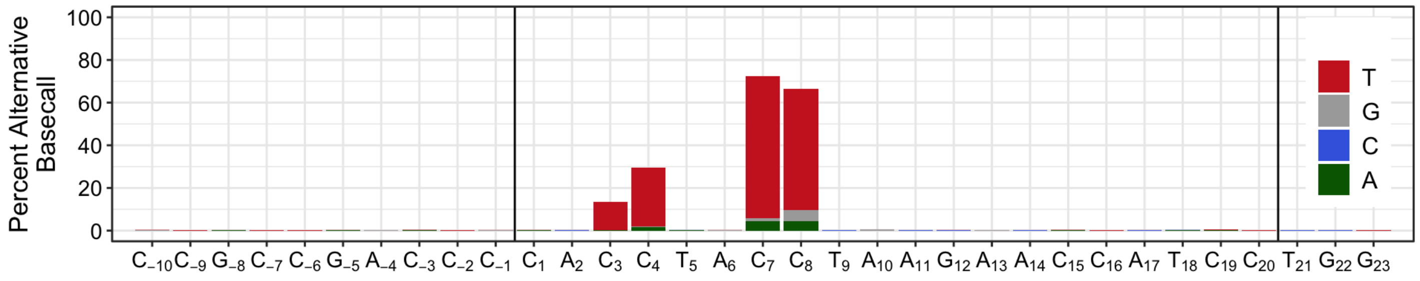

232. B 3G TRAC Ex.3 SA + BE4 RNP

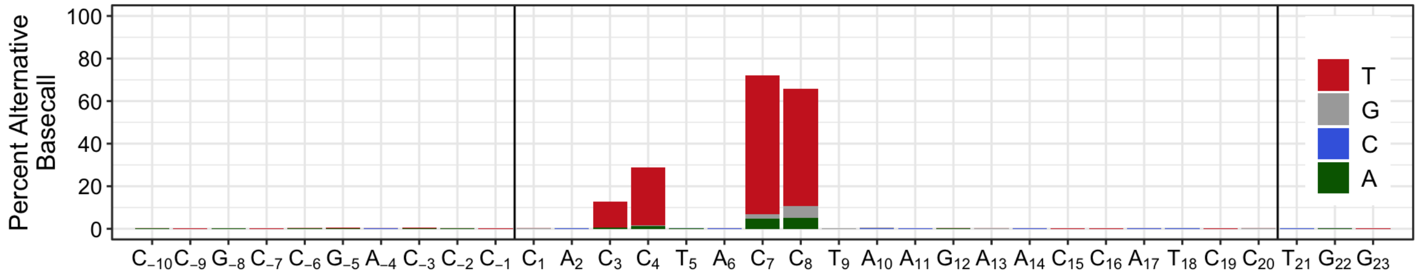

233. A B2M Ex.1 SD OnT + Pulse

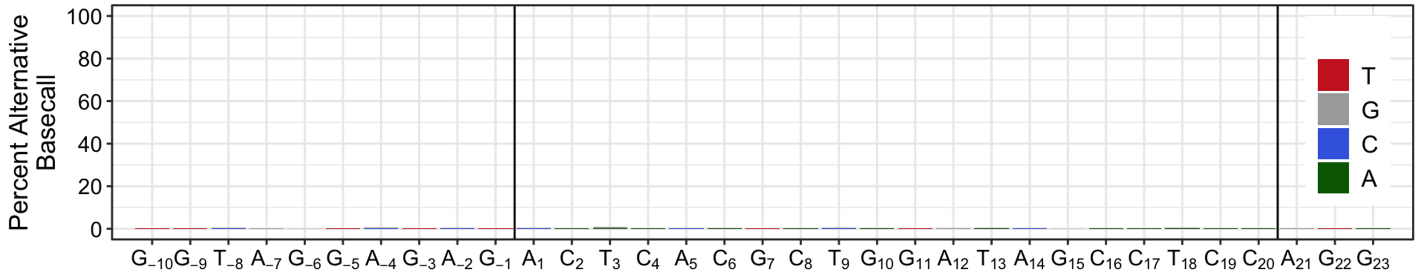

234. A B2M Ex.1 SD OT1 + Pulse

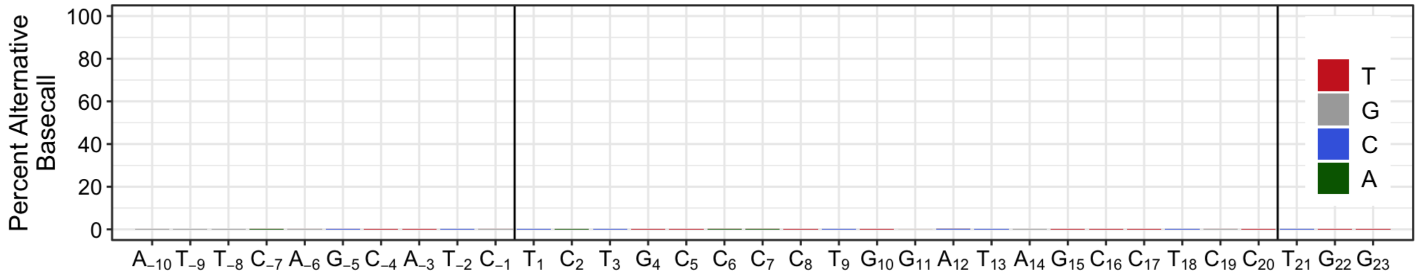

235. A B2M Ex.1 SD OT2 + Pulse

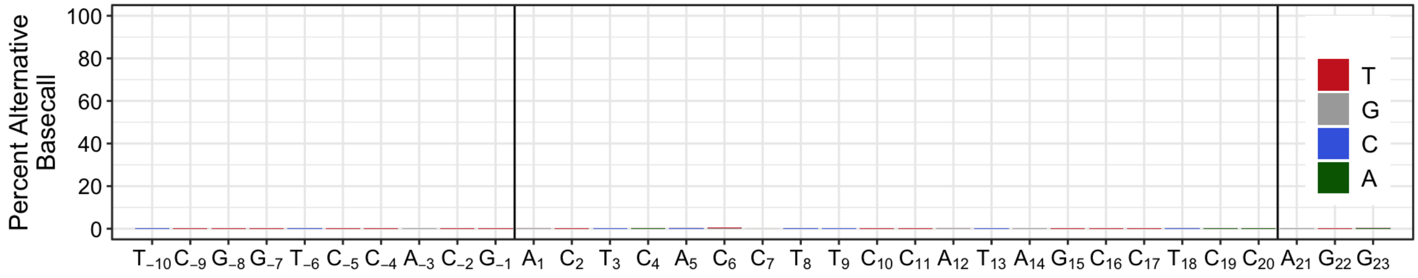

236. A B2M Ex.1 SD OT3 + Pulse

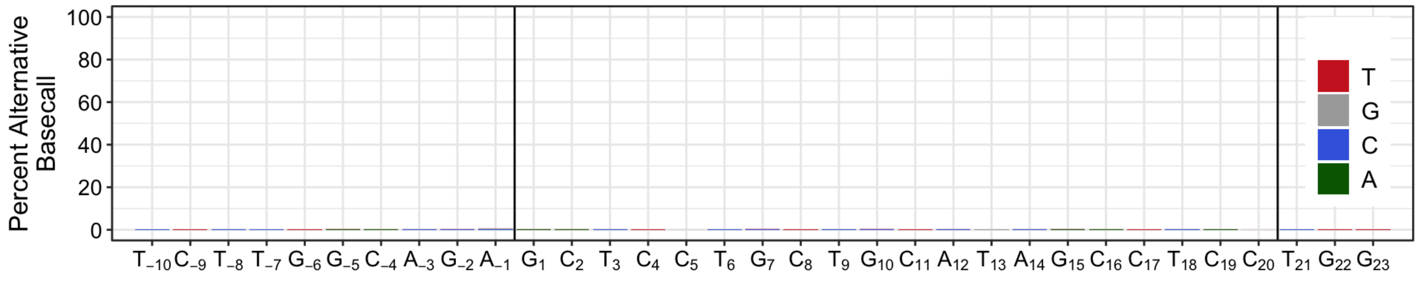

237. A B2M Ex.1 SD OT4 + Pulse

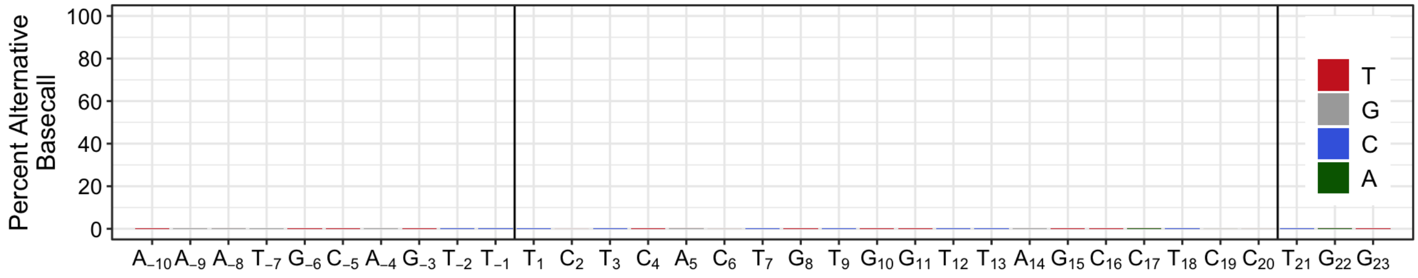

238. A B2M Ex.1 SD OT5 + Pulse

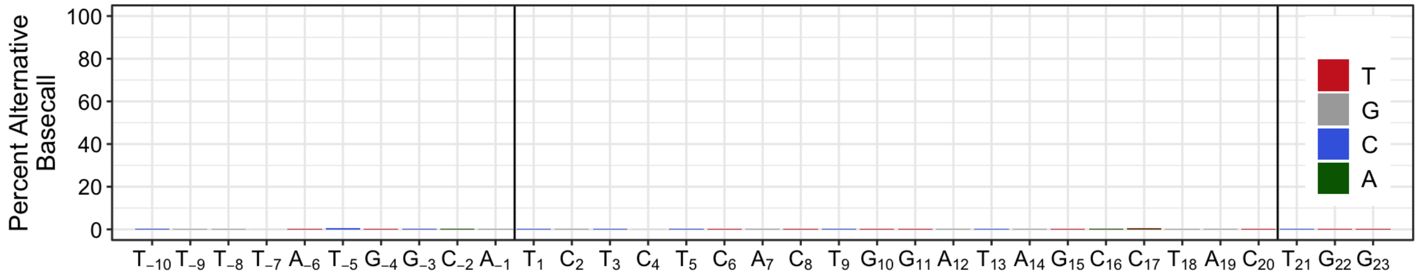

239. A B2M Ex.1 SD OT6 + Pulse

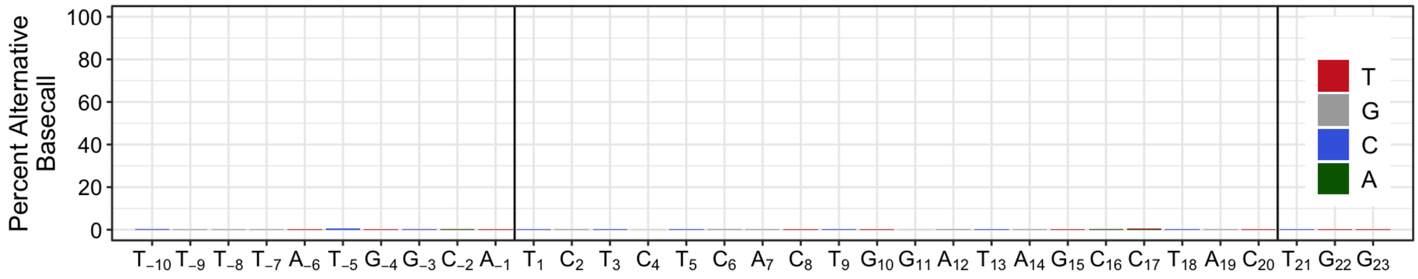

240. A B2M Ex.1 SD OT7 + Pulse

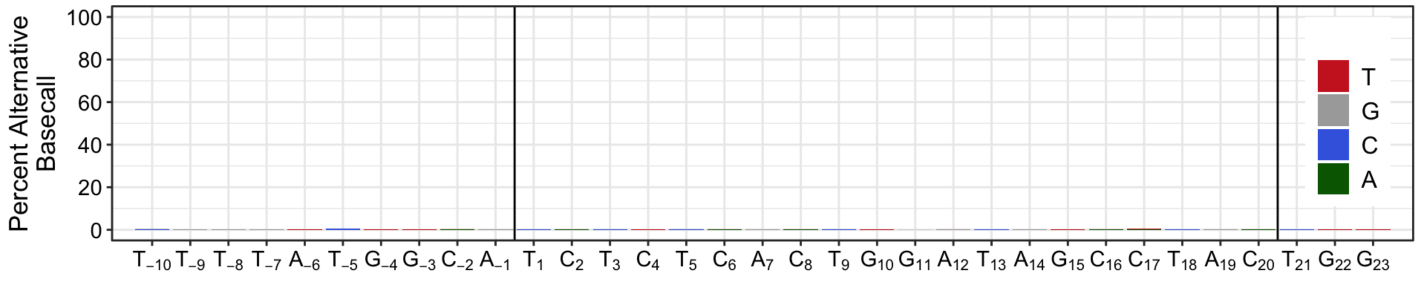

241. A B2M Ex.1 SD OT8 + Pulse

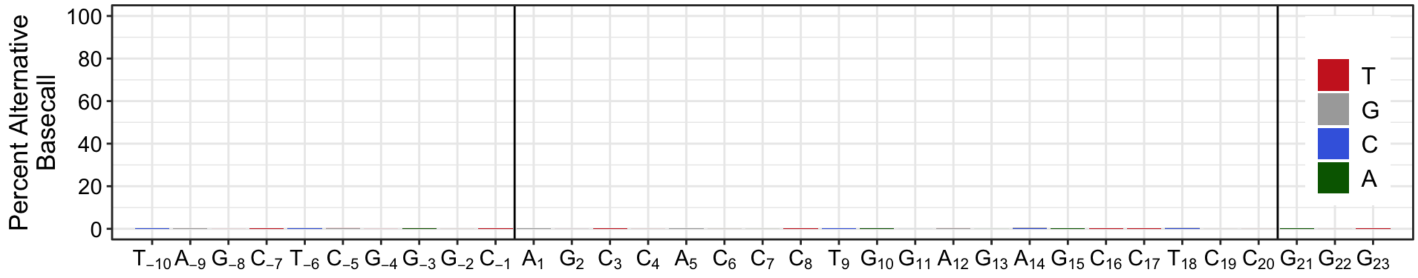

242. A B2M Ex.1 SD OT9 + Pulse

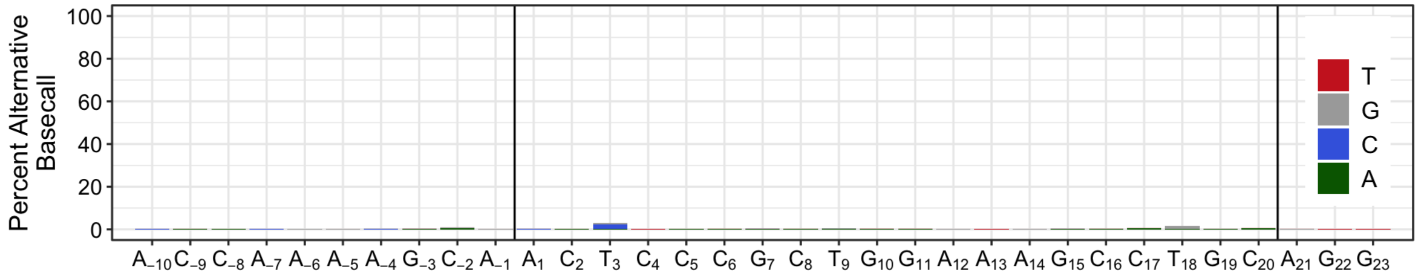

243. A B2M Ex.1 SD OT10 + Pulse

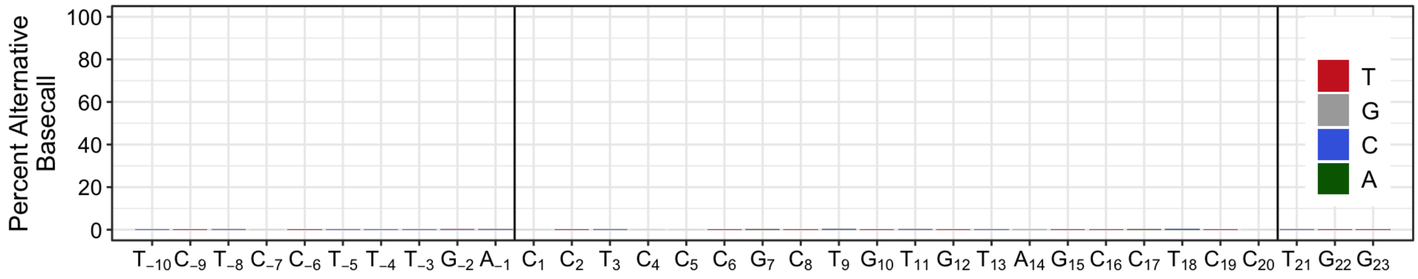

244. A B2M Ex.1 SD OnT + Cas9 mRNA

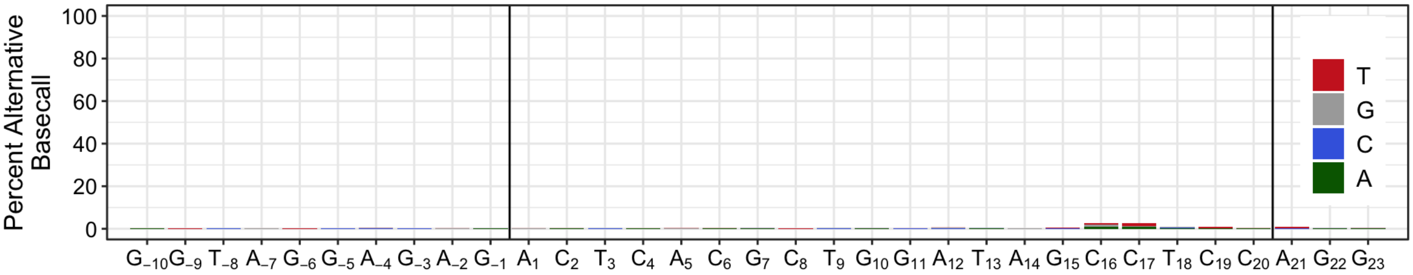

245. A B2M Ex.1 SD OT1 + Cas9 mRNA

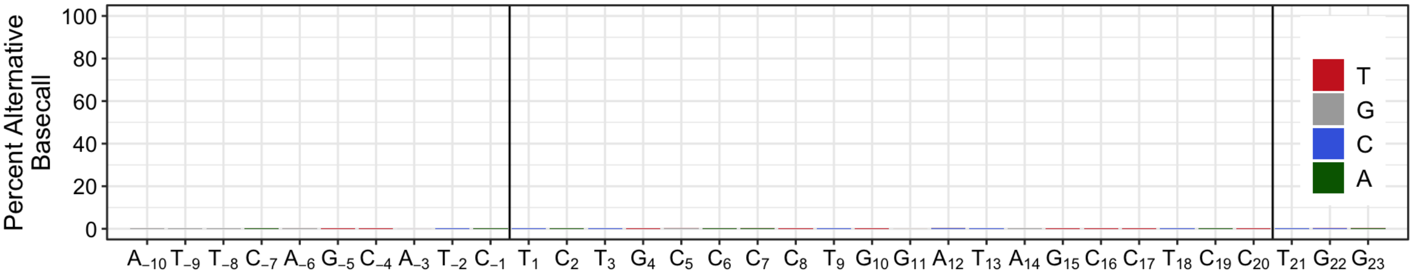

246. A B2M Ex.1 SD OT2 + Cas9 mRNA

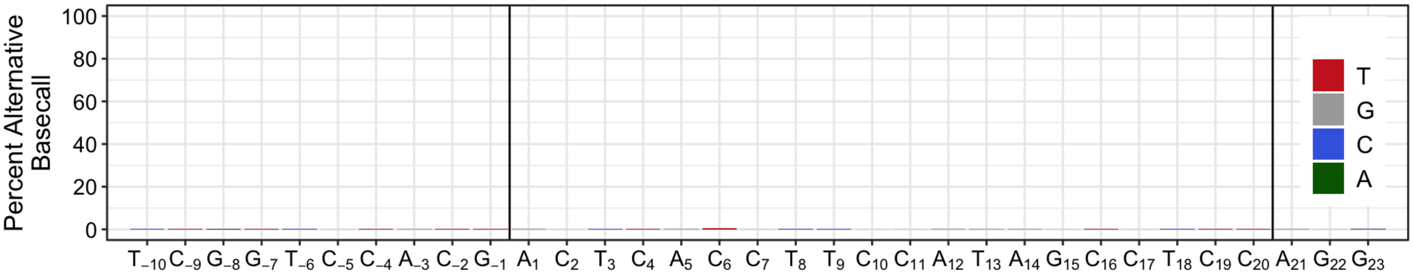

247. A B2M Ex.1 SD OT3 + Cas9 mRNA

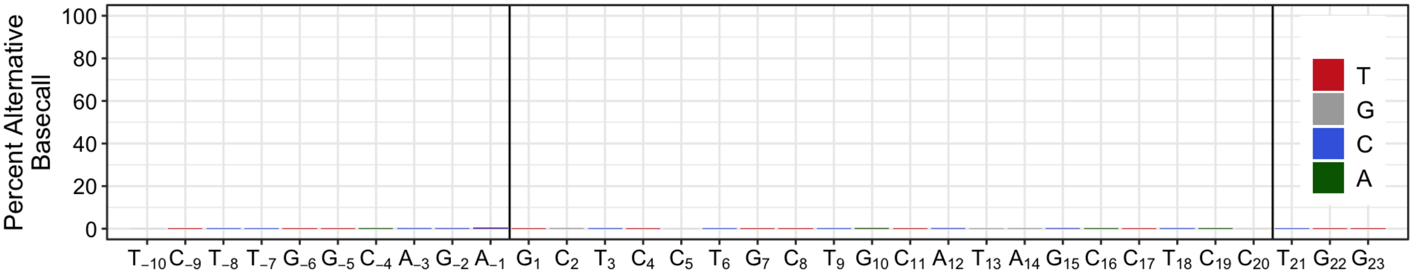

248. A B2M Ex.1 SD OT4 + Cas9 mRNA

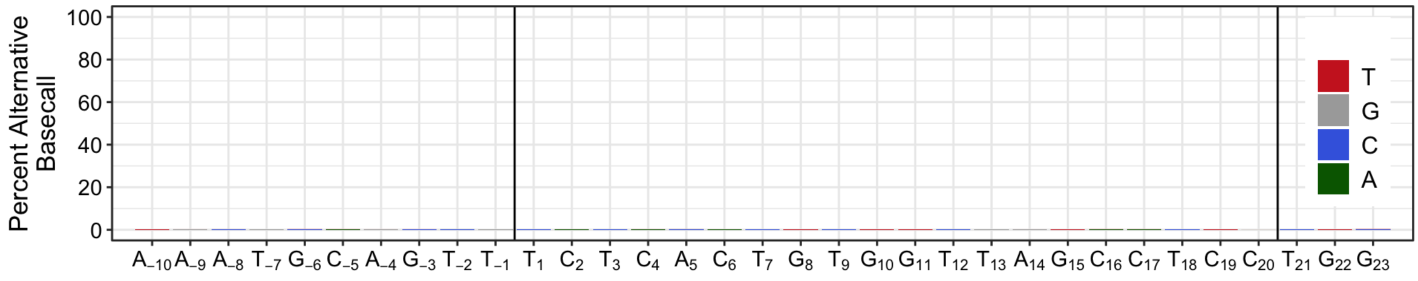

249. A B2M Ex.1 SD OT5 + Cas9 mRNA

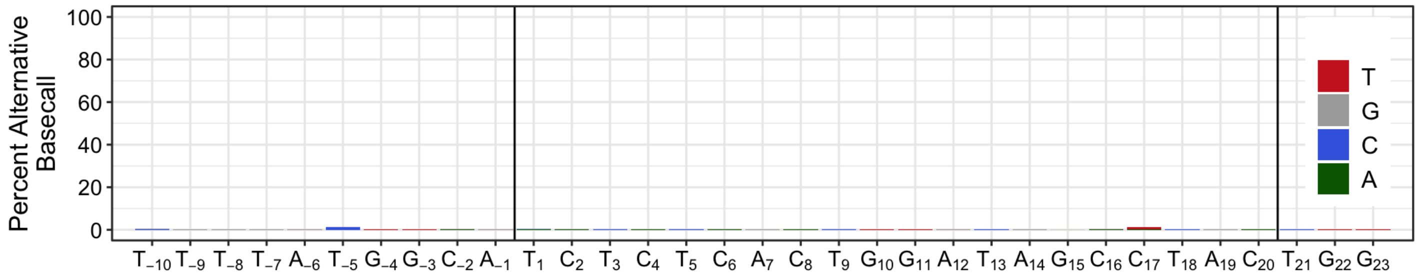

250. A B2M Ex.1 SD OT6 + Cas9 mRNA

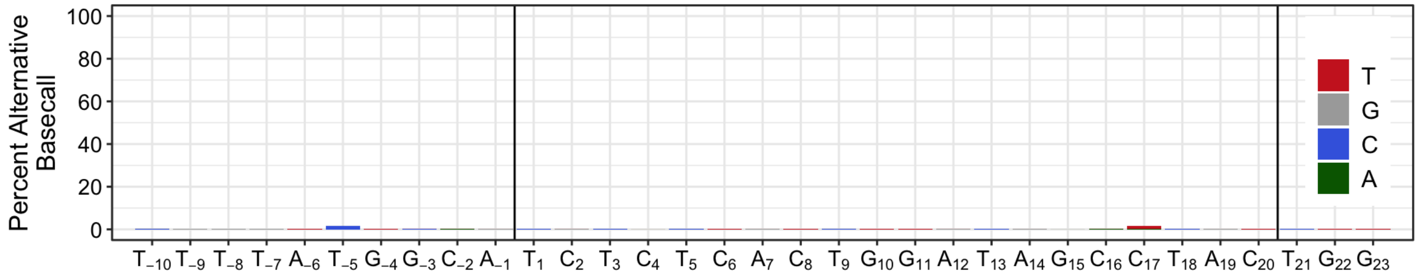

251. A B2M Ex.1 SD OT7 + Cas9 mRNA

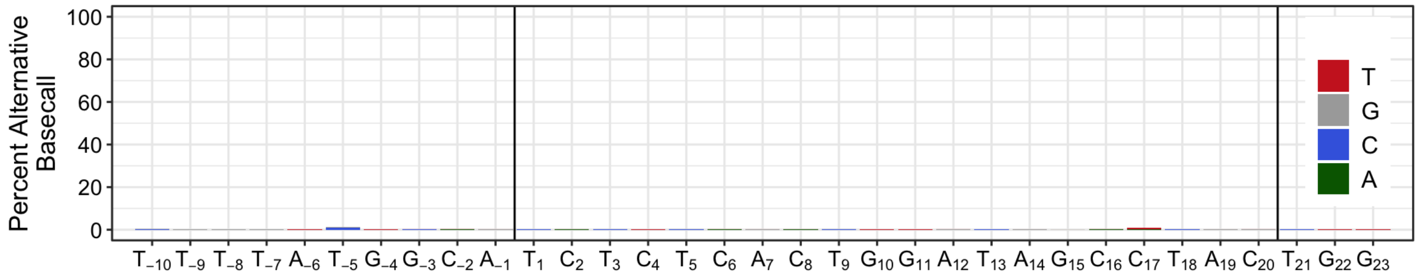

252. A B2M Ex.1 SD OT8 + Cas9 mRNA

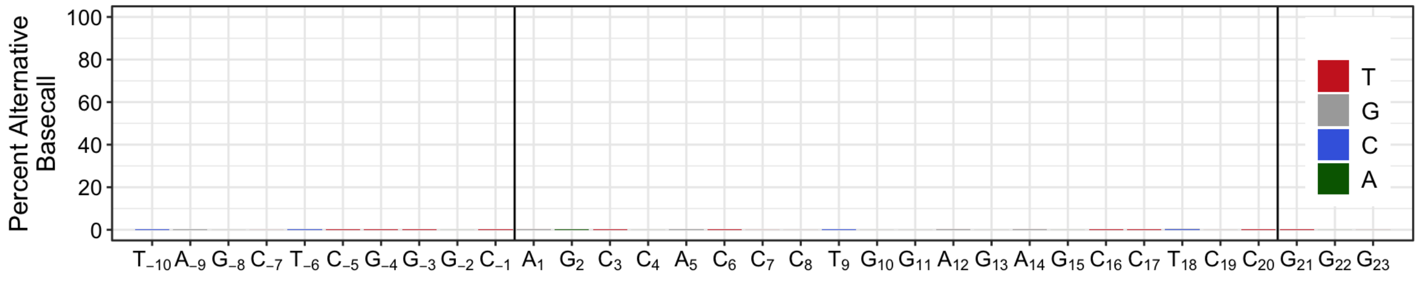

253. A B2M Ex.1 SD OT9 + Cas9 mRNA

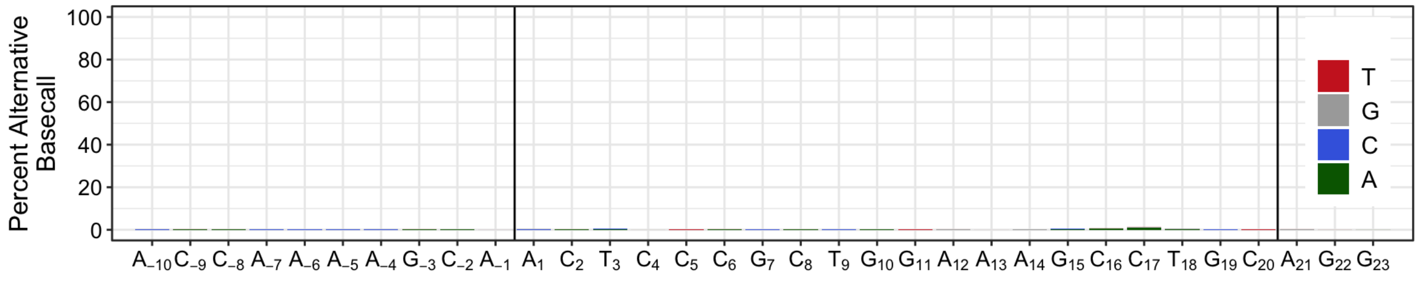

254. A B2M Ex.1 SD OT10 + Cas9 mRNA

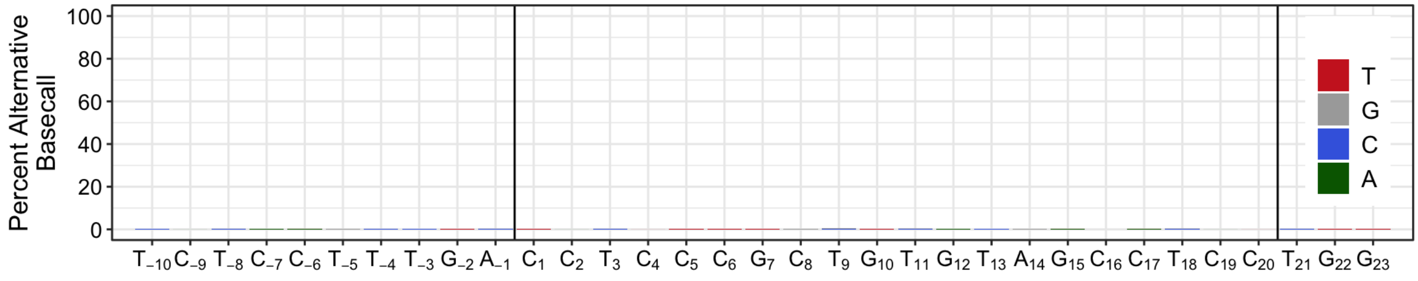

255. A B2M Ex.1 SD OnT + BE4 mRNA

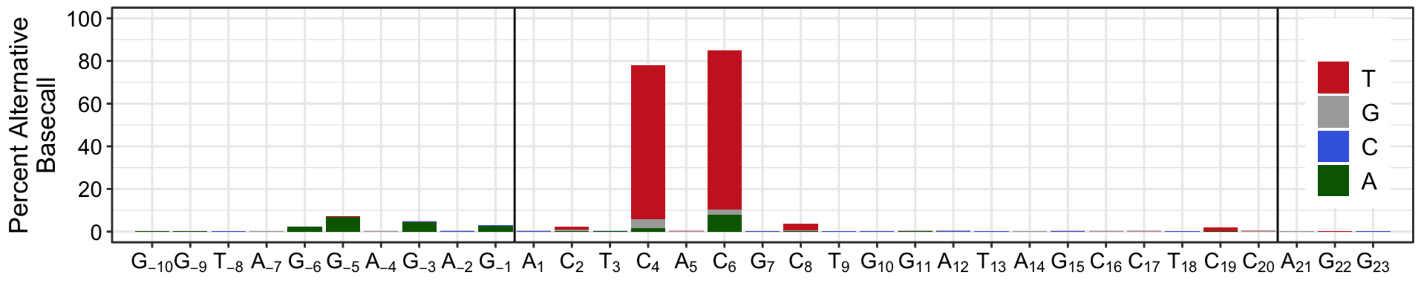

256. A B2M Ex.1 SD OT1 + BE4 mRNA

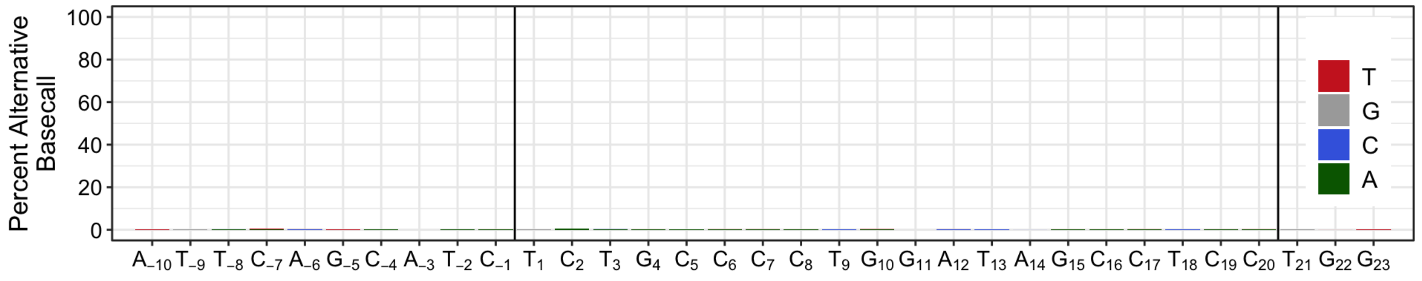

257. A B2M Ex.1 SD OT2 + BE4 mRNA

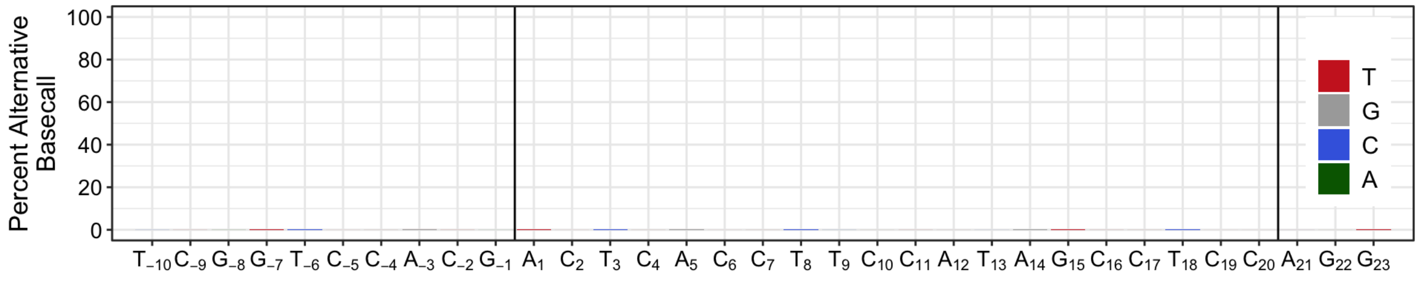

258. A B2M Ex.1 SD OT3 + BE4 mRNA

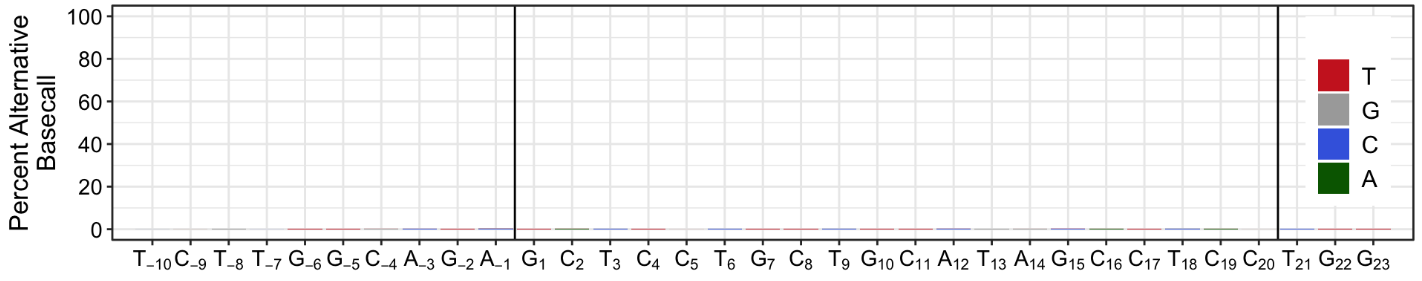

259. A B2M Ex.1 SD OT4 + BE4 mRNA

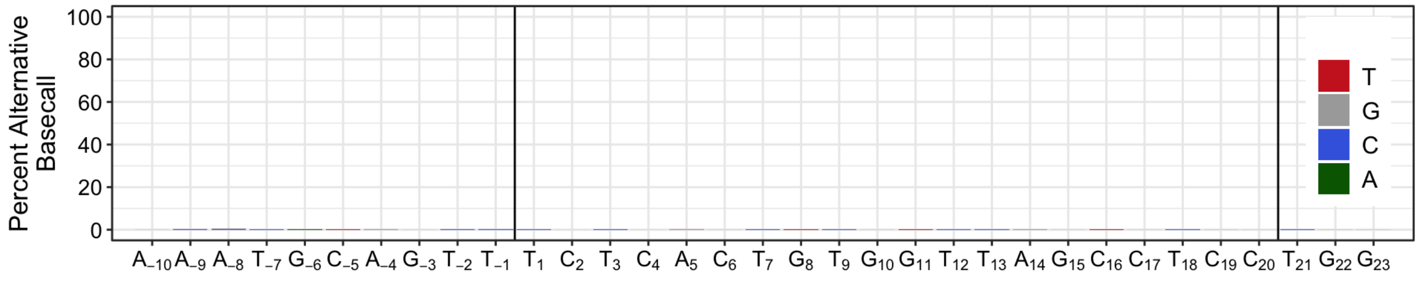

260. A B2M Ex.1 SD OT5 + BE4 mRNA

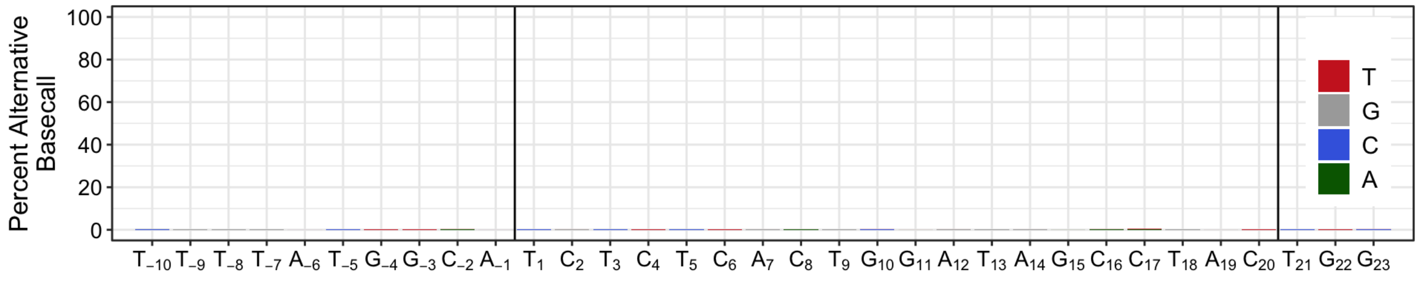

261. A B2M Ex.1 SD OT6 + BE4 mRNA

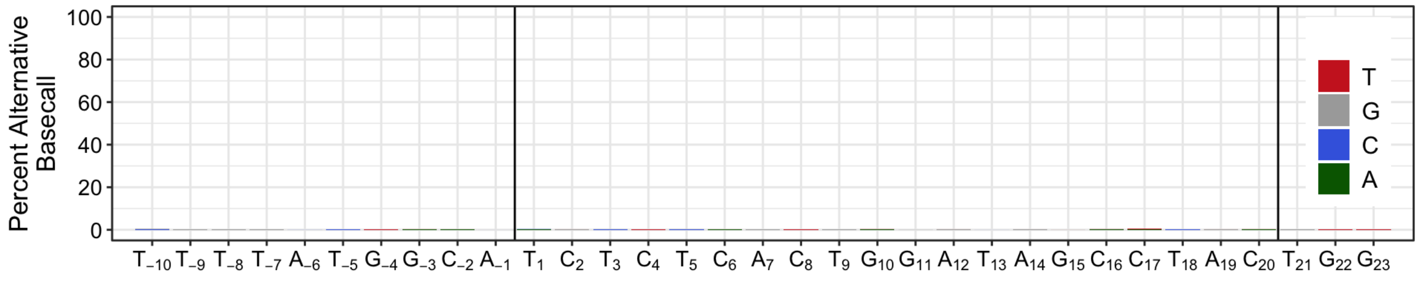

262. A B2M Ex.1 SD OT7 + BE4 mRNA

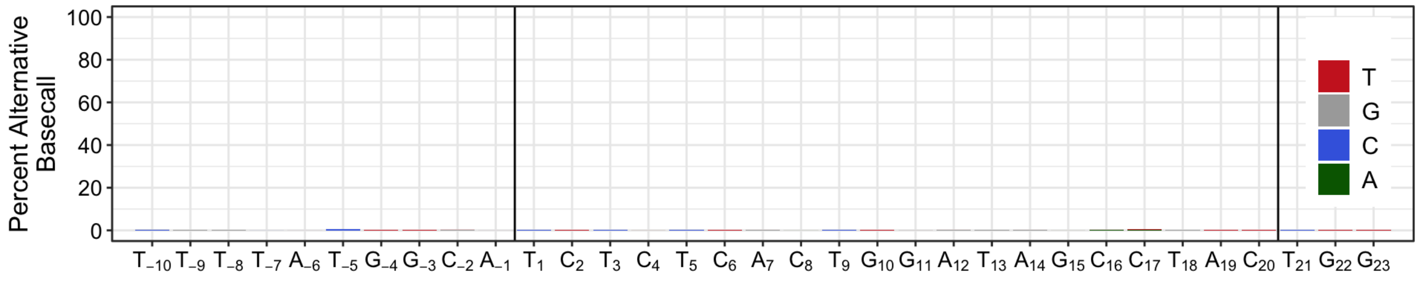

263. A B2M Ex.1 SD OT8 + BE4 mRNA

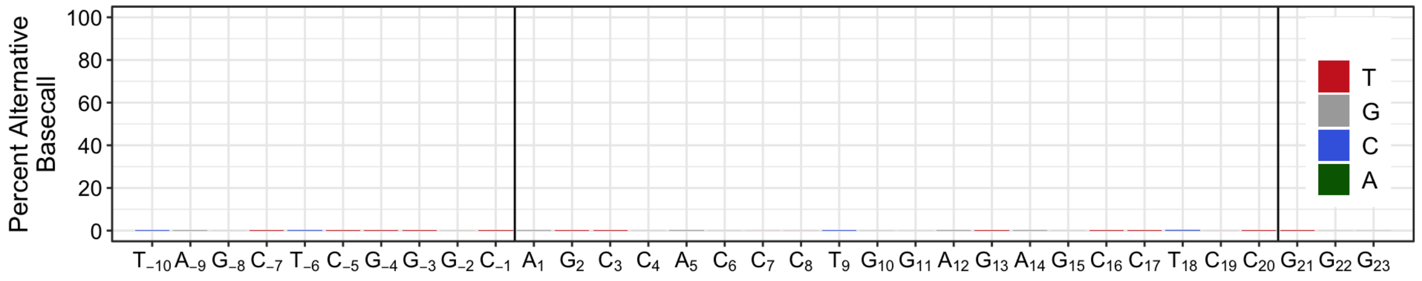

264. A B2M Ex.1 SD OT9 + BE4 mRNA

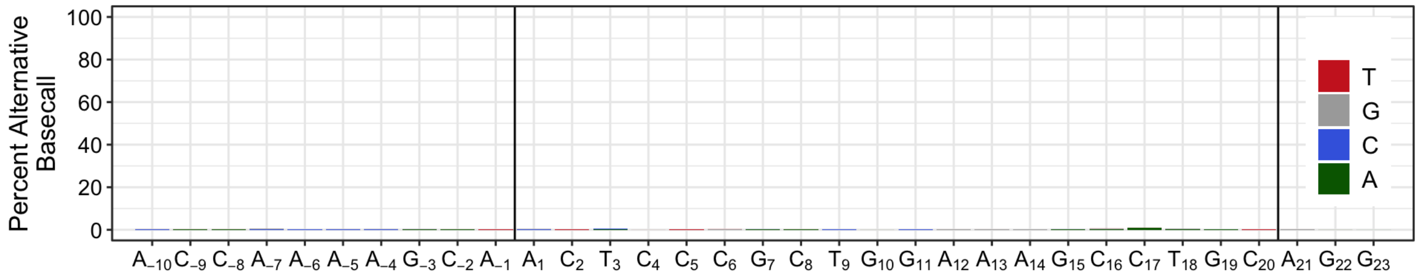

265. A B2M Ex.1 SD OT10 + BE4 mRNA

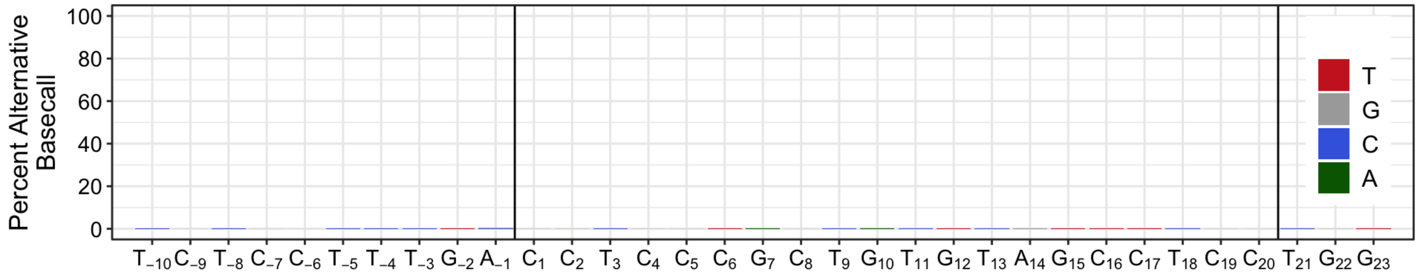

266. A TRAC Ex.3 SA OnT + Pulse

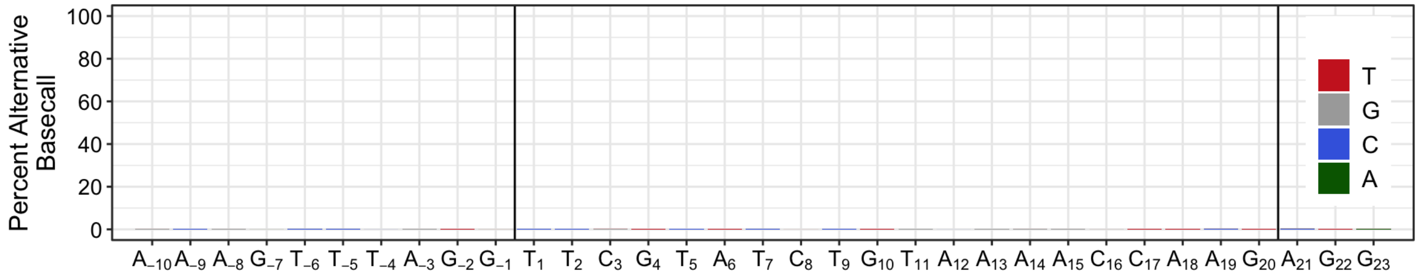

267. A TRAC Ex.3 SA OT1 + Pulse

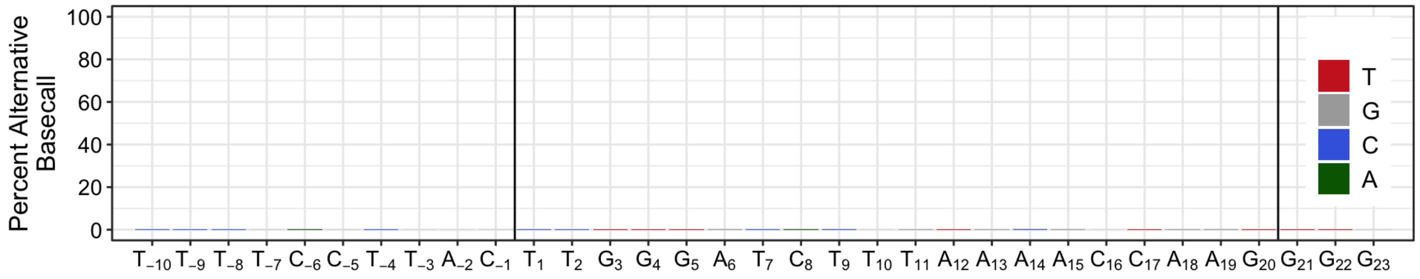

268. A TRAC Ex.3 SA OT2 + Pulse

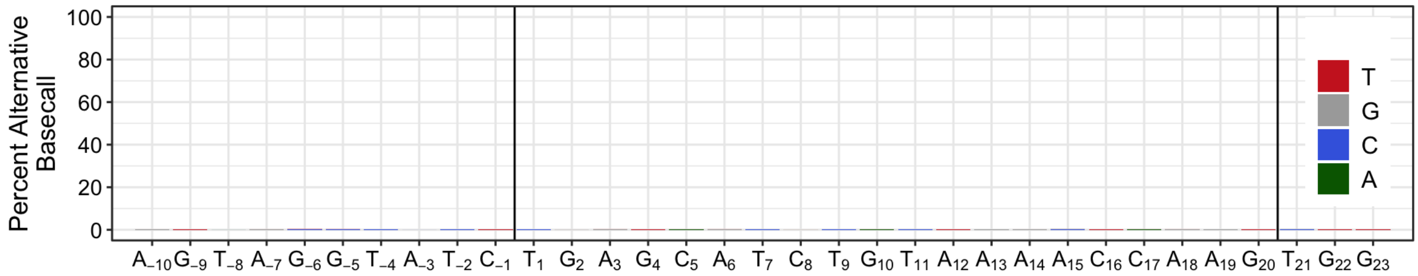

269. A TRAC Ex.3 SA OT3 + Pulse

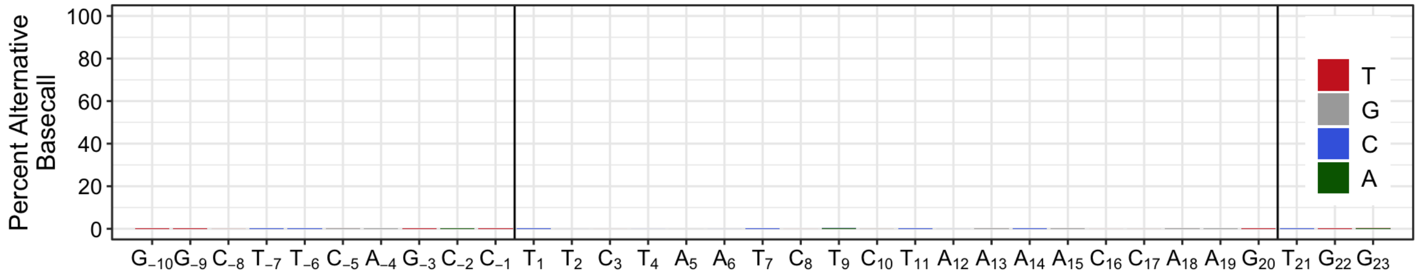

270. A TRAC Ex.3 SA OT4 + Pulse

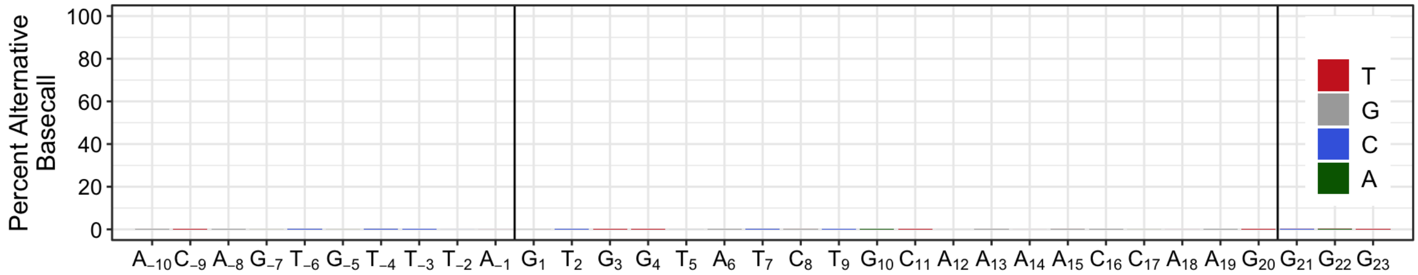

271. A TRAC Ex.3 SA OT5 + Pulse

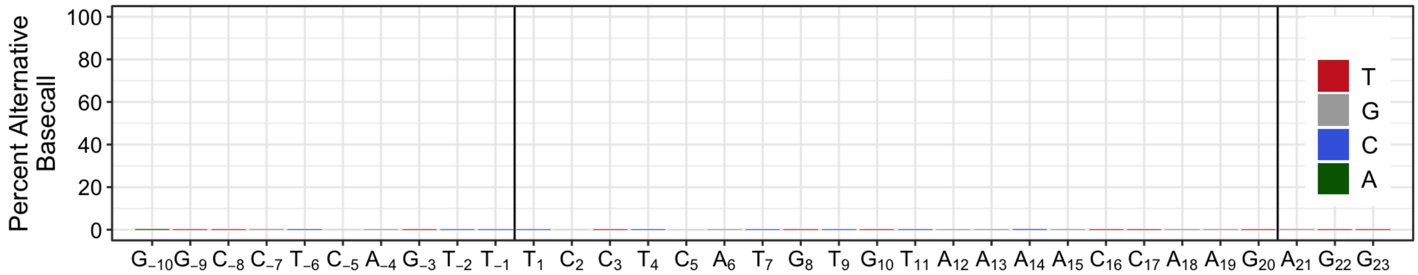

272. A TRAC Ex.3 SA OT6 + Pulse

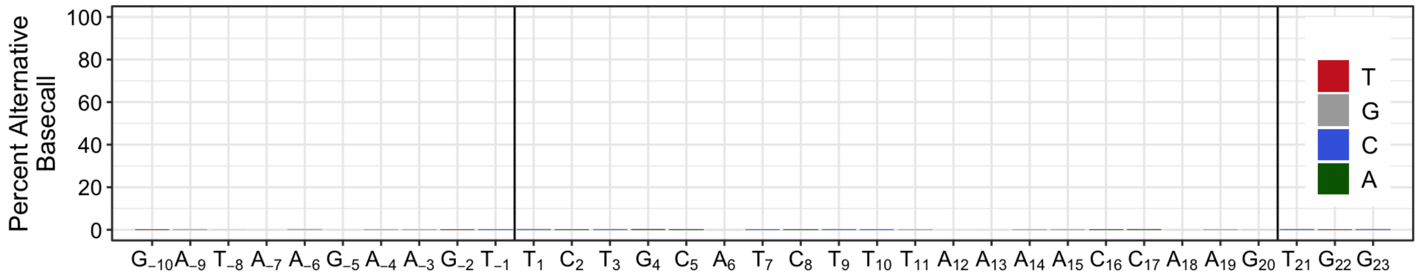

273. A TRAC Ex.3 SA OT7 + Pulse

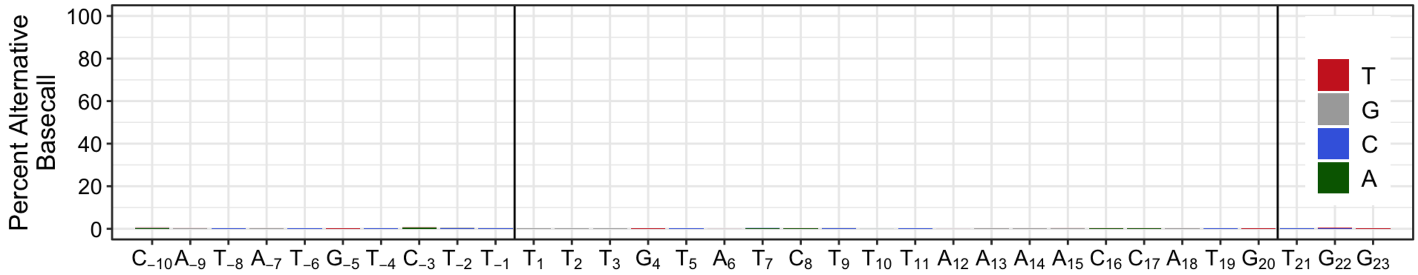

274. A TRAC Ex.3 SA OT8 + Pulse

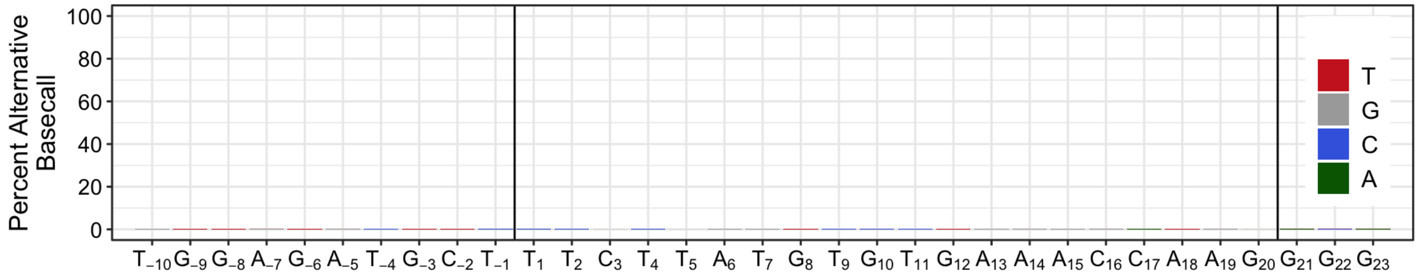

275. A TRAC Ex.3 SA OT9 + Pulse

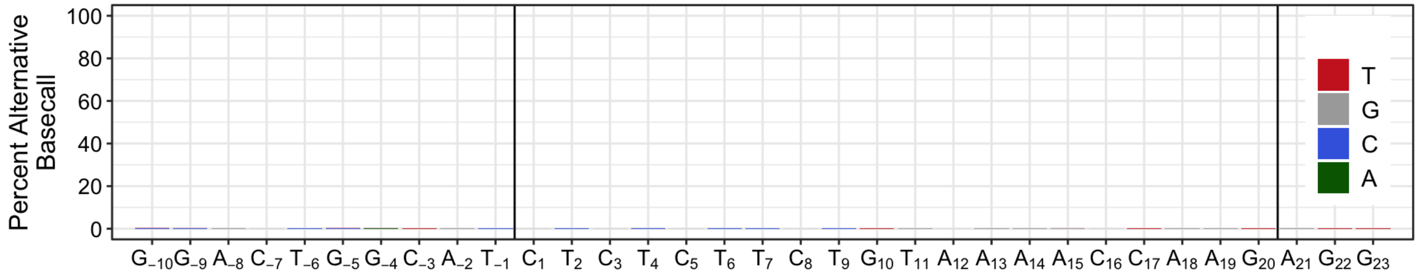

276. A TRAC Ex.3 SA OT10 + Pulse

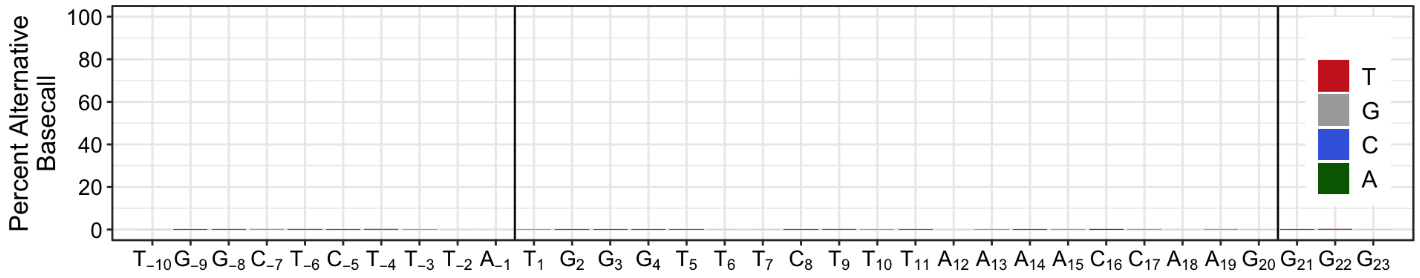

277. A TRAC Ex.3 SA OnT + Cas9 mRNA

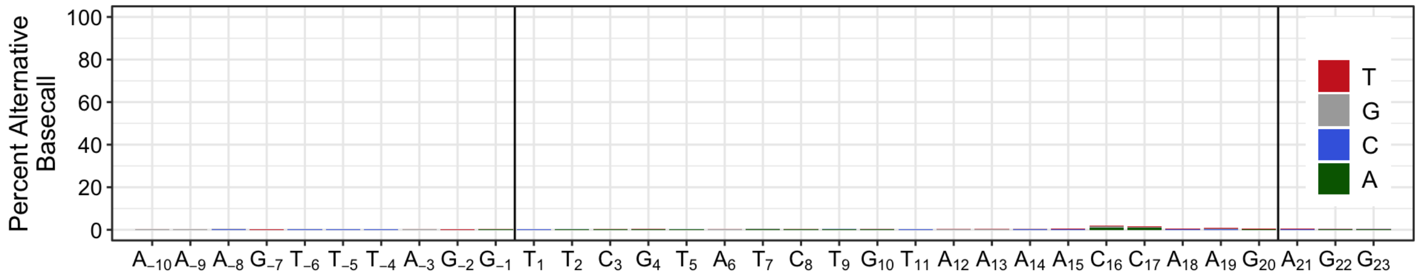

278. A TRAC Ex.3 SA OT1 + Cas9 mRNA

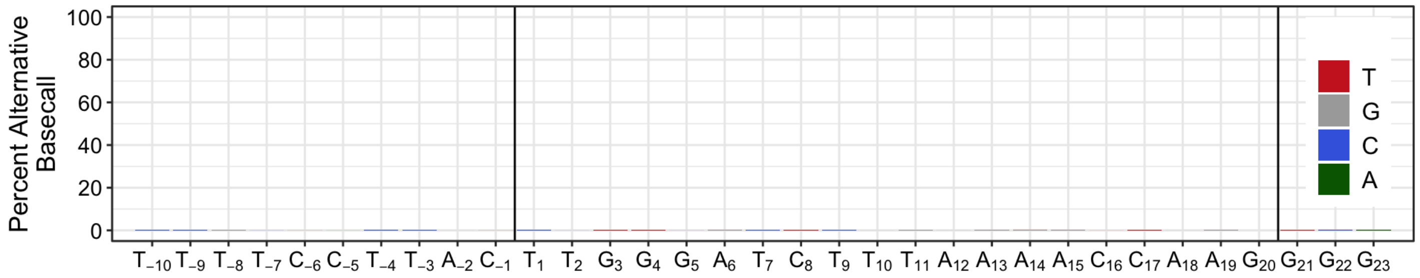

279. A TRAC Ex.3 SA OT2 + Cas9 mRNA

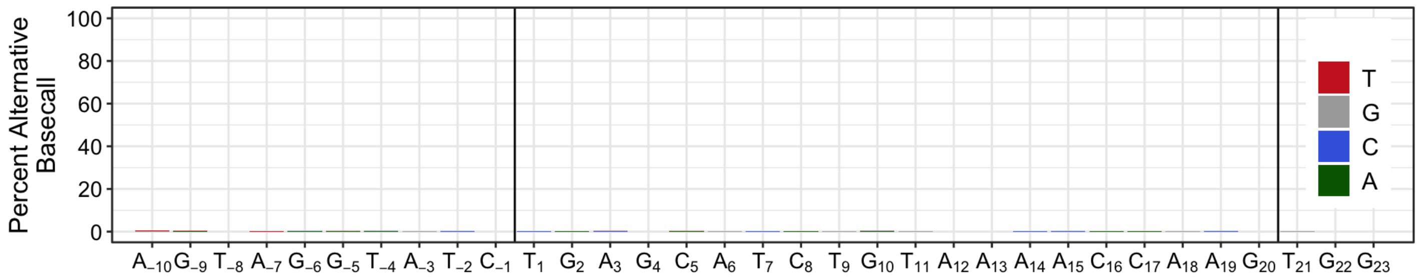

280. A TRAC Ex.3 SA OT3 + Cas9 mRNA

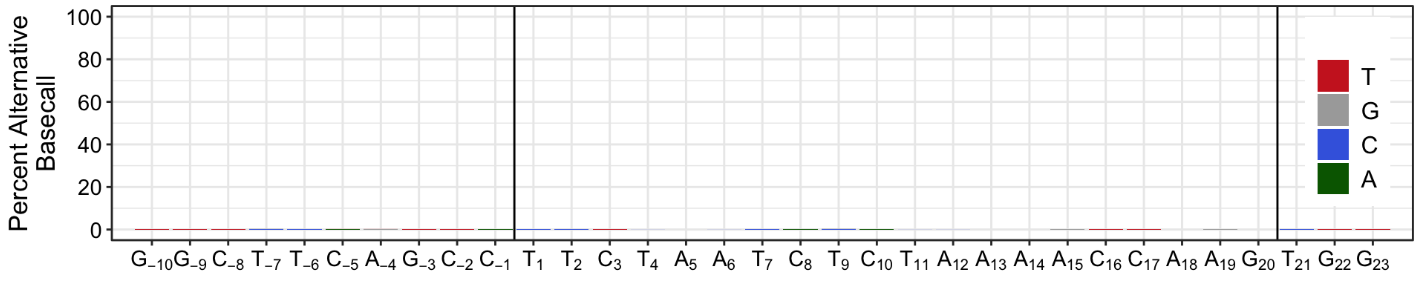

281. A TRAC Ex.3 SA OT4 + Cas9 mRNA

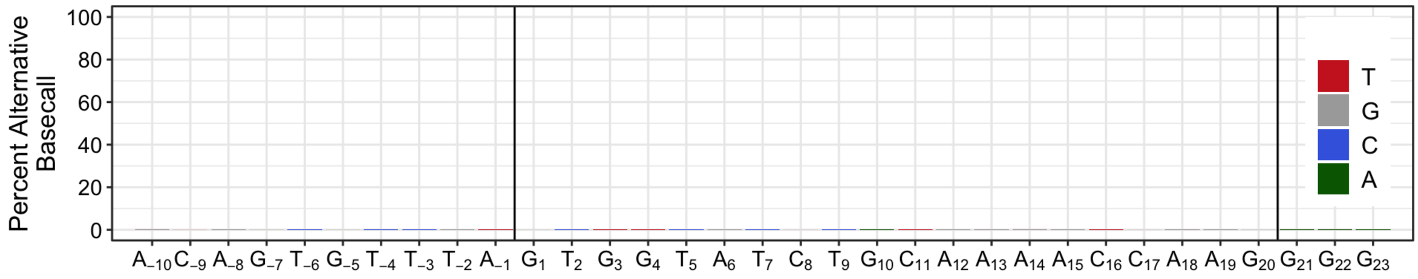

282. A TRAC Ex.3 SA OT5 + Cas9 mRNA

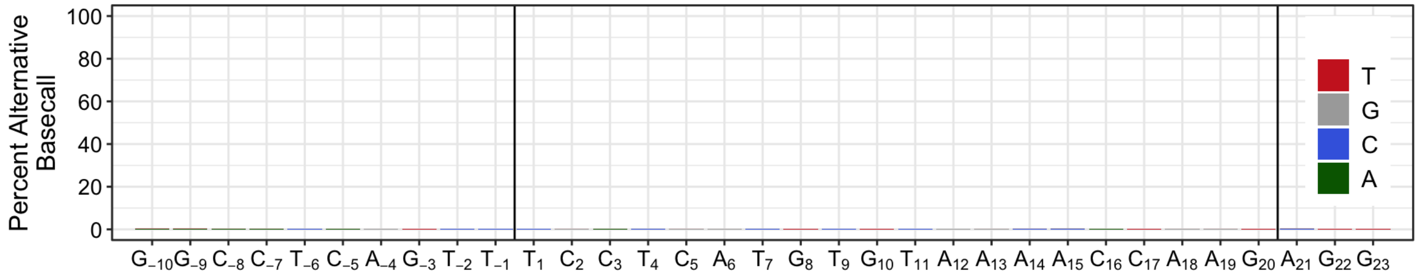

283. A TRAC Ex.3 SA OT6 + Cas9 mRNA

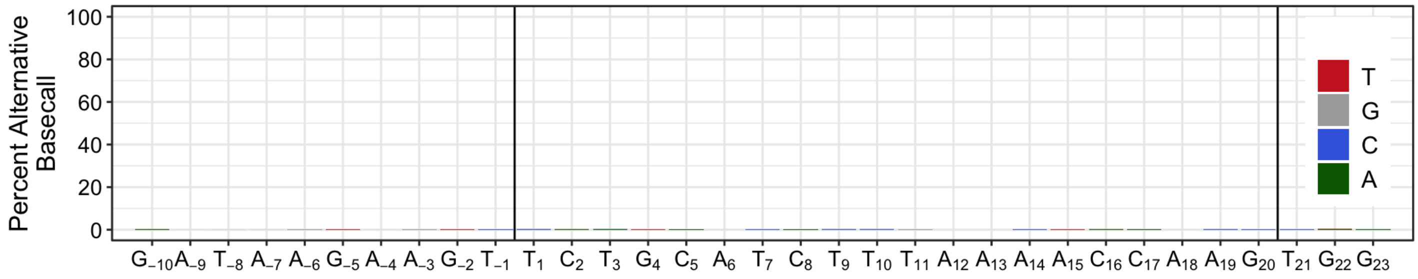

284. A TRAC Ex.3 SA OT7 + Cas9 mRNA

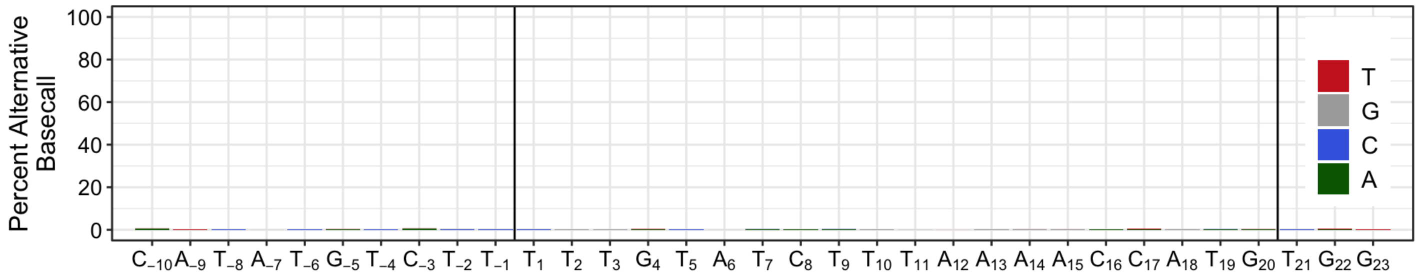

285. A TRAC Ex.3 SA OT8 + Cas9 mRNA

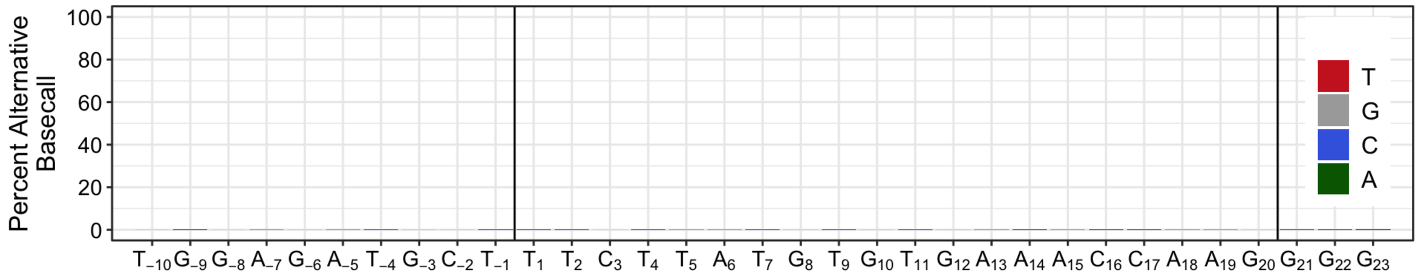

286. A TRAC Ex.3 SA OT9 + Cas9 mRNA

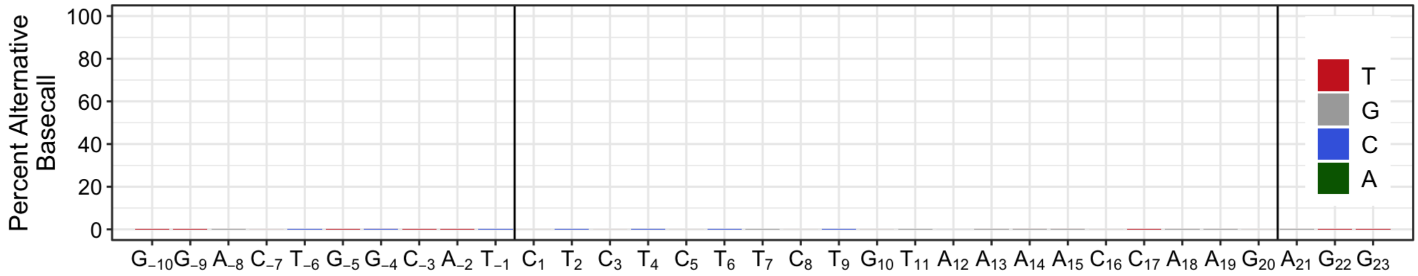

287. A TRAC Ex.3 SA OT10 + Cas9 mRNA

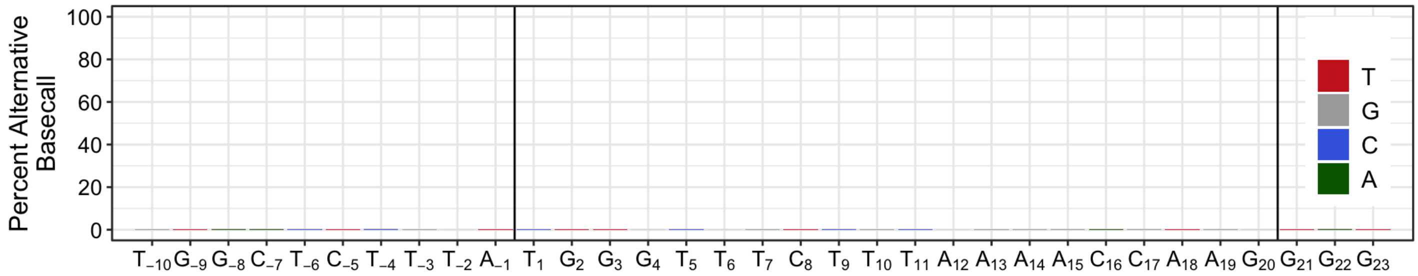

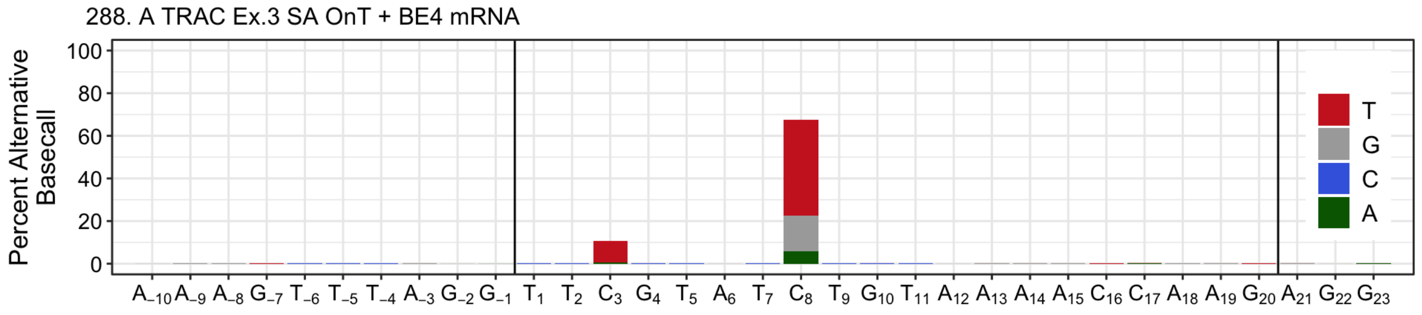

288. A TRAC Ex.3 SA OnT + BE4 mRNA
Percent Alternative Basecall
T
G
C
A

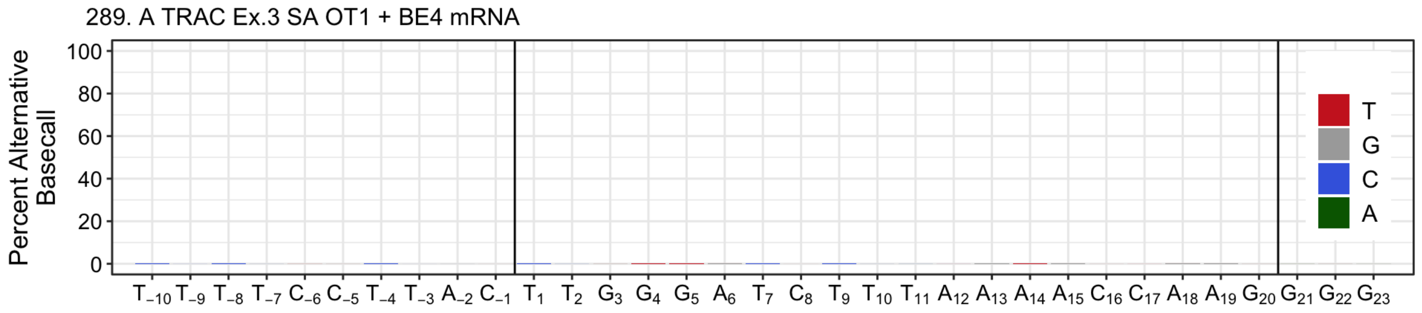

289. A TRAC Ex.3 SA OT1 + BE4 mRNA
Percent Alternative Basecall
T
G
C
A

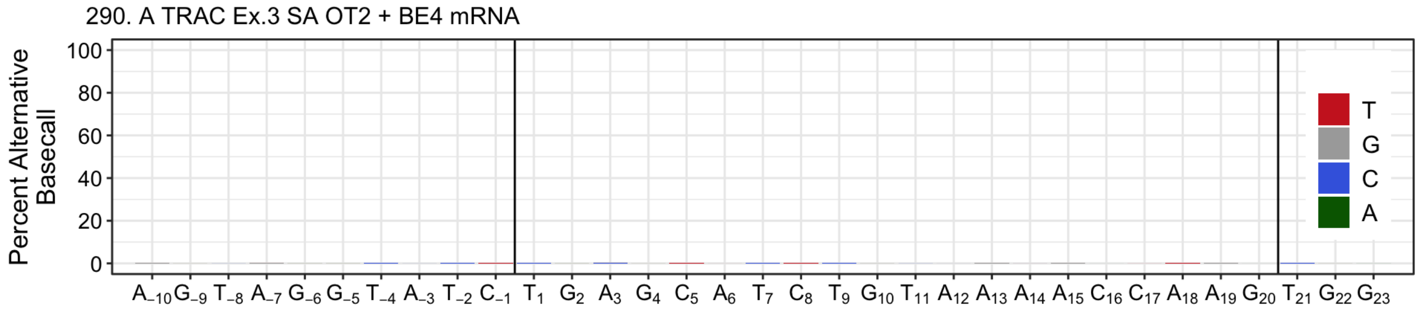

290. A TRAC Ex.3 SA OT2 + BE4 mRNA
Percent Alternative Basecall
T
G
C
A

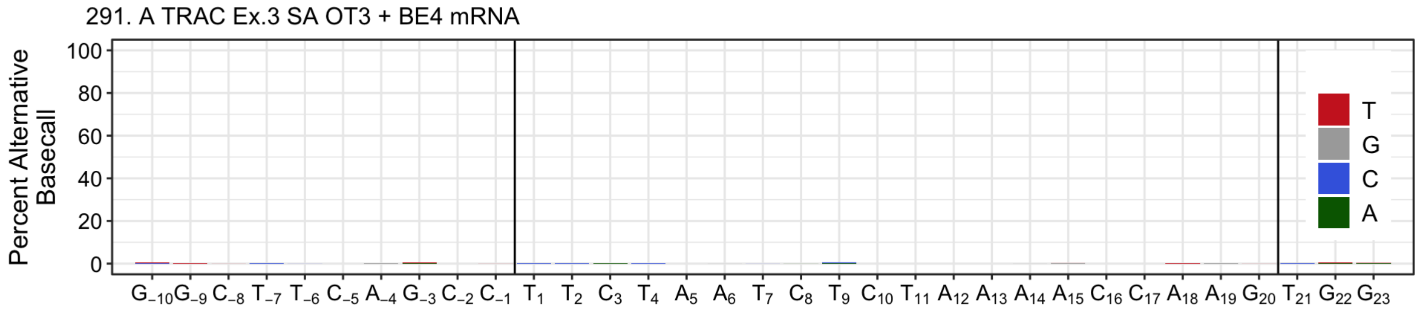

291. A TRAC Ex.3 SA OT3 + BE4 mRNA
Percent Alternative Basecall
T
G
C
A

292. A TRAC Ex.3 SA OT4 + BE4 mRNA

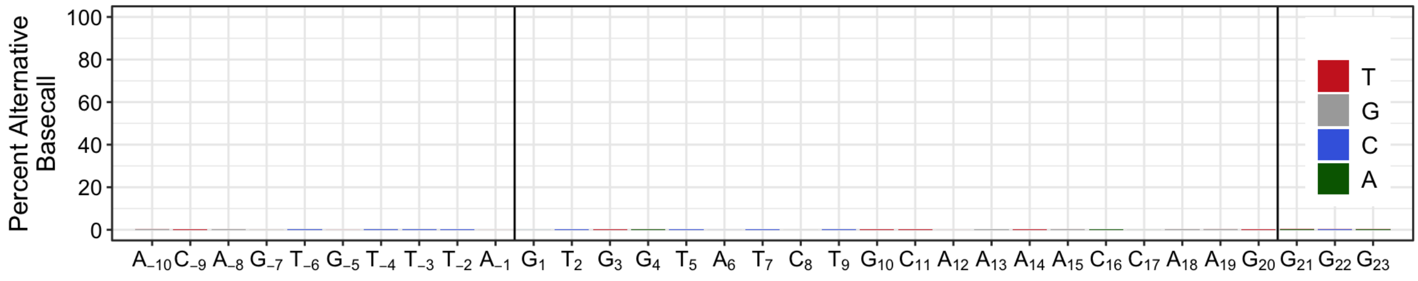

293. A TRAC Ex.3 SA OT5 + BE4 mRNA

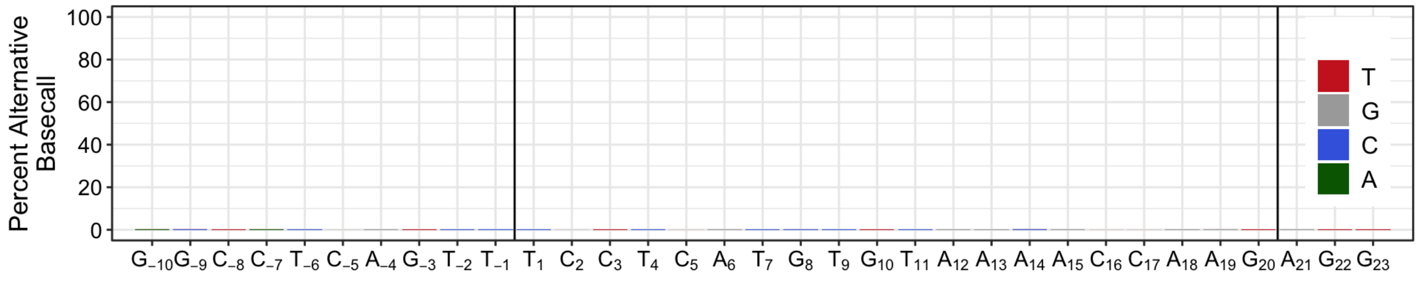

294. A TRAC Ex.3 SA OT6 + BE4 mRNA

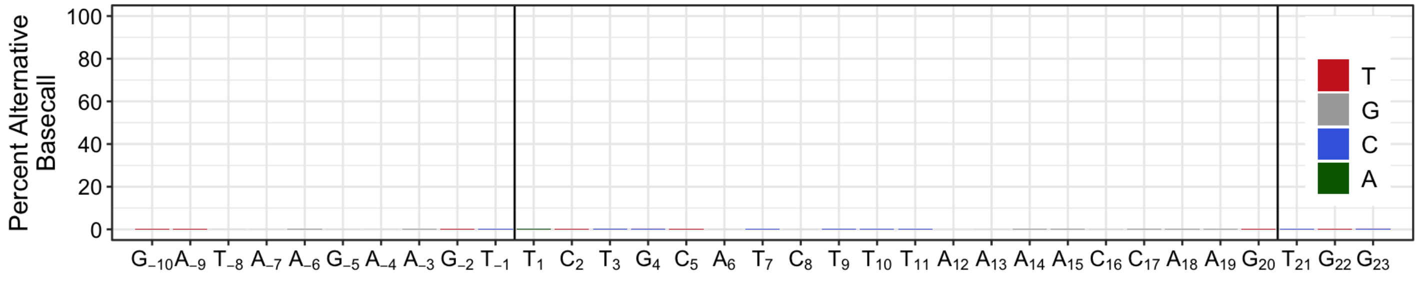

295. A TRAC Ex.3 SA OT7 + BE4 mRNA

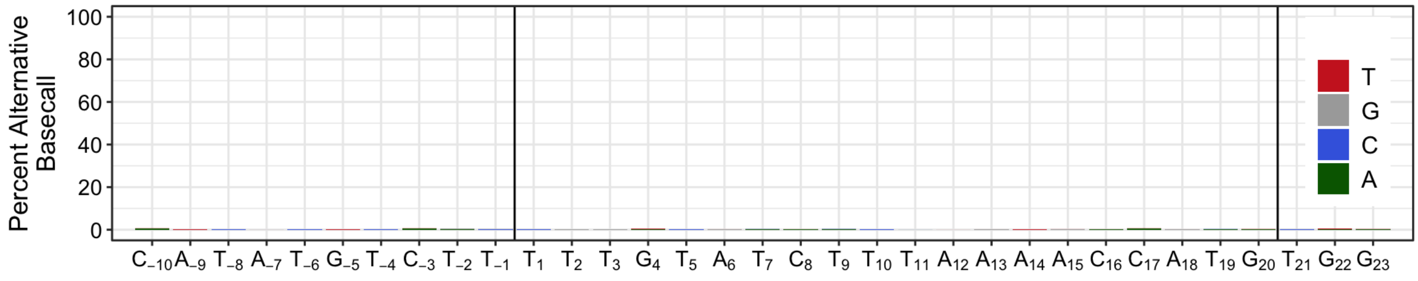

296. A TRAC Ex.3 SA OT8 + BE4 mRNA

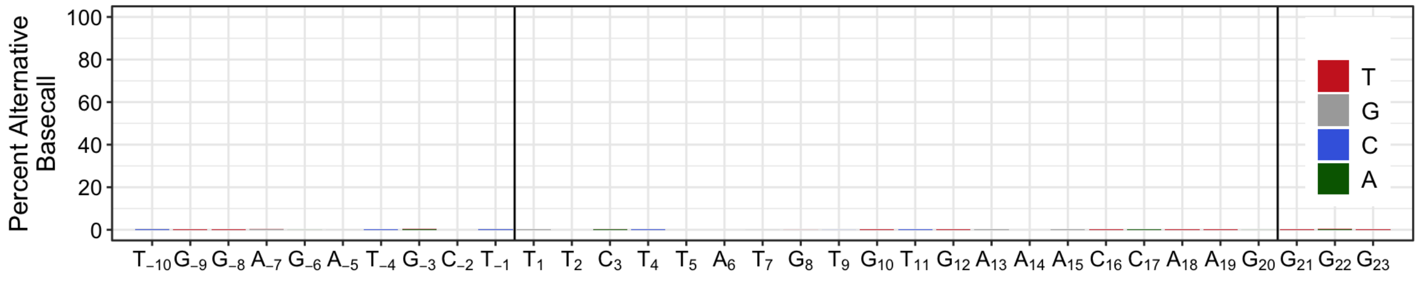

297. A TRAC Ex.3 SA OT9 + BE4 mRNA

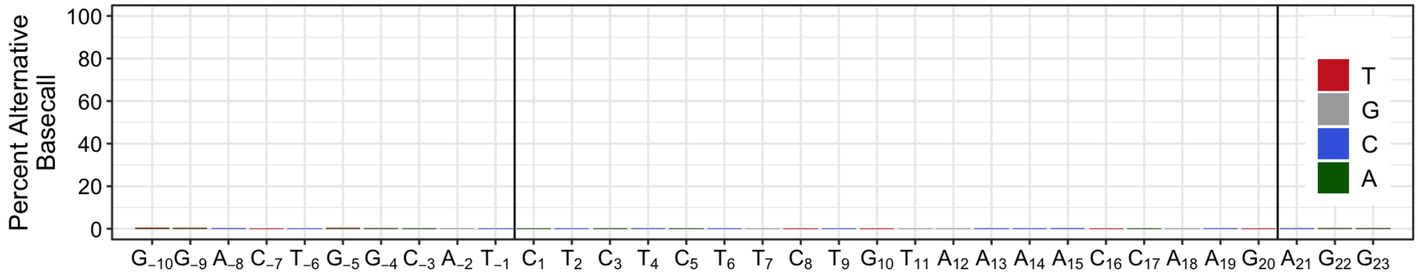

298. A TRAC Ex.3 SA OT10 + BE4 mRNA

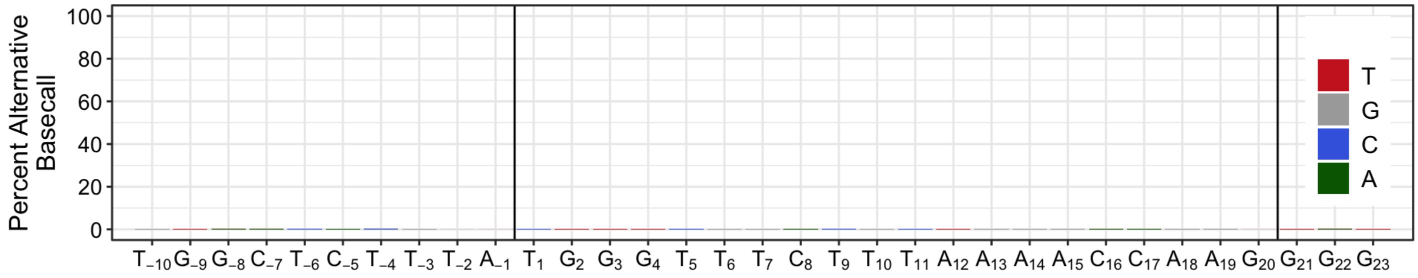

299. A PDCD1 Ex.1 SD OnT + Pulse

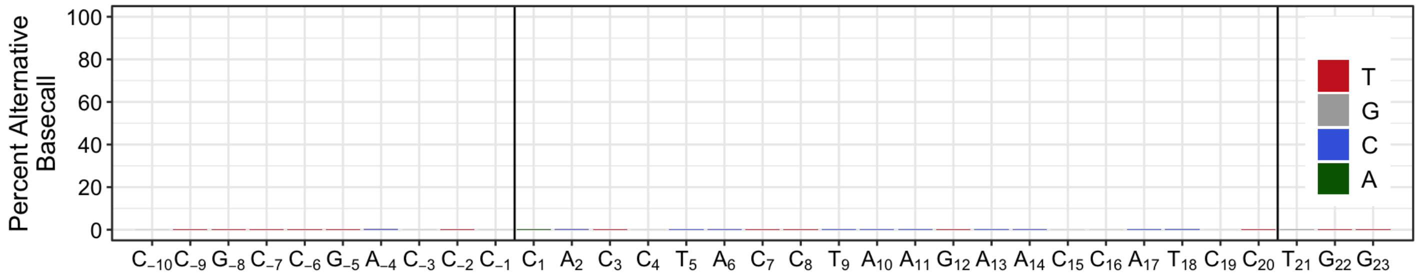

300. A PDCD1 Ex.1 SD OT1 + Pulse

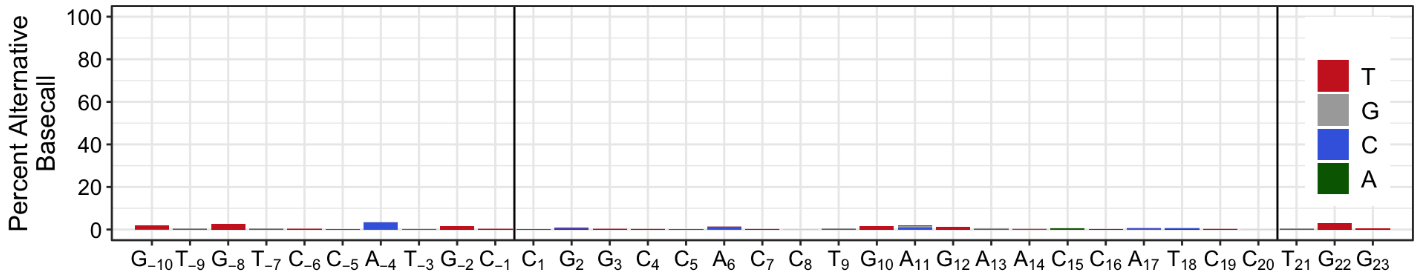

301. A PDCD1 Ex.1 SD OT2 + Pulse

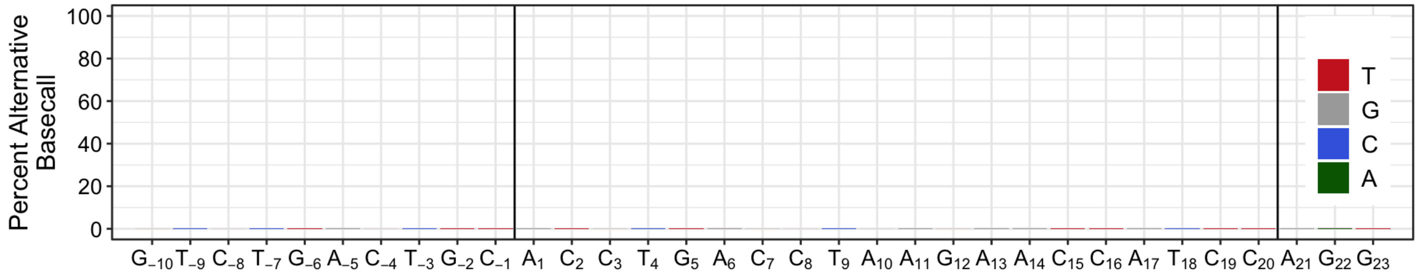

302. A PDCD1 Ex.1 SD OT3 + Pulse

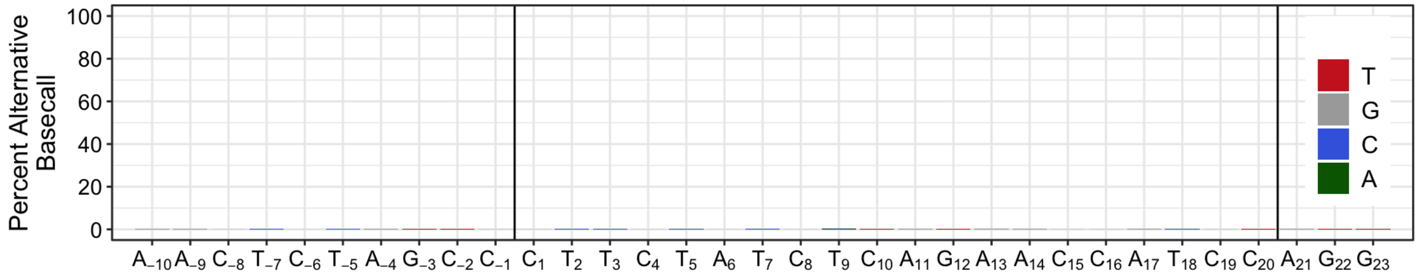

303. A PDCD1 Ex.1 SD OT4 + Pulse

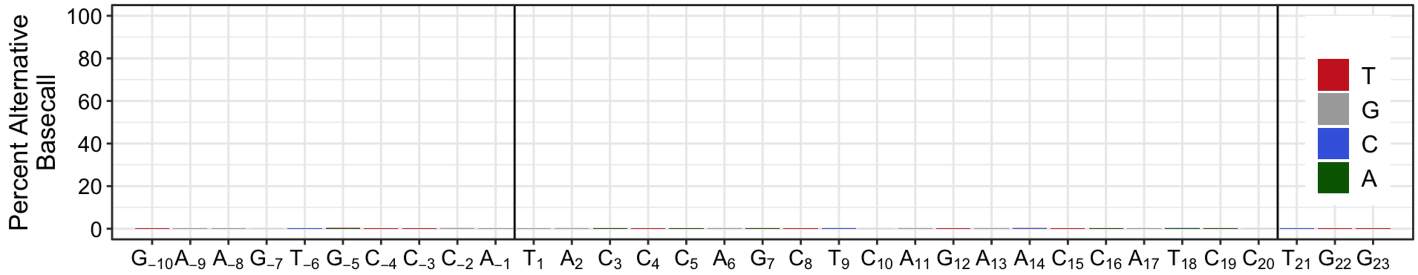

304. A PDCD1 Ex.1 SD OT5 + Pulse

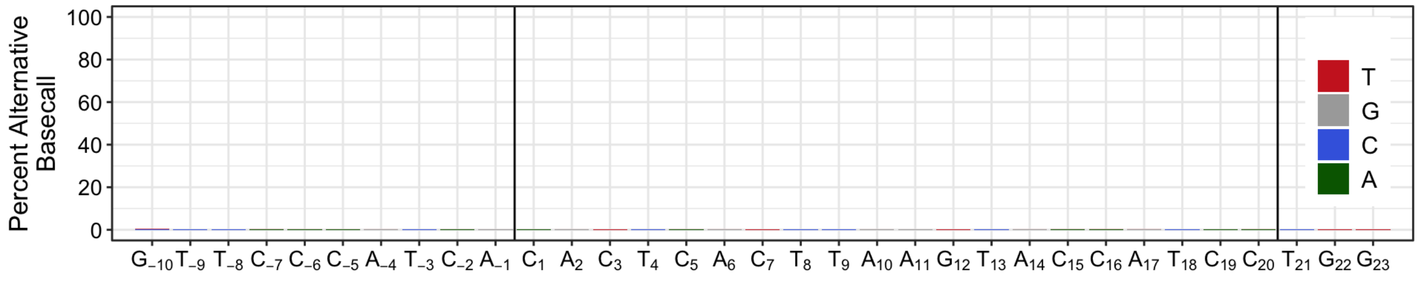

305. A PDCD1 Ex.1 SD OT6 + Pulse

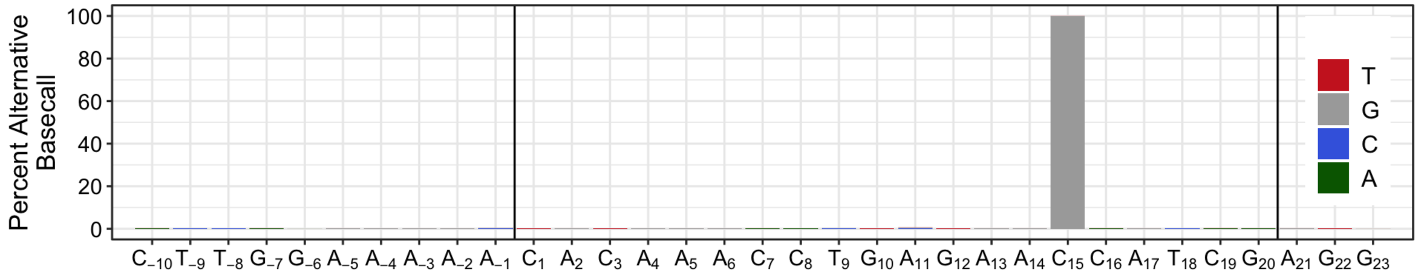

306. A PDCD1 Ex.1 SD OT7 + Pulse

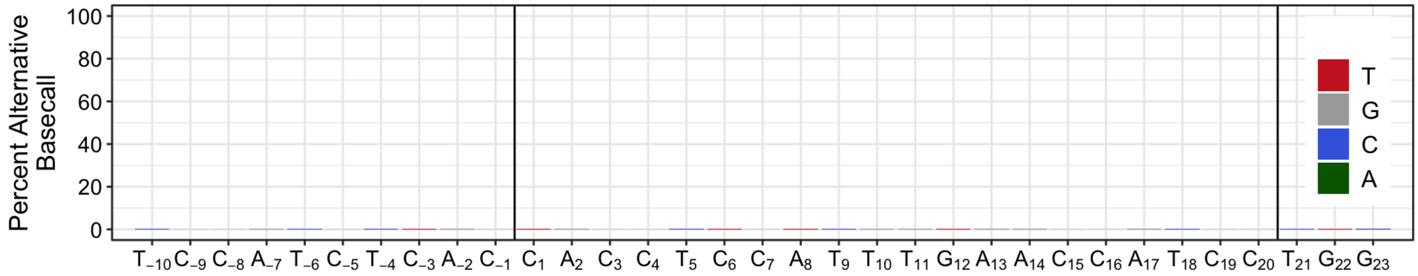

307. A PDCD1 Ex.1 SD OT8 + Pulse

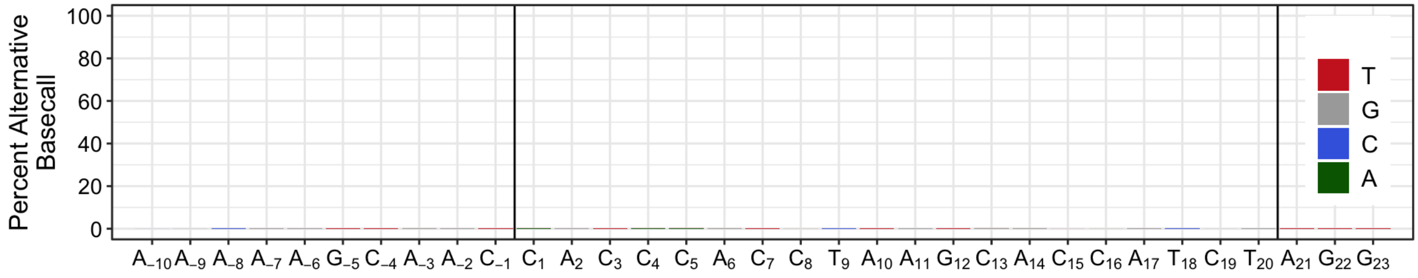

308. A PDCD1 Ex.1 SD OT9 + Pulse

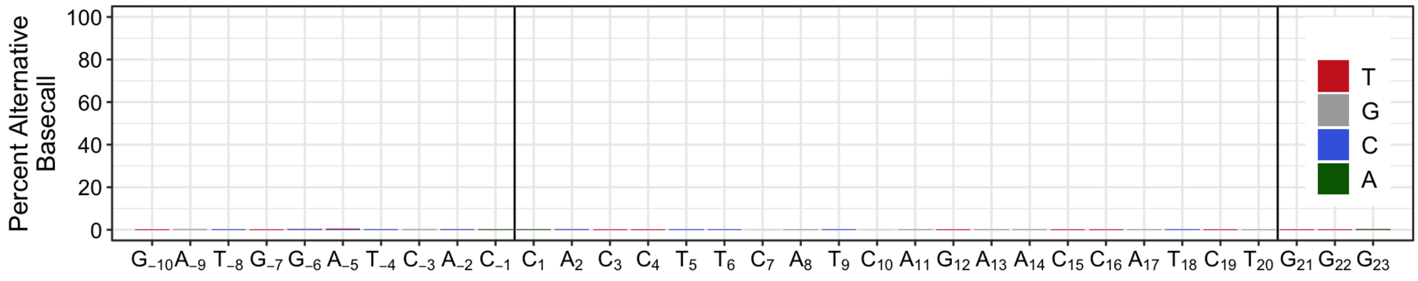

309. A PDCD1 Ex.1 SD OT10 + Pulse

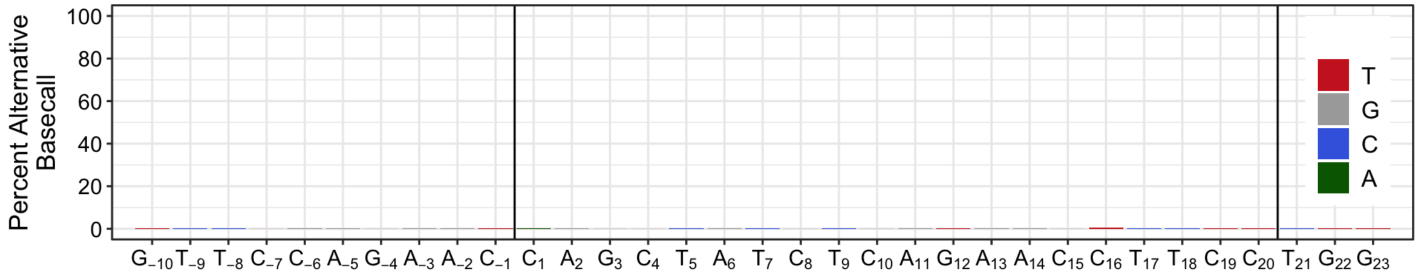

310. A PDCD1 Ex.1 SD OnT + Cas9 mRNA

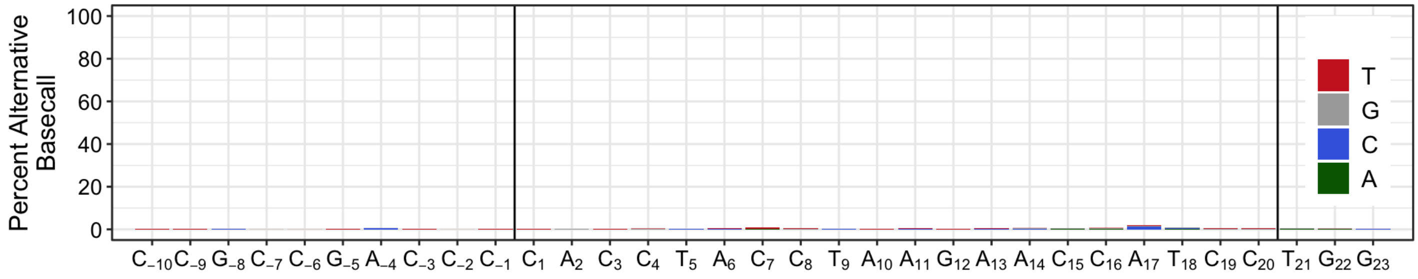

311. A PDCD1 Ex.1 SD OT1 + Cas9 mRNA

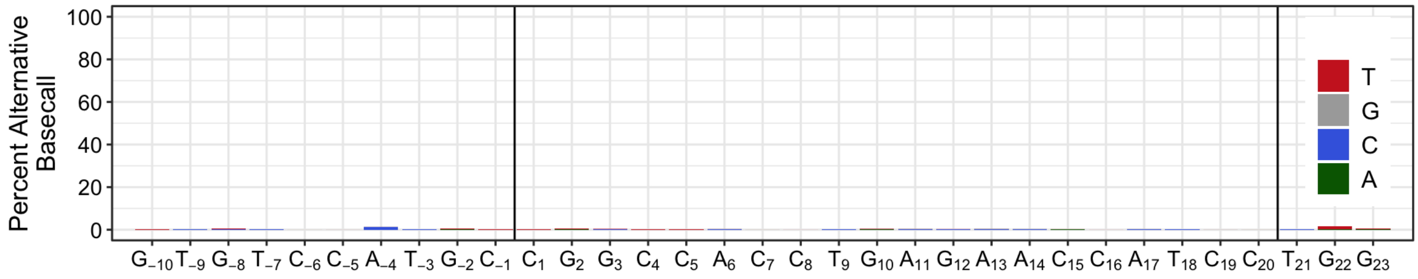

312. A PDCD1 Ex.1 SD OT2 + Cas9 mRNA

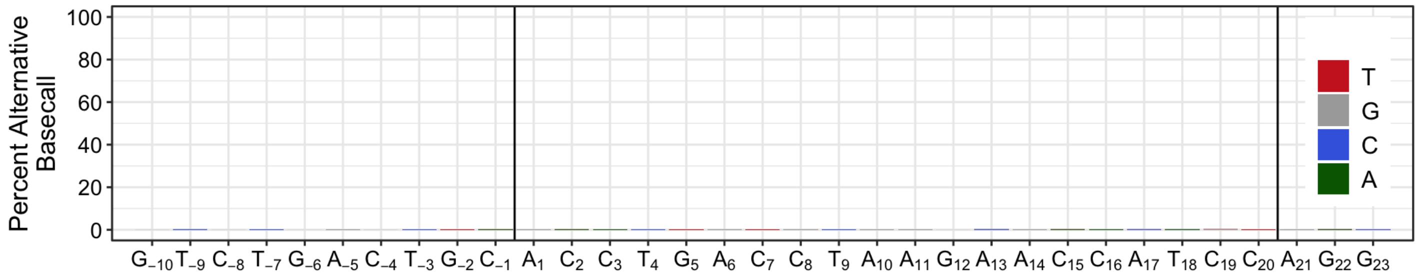

313. A PDCD1 Ex.1 SD OT3 + Cas9 mRNA

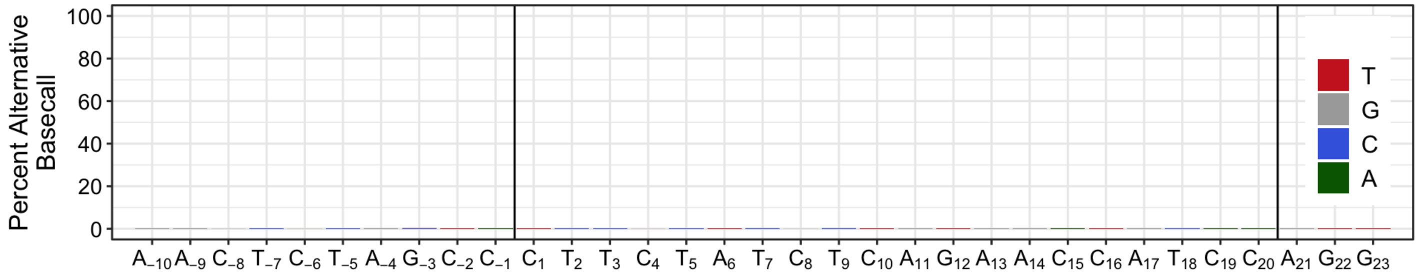

314. A PDCD1 Ex.1 SD OT4 + Cas9 mRNA

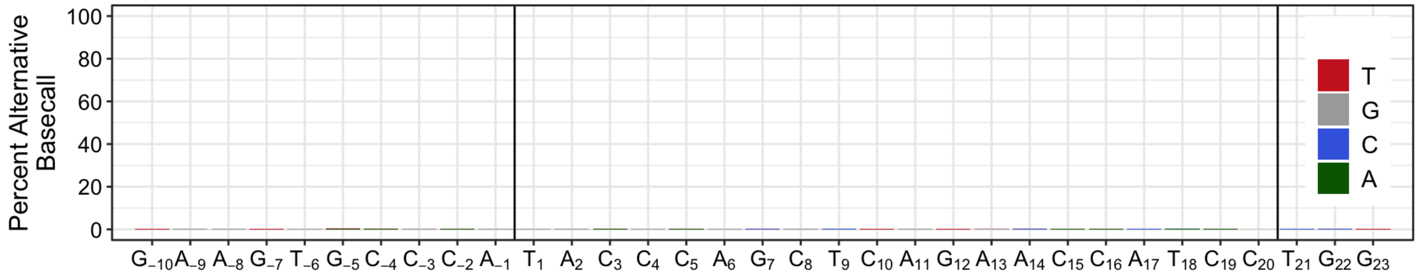

315. A PDCD1 Ex.1 SD OT5 + Cas9 mRNA

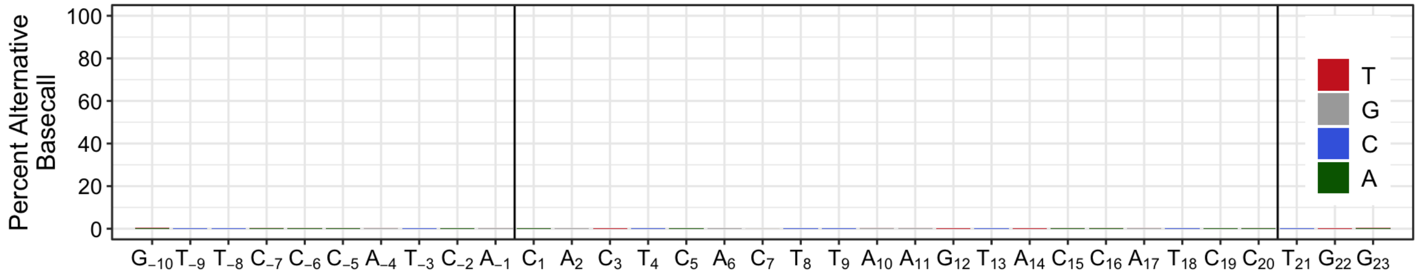

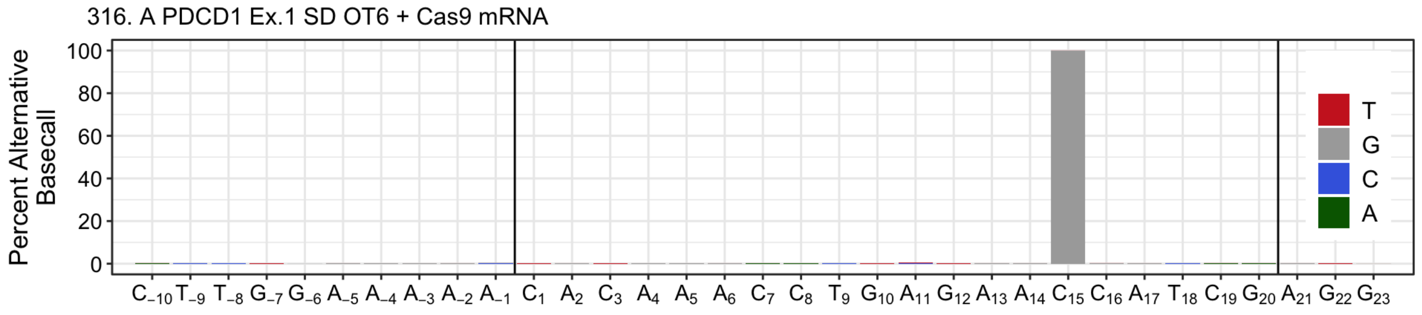
316. A PDCD1 Ex.1 SD OT6 + Cas9 mRNA
Percent Alternative Basecall
100
80
60
40
20
0
T
G
C
A

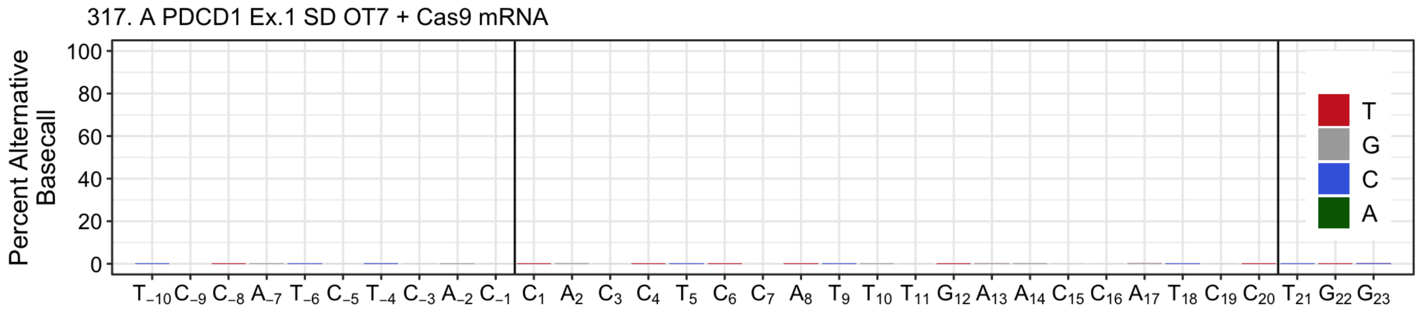
317. A PDCD1 Ex.1 SD OT7 + Cas9 mRNA
Percent Alternative Basecall
100
80
60
40
20
0
T
G
C
A

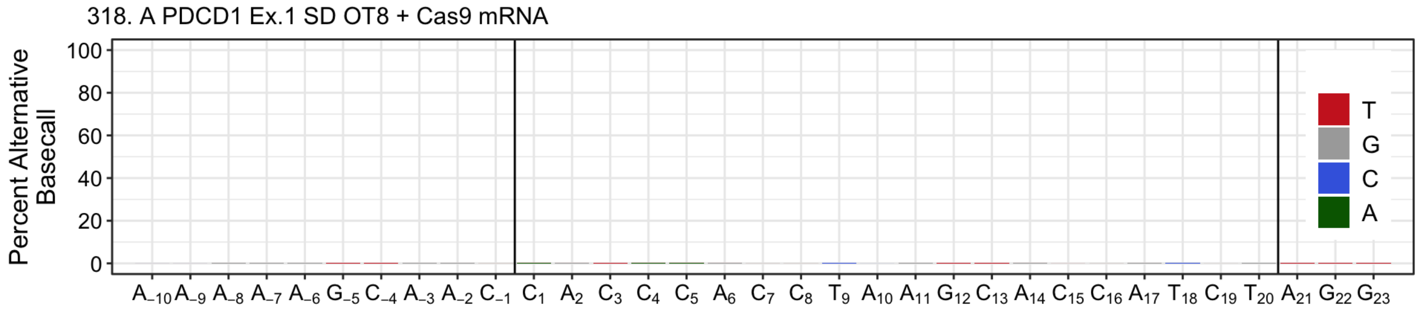
318. A PDCD1 Ex.1 SD OT8 + Cas9 mRNA
Percent Alternative Basecall
100
80
60
40
20
0
T
G
C
A

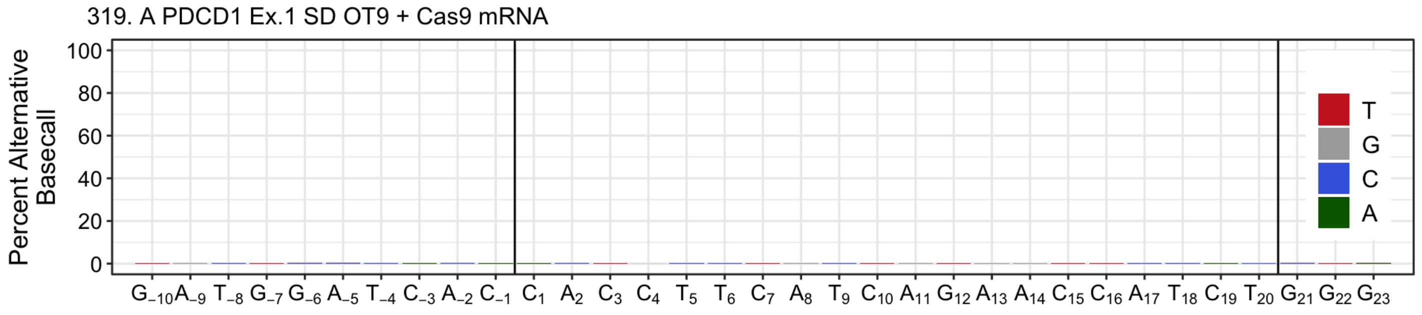
319. A PDCD1 Ex.1 SD OT9 + Cas9 mRNA
Percent Alternative Basecall
100
80
60
40
20
0
T
G
C
A

320. A PDCD1 Ex.1 SD OT10 + Cas9 mRNA

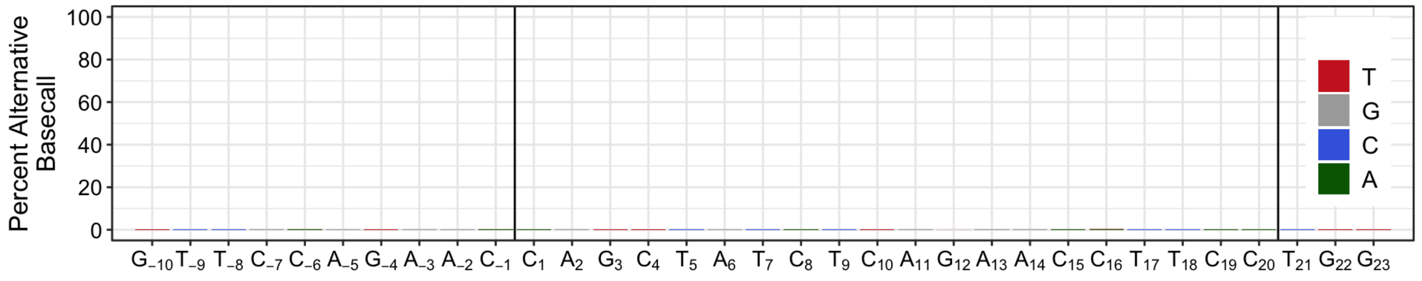

321. A PDCD1 Ex.1 SD OnT + BE4 mRNA

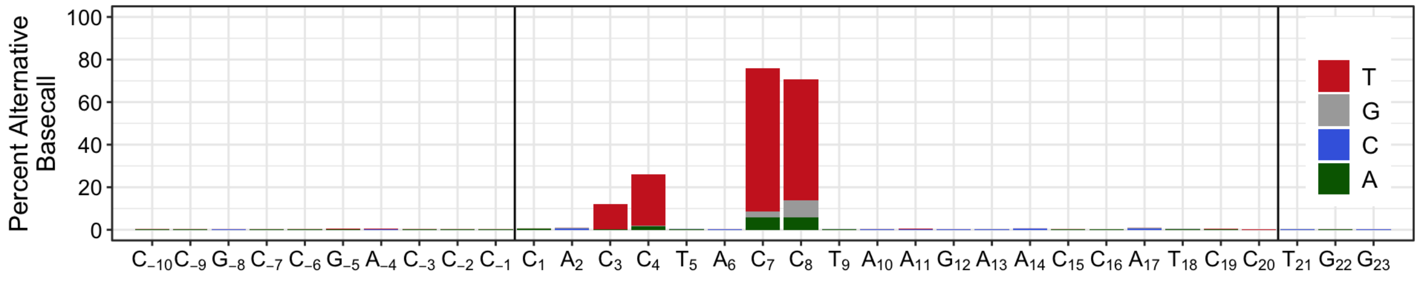

322. A PDCD1 Ex.1 SD OT1 + BE4 mRNA

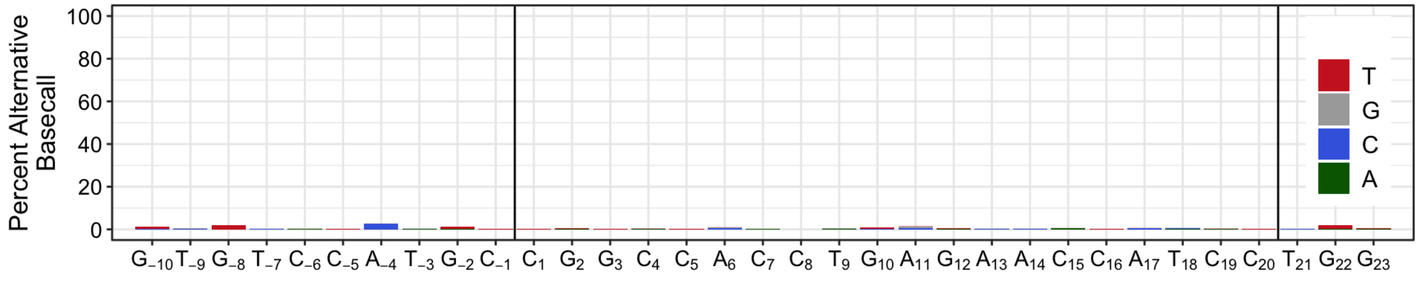

323. A PDCD1 Ex.1 SD OT2 + BE4 mRNA

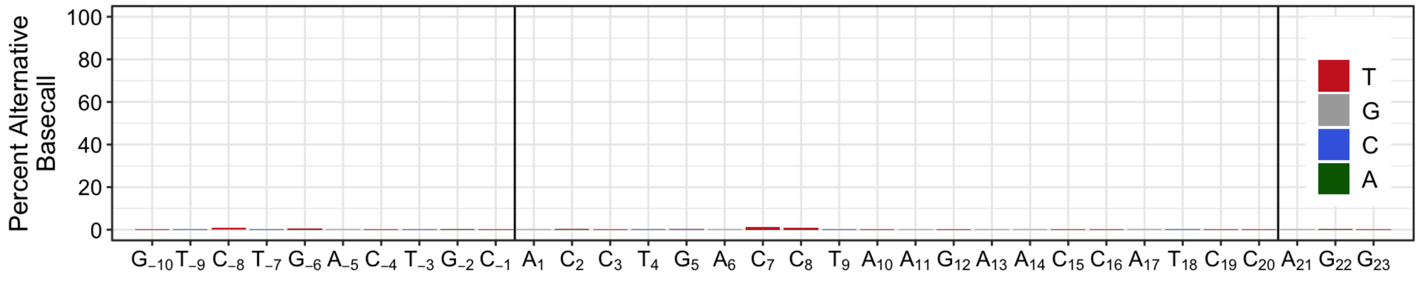

324. A PDCD1 Ex.1 SD OT3 + BE4 mRNA

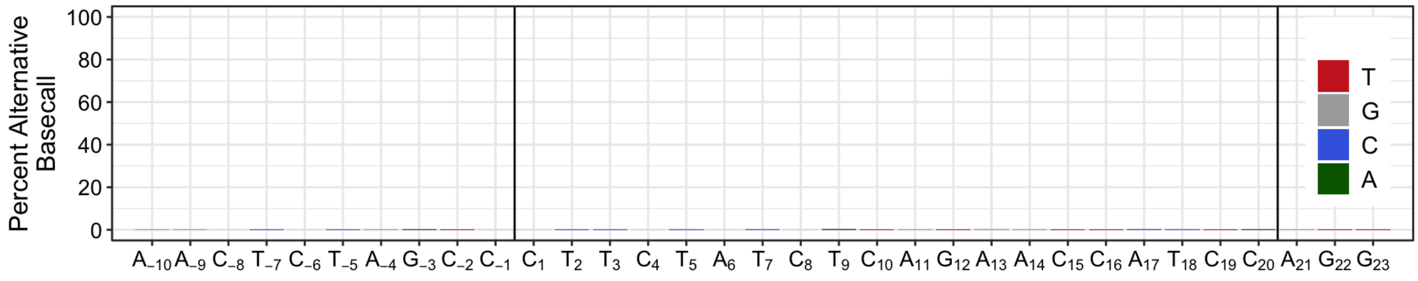

325. A PDCD1 Ex.1 SD OT4 + BE4 mRNA

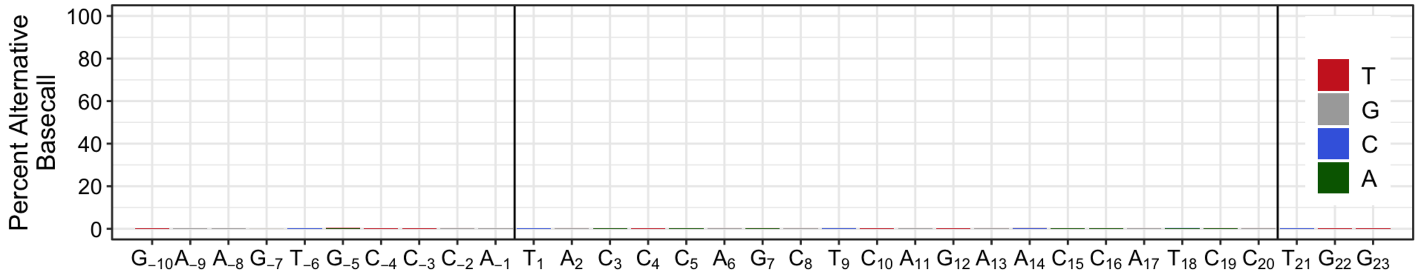

326. A PDCD1 Ex.1 SD OT5 + BE4 mRNA

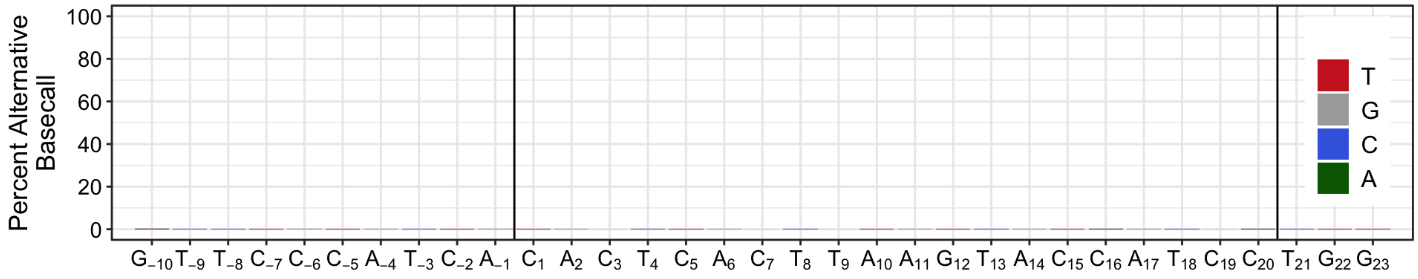

327. A PDCD1 Ex.1 SD OT6 + BE4 mRNA

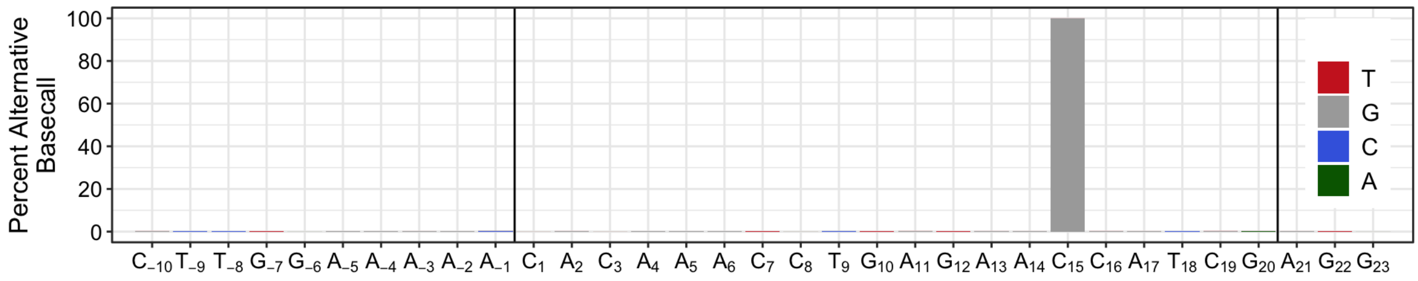

328. A PDCD1 Ex.1 SD OT7 + BE4 mRNA

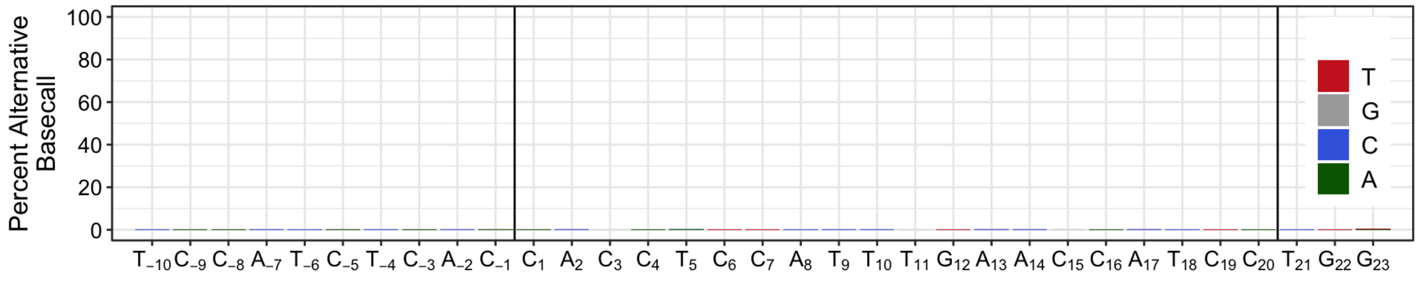

329. A PDCD1 Ex.1 SD OT8 + BE4 mRNA

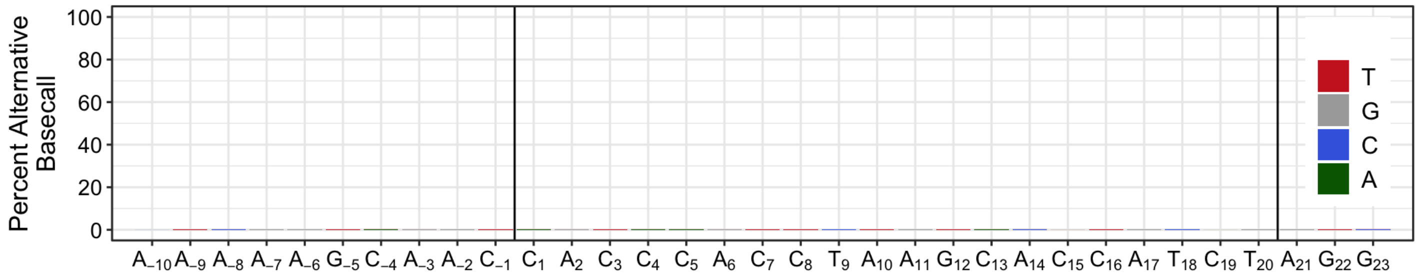

330. A PDCD1 Ex.1 SD OT9 + BE4 mRNA

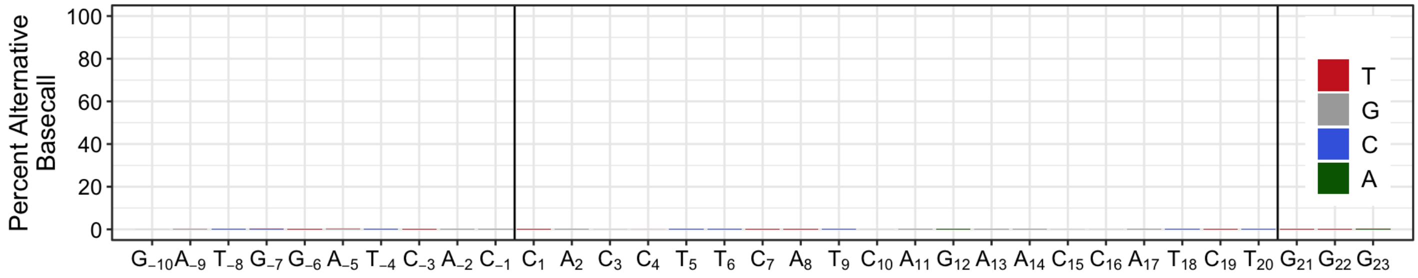

331. A PDCD1 Ex.1 SD OT10 + BE4 mRNA

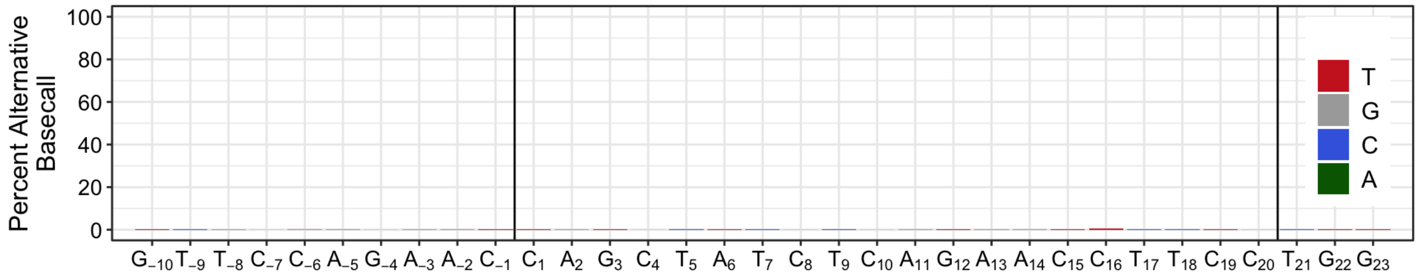

Supplement: Supplementary file 4 — Dataset 2 [file 41467_2019_13007_MOESM4_ESM.pdf]
