# Supplementary material for: Highly efficient multiplex human T cell engineering without double-strand breaks using Cas9 base editors
Source: Nat Commun. 2019 Nov 19;10:5222. doi: 10.1038/s41467-019-13007-6 (PMC6864045; doi:10.1038/s41467-019-13007-6)
Supplement: Supplementary file 5 — Description of Additional Supplementary Files [file 41467_2019_13007_MOESM5_ESM.pdf]

File Name: Supplementary Data 1
Description: Tile plots of Next Generation Sequencing

File Name: Supplementary Data 2
Description: Bar Plots of Next Generation Sequencing

File Name: Supplementary Data 3
Description: Indel Distribution Plots of Next Generation Sequencing
