# Supplementary material for: Highly efficient multiplex human T cell engineering without double-strand breaks using Cas9 base editors
Source: Nat Commun. 2019 Nov 19;10:5222. doi: 10.1038/s41467-019-13007-6 (PMC6864045; doi:10.1038/s41467-019-13007-6)
Supplement: Supplementary file 6 — Source Data [file 41467_2019_13007_MOESM6_ESM.pdf]

nature research

Corresponding author(s): Beau Webber

Last updated by author(s): Jan 14, 2019

Reporting Summary

Nature Research wishes to improve the reproducibility of the work that we publish. This form provides structure for consistency and transparency in reporting. For further information on Nature Research policies, see Authors & Referees and the Editorial Policy Checklist.

Statistics

For all statistical analyses, confirm that the following items are present in the figure legend, table legend, main text, or Methods section.

n/a	Confirmed	
☐	☒	The exact sample size (n) for each experimental group/condition, given as a discrete number and unit of measurement
☐	☒	A statement on whether measurements were taken from distinct samples or whether the same sample was measured repeatedly
☐	☒	The statistical test(s) used AND whether they are one- or two-sided<br>*Only common tests should be described solely by name; describe more complex techniques in the Methods section.*
☒	☐	A description of all covariates tested
☐	☒	A description of any assumptions or corrections, such as tests of normality and adjustment for multiple comparisons
☐	☒	A full description of the statistical parameters including central tendency (e.g. means) or other basic estimates (e.g. regression coefficient) AND variation (e.g. standard deviation) or associated estimates of uncertainty (e.g. confidence intervals)
☐	☒	For null hypothesis testing, the test statistic (e.g. F, t, r) with confidence intervals, effect sizes, degrees of freedom and P value noted<br>*Give P values as exact values whenever suitable.*
☒	☐	For Bayesian analysis, information on the choice of priors and Markov chain Monte Carlo settings
☒	☐	For hierarchical and complex designs, identification of the appropriate level for tests and full reporting of outcomes
☐	☒	Estimates of effect sizes (e.g. Cohen's d, Pearson's r), indicating how they were calculated

Our web collection on statistics for biologists contains articles on many of the points above.

Software and code

Policy information about availability of computer code

Data collection	Amplicons were sequenced on an Illumina MiSeq using 2 x 300 paired end reads
Data analysis	Raw amplicon fastq data was analyzed using the CRISPR-DAV pipeline (PMID: 28961906) and available at https://github.com/pinetree1/crispr-dav. Output 'sample_snp.xlsx' and 'sample_len.xlsx' were compiled and visualized in R studio using the custom R markdown script "NGS_pdfout.Rmd" with the input file "sample_information.txt". Custom script and example file are available at https://github.com/stradcello/CRISPR-DAV_analysis.

For manuscripts utilizing custom algorithms or software that are central to the research but not yet described in published literature, software must be made available to editors/reviewers. We strongly encourage code deposition in a community repository (e.g. GitHub). See the Nature Research guidelines for submitting code & software for further information.

Data

Policy information about availability of data

All manuscripts must include a data availability statement. This statement should provide the following information, where applicable:
- Accession codes, unique identifiers, or web links for publicly available datasets
- A list of figures that have associated raw data
- A description of any restrictions on data availability

Next-generation sequencing reads will be deposited in the NCBI Sequence Read Archive database prior to publication.

Field-specific reporting

Please select the one below that is the best fit for your research. If you are not sure, read the appropriate sections before making your selection.

☒ Life sciences ☐ Behavioural & social sciences ☐ Ecological, evolutionary & environmental sciences

For a reference copy of the document with all sections, see nature.com/documents/nr-reporting-summary-flat.pdf

Life sciences study design

All studies must disclose on these points even when the disclosure is negative.

Sample size	No power analyses were performed prior to experiments to determine sample size.
Data exclusions	No data was excluded
Replication	All editing and functional experiments were replicated in at least two independent healthy human donors.
Randomization	Lymphocytes derived from each individual human donor were randomly and equally distributed across all experimental conditions.
Blinding	Where appropriate for functional studies, researchers conducting cytokine production and cell killing assays were not provided with treatment condition of samples.

Reporting for specific materials, systems and methods

We require information from authors about some types of materials, experimental systems and methods used in many studies. Here, indicate whether each material, system or method listed is relevant to your study. If you are not sure if a list item applies to your research, read the appropriate section before selecting a response.

Materials & experimental systems

n/a	Involved in the study	
☐	☒	Antibodies
☐	☐	Eukaryotic cell lines
☒	☐	Palaeontology
☒	☐	Animals and other organisms
☐	☒	Human research participants
☒	☐	Clinical data

Methods

n/a	Involved in the study	
☒	☐	ChIP-seq
☐	☒	Flow cytometry
☒	☐	MRI-based neuroimaging

Antibodies

Antibodies used

Target - Clone - Fluorophore - Vendor - Catalog Number

CD3 - UCHT1 - APC - BD Biosciences - 555335

β2-microglobulin - 2M2 - PE - BioLegend - 316306

β2-microglobulin - 2M2 - APC/Fire 750 - BioLegend - 316314

CD279 (PD-1) - EH12.2H7 - Brilliant Violet 421 - BioLegend - 329920

CD34 - QBEnd/10 - PE - Thermo Fisher Scientific - MA1-10205

Mouse IgG1, k Isotype Control - X40 - Brilliant Violet 421 - BD Biosciences - 562438

TNF - TMAb11 - APC - eBioscience - 17-7349-82

IL2 - MQ1-17H12 - PECy7 - Invitrogen - 25-7029-42

IFNg - 4S.B3 - eFluor 450 - eBioscience - 48-7319-42

CD3 - SK7 - Brilliant Violet 395 - BD Horizon - 564001

CD4 - SK3 - Brilliant Violet 496 - BD Horizon - 564651

CD8 - SK1 - Brilliant Violet 805 - BD Horizon - 564912

CD45ro - UCHL11 - Brilliant Violet 711 - BioLegend - 304236

CD27 - O323 - Brilliant Violet 605 - BioLegend - 302830

beta-actin - D6A8 - Cell Signaling - 8457S

Cas9 - 7A9-3A3, Cell Signaling - 14697S

Validation	Antibody validations were performed by suppliers

Eukaryotic cell lines

Policy information about cell lines

Cell line source(s)	Raji (ATCC CCL-86) and K562 (ATCC CCL-243) cell lines were obtained from ATCC.
Authentication	Cell lines used were periodically authenticated by autosomal STR profiling suitable for verification using reference databases performed by University of Arizona Genetics Core.
Mycoplasma contamination	Cell lines (M202, M257, M407) were periodically tested for mycoplasma contamination and tested negative.
Commonly misidentified lines (See ICLAC register)	No commonly misidentified lines were used in the study.

Human research participants

Policy information about studies involving human research participants

Population characteristics	Healthy human blood donors were male or female and between the ages of 21 and 50.
Recruitment	Leukapheresis products were obtained from healthy donors at Memorial Blood Center with informed consent.
Ethics oversight	Human blood was utilized under University of Minnesota Institutional Review Board (IRB) approval (#1602E84302)

Note that full information on the approval of the study protocol must also be provided in the manuscript.

Flow Cytometry

Plots

Confirm that:

☒ The axis labels state the marker and fluorochrome used (e.g. CD4-FITC).

☒ The axis scales are clearly visible. Include numbers along axes only for bottom left plot of group (a 'group' is an analysis of identical markers).

☒ All plots are contour plots with outliers or pseudocolor plots.

☒ A numerical value for number of cells or percentage (with statistics) is provided.

Methodology

Sample preparation	Primary human T cells were washed once with 2ml FACS buffer (0.2% human AB serum and 1mM EDTA in PBS) prior to cell surface staining. Once cells were pelleted, buffer was poured off and antibodies were added to the remaining buffer and cells incubated at 4C for 30 minutes in the dark. Cells were washed once with 2ml FACS buffer and resuspended in 200ul for analysis on the flow cytometer.
Instrument	Analysis was performed on a BD LSR II or LSRFortessa
Software	Flow cytometric data analysis was performed using FlowJo v9 and v10. Analysis and graphical representation of multiplex knockout and multiplex cytokine production were conducted using the data analysis program Simplified Presentation of Incredibly Complex Evaluations (SPICE; version 5.3) (ref).<br>Ref: Roederer M., J. L. Nozzi, M. C. Nason. 2011. SPICE: exploration and analysis of post-cytometric complex multivariate datasets. Cytometry A 79: 167–174.
Cell population abundance	No flow sorting was used in the current study.
Gating strategy	Lymphocytes were first gated on SSC-A vs FSC-A. Single cells were then gated on FSC-H vs FSC-A, followed by live cells on SSC-A

Gating strategy	vs Viability Dye. Finally, CD3, B2M, PD-1, and CD34 positive cells were gated from live cell populations.

☒ Tick this box to confirm that a figure exemplifying the gating strategy is provided in the Supplementary Information.
